# DNA and RNA-sequence based GWAS highlights membrane-transport genes as key modulators of milk lactose content

**DOI:** 10.1186/s12864-017-4320-3

**Published:** 2017-12-15

**Authors:** Thomas J. Lopdell, Kathryn Tiplady, Maksim Struchalin, Thomas J. J. Johnson, Michael Keehan, Ric Sherlock, Christine Couldrey, Stephen R. Davis, Russell G. Snell, Richard J. Spelman, Mathew D. Littlejohn

**Affiliations:** 1 Research and Development, Livestock Improvement Corporation, Ruakura Road, Newstead, Hamilton, New Zealand; 2 School of Biological Sciences, University of Auckland, Symonds Street, Auckland, New Zealand

**Keywords:** QTL mapping, GWAS, Milk, Lactose, RNA sequencing, Genome sequencing

## Abstract

**Background:**

Lactose provides an easily-digested energy source for neonates, and is the primary carbohydrate in milk in most species. Bovine lactose is also a key component of many human food products. However, compared to analyses of other milk components, the genetic control of lactose has been little studied. Here we present the first GWAS focussed on analysis of milk lactose traits.

**Results:**

Using a discovery population of 12,000 taurine dairy cattle, we detail 27 QTL for lactose concentration and yield, and subsequently validate the effects of 26 of these loci in a distinct population of 18,000 cows. We next present data implicating causative genes and variants for these QTL. Fine mapping of these regions using imputed, whole genome sequence-resolution genotypes reveals protein-coding candidate causative variants affecting the *ABCG2*, *DGAT1*, *STAT5B*, *KCNH4*, *NPFFR2* and *RNF214* genes. Eleven of the remaining QTL appear to be driven by regulatory effects, suggested by the presence of co-locating, co-segregating eQTL discovered using mammary RNA sequence data from a population of 357 lactating cows. Pathway analysis of genes representing all lactose-associated loci shows significant enrichment of genes located in the endoplasmic reticulum, with functions related to ion channel activity mediated through the *LRRC8C*, *P2RX4*, *KCNJ2* and *ANKH* genes. A number of the validated QTL are also found to be associated with additional milk volume, fat and protein phenotypes.

**Conclusions:**

Overall, these findings highlight novel candidate genes and variants involved in milk lactose regulation, whose impacts on membrane transport mechanisms reinforce the key osmo-regulatory roles of lactose in milk.

**Electronic supplementary material:**

The online version of this article (doi:10.1186/s12864-017-4320-3) contains supplementary material, which is available to authorized users.

## Background

Lactose is the most abundant carbohydrate in milk, providing an energy source for neonates that is more easily digestible than other major milk components such as fats and proteins. Concentrations of carbohydrates in milk vary widely between species. In some seals, almost no carbohydrate is present, where functional inactivation of the *α*-lactalbumin gene (*LALBA*), a key lactose synthesis component, helps prevent involution of the gland during long foraging trips at sea [1]. By contrast, milk in prosimian primates, for example lemurs, is high in lactose (up to 8.9%; [2]). Human and cow milks are intermediate between these two extremes, at 6.7% [3] and 5.1% [4]. In cows, lactose yield (LY) is highly correlated (both phenotypically and genetically) with milk volume, fat yield, and protein yield [5, 6], while lactose concentration (LC) is negatively correlated with yield traits.

Lactose is synthesised from UDP-galactose and glucose in the epithelial cells of the lactating mammary gland. This process is catalysed by a complex, known collectively as lactose synthase, comprised of two protein subunits: the catalytic *β*1,4-galactosyltransferase-I (*B4GALT1*) and the regulatory unit *LALBA* [7]. *B4GALT1* resides permanently on the Golgi apparatus, where its standard function is to attach UDP-galactose residues to the terminal N-acetylglucosamine of glycans in the formation of glycoproteins and glycolipids [7]. LALBA is a major protein component of whey, requiring a high level of *LALBA* expression in mammary epithelial cells during lactation. The presence of LALBA in these cells induces a conformational change in the B4GALT1 enzyme which alters its specificity from N-acetylglucosamine to glucose. This structural change triggers the synthesis of lactose [8]. Lactose is then secreted into milk via secretory vesicles, along with milk proteins and ions. The presence of lactose in these vesicles affects their osmolarity, causing the uptake of water, which is also secreted into the milk [9]. Since milk is isosmotic with blood, this mechanism generates a strong correlation (0.99 [4]) between lactose production and milk volume, with lactose content helping to define the unique milk composition characteristics of individual species.

The economic importance of dairy cattle has driven the collection of very large genotypic and phenotypic datasets that serve genomic prediction-based breeding programmes. These same data can be used opportunistically to conduct quantitative trait locus (QTL) mapping, and thus cattle have become one of the most powerful and commonly investigated species for studying genetic aspects of mammary biology and lactation [10, 11]. Numerous major effect genes and mutations have now been described, impacting diverse milk yield and composition phenotypes (for example *DGAT1*[12], *ABCG2* [13], *GPAT4*[14], and *MGST1* [15]). Historically, lactose has been little studied, due in part to the strong correlation between lactose yield and milk volume, and that in New Zealand at least, dairy cattle breeding objectives do not place a direct, commercial value on lactose. More recently, the New Zealand dairy industry has shifted focus towards producing dried milk powder for export, causing deficits in lactose availability [16] and providing impetus for research into this trait. Although quantitative genetic parameters for lactose have been published [5, 6], to our knowledge no studies have reported genome-wide analyses focussed on investigation of lactose traits. The aim of the current work was to conduct GWAS analysis for lactose concentration (LC) and yield (LY) traits in New Zealand dairy cattle. Subsequently, we aimed to identify candidate causative genes underlying discovered QTL, leveraging sequence-based datasets to impute, fine-map and investigate the regulatory architecture of lactose-associated loci.

## Results

### Lactose phenotypes and heritibilities

The lactose concentration (LC) phenotype was defined as the percentage of the milk volume that consisted of lactose, as quantified using calibrations of Fourier transform infrared spectroscopy against a lactose monohydrate standard. The lactose yield (LY) phenotype comprised the LC percentage multiplied by the total daily milk volume expressed in units of kg/day. Genetic analysis was undertaken in several different populations. These included 12,000 outbred New Zealand dairy cows composed of Holstein-Friesians (HF), Jerseys, and their crosses (the ‘QTL discovery’ set), a distinct group of 18,000 animals of similar breed composition (the ‘QTL validation’ set), and two purebred cohorts of 14,857 HF and 8 995 Jersey cows (see Methods for further details and breed definitions). After all phenotype adjustments (see Methods), the mean LC and LY phenotype values for the combined discovery and validation animals (*N*=30,000) were 5.146 and 0.833 respectively (Table 1). For the purebreds within this dataset, HF animals had higher mean LY phenotypes (0.851) than Jersey animals (0.809), and Jersey animals showed slightly higher LC values (Table 1). Narrow sense heritabilities are also indicated in Table 1. The LY heritability was 0.253 for the combined population (*N*=30,000 animals), with estimates also similar between the two breeds (Table 1). The LC heritability was 0.557 in the combined population, though lower for Jersey animals (*h* ^2^=0.450; Table 1), presumably reflecting the lower genetic diversity in this breed. Genotypic principle component analysis was used to visualise the genetic structure of the combined discovery and validation population. Additional file 1: Figure S1 shows the first two principal components of the population plotted by breed.

**Table 1:** Summary statistics for lactose concentration and yield phenotypes

Breed	N	Phenotype	Mean±SD	$\sigma ^{2}_{P}$	$\sigma ^{2}_{A}$	*h* ^2^
All	30,000	LY (kg/day)	0.830±0.119	0.0147±0.0001	0.0037±0.0001	0.253±0.008
		LC (%)	5.146±0.130	0.0189±0.0002	0.0105±0.0003	0.557±0.008
Jersey	3 998	LY	0.809±0.106	0.0112±0.0003	0.0030±0.0003	0.269±0.025
		LC	5.152±0.124	0.0156±0.0004	0.0070±0.0005	0.450±0.025
Holstein-Friesian	8 292	LY	0.851±0.132	0.0176±0.0003	0.0041±0.0003	0.236±0.017
		LC	5.135±0.134	0.0189±0.0004	0.0105±0.0005	0.557±0.016

Summary statistics for the lactose concentration (LC) and lactose yield (LY) phenotypes, calculated for 30,000 cows. Phenotype means are shown with standard deviations. The phenotypic variance ($\sigma ^{2}_{P}$), additive genetic variance ($\sigma ^{2}_{A}$), and narrow-sense heritability (*h* ^2^) are shown with standard errors. The Jersey and Holstein-Friesian subsets included those animals where at least 15/16 of the animal’s ancestry is from the appropriate breed

### SNP-chip-based genome-wide association analysis

**Fig. 1:**
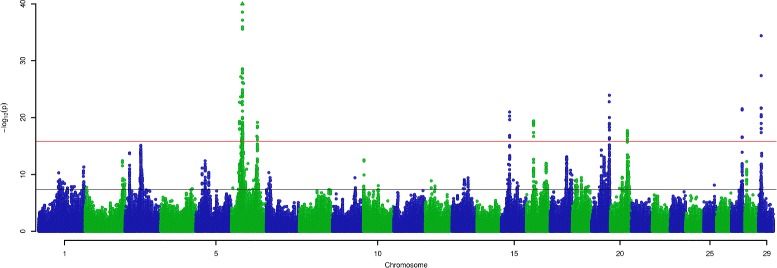
Manhattan plot of QTL locations for lactose concentration. The X-axis shows the positions of 1.1 million variants across the 29 autosomes in the UMD 3.1 *Bos taurus* reference genome; the Y-axis shows the negative log of the *p*-values calculated for each variant. Variants illustrated using a triangle sit beyond the limit of the Y-axis. The black line shows the nominal significance threshold incorporating a Bonferroni correction for multiple hypothesis testing. The red line shows the combined inflation and multiple testing-adjusted threshold

**Fig. 2:**
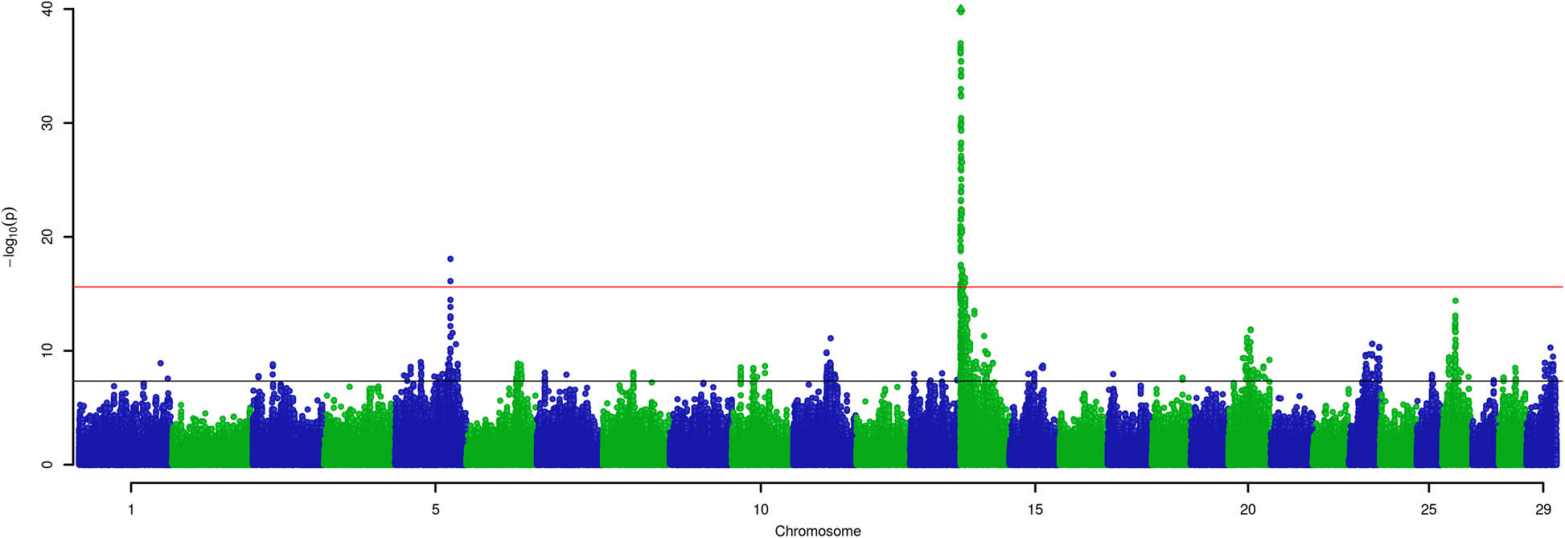
Manhattan plot showing QTL locations for lactose yield. The X-axis shows the positions of 1.1 million variants across the 29 autosomes in the UMD 3.1 *Bos taurus* reference genome; the Y-axis shows the negative log of the *p*-values calculated for each variant for a lactose yield QTL. Variants illustrated using a triangle sit beyond the limit of the Y-axis. The black and red lines as per Fig. 1 legend

Genome-wide association mapping was conducted using 1,091,000 variants in conjunction with LC and LY phenotypes in the discovery population (*N*=12,000), applying generalised least-squares models that accounted for population structure and pedigree (see “Methods” section). Analysis of the LC phenotype revealed genome-wide significant effects on 22 of the 29 autosomes (Fig. 1). Applying a more conservative, additional inflation adjusted threshold of 1.61×10^−16^ yielded eight discrete loci on seven chromosomes (Fig. 1). Twenty chromosomes had significant effects for LY, though compared to the LC trait, the genetic architecture was comprised of fewer highly associated regions, with only two loci passing the more stringent, inflation adjusted threshold of 2.50×10^−16^ (Fig. 2).

### Fine-mapping of lactose loci using imputed whole-genome sequence data

To fine-map lactose QTL, we imputed whole-genome sequence-resolution data into the highest priority regions to attempt to map putative causative variants directly. For these analyses, we focussed on the largest QTL, applying an arbitrary threshold to include loci where the top tag-SNP had − log10(*p*-value)  > 1.5× the nominal, Bonferroni-adjusted threshold. These criteria resulted in 22 loci for LC and five additional loci for LY (Table 2). Importantly, this list included regions of biological interest that would otherwise have been lost using the stricter, inflation-adjusted threshold, comprising genes and loci with previously demonstrated roles in milk composition regulation and/or obvious mechanistic roles in lactose synthesis (e.g. chr19:43Mbp, *STAT5A* and *STAT5B* [17]; chr5:32Mbp, *LALBA* [7]; chr20:32Mbp, *GHR* [18]).

**Table 2:** QTL locations for lactose QTL in bovine milk

			HD+RNA			Sequence			
Pheno	Chr	Mbp	QTL *P*Val	Tag Var	Var effect	QTL *P*Val	Tag Var	SNP effect	Validation
LC	1	154.14	4.98×10^−12^	rs43280825	−0.0143±0.0021	2.25×10^−13^	rs43282035	−0.0155±0.0021	3.09×10^−24^
LC	2	127.64	3.93×10^−13^	rs209230743	−0.0160±0.0022	1.37×10^−13^	rs208702482	−0.0164±0.0022	2.84×10^−19^
LC	3	15.52	2.25×10^−14^	rs210326427	−0.0136±0.0018	1.26×10^−14^	rs211336034	−0.0137±0.0018	3.82×10^−22^
LC	3	53.84	7.86×10^−16^	rs136784524	−0.0152±0.0019	6.02×10^−16^	rs109613143	−0.0153±0.0019	3.52×10^−23^
LC	5	21.14	7.55×10^−12^	rs133198838	0.0128±0.0019	3.46×10^−12^	rs377953581	0.0130±0.0019	0.210
LC	5	31.56	4.18×10^−13^	rs109681546	0.0138±0.0019	1.40×10^−14^	rs137534989	−0.0140±0.0018	6.90×10^−23^
LC	5	44.16	4.25×10^−11^	rs378337086	0.0134±0.0020	3.51×10^−11^	rs383349320	0.0134±0.0020	2.91×10^−10^
LC	6	37.76	**1** **.** **9** **5** **×** **1**0^−221^	rs381223633	0.2656±0.0084	<1.05×10^−308^	rs43702337	−0.3379±0.0090	<1.05×10^−308^
LC	6	89.04	**6** **.** **9** **6** **×** **1**0^−20^	rs110432804	−0.0208±0.0023	1.25×10^−20^	rs478177439	−0.0213±0.0023	1.73×10^−29^
LC	7	8.77	4.55×10^−11^	rs137176653	0.0115±0.0017	5.92×10^−11^	rs210686953	0.0120±0.0017	4.02×10^−11^
LC	10	2.14	2.94×10^−13^	rs137007518	−0.0143±0.0020	2.63×10^−13^	rs137774567	−0.0145±0.0020	6.63×10^−10^
LC	15	28.36	**1** **.** **0** **6** **×** **1**0^−21^	rs209807357	−0.0281±0.0029	2.70×10^−21^	rs211369213	−0.0278±0.0029	2.02×10^−45^
LC	16	24.99	**3** **.** **9** **6** **×** **1**0^−20^	rs109379517	−0.0183±0.0020	1.25×10^−21^	rs109379517	−0.0192±0.0020	1.60×10^−29^
LC	16	67.77	1.11×10^−12^	rs137354702	0.0124±0.0017	1.11×10^−12^	rs380467601	0.0128±0.0018	1.43×10^−23^
LC	17	56.47	7.97×10^−14^	rs109194382	−0.0142±0.0019	7.40×10^−14^	rs134672113	−0.0142±0.0019	8.35×10^−07^
LC	19	33.51	5.19×10^−15^	rs109898712	−0.0149±0.0019	1.01×10^−15^	rs109514832	−0.0149±0.0019	2.14×10^−38^
LC	19	42.99	9.38×10^−14^	rs133057731	0.0233±0.0031	6.85×10^−14^	rs517084099	0.0248±0.0033	8.68×10^−31^
LC	19	61.13	**1** **.** **2** **3** **×** **1**0^−24^	rs41923843	−0.0187±0.0018	1.23×10^−24^	rs41923843	−0.0187±0.0018	1.63×10^−34^
LC	20	58.45	**2** **.** **0** **1** **×** **1**0^−18^	rs135594014	−0.0175±0.0020	9.37×10^−19^	rs135934727	−0.0178±0.0020	1.34×10^−27^
LC	27	36.21	**2** **.** **8** **4** **×** **1**0^−22^	rs208675276	0.0173±0.0018	2.34×10^−22^	rs209987511	0.0175±0.0018	2.71×10^−41^
LC	28	6.56	5.47×10^−13^	rs110674951	0.0133±0.0019	5.47×10^−13^	rs110674951	−0.0138±0.0019	3.90×10^−35^
LC	29	9.61	**4** **.** **0** **3** **×** **1**0^−35^	rs471922429	0.0252±0.0020	5.96×10^−36^	rs378183369	0.0245±0.0020	2.32×10^−90^
LY	5	93.94	**8** **.** **5** **4** **×** **1**0^−19^	rs134637616	0.0175±0.0020	4.35×10^−22^	rs211210569	0.0183±0.0019	1.09×10^−26^
LY	11	63.45	7.94×10^−12^	rs210726760	0.0133±0.0019	2.65×10^−11^	rs210726760	0.0130±0.0019	3.60×10^−9^
LY	14	1.77	**2** **.** **0** **0** **×** **1**0^−138^	rs134364612	−0.0421±0.0017	1.09×10^−137^	rs109234250	−0.0420±0.0017	9.43×10^−179^
LY	20	31.69	7.18×10^−12^	rs110728486	−0.0114±0.0017	6.54×10^−12^	rs208881195	−0.0114±0.0017	7.52×10^−9^
LY	26	22.96	4.06×10^−15^	rs110996268	0.0150±0.0019	1.30×10^−15^	rs208730573	0.0152±0.0019	1.50×10^−6^

Loci identified using GWAS on the lactose concentration (LC) and lactose yield (LY) phenotypes. Chromosomes and base positions are from the UMD 3.1 bovine reference genome, with positions in millions of bases. The *p*-values are for the variant at each locus that was most strongly associated with the phenotype: values in bold pass genome-wide significance after both Bonferroni correction and adjustment for inflation. Allele effects are relative to the allele present in the reference bovine genome

For each of the 27 target regions, 1Mbp intervals of sequence were imputed using Beagle software (see “Methods” section), centred on the top tag-SNP identified from the genome-wide analysis. Association analysis of imputed sequence was conducted as described for analysis using SNP-chip content. Exploded-view (1Mbp), sequence-resolution Manhattan plots for all 27 regions are shown in Additional file 2: Figure S2. For 22 of the 27 QTL, genome sequence based analysis yielded an increase in the strength of association compared to SNP-chip and RNA sequence based content, and in the case of the chr6:37.76Mbp locus, this increase was substantial (Table 2). Using the top-associated variant as a proxy for each of these loci, the 22 high-priority LC QTL explained 21.1% of the phenotypic variance for this trait. For LY, the top 5 tag-variants together explained 5.0% of the phenotypic variance.

### Validation of the largest lactose QTL

To validate the QTL observed in our initial genome-wide screen, and obtain more robust estimates of likely effect sizes, we conducted a validation study of the 27 implicated regions. The validation sample of 18,000 lactating cows was imputed for the 27 tag-variants of interest, comprising the most highly associated polymorphisms from sequence-based fine mapping of the prioritised regions. These animals were of similar breed composition to the 12,000 animals in the discovery set, selected to avoid potential problems with varying allele frequencies across breeds. Association analysis validated 26 of the 27 QTL, with only the chr5:21.14Mbp locus failing to replicate (Table 2 and Additional file 3: Table S3). For the remaining regions, the 21 LC tag-variants explained 17.5% of the phenotypic variance, with the 5 LY loci together explaining 4.4% of the variance.

#### Within-breed analyses

Since genome-wide analysis was conducted using mixed breed animals, and QTL might represent false positive associations resulting from population stratification, we also examined the impacts of QTL-tag SNPs within breed. Tag-variant minor allele frequencies (MAFs) for the discovery and validation animal sets are indicated in Table 3. Several variants had markedly different frequencies between breeds. Referencing the Jersey breed in the discovery population, these included: rs208702482 (0.094 vs 0.277), rs43702337 (0.002 vs 0.008), rs478177439 (0.005 vs 0.362), rs110674951 (0.092 vs 0.658), rs211210569 (0.023 vs 0.535), rs210726760 (0.056 vs 0.330), and rs208730573 (0.012 vs 0.495). For association analysis, we took the purebred animals referenced above (8 292 HF and 3 998 Jerseys present in the combined discovery and validation sets), and augmented these with an additional 6 656 HF and 4 997 Jersey animals for which we also had genotype and phenotype records (total *N*=14,857 and 8 995 HF and Jerseys respectively). Imputing tag-variants and conducting association analysis using the same approaches described above, these analyses showed that, of the 26 previously validated QTL, all surpassed a pointwise significance threshold of *P*<0.05 in at least one breed, and 24 of 26 passed a genome-wide Bonferroni threshold of *P*=4.58×10^−8^ (Table 4). Importantly, aside from a single locus that showed highly significant, yet opposite allelic effects between breeds (chr19:42.99Mbp), the sign of effect for all other loci was the same between breeds, and agreed with the effects estimated in the mixed breed populations. These results suggested that, although some inflation was present in the genome-wide results, breed stratification effects were unlikely to be a major source of confounding for the largest QTL detected in our study.

**Table 3:** Minor allele frequencies for lactose QTL tag variants

				Discovery		Validation			
Pheno	Chr	Mbp	Tag Var	All (*n*=12,000)	HF (*n*=3 704)	Je (*n*=1 648)	All (*n*=18,000)	HF (*n*=4 588)	Je (*n*=2 350)
LC	1	154.14	rs43282035	*0.226	*0.226	*0.209	*0.231	*0.211	*0.236
LC	2	127.64	rs208702482	0.203	0.277	0.094	0.204	0.281	0.092
LC	3	15.52	rs211336034	0.449	0.440	0.465	0.454	0.448	0.456
LC	3	53.84	rs109613143	0.365	*0.489	0.190	0.377	*0.483	0.189
LC	5	21.14	rs377953581	0.444	0.264	*0.291	0.464	0.291	*0.304
LC	5	31.56	rs137534989	*0.493	*0.368	0.324	*0.483	*0.341	0.326
LC	5	44.16	rs383349320	0.253	0.176	0.364	0.247	0.169	0.352
LC	6	37.76	rs43702337	0.008	0.008	0.002	0.006	0.007	0.003
LC	6	89.04	rs478177439	0.215	0.362	0.005	0.207	0.360	0.010
LC	7	8.77	rs210686953	0.450	0.458	0.452	0.448	0.460	0.446
LC	10	2.14	rs137774567	0.302	0.452	0.100	0.304	0.447	0.089
LC	15	28.36	rs211369213	0.096	0.151	0.008	0.084	0.129	0.006
LC	16	24.99	rs109379517	0.303	0.405	0.178	0.285	0.388	0.157
LC	16	67.77	rs380467601	0.406	0.411	0.364	0.403	0.420	0.352
LC	17	56.47	rs134672113	0.375	*0.462	0.161	0.366	0.484	0.173
LC	19	33.51	rs109514832	*0.351	*0.258	0.486	*0.385	*0.328	0.488
LC	19	42.99	rs517084099	0.088	0.072	0.120	0.106	0.114	0.122
LC	19	61.13	rs41923843	*0.338	*0.326	*0.351	*0.327	*0.321	*0.349
LC	20	58.45	rs135934727	0.242	0.193	0.313	0.238	0.180	0.317
LC	27	36.21	rs209987511	0.367	0.291	*0.496	0.387	0.271	*0.450
LC	28	6.56	rs110674951	*0.447	0.342	*0.092	*0.438	0.387	*0.116
LC	29	9.61	rs378183369	0.250	0.310	0.198	0.268	0.326	0.222
LY	5	93.94	rs211210569	0.320	*0.465	0.023	0.324	*0.468	0.029
LY	11	63.45	rs210726760	0.229	0.330	0.056	0.229	0.367	0.054
LY	14	1.77	rs109234250	*0.408	0.466	*0.225	*0.407	0.462	*0.207
LY	20	31.69	rs208881195	*0.458	0.401	*0.248	*0.499	0.329	*0.259
LY	26	22.96	rs208730573	0.292	0.495	0.012	0.275	0.472	0.013

Minor allele frequencies for tag variants from WGS, for discovery and validation set cows. Frequencies for each animal set are shown in total and for pure-bred subsets. Cows are defined as belonging to the Holstein-Friesian (HF) or Jersey (Je) breed if 15/16 of their ancestry is recorded to that breed. Cases where the minor allele is the reference allele are marked with an asterisk (*), in the remaining cases, the minor allele is the alternative allele

**Table 4:** Allele effects for lactose QTL tag variants in Holstein-Friesian and Jersey Cows

				Holstein-Friesian (*n*=14,857)		Jersey (*n*=8,995)	
Pheno	Chr	Mbp	Tag variant	Beta±SE	*P*Val	Beta±SE	*P*Val
LC	1	154.14	rs43282035	−0.014±0.002	**1** **.** **7** **7** **×** **1**0^−12^	−0.016±0.002	**3** **.** **5** **9** **×** **1**0^−11^
LC	2	127.64	rs208702482	−0.019±0.002	**2** **.** **2** **6** **×** **1**0^−24^	−0.013±0.003	1.04×10^−4^
LC	3	15.52	rs211336034	−0.015±0.002	**1** **.** **9** **1** **×** **1**0^−18^	−0.018±0.002	**1** **.** **7** **4** **×** **1**0^−19^
LC	3	53.84	rs109613143	−0.020±0.002	**2** **.** **0** **6** **×** **1**0^−33^	−0.001±0.002	0.595
LC	5	21.14	rs377953581	0.006±0.002	6.30×10^−4^	0.002±0.002	0.300
LC	5	31.56	rs137534989	−0.014±0.002	**6** **.** **8** **5** **×** **1**0^−15^	−0.010±0.002	2.25×10^−7^
LC	5	44.16	rs383349320	0.009±0.002	2.37×10^−5^	0.005±0.002	0.0205
LC	6	37.76	rs43702337	−0.329±0.009	**<** **1** **.** **0** **5** **×** **1**0^−308^	−0.294±0.019	**1** **.** **5** **1** **×** **1**0^−54^
LC	6	89.04	rs478177439	−0.022±0.002	**6** **.** **0** **7** **×** **1**0^−36^	−0.018±0.009	0.0417
LC	7	8.77	rs210686953	0.005±0.002	7.02×10^−3^	0.015±0.002	**6** **.** **8** **1** **×** **1**0^−14^
LC	10	2.14	rs137774567	−0.012±0.002	**2** **.** **5** **4** **×** **1**0^−12^	−0.020±0.003	**2** **.** **5** **5** **×** **1**0^−9^
LC	15	28.36	rs211369213	−0.034±0.002	**1** **.** **2** **0** **×** **1**0^−43^	−0.026±0.009	5.07×10^−3^
LC	16	24.99	rs109379517	−0.018±0.002	**6** **.** **4** **7** **×** **1**0^−24^	−0.015±0.003	**4** **.** **8** **5** **×** **1**0^−9^
LC	16	67.77	rs380467601	0.015±0.002	**3** **.** **9** **2** **×** **1**0^−20^	0.009±0.002	1.27×10^−5^
LC	17	56.47	rs134672113	−0.012±0.002	**3** **.** **7** **0** **×** **1**0^−13^	−0.011±0.003	2.07×10^−5^
LC	19	33.51	rs109514832	−0.023±0.002	**6** **.** **0** **5** **×** **1**0^−37^	−0.009±0.002	4.46×10^−6^
LC	19	42.99	rs517084099	−0.018±0.003	**1** **.** **2** **2** **×** **1**0^−10^	0.022±0.003	**2** **.** **4** **9** **×** **1**0^−12^
LC	19	61.13	rs41923843	−0.025±0.002	**4** **.** **4** **7** **×** **1**0^−46^	−0.007±0.002	3.47×10^−4^
LC	20	58.45	rs135934727	−0.011±0.002	5.46×10^−7^	−0.019±0.002	**2** **.** **7** **2** **×** **1**0^−20^
LC	27	36.21	rs209987511	0.018±0.002	**2** **.** **5** **8** **×** **1**0^−22^	0.021±0.002	**1** **.** **3** **2** **×** **1**0^−26^
LC	28	6.56	rs110674951	−0.013±0.002	**4** **.** **6** **4** **×** **1**0^−15^	−0.017±0.003	**7** **.** **3** **7** **×** **1**0^−9^
LC	29	9.61	rs378183369	0.029±0.002	**1** **.** **4** **8** **×** **1**0^−58^	0.023±0.002	**8** **.** **8** **6** **×** **1**0^−22^
LY	5	93.94	rs211210569	0.021±0.002	**9** **.** **1** **6** **×** **1**0^−37^	0.016±0.004	1.91×10^−4^
LY	11	63.45	rs210726760	0.010±0.002	**9** **.** **8** **1** **×** **1**0^−9^	0.005±0.004	0.205
LY	14	1.77	rs109234250	−0.046±0.002	**9** **.** **0** **3** **×** **1**0^−170^	−0.035±0.002	**1** **.** **0** **8** **×** **1**0^−61^
LY	20	31.69	rs208881195	−0.010±0.002	5.15×10^−8^	−0.005±0.002	5.97×10^−3^
LY	26	22.96	rs208730573	0.008±0.002	1.12×10^−6^	0.042±0.006	**1** **.** **6** **1** **×** **1**0^−13^

Allele effects for each WGS tag variant for Holstein-Friesian and Jersey cows, assuming an additive model. *P*-values in bold font indicate tag variants which pass the genome-wide Bonferroni-corrected threshold (4.58×10^−8^) for that breed. All but three variants pass Bonferroni in at least one breed; one of these three tags the QTL which failed validation. The direction of the allele effect is the same in both breeds for all but one (rs517084099) of the variants. Allele effects are relative to the allele present in the reference bovine genome. Phenotypes are lactose concentration (top) and lactose yield (bottom)

### Analysis of other lactation traits

We have previously observed sharing of genetic signals across different lactation traits [15], so to test whether lactose-associated loci showed pleiotropic effects, we conducted analysis of milk volume, fat, and protein phenotypes in conjunction with the 26 validated tag-variants. Phenotypes for the 12,000 discovery set of cows were derived from herd test data using the same approach outlined for lactose traits. Twenty-two of the 26 validated QTL passed a pointwise significance threshold of *P*<0.05 in at least one other trait, and 13 loci were significant for at least one trait at the Bonferroni threshold of P=4.58×10^−8^ (Additional file 4: Table S4). Nineteen of the 26 loci were significant (*P*<0.05) for more than one additional trait, and two loci were significant across all additional traits (chr14:1.77Mbp and chr20:31.69Mbp).

### Positional candidate genes and variants

We employed two approaches to attempt to identify causative genes and variants underlying the list of 26 validated lactose QTL, using methods that inform on potential protein function-based effects and regulatory mechanisms. For the first, bioinformatic annotation tools were used to predict functional consequences of WGS-resolution association data. For the second, we leveraged a large RNA sequence resource to look for evidence of genotypically-driven gene expression changes co-locating with lactose QTL.

#### Protein function-based prediction of candidate causative variants

**Fig. 3:**
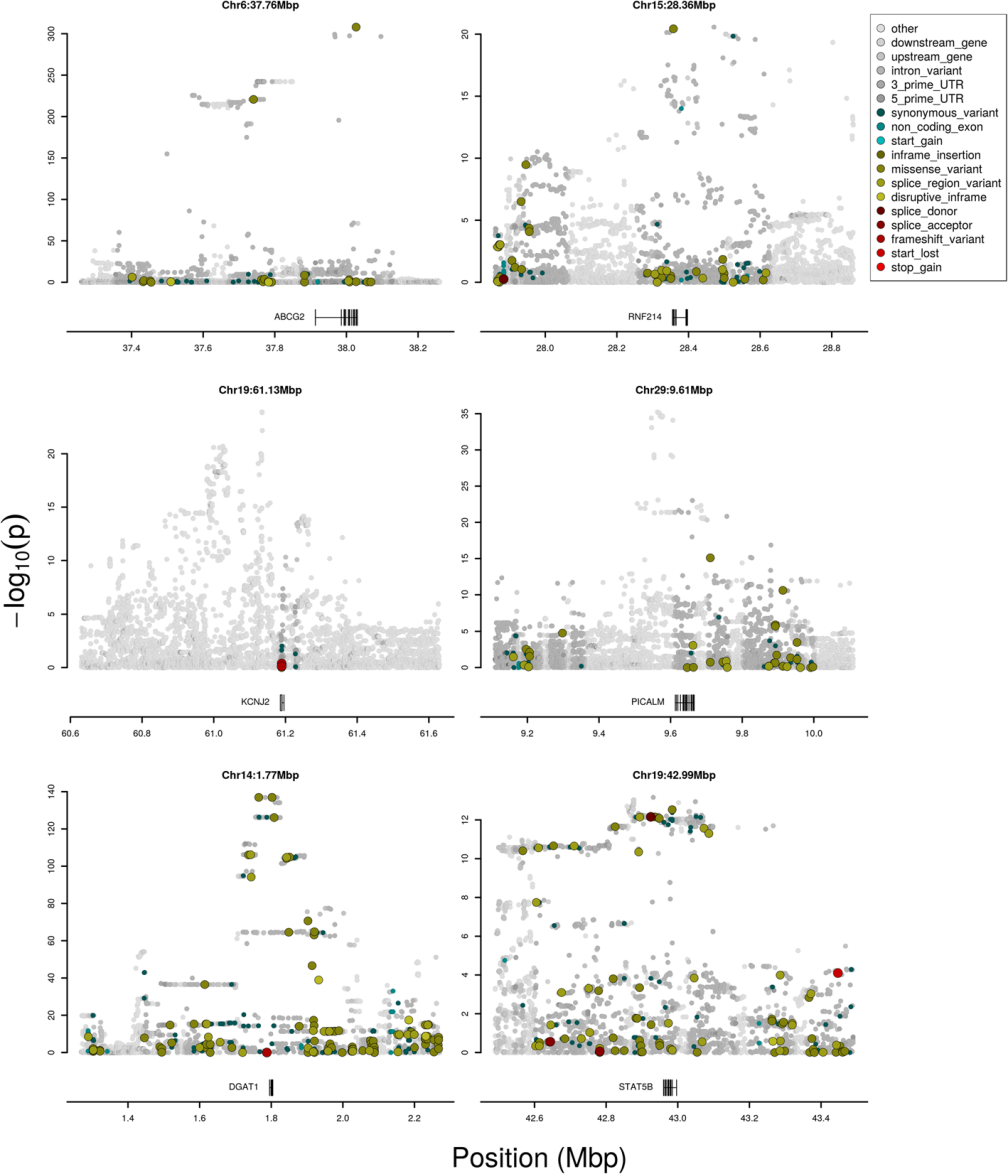
WGS QTL coloured by SNP effect predictions. Six example 1Mbp windows of imputed WGS resolution associations centred on five QTL for LC and one QTL for LY (Chr14:1.77Mbp). Variants are coloured by predicted variant effect

To assess the candidacy of strongly associated variants in the context of their predicted impact on protein sequence and structures, we annotated all variants in each 1Mbp of interest using SNPEff [19] and the Variant Effect Predictor [20]. Examples of QTL annotated with functional predictions are shown in Fig. 3. Since errors in genotyping, phenotyping and imputation are expected to impact the association rankings of candidate variants, we also used a linkage disequilibrium (LD)-based approach to prioritise variants, acknowledging that true functional polymorphisms will not necessarily be the most significantly associated variants [21]. Using an LD threshold of *R* ^2^>0.9, Table 5 shows those loci where at least one protein-coding mutation was predicted in strong linkage disequilibrium with the most strongly associated variant from sequence-based analysis.

**Table 5:** Peak variants with protein sequence mutations

Locus	Phenotype	ID	Class	LD	Gene	Description	VEP	SIFT
chr3:15.52Mbp	LC	rs109816684	Splice Region	0.997	*SLC50A1*	c.282+7G>A	L	—
chr6:37.76Mbp	LC	rs43702337	Missense	1.000	*ABCG2*	Y581S	M	0.00 (D)
chr6:89.04Mbp	LC	rs110326785	Missense	0.987	*NPFFR2*	E392K	M	0.56 (T)
chr15:28.36Mbp	LC	rs208325660	Missense	0.967	*RNF214*	G105E	M	0.07 (TLC)
chr16:24.99Mbp	LC	rs110899826	Missense	0.986	*MARC1*	P194R	M	0.27 (T)
chr16:24.99Mbp	LC	rs109896036	Splice Region	0.986	*MARC1*	c.628-5C>T	L	—
chr19:42.99Mbp	LC	rs377779402	Splice Donor	1.000	*KCNH4*	c.2663+2T>C	H	—
chr19:42.99Mbp	LC	rs209410283	Missense	1.000	*KCNH4*	S136R	M	0.13 (T)
chr19:42.99Mbp	LC	rs211002889	Missense	1.000	*GHDC*	P335R	M	0.55 (T)
chr19:42.99Mbp	LC	rs208379505	Missense	1.000	*GHDC*	P233A	M	0.03 (D)
chr19:42.99Mbp	LC	rs207799702	Splice Region	0.999	*KAT2A*	c.700-7C>G	L	—
chr19:42.99Mbp	LC	rs211108888	Splice Region	0.996	*KAT2A*	c.1723-8T>C	L	—
chr19:42.99Mbp	LC	rs133665517	Missense	0.956	*STAT5B*	G40S	M	1.00 (T)
chr19:42.99Mbp	LC	rs381010891	Missense	0.919	*ZNF385C*	P210A	M	0.29 (T)
chr19:42.99Mbp	LC	rs132867911	Missense	0.919	*FKBP10*	T261I	M	0.75 (T)
chr19:42.99Mbp	LC	rs209920132	Splice Region	0.916	*ACLY*	c.1846-3T>C	L	—
chr19:42.99Mbp	LC	rs209373086	Splice Region	0.915	*JUP*	c.1055-4C>G	L	—
chr14:1.77Mbp	LY	rs109234250	Missense	1.000	*DGAT1*	A232K	M	1.00 (T)
chr14:1.77Mbp	LY	rs134364612	Missense	1.000	*SLC52A2*	K242E	M	1.00 (T)
chr14:1.77Mbp	LY	rs135258919	Missense	0.902	*HSF1*	V344A	M	1.00 (T)

Numbers of missense or splice region mutations in QTL for LC and LY which have LD>=0.90 with the top whole-genome sequence mutation. Only those QTL with at least one such variant are included. Mutation classifications are per SNPEff predictions. Abbreviations (L,M,H) for Variant Effect Predictor (VEP) are low, moderate and high impact respectively. For SIFT, T is tolerated, TLC is tolerated with low confidence, and D is deleterious

Seven loci had predicted protein effects that were highly associated with LC or LY, with five of the locations having only one plausible mutation for the effect. At the chr6:37.76Mbp and chr14:1.77Mbp loci, the top variants were missense mutations in the *ABCG2* [22] and *DGAT1* [12] genes. Both variants (*ABCG2* Y581S and *DGAT1* K232A) have been previously demonstrated to have major impacts on diverse milk composition phenotypes [23–25] and, therefore, can be assumed to be the causative variants for these QTL.

The remaining five QTL include loci that either appear to be novel to the current study, or represent regions that have been reported in other analyses of milk composition traits, but have had no causative gene and variant definitively assigned. Of these QTL regions, the chr19:42.99Mbp locus presented a number of candidates, with 11 sequence variants spread across eight genes. Notably, one of these was a predicted splice donor non-sense mutation in the *KCNH4* gene. The list of candidates for this region also included a predicted tolerated *STAT5B* G40S missense mutation, representing a gene that has been previously speculated to underlie milk composition and production traits in other populations [10, 26].

#### Expression analysis and identification of putative regulatory eQTL

**Fig. 4:**
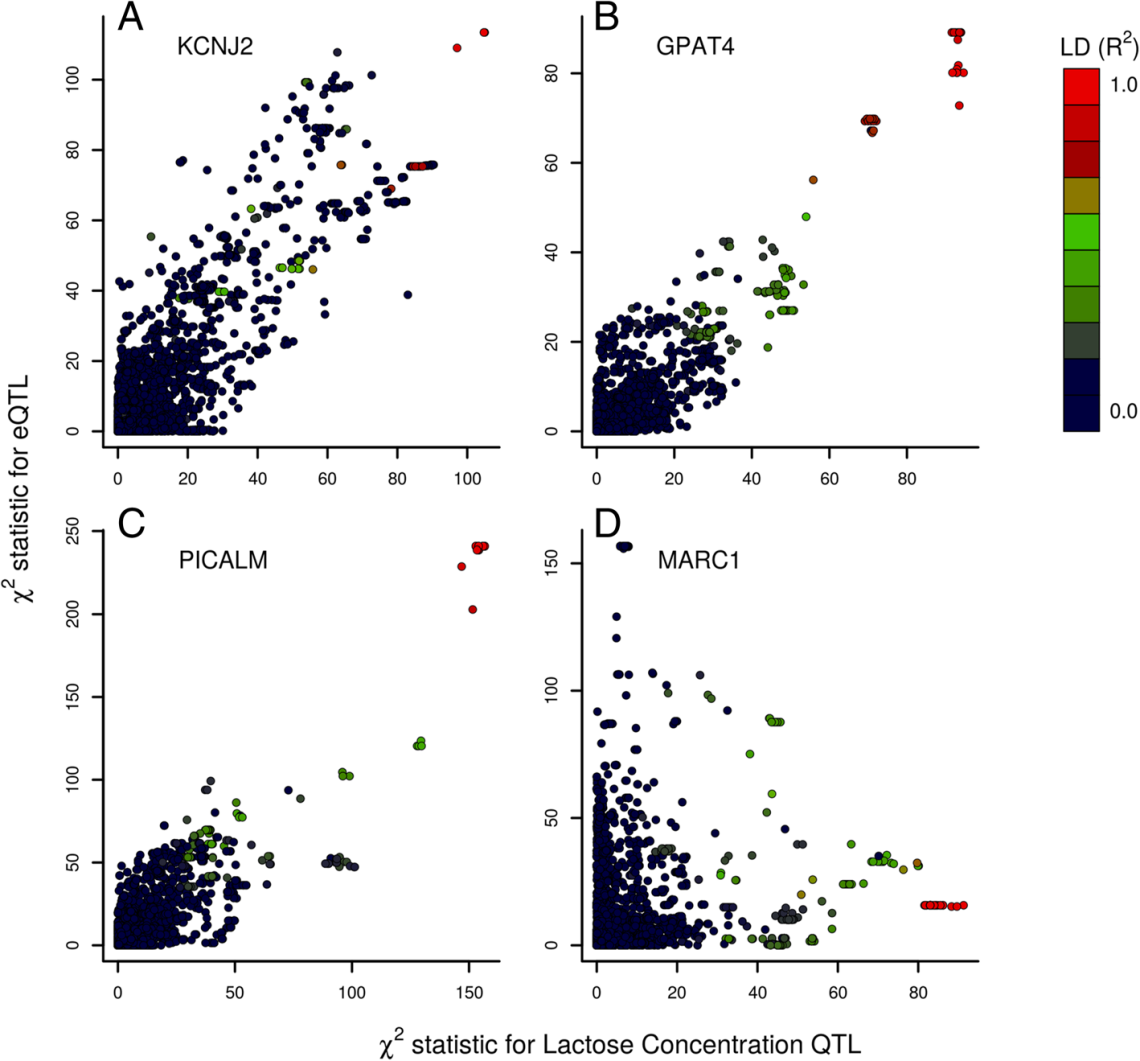
Correlations between lactose concentration QTL and eQTL. Panels **a**, **b**, and **c** show eQTL correlated with LC QTL where both QTL share the same top variant (*R* ^2^=1). Panel **d** shows a lactose QTL x eQTL pair for which no correlation is demonstrated. In each plot, the *χ* ^2^ statistic for each variant is plotted for the LC QTL on the X-axis and the eQTL on the Y-axis. Colours represent LD between each variant and the most strongly-associated variant for lactose concentration

Since most QTL are expected to be underpinned by regulatory mechanisms [27], and lack of functional annotation resources in cattle makes prediction of non-coding variants intractable, we next used a large mammary RNA sequence dataset to identify causative genes through co-segregating expression QTL (eQTL). In this context, eQTL analyses can provide functional evidence of the molecular basis of the QTL in question: cases where genetic signals not only collocate, but also share top associated variants, provide strong evidence of causality for the implicated gene [28–30]. Using imputed whole genome sequence data in a population of 357 lactating cows, *cis*-eQTL mapping was conducted using transformed mammary gene expression values representing all genes in each 1Mbp target interval (*n*=313 genes for all intervals; see “Methods” section). In an approach similar to that described previously [14, 15], analyses were also performed to calculate *χ* ^2^ correlation values for each 1Mbp interval of variants, with the expectation that an eQTL and lactose QTL underpinned by a common genetic element would share similar variant association statistics. Table 6 shows lactose QTL and eQTL pairs that: shared top associated variants in strong LD (*R* ^2^>0.9) that exceeded the eQTL genome-wide significance threshold of 2.53×10^−7^, and/or had an eQTL where at least one of the Pearson and Spearman (rank) correlations was greater than 0.7. Of the 26 loci, 14 have at least one gene meeting these criteria, with 11 having only one such gene. Notably, seven of these genes also shared top variants that were the same or were in strong LD (Table 6). Four eQTL × QTL association plots are illustrated in Fig. 4, showing examples of both highly correlated, and non-correlated QTL pairs. Of the highly correlated QTL, *LRRC8C*, *RAB3IP*, *NREP*, *IVNS1ABP*, *P2RX4*, *KCNJ2*, *ANKH*, *GPAT4*, *PICALM*, and *MGST1* are strong candidate causative genes for these effects, representing loci for which there is only one co-segregating eQTL, and where no plausible protein-coding variants have been identified.

**Table 6:** Correlations between lactose QTL and co-localised eQTL

Phenotype	Locus	Gene	eQTL *P*Val	Tag *P*Val	Pearson	Spearman	LD (*R* ^2^)
LC	chr1:154.14Mbp	*SH3BP5*	2.58×10^−32^	6.18×10^−32^	0.173	0.071	0.993
LC	chr3:15.52Mbp	*SLC50A1*	8.70×10^−16^	8.70×10^−16^	0.705	0.272	1.000
LC	chr3:53.84Mbp	*LRRC8C*	3.46×10^−39^	1.53×10^−36^	0.900	0.868	0.816
LC	chr5:44.16Mbp	*RAB3IP*	9.10×10^−23^	2.22×10^−22^	0.835	0.489	0.979
LC	chr10:2.14Mbp	*NREP*	6.12×10^−10^	1.55×10^−9^	0.822	0.774	0.676
LC	chr16:67.77Mbp	*IVNS1ABP*	4.54×10^−27^	6.54×10^−24^	0.812	0.339	0.887
LC	chr17:56.47Mbp	*P2RX4*	2.46×10^−39^	3.26×10^−13^	0.743	0.692	0.280
LC	chr19:42.99Mbp	*GHDC*	1.80×10^−22^	5.77×10^−16^	0.951	0.849	0.981
LC	chr19:42.99Mbp	*DHX58*	1.77×10^−8^	1.31×10^−5^	0.918	0.802	1.000
LC	chr19:42.99Mbp	*STAT5B*	5.72×10^−9^	1.51×10^−6^	0.915	0.773	0.524
LC	chr19:61.13Mbp	*KCNJ2*	1.72×10^−26^	1.72×10^−26^	0.870	0.645	1.000
LC	chr20:58.45Mbp	*ANKH*	2.40×10^−16^	2.40×10^−16^	0.783	0.404	1.000
LC	chr27:36.21Mbp	*GPAT4*	3.67×10^−21^	3.49×10^−19^	0.812	0.607	0.909
LC	chr29:9.61Mbp	*PICALM*	2.40×10^−54^	2.40×10^−54^	0.752	0.600	1.000
LC	chr29:9.61Mbp	*EED*	2.31×10^−9^	2.43×10^−9^	0.319	0.356	0.994
LY	chr5:93.94Mbp	*MGST1*	3.18×10^−43^	9.37×10^−43^	0.769	0.486	0.934
LY	chr14:1.77Mbp	*DGAT1*	8.87×10^−42^	3.68×10^−39^	0.946	0.829	0.923
LY	chr14:1.77Mbp	*CCDC166*	2.93×10^−8^	8.53×10^−3^	0.216	0.703	0.066

Co-localised genes for each lactose locus, where: the Pearson or Spearman correlation between the lactose QTL and eQTL is greater than 0.7, or, the LD between the top variants in the lactose QTL and eQTL is greater than 0.9. The *p*-value shown for the eQTL is that of the most significant SNP. The tag *p*-value is the significance of the lactose phenotype tag variant for the eQTL. Within each locus, genes are shown in descending order by Pearson correlation. Only eQTL which pass Bonferroni correction (threshold *p*=2.59×10^−8^) are shown

### Pathway analysis

We conducted functional clustering analysis using 44 genes using the DAVID database [31]. These genes represented candidates corresponding to predicted protein and regulatory effects, or proximity to the QTL peak (see “Methods” section). This analysis identified four significantly enriched annotation clusters (see Additional file 5: Table S1), using an alpha value of 0.05 (translating to an enrichment threshold calculated as − log10(0.05)≈1.3 [31]). The most highly enriched cluster (enrichment score 1.99) was for ion channels/transport, followed by the endoplasmic reticulum cellular compartment (1.95), potassium/voltage-gated ion channels (1.33), and lipid metabolic process (1.31) annotations.

## Discussion

### Lactose heritability and genetic architecture

Association analyses of milk composition and yield traits have now been published in multiple independent cattle populations [10, 26, 32], however, we are unaware of any such genome-wide studies focussed on the identification of QTL for lactose traits. Here we present such analyses, detailing 26 validated QTL spanning 18 bovine autosomes. Although lactose GWAS have been lacking, heritability estimates from the literature broadly fit with the estimates yielded for LC [4, 33] and LY [4, 34] in the current study. The comparatively lower heritability of LY compared to LC is similarly consistent with these estimates, and with the genetic architecture of the observed QTL. Like other milk composition phenotypes such as fat and protein [29], fewer significant QTL were observed for yield compared to concentration. Together, tag-variants of the validated LC QTL explained 4× the phenotypic variance of the five LY loci that also met our nominated *p*-value threshold, confirming that, like the highly correlated trait of milk yield, LY has a more distributed, infinitesimal genetic architecture. Importantly, 26 of 27 prioritised QTL validated between populations, with only the chr5:21.15Mbp locus failing to replicate. For the validated loci, inflation of effects was relatively modest, with the cumulative variance for each trait >80% of that estimated at the discovery stage (Table 2 and Additional file 3: Table S3).

### Genomic inflation

Large genomic inflation factors were observed in the discovery set for both the LC (2.28) and LY (2.25) phenotypes, potentially indicating the presence of false positive results. Since the most likely source of inflation was population stratification due to the mix of breeds in the data set, we also performed within-breed analyses. Heritabilities calculated for pure-bred subsets of animals were similar to those calculated for the total population, indicating that model adjustments to the phenotypes are likely adequately accounting for breeds and crosses in the population. Likewise, 24 of the 26 validated QTL identified in the mixed-breed population were replicated in at least one pure-bred subpopulation, with concordant signs of effect between breeds for all but one locus. These results suggest using the mixed-breed population has not substantially distorted the results, and although effect sizes might still be over-estimated, the QTL presented are unlikely to represent false positive signals.

### Pathway analysis highlights lactose regulation through osmotic balancing mechanisms and pleiotropic fat synthesis QTL

Functional clustering revealed four significantly enriched annotation clusters for the 26 validated LC and LY loci (Additional file 5: Table S1). These clusters encompass cellular and molecular functions that support the key role of lactose as the major osmolyte in milk. Notable classes of genes include transmembrane transport molecules that could be expected to impact osmotic balance through modulation of ion concentrations (*KCNH4*, *LRRC8C*, *KCNJ2*, *ANKH*). This is emphasised by the presence of a second significantly enriched cluster representing voltage-gated potassium ion channels. The enrichment of genes annotated to the endoplasmic reticulum cluster include candidates overlapping with transport functions (*LRRC8C*), and other endoplasmic reticulum membrane-bound enzymes including *MGST1*, *DGAT1*, and *GPAT4*. All three of these latter genes represent QTL with major impacts on other milk composition traits [12, 14, 15], with the enriched cluster encompassing lipid metabolic processes also assigned due to the *DGAT1* and *GPAT4* genes.

### Discovery of candidate causative genes and variants

To attempt to identify causative genes and variants underlying the biggest QTL, we applied two complementary approaches to fine map prioritised loci and look for co-locating, co-segregating eQTL as molecular signatures of these effects. These methods relied on two large-scale sequence-based datasets, comprising a whole-genome sequence reference population of 565 HF, Jerseys, and crossbreeds, and a mammary RNAseq dataset representing 357 lactating cows of similar composition. Sequence-based association analysis revealed several QTL where the top associated variants included polymorphisms anticipated to impact the coding sequence of protein-coding genes, and *cis*-regulated eQTL genes that likely underpin a proportion of the other lactose signals.

#### Protein-coding sequence variants

The two largest QTL with protein-coding effects were the chr6:37.76Mbp and chr14:1.77Mbp loci, each likely underpinned by the *ABCG2* Y581S and *DGAT1* K232A amino acid substitutions, respectively. These QTL were also the largest effects overall, with major impacts on LC and LY. These previously described variants represent two of the most highly cited and validated milk composition variants in the bovine literature [12, 13, 23–25], and serve as positive controls in our analyses.

Encouragingly, both missense variants appear in our list of SNPEff-filtered protein-coding candidate mutations, and both variants were the most highly associated markers in the sequence-resolution analysis of the respective traits. The ability to directly resolve the causative variants as the top-associated variants is also encouraging, though likely reflects the strength of association for these two major effect mutations. The ABCG2 Y581S mutation effect on LC is roughly equivalent to effects of the other 20 validated LC QTL combined, and it is also notable that, despite the magnitude of effect, no genome-wide significant effect was observed for LY (*p*=0.22). Given that LY and milk yield are highly correlated (0.99±0.01, [4]), and that the Y581S mutation was initially described for its impact on milk yield [13] and significantly impacts that trait in the current study, the lack of a corresponding effect on LY is surprising. This discrepancy could be explained by limited statistical power as a consequence of the very low MAF (0.009) of the Y581S variant in the discovery population, though an alternative explanation hints at a possible underlying mechanism for the variant. Although the Y581S mutation was first described >10yrs ago [13], no obvious mechanistic role for the impact on milk yield has yet emerged. A scenario where Y581S impacts milk yield and LC, but not LY, would suggest that *ABCG2* may be pumping some as yet unknown, osmotically active component into milk, with milk volume increasing as a consequence. This hypothesis fits with the status of *ABCG2* as an efflux transporter, and reconciles the profound impact of Y581S on LC (7.00% of phenotypic variance in the validation population, despite a low MAF).

Another curious observation is the substantial impact of the *DGAT1* K232A mutation on LY, in the absence of an effect on LC. This is despite a major effect on LC attributed to *GPAT4* (chr27:36.21Mbp). The *GPAT4* gene is a known causative gene for milk composition traits [14], and is functionally paralogous to *DGAT1*, with the two genes occupying adjacent nodes of the mammary triglyceride synthesis chain [35]. This observation is particularly puzzling given that the impact of *DGAT1* K232A on milk yield is much larger than that of the *GPAT4* locus [14], demonstrating the capacity for idiosyncratic effects of individual genes on milk composition, despite pleiotropy of effects more broadly [15, 29, 30]. Comparing between the milk composition and yield effects of other loci in the current study, similarly shows instances where an individual locus may associate with many traits (e.g. chr20:58.45Mbp for LC, fat, milk, and protein yield, and protein percentage), or may have pronounced effects on one characteristic, yet be completely unassociated with others (e.g. chr16:24.99Mbp for LC). The relativity of sign of effects between traits also appears to follow some rules (i.e. increasing milk volume and lactose yield, with decreasing component percentages), though exceptions to these observations abound.

Two other previously reported milk production and composition loci annotated with candidate protein-coding mutations are the chr6:89.04Mbp and chr19:42.99Mbp LC QTL. The chr6:89.04Mbp locus is adjacent two genes of note: GC and NPFFR2, the former favoured as a candidate gene for milk production and mastitis QTL in other populations [36, 37], with the latter highlighted by a highly significant missense mutation as a possible causative variant in the current study. These observations make both genes valid candidates for the LC QTL, though the proximity of the locus to the casein gene cluster at chr6:87Mbp should also be noted, presenting the possibility of long-distance LD effects. Since neither GC nor NPFFR2 were expressed in our lactating mammary dataset, further differentiation on the basis of expression information is not possible.

The chr19:42.99Mbp QTL has similarly been observed in other populations, and although no causative variants have been functionally demonstrated for the region, the signal has been generally assigned to the *STAT5A* and *STAT5B* genes [10, 26]. These genes make excellent candidates, given the key roles of the *STAT5* transcription factors in alveologenesis and milk protein gene expression [38]. A *STAT5B* G40S missense mutation is included on the list of candidates for the locus in the present study. However, two other protein coding variants in the *GHDC* and *KCNH4* genes also make this list, encoding a predicted deleterious amino acid substitution (*GHDC* P233A) and a splice donor non-sense mutation (*KCNH4* c.2663+2T>C). The *KCNH4* mutation in particular represents a plausible alternative to the *STAT5B* G40S variant as potentially underpinning this QTL, predicted to disrupt the function of a gene whose role as an ion transporter is part of an enriched class of genes in our dataset. It is also noteworthy however that co-segregating eQTL for the *GHDC*, *STAT5B*, and *DHX58* genes also coincide with this QTL. On that basis, disentangling the relative contribution of individual variants and mechanisms to this QTL is likely to be particularly challenging, potentially involving multiple molecular effects in strong LD. A further indication of the potential biological complexity at this locus can be gleaned from the within-breed analyses. Curiously, the chr19:42.99Mbp locus is the only QTL with opposite signs of effect between HF and Jerseys. Although this might otherwise make the locus a candidate false positive region, the effects are highly significant in both breeds, and given the variety of strong candidate genes, and observations of the QTL in independent populations [10, 26], it seems plausible the locus comprises multiple, functionally independent variants.

Three other loci annotated with protein function-based candidate causative variants appear to represent QTL novel to the current study. Two of these are LC QTL that we can find little reference to in GWAS of other milk traits. These two QTL at chr15:28.36Mbp and chr16:24.99Mbp are represented by highly significant missense mutations in genes for which either little is known (*RNF214*), or that have no straightforward biological role in the context of lactation (*MARC1*). With no alternative coding variants or co-segregating eQTL, these variants are therefore the most plausible candidates for these effects.

The chr3:15.52Mbp LC locus contains a strong candidate gene, the sugar transporter *SLC50A1* (aka *SWEET1*). This gene is annotated with a single candidate mutation with a possible impact on coding sequence, comprising a splice region variant c.282+7G>A. Manual visualisation of RNAseq alignments of animals of opposing QTL genotype suggest c.282+7G>A is unlikely to be the causative variant for this QTL, with no apparent impact on alternative splicing at the relevant *SLC50A1* intron 3 junction. However, observation of a lone, co-segregating *SLC50A1* eQTL at the locus strongly supports the causative status of this gene, particularly given its previously demonstrated roles in the murine mammary gland. Mammary expression of *SLC50A1* is strongly induced during lactation in the mouse, where critically, it is proposed to impact lactose production by altering glucose availability to the lactose synthase enzyme complex [39].

#### Expression-based effects

We previously generated a large, mammary RNAseq dataset to act as a resource for identification of causative genes for lactation traits. Our approach aims to identify co-locating eQTL and milk composition/production QTL with shared association signatures, providing functional and genetic evidence of causality for the implicated gene [14, 15, 29, 30]. Conversely, the presence of an uncorrelated eQTL may suggest that a gene is unlikely to be involved, at least through an expression-based mechanism. These methods rely on the assumption that the LD structures between the RNAseq population and GWAS population are similar at the loci of interest, and that the strength of association is sufficient to resolve both top and middle-order variants. These assumptions may not always hold, so we also consider genes candidates for collocating QTL if the lead associated eQTL variant is the same (or captures the same LD block) as the milk composition QTL. Of the 26 lactose QTL prioritised in the current study, 14 had strongly correlated, co-locating eQTL in lactating mammary tissue.

Observation of strong correlations for *MGST1* and *GPAT4* eQTL for LY and LC provide further positive controls for our analyses, where the likely causality of these genes in underlying QTL for other milk traits has been confirmed previously [14, 15]. As with assessment of the potential role of protein-coding variants for lactose QTL, the remaining 12 candidate causative eQTL represent both ‘simple case’ loci for which causality can be assumed (collocating with a single, highly correlated eQTL), to confounded regions presenting multiple overlapping expression and protein sequence-based candidate effects. Some of the more straightforward, and novel, examples are discussed below.

The *P2RX4*, *KCNJ2*, *LRRC8C*, and *ANKH* genes encode transmembrane proteins involved in ion transport, all four of which show strong, highly correlated eQTL. The *LRRC8C*, *ANKH* and *KCNJ2* genes in particular make likely candidates for these effects, since the top associated eQTL variants are also in strong LD with the lead LC variant for each region. The *KCNJ2* gene encodes an inwardly-rectifying potassium transporter that has previously been identified in the membranes of secretory cells in murine mammary glands [40]. Early work examining ionic concentrations in milk demonstrated the strong correlation between concentrations of lactose and various ions in milk (including K ^+^) [41], so an eQTL that drives changes in abundance of *KCNJ2* protein (and consequently K ^+^ ion transport) could be expected to result in some form of osmotic compensation impacting LC. The same is true of *LRRC8C*, an anion channel that is part of a family of genes with a key role in osmotic regulation [42]. Members of the *LRRC8* gene family are sensitive to changes in cell volume, specifically activated through cell swelling in response to osmotic challenge [42]. The *ANKH* gene is another small molecule transporter with potential impact on the osmotic status of mammary cells and vesicles, responsible for transport of the oxyanion inorganic pyrophosphate [43]. As a related or additional mechanism, the interaction of *ANKH* with LC might somehow derive from the regulation of calcium availability in the mammary gland, given the importance of pyrophosphate to calcium sequestration [43], and the fact that calcium is the most abundant mineral in milk. The *PICALM* gene is another excellent candidate causative gene that, although not involved in ion transport, is involved in vesicle transport and assembly as a clathrin recruitment protein [44]. Although it is unclear whether the class of vesicles targeted by *PICALM* are directly relevant to vesicular secretion of lactose [9], the gene displays a highly significant, highly correlated mammary eQTL, and could equally be expected to impact LC through secondary effects of vesicular transport of other milk components, or vesicle membrane recycling [45].

## Conclusions

We have conducted the first GWAS experiments focussed on milk lactose phenotypes, detailing discovery and validation of 26 QTL with large to moderate effects. Compared to previous GWAS of other lactation phenotypes, these 26 loci represent a mixture of novel and previously-described chromosomal regions. Using a combination of eQTL mapping and sequence-resolution association analysis, we propose candidate genes and mutations at the majority of these loci. Pathway analysis indicates that a number of the novel QTL are associated with ion transport and pathways impacting the osmolality of milk, emphasising the importance of lactose in this context. Together, these new QTL enhance our understanding of lactation physiology, and may have further implications for breeding dairy animals with customised milk characteristics.

## Methods

### Animal populations, lactose phenotypes and heritability estimation

Heritability estimation and GWAS was conducted in several different populations, consisting of varying proportions of HF, J, and their crosses. Purebreds were defined as animals with at least 15/16ths HF or J ancestry. The study populations comprised 12,000 mixed breed cows (3 704 HF, 1 648 J, and 6 648 crosses; referred to as the ‘discovery’ set), a distinct group of 18,000 animals of broadly matched breed composition (4 588 HF, 2 350 J, and 11,062 crosses; the ‘validation’ set), and two purebred cohorts of 14,857 Holstein-Friesians and 8 995 Jersey cows. All purebred cows from the discovery and validation sets were included in the enlarged, purebred-only cohorts. All 30,000 cows were located in commercial New Zealand dairy herds.

LC and LY phenotypes were derived from measurements taken as part of standard herd-testing procedures. Milk samples were processed by LIC Testlink (Newstead, Hamilton, New Zealand) using Fourier transform infrared spectroscopy with the Milkoscan FT6000 instrument (FOSS, Hillerød, Denmark) against a lactose monohydrate standard. Individual phenotypic measurements for each animal were estimated from repeated measures models in ASReml-R, where concentrations and yield values were fitted against birth year, stage of lactation, and age of calving as fixed effects, animal as a random effect, and contemporary group as an absorbed/sparse effect. Measurements were restricted to herd tests during the cow’s first lactation, with somatic cell count <250k and at peak lactation (October to January inclusive). Subject to these restrictions, 59.5% of the discovery animals had data from at least two herd tests available, and 57.6% of the validation animals.

Narrow-sense heritabilities (*h* ^2^) were calculated for LC and LY using the GCTA (version 1.25.3) software package [46] for 30,000 animals, including those in both the test and validation sets. Heritabilities were determined using the genomic relationship matrix (GRM) calculated for these animals by GCTA from a combination of physically genotyped and imputed Illumina BovineHD genotypes (see Genotypes and imputation for GWAS section below), with MAF>0.05. These heritability estimates were used as parameters in the linear models to map associations between the phenotypes and the SNP genotypes. Genotypic principal component analysis was also conducted in the combined discovery and validation animal population, using the same BovineHD genotype set used for heritability calculations.

### Genotypes and imputation for GWAS

Lactose GWAS were conducted using SNPs imputed from a reference population of animals for which both SNP chip and RNAseq-derived genotypes were available. Animals were imputed using Beagle 4 software [46], using a stepwise procedure. In the first step, Illumina BovineHD SNP-chip content was imputed into the subset of 27 cows that had been genotyped on a lower density panel (Illumina Bovine SNP50 BeadChip platform) than the other RNA-sequenced animals. This process yielded 400 animals with 675,321 BovineHD SNPs.

To increase the density of variants available for genetic mapping, RNAseq alignments (see “RNA sequencing and gene expression phenotypesRNA sequencing andgene expression phenotypes” section below) were used as inputs for variant calling. These variants were chosen since they represented mammary-expressed genes, and hence had higher *a priori* likelihoods of affecting lactation phenotypes. Variants were called using Samtools (version 1.0)[47] and GATK HaplotypeCaller (version 3.3) [48]. Variants not called by both callers were excluded, with the remainder phased using Beagle 4 [49]. Variants that generated poor phasing metrics, as defined by an allelic *R* ^2^<0.95, were also excluded, along with markers with read depth <8, call rate <0.9 or minor allele frequency <2.5%. These criteria resulted in an RNAseq variant set of 410 animals and 477,531 variants. The imputation reference population was generated by merging genotypes for animals in both the HD and RNAseq variant sets. Phasing the merged variant set and excluding variants with allelic *R* ^2^<0.95 yielded the final imputation reference population of 394 animals and 1,093,581 variants.

This variant set was then used to impute all variants into the GWAS population of dairy cows (*n*=12,000: the discovery set) that had been physically genotyped on a mixture of Illumina BovineSNP50 (*N*=10,217), BovineHD (*N*=189), and GeneSeek Genomic Profiler BeadChip (*N*=1,945; GeneSeek/Illumina) SNP platforms. Three hundred and forty six animals had been genotyped on at least two platforms. After imputation, additional variants that did not impute well in this population, primarily multi-allelic indel mutations, were also removed, yielding 1,090,999 variants for GWAS. Because of difficulties in imputing sex chromosomes, only autosomal variants were targeted.

### Generalised least squares models

Generalised least squares models were run for both LC and LY phenotypes, as well as for the gene expression phenotypes described below. Variant effects were estimated using the single-SNP linear model in Eq. 1, where *X* is a matrix of SNP genotypes (coded 0,1,2 and centred to a mean of zero) and *y* is the vector of phenotypes. This model accounted for covariances between animals, caused by family relatedness or population stratification (different breeds), by using the covariance matrix in Eq. 2, which partitioned the phenotypic variance ($\sigma ^{2}_{P}$) into an additive genetic component, with covariance modelled by the numerator relationship (*A*) matrix, and an environmental component, with covariance modelled by an identity matrix (*I*). The proportions of variance allocated to each component were determined by the heritability. In this study, the *A* matrix was calculated from pedigree records.

1 $$\begin{array}{*{20}l} \hat\beta = \left(X{\prime} W^{-1} X \right)^{-1} \cdot X{\prime} W^{-1} y \end{array} $$

2 $$\begin{array}{*{20}l} W = \sigma^{2}_{p} \cdot \left(h^{2} A + (1-h^{2})I \right) \end{array} $$

The association strength for each SNP was calculated as per Eq. 3. The resulting statistic was *χ* ^2^-distributed with one degree of freedom, under the null hypothesis of no association between the SNP and phenotype. The calculation for the standard error of the estimated SNP effect is given in Eq. 4.

3 $$ \hat\chi^{2} = \left(\frac{\hat\beta_{2} }{ s.e(\hat\beta_{2})} \right)^{2}  $$

4 $$ s.e(\hat\beta) = \sqrt{\text{diag}(X' W^{-1} X)^{-1} }  $$

Because the expected distribution of the association statistic is known, the inflation factor of the statistics, denoted by *λ*, could be estimated by comparing the observed and theoretical medians of the *χ* ^2^ statistics. In particular, the theoretical median of the $\chi ^{2}_{df=1}$ distribution is 0.45494, and dividing the observed median by this value yielded the inflation factor.

Significance levels were calculated using Bonferroni corrections to adjust for multiple testing. Thresholds were calculated for each discrete experiment, where the nominal *p*-value for each of the lactose phenotypes was *P*=4.58×10^−8^ (*n*=1,090,999 variants). Bonferroni thresholds were set for the whole-genome sequence window analysis by considering all variants cumulatively, yielding a value of *P*= 2.53×10^−7^ (*n*=197,338 total variants). To calculate inflation-adjusted values, the value in the $\chi ^{2}_{df=1}$ distribution with an upper tail equal to this *p*-value was obtained and multiplied by *λ*. The *λ* inflation factors for the LC and LY phenotypes were 2.28 and 2.25 respectively, yielding nominal inflation adjusted thresholds of 1.61×10^−16^ and 2.52×10^−16^. Due to the exclusion of obvious true positive signals, inflation values are reported and visualised in Figs. 1 and 2 for comparison purposes, though not used subsequently.

### Whole genome sequencing, imputation, and association analysis

Whole genome sequencing was performed as described previously [11, 15]. Briefly, 565 animals comprising Holstein-Friesians, Jerseys, and crossbreeds thereof were sequenced using 100bp paired-end reads on the Illumina HiSeq 2000 instrument. Mapping was conducted using BWA MEM 0.7.8 [50], yielding mean and median mapped read depths of 15× and 8× respectively for the 565 samples. Variant calling was conducted using GATK HaplotypeCaller (version 3.2) [48] with base quality score recalibration. This variant set was phased using Beagle 4 [49], and variants with allelic *R* ^2^<0.95 were excluded.

To conduct sequence-based association analysis, 1Mbp windows centred on the top LC and LY QTL markers were imputed to whole-genome sequence resolution using Beagle 4 [49] with the reference population of 565 animals described above. Across all 27 chromosomal regions, this process resulted in a total of 197,338 variants (average 7 309; min 3 862; max 11,307 per interval). Although we have no truth set with which to directly determine the imputation accuracy for these animals, previous work we have performed [15] indicates accuracies of around 98–99% when imputing BovineHD genotypes to WGS. Association analysis was conducted as for analysis of other populations, using the same generalised least square models described.

Following discovery of the 27 LY and LC large to moderate effect QTL, a validation study using tag-variants of these regions was conducted in a separate population of 18,000 animals. These 27 sequence variants were imputed as described above. Association analysis was conducted as for analysis of other populations, using the same generalised least square models described above.

To determine whether or not the 27 observed QTL were segregating in both the HF and Jersey breeds, we calculated the within-breed MAF for each tag variant for both the discovery and validation animal sets (Table 3). MAFs were calculated using PLINK software [51] version 1.90b3i. To verify that effect directions were concordant across breeds and look for potential indicators of population stratification, genotypes of tag variants were extracted for larger pure-bred populations (*n*=14,875 for HF; 8 995 for Jersey), where these populations included all pure-bred animals from the discovery and validation populations. Allele effects were calculated using the generalised least-squares model as described above.

### RNA sequencing and gene expression phenotypes

Mammary biopsy, RNA sequencing, and RNAseq bioinformatics were performed as reported previously [15]. Briefly, high-depth mammary RNAseq was conducted on tissue from 411 cows, sampled as three groups at different points in time. Following library preparation, samples were sequenced using the Illumina HiSeq 2000 instrument to produce 100 bp paired-end reads, multiplexed at two samples per lane [15].

RNASeq reads for all 411 cows were mapped to the UMD 3.1 bovine reference genome using Tophat2 (version 2.0.12) [52], mapping an average of 88.9 million read-pairs per sample. Duplicate reads were marked using the MarkDuplicates command in the Picard software package (version 1.89; Broad Institute) and were excluded from SNP calling. Gene expression, in fragments per kilobase of transcript per million mapped reads (FPKM) and transcripts per million (TPM) [53], was quantified using Stringtie software (version 1.2.4) [54], and Ensembl genebuild release 81. Animals were filtered to remove those with outlier gene expression values using principal component analysis (PCA). Those with values more than three standard deviations from the mean in any of the first six components were excluded, based on the guidelines of Ellis et al. [55]. The resultant data set contained 375 animals. Expression data were also processed using the bioconductor package DESeq [56] to transform read counts using the “variance stabilising transformation” (VST) function, resulting in gene expression data suitable for linear model analysis. Only reads that mapped to exons (Ensembl release 81) were counted.

### Functional prediction of protein-coding variants and identification of co-segregating eQTL

For each 1Mbp window of whole genome sequence-resolution genotypes, SNPEff [19] (version 4.3) was used to predict functional consequences of candidate variants in conjunction with the Ensembl UMD3.1.82 gene annotations. Variants predicted to impact protein-coding sequences were also annotated using the Variant Effect Predictor [20] (Ensembl release 87). For eQTL analyses, transformed gene expression phenotypes for all expressed genes overlapping each of the 1Mbp windows were used to identify eQTL, where a nominal expression threshold of >8 exonic reads per animal was used. Animals whose genotypes were not concordant with genotypes from their sire (*n*=5) or dam (*n*=11), or had excessively low call rates (*n*=2) were not imputed to sequence resolution, yielding a final eQTL dataset of 357 animals. For these 357 animals, mapping was performed using imputed sequence variants and VST-transformed read counts, using the same generalised least squares models described above. Additional file 6: Figure S3 shows the Manhattan plots for each eQTL tested.

Following eQTL detection, correlation analysis of eQTL and lactose QTL association statistics was performed to highlight shared regulatory architecture between QTL. This method assumes that pairs of QTL regulated by a common genetic element will have similar association statistics, sharing the same highly associated (and un-associated) variants for a given interval. Correlations between the eQTL and the LC or LY QTL were calculated in the discovery animal set using Pearson (*r*) and Spearman (*ρ*) statistics between the *χ* ^2^ for each SNP in the window. Linkage disequilibrium statistics (*r* ^2^) between the genotypes of the top SNP for each lactose QTL and eQTL pair were also calculated.

### Pathway analysis

Candidate genes were nominated based on a triage of one or more of the following features: a protein-coding mutation with *r* ^2^>0.9 with the top LC or LY QTL variant; an eQTL with *r*>0.7 or *ρ*>0.7 with the LC or LY QTL; the top variants in the eQTL and the LC or LY QTL with *r* ^2^>0.9. This generated a list of 30 candidate genes covering 18 of the 26 validated QTL. For the eight remaining loci, for which genes could be less definitively implicated, candidates were added based on their proximity to the lead tag-QTL SNP, and/or the presence of an eQTL (whether this was co-segregating with the lactose QTL or otherwise) and/or strong *a priori* candidacy. The latter classification was invoked for LALBA (chr5:31.56Mbp) and GHR (chr20:31.69Mbp) only. The final candidate list consisted of 44 genes (Additional file 5: Table S1).

Ensembl IDs for candidate genes were input into the Database for Annotation, Visualization and Integrated Discovery (DAVID; [31]) online pathway analysis software (version 6.8; https://david.ncifcrf.gov/home.jsp). The Functional Annotation Clustering tool on this site was run using *Bos taurus* as the background species and using all DAVID default annotations as input, with the exception of the gene ontology annotations, where GOTERM_MF_ALL, GOTERM_CC_ALL and GOTERM_BP_ALL were used. Classification stringency for clustering was set to Highest.

## Additional files

Additional file 1 **Figure S1**. Stratification in the 30,000 discovery and validation animals, illustrated using PCA on the GRM matrix. Animals are coloured by the percentages of ancestry recorded in the LIC animal recording database. Breeds are Jersey and Holstein-Friesian. PCA was performed using GCTA [46]. (PDF 1770 kb)

Additional file 2 **Figure S2**. WGS resolution for 1Mbp windows centred on QTL peaks for lactose phenotypes. (PDF 1010 kb)

Additional file 3 **Table S3**. Tag-variant results for LC and LY QTL peaks in the validation data set. (XLSX 7 kb)

Additional file 4 **Table S4**. Associations between tag variants and milk phenotypes. Tag variants represent the 26 validated QTL detected for the LC and LY phenotypes. Phenotypes are milk yield (litres/day), milk fat and milk protein yield (kg/day) and milk fat and protein concentrations (percentage). (XLSX 42 kb)

Additional file 5 **Table S1**. All genes used in the functional annotation clusters pathway analysis tool (DAVID), along with the output clusters and associated enrichment scores and enriched annotation classes. (XLSX 41 kb)

Additional file 6 **Figure S3**. WGS resolution for eQTL of all gene located within 1Mbp windows centred on QTL peaks for lactose phenotypes. (PDF 8428 kb)

## Abbreviations

eQTL: Expression quantitative trait locus
HF: Holstein-Friesian
LC: Milk lactose concentration
LD: Linkage disequilibrium
LY: Lactose yield
MAF: Minor allele frequency
QTL: Quantitative trait locus
RNAseq: RNA sequence data
SNP: Single nucleotide polymorphism
