# Supplementary figures and images for: DNA and RNA-sequence based GWAS highlights membrane-transport genes as key modulators of milk lactose content
Source: BMC Genomics. 2017 Dec 15;18:968. doi: 10.1186/s12864-017-4320-3 (PMC5731188; doi:10.1186/s12864-017-4320-3)

PCA: Holstein–Friesians and Jerseys

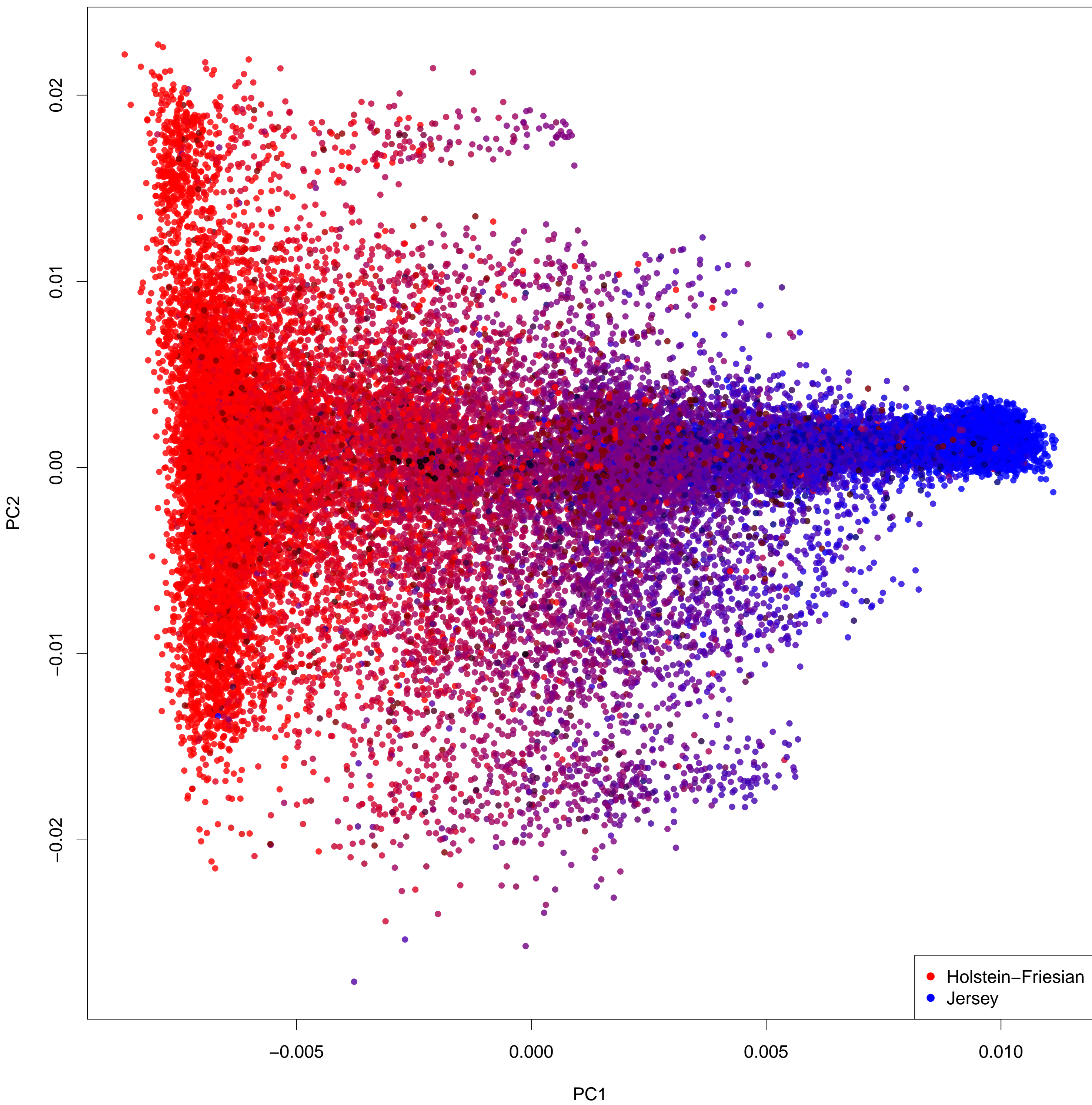

Supplement: Supplementary file 1 — Figure S1. Stratification in the 30,000 discovery and validation animals, illustrated using PCA on the GRM matrix. Animals are coloured by the percentages of ancestry recorded in the LIC animal recording database. Breeds are Jersey and Holstein-Friesian. PCA was performed using GCTA [46]. (PDF 1770 kb) [file 12864_2017_4320_MOESM1_ESM.pdf]
