# Supplementary material for: DNA and RNA-sequence based GWAS highlights membrane-transport genes as key modulators of milk lactose content
Source: BMC Genomics. 2017 Dec 15;18:968. doi: 10.1186/s12864-017-4320-3 (PMC5731188; doi:10.1186/s12864-017-4320-3)
Supplement: Supplementary file 2 — Figure S2. WGS resolution for 1Mbp windows centred on QTL peaks for lactose phenotypes. (PDF 1010 kb) [file 12864_2017_4320_MOESM2_ESM.pdf]

GWAS for Lactose Concentration at Chr1:154.14

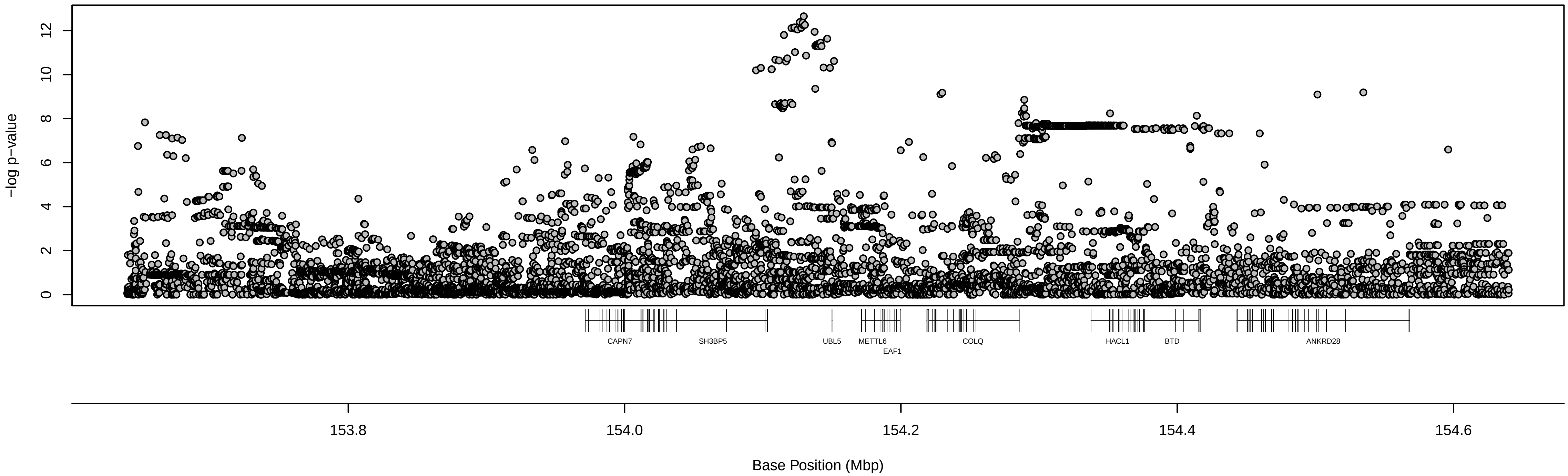

GWAS for Lactose Concentration at Chr2:127.64

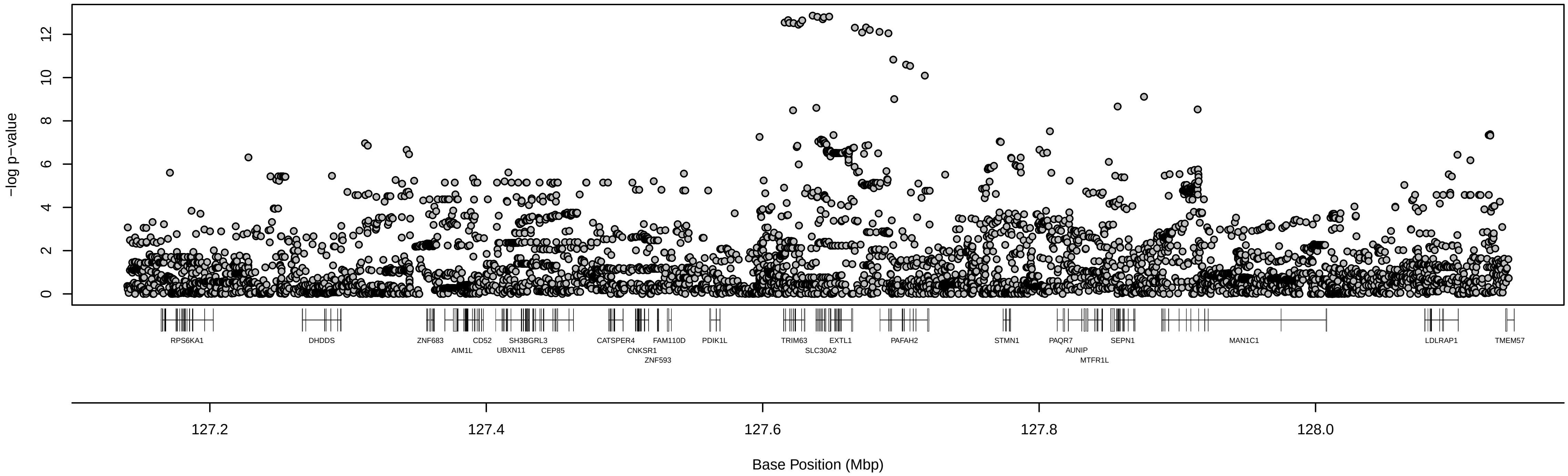

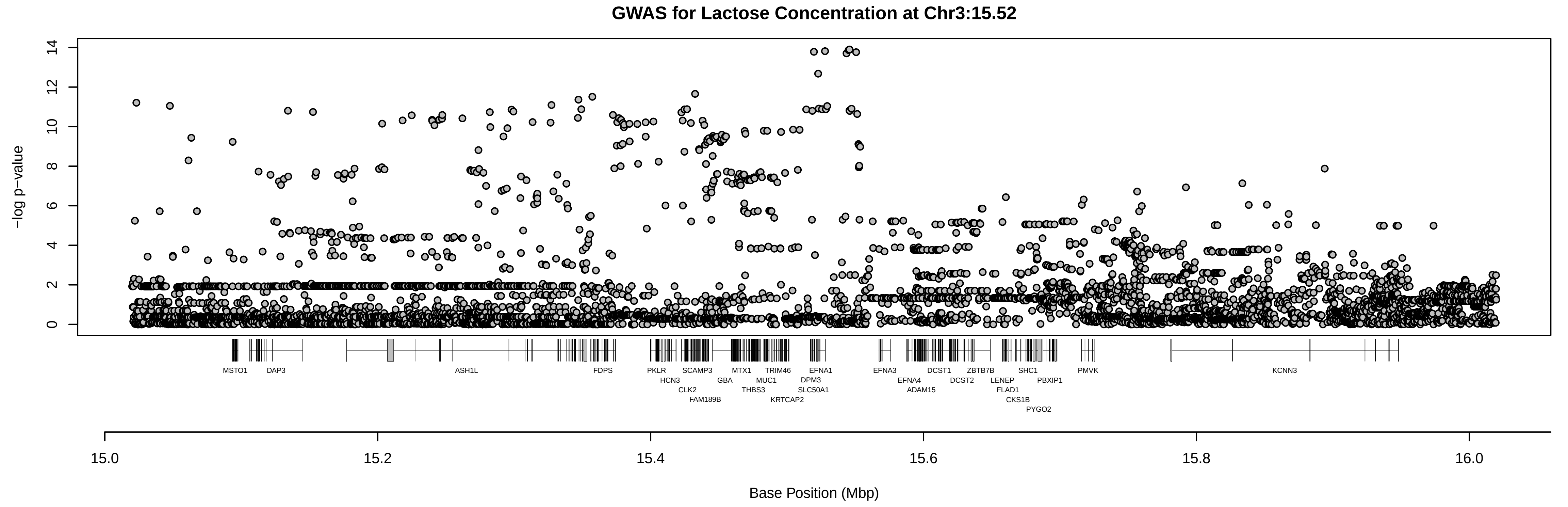
GWAS for Lactose Concentration at Chr3:15.52
−log p−value
0
2
4
6
8
10
12
14
MSTO1
DAP3
ASH1L
FDPS
PKLR
HCN3
CLK2
SCAMP3
FAM189B
GBA
MTX1
THBS3
MUC1
TRIM46
KRTCAP2
EFNA1
DPM3
SLC50A1
EFNA3
EFNA4
ADAM15
DCST1
DCST2
ZBTB7B
LENEP
FLAD1
CKS1B
SHC1
PYGO2
PBXIP1
PMVK
KCNN3
15.0
15.2
15.4
15.6
15.8
16.0
Base Position (Mbp)

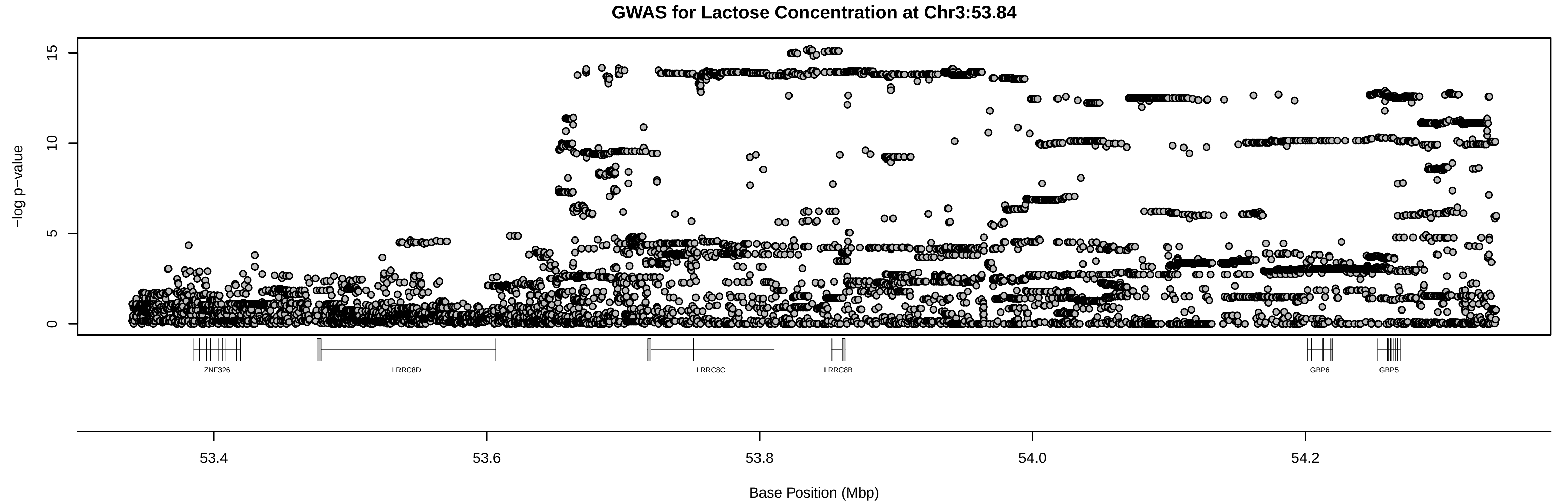
GWAS for Lactose Concentration at Chr3:53.84
−log p−value
0
5
10
15
ZNF326
LRRC8D
LRRC8C
LRRC8B
GBP6
GBP5
53.4
53.6
53.8
54.0
54.2
Base Position (Mbp)

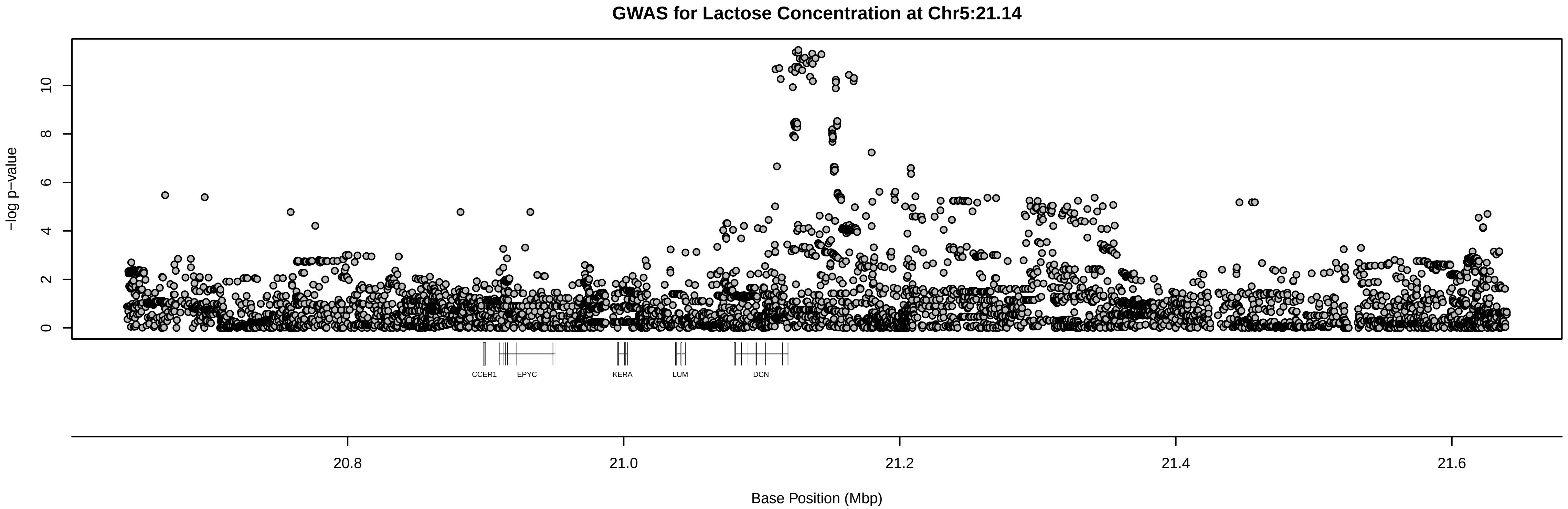
GWAS for Lactose Concentration at Chr5:21.14
−log p−value
0
2
4
6
8
10
CCER1
EPYC
KERA
LUM
DCN
20.8
21.0
21.2
21.4
21.6
Base Position (Mbp)

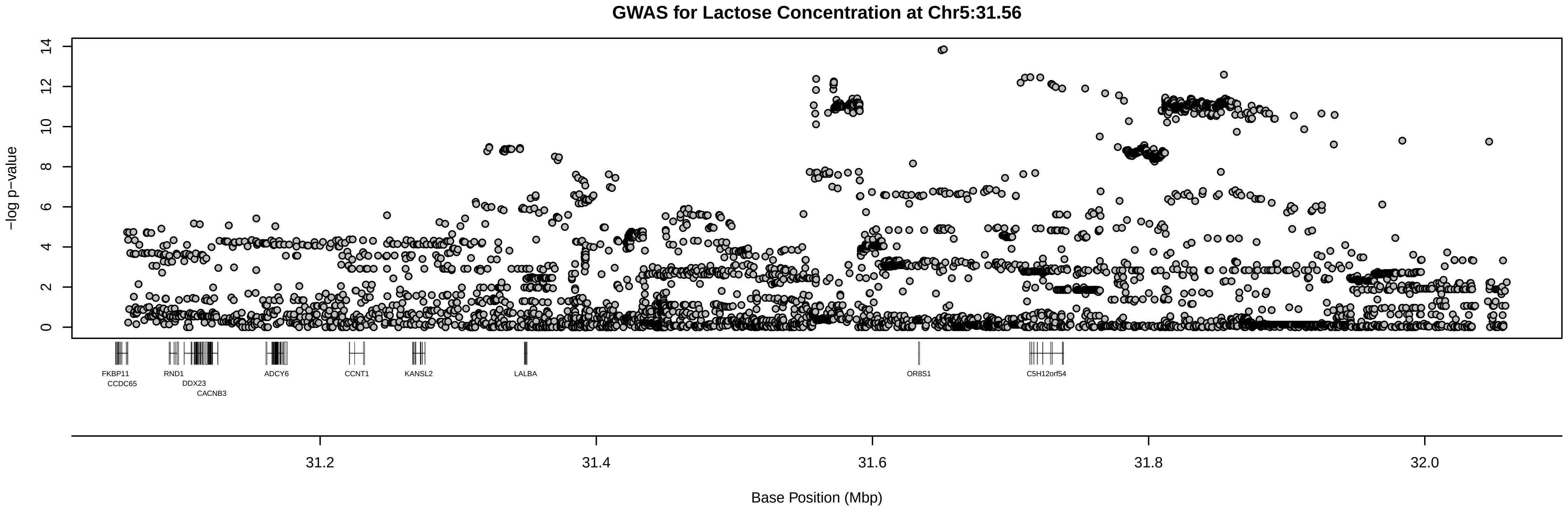
GWAS for Lactose Concentration at Chr5:31.56
−log p−value
0
2
4
6
8
10
12
14
FKBP11
CCDC65
RND1
DDX23
CACNB3
ADCY6
CCNT1
KANSL2
LALBA
OR8S1
C5H12orf54
31.2
31.4
31.6
31.8
32.0
Base Position (Mbp)

GWAS for Lactose Concentration at Chr5:44.16

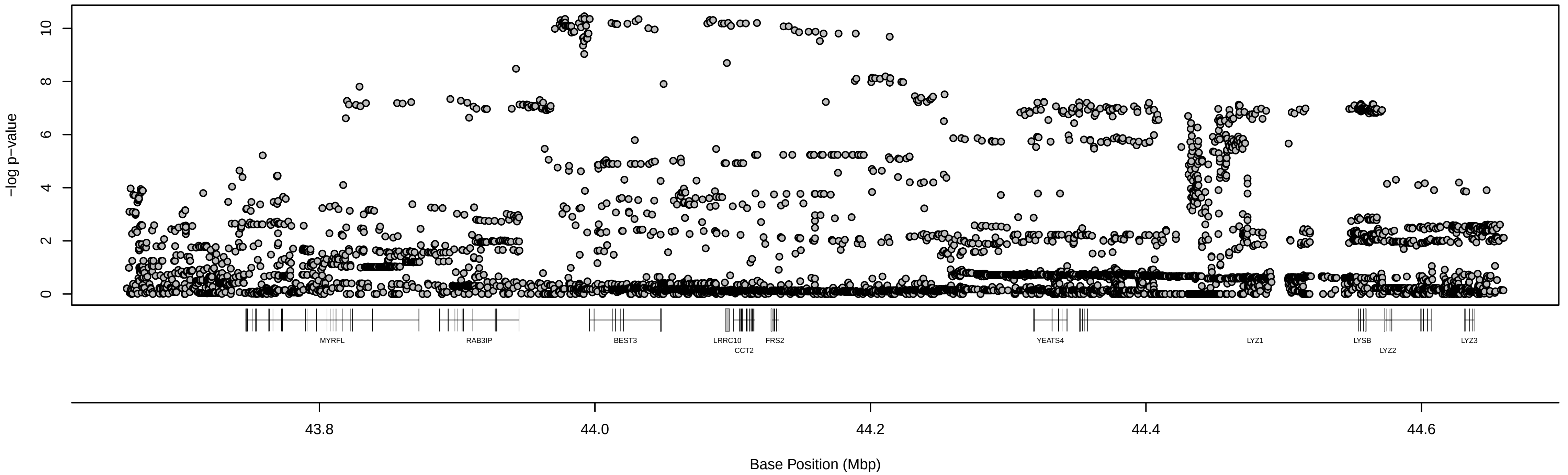

GWAS for Lactose Concentration at Chr6:37.76

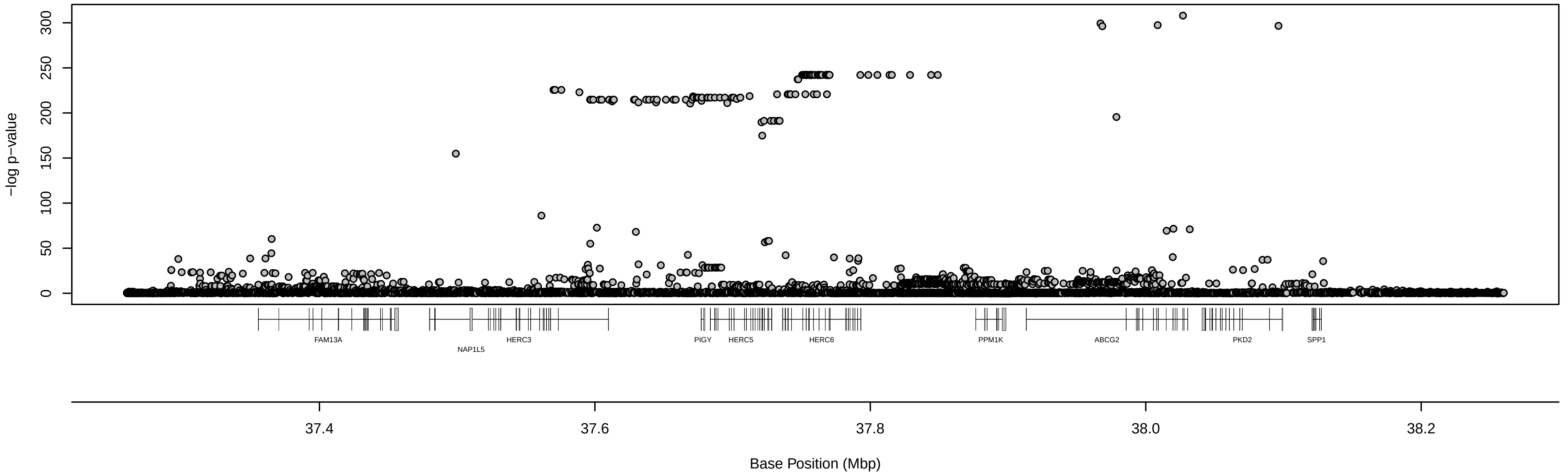

GWAS for Lactose Concentration at Chr6:89.04

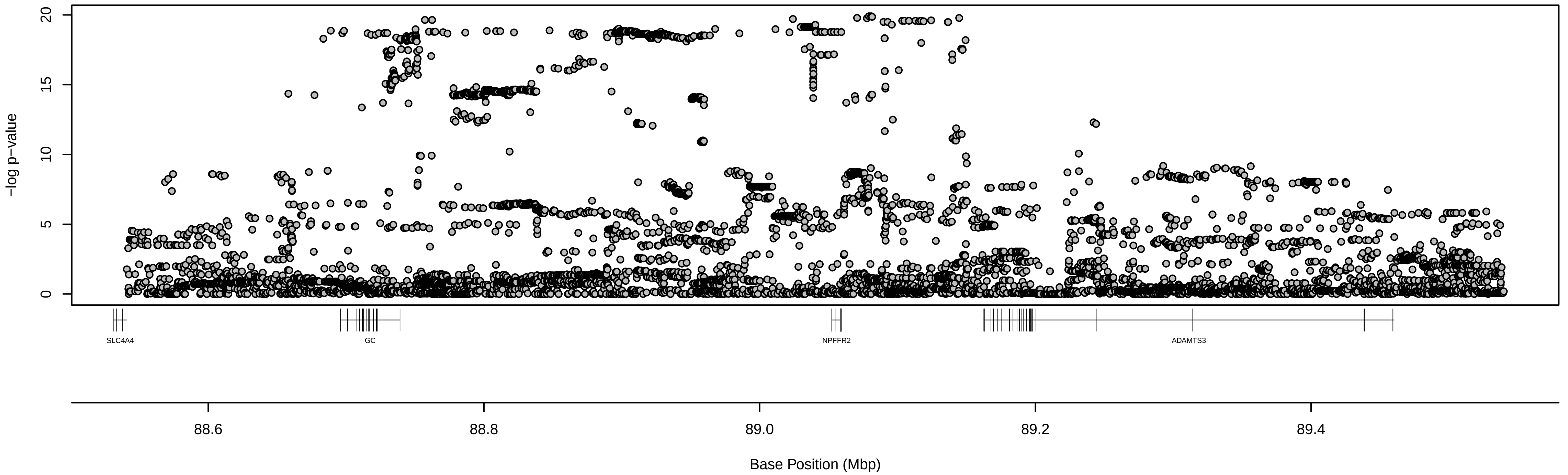

GWAS for Lactose Concentration at Chr7:8.77

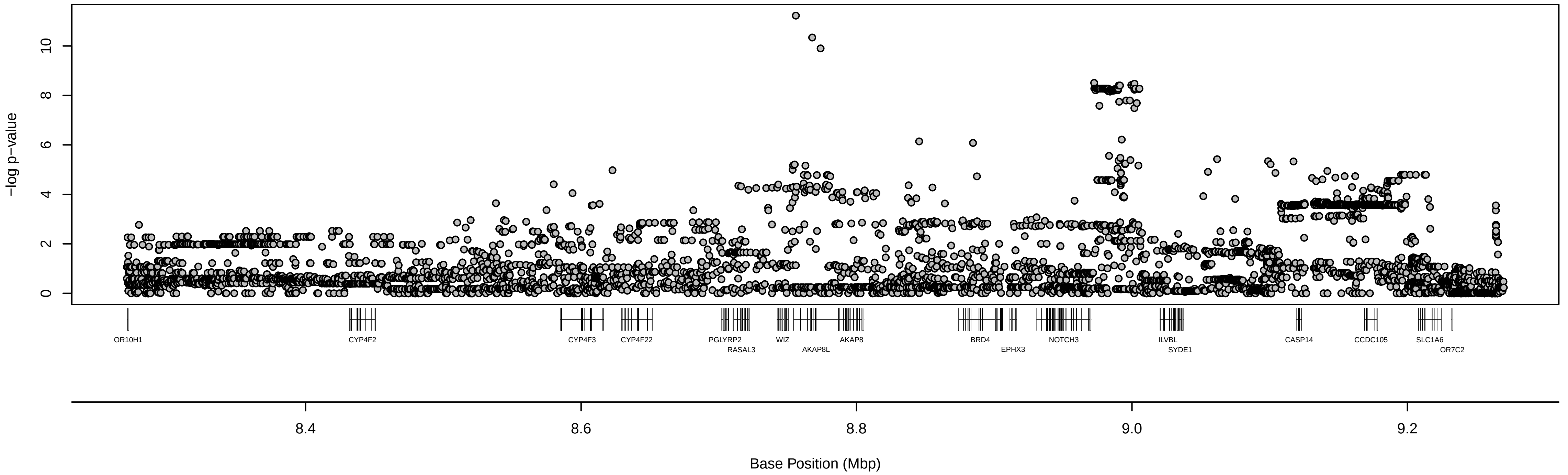

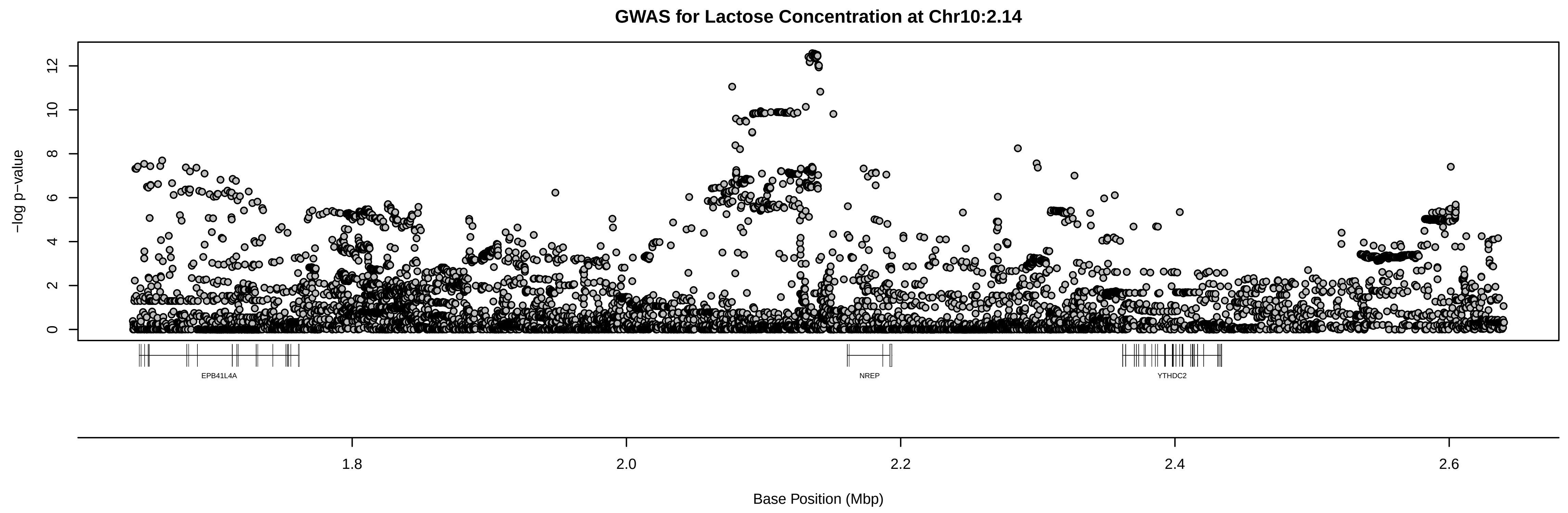
GWAS for Lactose Concentration at Chr10:2.14
−log p−value
0
2
4
6
8
10
12
EPB41L4A
NREP
YTHDC2
1.8
2.0
2.2
2.4
2.6
Base Position (Mbp)

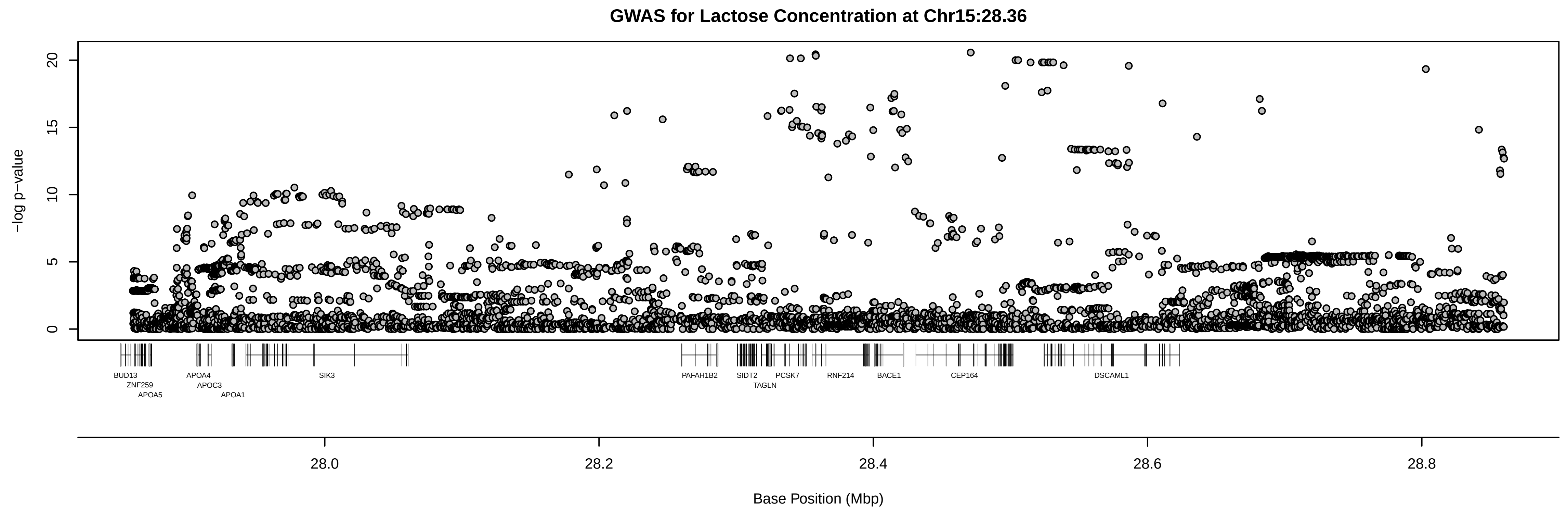
GWAS for Lactose Concentration at Chr15:28.36
−log p−value
0
5
10
15
20
BUD13
ZNF259
APOA5
APOA4
APOC3
APOA1
SIK3
PAFAH1B2
SIDT2
TAGLN
PCSK7
RNF214
BACE1
CEP164
DSCAML1
28.0
28.2
28.4
28.6
28.8
Base Position (Mbp)

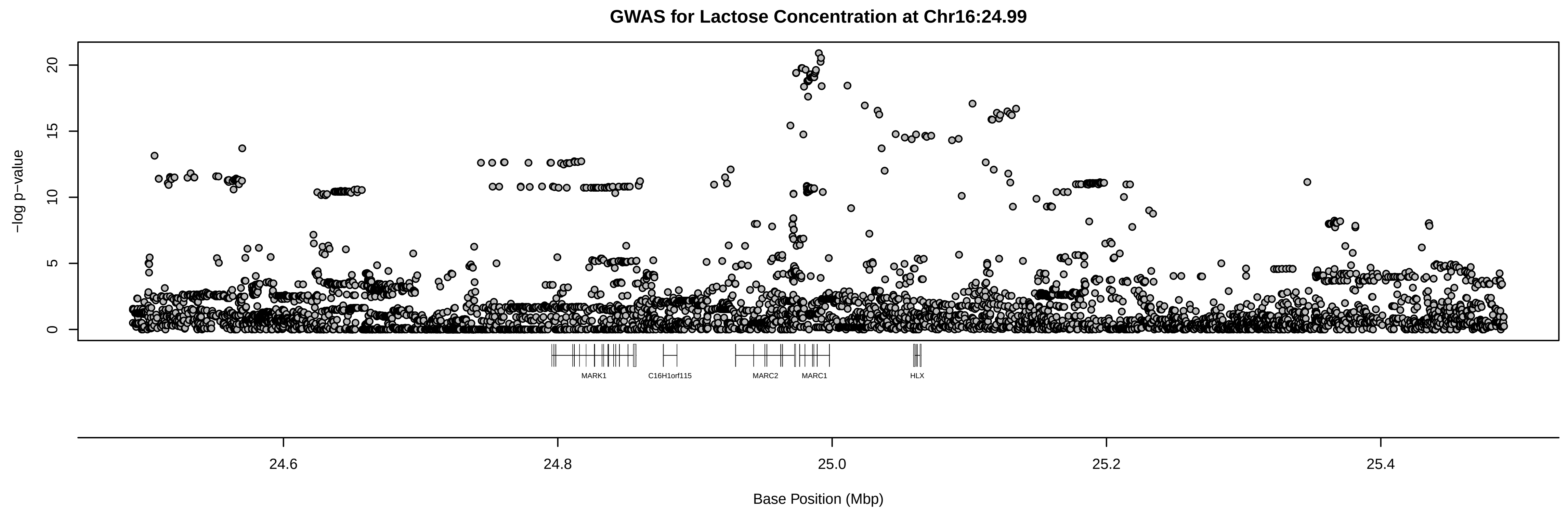

GWAS for Lactose Concentration at Chr16:24.99
−log p−value
MARK1
C16H1orf115
MARC2
MARC1
HLX
24.6
24.8
25.0
25.2
25.4
Base Position (Mbp)

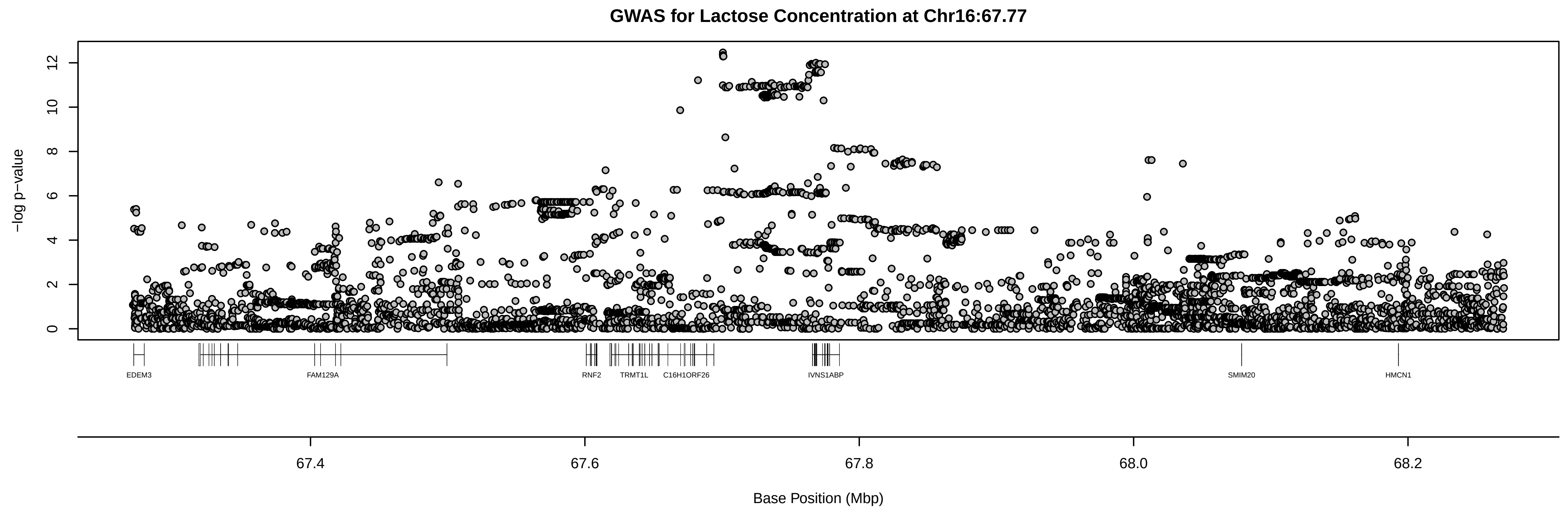

GWAS for Lactose Concentration at Chr16:67.77
−log p−value
EDEM3
FAM129A
RNF2
TRMT1L
C16H1ORF26
IVNS1ABP
SMIM20
HMCN1
67.4
67.6
67.8
68.0
68.2
Base Position (Mbp)

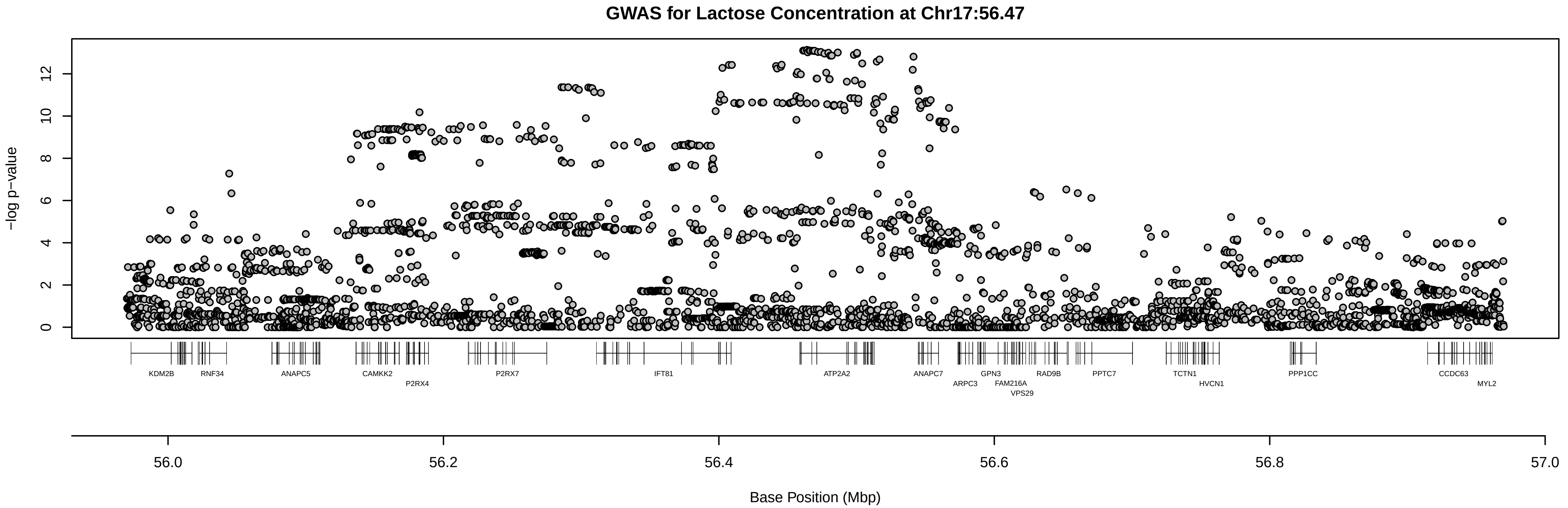
GWAS for Lactose Concentration at Chr17:56.47
−log p−value
0
2
4
6
8
10
12
KDM2B
RNF34
ANAPC5
CAMKK2
P2RX4
P2RX7
IFT81
ATP2A2
ANAPC7
ARPC3
GPN3
FAM216A
VPS29
RAD9B
PPTC7
TCTN1
HVCN1
PPP1CC
CCDC63
MYL2
56.0
56.2
56.4
56.6
56.8
57.0
Base Position (Mbp)

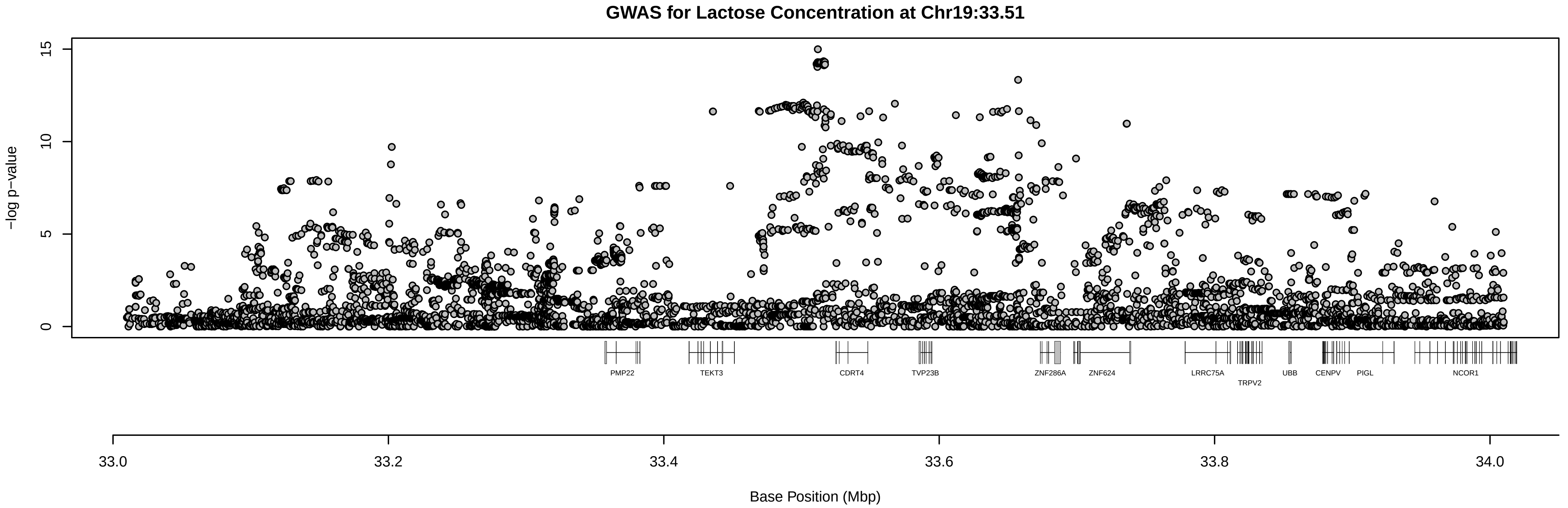
GWAS for Lactose Concentration at Chr19:33.51
−log p−value
0
5
10
15
PMP22
TEKT3
CDRT4
TVP23B
ZNF286A
ZNF624
LRRC75A
TRPV2
UBB
CENPV
PIGL
NCOR1
33.0
33.2
33.4
33.6
33.8
34.0
Base Position (Mbp)

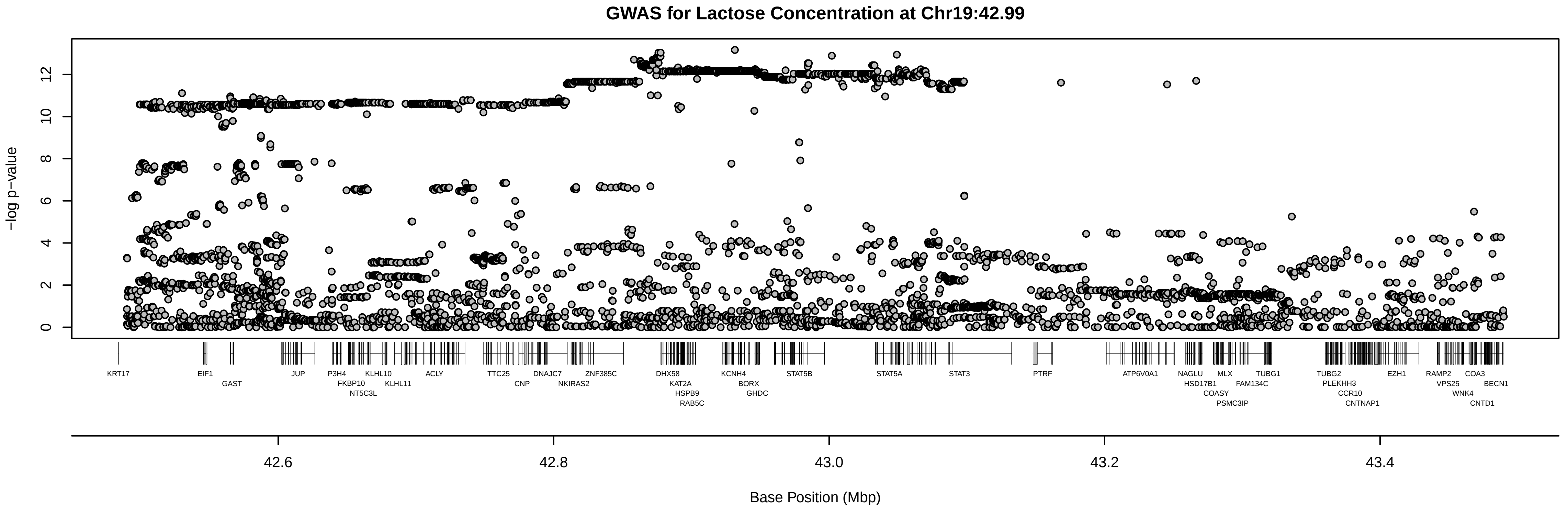
GWAS for Lactose Concentration at Chr19:42.99
−log p−value
0
2
4
6
8
10
12
KRT17
EIF1
GAST
JUP
P3H4
FKBP10
NT5C3L
KLHL10
KLHL11
ACLY
TTC25
CNP
DNAJC7
NKIRAS2
ZNF385C
DHX58
KAT2A
HSPB9
RAB5C
KCNH4
BORX
GHDC
STAT5B
STAT5A
STAT3
PTRF
ATP6V0A1
NAGLU
HSD17B1
COASY
MLX
PSMC3IP
FAM134C
TUBG1
TUBG2
PLEKHH3
CCR10
CNTNAP1
EZH1
RAMP2
VPS25
WNK4
COA3
CNTD1
BECN1
42.6
42.8
43.0
43.2
43.4
Base Position (Mbp)

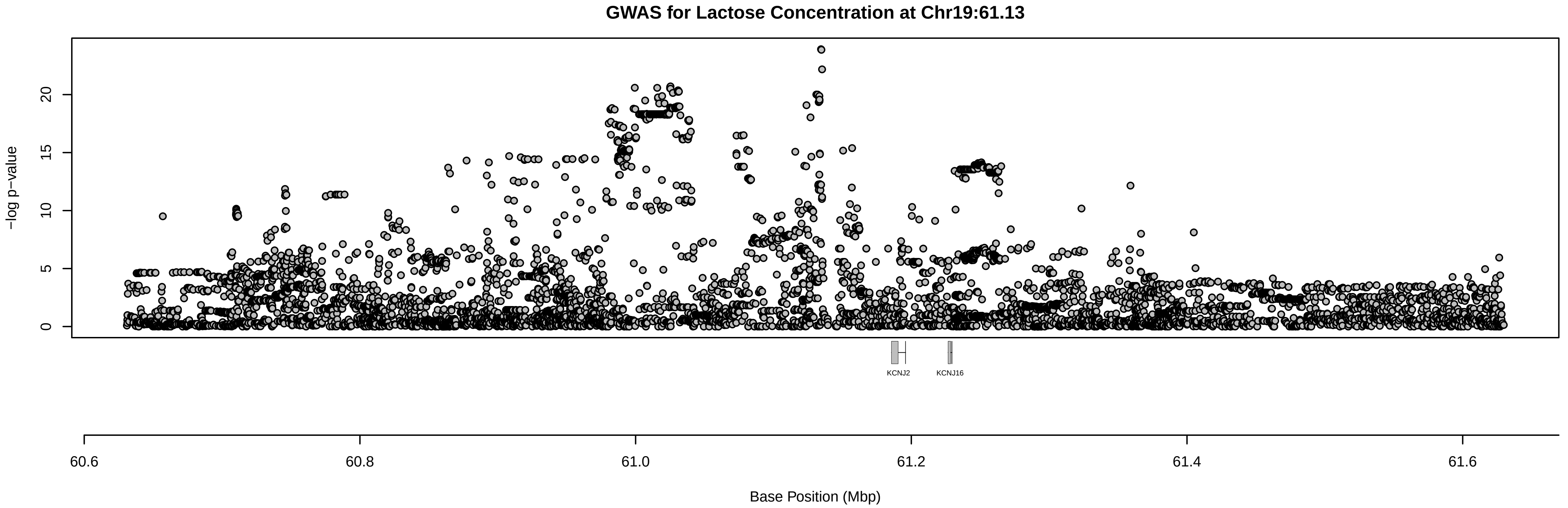
GWAS for Lactose Concentration at Chr19:61.13
−log p−value
0
5
10
15
20
KCNJ2
KCNJ16
60.6
60.8
61.0
61.2
61.4
61.6
Base Position (Mbp)

GWAS for Lactose Concentration at Chr20:58.45

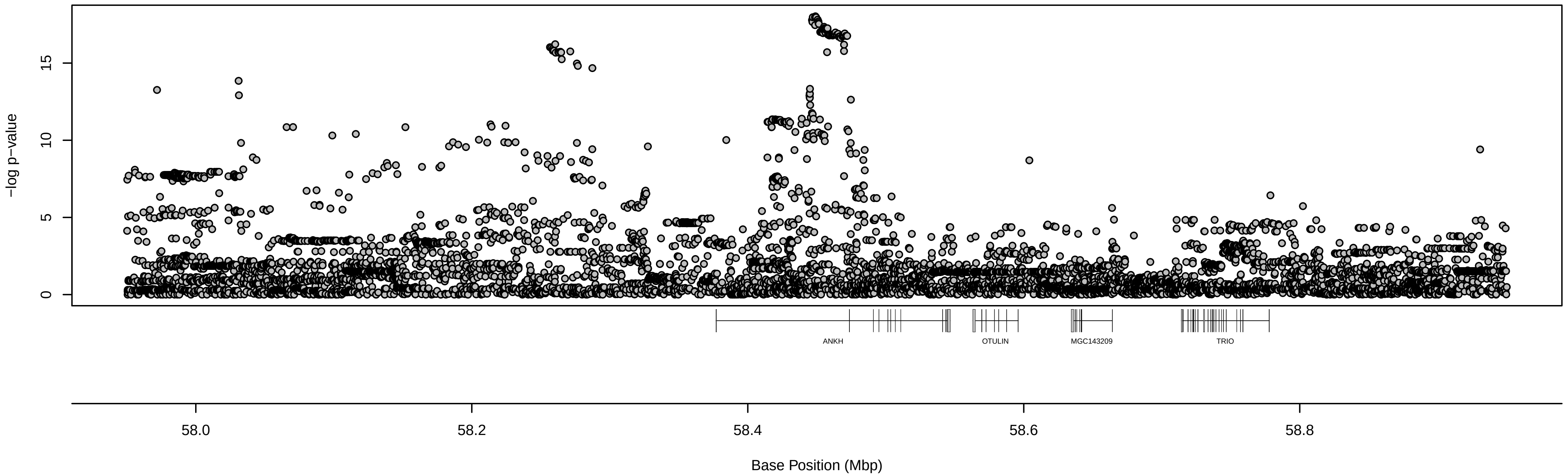

GWAS for Lactose Concentration at Chr27:36.21

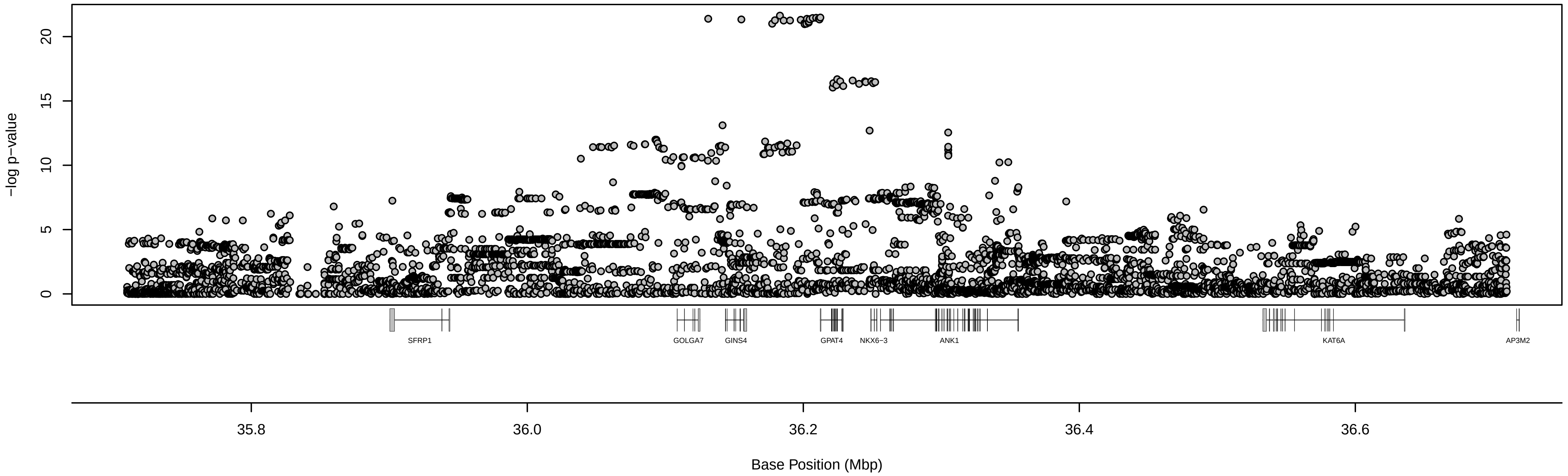

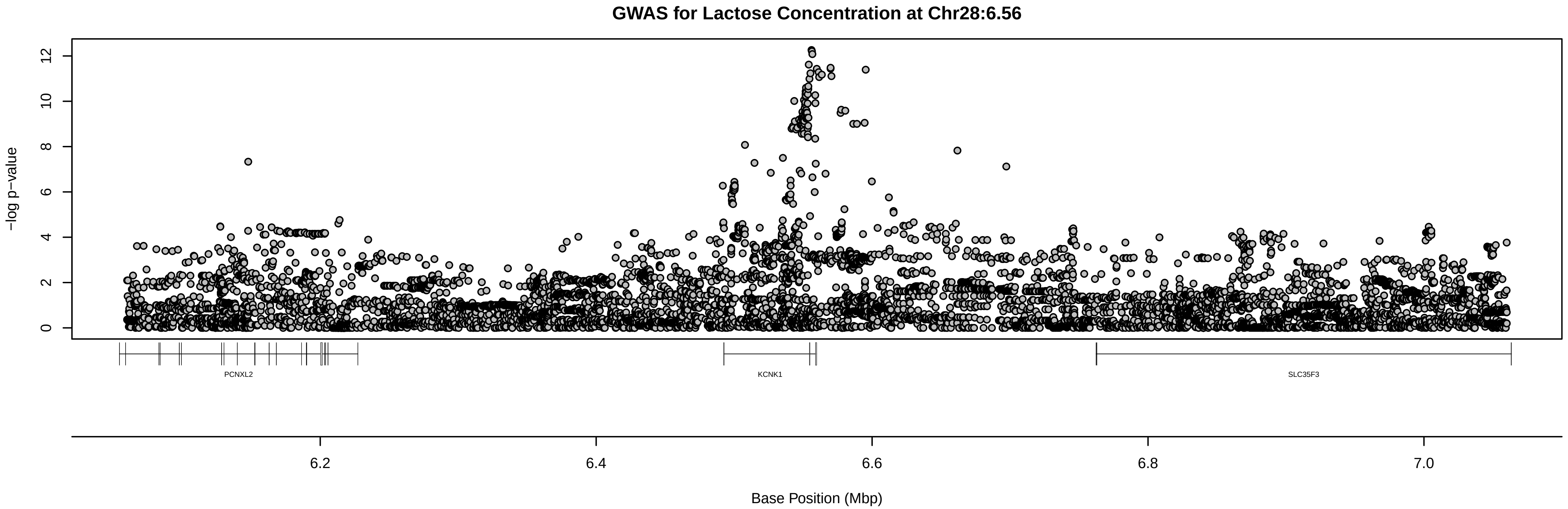

GWAS for Lactose Concentration at Chr28:6.56
−log p−value
0
2
4
6
8
10
12
PCNXL2
KCNK1
SLC35F3
6.2
6.4
6.6
6.8
7.0
Base Position (Mbp)

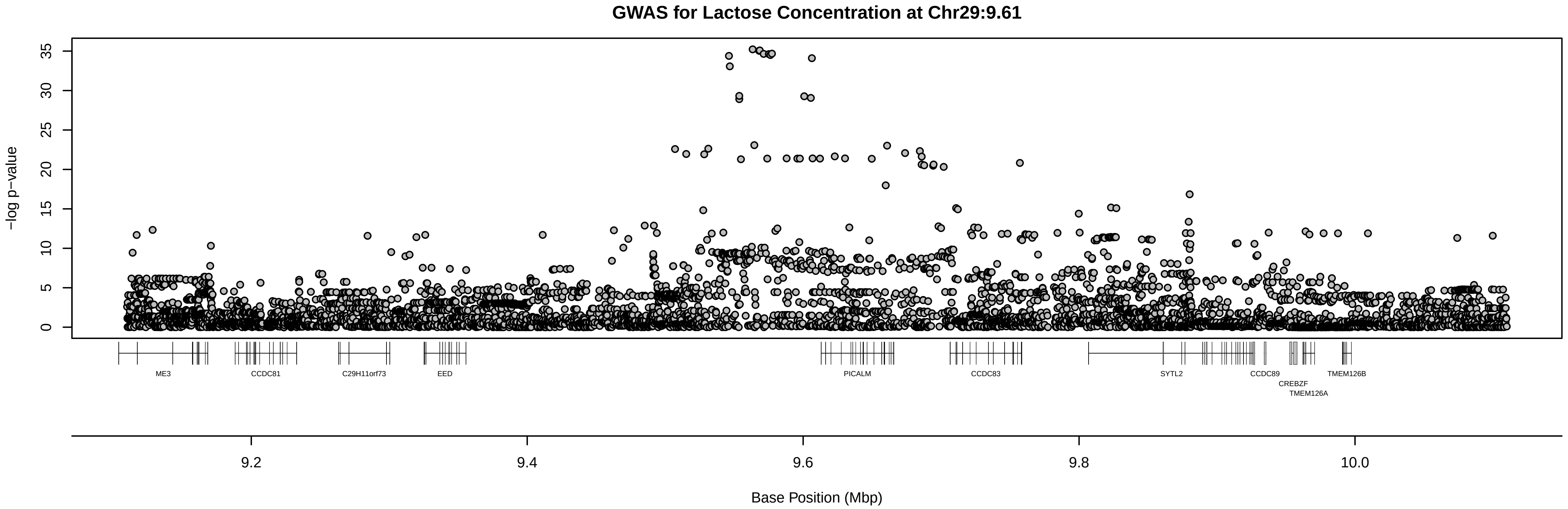

GWAS for Lactose Concentration at Chr29:9.61
−log p−value
0
5
10
15
20
25
30
35
ME3
CCDC81
C29H11orf73
EED
PICALM
CCDC83
SYTL2
CCDC89
CREBZF
TMEM126A
TMEM126B
9.2
9.4
9.6
9.8
10.0
Base Position (Mbp)

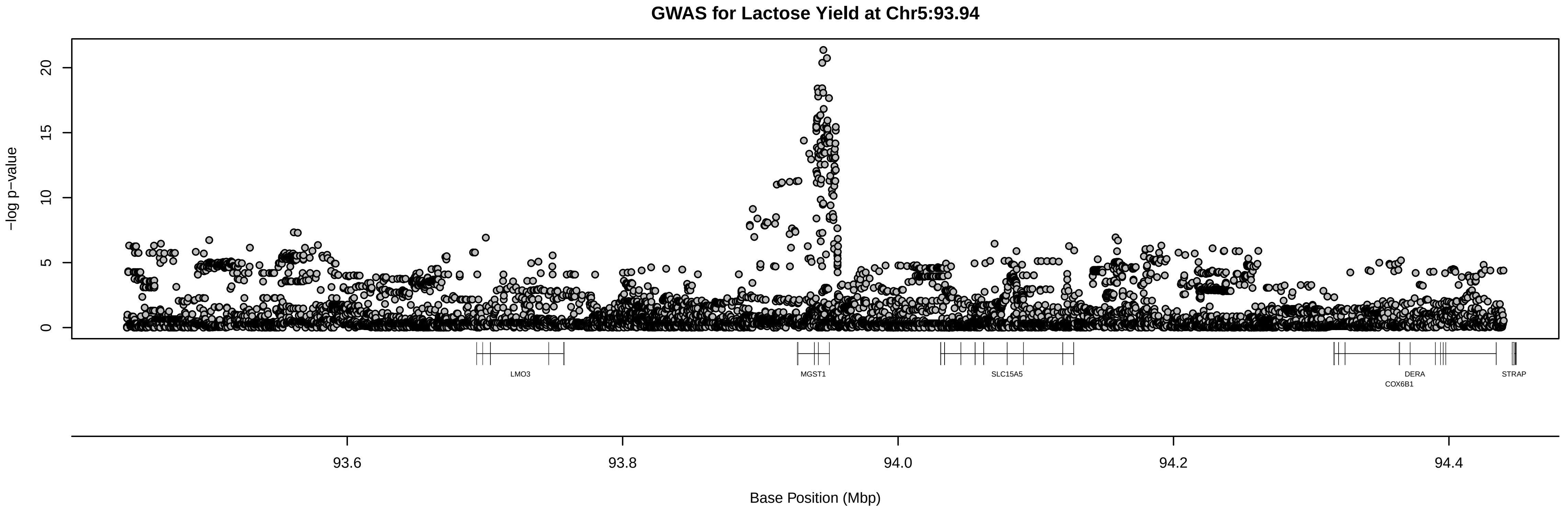
GWAS for Lactose Yield at Chr5:93.94
−log p−value
0
5
10
15
20
LMO3
MGST1
SLC15A5
DERA
COX6B1
STRAP
93.6
93.8
94.0
94.2
94.4
Base Position (Mbp)

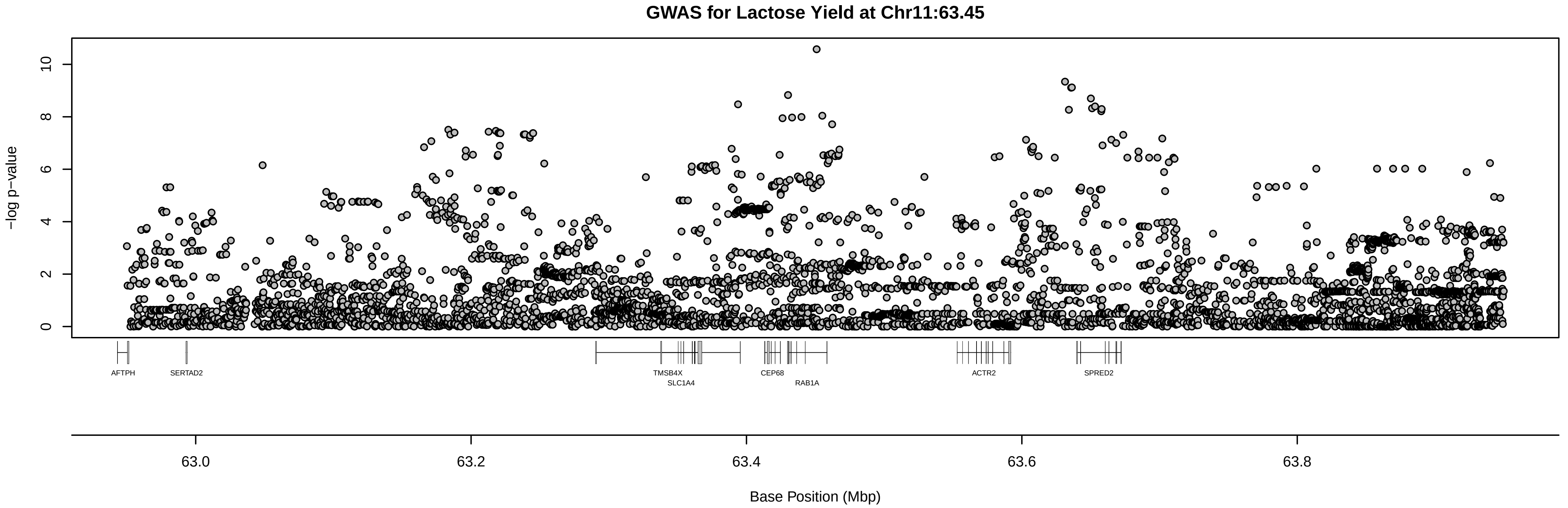
GWAS for Lactose Yield at Chr11:63.45
−log p−value
0
2
4
6
8
10
AFTPH
SERTAD2
TMSB4X
SLC1A4
CEP68
RAB1A
ACTR2
SPRED2
63.0
63.2
63.4
63.6
63.8
Base Position (Mbp)

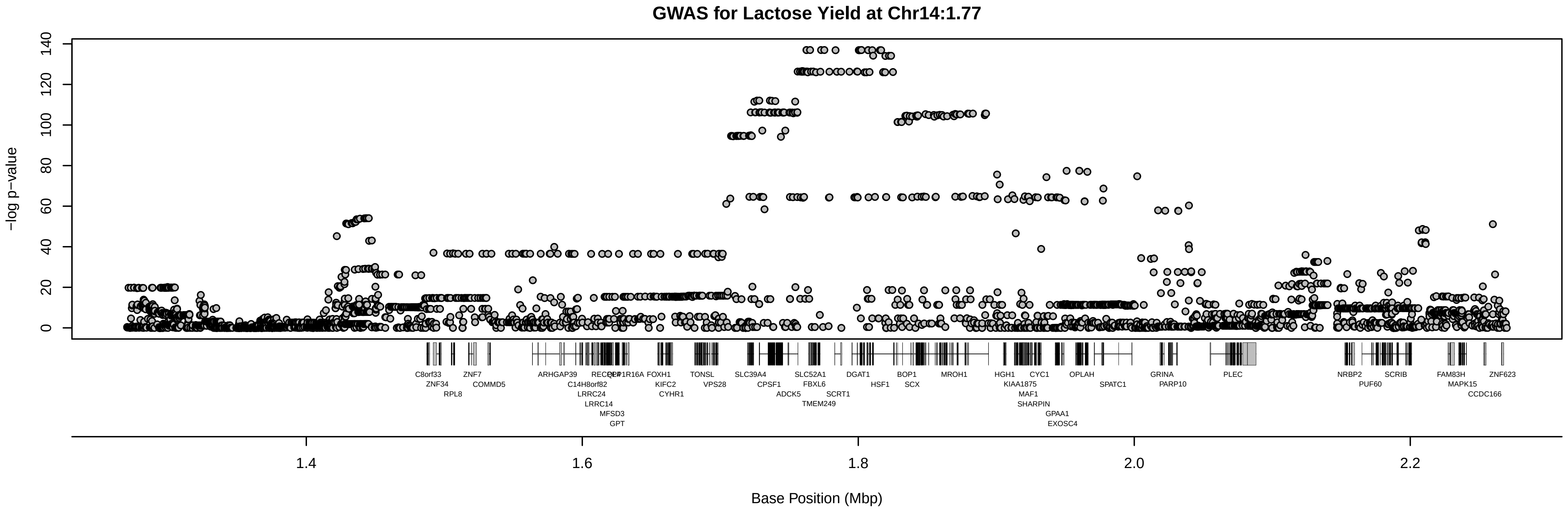
GWAS for Lactose Yield at Chr14:1.77
−log p−value
0
20
40
60
80
100
120
140
C8orf33
ZNF34
RPL8
ZNF7
COMMD5
ARHGAP39
C14H8orf82
LRRC24
LRRC14
RECQL4
PPP1R16A
MFSD3
GPT
FOXH1
KIFC2
CYHR1
TONSL
VPS28
SLC39A4
CPSF1
ADCK5
SLC52A1
FBXL6
TMEM249
SCRT1
DGAT1
HSF1
BOP1
SCX
MROH1
HGH1
KIAA1875
MAF1
SHARPIN
CYC1
GPAA1
EXOSC4
OPLAH
SPATC1
GRINA
PARP10
PLEC
NRBP2
PUF60
SCRIB
FAM83H
MAPK15
ZNF623
CCDC166
1.4
1.6
1.8
2.0
2.2
Base Position (Mbp)

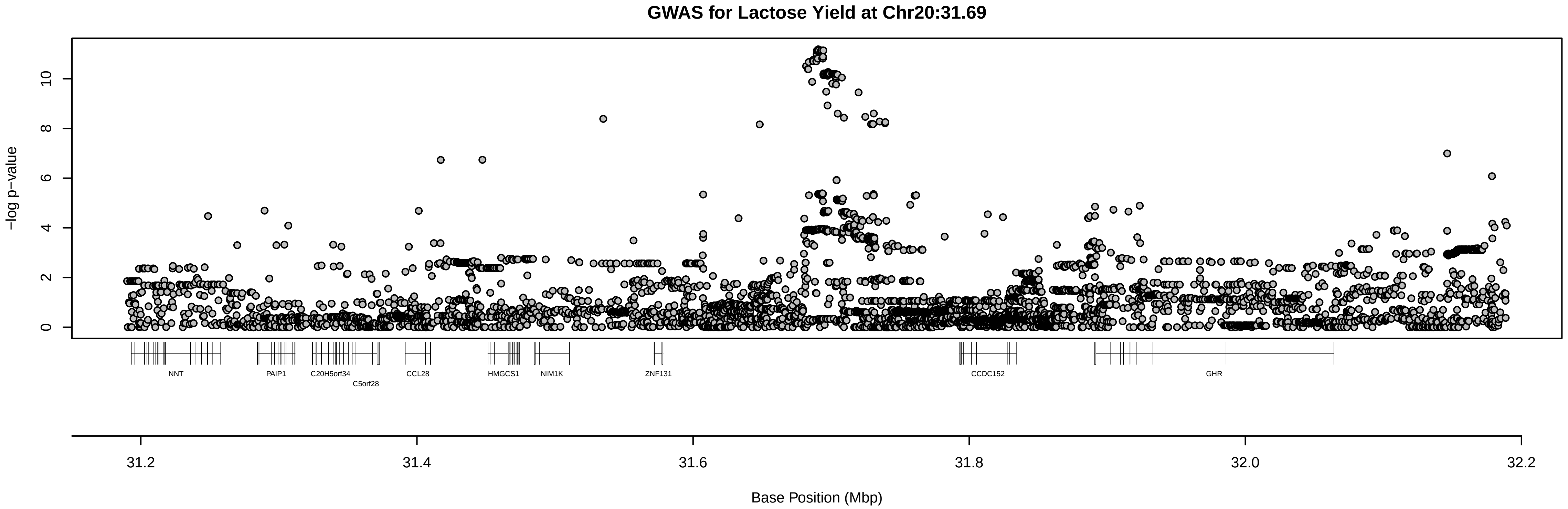
GWAS for Lactose Yield at Chr20:31.69
−log p−value
0
2
4
6
8
10
NNT
PAIP1
C20H5orf34
C5orf28
CCL28
HMGCS1
NIM1K
ZNF131
CCDC152
GHR
31.2
31.4
31.6
31.8
32.0
32.2
Base Position (Mbp)

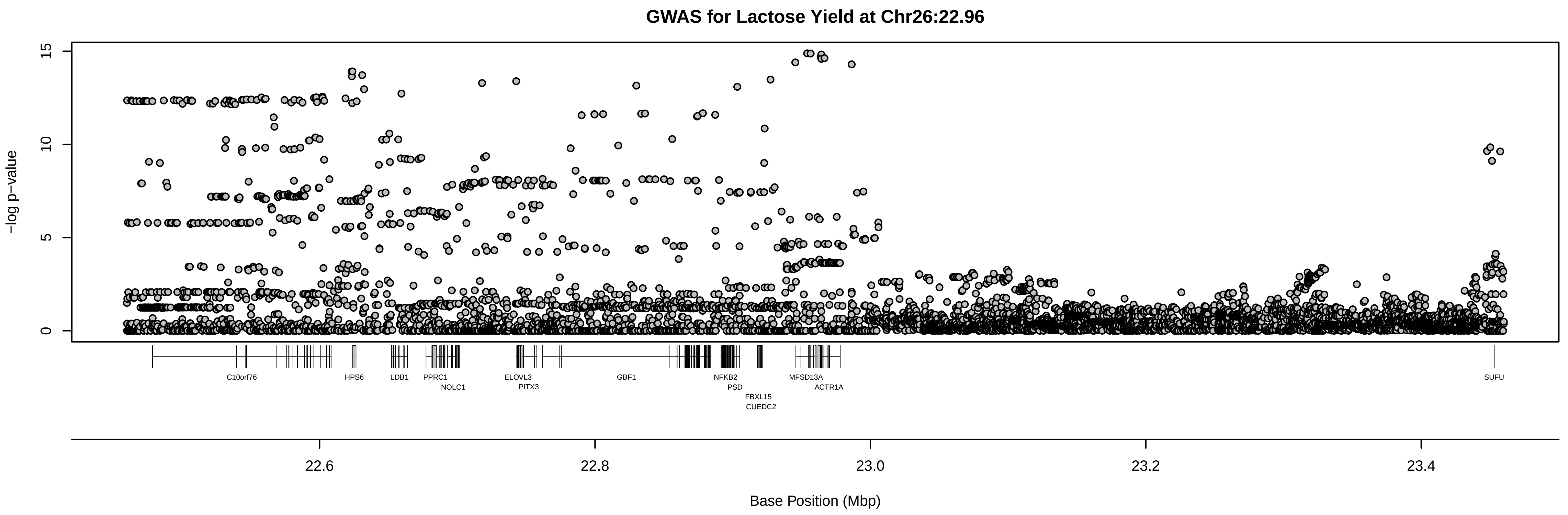
GWAS for Lactose Yield at Chr26:22.96
−log p−value
0
5
10
15
C10orf76
HPS6
LDB1
PPRC1
NOLC1
ELOVL3
PITX3
GBF1
NFKB2
PSD
FBXL15
CUEDC2
MFSD13A
ACTR1A
SUFU
22.6
22.8
23.0
23.2
23.4
Base Position (Mbp)
