# Supplementary material for: DNA and RNA-sequence based GWAS highlights membrane-transport genes as key modulators of milk lactose content
Source: BMC Genomics. 2017 Dec 15;18:968. doi: 10.1186/s12864-017-4320-3 (PMC5731188; doi:10.1186/s12864-017-4320-3)
Supplement: Supplementary file 6 — Figure S3. WGS resolution for eQTL of all gene located within 1Mbp windows centred on QTL peaks for lactose phenotypes. (PDF 8428 kb) [file 12864_2017_4320_MOESM6_ESM.pdf]

eQTL for ABCG2 (chr6)

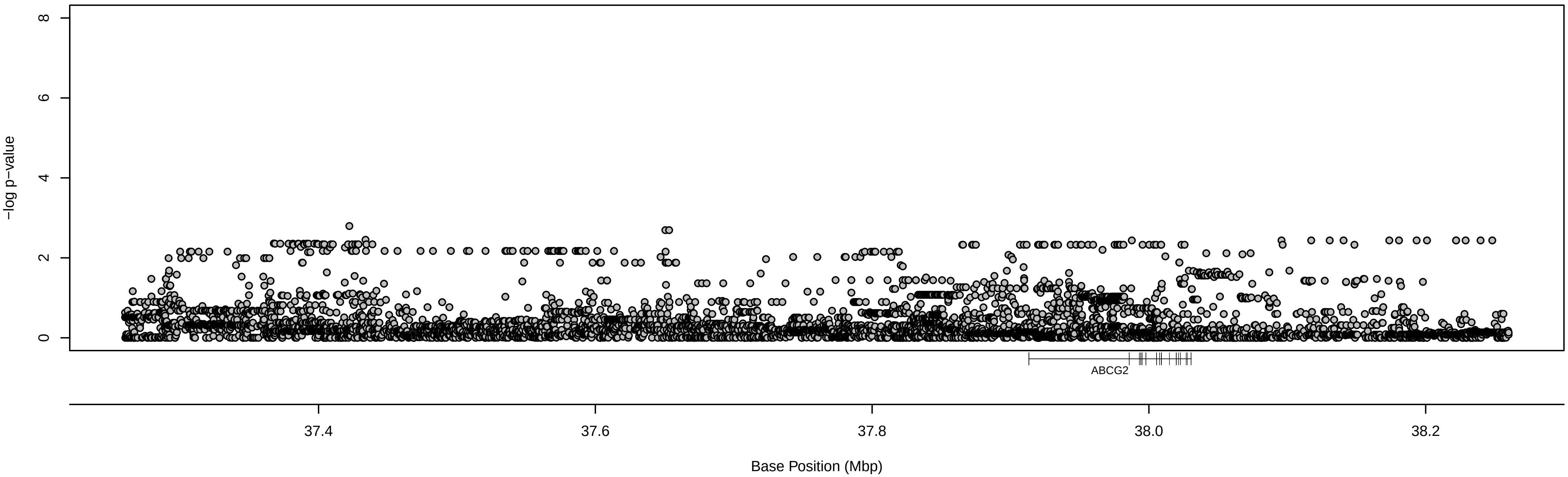

eQTL for ACLY (chr19)

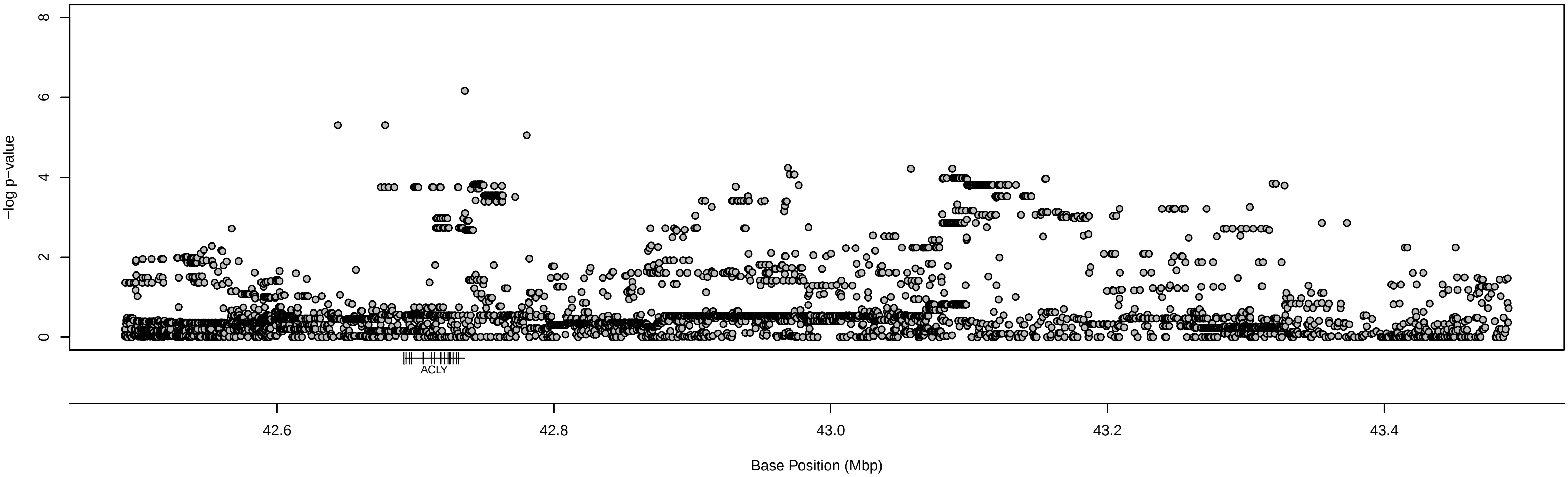

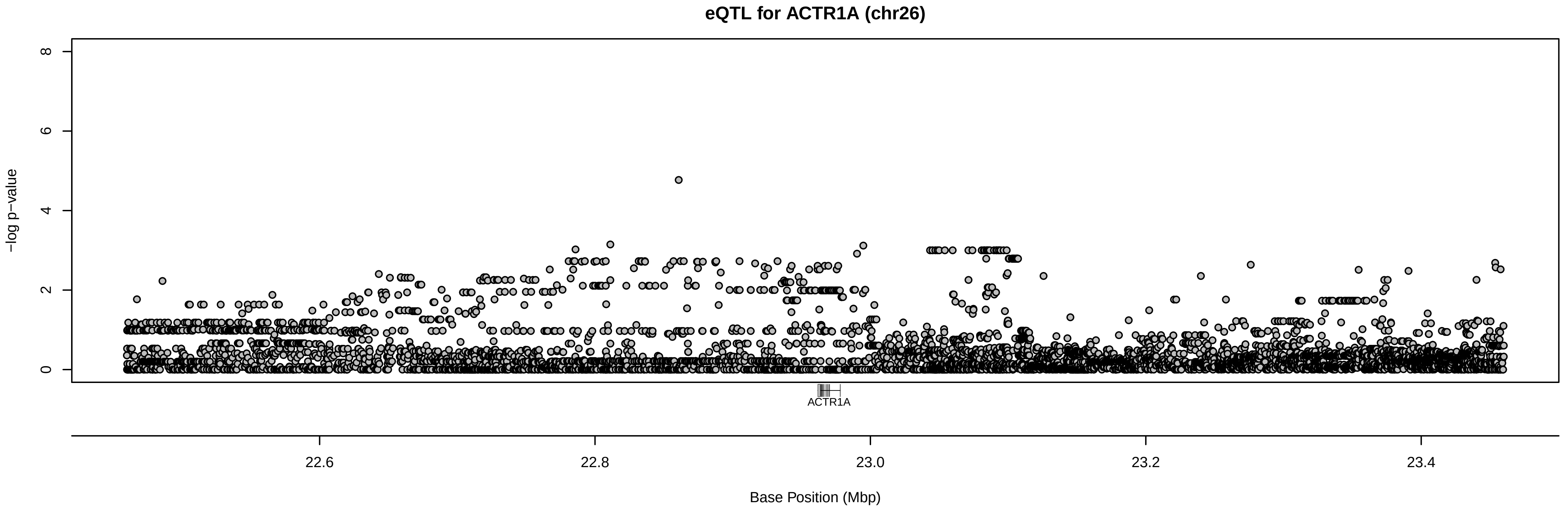
eQTL for ACTR1A (chr26)
−log p−value
0
2
4
6
8
ACTR1A
22.6
22.8
23.0
23.2
23.4
Base Position (Mbp)

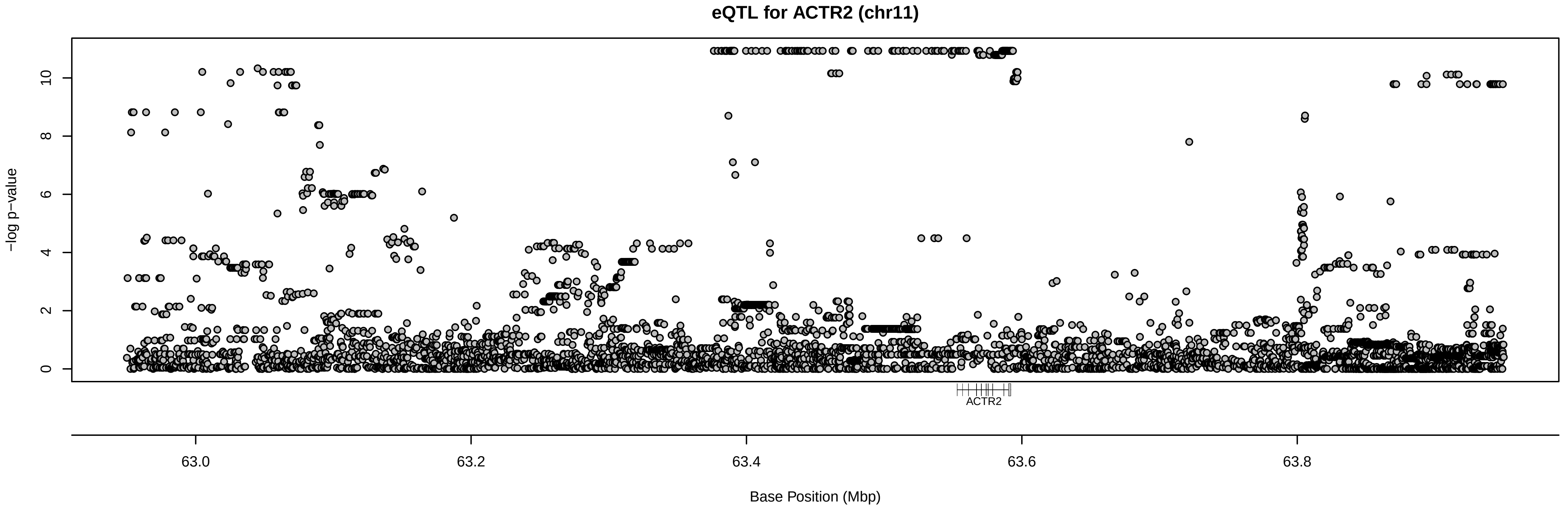
eQTL for ACTR2 (chr11)
−log p−value
0
2
4
6
8
10
ACTR2
63.0
63.2
63.4
63.6
63.8
Base Position (Mbp)

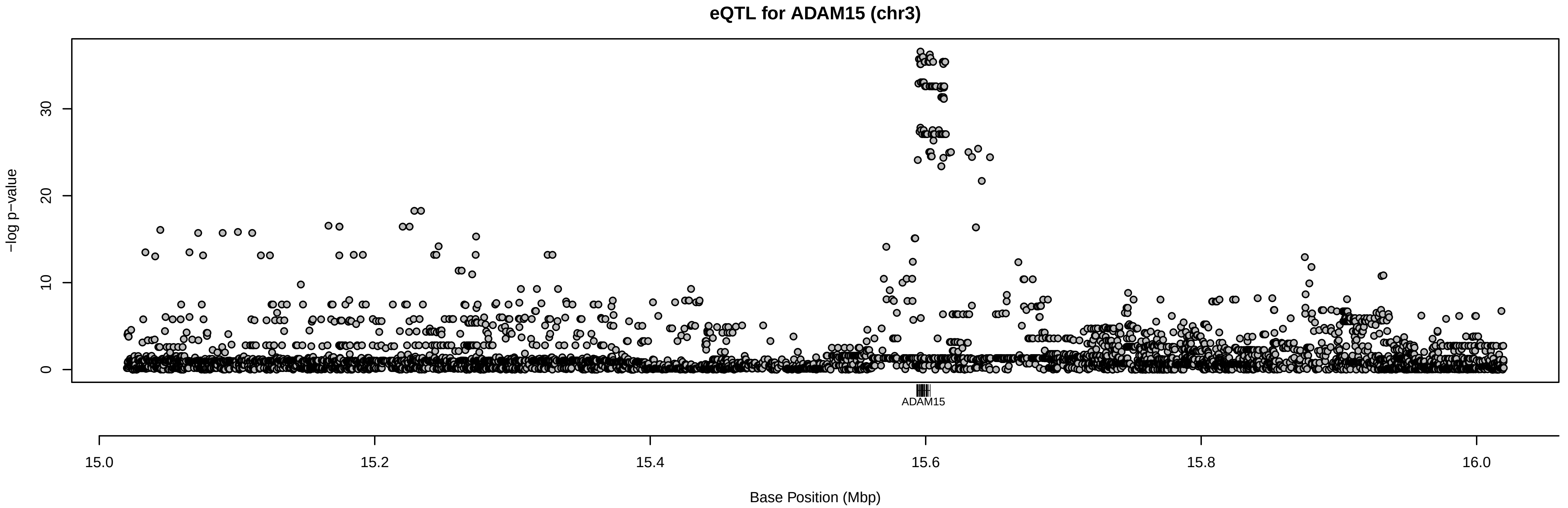
eQTL for ADAM15 (chr3)
−log p−value
0
10
20
30
ADAM15
15.0
15.2
15.4
15.6
15.8
16.0
Base Position (Mbp)

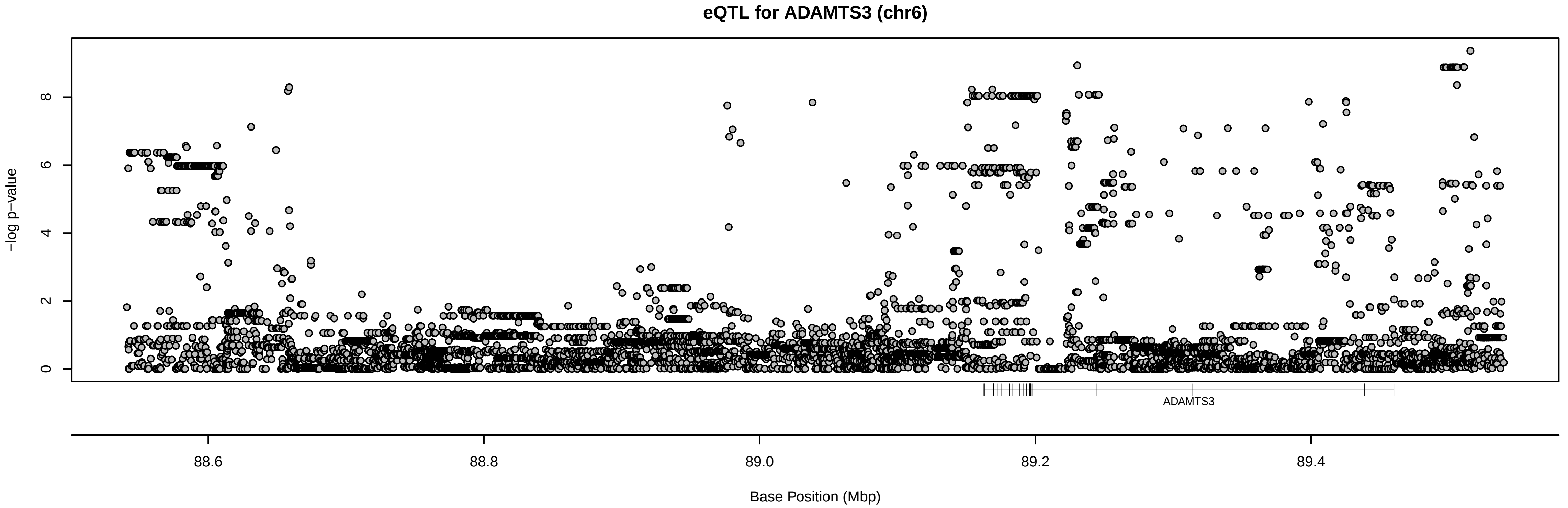
eQTL for ADAMTS3 (chr6)
−log p−value
0
2
4
6
8
ADAMTS3
88.6
88.8
89.0
89.2
89.4
Base Position (Mbp)

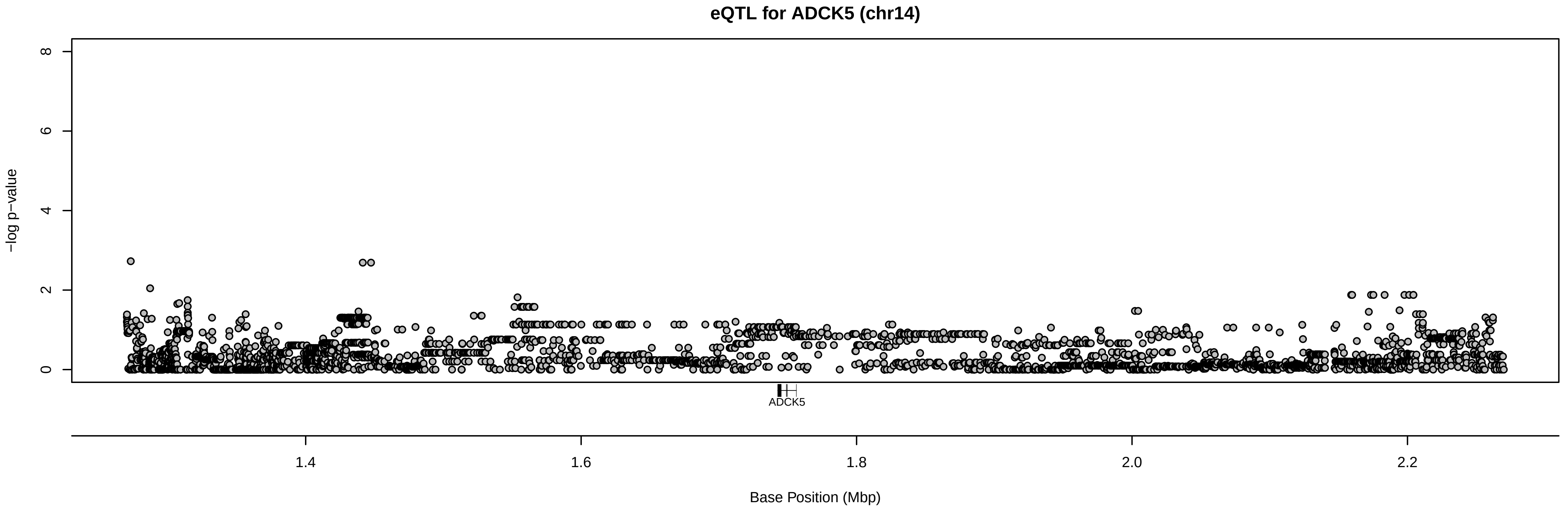

eQTL for ADCK5 (chr14)
−log p−value
0
2
4
6
8
ADCK5
1.4
1.6
1.8
2.0
2.2
Base Position (Mbp)

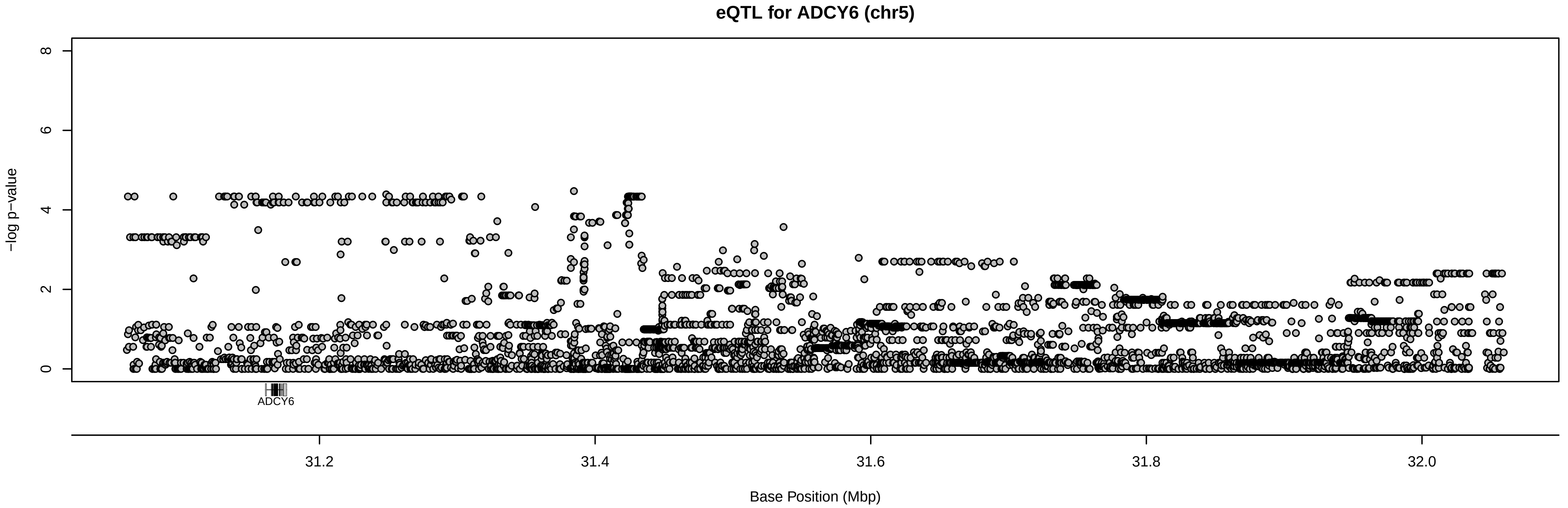

eQTL for ADCY6 (chr5)
−log p−value
0
2
4
6
8
ADCY6
31.2
31.4
31.6
31.8
32.0
Base Position (Mbp)

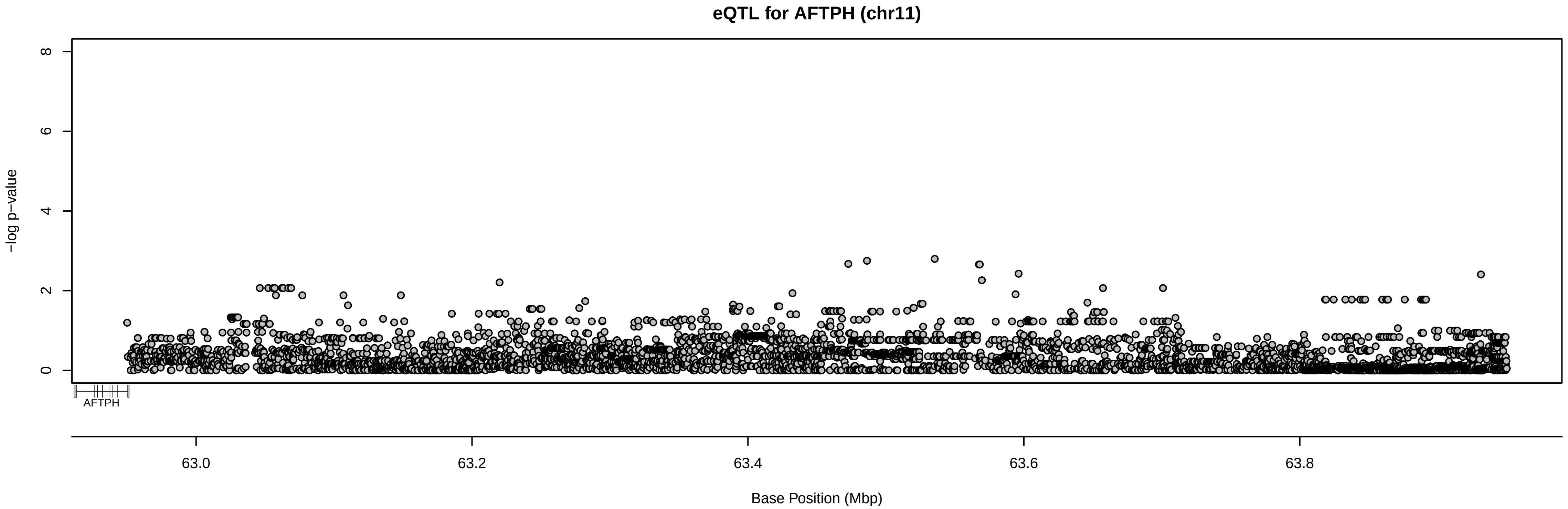

eQTL for AFTPH (chr11)
−log p−value
0
2
4
6
8
AFTPH
63.0
63.2
63.4
63.6
63.8
Base Position (Mbp)

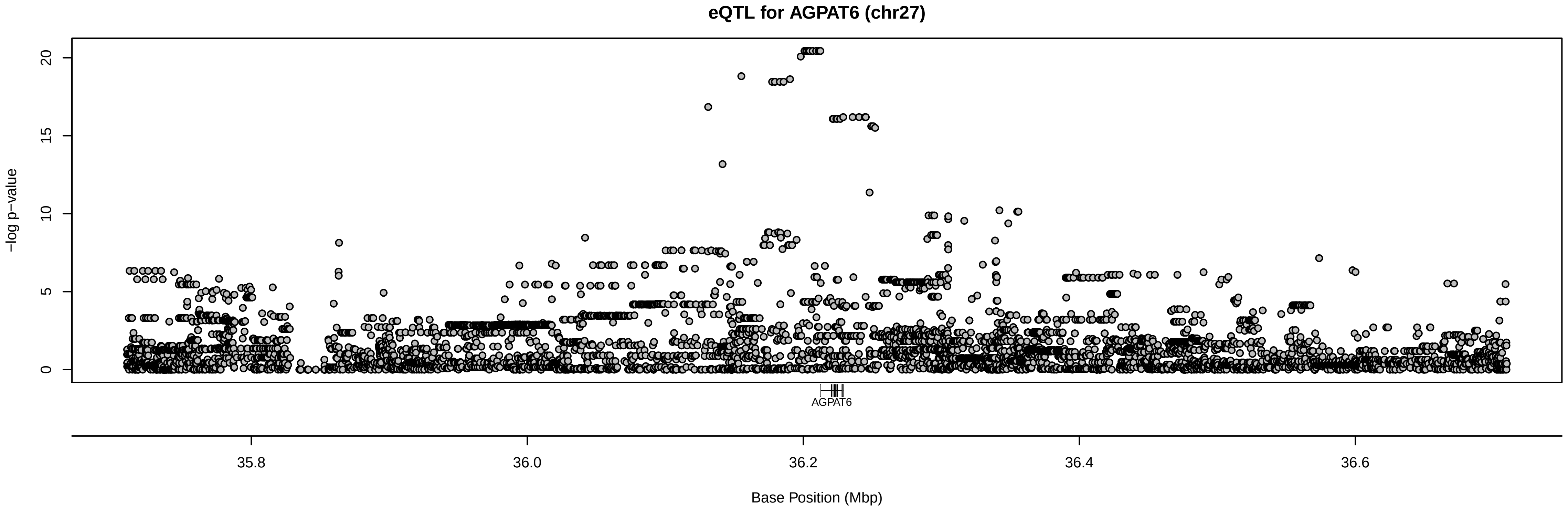

eQTL for AGPAT6 (chr27)
−log p−value
0
5
10
15
20
AGPAT6
35.8
36.0
36.2
36.4
36.6
Base Position (Mbp)

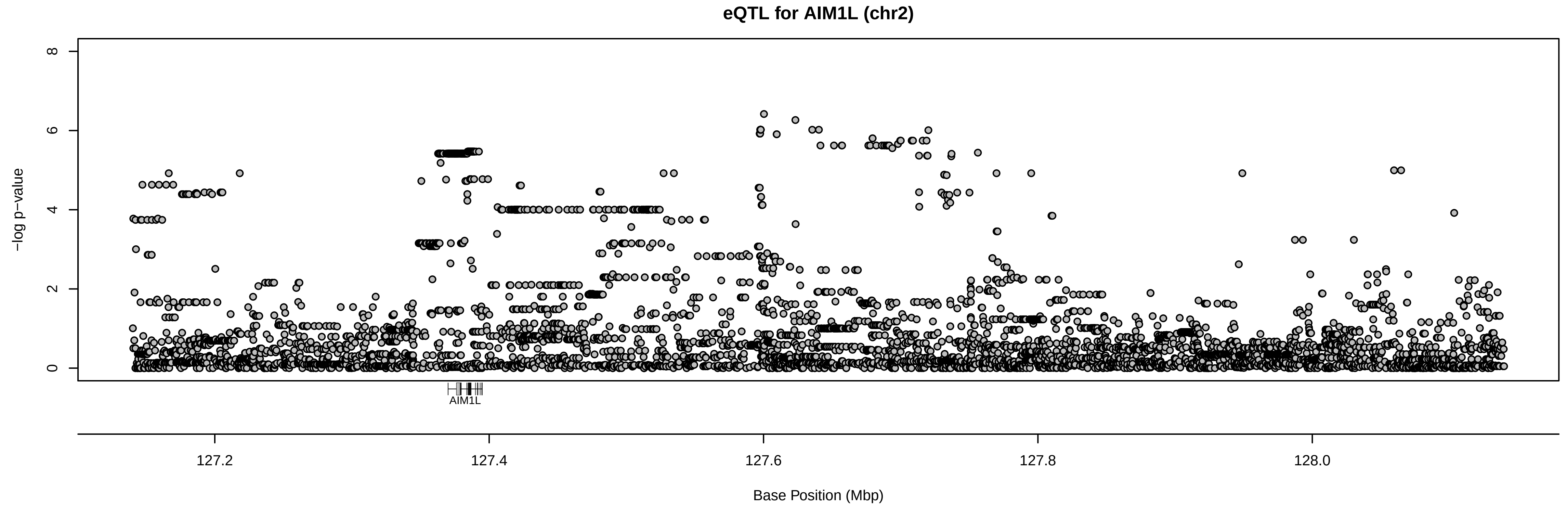
eQTL for AIM1L (chr2)
−log p−value
0
2
4
6
8
AIM1L
127.2
127.4
127.6
127.8
128.0
Base Position (Mbp)

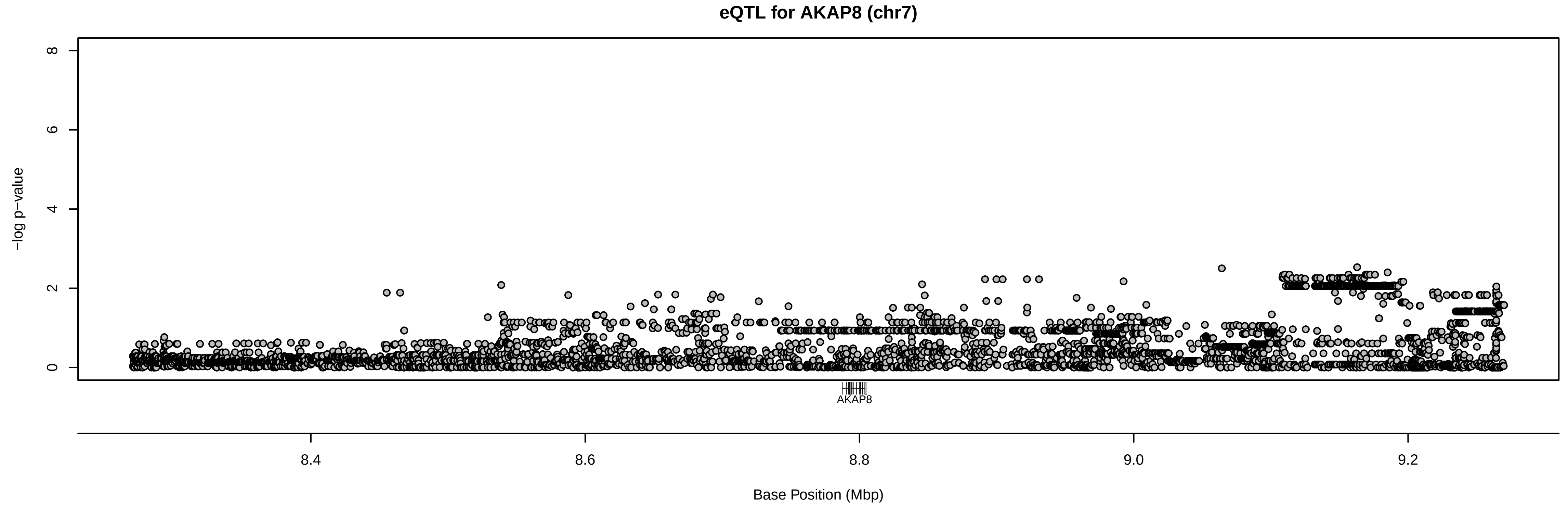
eQTL for AKAP8 (chr7)
−log p−value
0
2
4
6
8
AKAP8
8.4
8.6
8.8
9.0
9.2
Base Position (Mbp)

eQTL for AKAP8L (chr7)

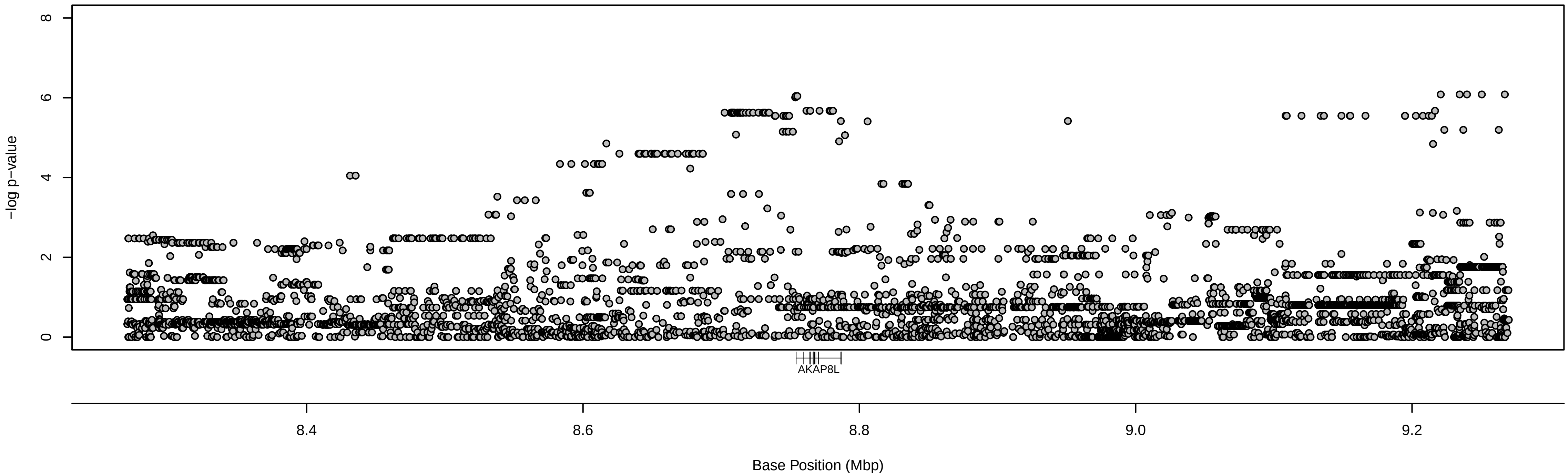

eQTL for ANAPC5 (chr17)

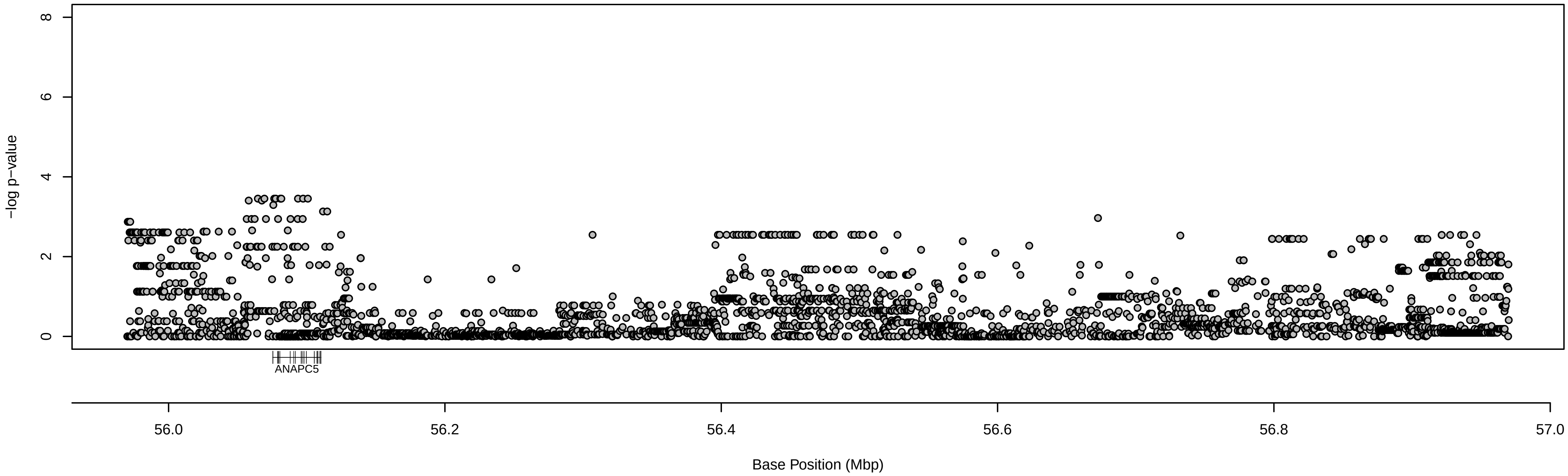

eQTL for ANAPC7 (chr17)

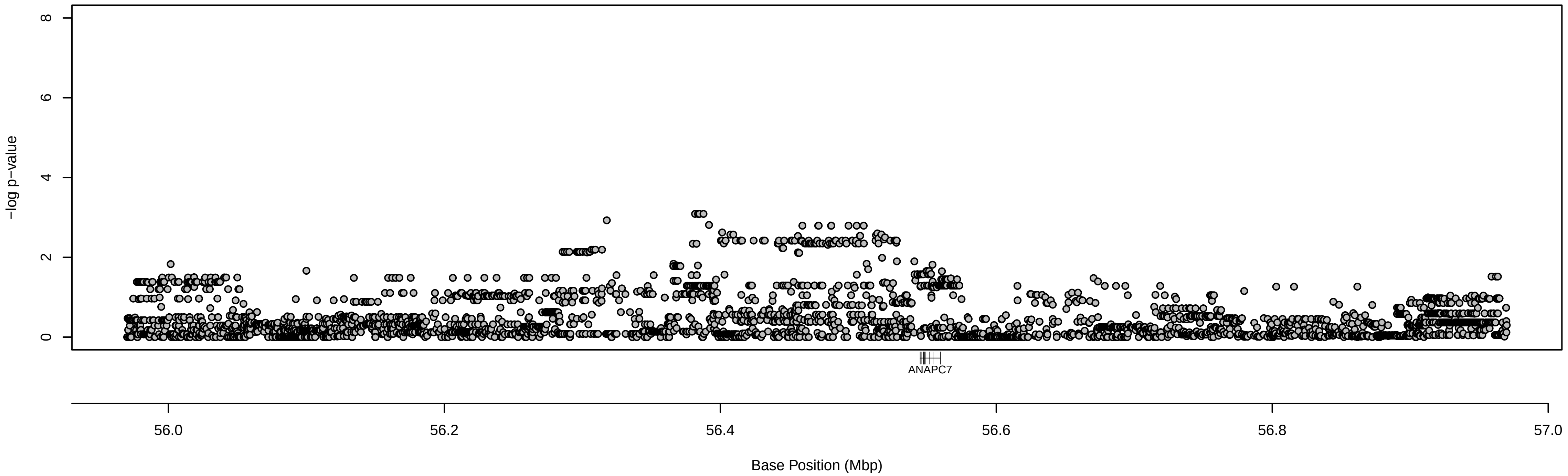

eQTL for ANK1 (chr27)

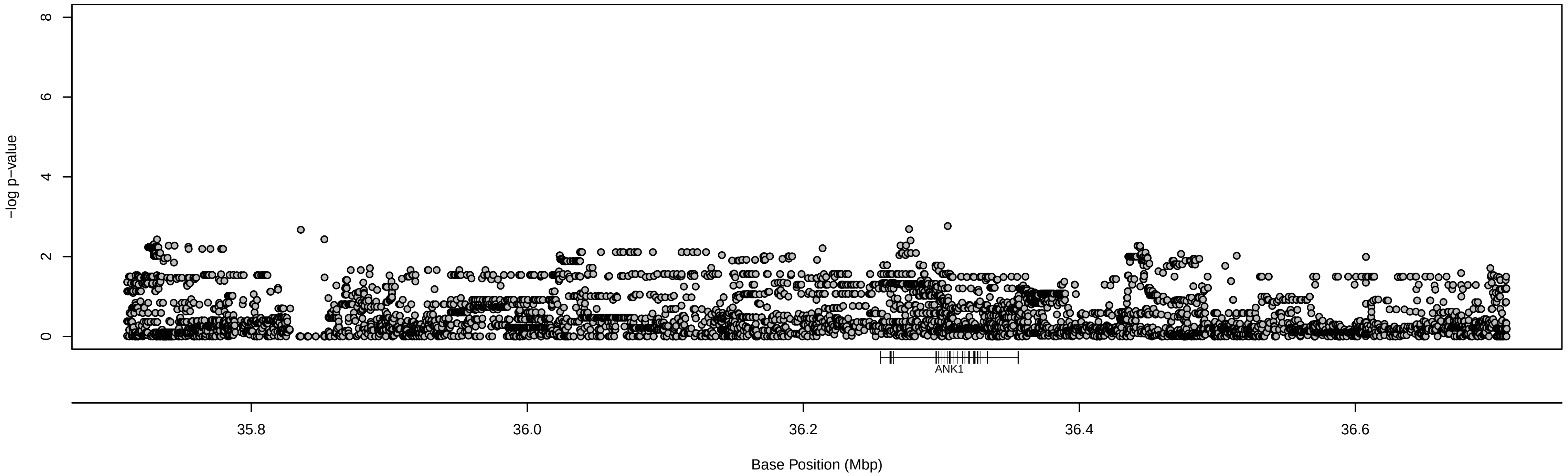

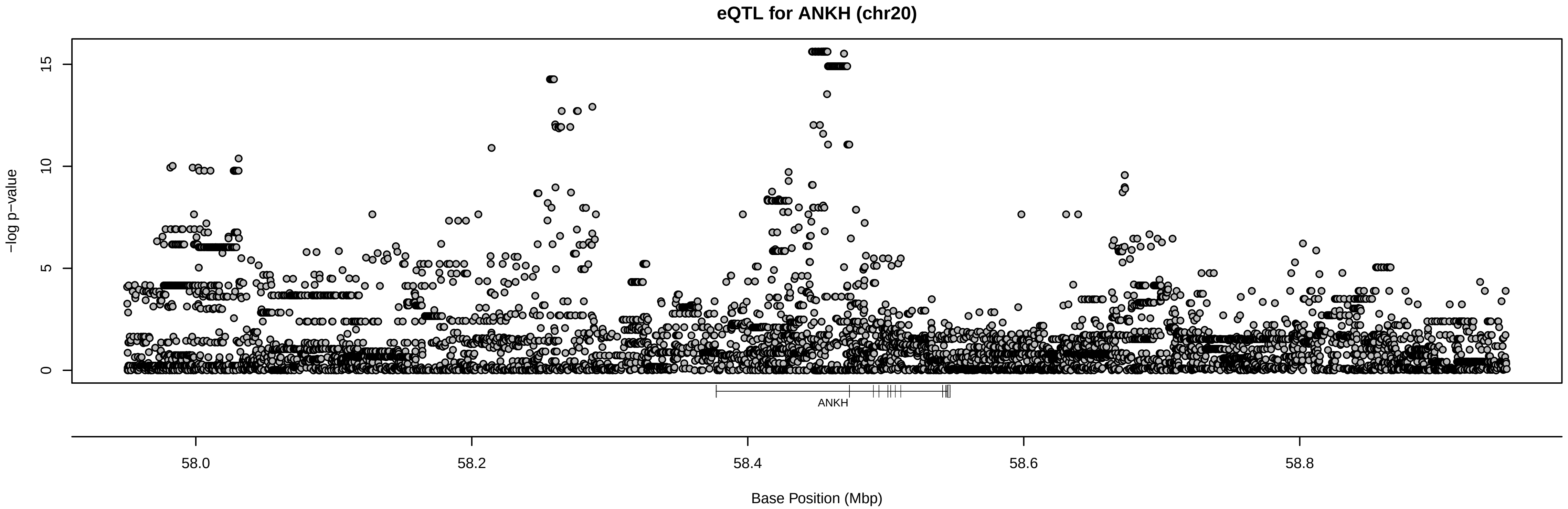
eQTL for ANKH (chr20)
−log p−value
0
5
10
15
ANKH
58.0
58.2
58.4
58.6
58.8
Base Position (Mbp)

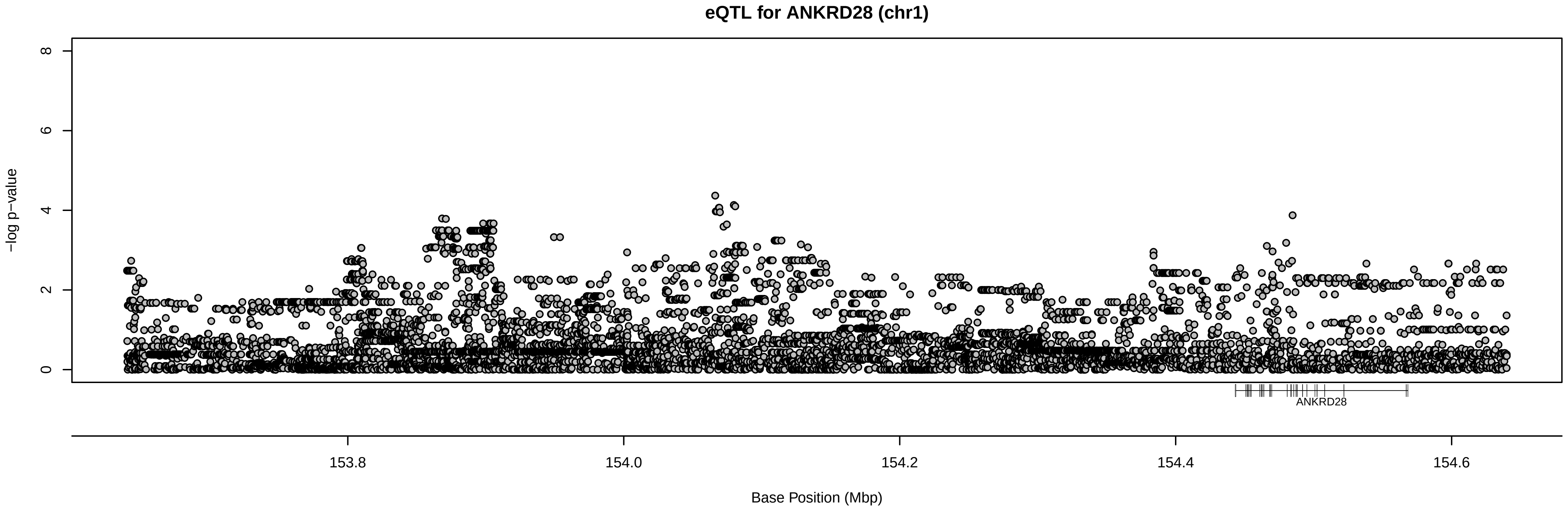
eQTL for ANKRD28 (chr1)
−log p−value
0
2
4
6
8
ANKRD28
153.8
154.0
154.2
154.4
154.6
Base Position (Mbp)

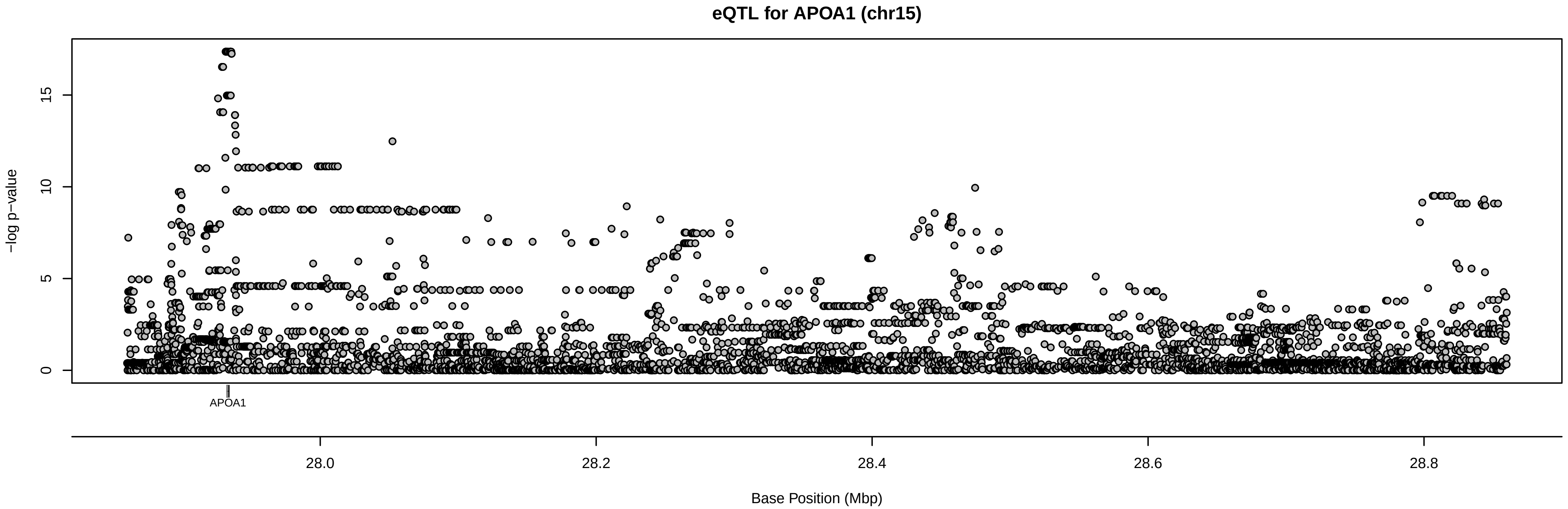

eQTL for APOA1 (chr15)
−log p−value
0
5
10
15
APOA1
28.0
28.2
28.4
28.6
28.8
Base Position (Mbp)

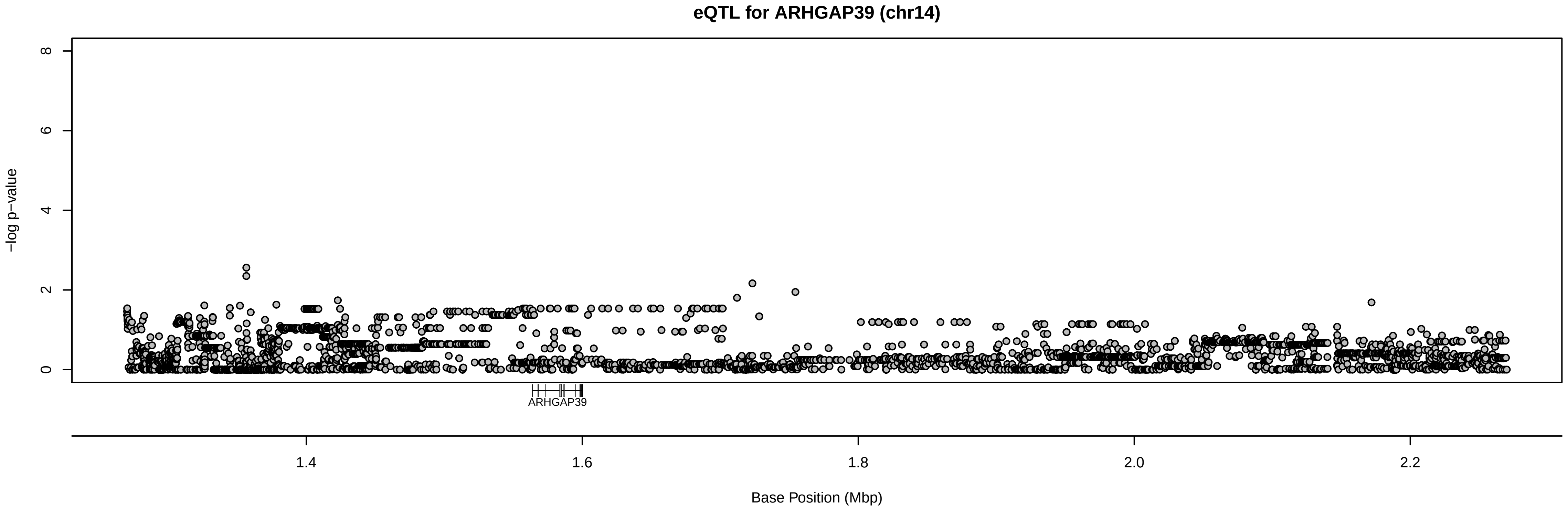

eQTL for ARHGAP39 (chr14)
−log p−value
0
2
4
6
8
ARHGAP39
1.4
1.6
1.8
2.0
2.2
Base Position (Mbp)

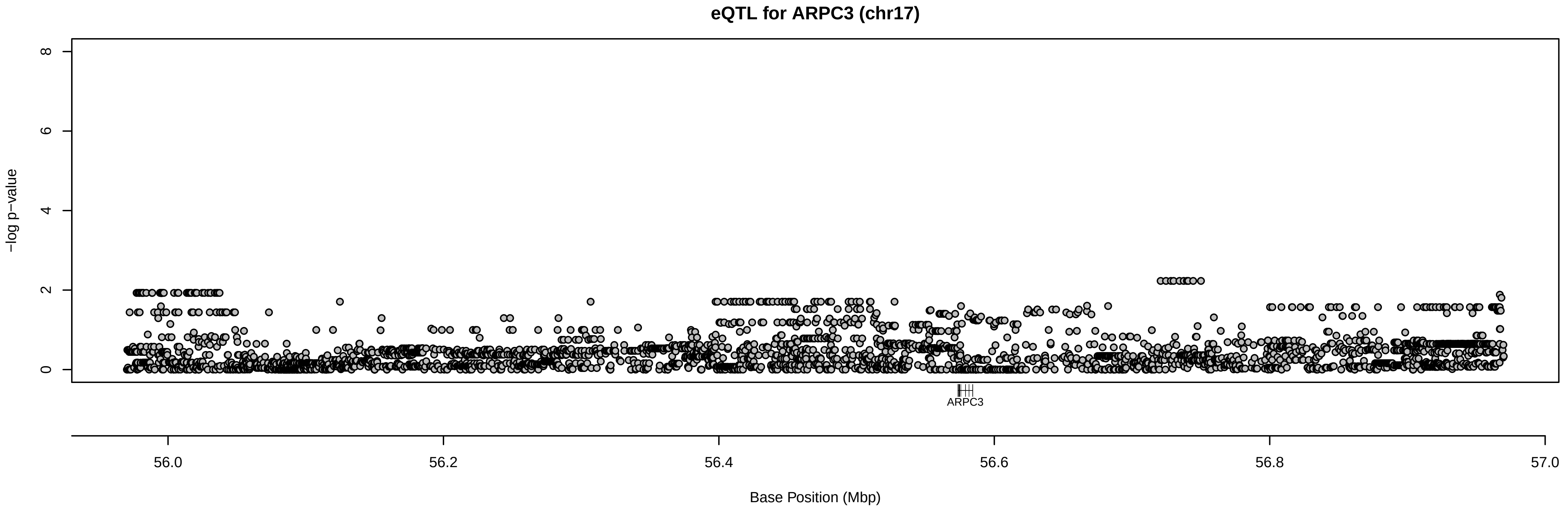
eQTL for ARPC3 (chr17)
−log p−value
0
2
4
6
8
ARPC3
56.0
56.2
56.4
56.6
56.8
57.0
Base Position (Mbp)

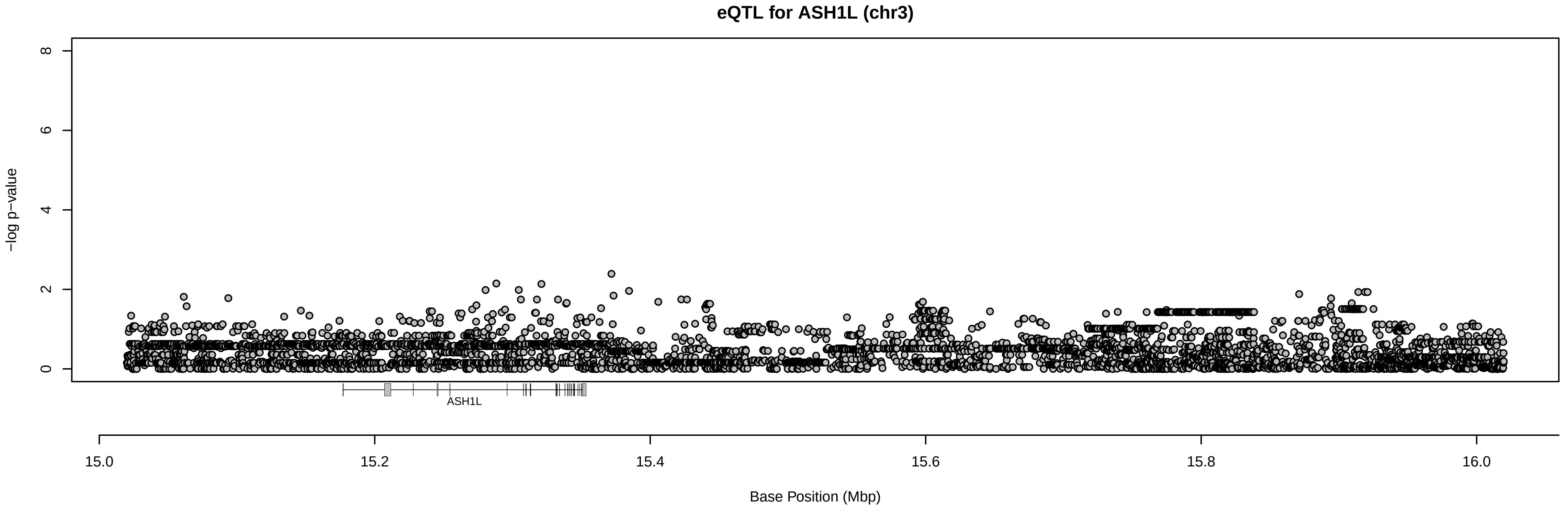
eQTL for ASH1L (chr3)
−log p−value
0
2
4
6
8
ASH1L
15.0
15.2
15.4
15.6
15.8
16.0
Base Position (Mbp)

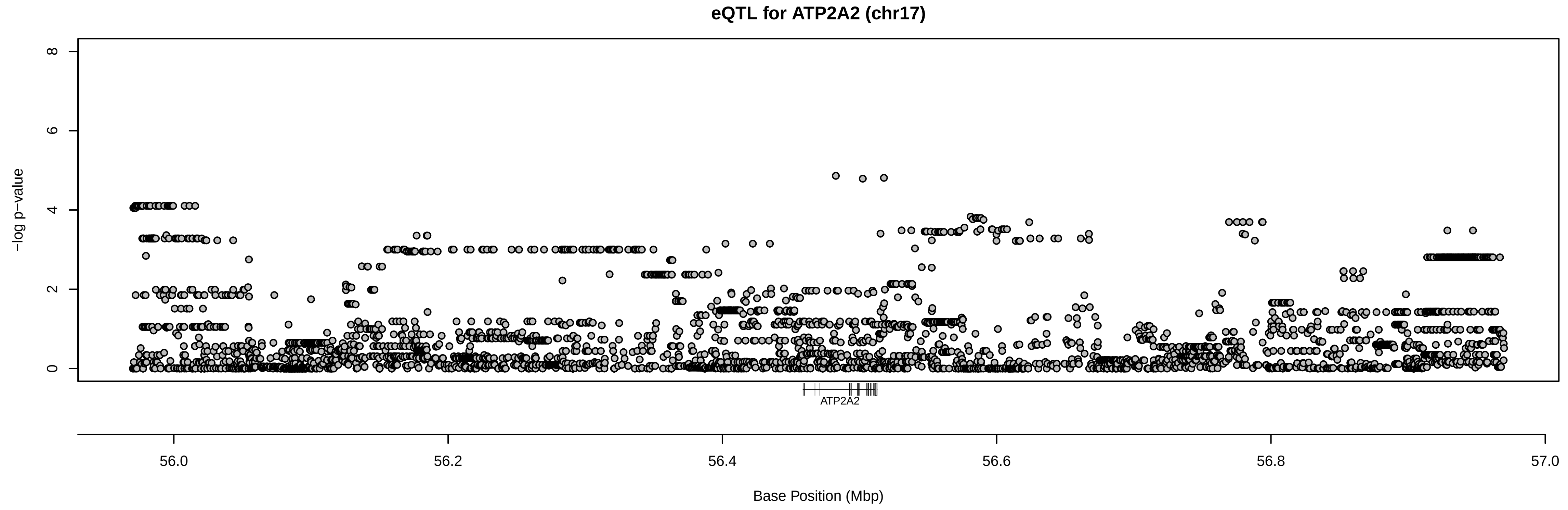
eQTL for ATP2A2 (chr17)
−log p−value
0
2
4
6
8
ATP2A2
56.0
56.2
56.4
56.6
56.8
57.0
Base Position (Mbp)

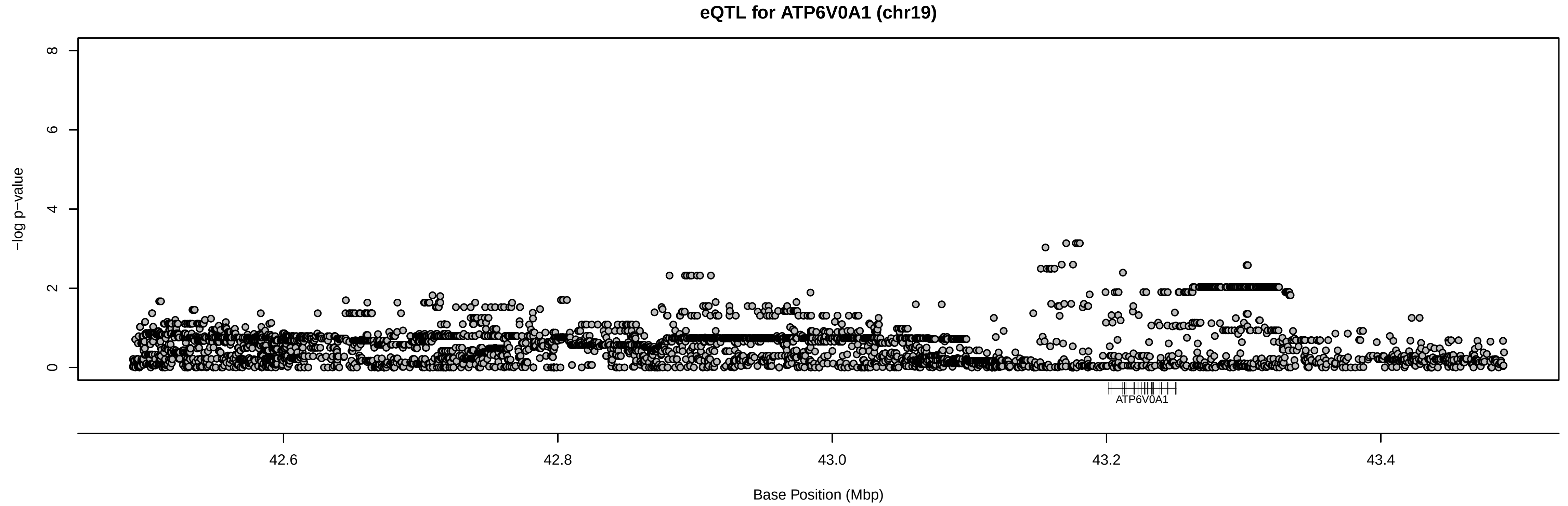
eQTL for ATP6V0A1 (chr19)
−log p−value
0
2
4
6
8
ATP6V0A1
42.6
42.8
43.0
43.2
43.4
Base Position (Mbp)

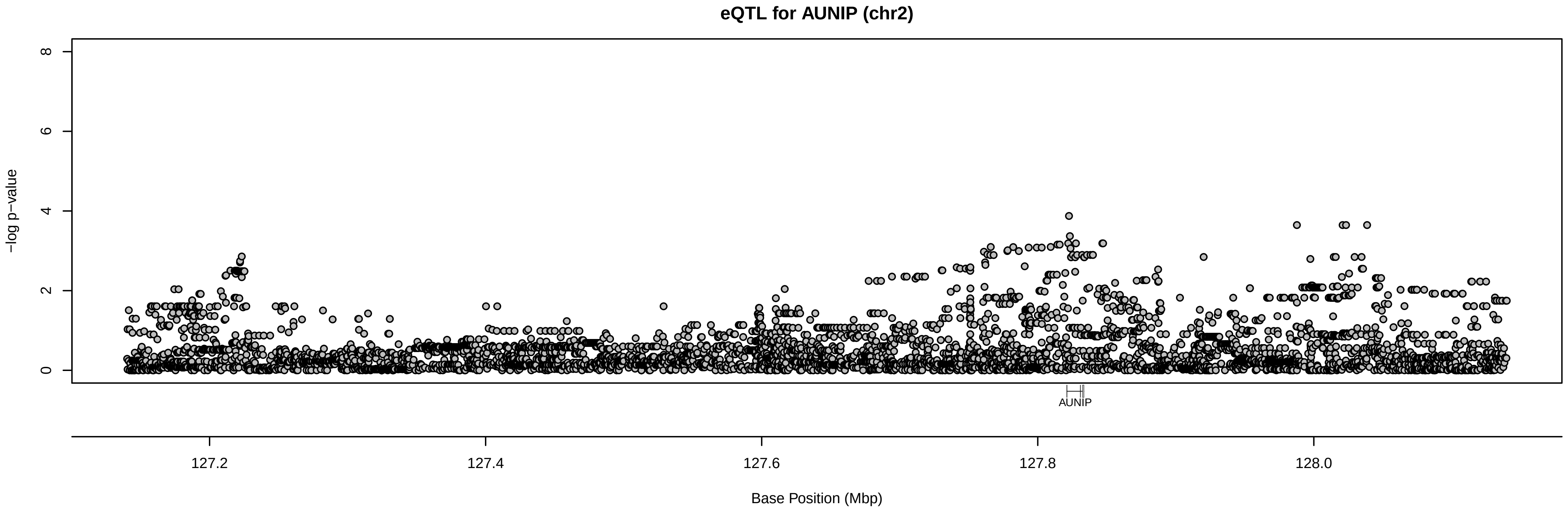
eQTL for AUNIP (chr2)
−log p−value
0
2
4
6
8
AUNIP
127.2
127.4
127.6
127.8
128.0
Base Position (Mbp)

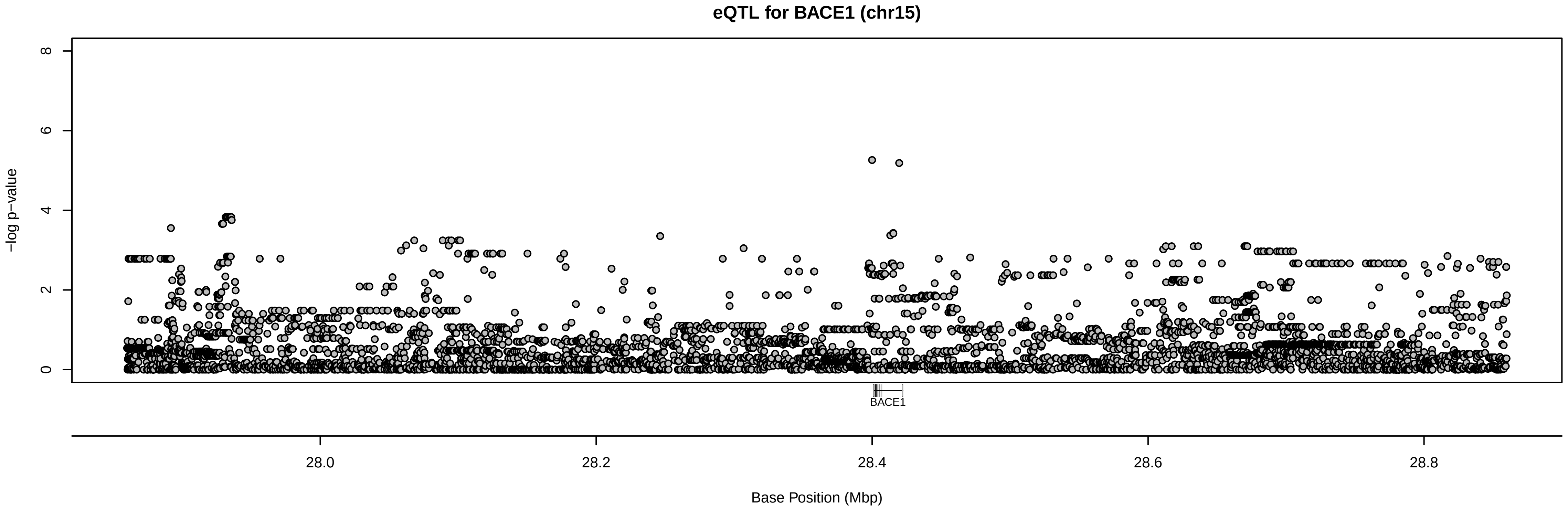
eQTL for BACE1 (chr15)
−log p−value
0
2
4
6
8
BACE1
28.0
28.2
28.4
28.6
28.8
Base Position (Mbp)

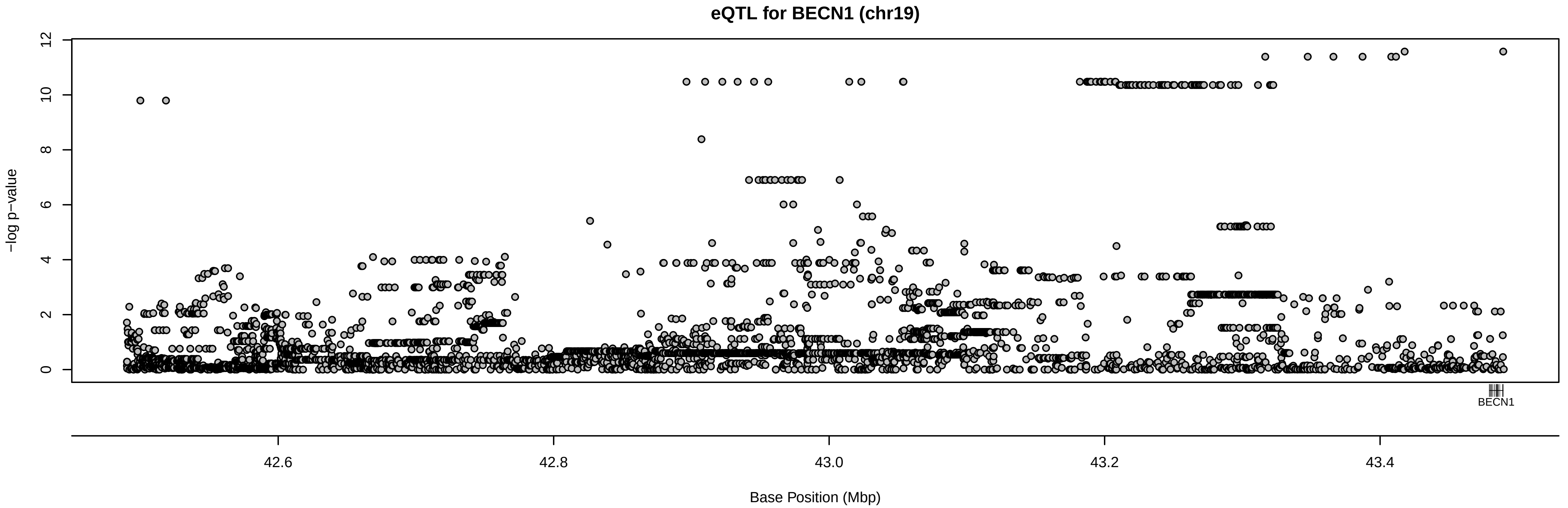
eQTL for BECN1 (chr19)
−log p−value
0
2
4
6
8
10
12
BECN1
42.6
42.8
43.0
43.2
43.4
Base Position (Mbp)

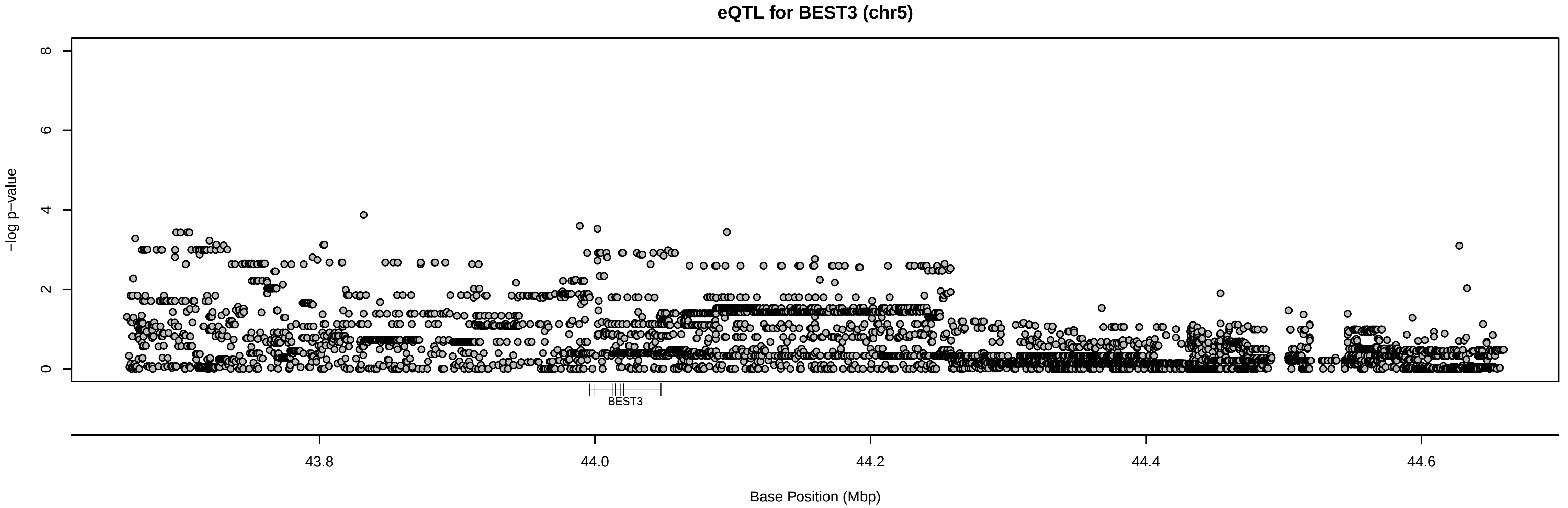
eQTL for BEST3 (chr5)
−log p−value
0
2
4
6
8
BEST3
43.8
44.0
44.2
44.4
44.6
Base Position (Mbp)

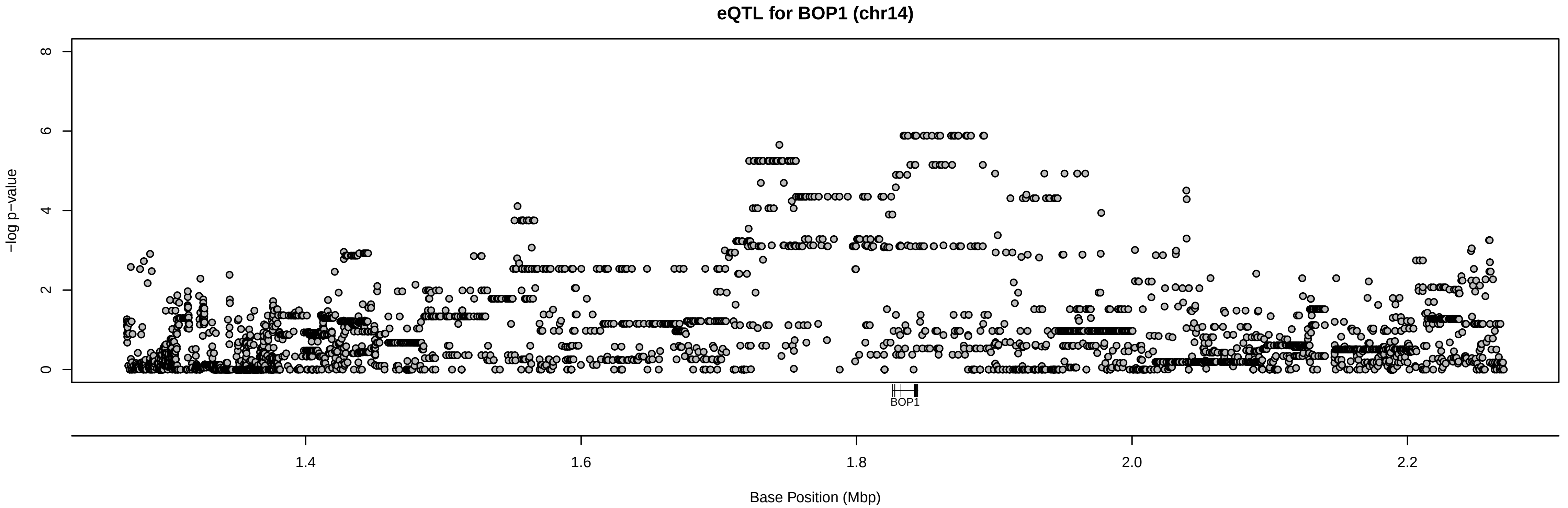
eQTL for BOP1 (chr14)
−log p−value
0
2
4
6
8
BOP1
1.4
1.6
1.8
2.0
2.2
Base Position (Mbp)

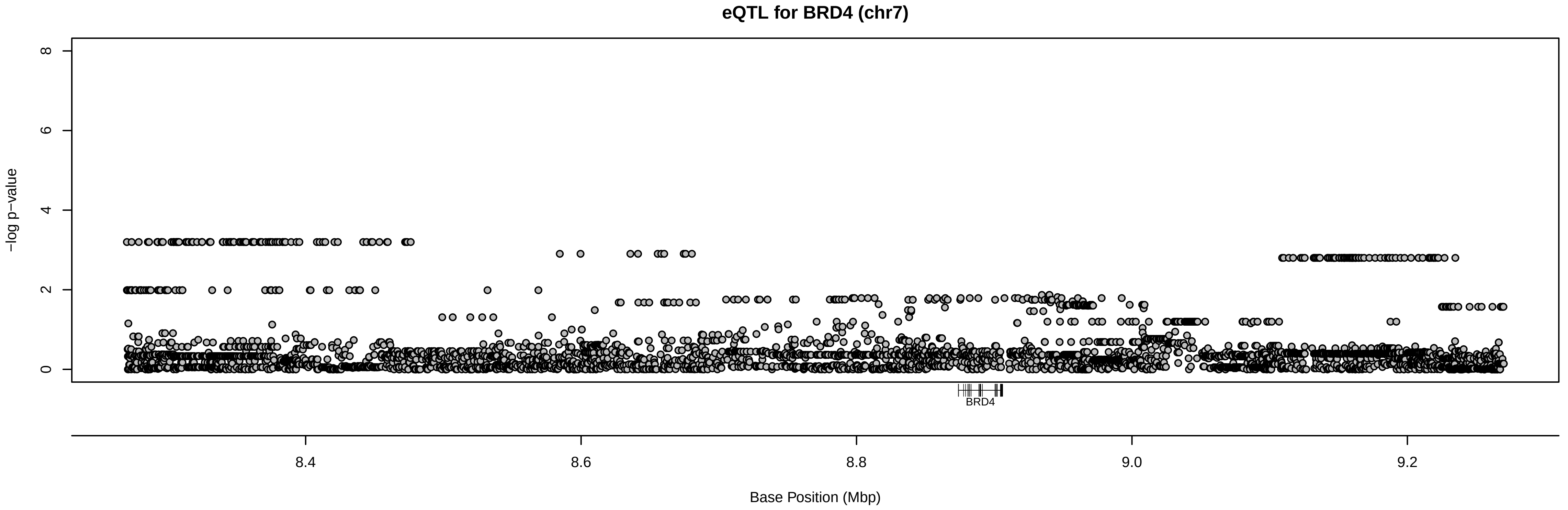
eQTL for BRD4 (chr7)
−log p−value
0
2
4
6
8
BRD4
8.4
8.6
8.8
9.0
9.2
Base Position (Mbp)

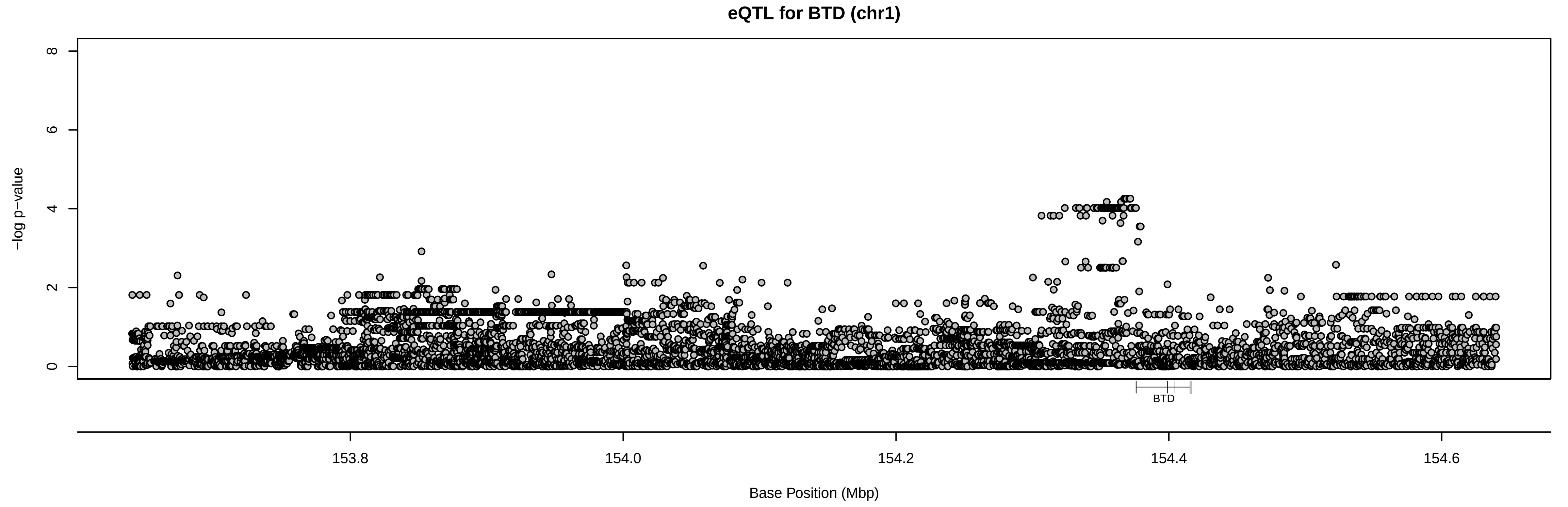

eQTL for BTD (chr1)
−log p−value
0
2
4
6
8
BTD
153.8
154.0
154.2
154.4
154.6
Base Position (Mbp)

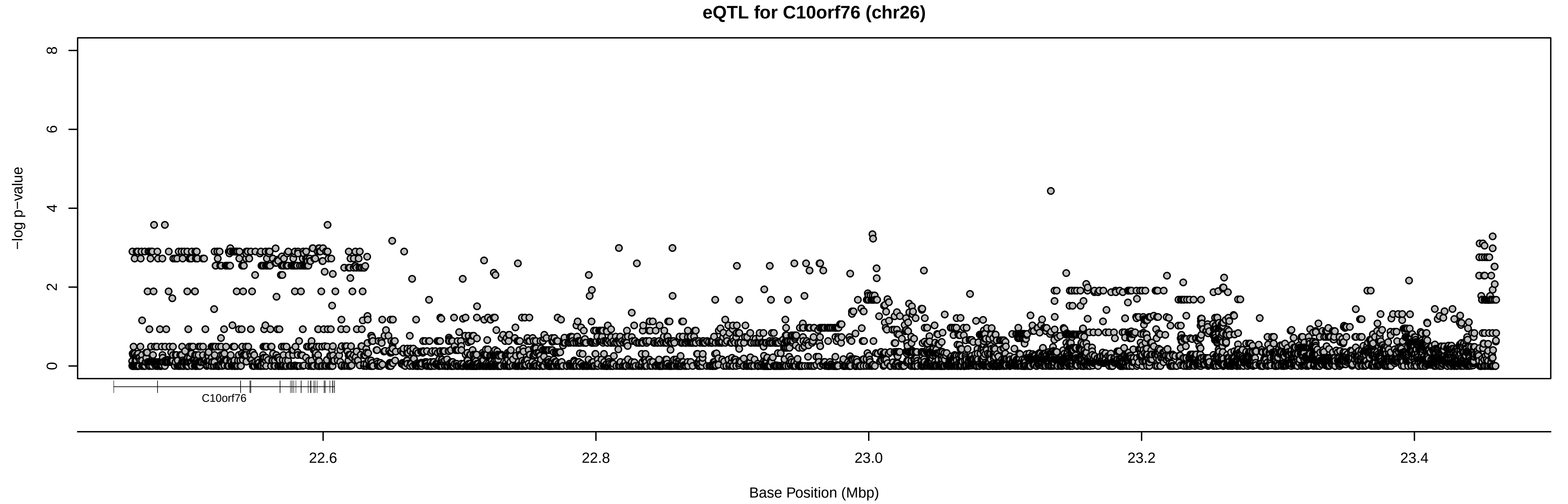

eQTL for C10orf76 (chr26)
−log p−value
0
2
4
6
8
C10orf76
22.6
22.8
23.0
23.2
23.4
Base Position (Mbp)

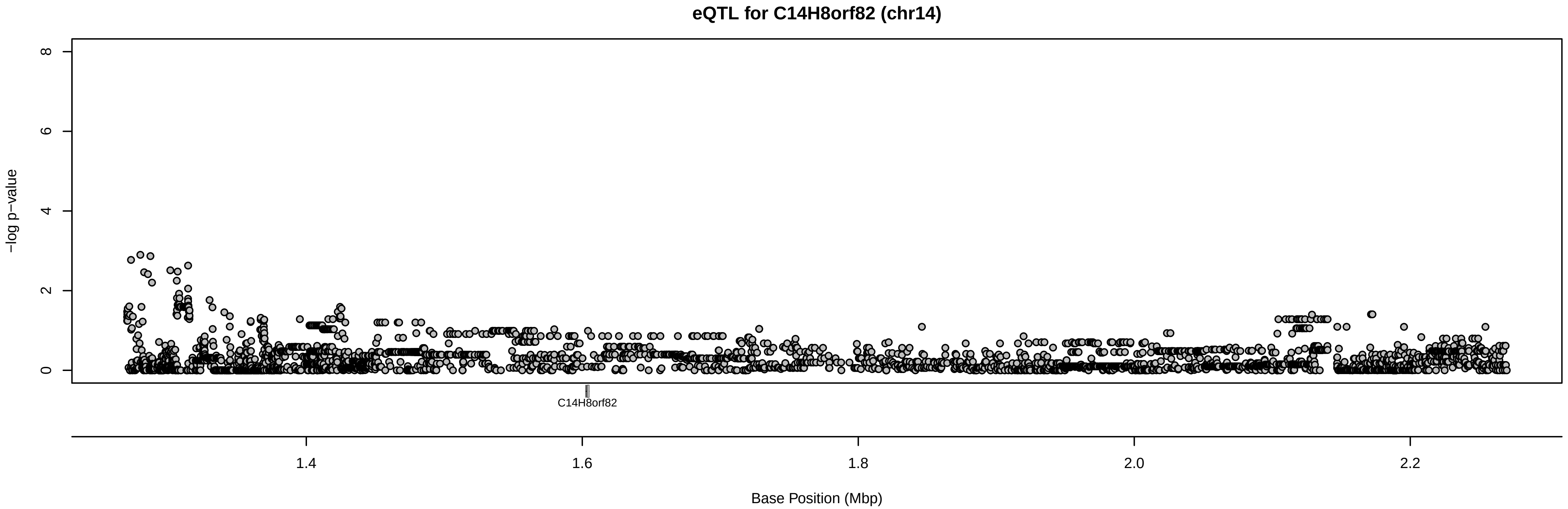
eQTL for C14H8orf82 (chr14)
−log p−value
0
2
4
6
8
C14H8orf82
1.4
1.6
1.8
2.0
2.2
Base Position (Mbp)

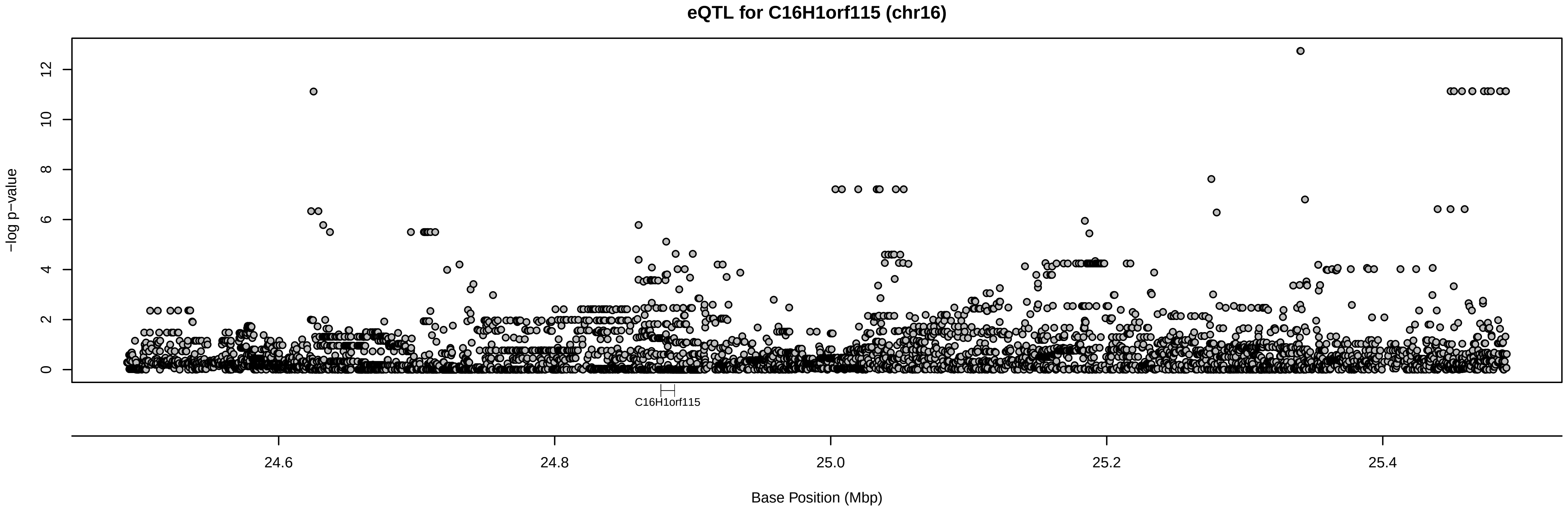
eQTL for C16H1orf115 (chr16)
−log p−value
0
2
4
6
8
10
12
C16H1orf115
24.6
24.8
25.0
25.2
25.4
Base Position (Mbp)

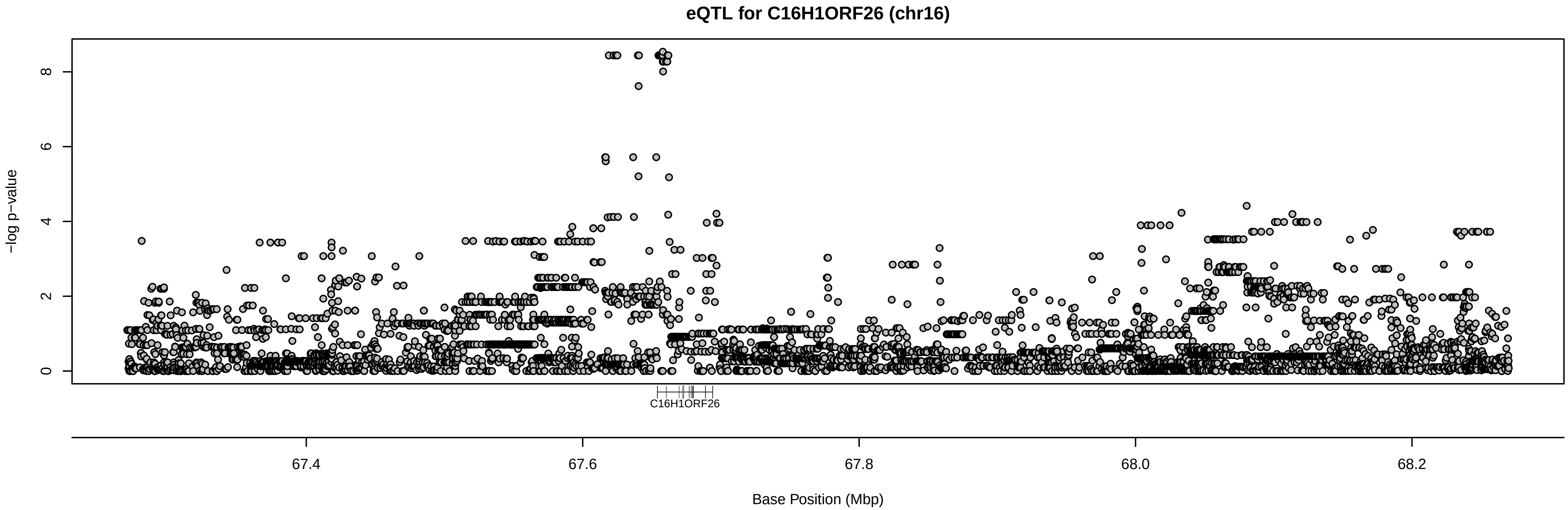

eQTL for C16H1ORF26 (chr16)
−log p−value
0
2
4
6
8
C16H1ORF26
67.4
67.6
67.8
68.0
68.2
Base Position (Mbp)

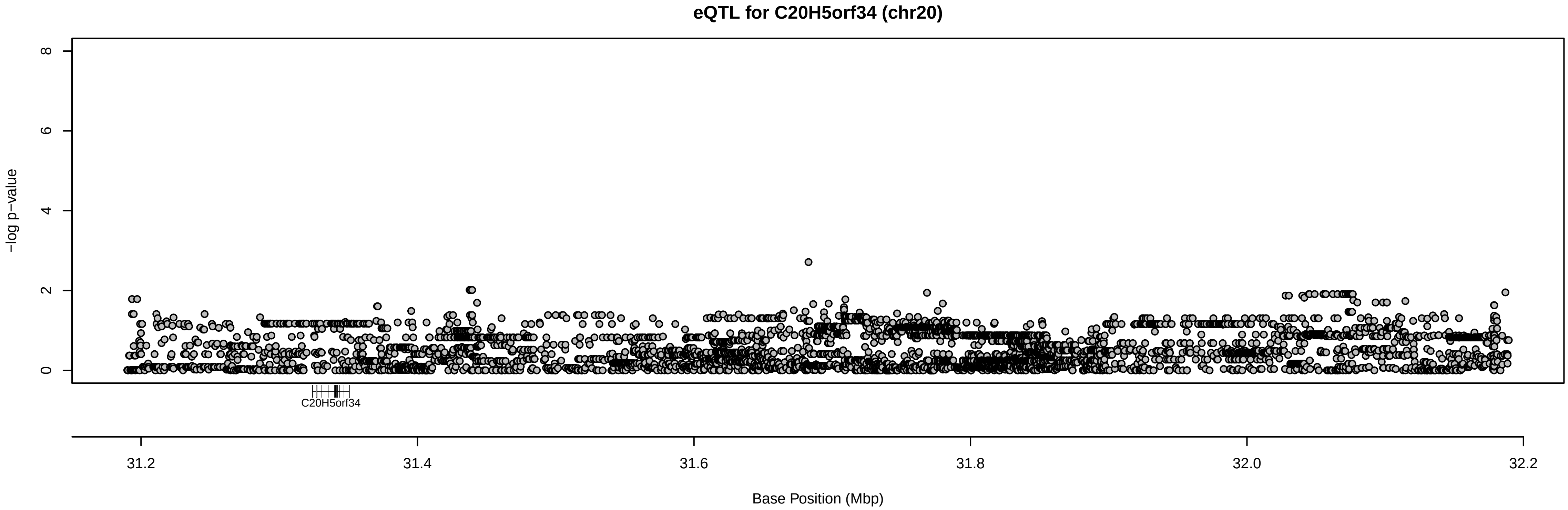

eQTL for C20H5orf34 (chr20)
−log p−value
0
2
4
6
8
C20H5orf34
31.2
31.4
31.6
31.8
32.0
32.2
Base Position (Mbp)

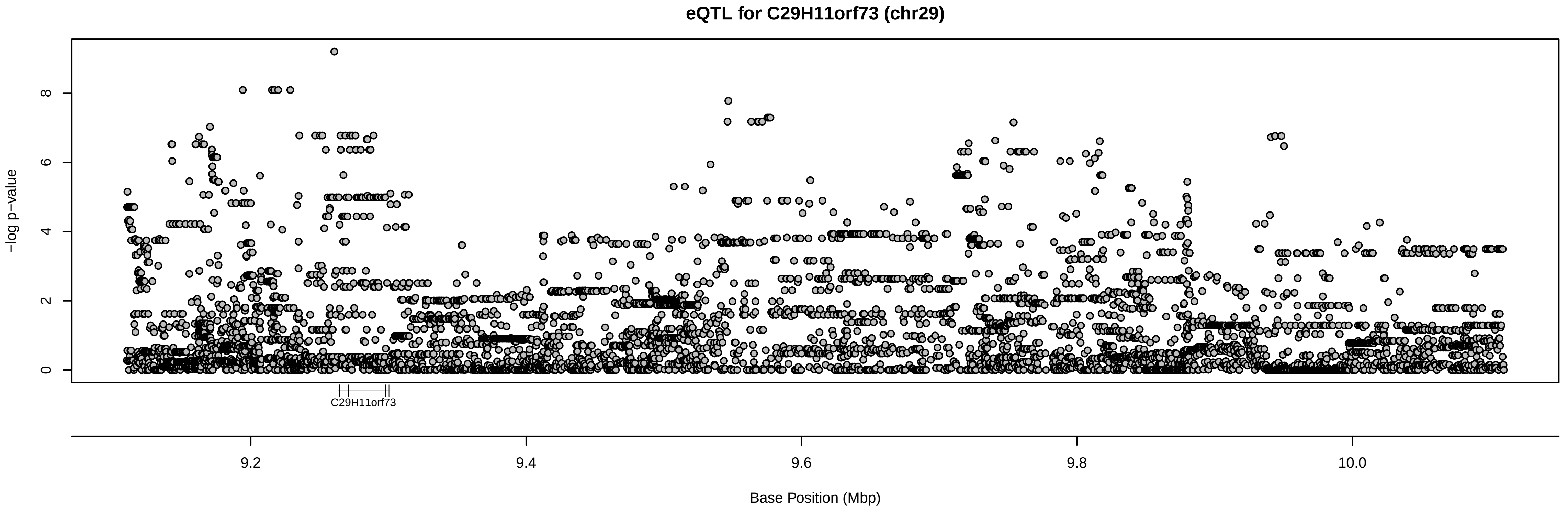
eQTL for C29H11orf73 (chr29)
−log p−value
0
2
4
6
8
C29H11orf73
9.2
9.4
9.6
9.8
10.0
Base Position (Mbp)

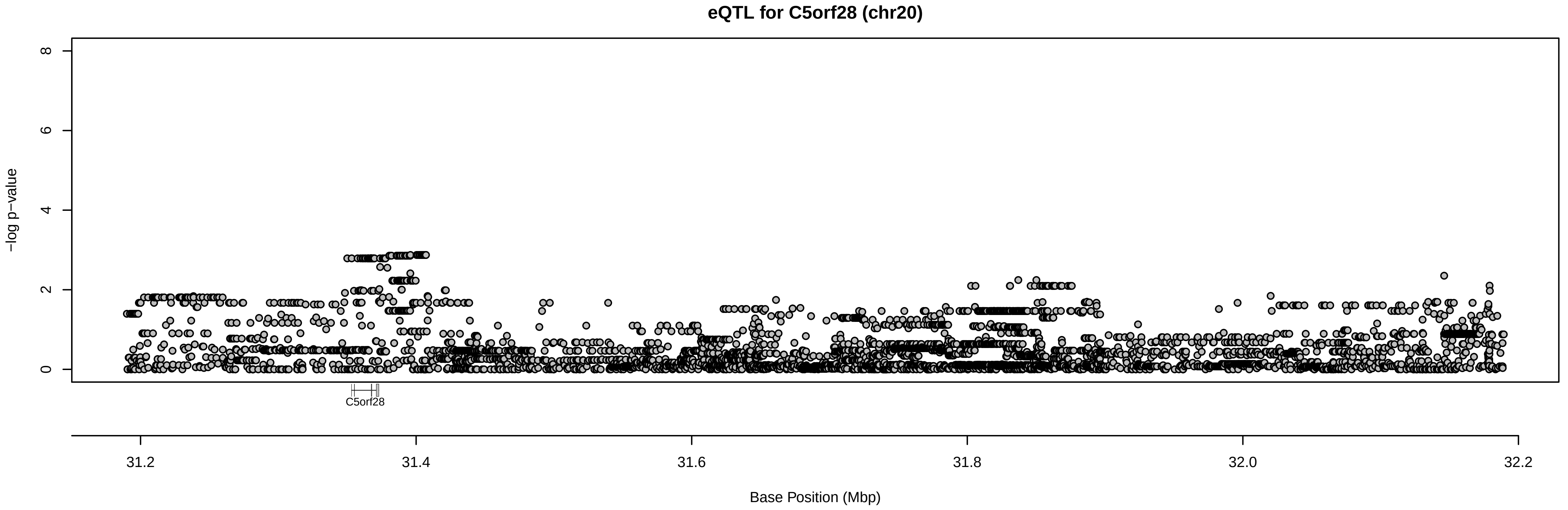
eQTL for C5orf28 (chr20)
−log p−value
0
2
4
6
8
C5orf28
31.2
31.4
31.6
31.8
32.0
32.2
Base Position (Mbp)

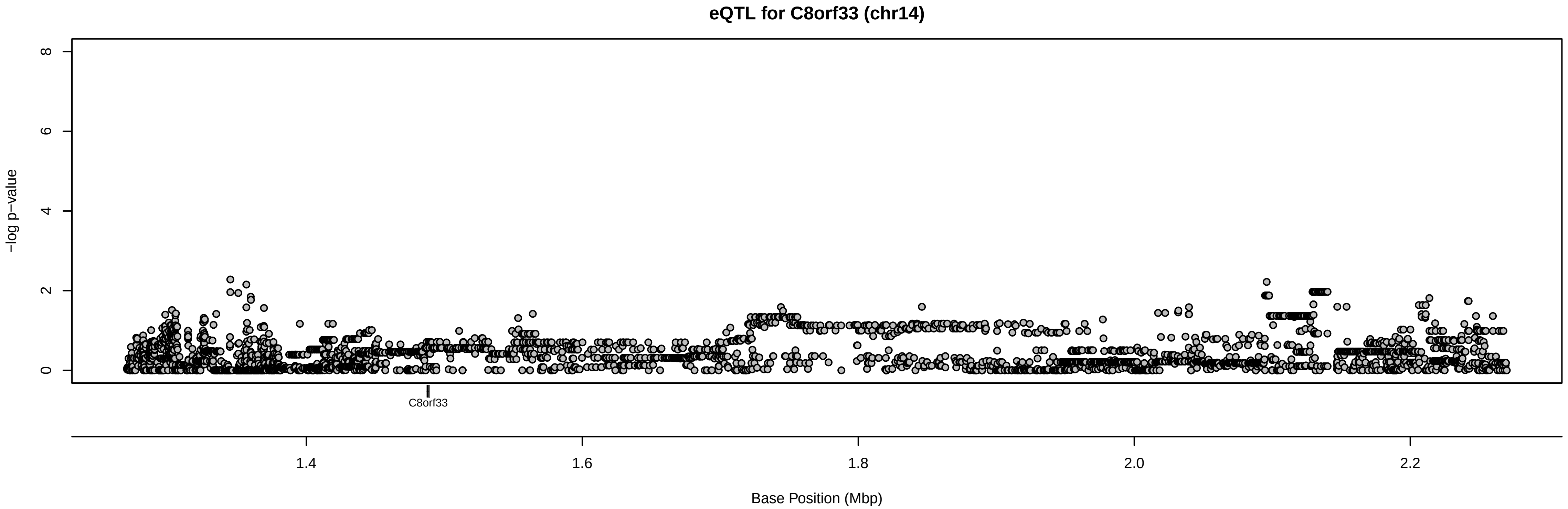
eQTL for C8orf33 (chr14)
−log p−value
0
2
4
6
8
C8orf33
1.4
1.6
1.8
2.0
2.2
Base Position (Mbp)

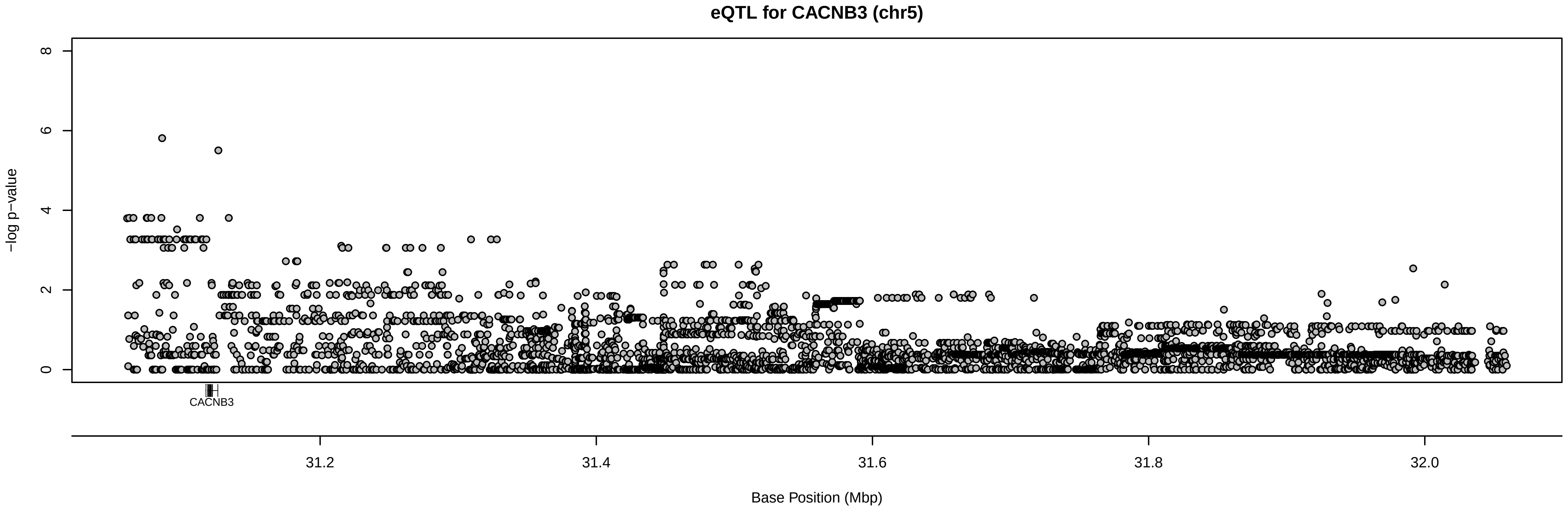
eQTL for CACNB3 (chr5)
−log p−value
0
2
4
6
8
CACNB3
31.2
31.4
31.6
31.8
32.0
Base Position (Mbp)

eQTL for CAMKK2 (chr17)

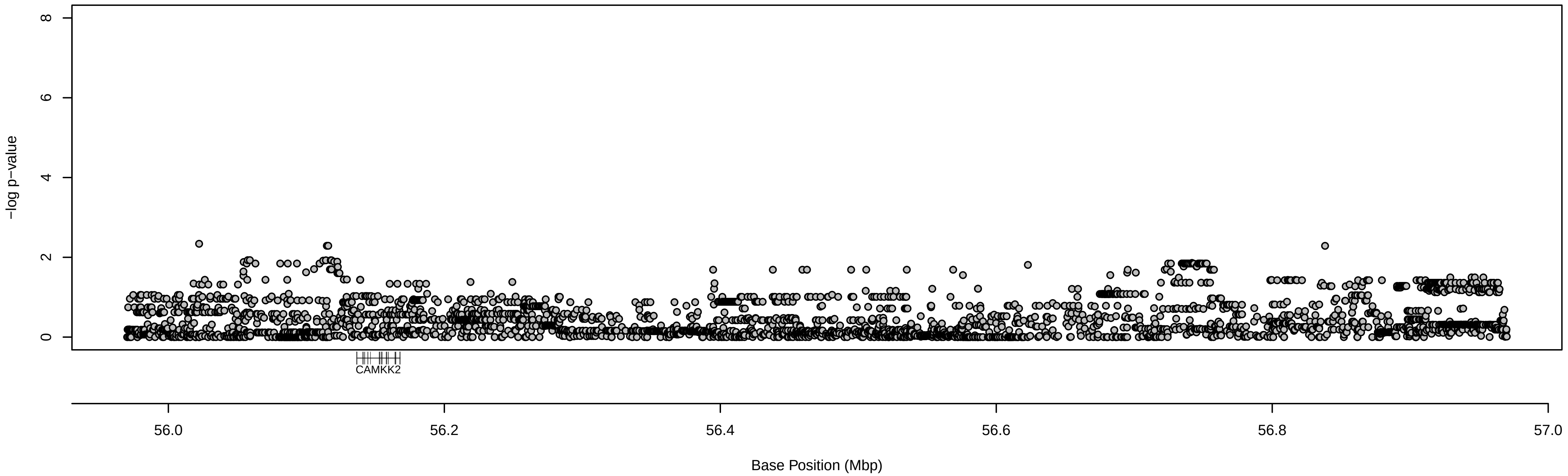

eQTL for CAPN7 (chr1)

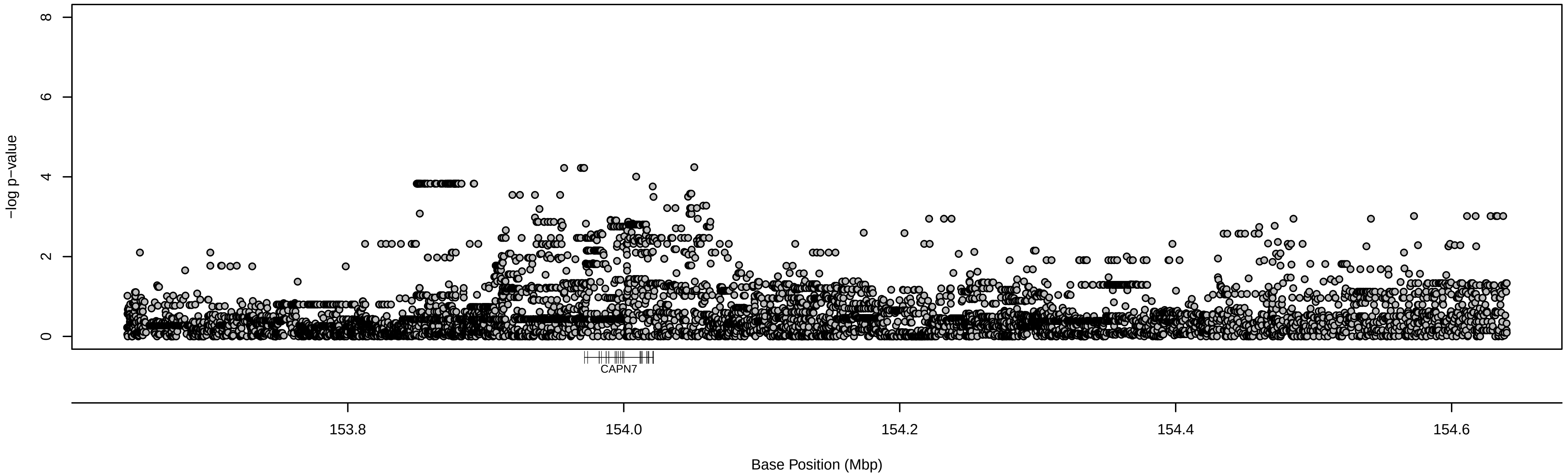

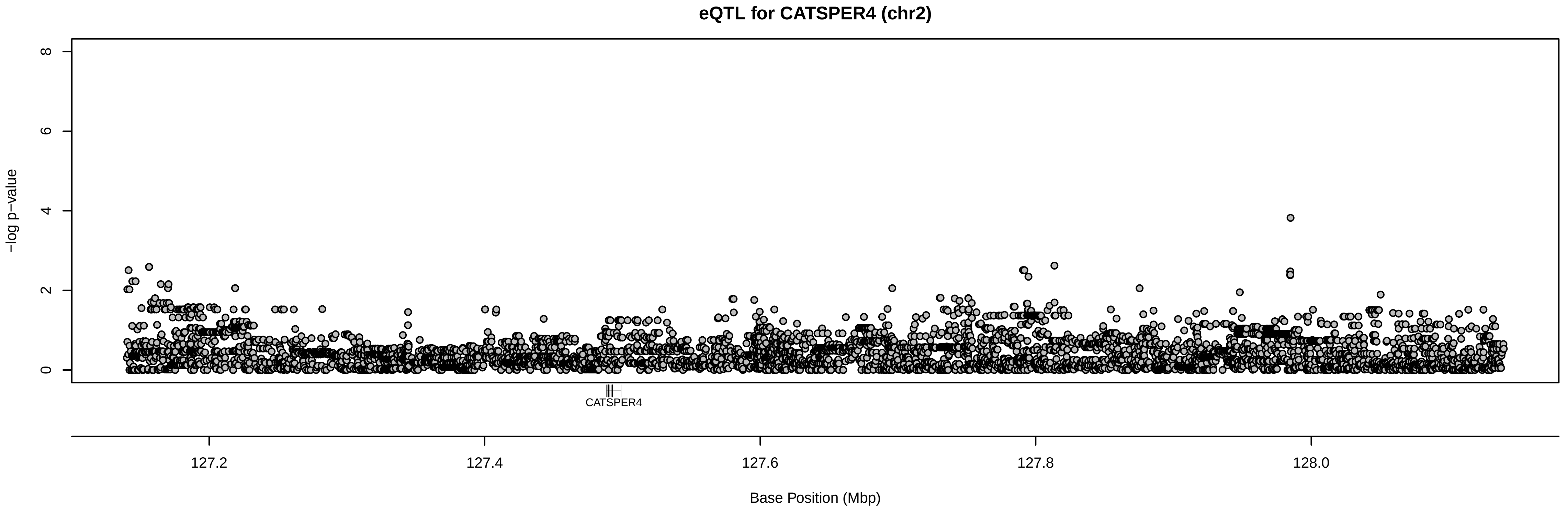
eQTL for CATSPER4 (chr2)
−log p−value
0
2
4
6
8
CATSPER4
127.2
127.4
127.6
127.8
128.0
Base Position (Mbp)

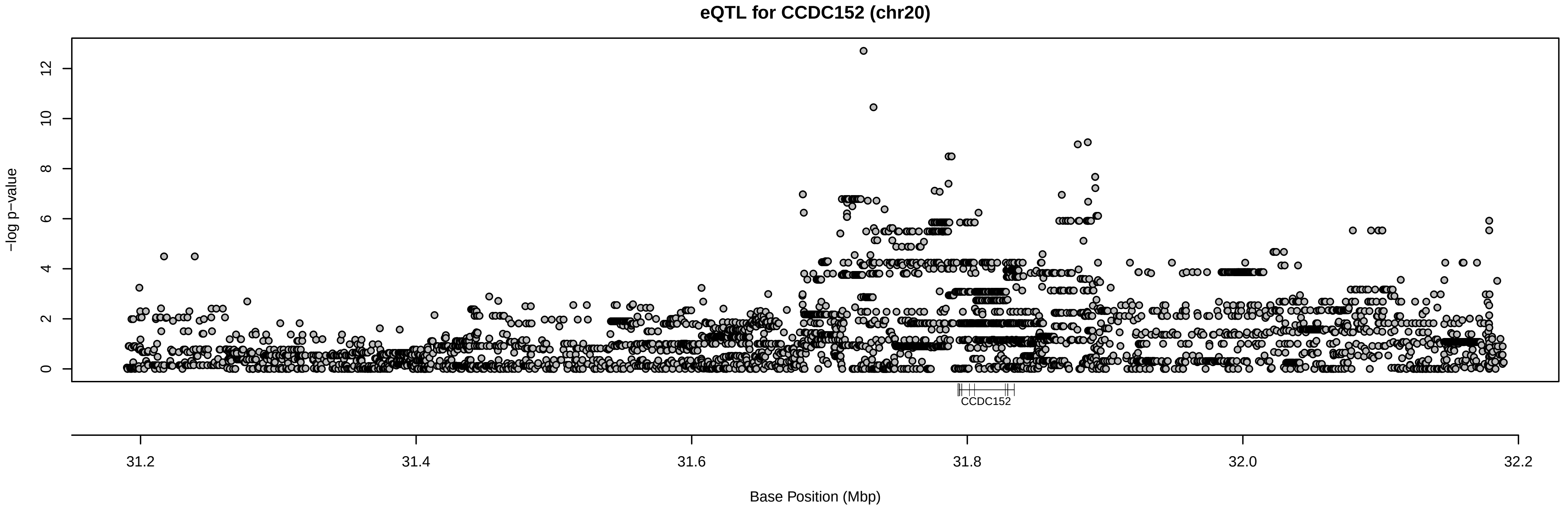
eQTL for CCDC152 (chr20)
−log p−value
0
2
4
6
8
10
12
CCDC152
31.2
31.4
31.6
31.8
32.0
32.2
Base Position (Mbp)

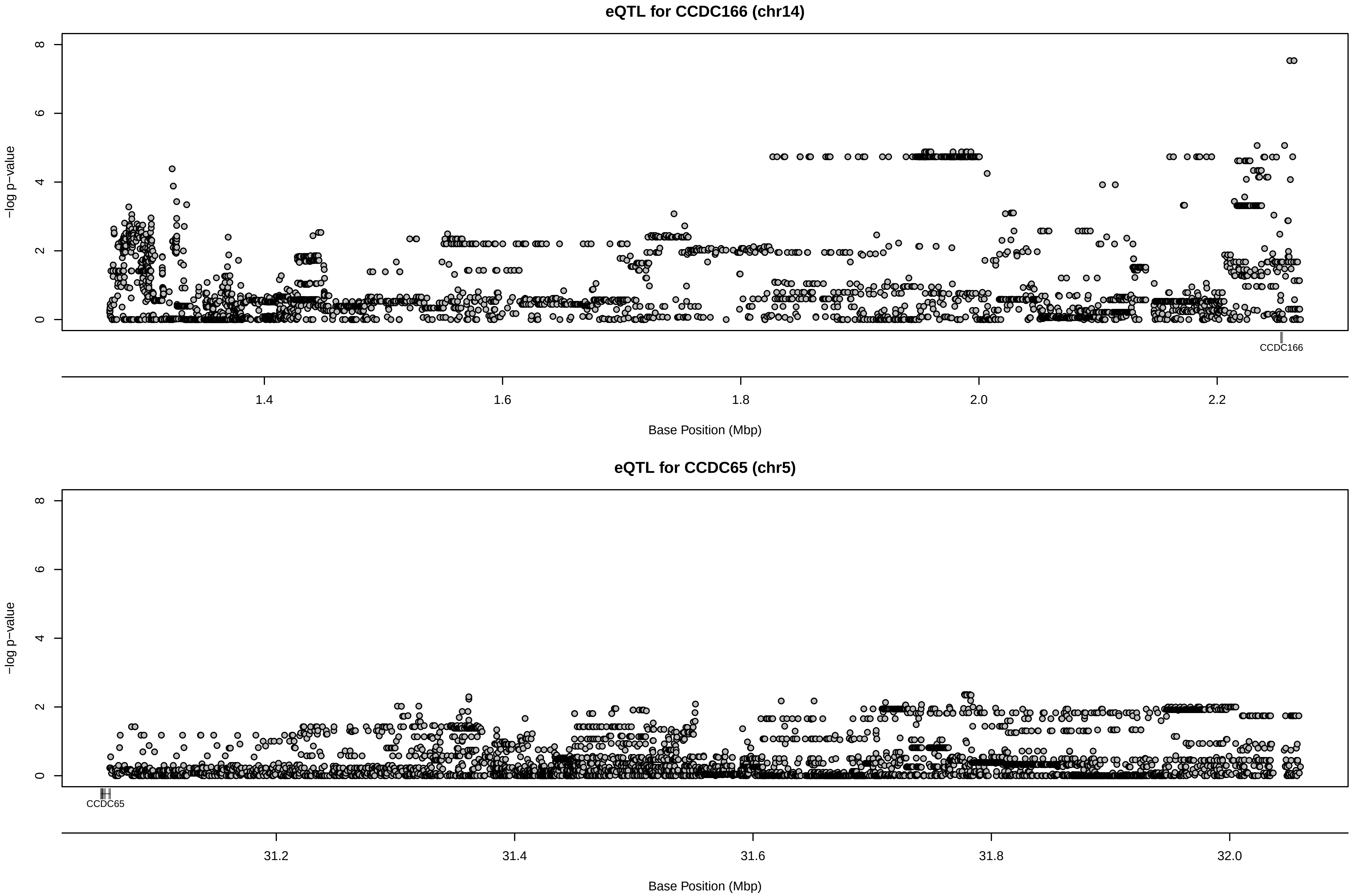

eQTL for CCDC166 (chr14)
−log p−value
0
2
4
6
8
CCDC166
1.4
1.6
1.8
2.0
2.2
Base Position (Mbp)
eQTL for CCDC65 (chr5)
−log p−value
0
2
4
6
8
CCDC65
31.2
31.4
31.6
31.8
32.0
Base Position (Mbp)

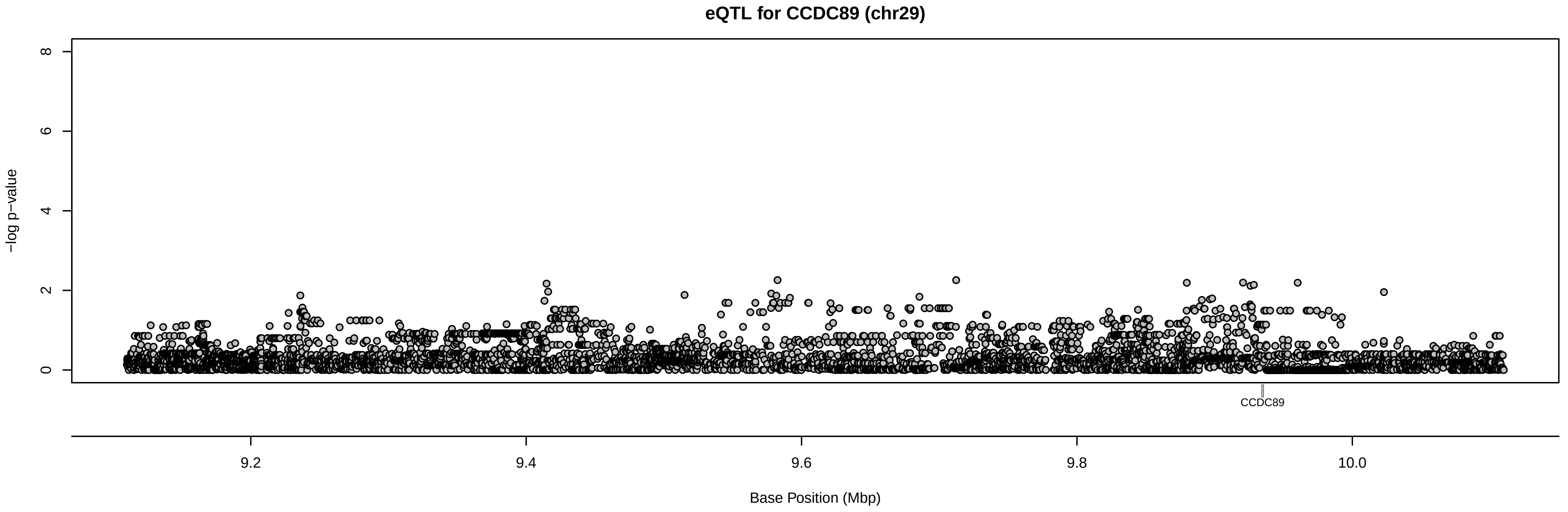
eQTL for CCDC89 (chr29)
−log p−value
0
2
4
6
8
CCDC89
9.2
9.4
9.6
9.8
10.0
Base Position (Mbp)

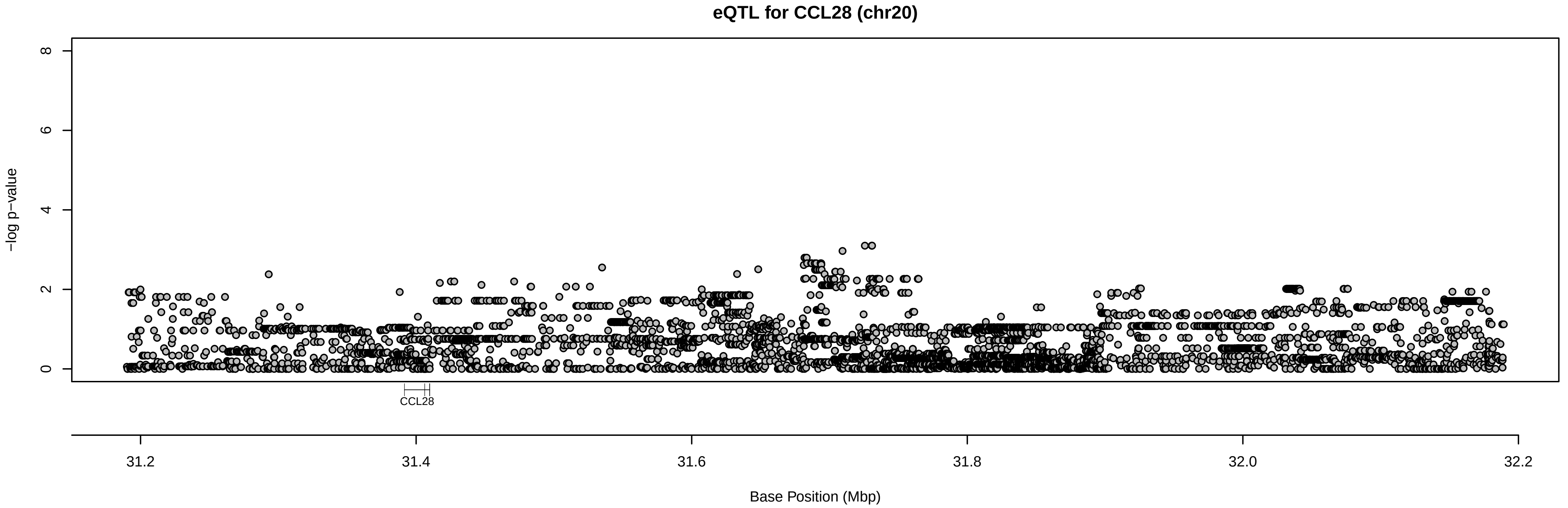
eQTL for CCL28 (chr20)
−log p−value
0
2
4
6
8
CCL28
31.2
31.4
31.6
31.8
32.0
32.2
Base Position (Mbp)

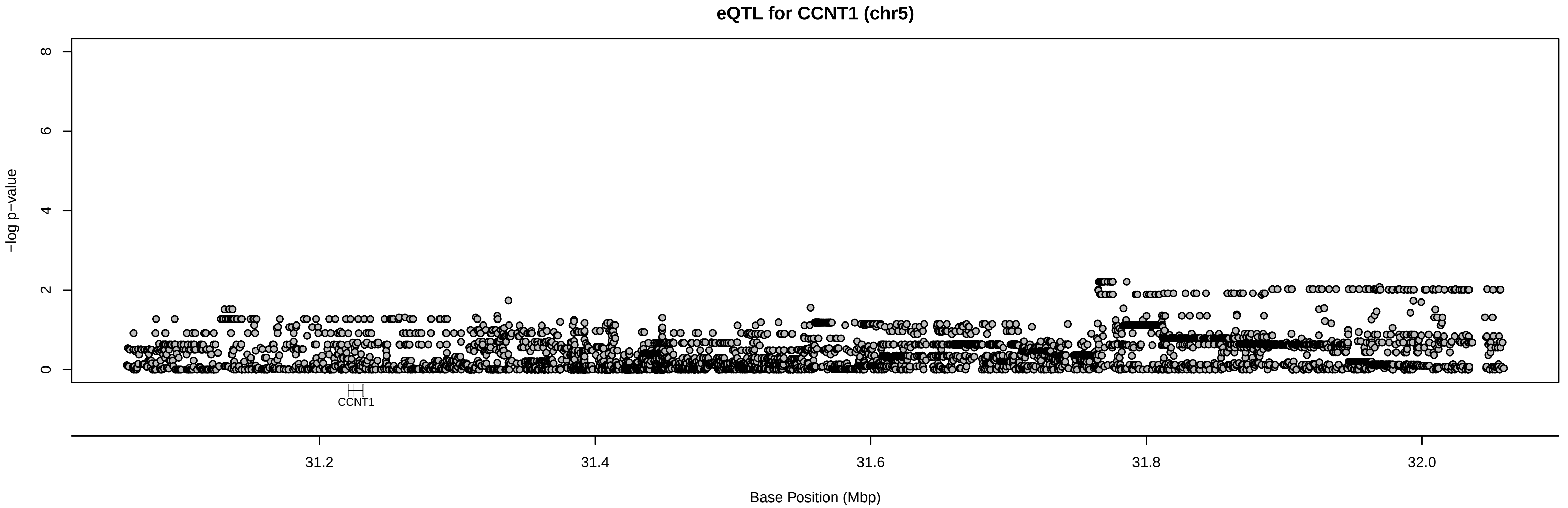

eQTL for CCNT1 (chr5)
−log p−value
0
2
4
6
8
CCNT1
31.2
31.4
31.6
31.8
32.0
Base Position (Mbp)

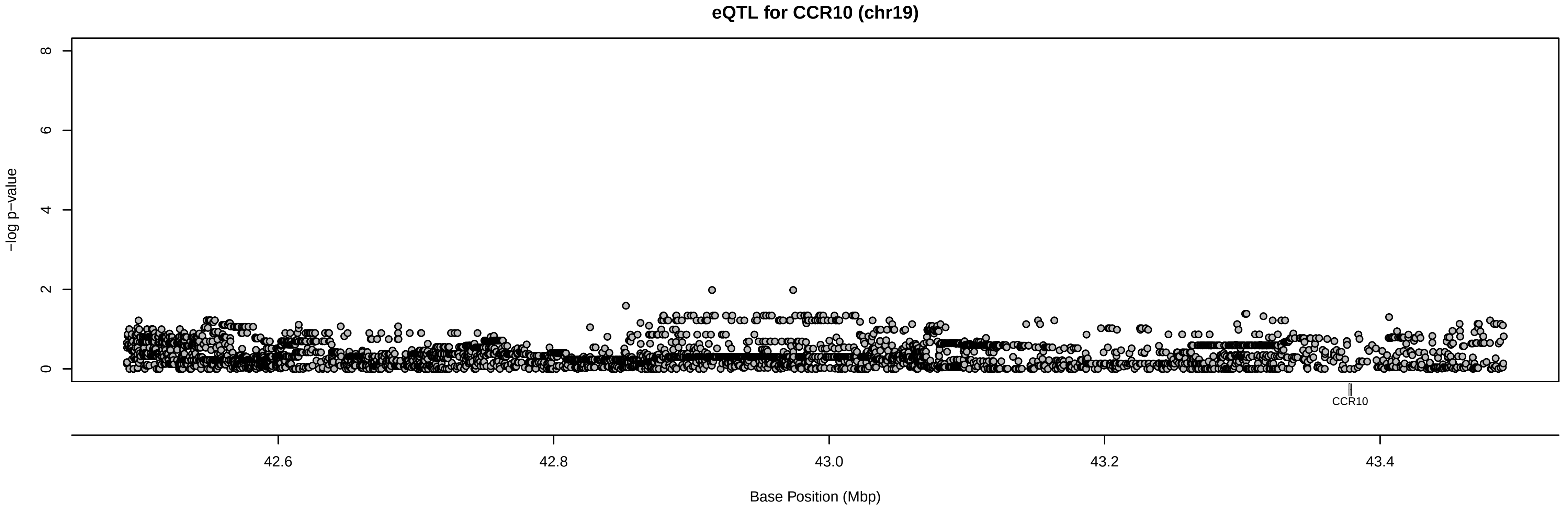

eQTL for CCR10 (chr19)
−log p−value
0
2
4
6
8
CCR10
42.6
42.8
43.0
43.2
43.4
Base Position (Mbp)

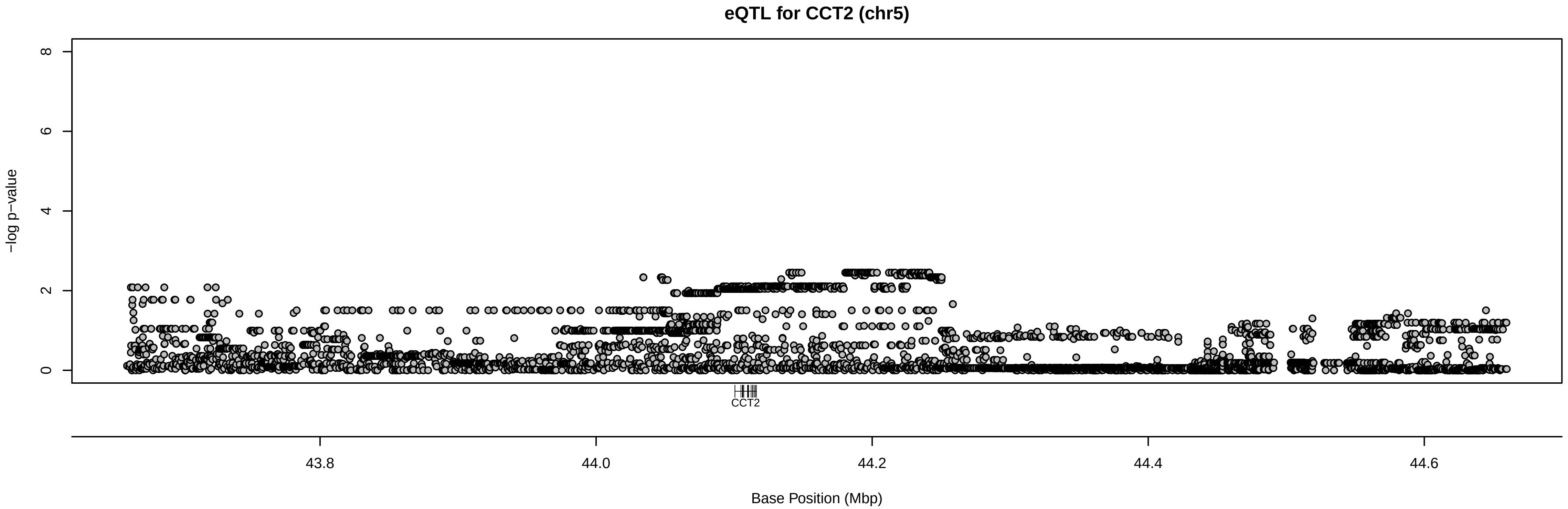
eQTL for CCT2 (chr5)
−log p−value
CCT2
Base Position (Mbp)
43.8
44.0
44.2
44.4
44.6
0
2
4
6
8

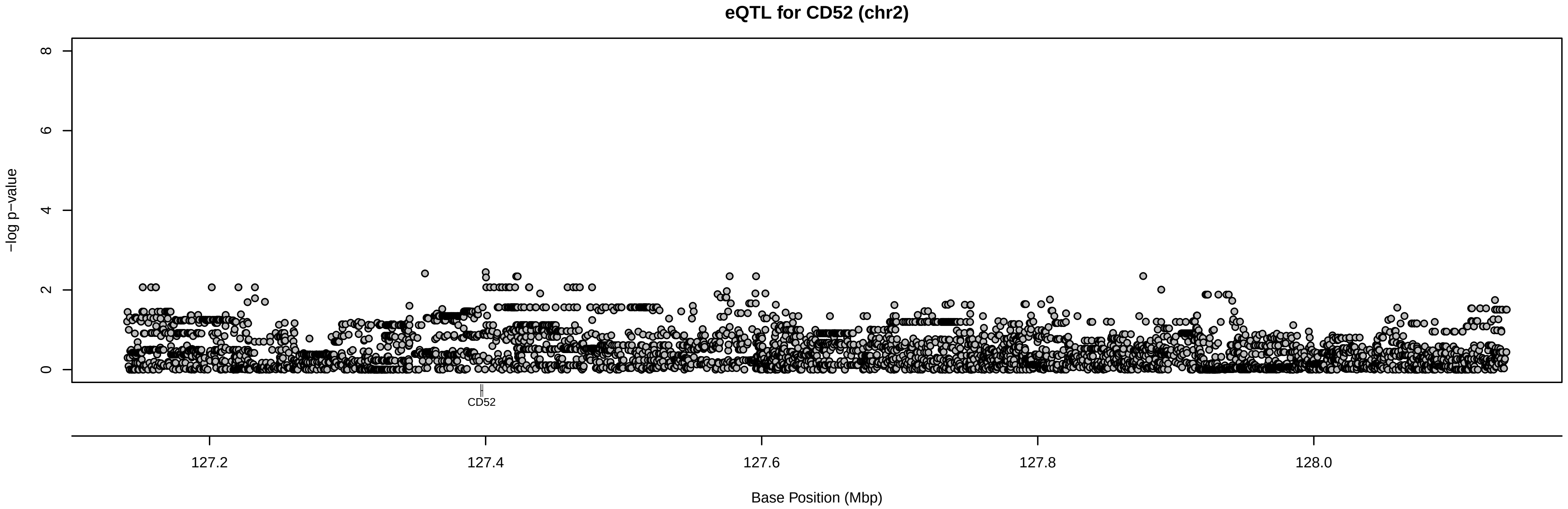
eQTL for CD52 (chr2)
−log p−value
CD52
Base Position (Mbp)
127.2
127.4
127.6
127.8
128.0
0
2
4
6
8

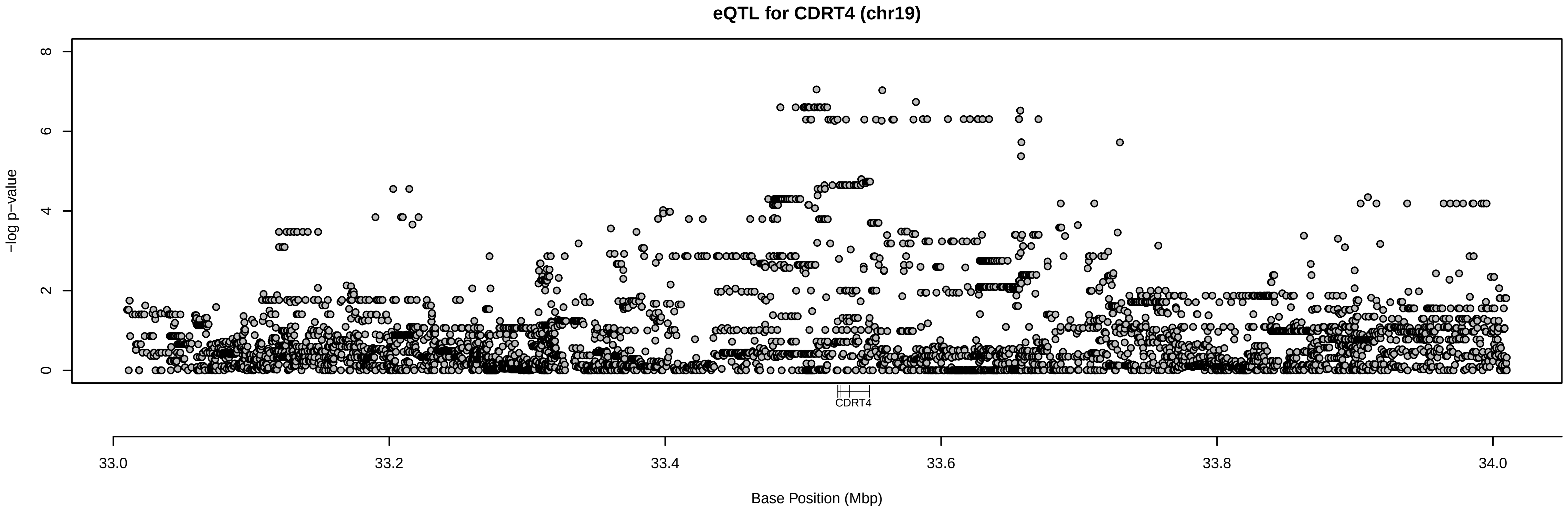

eQTL for CDRT4 (chr19)
−log p−value
0
2
4
6
8
CDRT4
33.0
33.2
33.4
33.6
33.8
34.0
Base Position (Mbp)

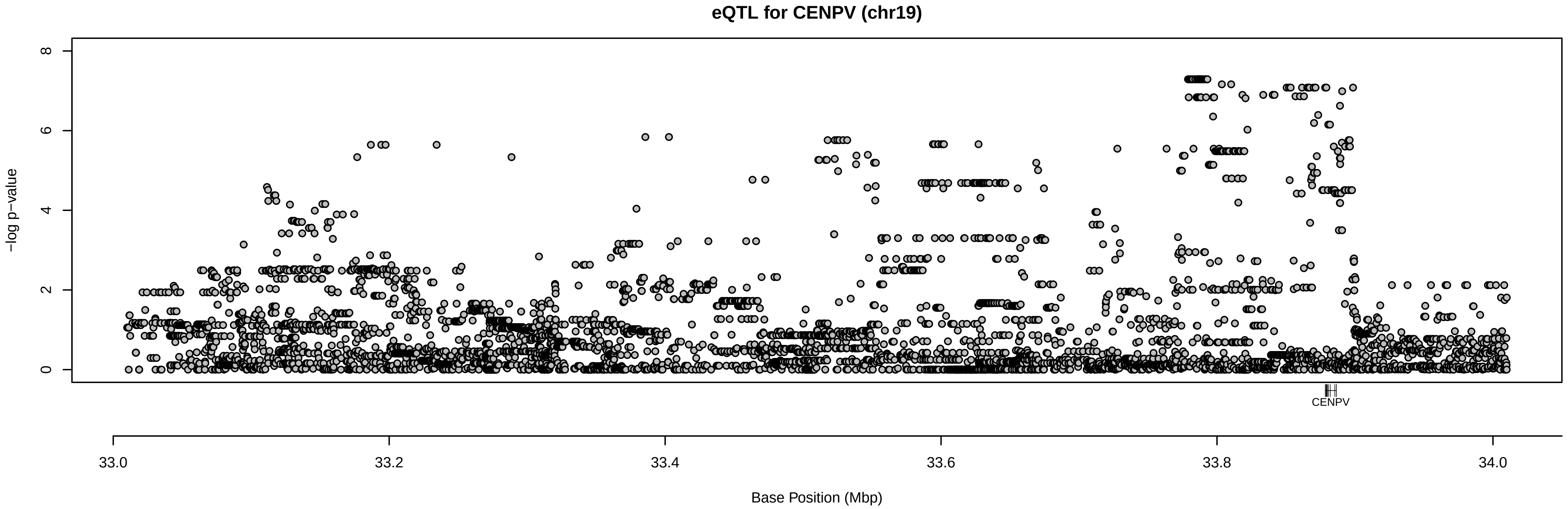

eQTL for CENPV (chr19)
−log p−value
0
2
4
6
8
CENPV
33.0
33.2
33.4
33.6
33.8
34.0
Base Position (Mbp)

eQTL for CEP164 (chr15)

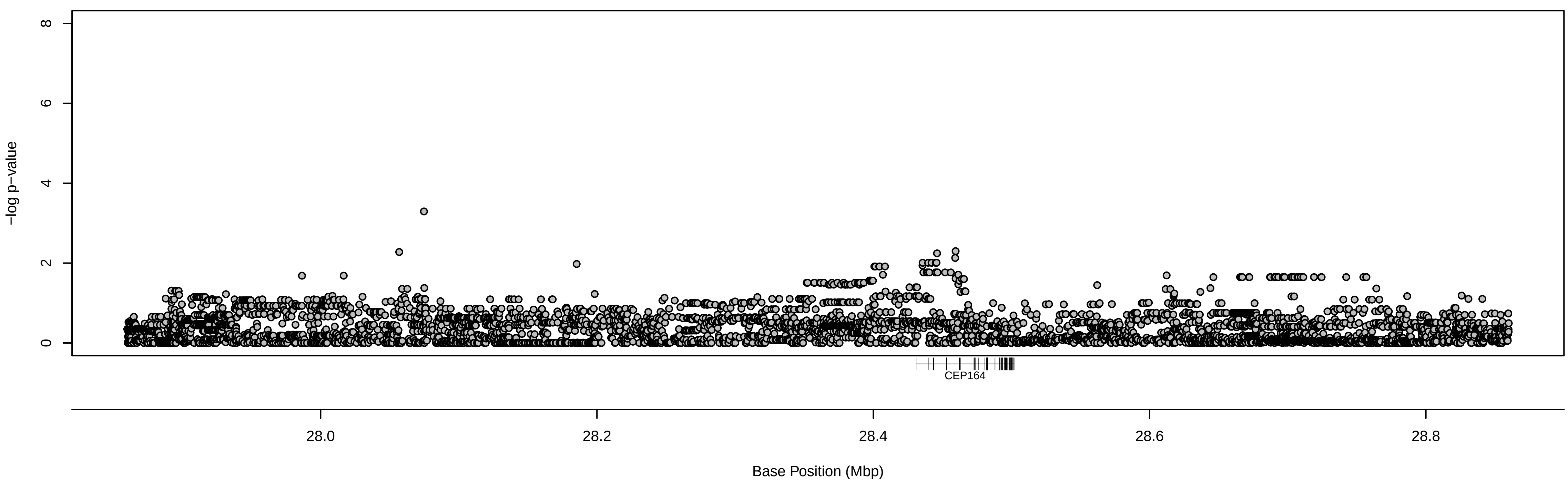

eQTL for CEP68 (chr11)

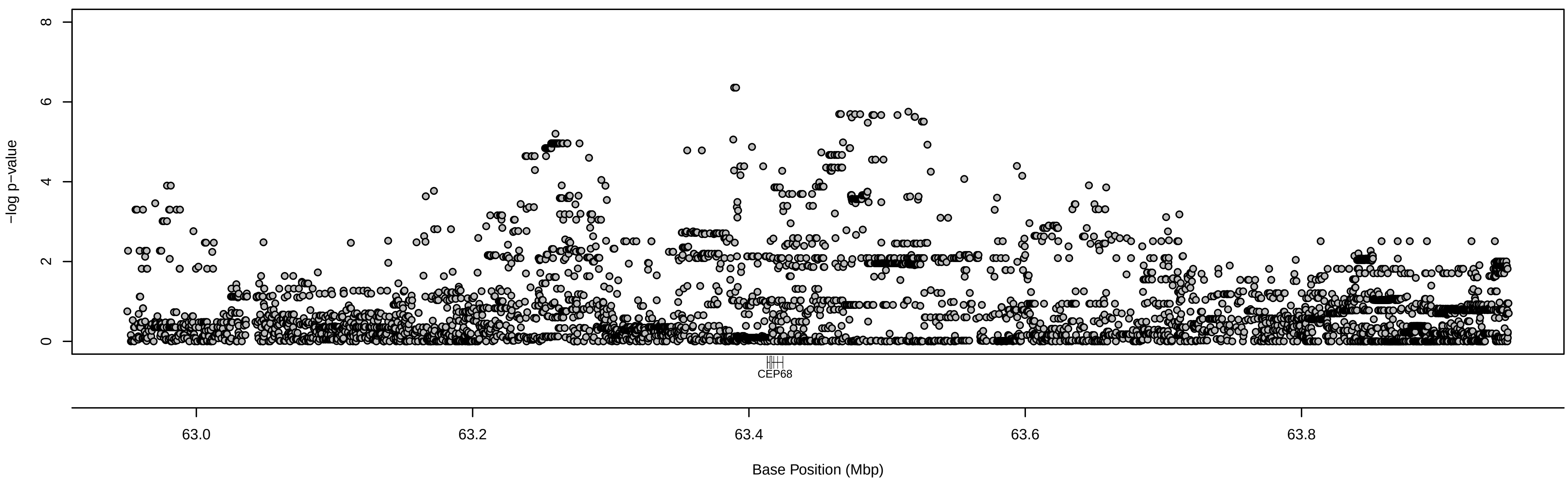

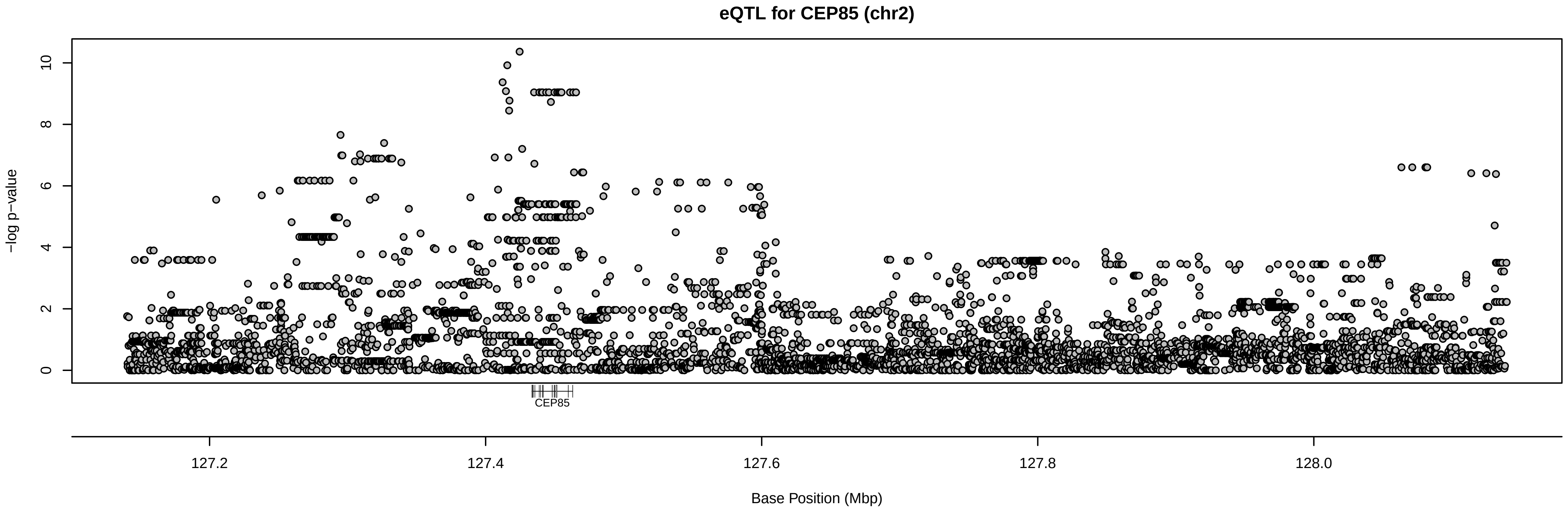
eQTL for CEP85 (chr2)
−log p−value
0
2
4
6
8
10
CEP85
127.2
127.4
127.6
127.8
128.0
Base Position (Mbp)

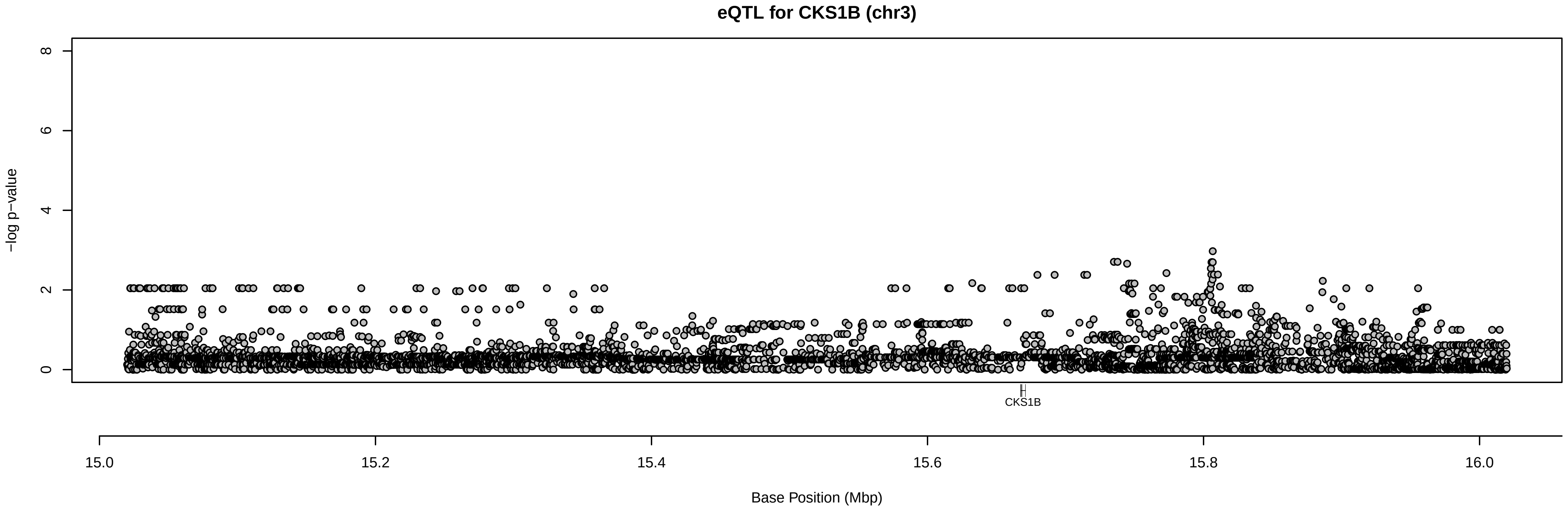
eQTL for CKS1B (chr3)
−log p−value
0
2
4
6
8
CKS1B
15.0
15.2
15.4
15.6
15.8
16.0
Base Position (Mbp)

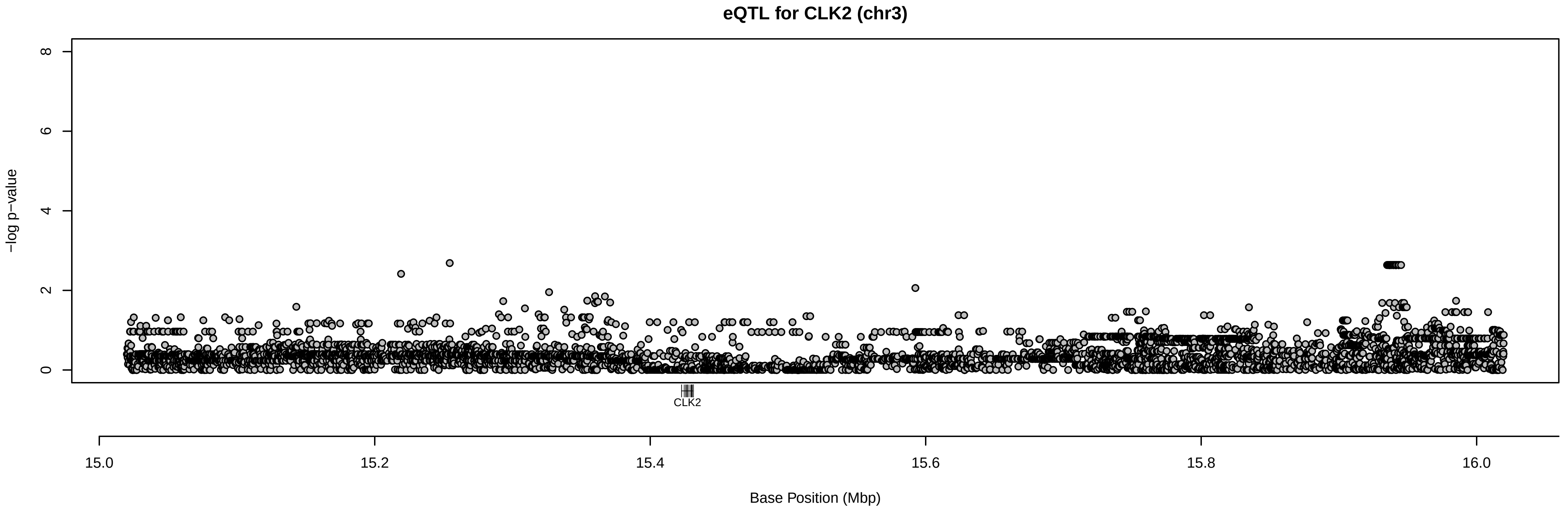
eQTL for CLK2 (chr3)
−log p−value
0
2
4
6
8
CLK2
15.0
15.2
15.4
15.6
15.8
16.0
Base Position (Mbp)

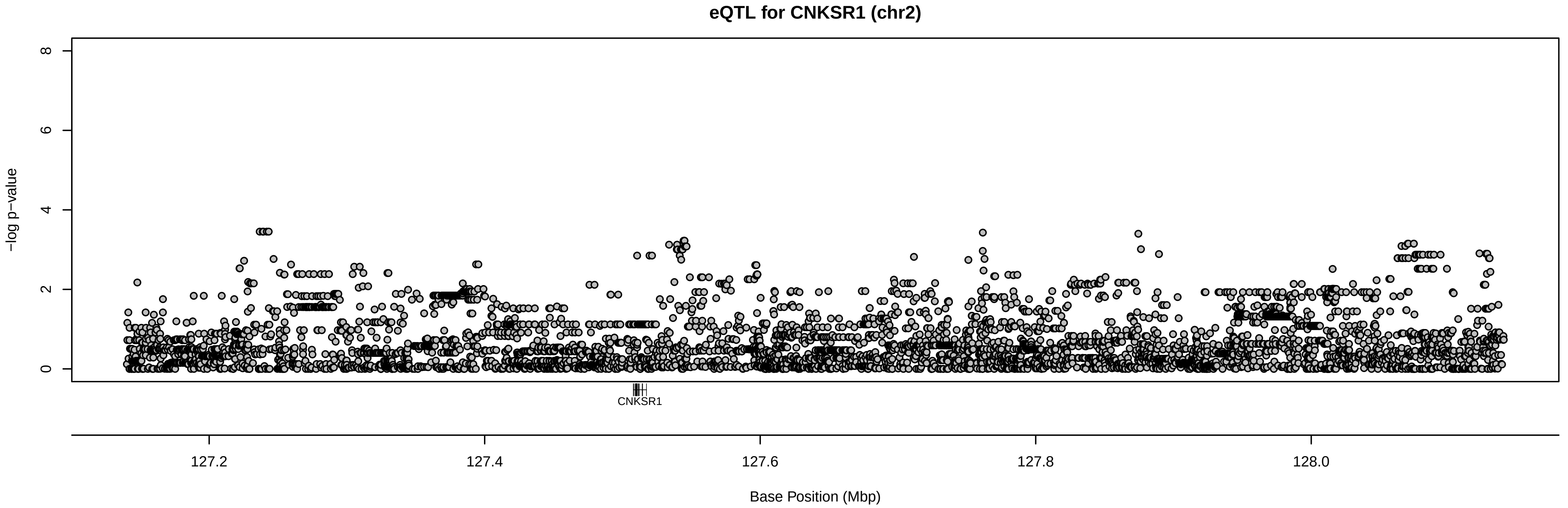
eQTL for CNKSR1 (chr2)
−log p−value
0
2
4
6
8
CNKSR1
127.2
127.4
127.6
127.8
128.0
Base Position (Mbp)

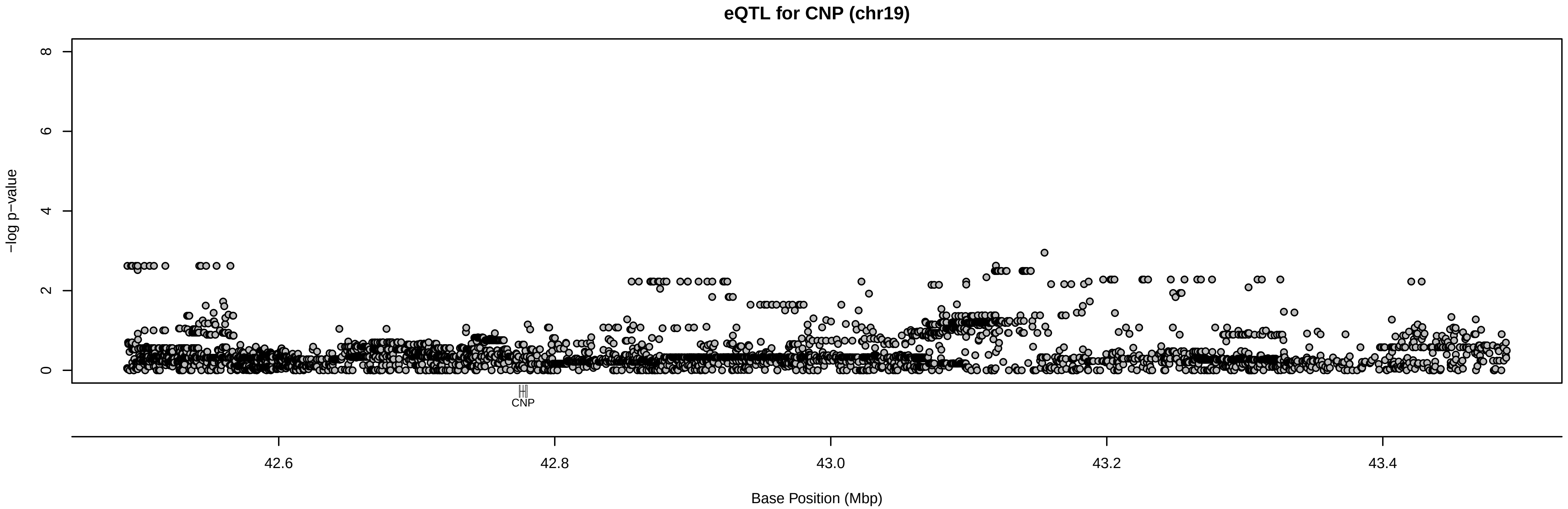

eQTL for CNP (chr19)
−log p−value
0
2
4
6
8
CNP
42.6
42.8
43.0
43.2
43.4
Base Position (Mbp)

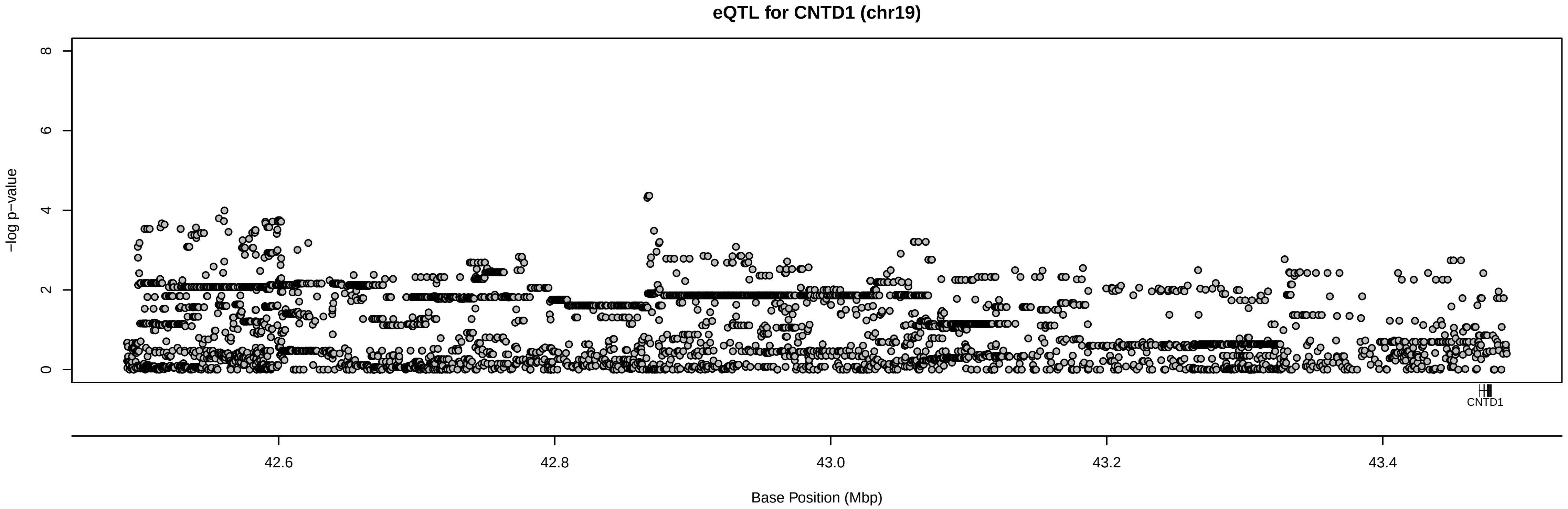

eQTL for CNTD1 (chr19)
−log p−value
0
2
4
6
8
CNTD1
42.6
42.8
43.0
43.2
43.4
Base Position (Mbp)

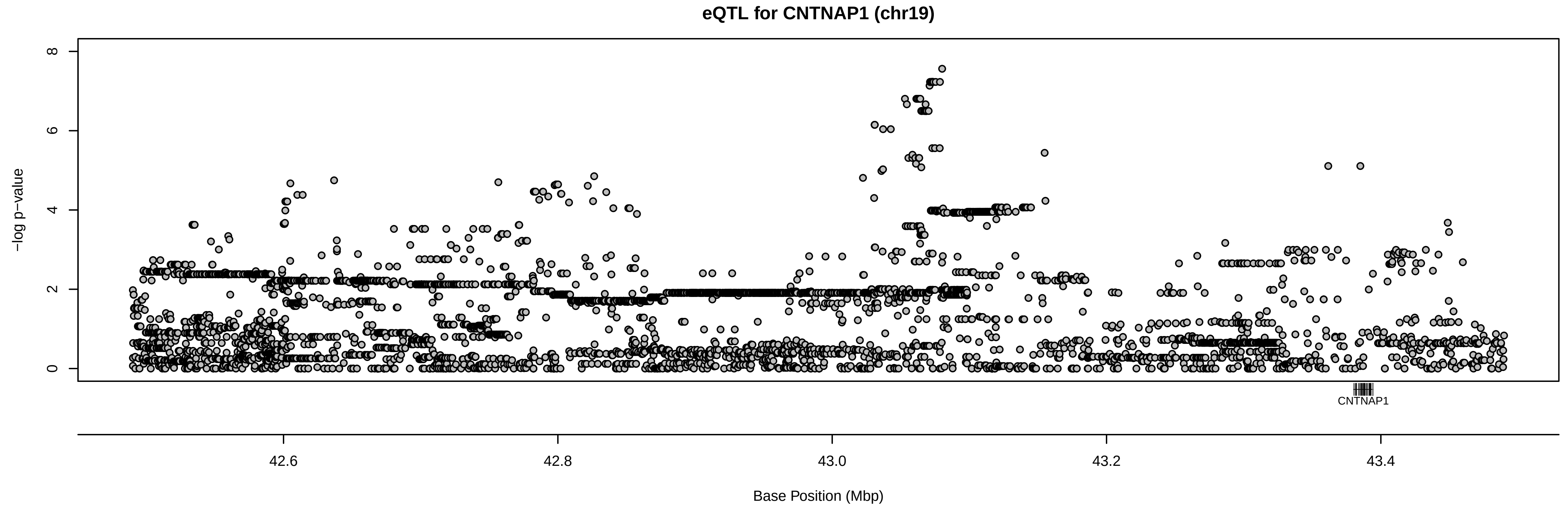
eQTL for CNTNAP1 (chr19)
−log p−value
0
2
4
6
8
CNTNAP1
42.6
42.8
43.0
43.2
43.4
Base Position (Mbp)

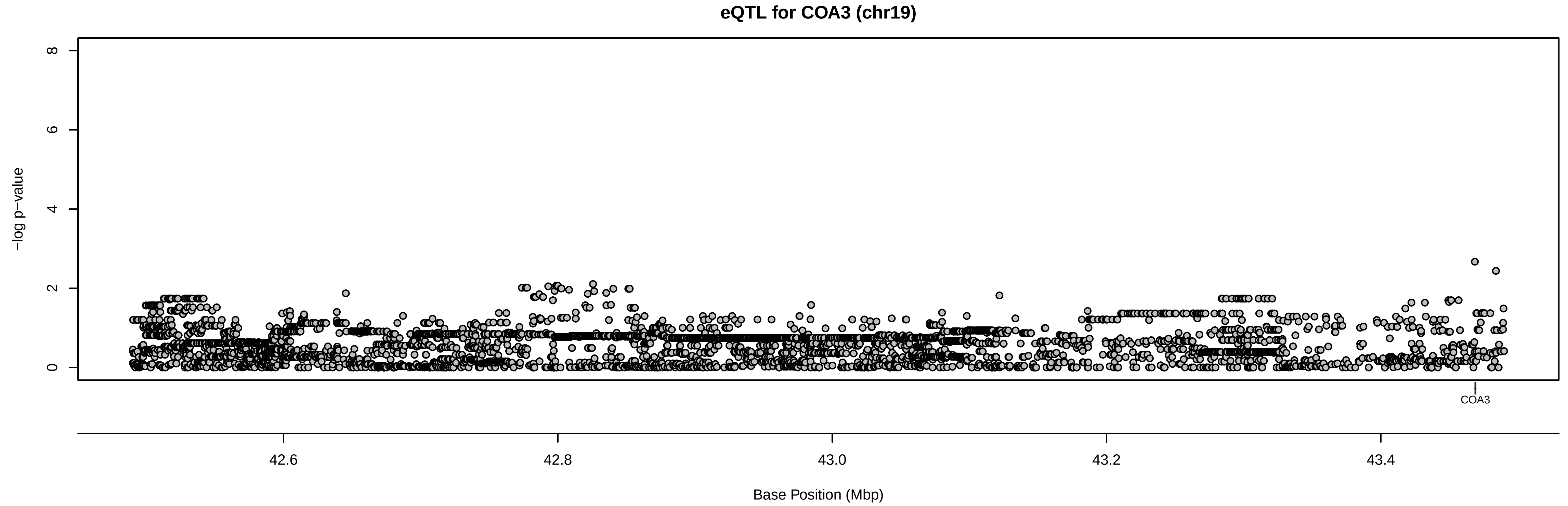
eQTL for COA3 (chr19)
−log p−value
0
2
4
6
8
COA3
42.6
42.8
43.0
43.2
43.4
Base Position (Mbp)

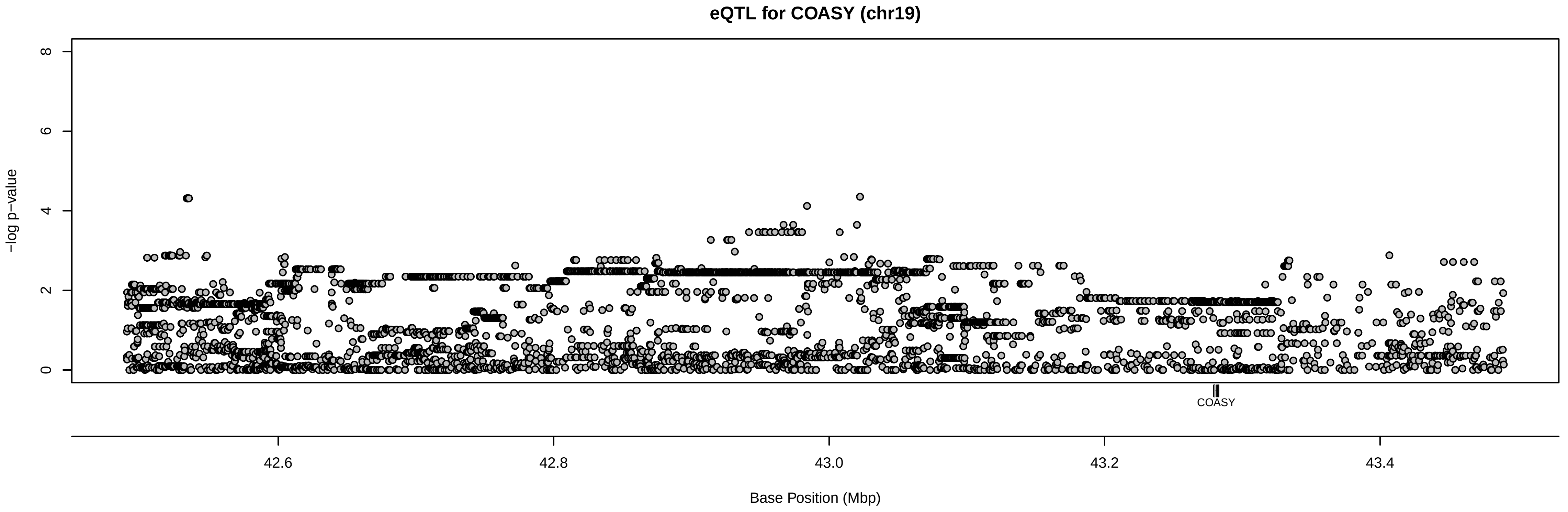
eQTL for COASY (chr19)
−log p−value
0
2
4
6
8
COASY
42.6
42.8
43.0
43.2
43.4
Base Position (Mbp)

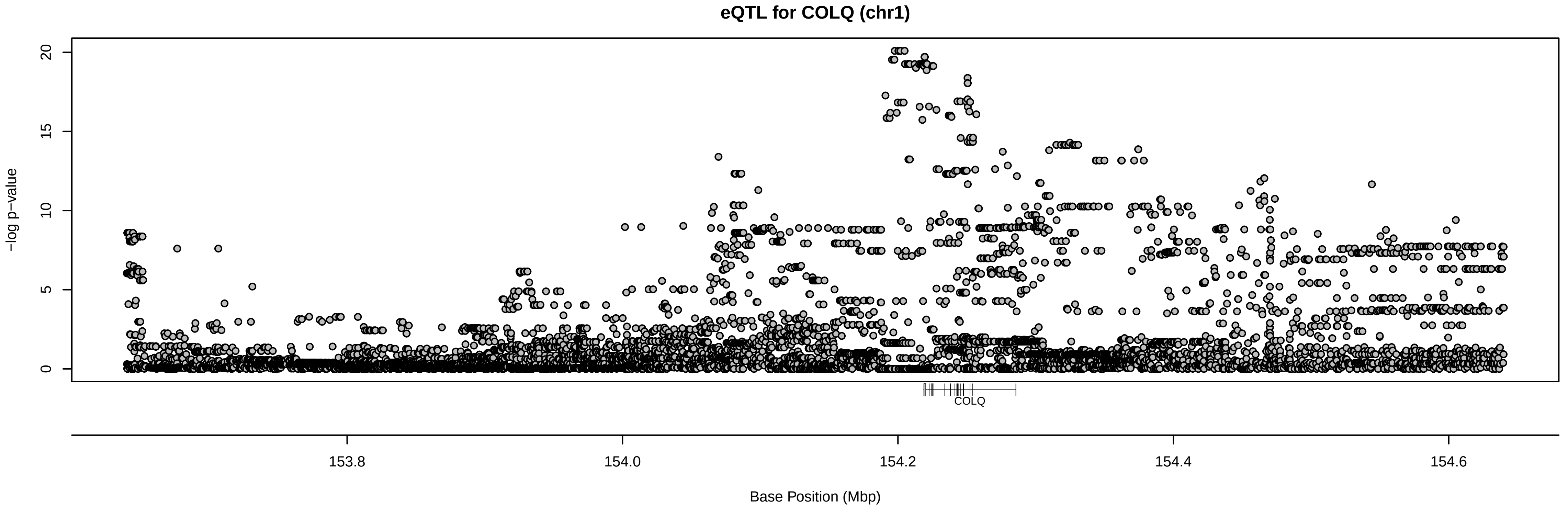
eQTL for COLQ (chr1)
−log p−value
0
5
10
15
20
COLQ
153.8
154.0
154.2
154.4
154.6
Base Position (Mbp)

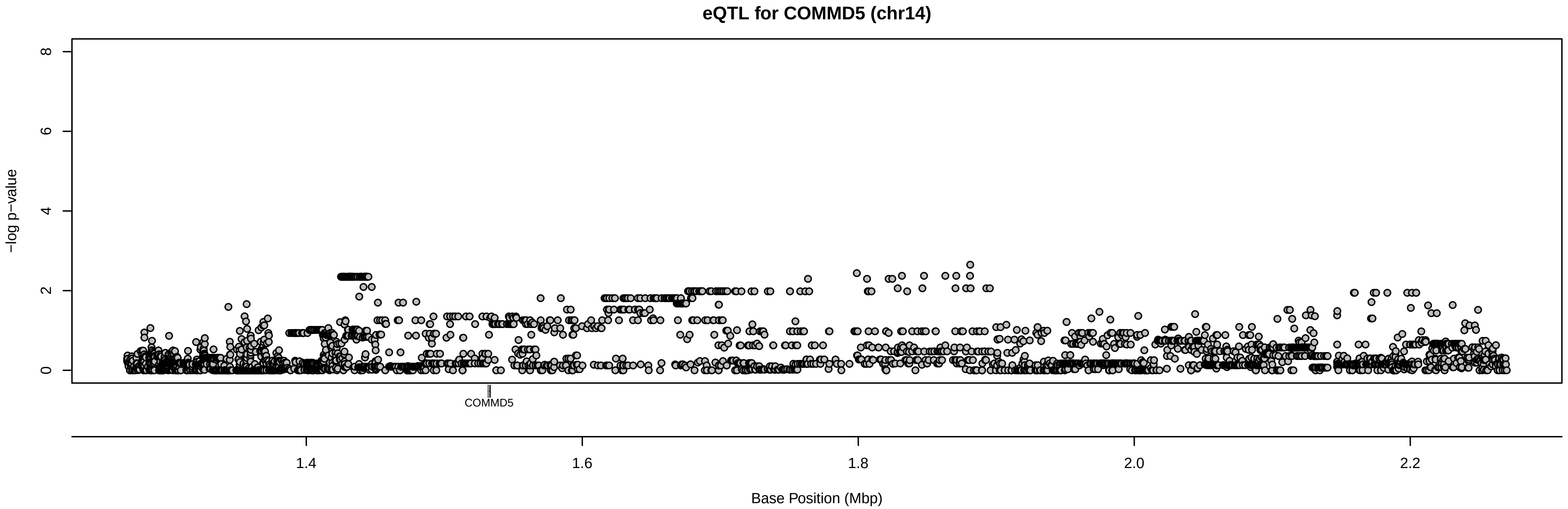

eQTL for COMMD5 (chr14)
−log p−value
0
2
4
6
8
COMMD5
1.4
1.6
1.8
2.0
2.2
Base Position (Mbp)

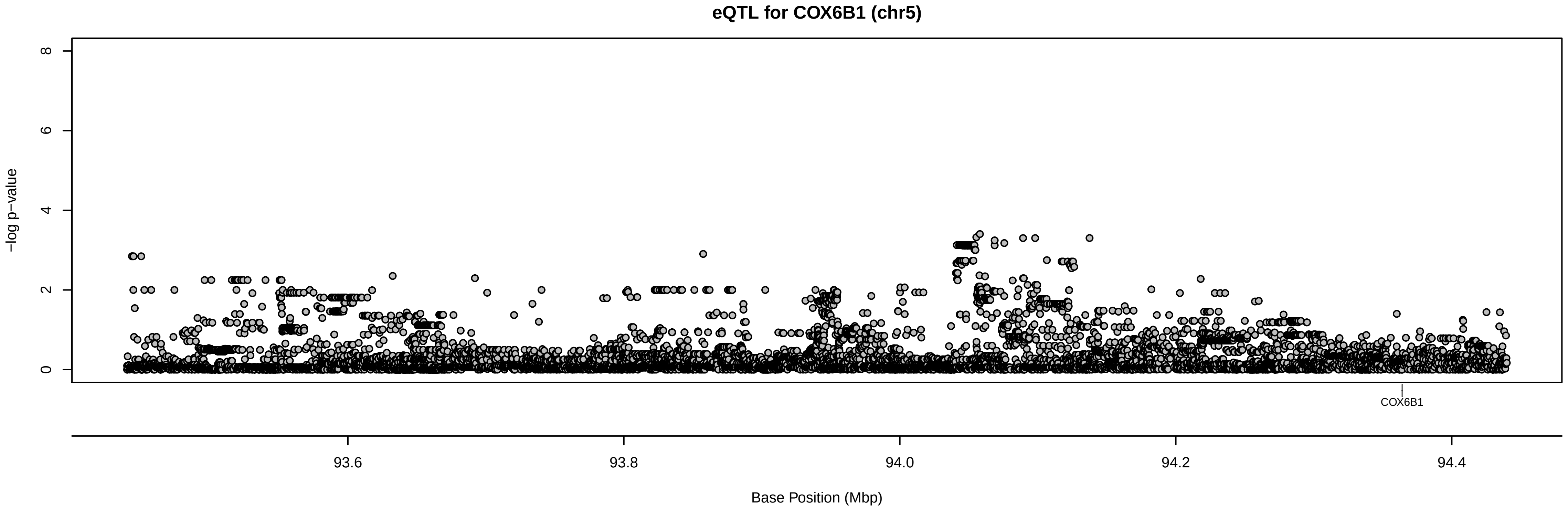

eQTL for COX6B1 (chr5)
−log p−value
0
2
4
6
8
COX6B1
93.6
93.8
94.0
94.2
94.4
Base Position (Mbp)

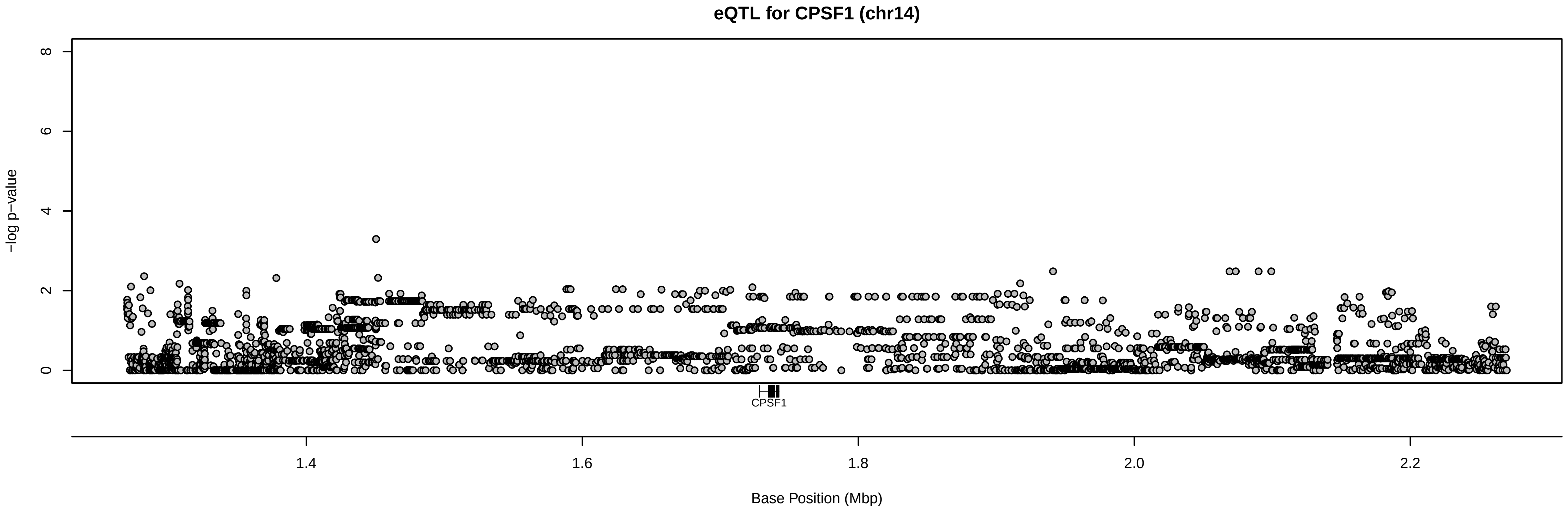
eQTL for CPSF1 (chr14)
−log p−value
0
2
4
6
8
CPSF1
1.4
1.6
1.8
2.0
2.2
Base Position (Mbp)

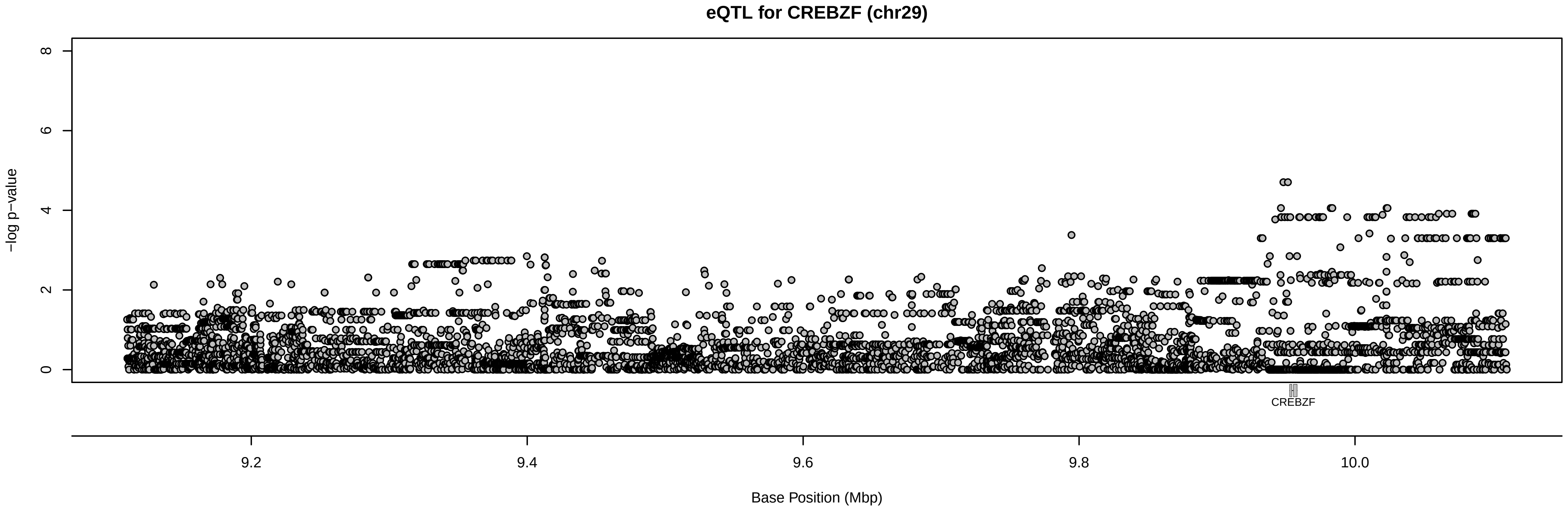
eQTL for CREBZF (chr29)
−log p−value
0
2
4
6
8
CREBZF
9.2
9.4
9.6
9.8
10.0
Base Position (Mbp)

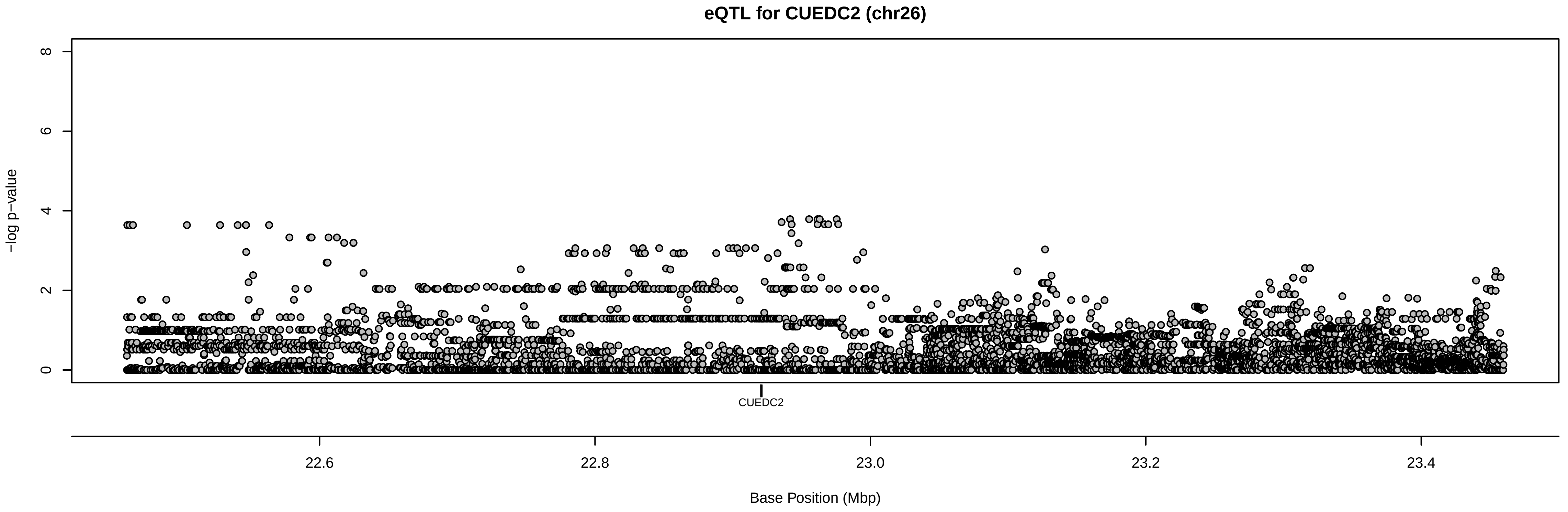
eQTL for CUEDC2 (chr26)
−log p−value
0
2
4
6
8
CUEDC2
22.6
22.8
23.0
23.2
23.4
Base Position (Mbp)

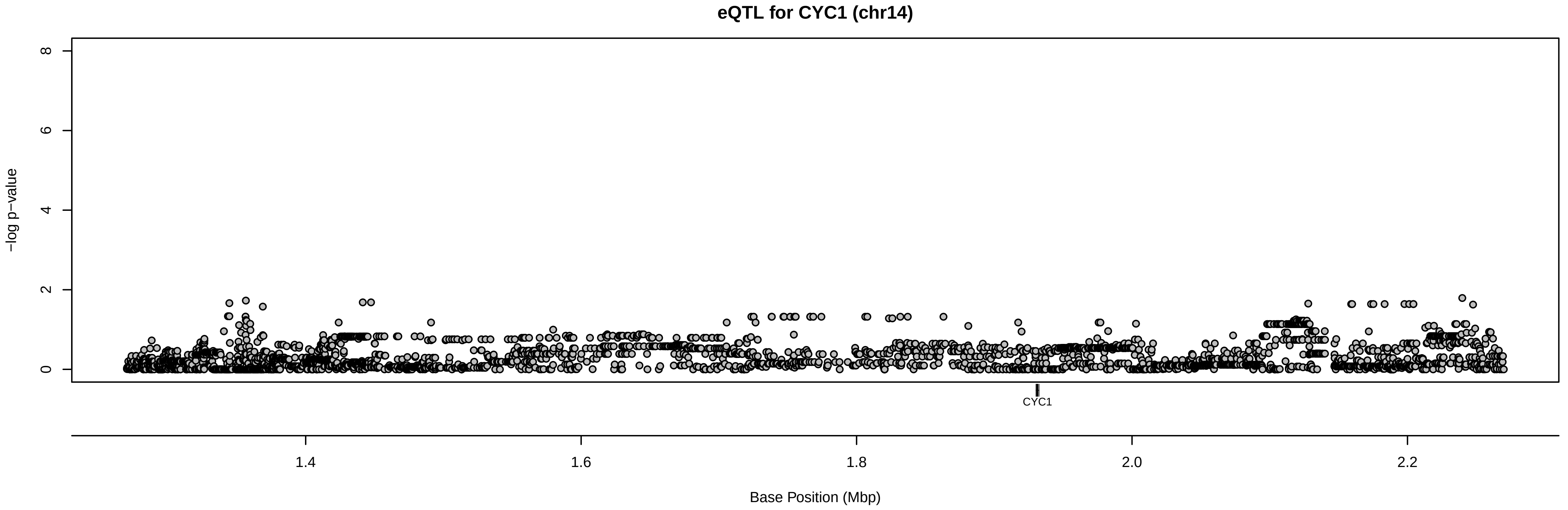
eQTL for CYC1 (chr14)
−log p−value
0
2
4
6
8
CYC1
1.4
1.6
1.8
2.0
2.2
Base Position (Mbp)

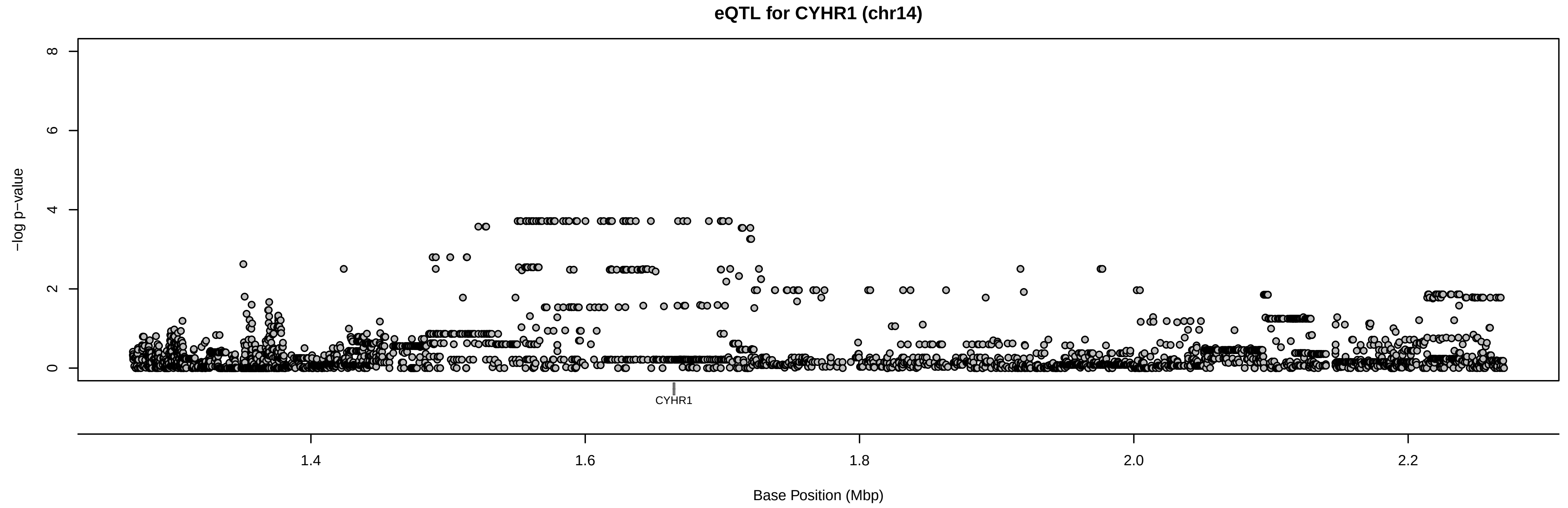
eQTL for CYHR1 (chr14)
−log p−value
0
2
4
6
8
CYHR1
1.4
1.6
1.8
2.0
2.2
Base Position (Mbp)

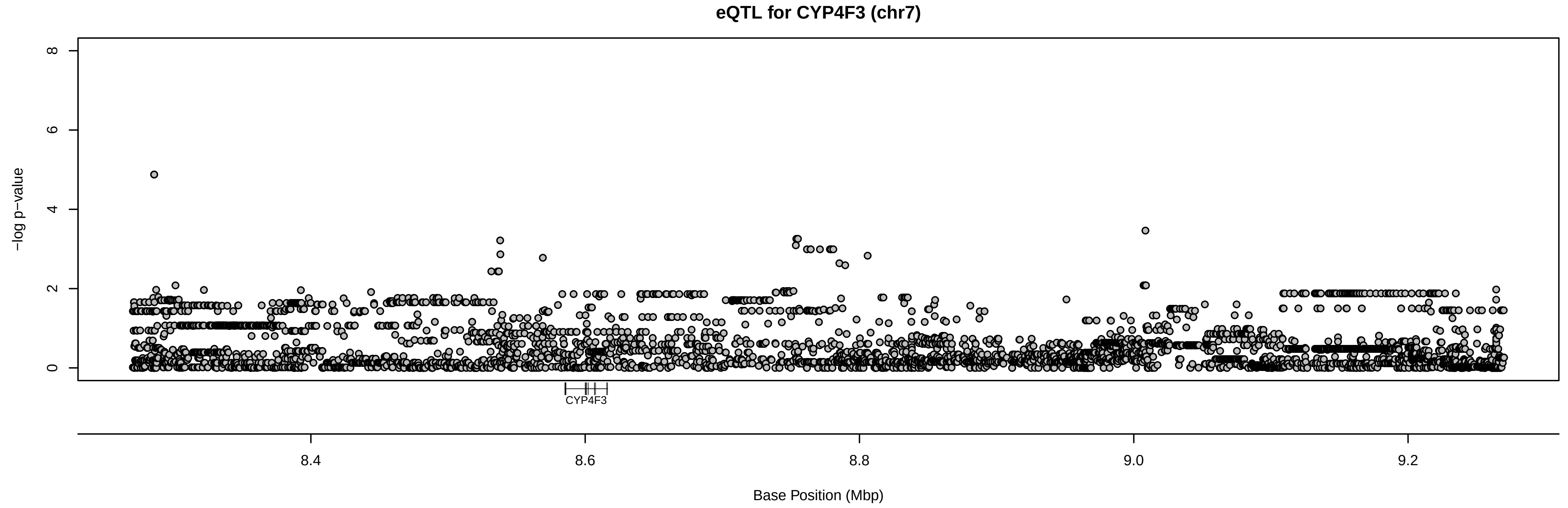
eQTL for CYP4F3 (chr7)
−log p−value
0
2
4
6
8
CYP4F3
8.4
8.6
8.8
9.0
9.2
Base Position (Mbp)

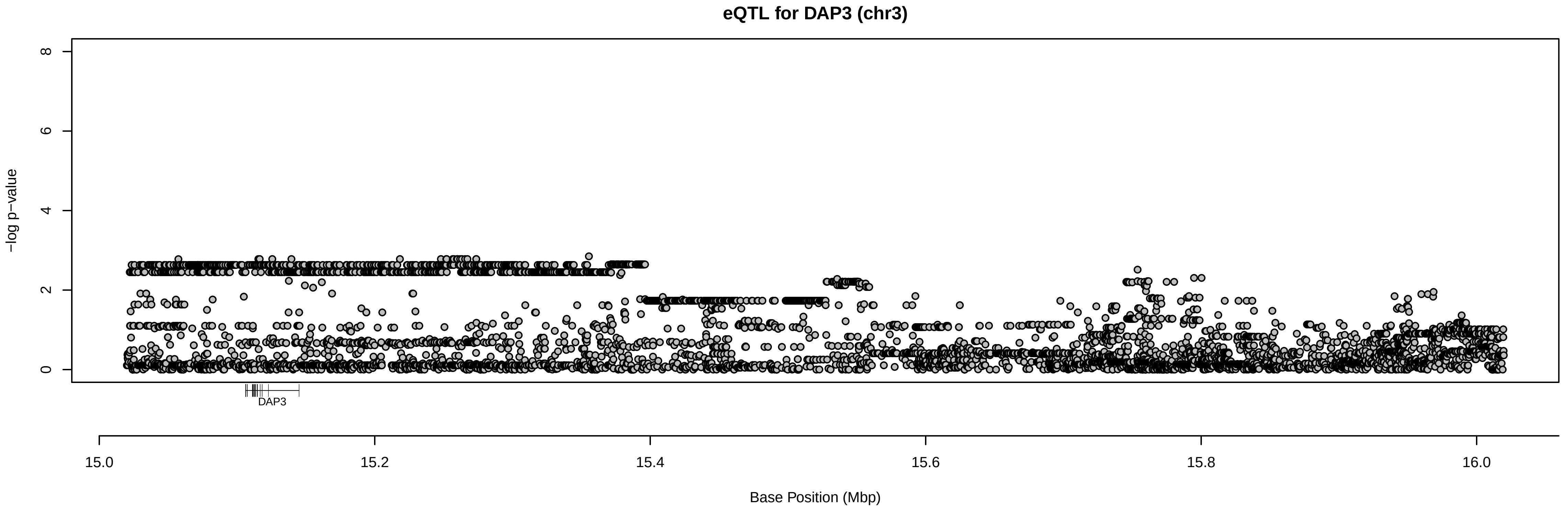
eQTL for DAP3 (chr3)
−log p−value
0
2
4
6
8
DAP3
15.0
15.2
15.4
15.6
15.8
16.0
Base Position (Mbp)

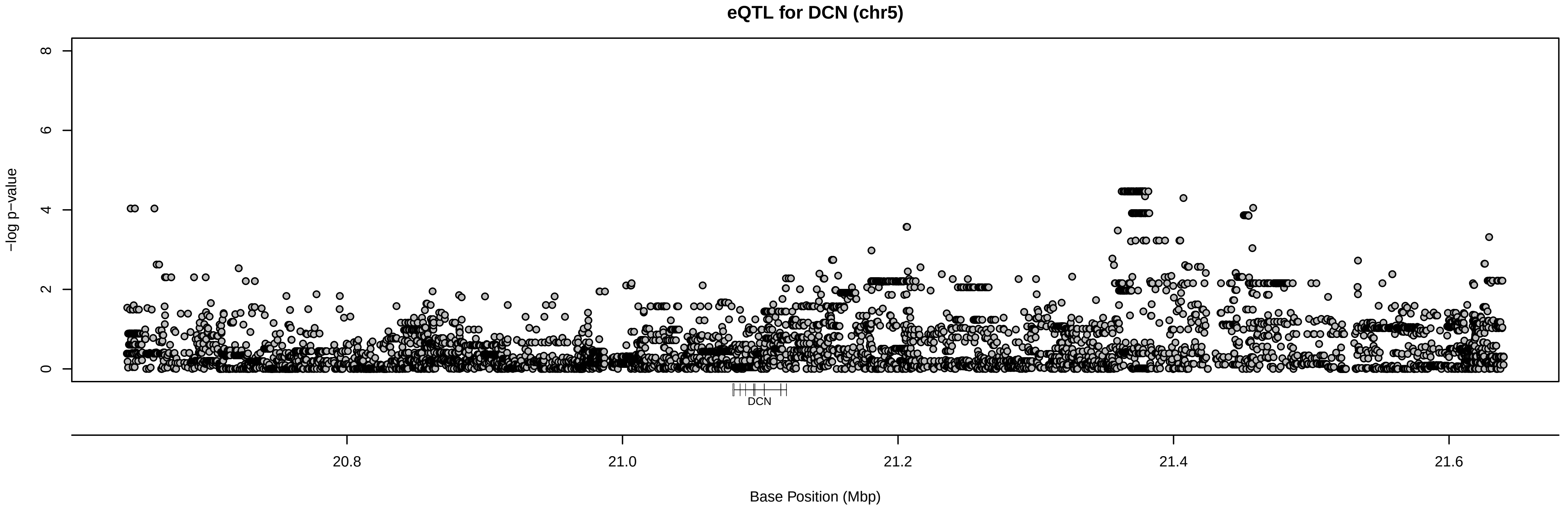
eQTL for DCN (chr5)
−log p−value
0
2
4
6
8
DCN
20.8
21.0
21.2
21.4
21.6
Base Position (Mbp)

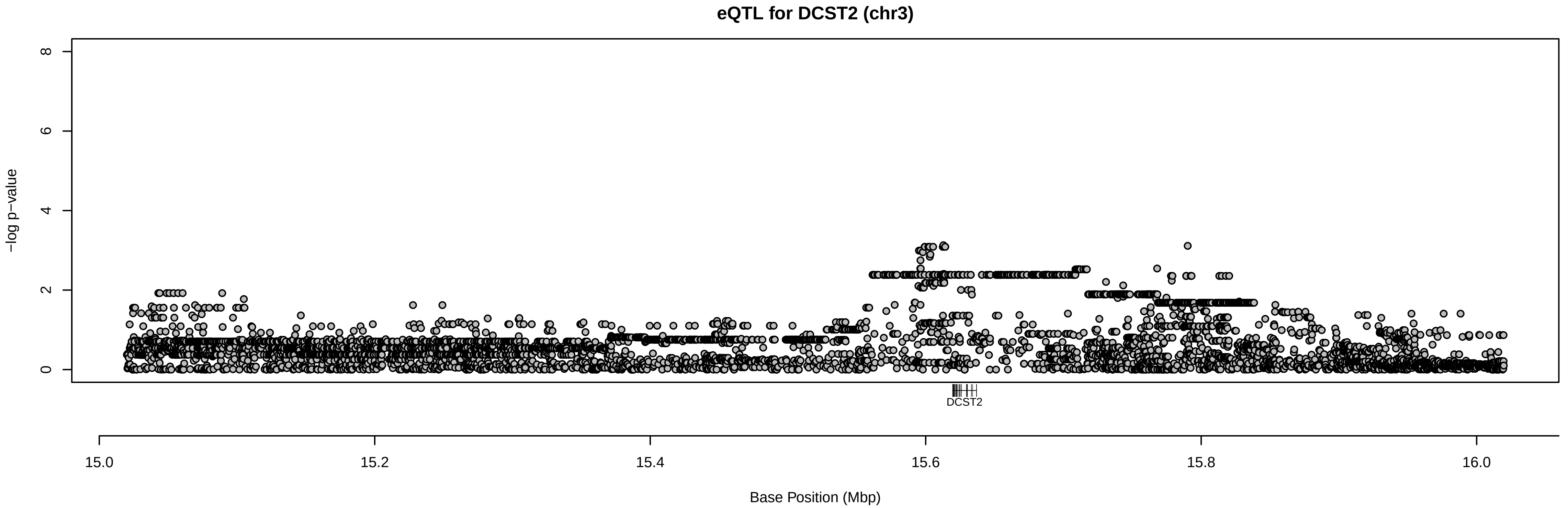

eQTL for DCST2 (chr3)
−log p−value
0
2
4
6
8
DCST2
15.0
15.2
15.4
15.6
15.8
16.0
Base Position (Mbp)

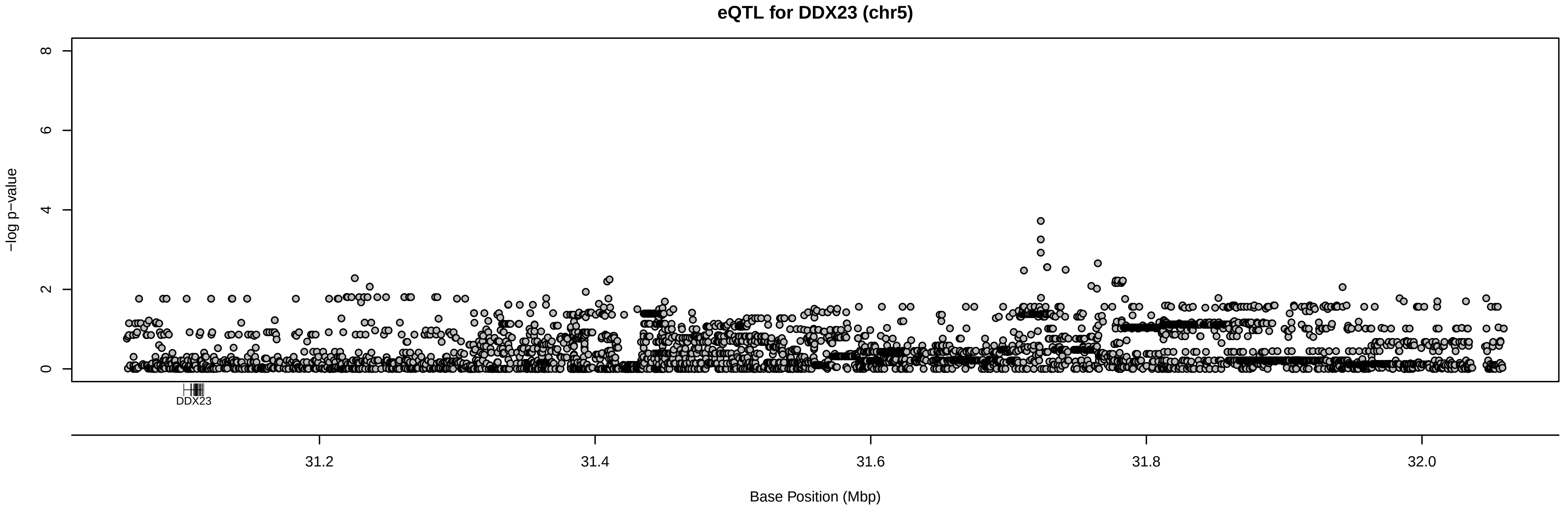

eQTL for DDX23 (chr5)
−log p−value
0
2
4
6
8
DDX23
31.2
31.4
31.6
31.8
32.0
Base Position (Mbp)

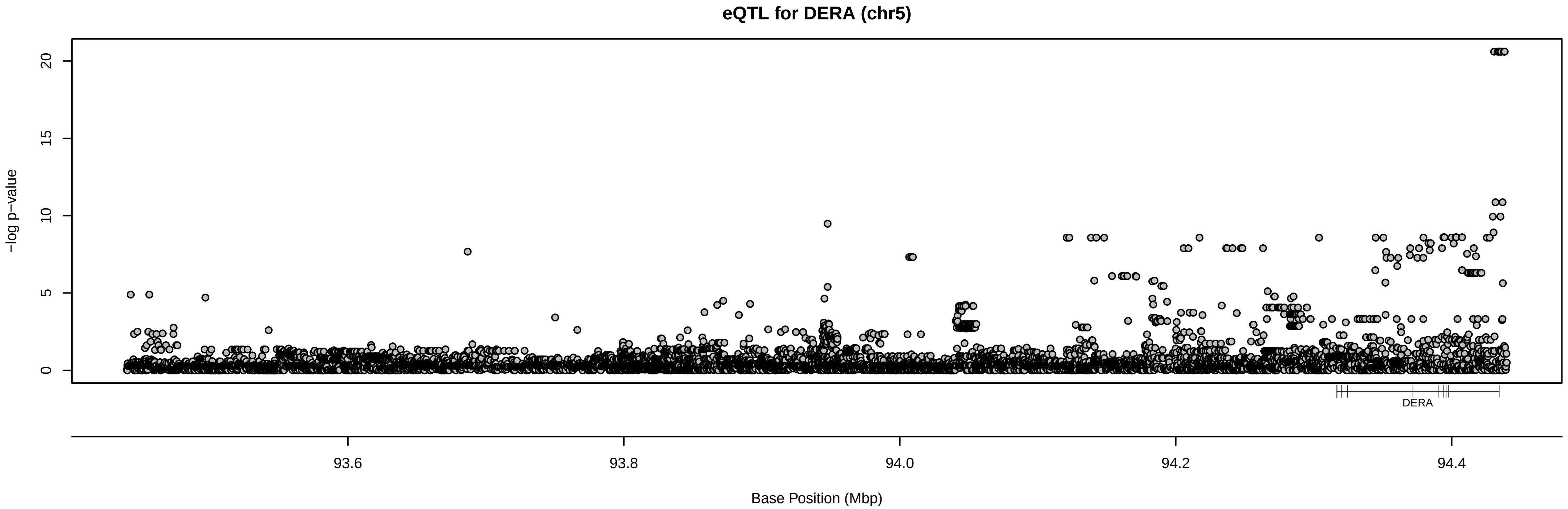

eQTL for DERA (chr5)
−log p−value
0
5
10
15
20
DERA
93.6
93.8
94.0
94.2
94.4
Base Position (Mbp)

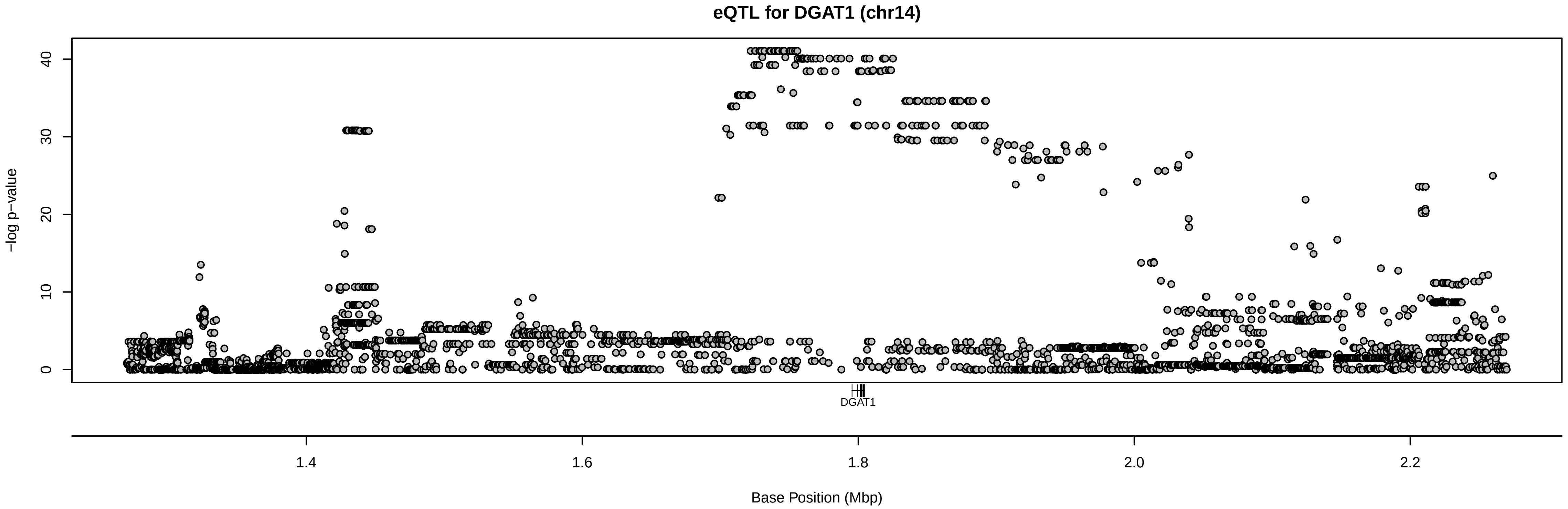

eQTL for DGAT1 (chr14)
−log p−value
0
10
20
30
40
DGAT1
1.4
1.6
1.8
2.0
2.2
Base Position (Mbp)

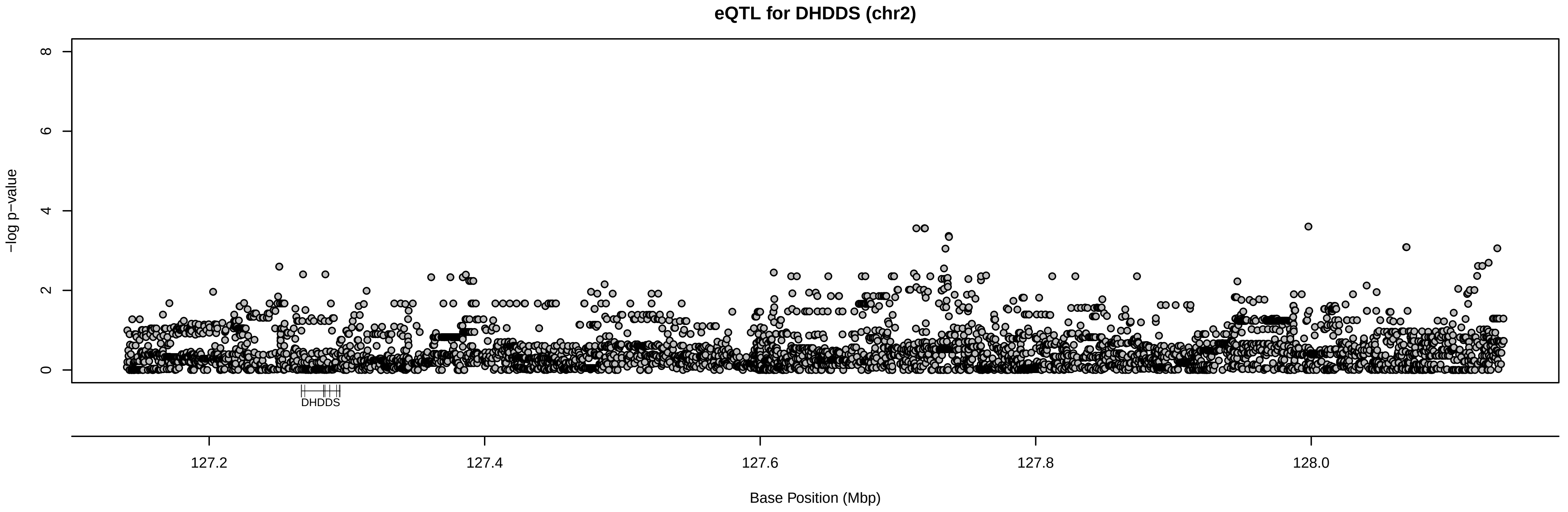
eQTL for DHDDS (chr2)
−log p−value
0
2
4
6
8
DHDDS
127.2
127.4
127.6
127.8
128.0
Base Position (Mbp)

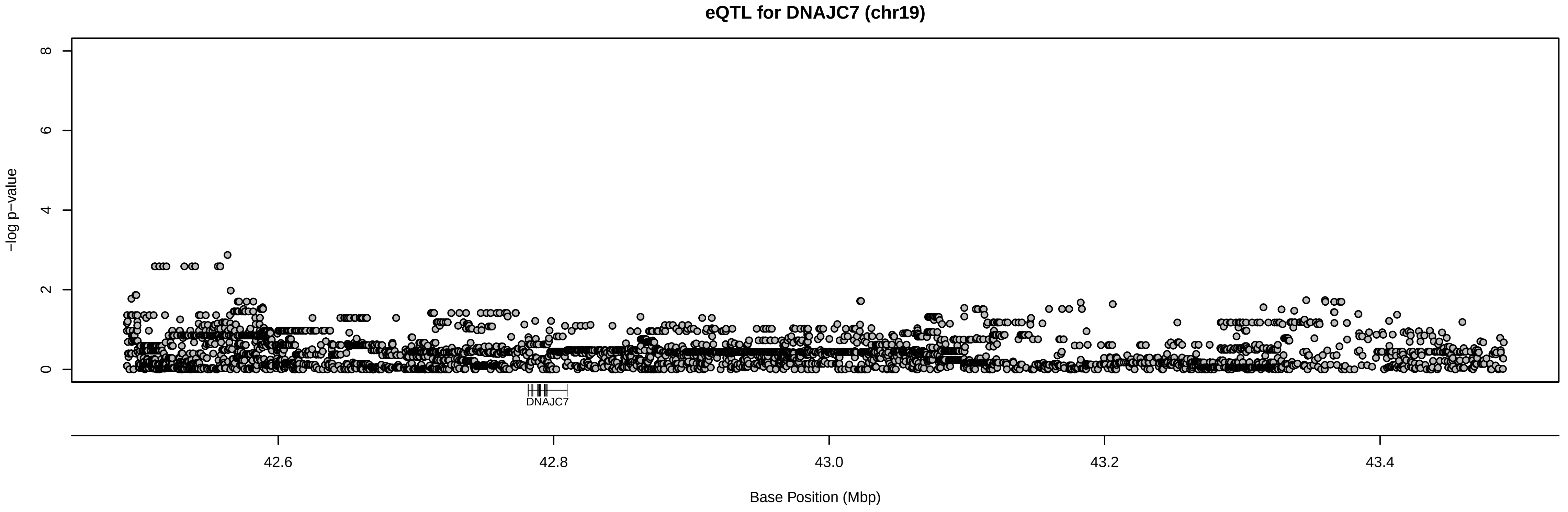
eQTL for DNAJC7 (chr19)
−log p−value
0
2
4
6
8
DNAJC7
42.6
42.8
43.0
43.2
43.4
Base Position (Mbp)

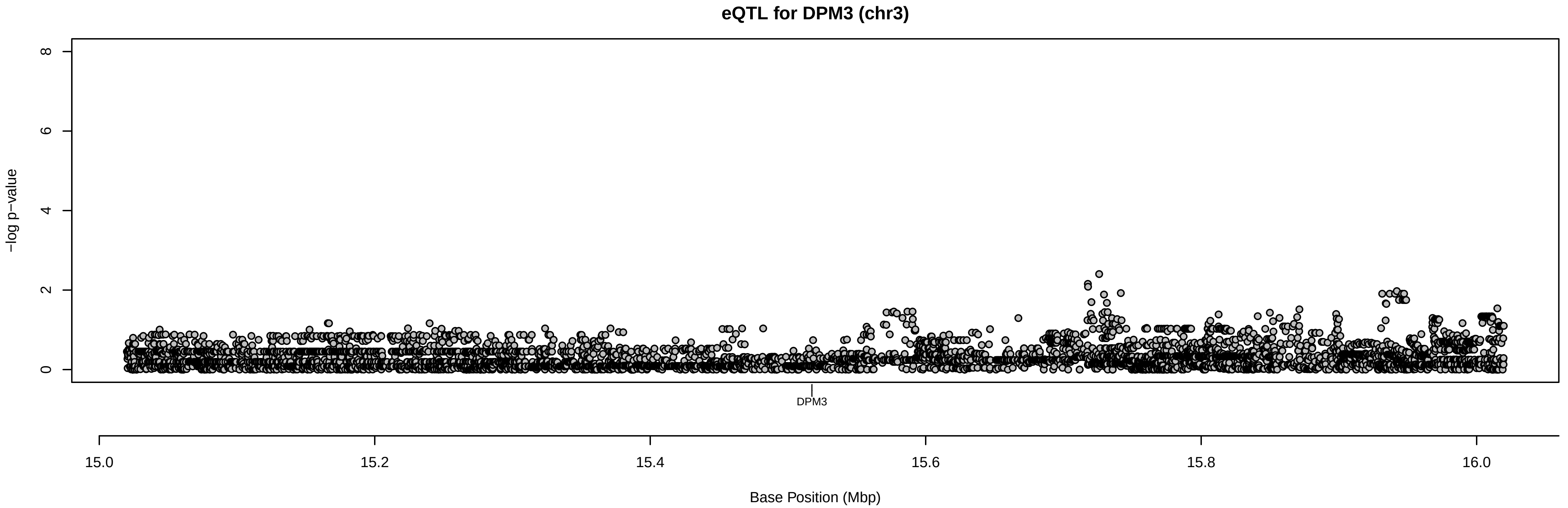
eQTL for DPM3 (chr3)
−log p−value
0
2
4
6
8
DPM3
15.0
15.2
15.4
15.6
15.8
16.0
Base Position (Mbp)

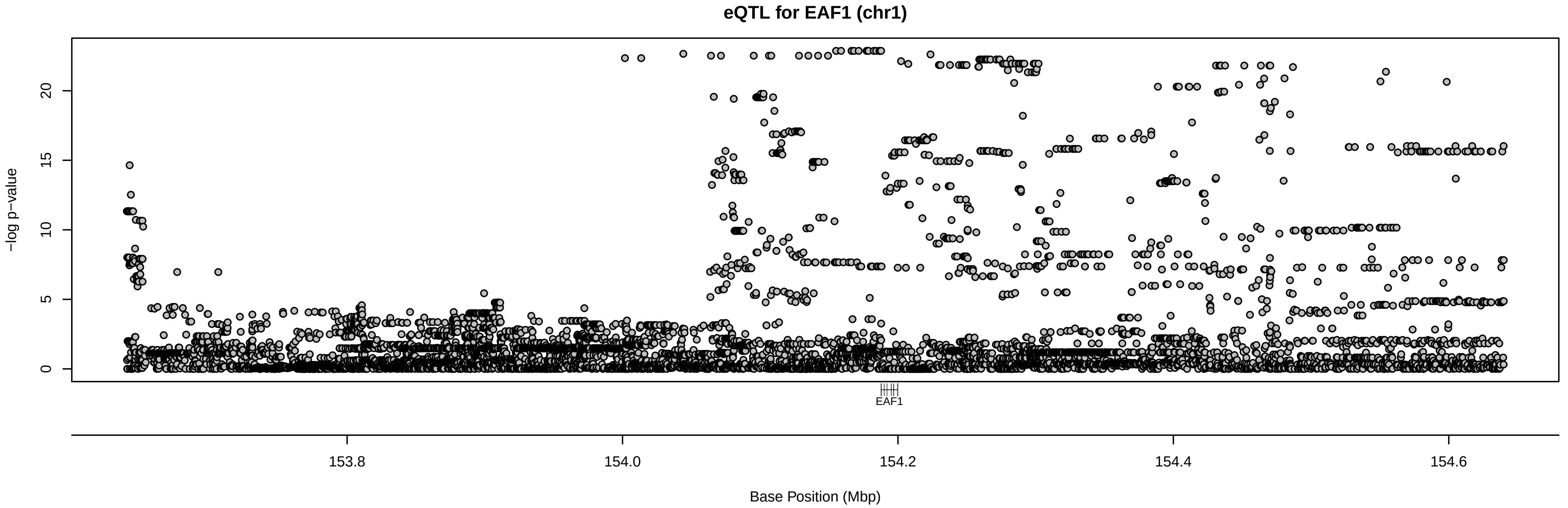
eQTL for EAF1 (chr1)
−log p−value
0
5
10
15
20
EAF1
153.8
154.0
154.2
154.4
154.6
Base Position (Mbp)

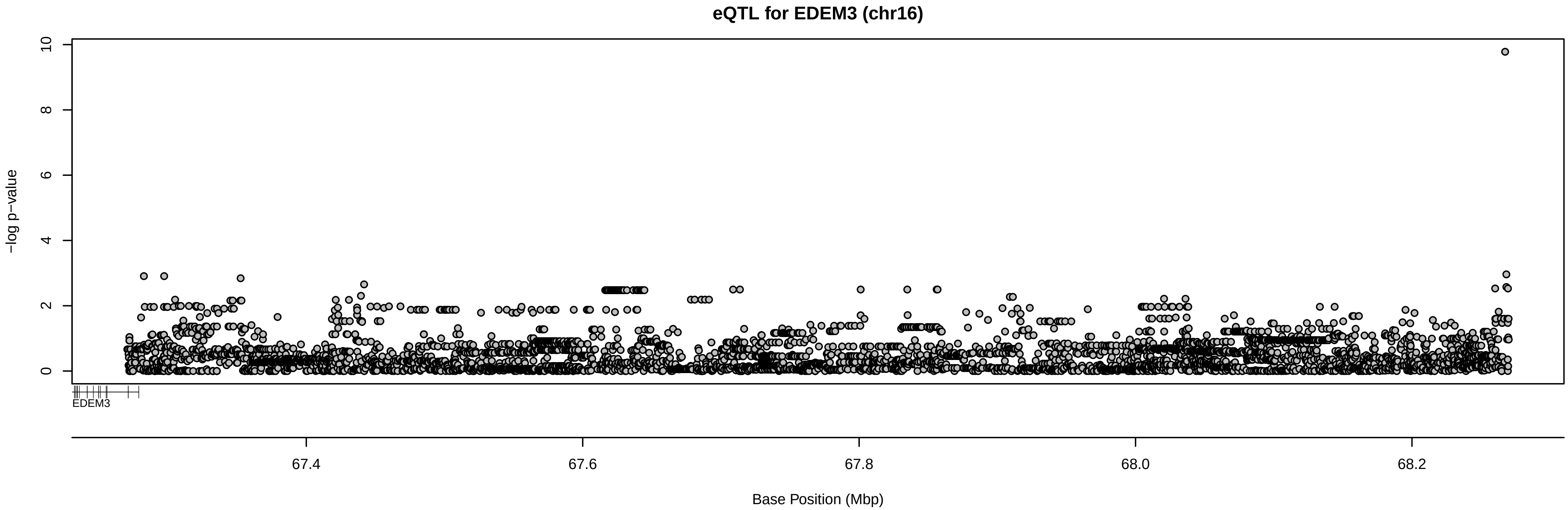

eQTL for EDEM3 (chr16)
−log p−value
0
2
4
6
8
10
EDEM3
67.4
67.6
67.8
68.0
68.2
Base Position (Mbp)

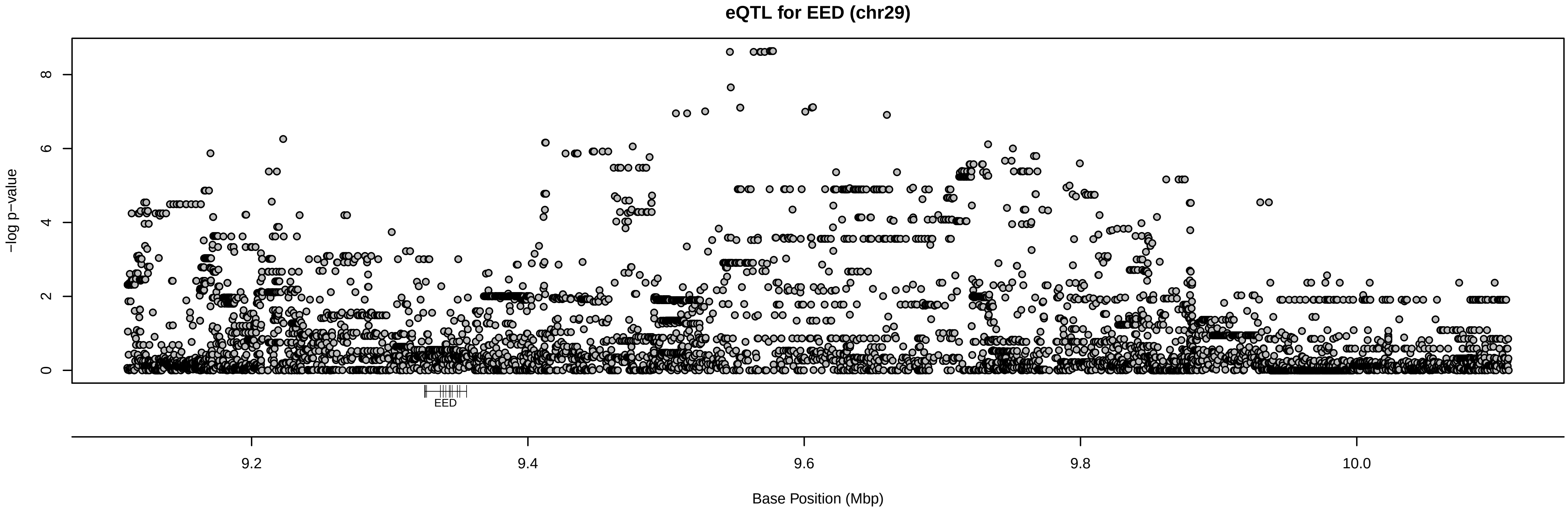

eQTL for EED (chr29)
−log p−value
0
2
4
6
8
EED
9.2
9.4
9.6
9.8
10.0
Base Position (Mbp)

eQTL for EFNA1 (chr3)

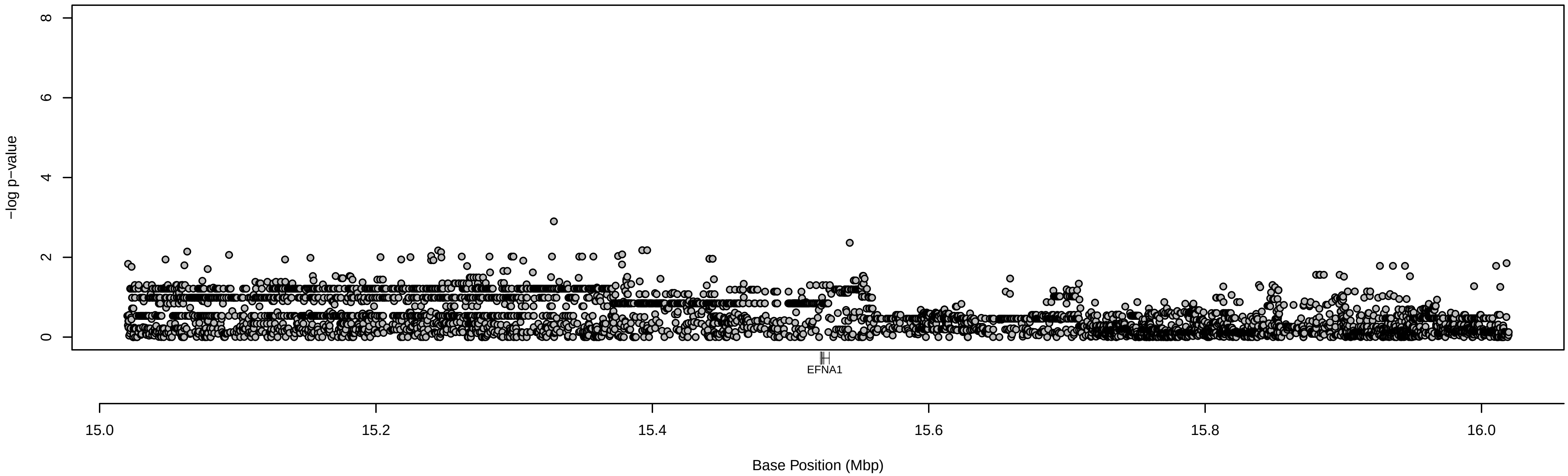

eQTL for EFNA3 (chr3)

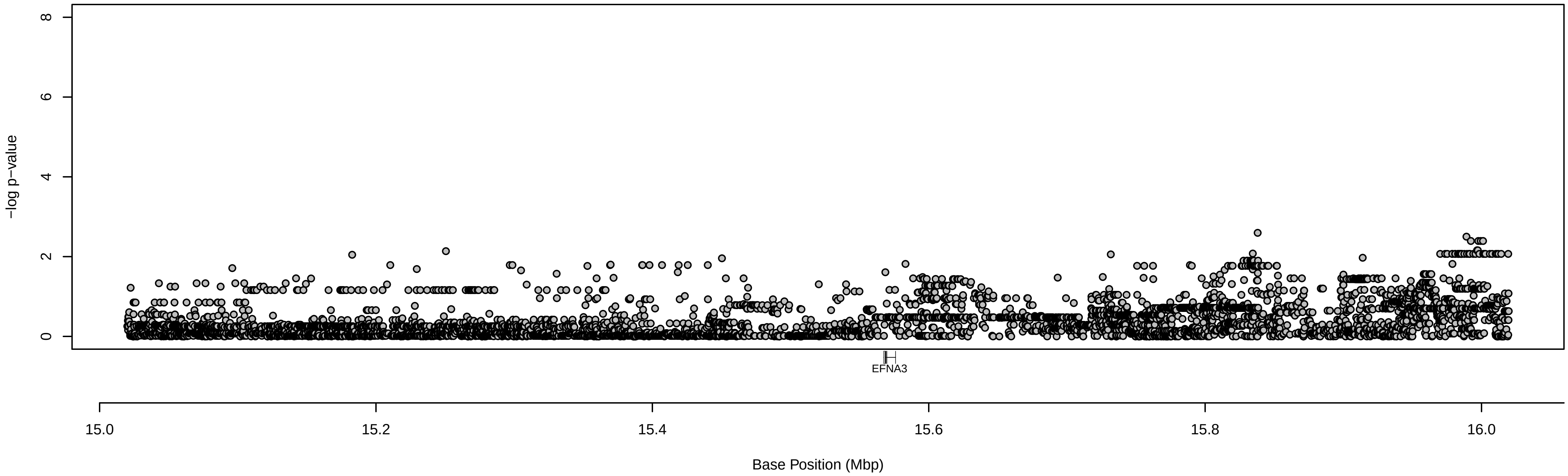

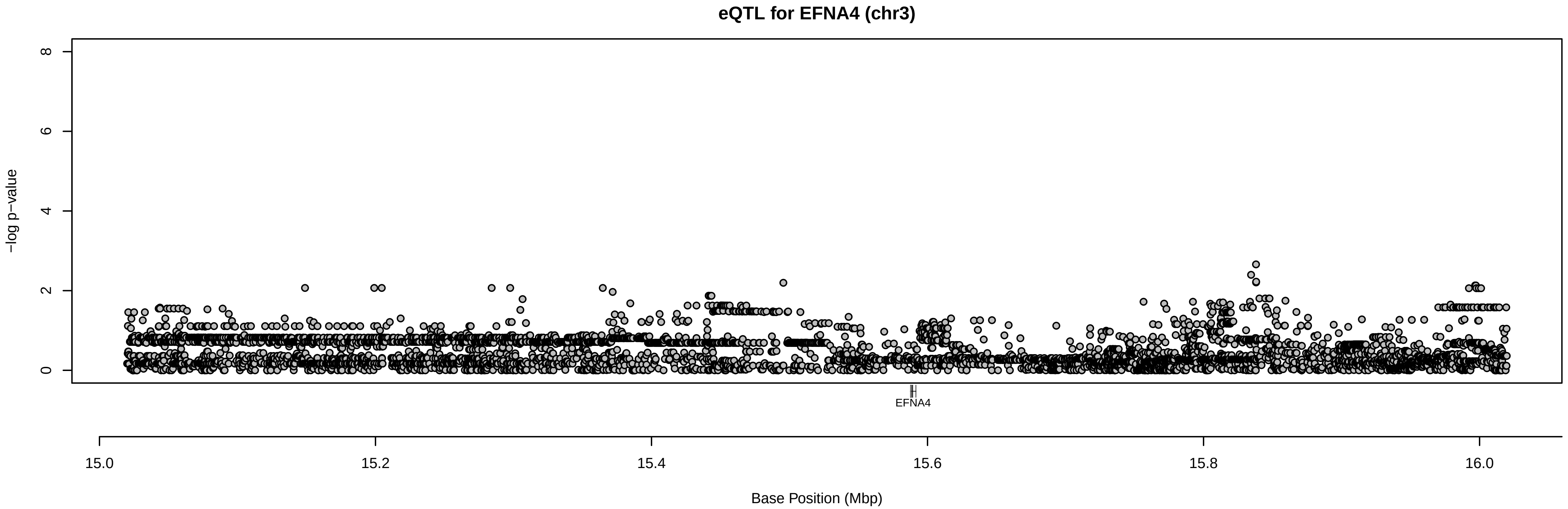

eQTL for EFNA4 (chr3)
−log p−value
0
2
4
6
8
EFNA4
15.0
15.2
15.4
15.6
15.8
16.0
Base Position (Mbp)

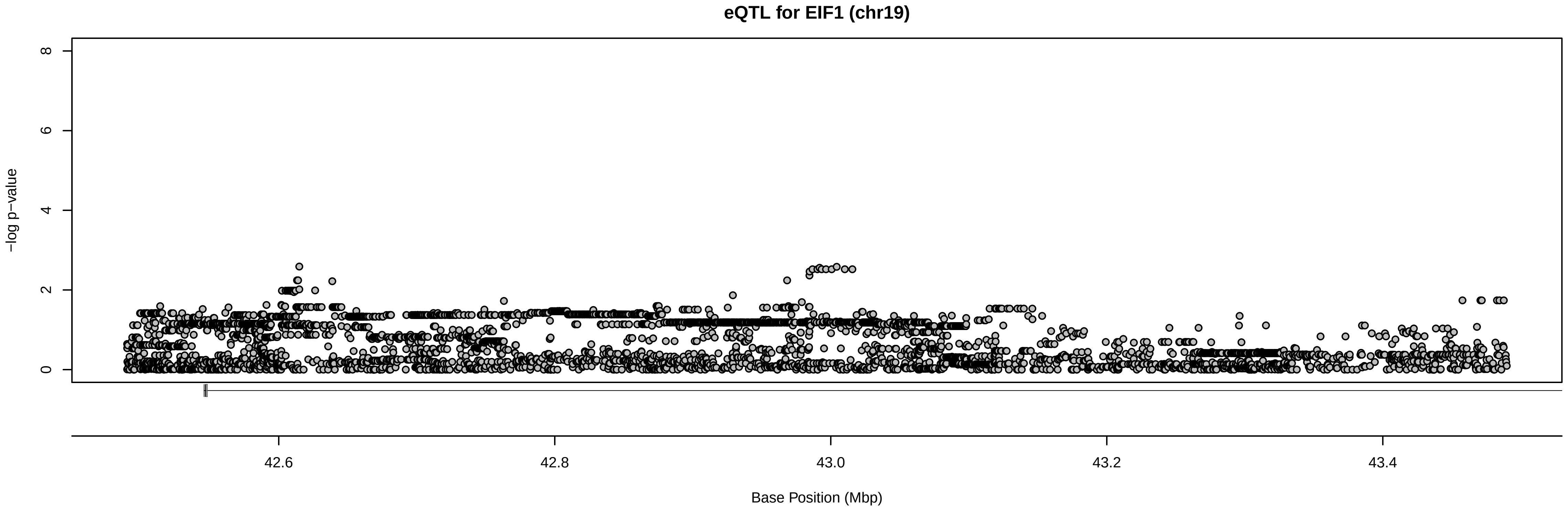

eQTL for EIF1 (chr19)
−log p−value
0
2
4
6
8
42.6
42.8
43.0
43.2
43.4
Base Position (Mbp)

eQTL for EPB41L4A (chr10)

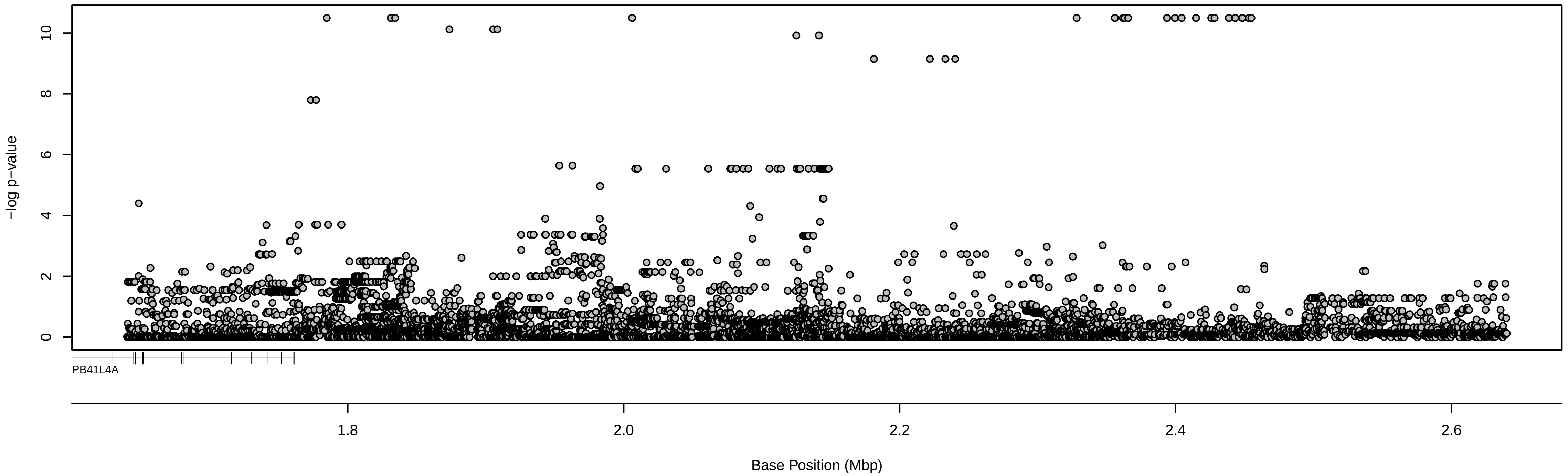

eQTL for EPHX3 (chr7)

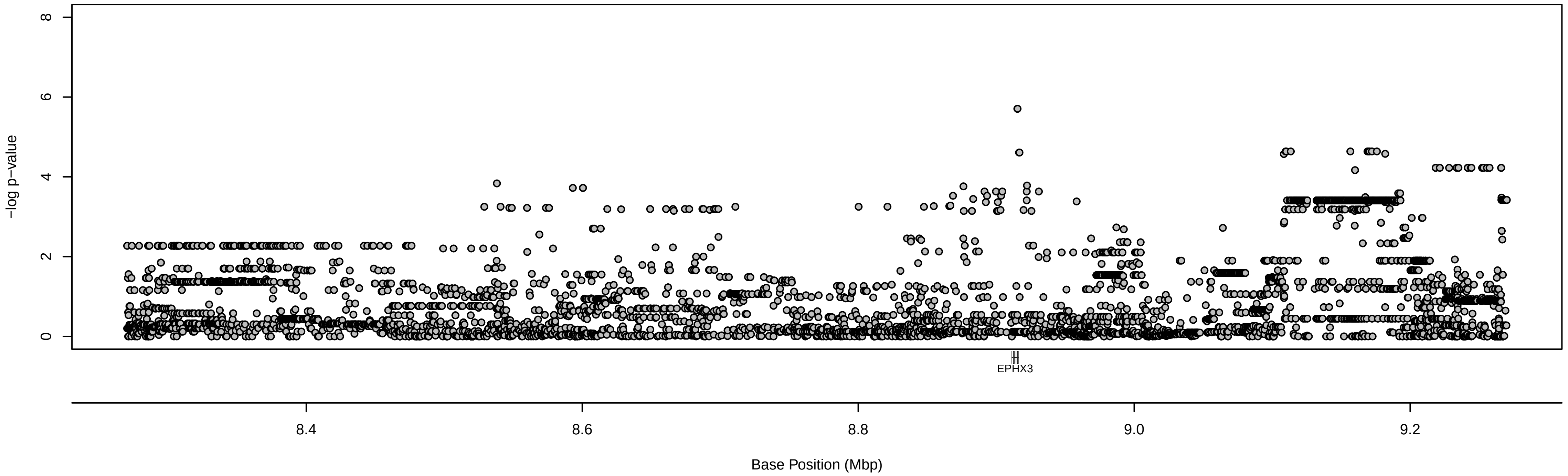

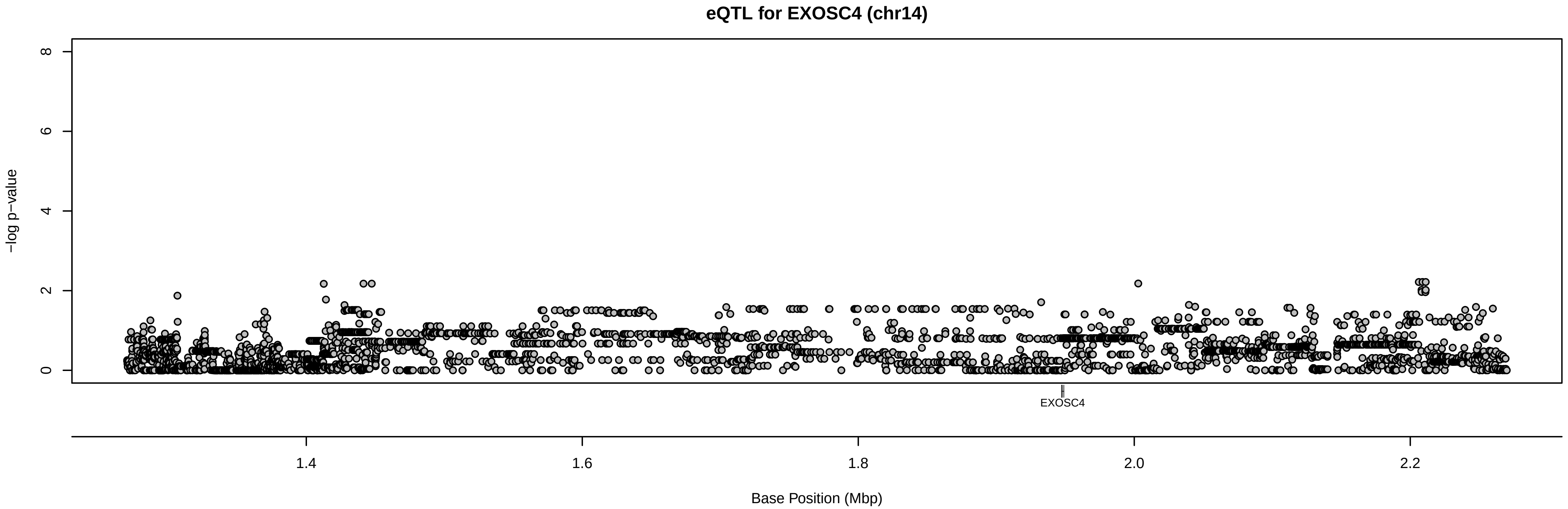
eQTL for EXOSC4 (chr14)
−log p−value
0
2
4
6
8
EXOSC4
1.4
1.6
1.8
2.0
2.2
Base Position (Mbp)

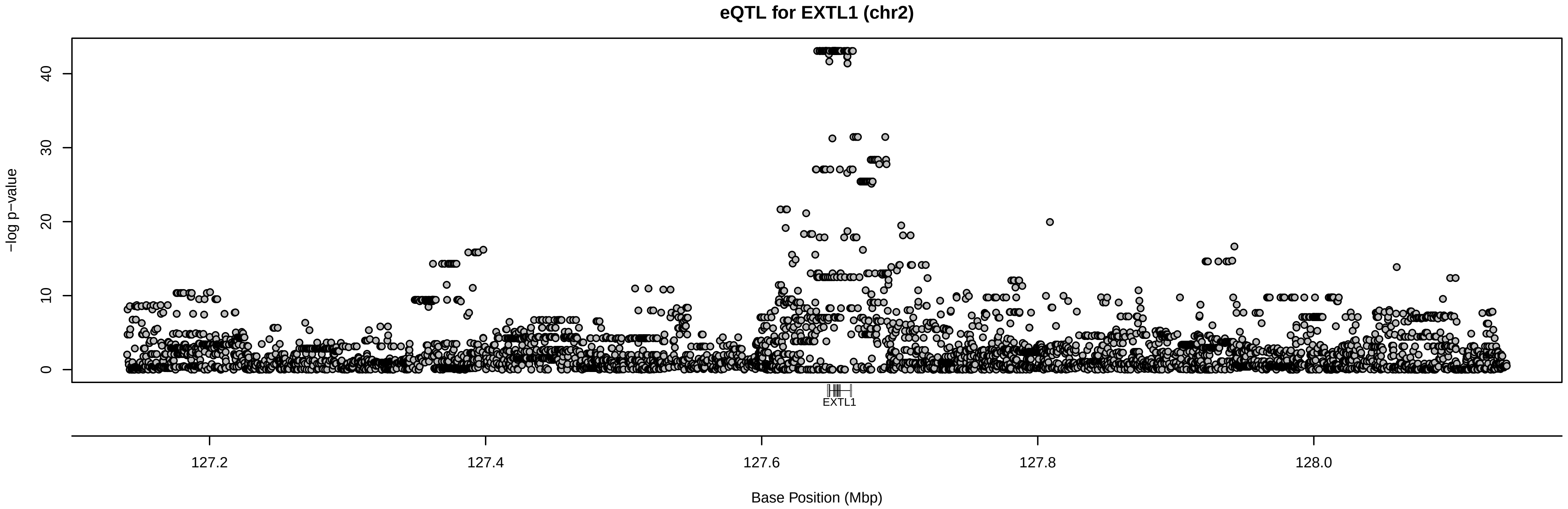
eQTL for EXTL1 (chr2)
−log p−value
0
10
20
30
40
EXTL1
127.2
127.4
127.6
127.8
128.0
Base Position (Mbp)

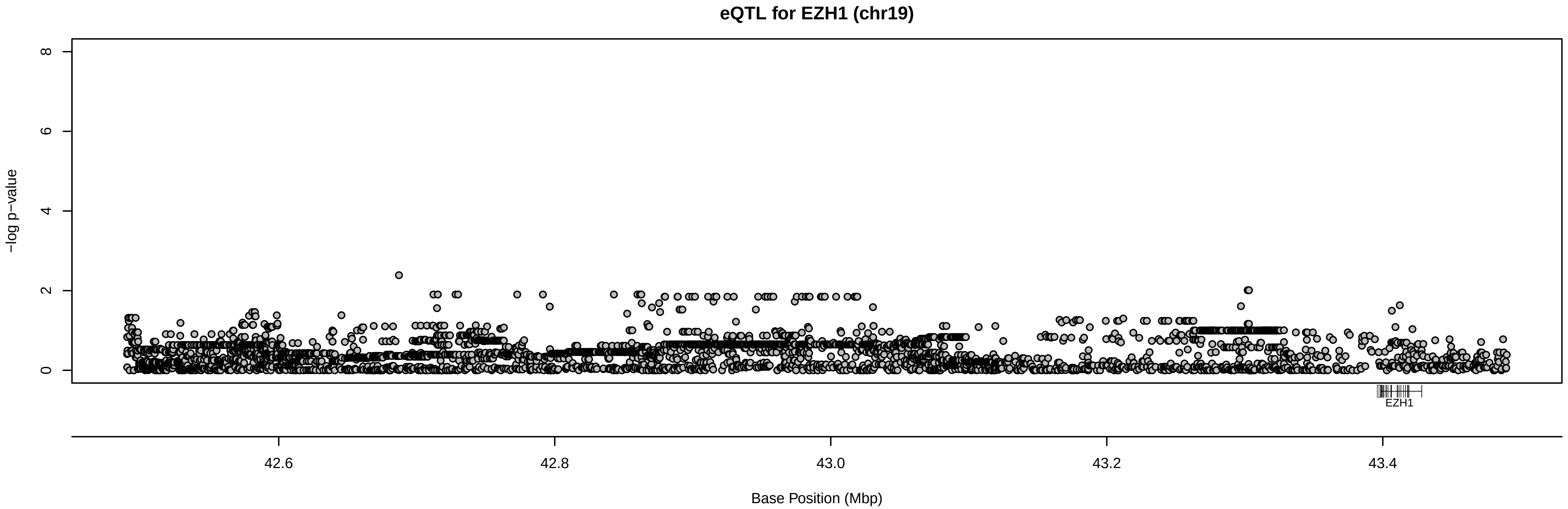
eQTL for EZH1 (chr19)
−log p−value
0
2
4
6
8
EZH1
42.6
42.8
43.0
43.2
43.4
Base Position (Mbp)

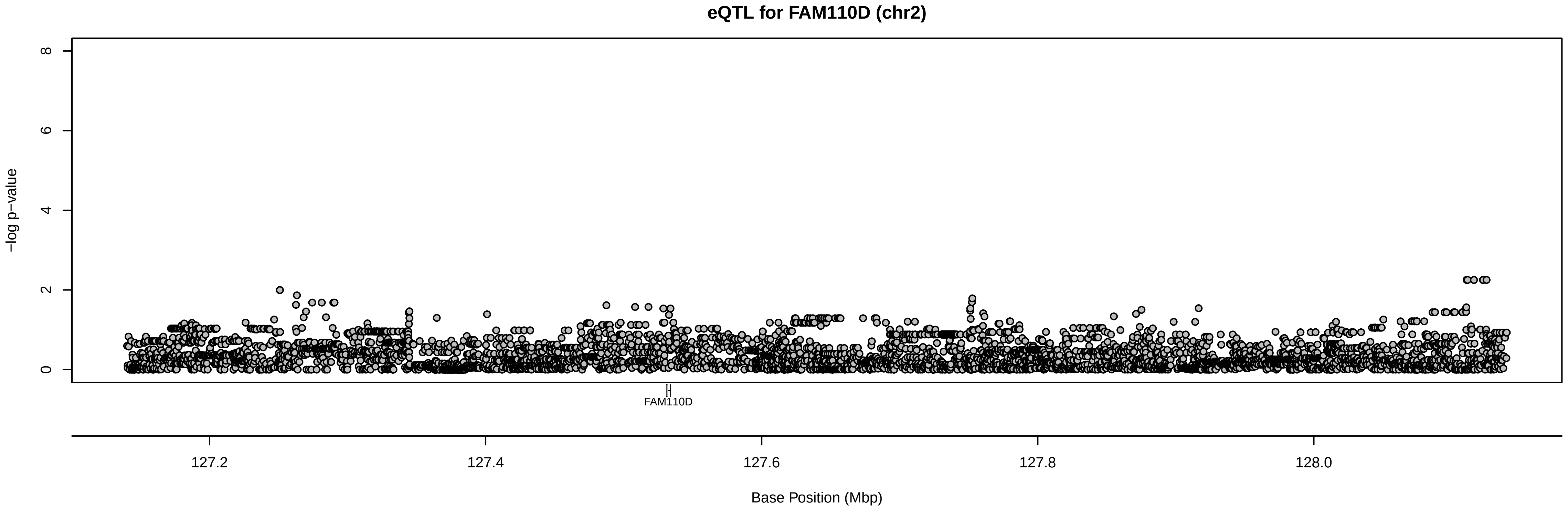
eQTL for FAM110D (chr2)
−log p−value
0
2
4
6
8
FAM110D
127.2
127.4
127.6
127.8
128.0
Base Position (Mbp)

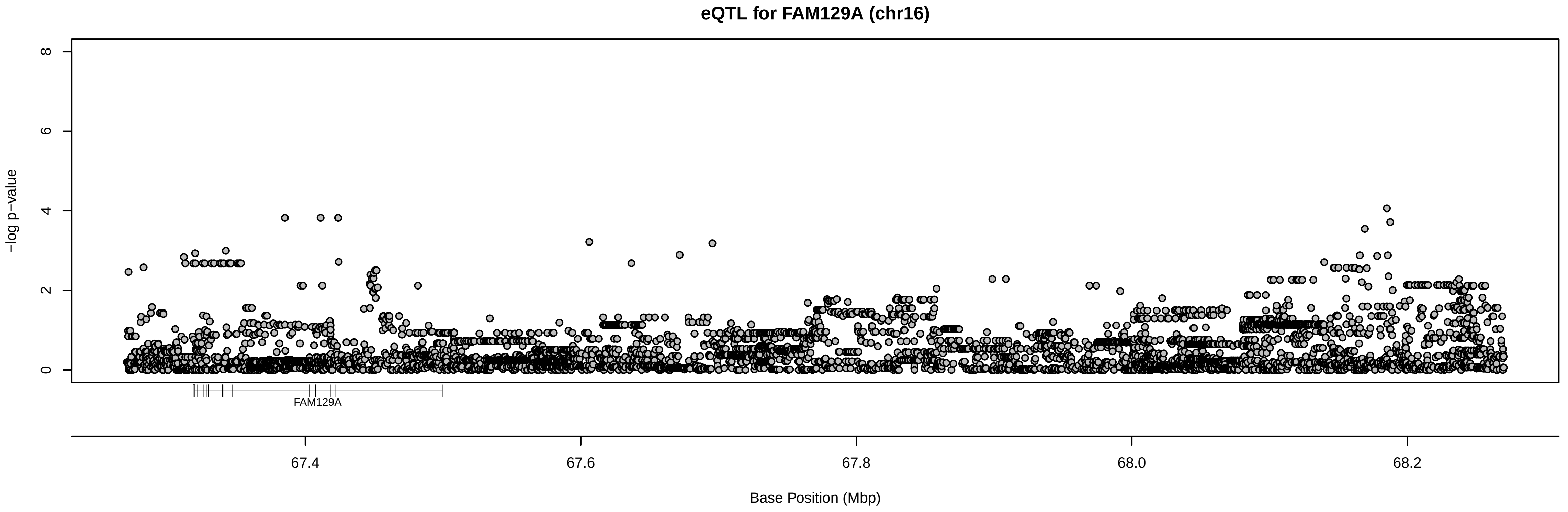
eQTL for FAM129A (chr16)
−log p−value
8
6
4
2
0
FAM129A
67.4
67.6
67.8
68.0
68.2
Base Position (Mbp)

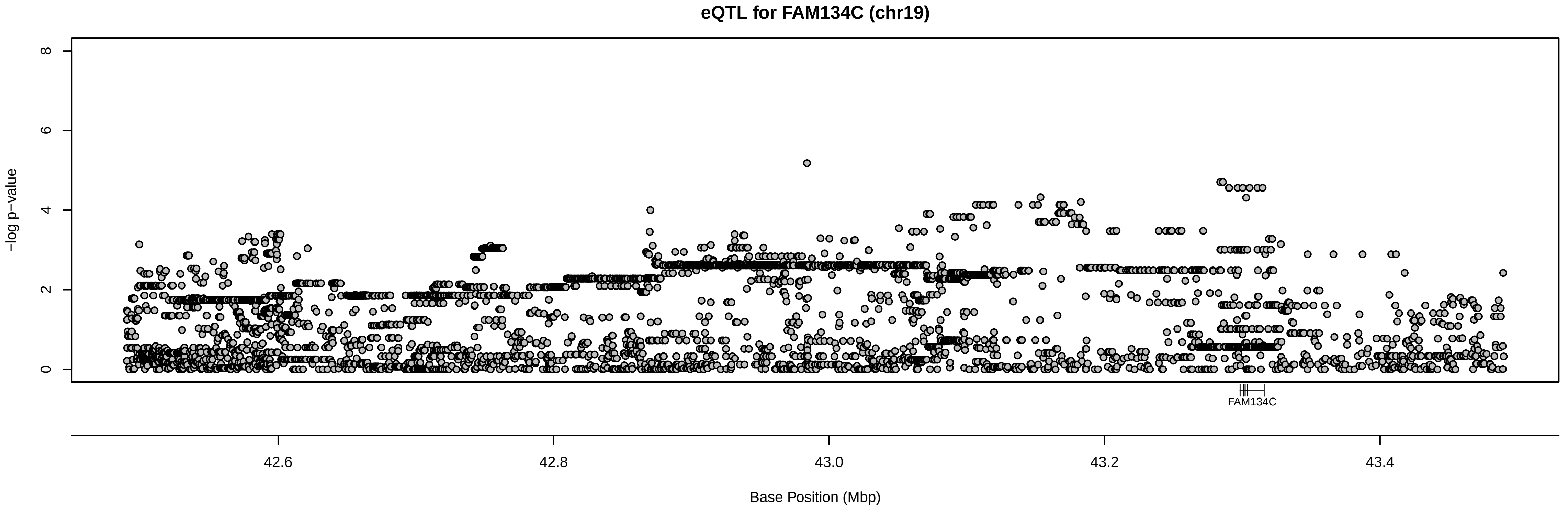
eQTL for FAM134C (chr19)
−log p−value
8
6
4
2
0
FAM134C
42.6
42.8
43.0
43.2
43.4
Base Position (Mbp)

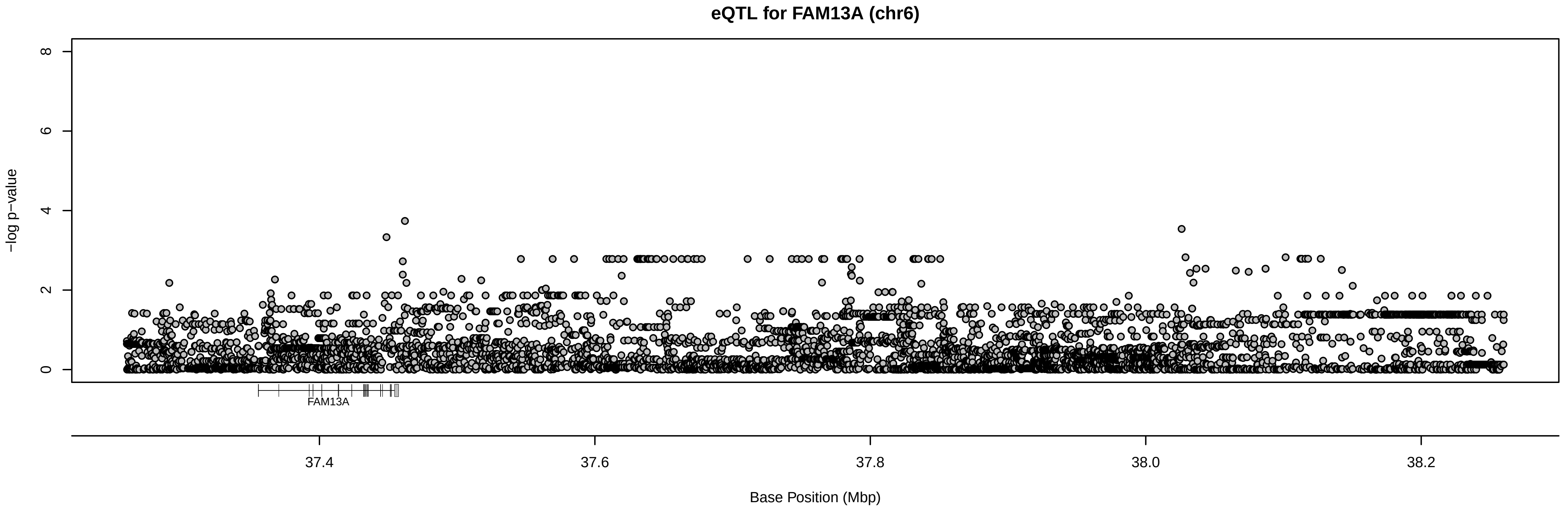
eQTL for FAM13A (chr6)
−log p−value
0
2
4
6
8
FAM13A
37.4
37.6
37.8
38.0
38.2
Base Position (Mbp)

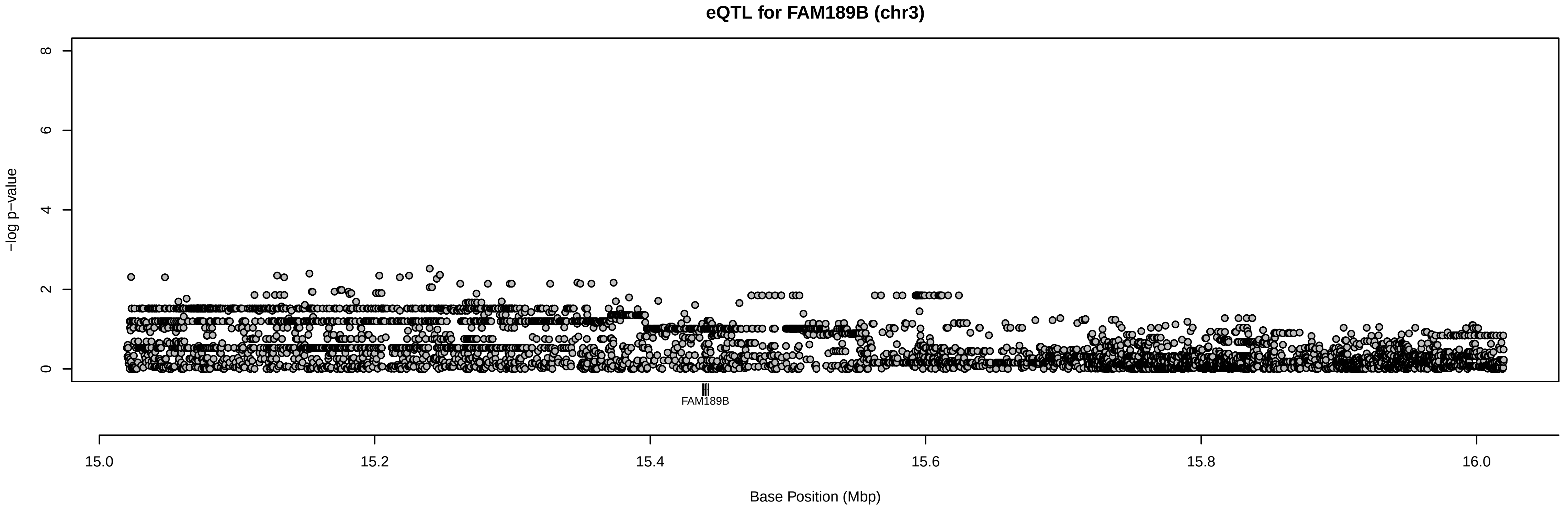
eQTL for FAM189B (chr3)
−log p−value
0
2
4
6
8
FAM189B
15.0
15.2
15.4
15.6
15.8
16.0
Base Position (Mbp)

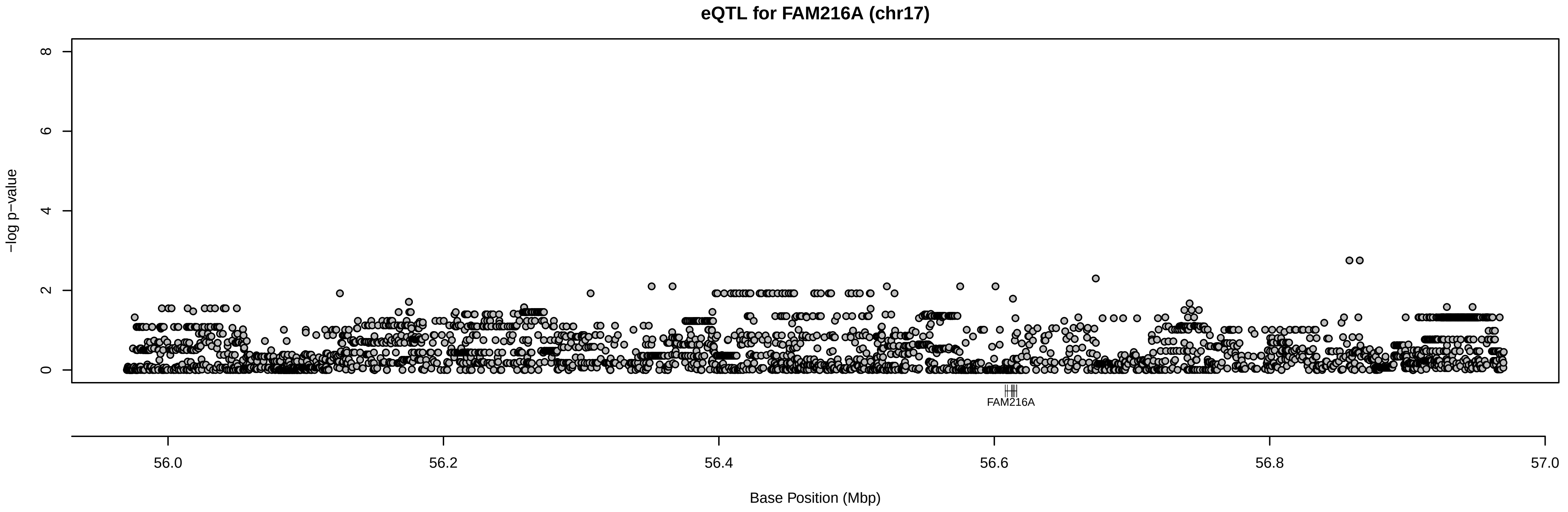
eQTL for FAM216A (chr17)
−log p−value
0
2
4
6
8
FAM216A
56.0
56.2
56.4
56.6
56.8
57.0
Base Position (Mbp)

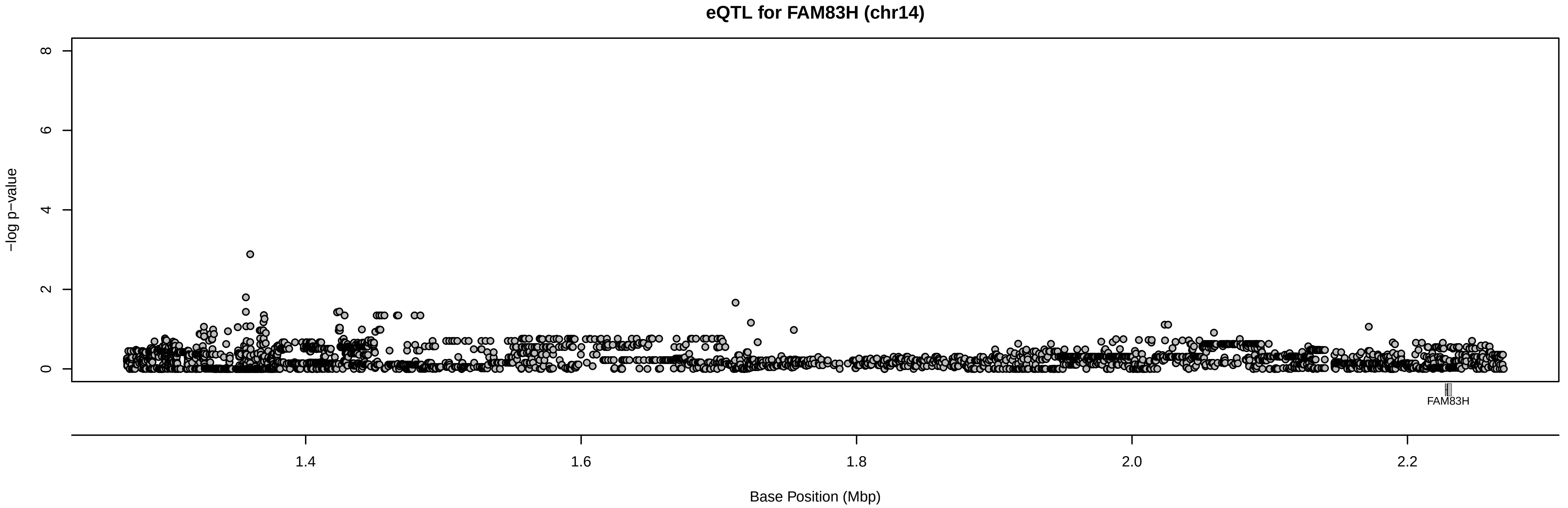
eQTL for FAM83H (chr14)
−log p−value
0
2
4
6
8
FAM83H
1.4
1.6
1.8
2.0
2.2
Base Position (Mbp)

eQTL for FBXL15 (chr26)

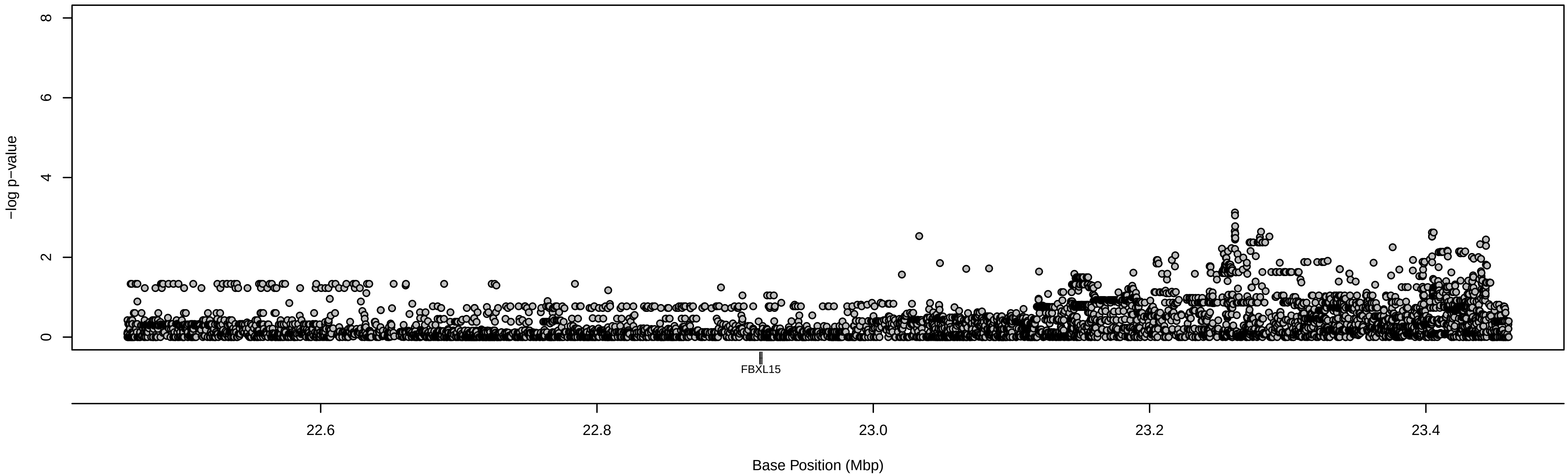

eQTL for FBXL6 (chr14)

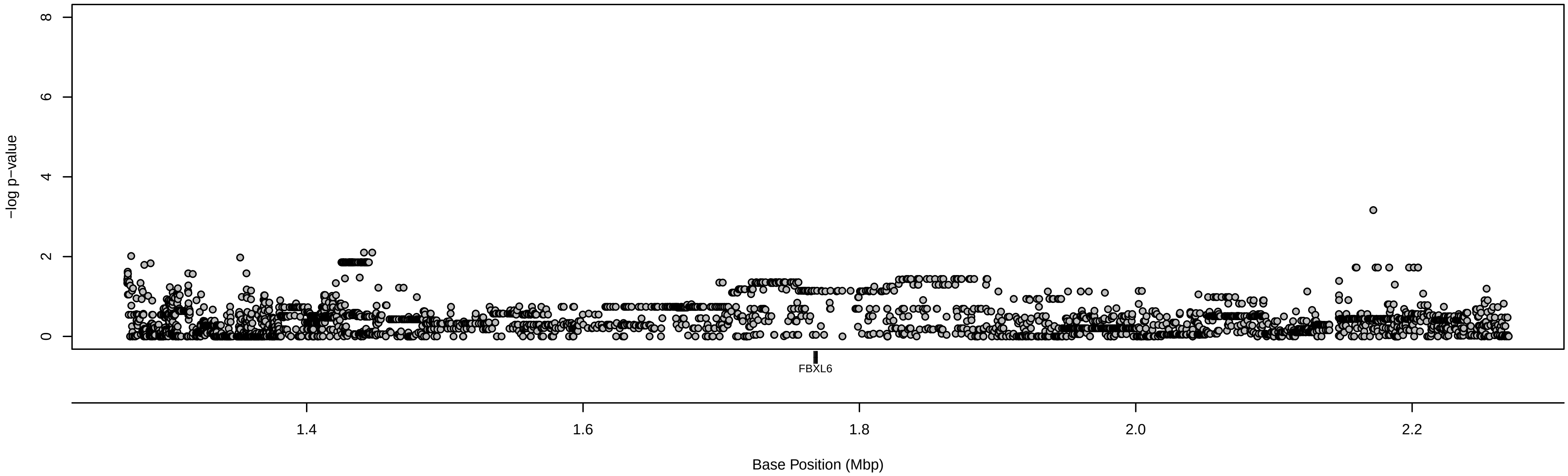

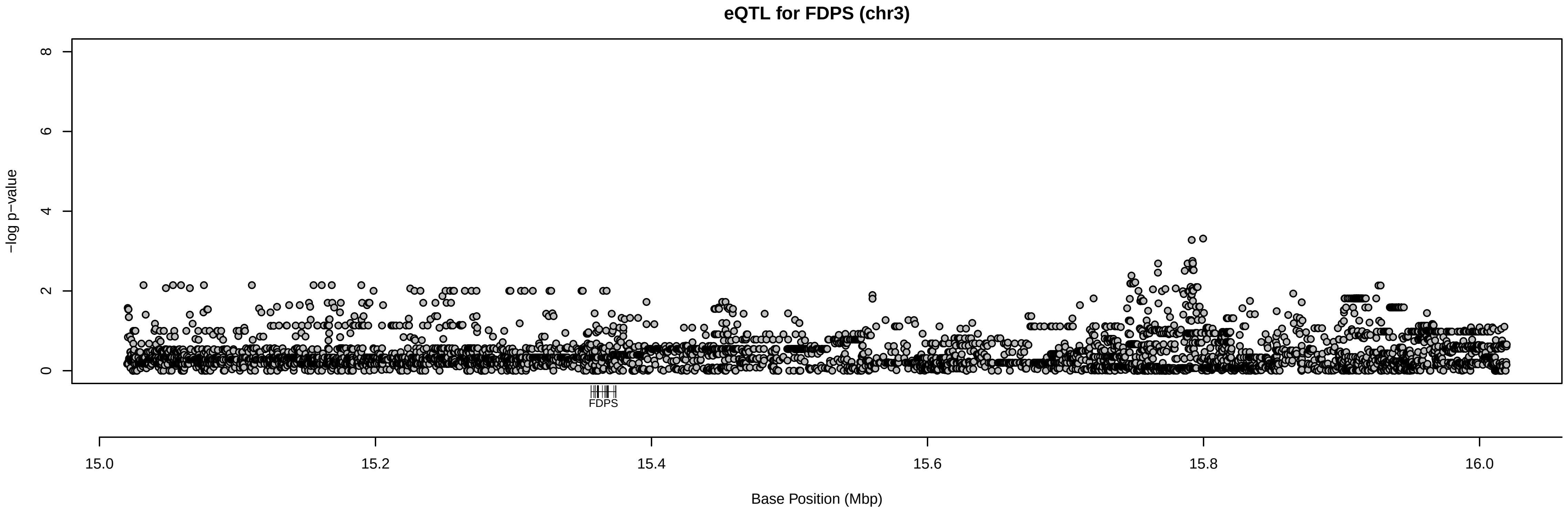
eQTL for FDPS (chr3)
−log p−value
0
2
4
6
8
FDPS
15.0
15.2
15.4
15.6
15.8
16.0
Base Position (Mbp)

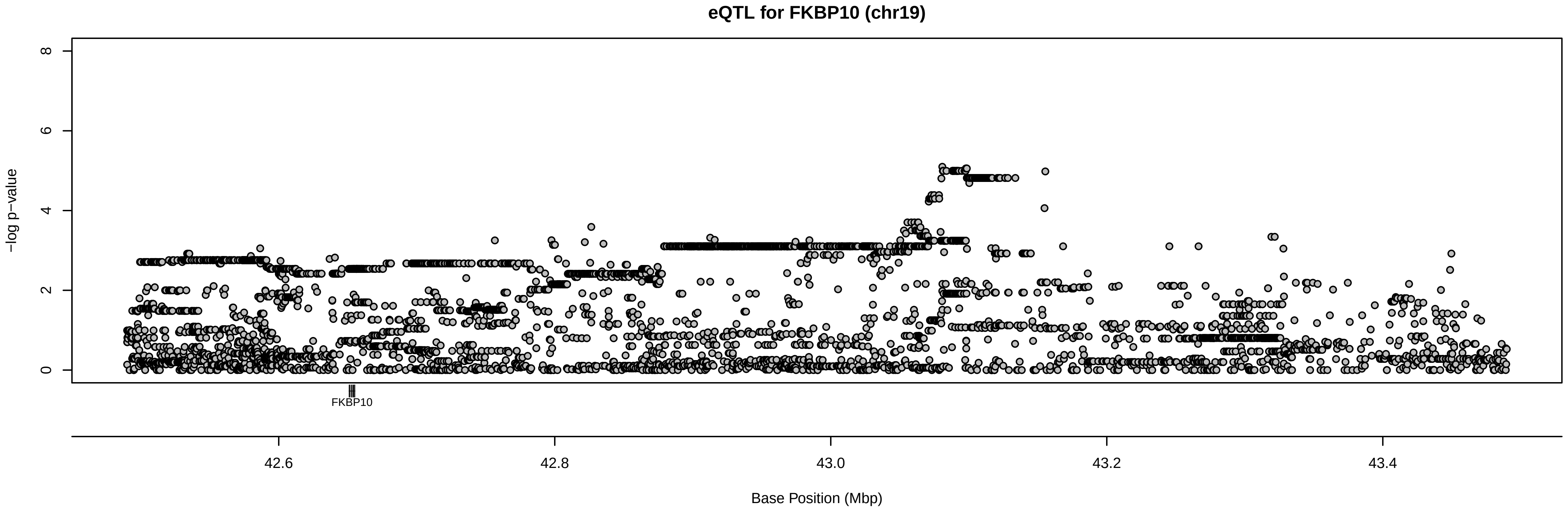
eQTL for FKBP10 (chr19)
−log p−value
0
2
4
6
8
FKBP10
42.6
42.8
43.0
43.2
43.4
Base Position (Mbp)

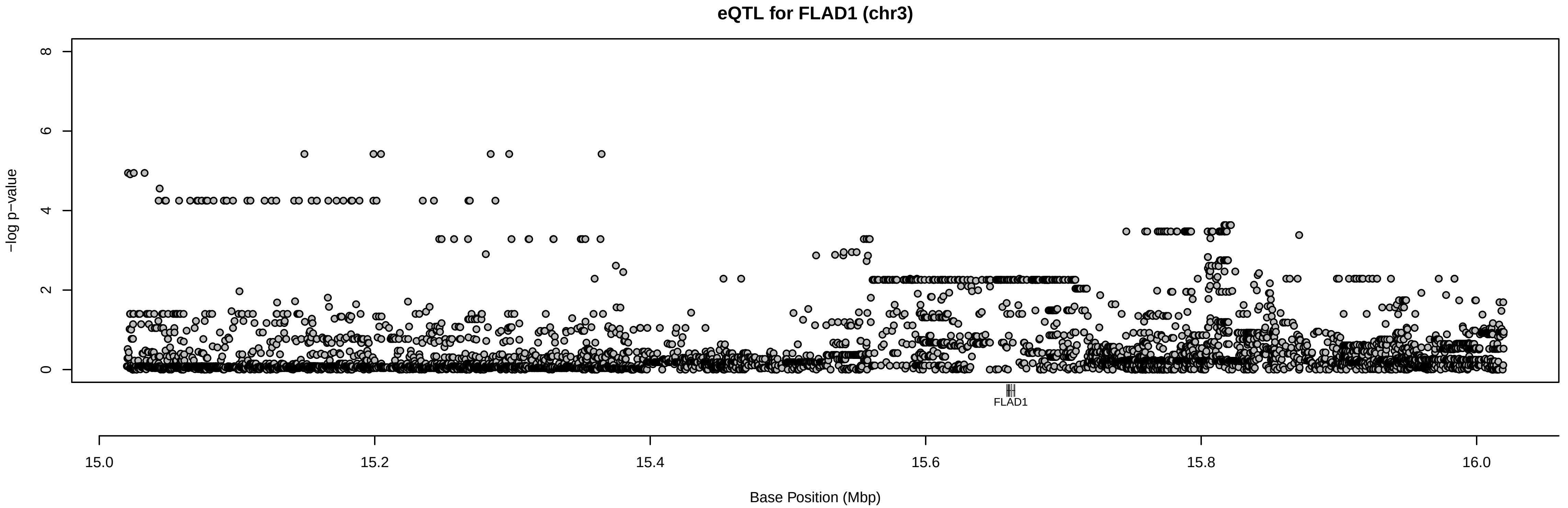

eQTL for FLAD1 (chr3)
−log p−value
0
2
4
6
8
FLAD1
15.0
15.2
15.4
15.6
15.8
16.0
Base Position (Mbp)

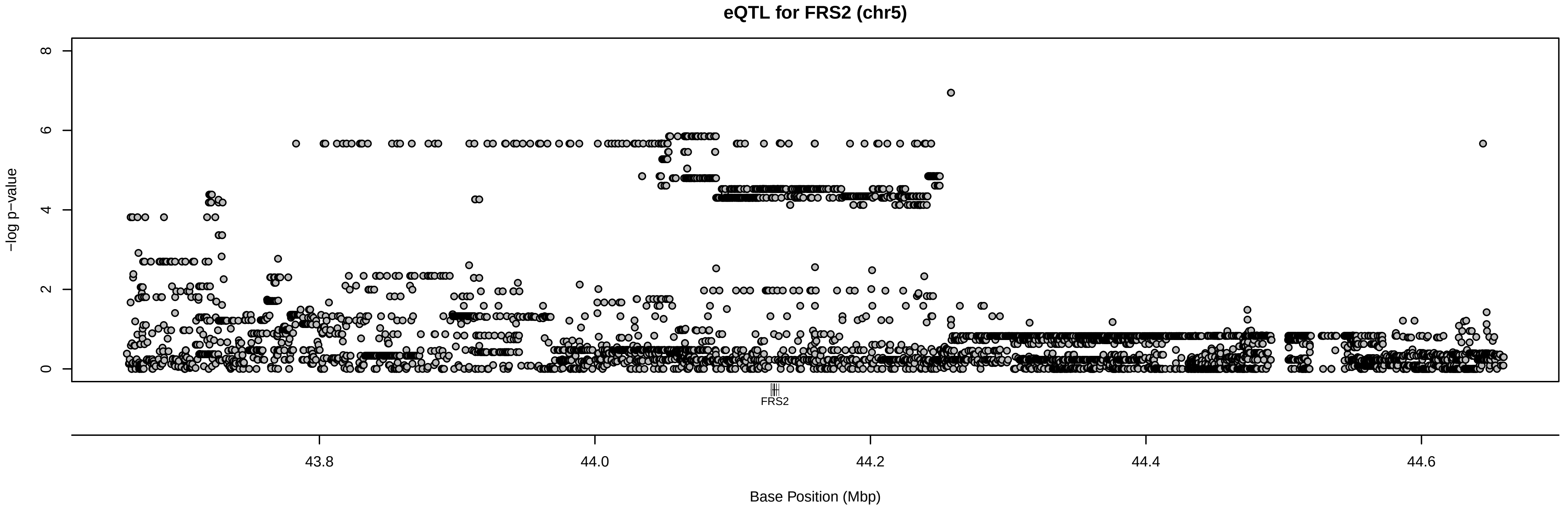

eQTL for FRS2 (chr5)
−log p−value
0
2
4
6
8
FRS2
43.8
44.0
44.2
44.4
44.6
Base Position (Mbp)

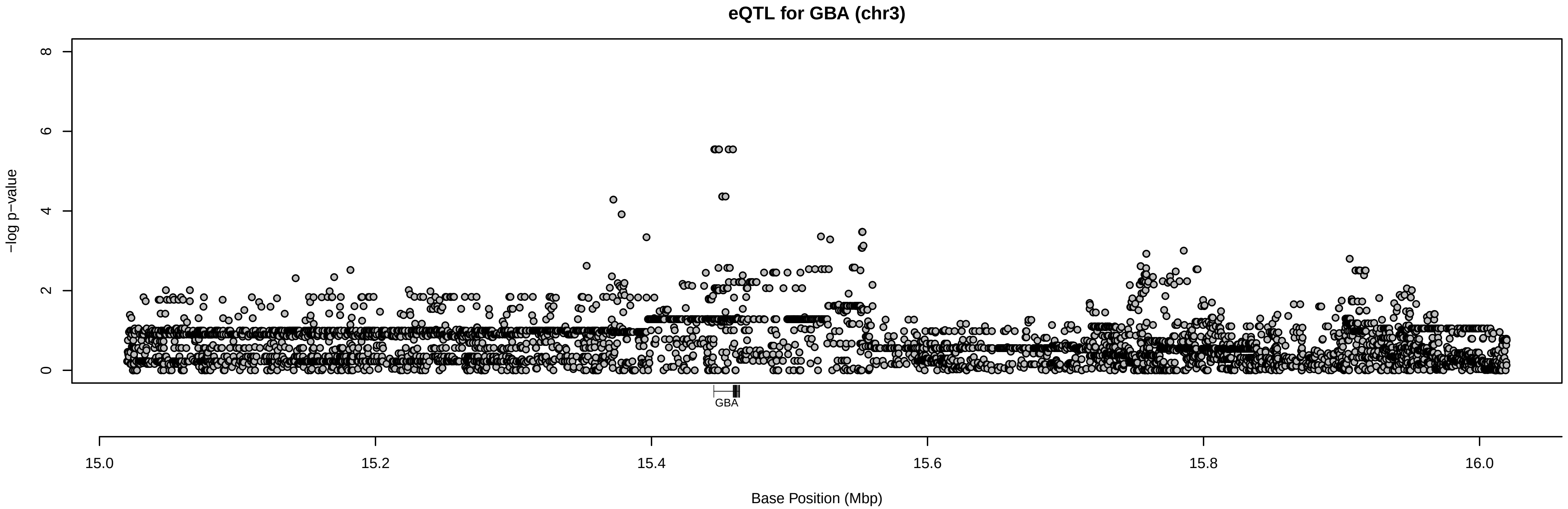
eQTL for GBA (chr3)
−log p−value
0
2
4
6
8
GBA
15.0
15.2
15.4
15.6
15.8
16.0
Base Position (Mbp)

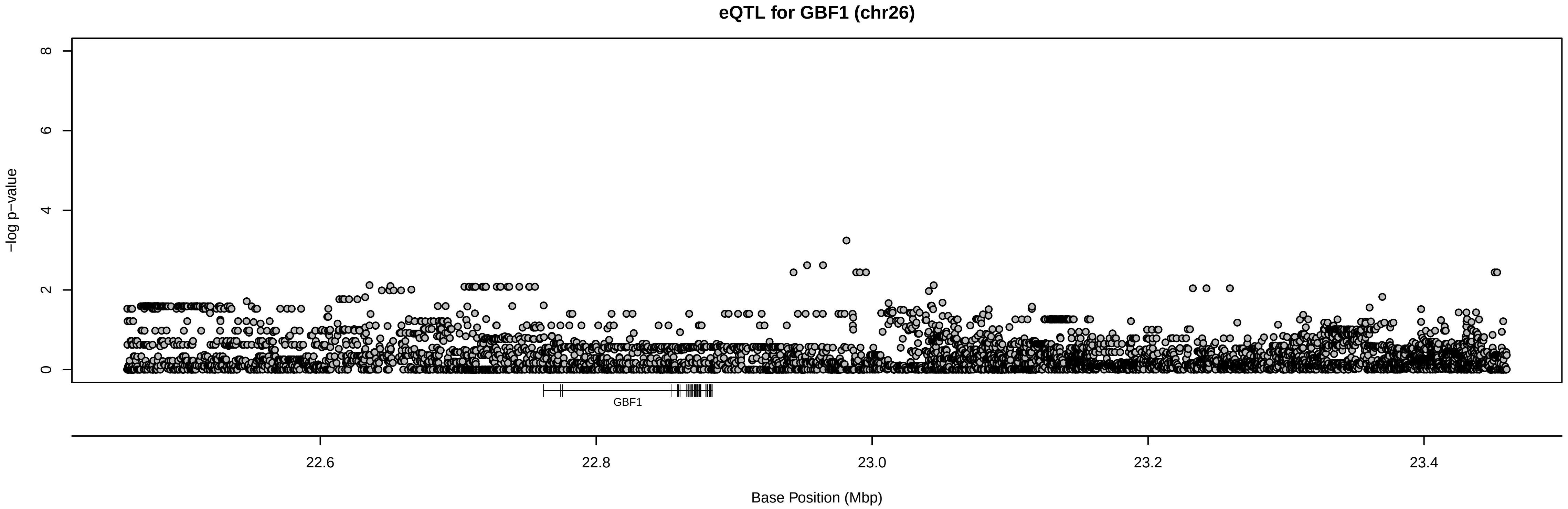
eQTL for GBF1 (chr26)
−log p−value
0
2
4
6
8
GBF1
22.6
22.8
23.0
23.2
23.4
Base Position (Mbp)

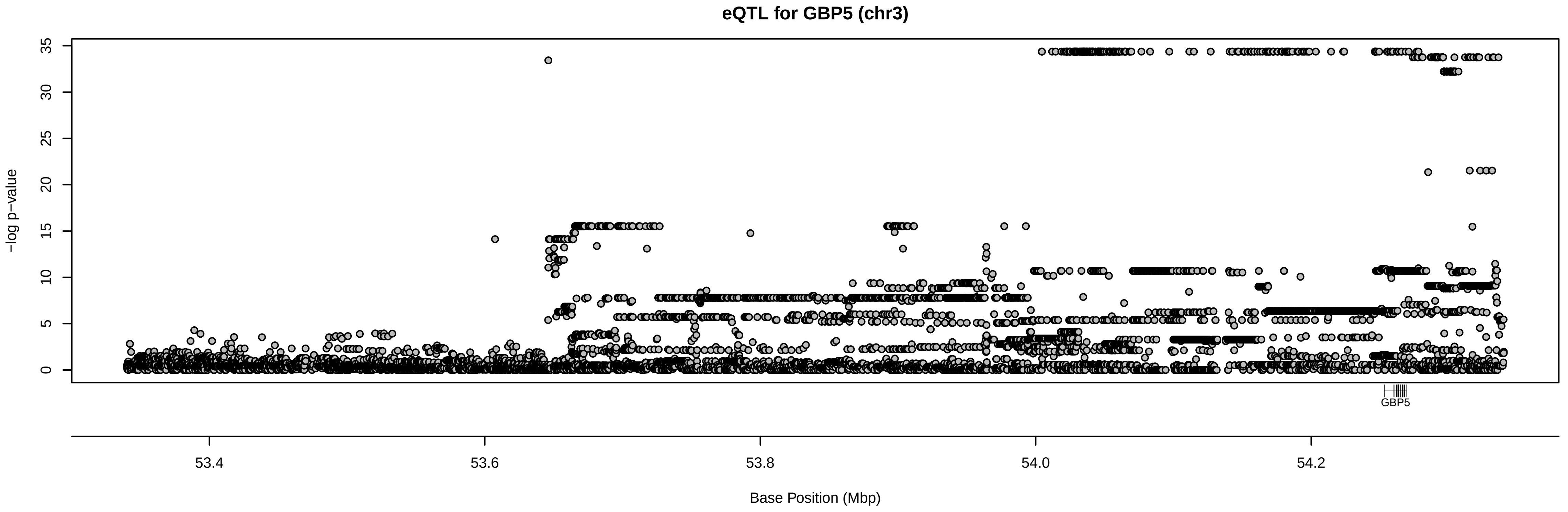
eQTL for GBP5 (chr3)
−log p−value
0
5
10
15
20
25
30
35
GBP5
53.4
53.6
53.8
54.0
54.2
Base Position (Mbp)

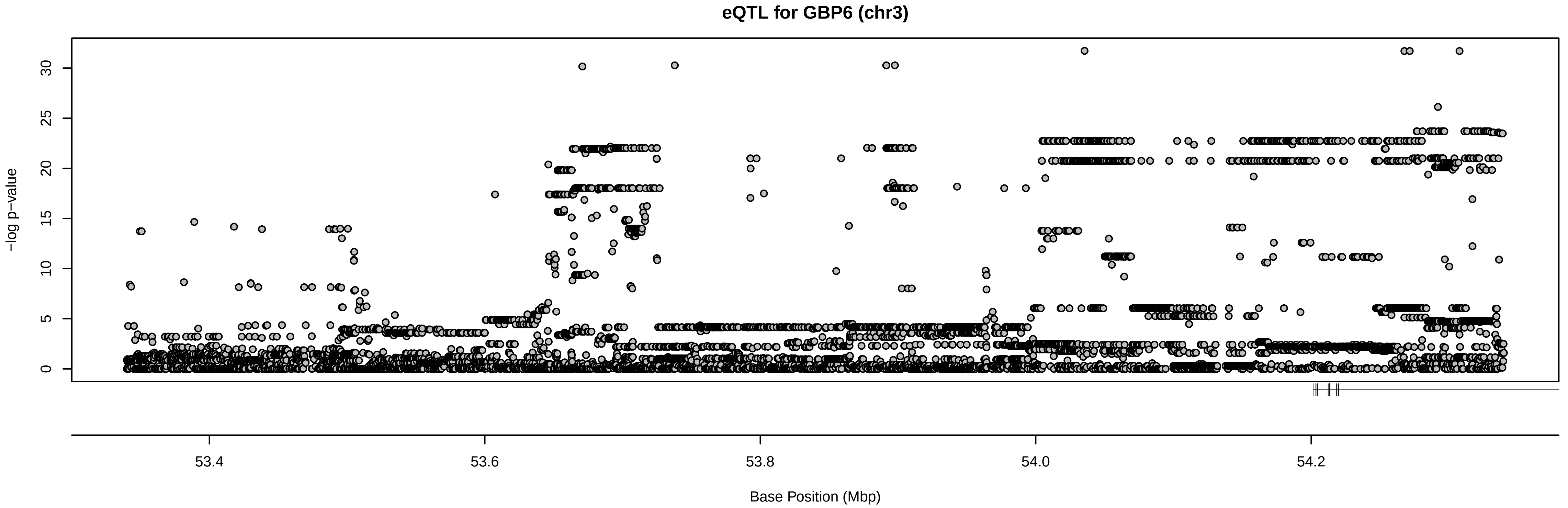
eQTL for GBP6 (chr3)
−log p−value
0
5
10
15
20
25
30
53.4
53.6
53.8
54.0
54.2
Base Position (Mbp)

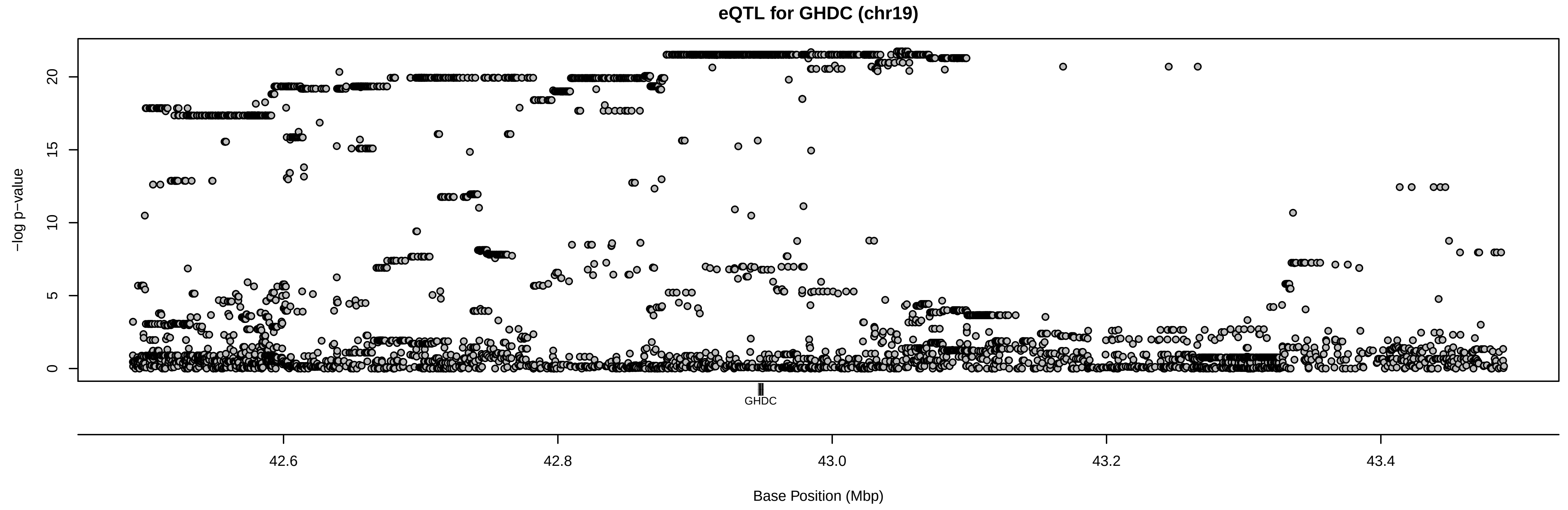
eQTL for GHDC (chr19)
−log p−value
0
5
10
15
20
GHDC
42.6
42.8
43.0
43.2
43.4
Base Position (Mbp)

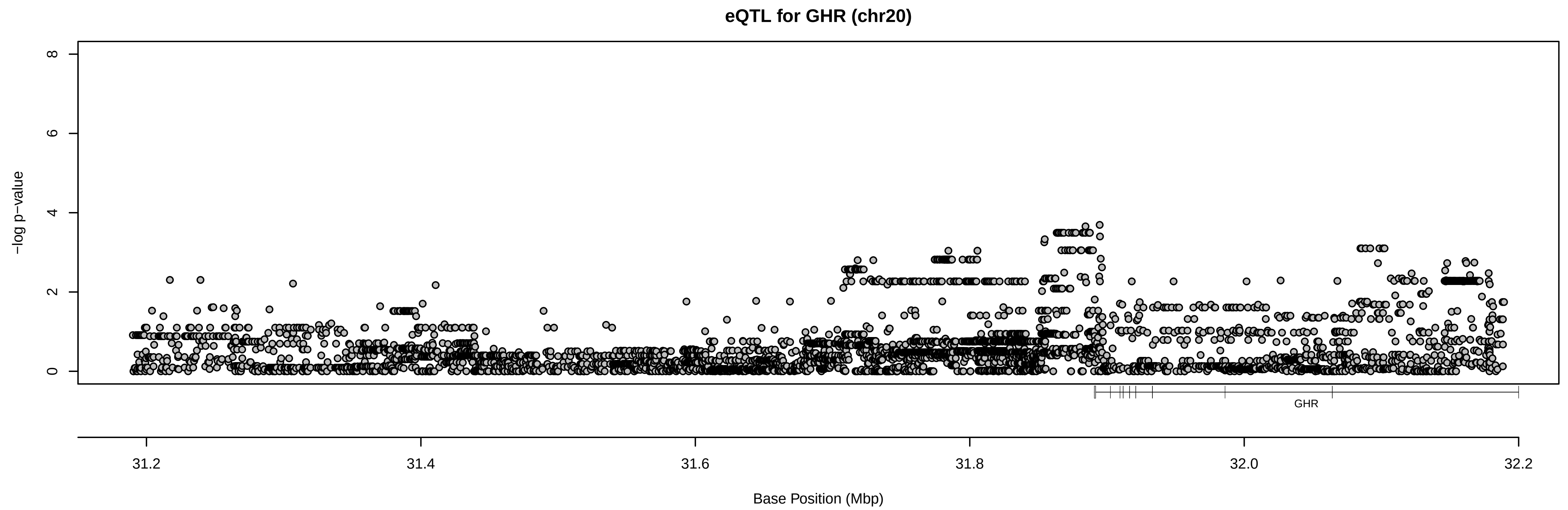
eQTL for GHR (chr20)
−log p−value
0
2
4
6
8
GHR
31.2
31.4
31.6
31.8
32.0
32.2
Base Position (Mbp)

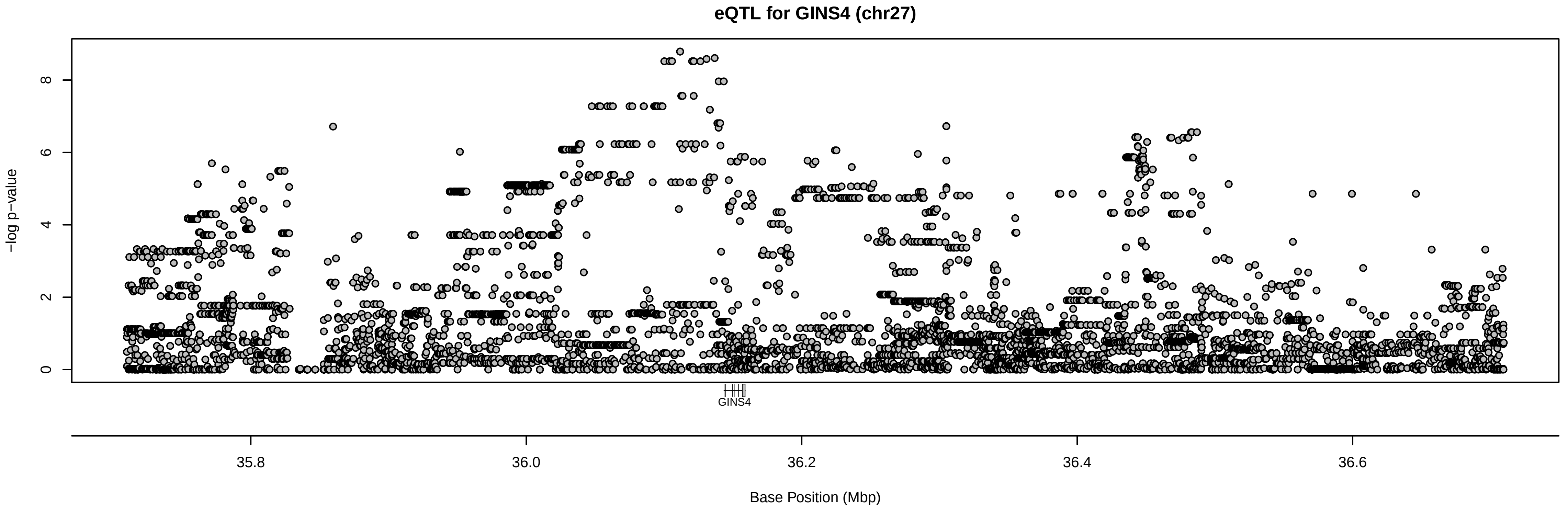
eQTL for GINS4 (chr27)
−log p−value
0
2
4
6
8
GINS4
35.8
36.0
36.2
36.4
36.6
Base Position (Mbp)

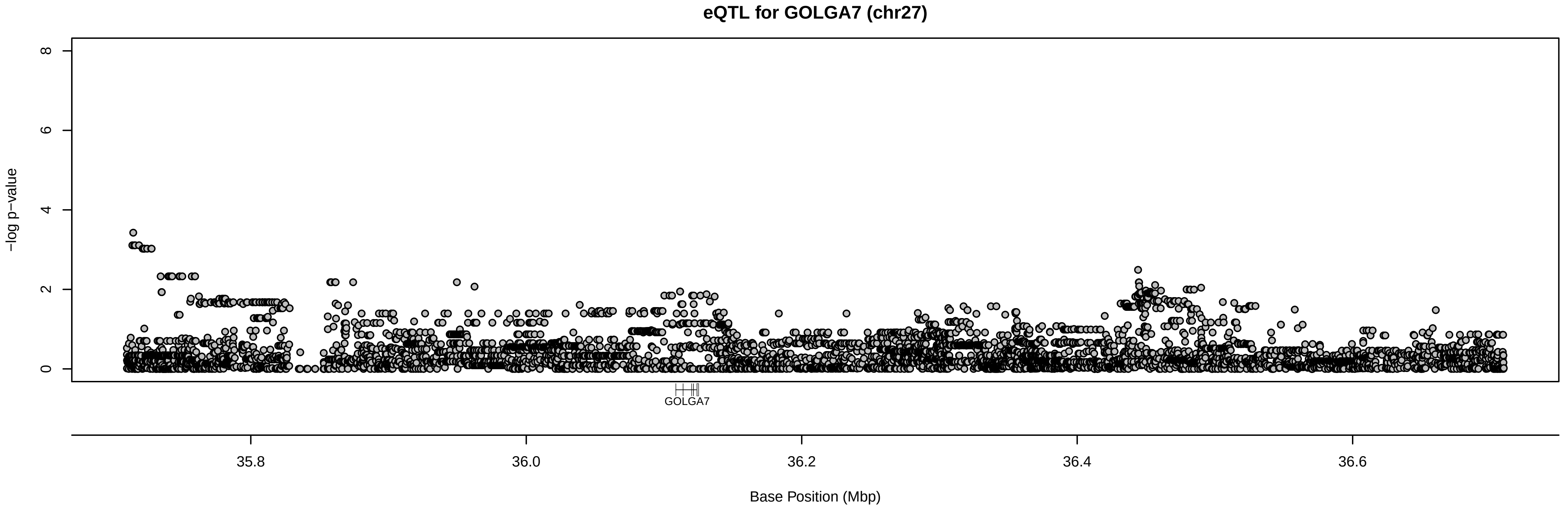
eQTL for GOLGA7 (chr27)
−log p−value
0
2
4
6
8
GOLGA7
35.8
36.0
36.2
36.4
36.6
Base Position (Mbp)

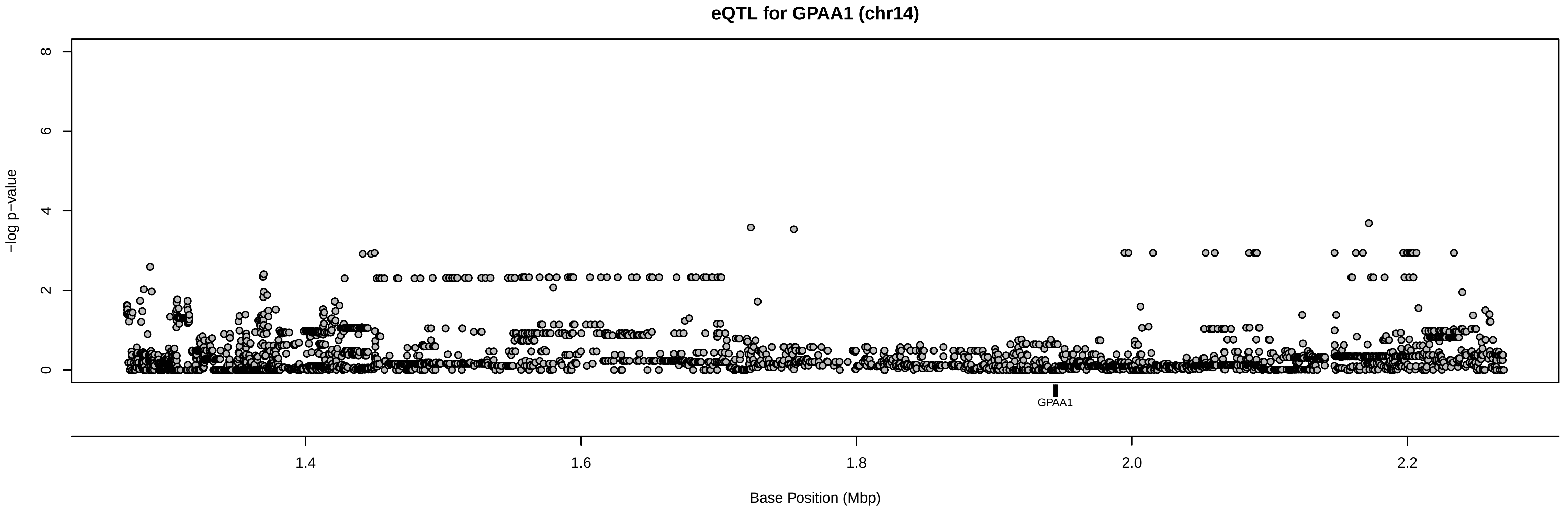
eQTL for GPAA1 (chr14)
−log p−value
0
2
4
6
8
GPAA1
1.4
1.6
1.8
2.0
2.2
Base Position (Mbp)

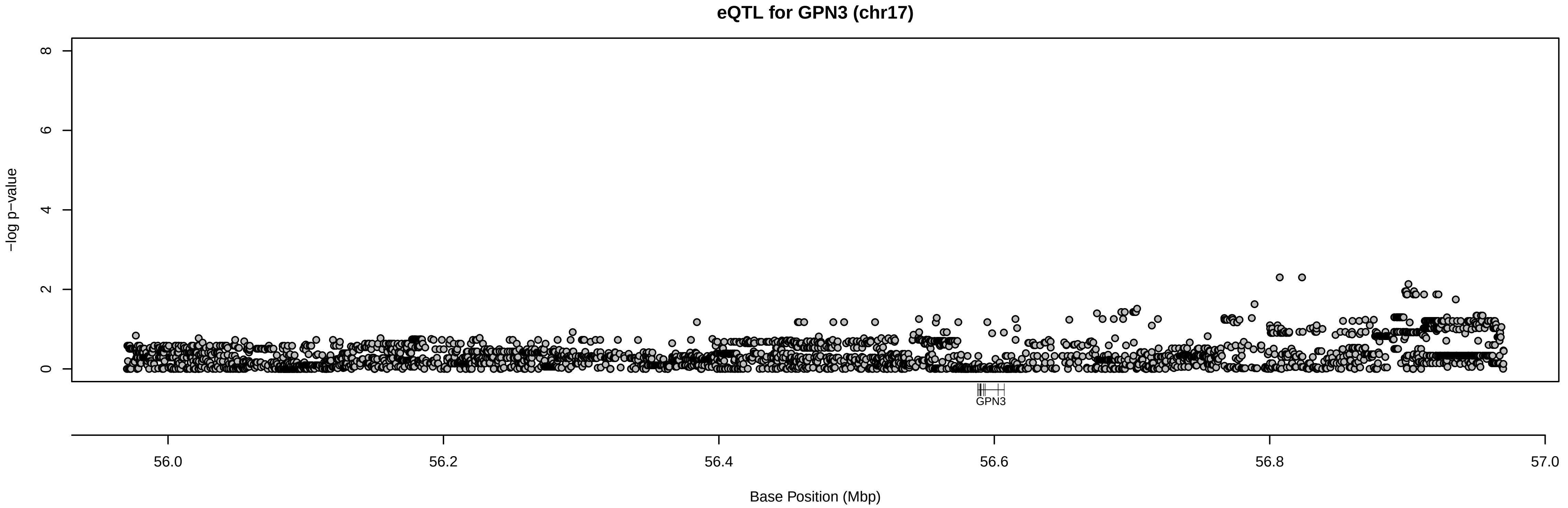
eQTL for GPN3 (chr17)
−log p−value
0
2
4
6
8
GPN3
56.0
56.2
56.4
56.6
56.8
57.0
Base Position (Mbp)

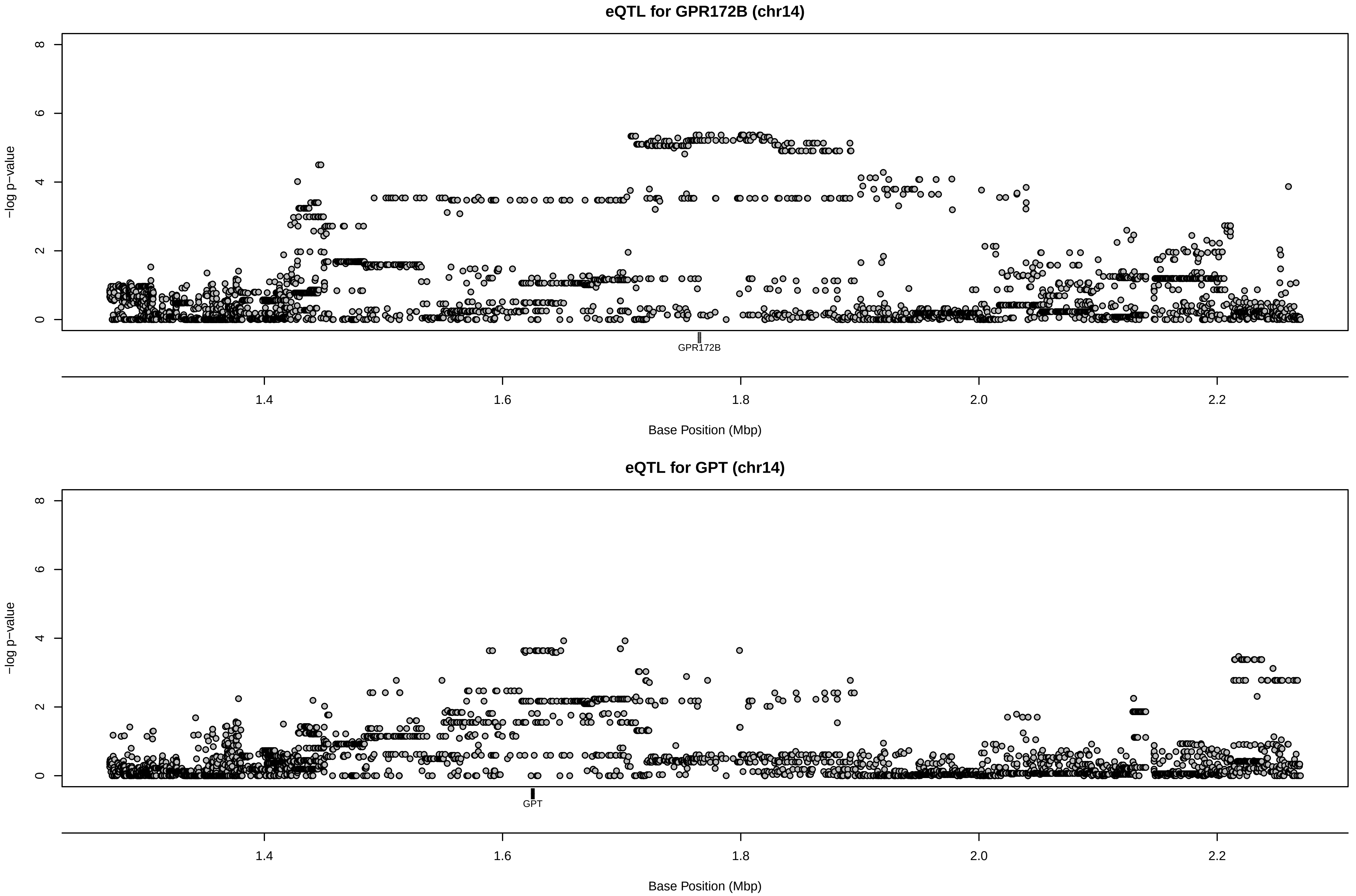
eQTL for GPR172B (chr14)
−log p−value
GPR172B
Base Position (Mbp)
eQTL for GPT (chr14)
−log p−value
GPT
Base Position (Mbp)

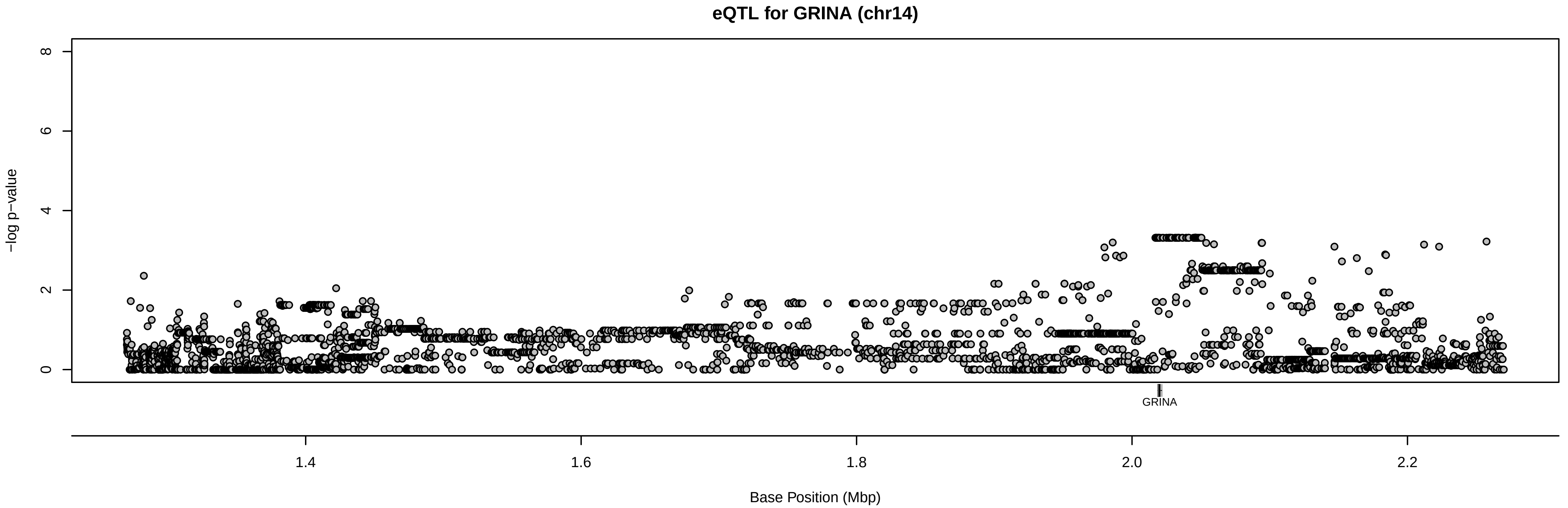
eQTL for GRINA (chr14)
−log p−value
0
2
4
6
8
GRINA
1.4
1.6
1.8
2.0
2.2
Base Position (Mbp)

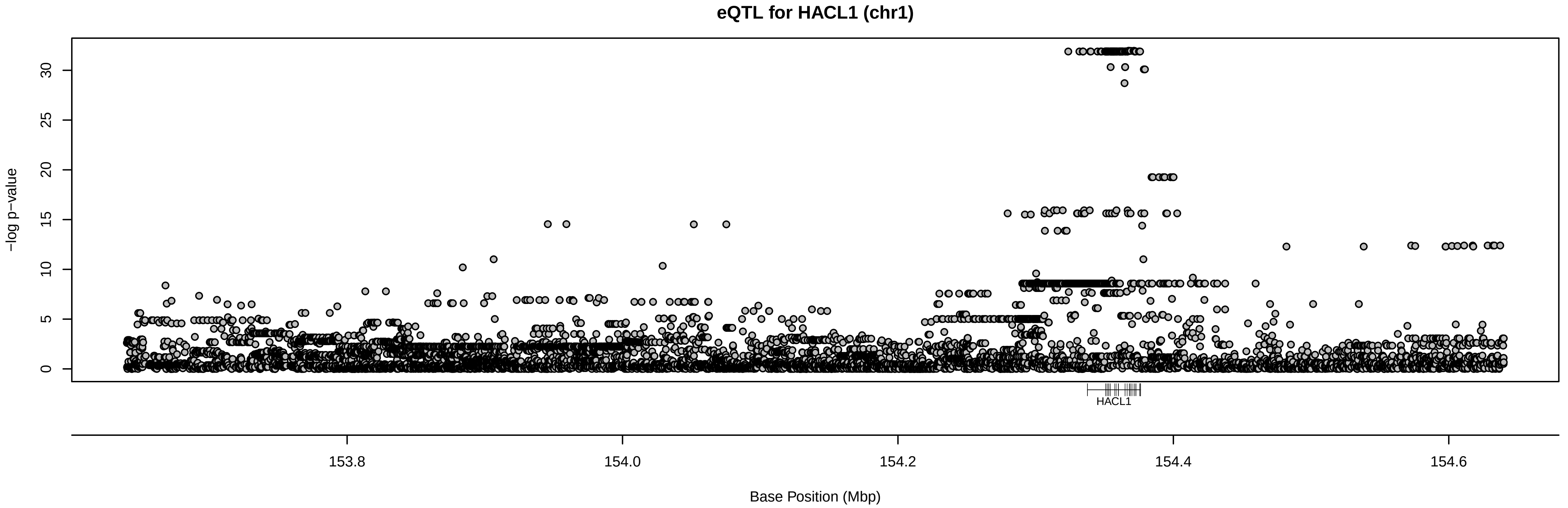
eQTL for HACL1 (chr1)
−log p−value
0
5
10
15
20
25
30
HACL1
153.8
154.0
154.2
154.4
154.6
Base Position (Mbp)

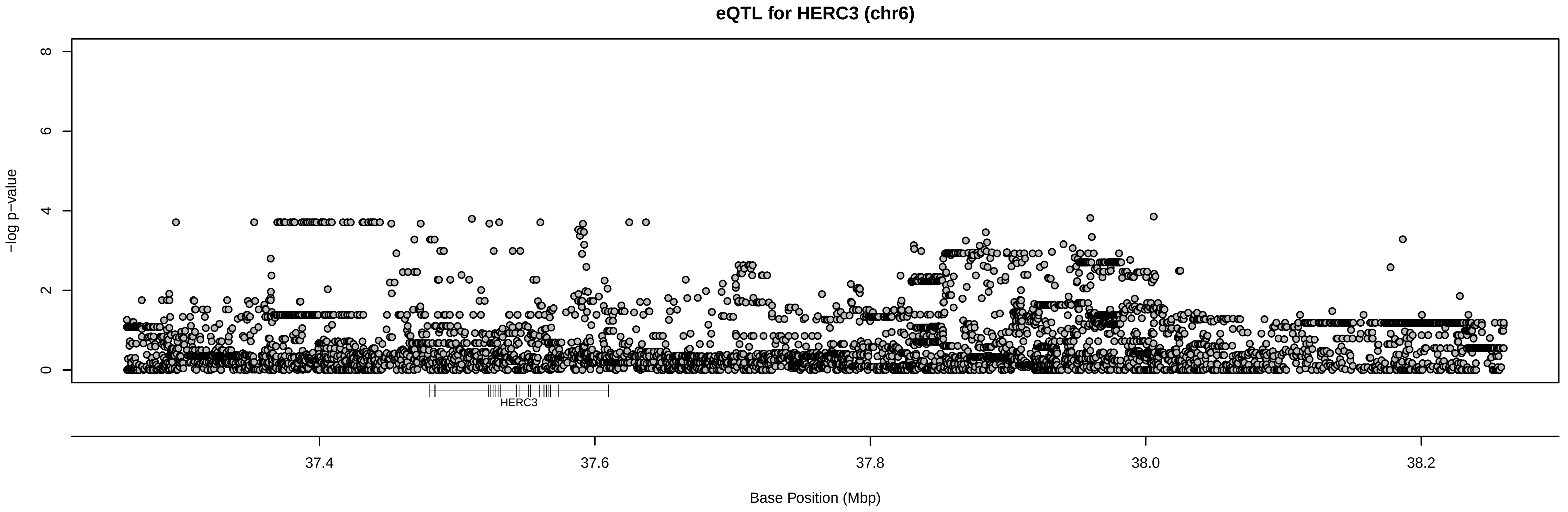

eQTL for HERC3 (chr6)
−log p−value
0
2
4
6
8
HERC3
37.4
37.6
37.8
38.0
38.2
Base Position (Mbp)

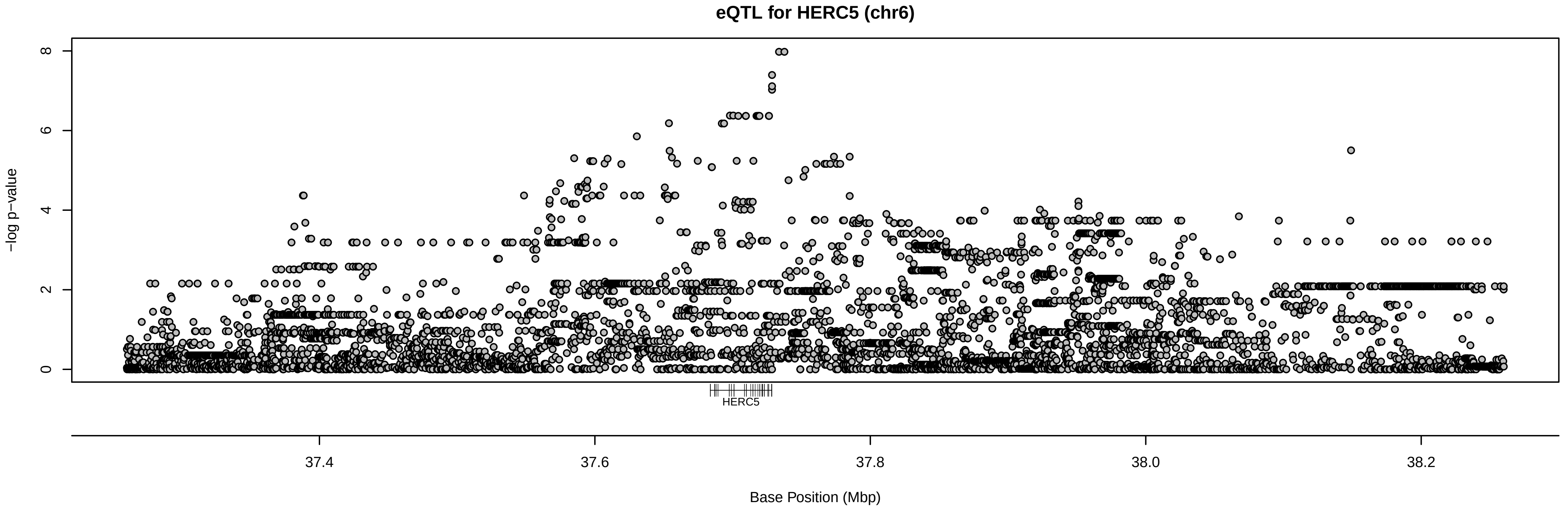

eQTL for HERC5 (chr6)
−log p−value
0
2
4
6
8
HERC5
37.4
37.6
37.8
38.0
38.2
Base Position (Mbp)

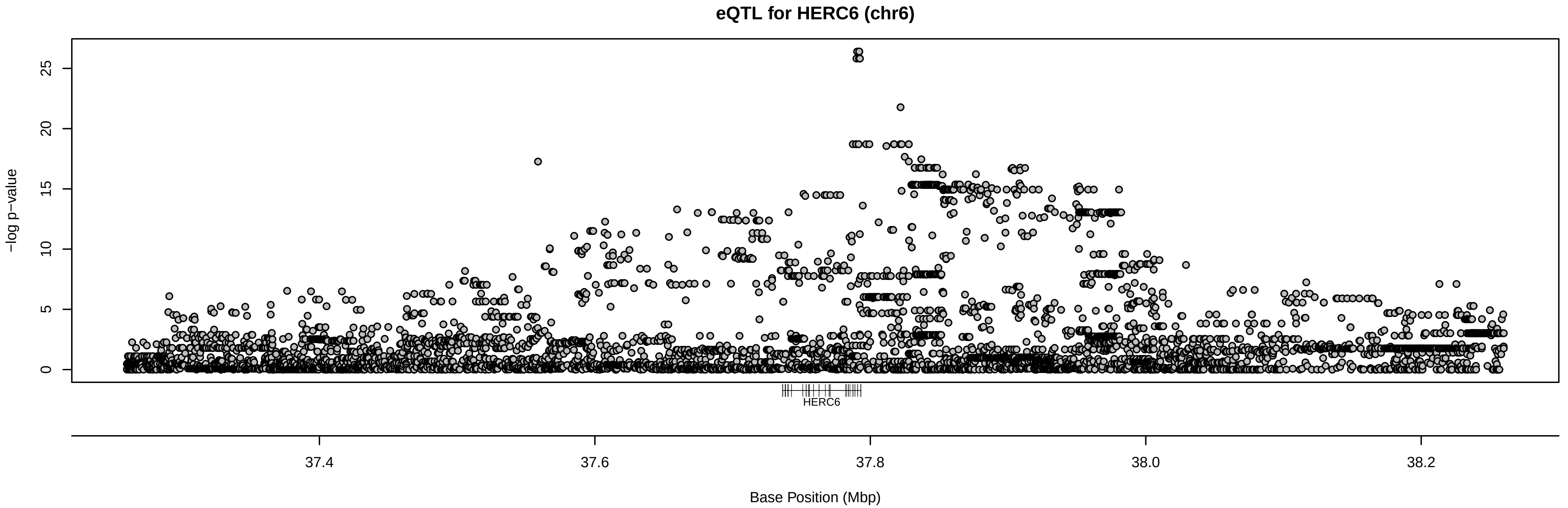

eQTL for HERC6 (chr6)
−log p−value
HERC6
Base Position (Mbp)

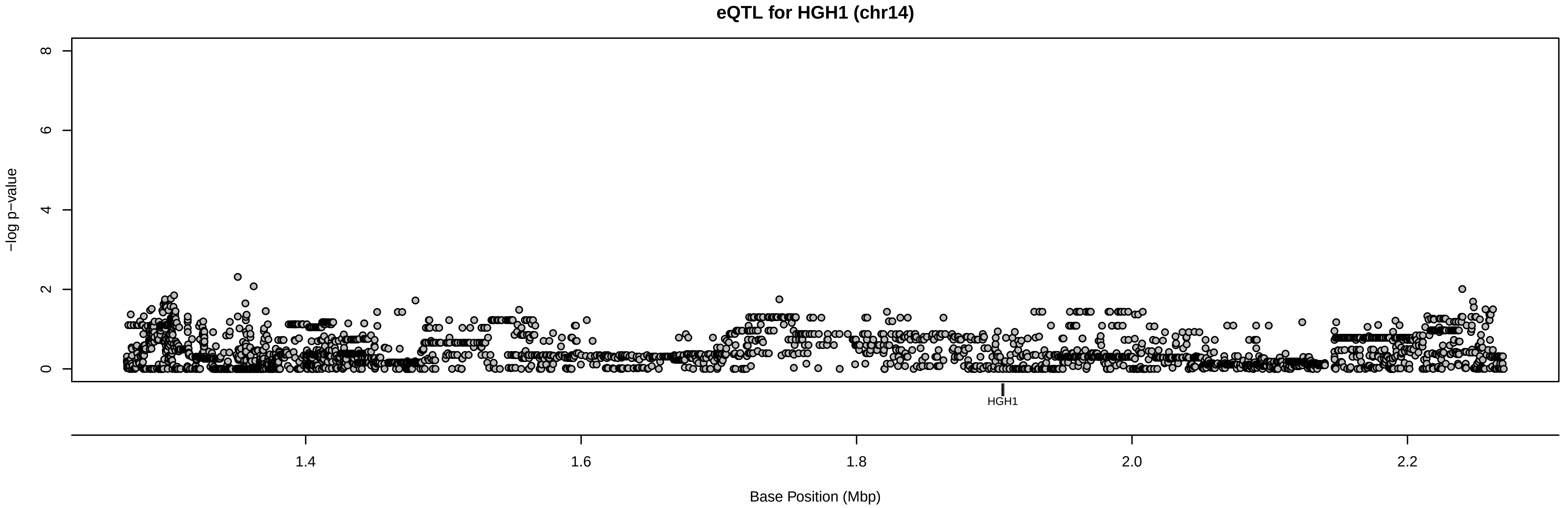

eQTL for HGH1 (chr14)
−log p−value
HGH1
Base Position (Mbp)

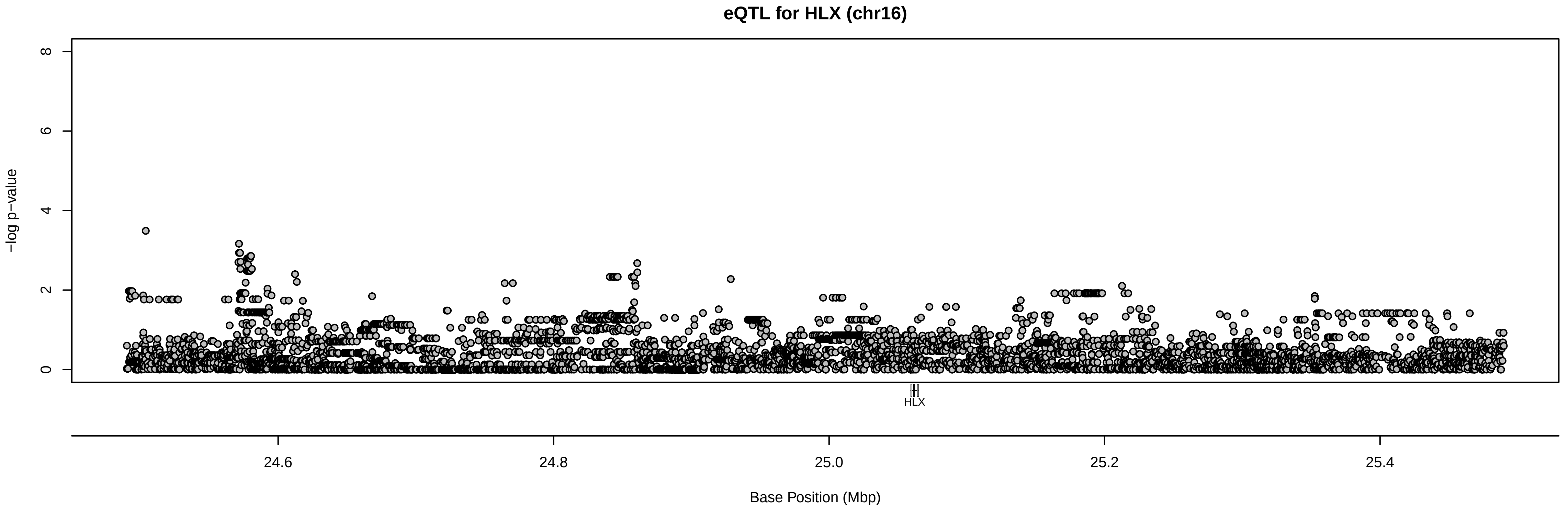
eQTL for HLX (chr16)
−log p−value
0
2
4
6
8
HLX
24.6
24.8
25.0
25.2
25.4
Base Position (Mbp)

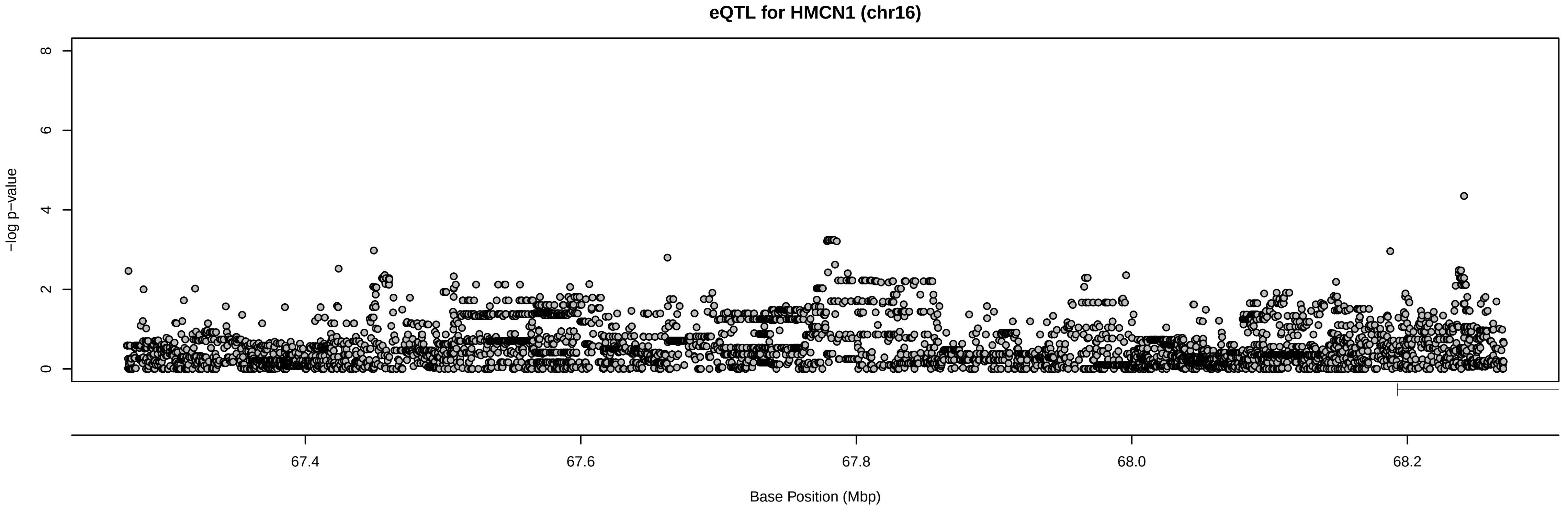
eQTL for HMCN1 (chr16)
−log p−value
0
2
4
6
8
67.4
67.6
67.8
68.0
68.2
Base Position (Mbp)

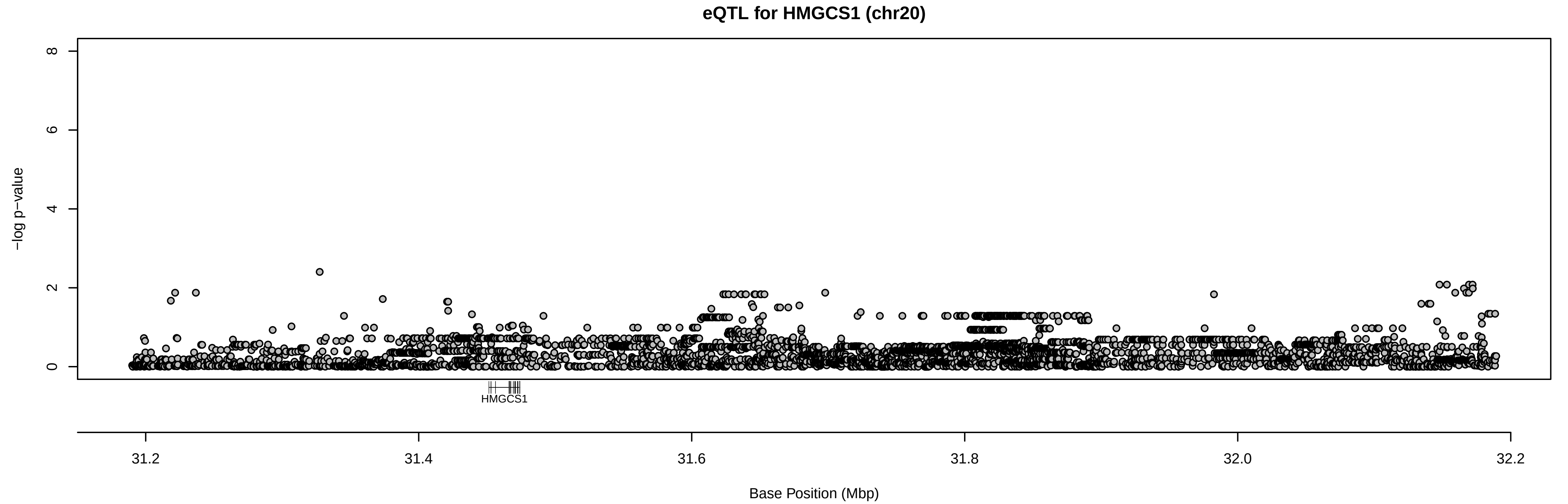

eQTL for HMGCS1 (chr20)
−log p−value
0
2
4
6
8
HMGCS1
31.2
31.4
31.6
31.8
32.0
32.2
Base Position (Mbp)

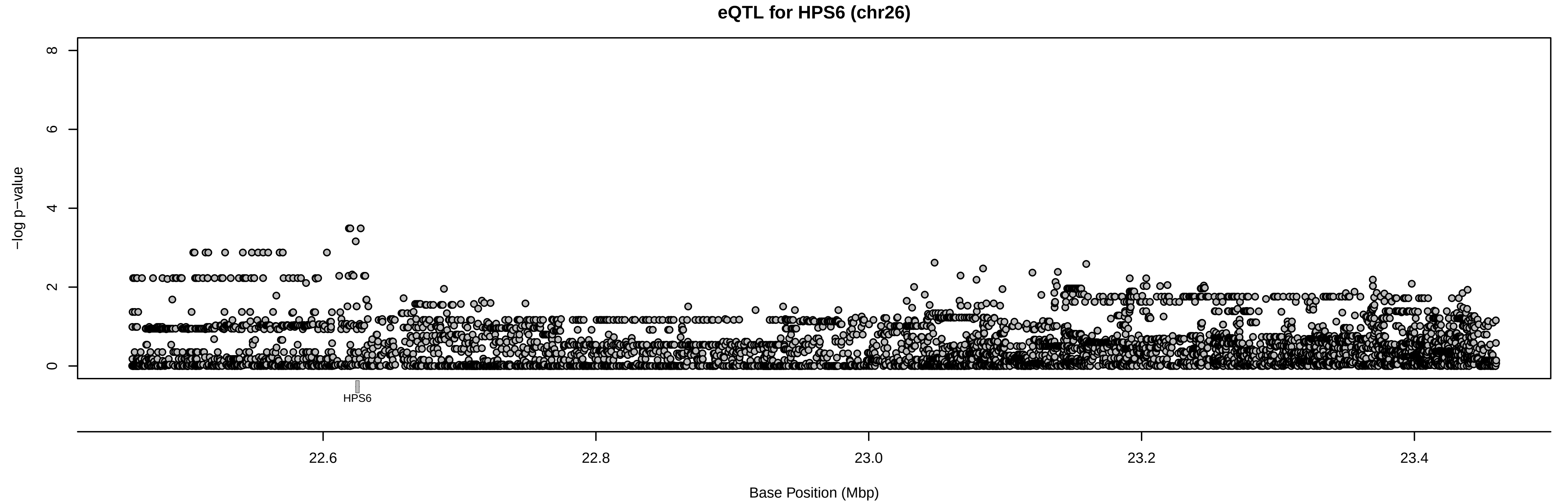

eQTL for HPS6 (chr26)
−log p−value
0
2
4
6
8
HPS6
22.6
22.8
23.0
23.2
23.4
Base Position (Mbp)

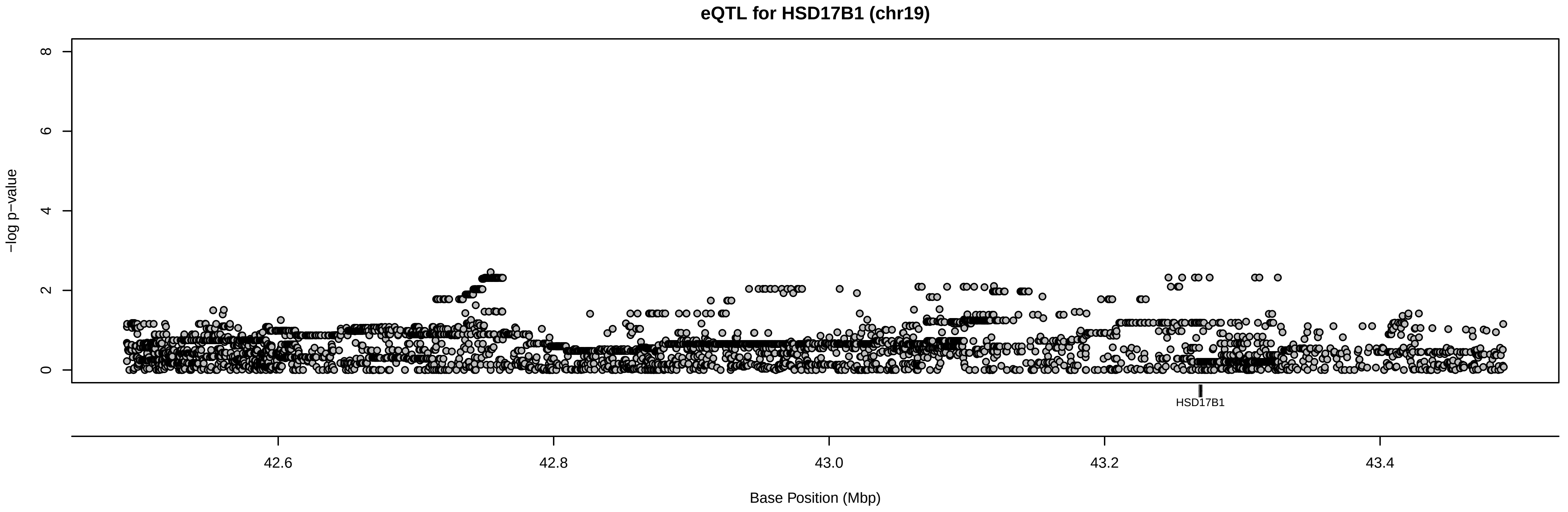
eQTL for HSD17B1 (chr19)
−log p−value
0
2
4
6
8
HSD17B1
42.6
42.8
43.0
43.2
43.4
Base Position (Mbp)

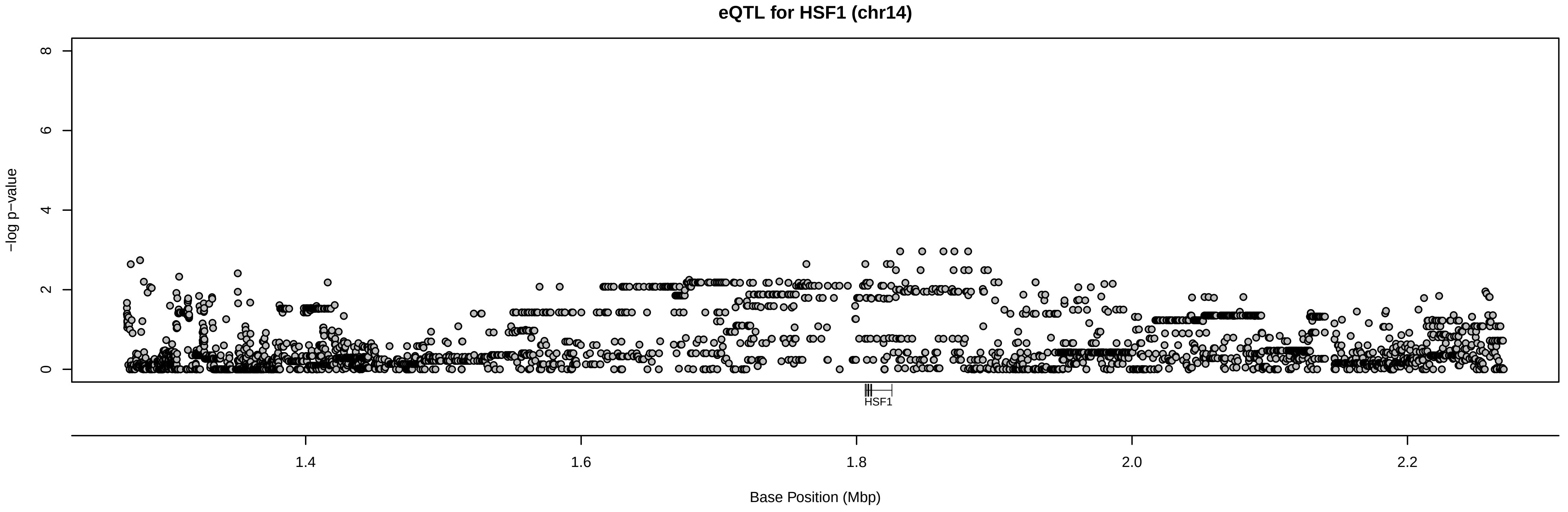
eQTL for HSF1 (chr14)
−log p−value
0
2
4
6
8
HSF1
1.4
1.6
1.8
2.0
2.2
Base Position (Mbp)

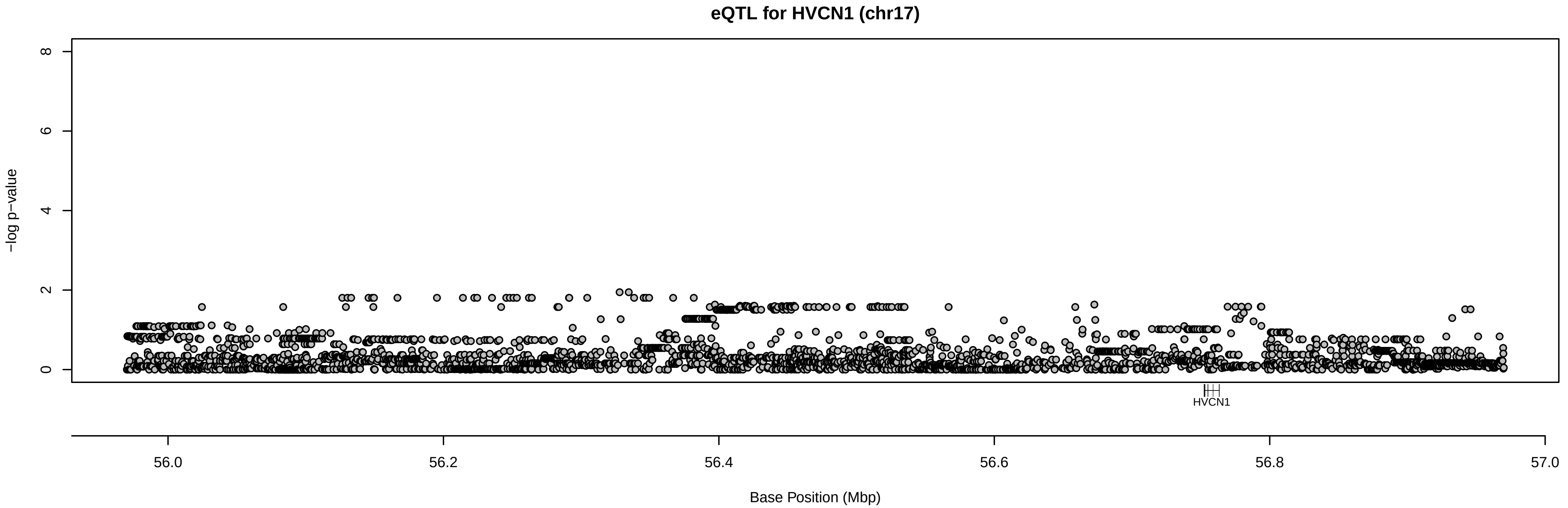
eQTL for HVCN1 (chr17)
−log p−value
0
2
4
6
8
HVCN1
56.0
56.2
56.4
56.6
56.8
57.0
Base Position (Mbp)

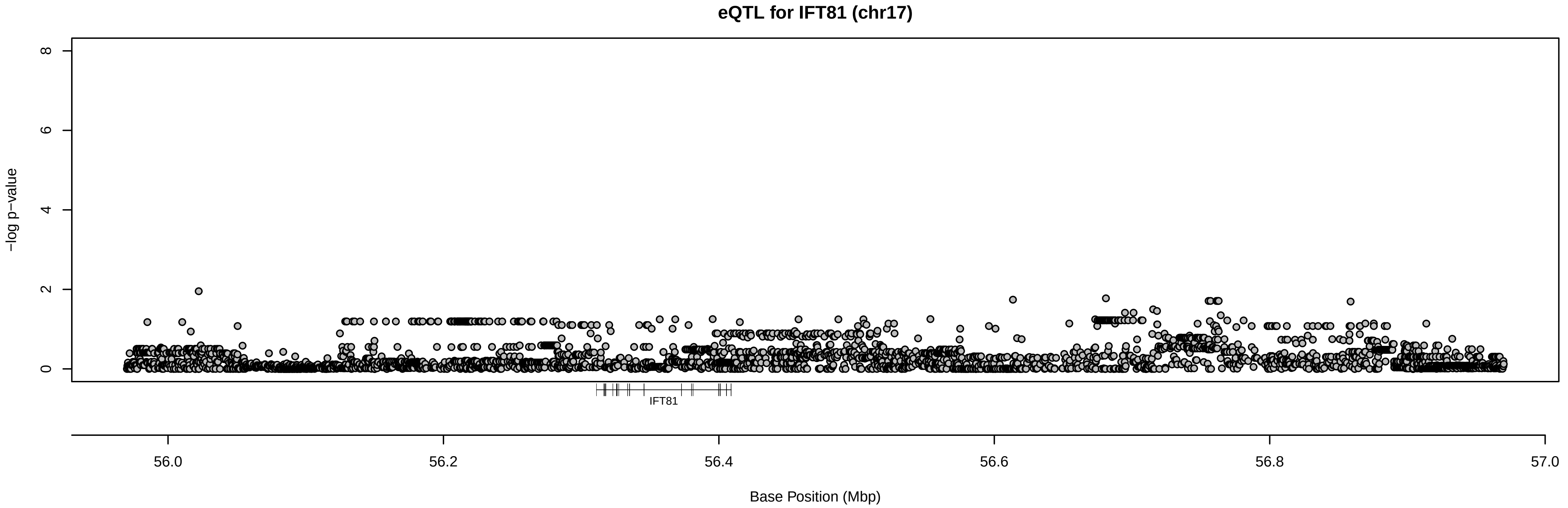
eQTL for IFT81 (chr17)
−log p−value
0
2
4
6
8
IFT81
56.0
56.2
56.4
56.6
56.8
57.0
Base Position (Mbp)

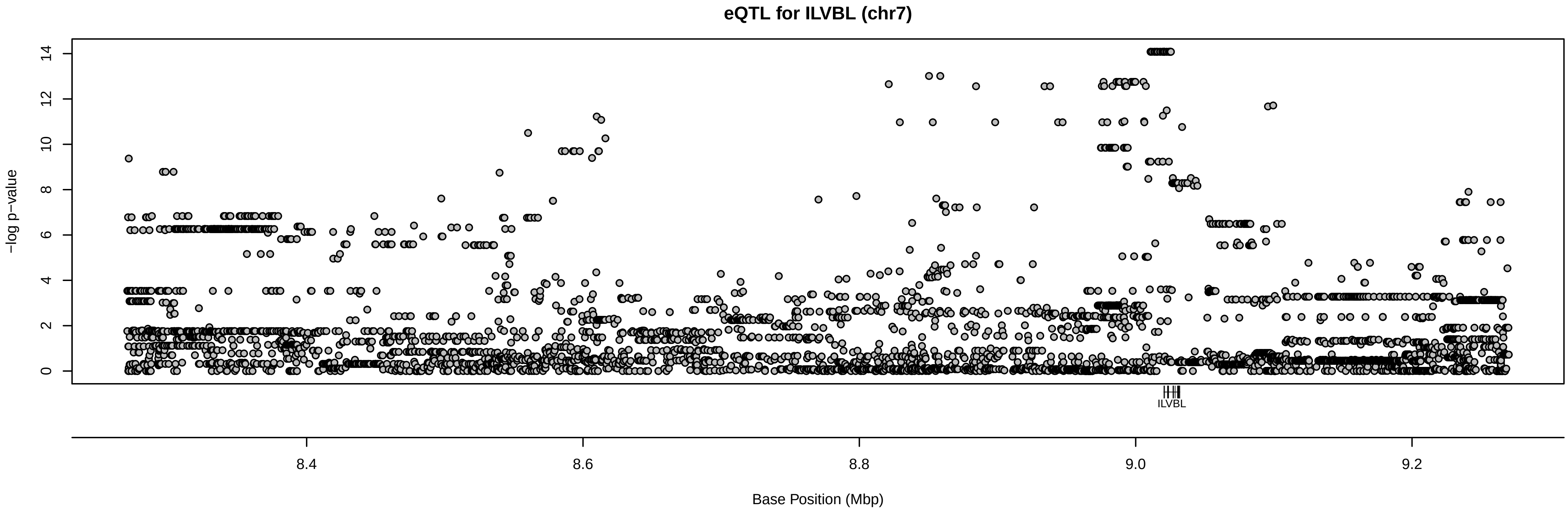
eQTL for ILVBL (chr7)
−log p−value
0
2
4
6
8
10
12
14
ILVBL
8.4
8.6
8.8
9.0
9.2
Base Position (Mbp)

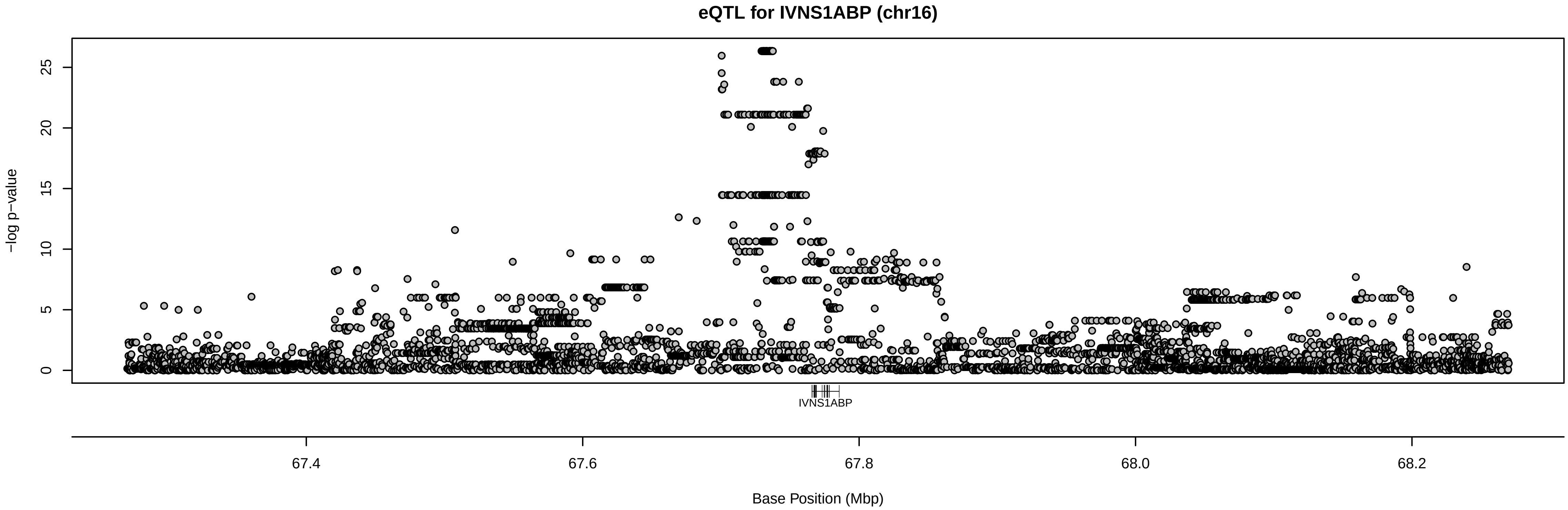
eQTL for IVNS1ABP (chr16)
−log p−value
0
5
10
15
20
25
IVNS1ABP
67.4
67.6
67.8
68.0
68.2
Base Position (Mbp)

eQTL for JUP (chr19)

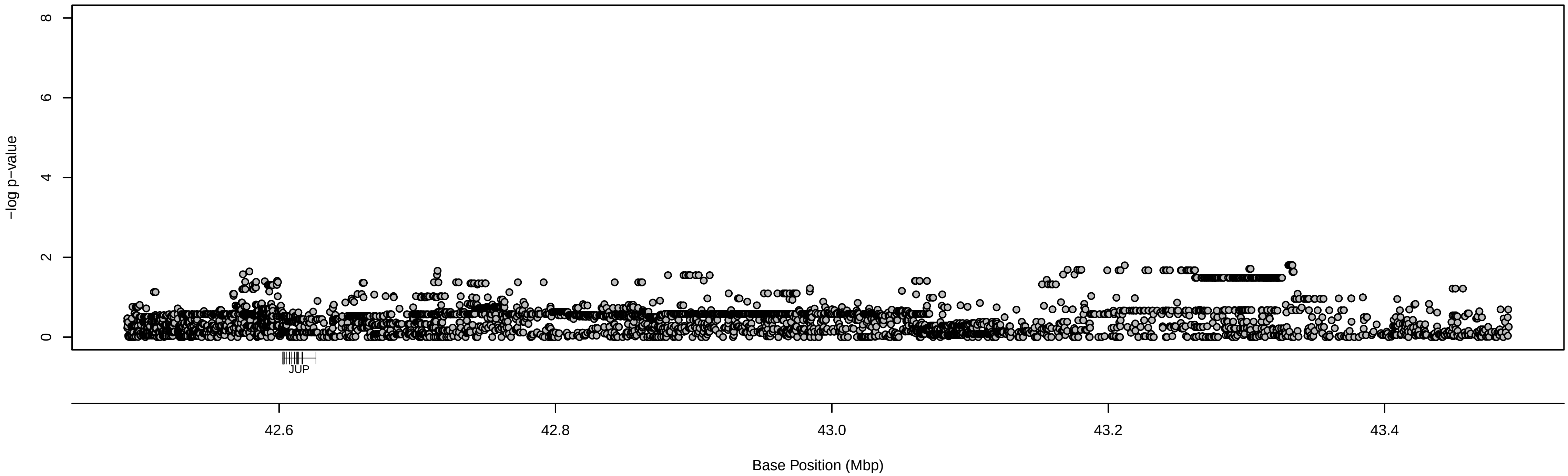

eQTL for KANSL2 (chr5)

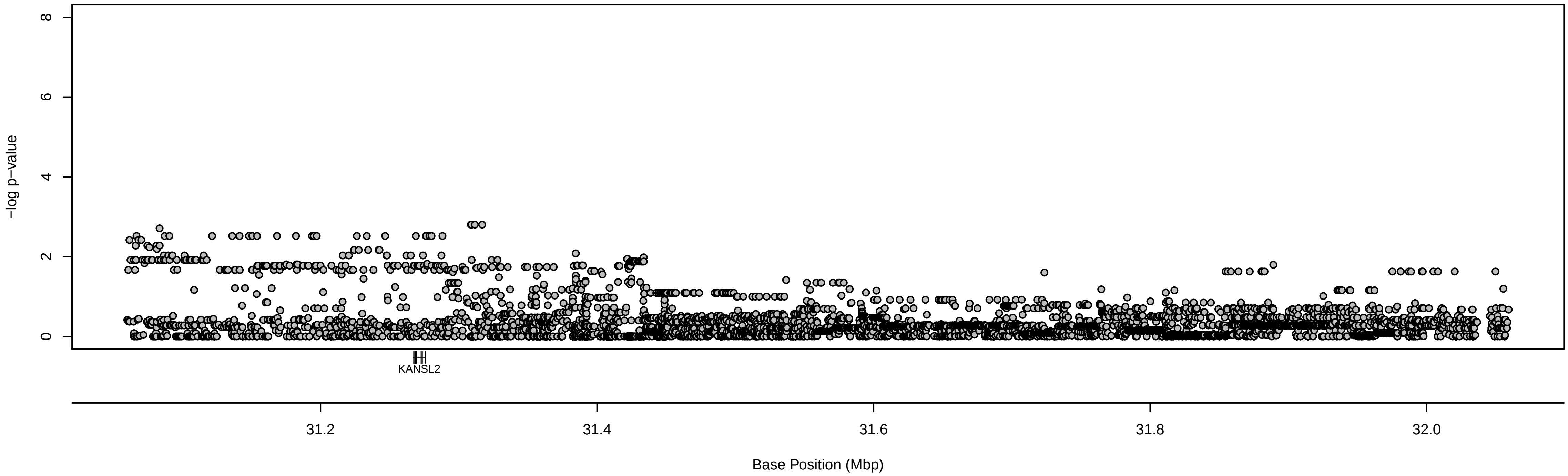

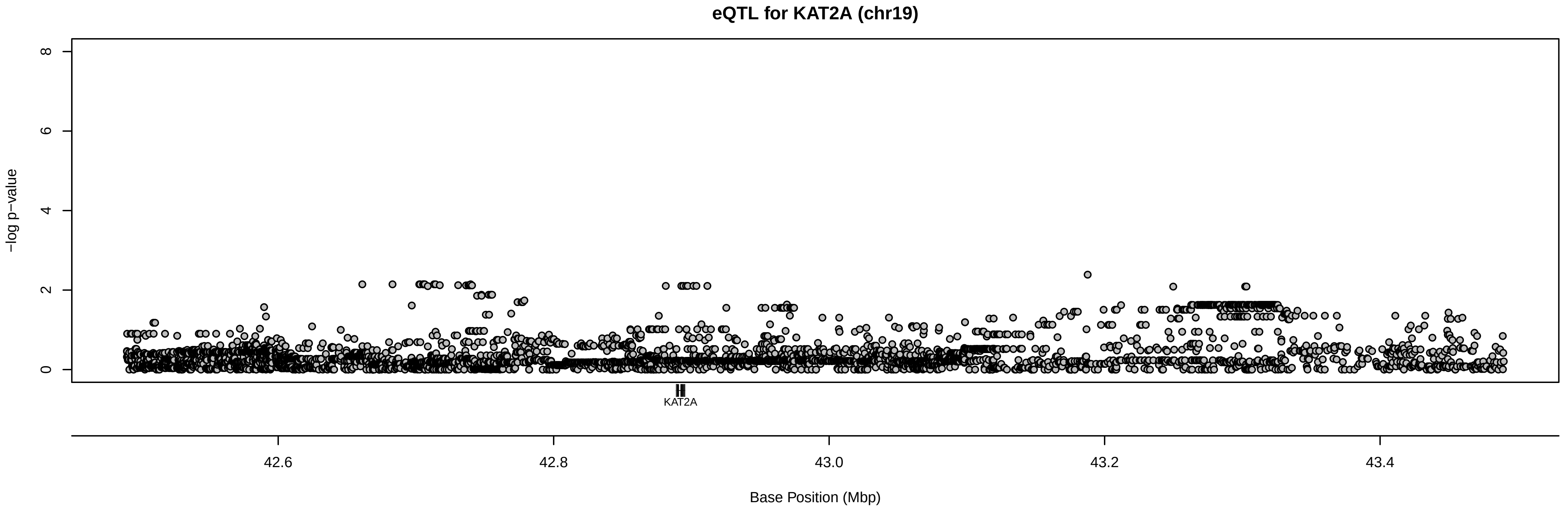
eQTL for KAT2A (chr19)
−log p−value
0
2
4
6
8
KAT2A
42.6
42.8
43.0
43.2
43.4
Base Position (Mbp)

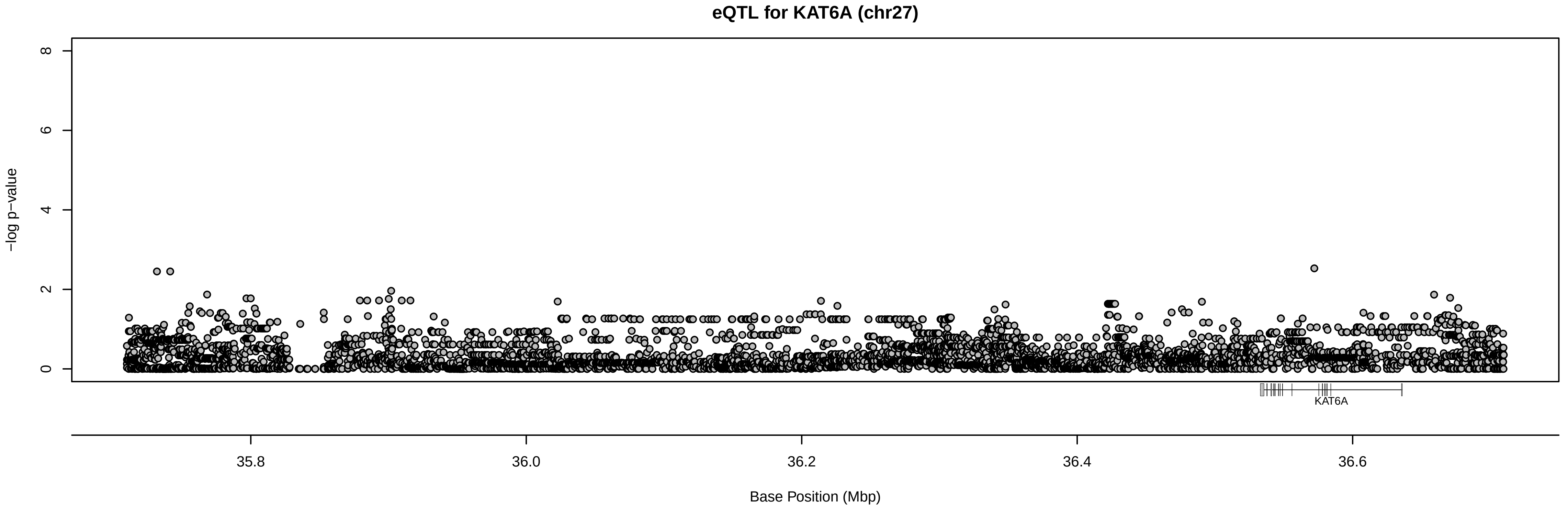
eQTL for KAT6A (chr27)
−log p−value
0
2
4
6
8
KAT6A
35.8
36.0
36.2
36.4
36.6
Base Position (Mbp)

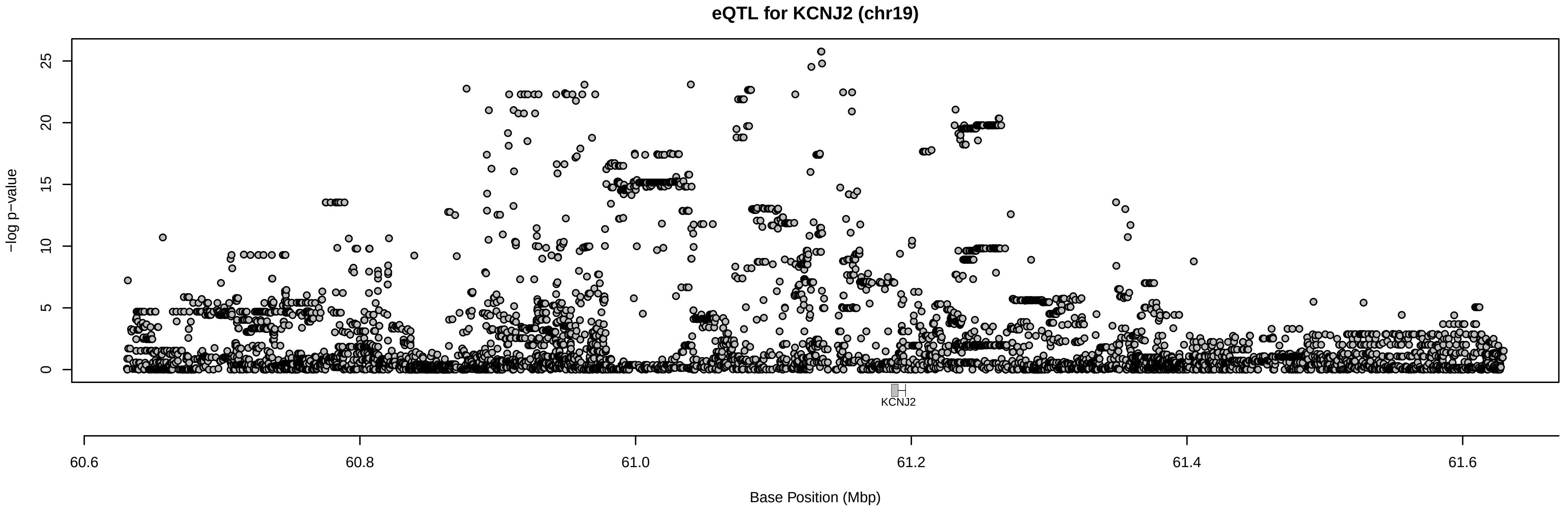
eQTL for KCNJ2 (chr19)
−log p−value
0
5
10
15
20
25
KCNJ2
60.6
60.8
61.0
61.2
61.4
61.6
Base Position (Mbp)

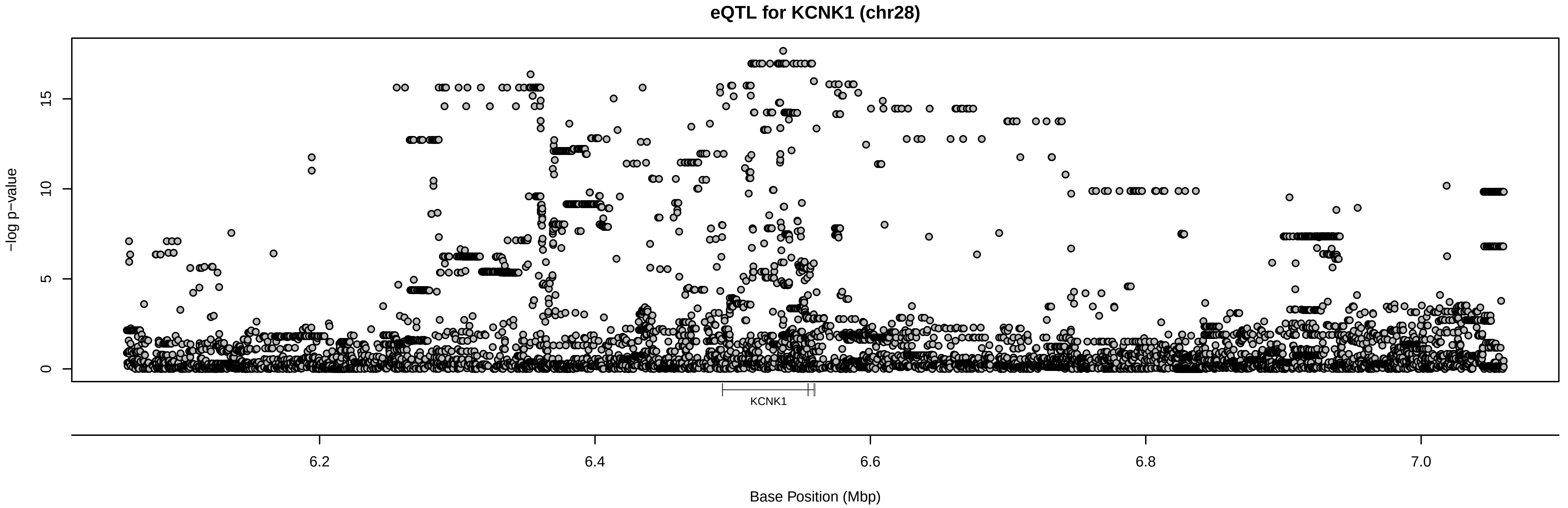
eQTL for KCNK1 (chr28)
−log p−value
0
5
10
15
KCNK1
6.2
6.4
6.6
6.8
7.0
Base Position (Mbp)

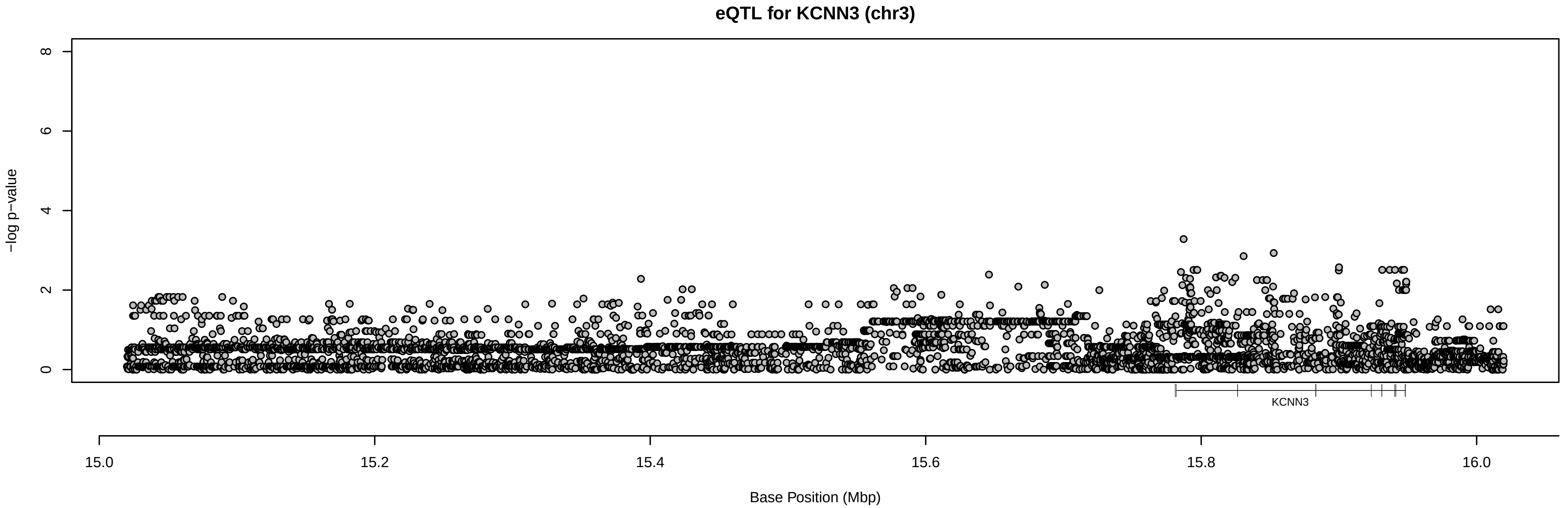
eQTL for KCNN3 (chr3)
−log p−value
0
2
4
6
8
KCNN3
15.0
15.2
15.4
15.6
15.8
16.0
Base Position (Mbp)

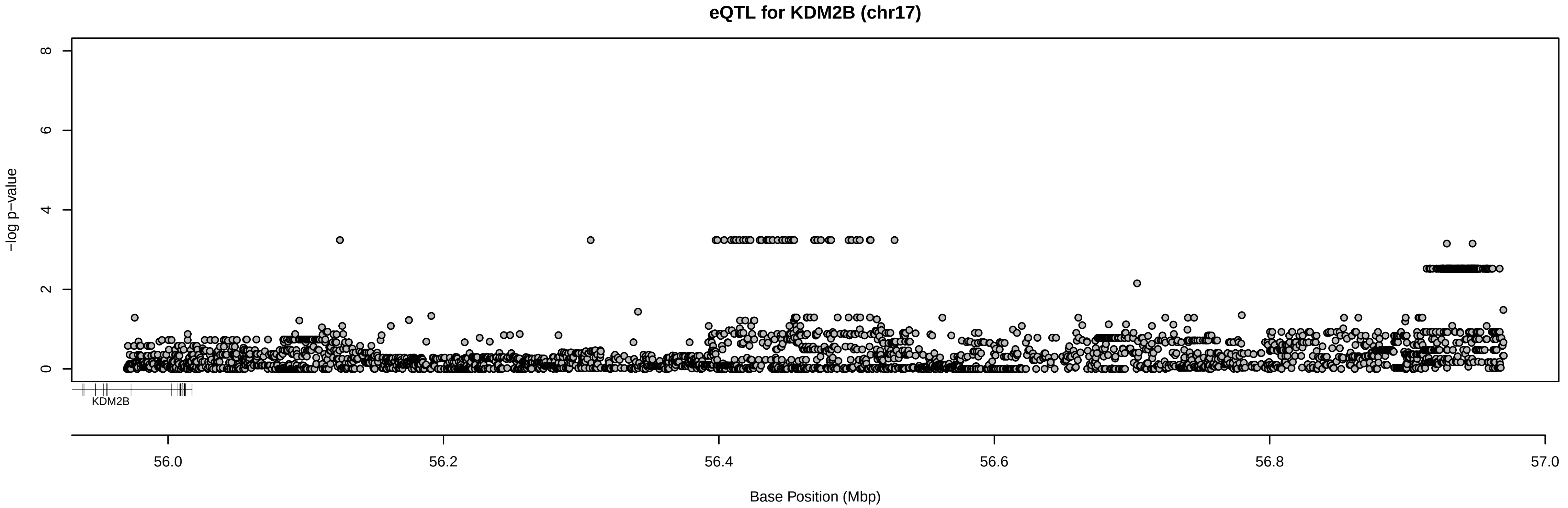
eQTL for KDM2B (chr17)
−log p−value
0
2
4
6
8
KDM2B
56.0
56.2
56.4
56.6
56.8
57.0
Base Position (Mbp)

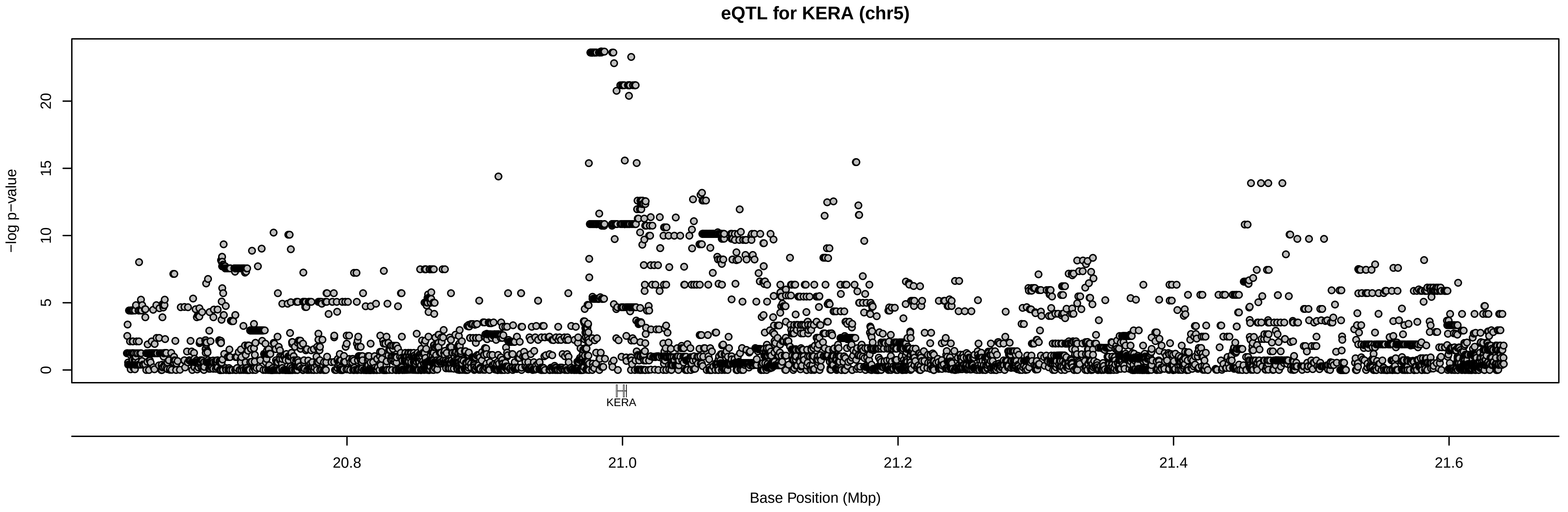
eQTL for KERA (chr5)
−log p−value
0
5
10
15
20
KERA
20.8
21.0
21.2
21.4
21.6
Base Position (Mbp)

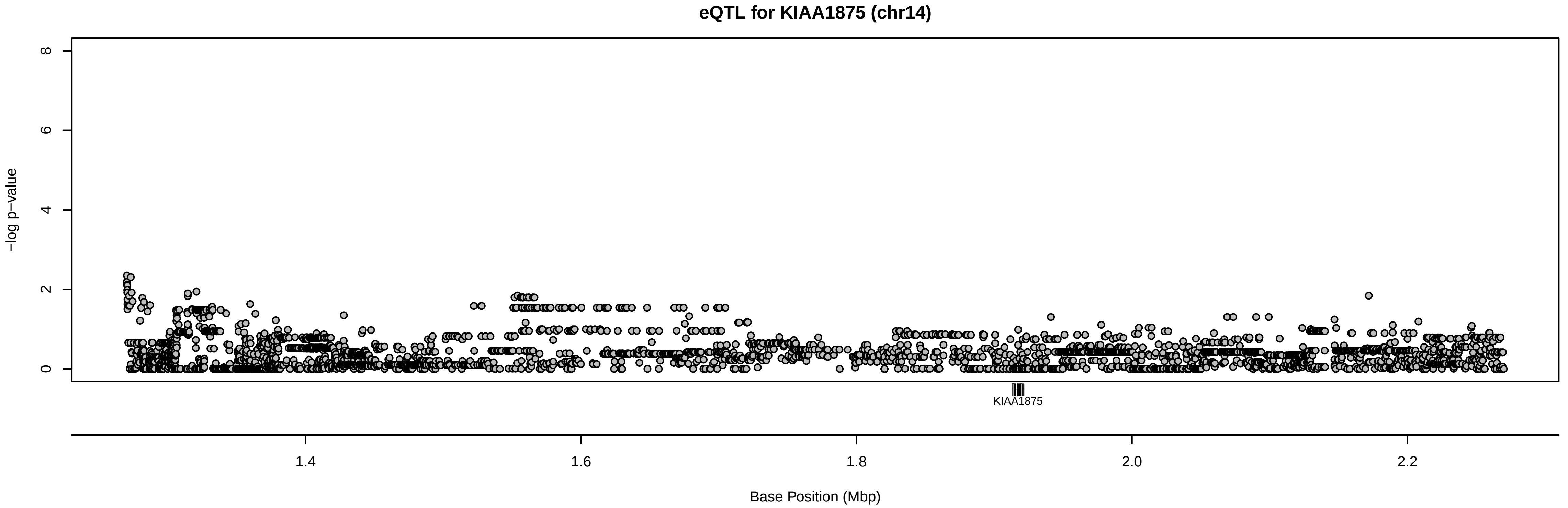
eQTL for KIAA1875 (chr14)
−log p−value
0
2
4
6
8
KIAA1875
1.4
1.6
1.8
2.0
2.2
Base Position (Mbp)

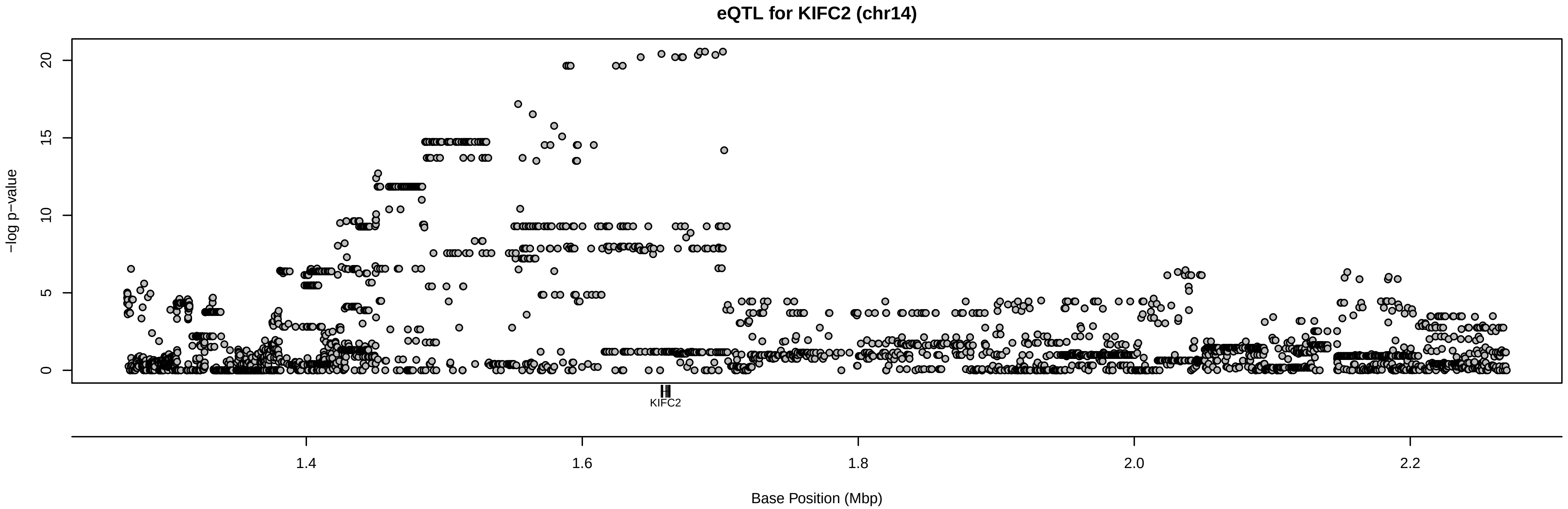
eQTL for KIFC2 (chr14)
−log p−value
0
5
10
15
20
KIFC2
1.4
1.6
1.8
2.0
2.2
Base Position (Mbp)

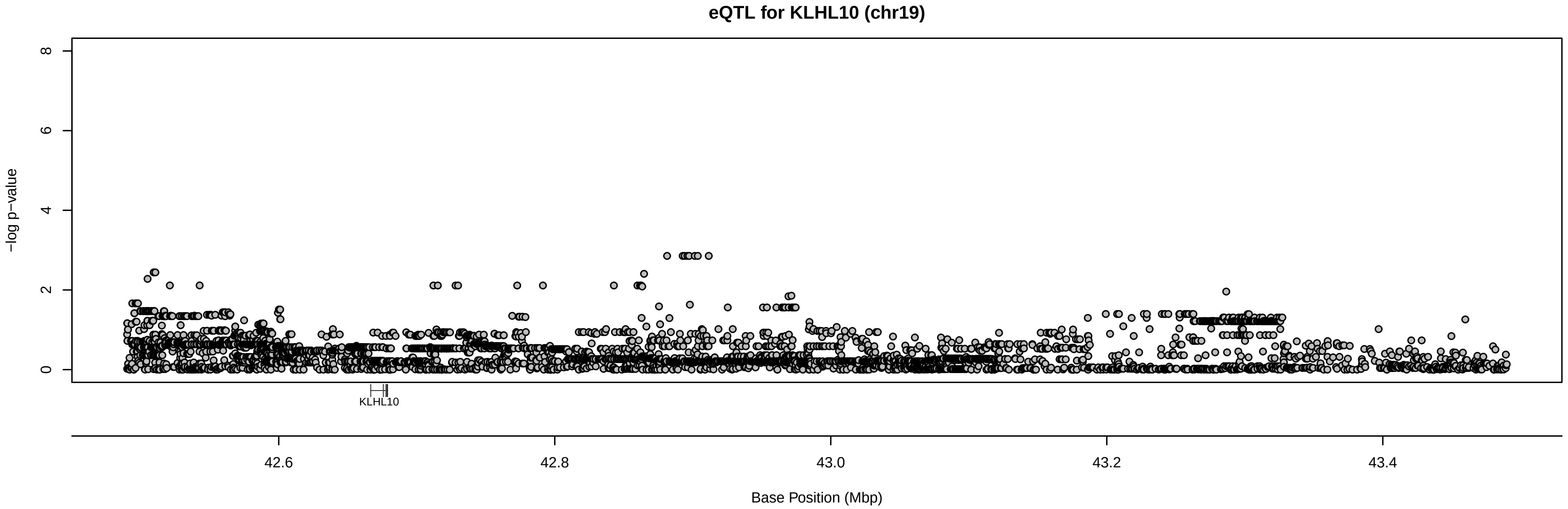
eQTL for KLHL10 (chr19)
−log p−value
0
2
4
6
8
KLHL10
42.6
42.8
43.0
43.2
43.4
Base Position (Mbp)

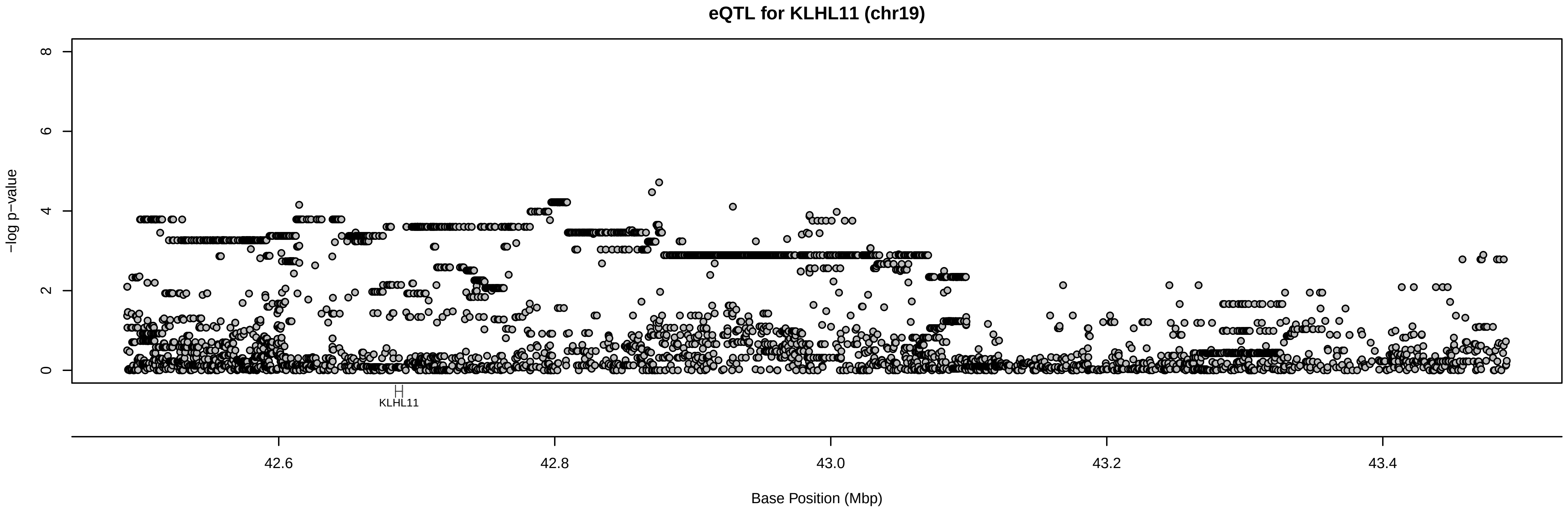
eQTL for KLHL11 (chr19)
−log p−value
0
2
4
6
8
KLHL11
42.6
42.8
43.0
43.2
43.4
Base Position (Mbp)

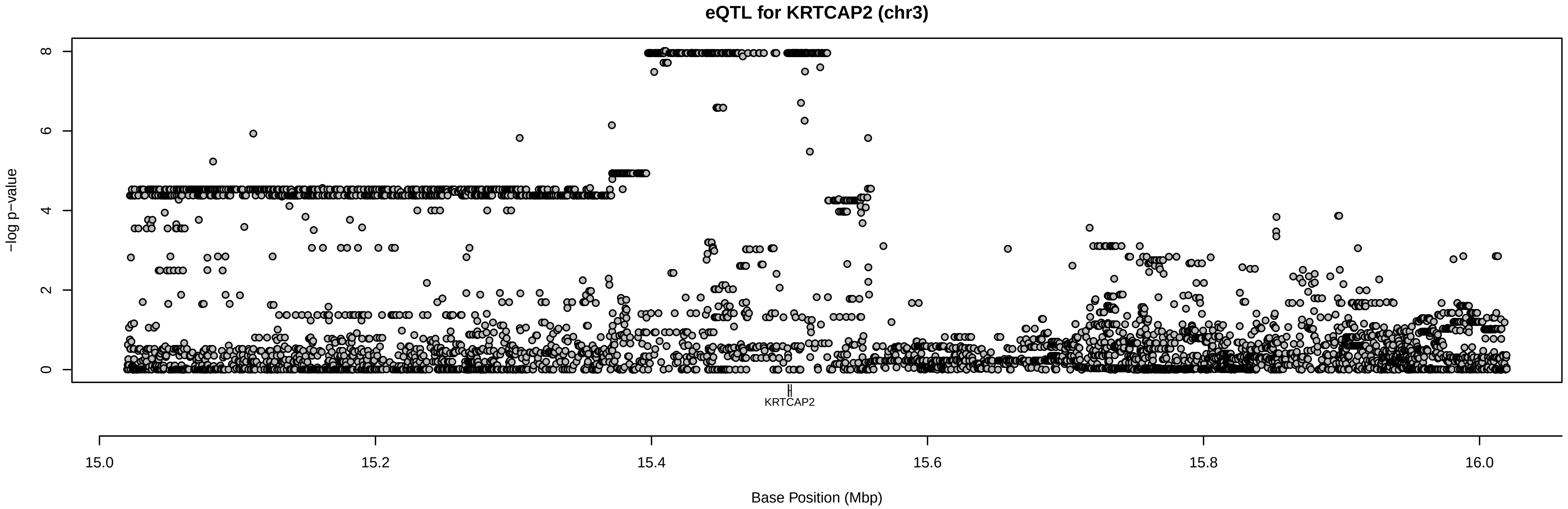
eQTL for KRTCAP2 (chr3)
−log p−value
0
2
4
6
8
KRTCAP2
15.0
15.2
15.4
15.6
15.8
16.0
Base Position (Mbp)

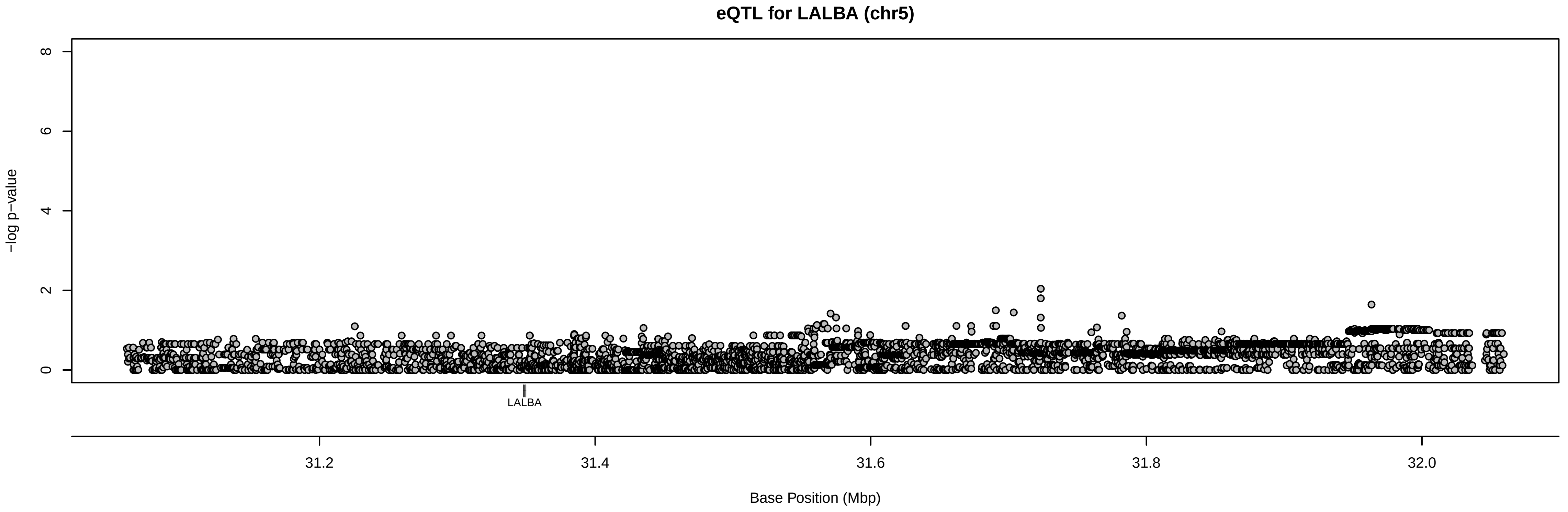
eQTL for LALBA (chr5)
−log p−value
0
2
4
6
8
LALBA
31.2
31.4
31.6
31.8
32.0
Base Position (Mbp)

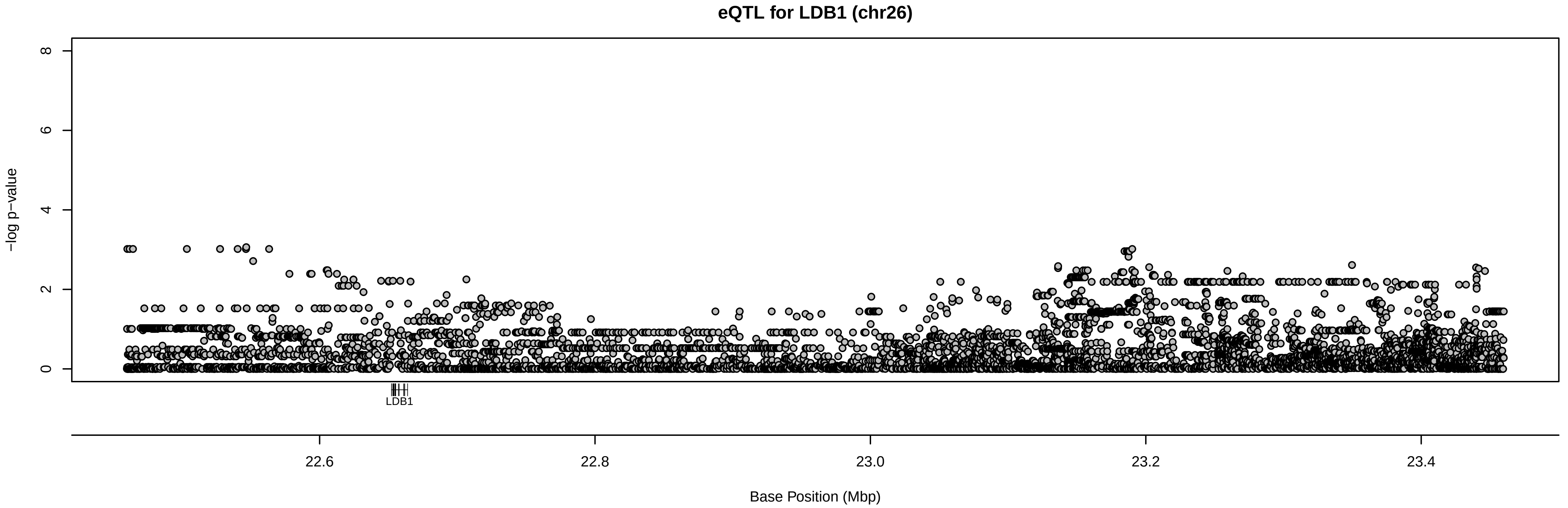
eQTL for LDB1 (chr26)
−log p−value
0
2
4
6
8
LDB1
22.6
22.8
23.0
23.2
23.4
Base Position (Mbp)

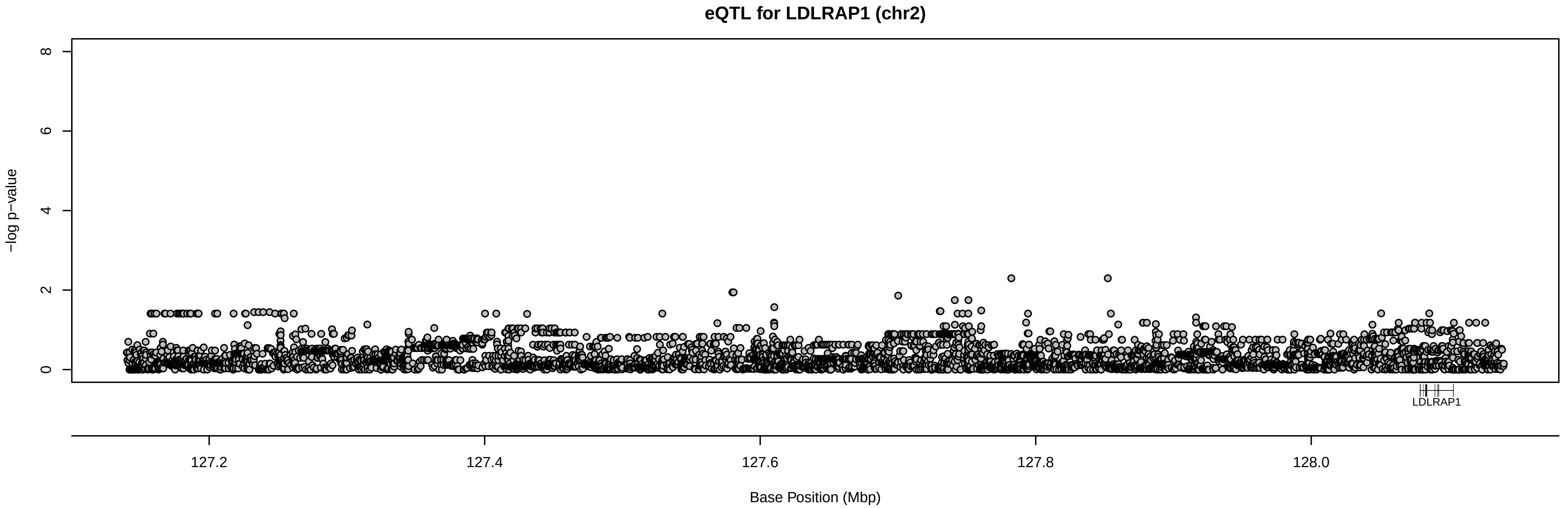
eQTL for LDLRAP1 (chr2)
−log p−value
0
2
4
6
8
LDLRAP1
127.2
127.4
127.6
127.8
128.0
Base Position (Mbp)

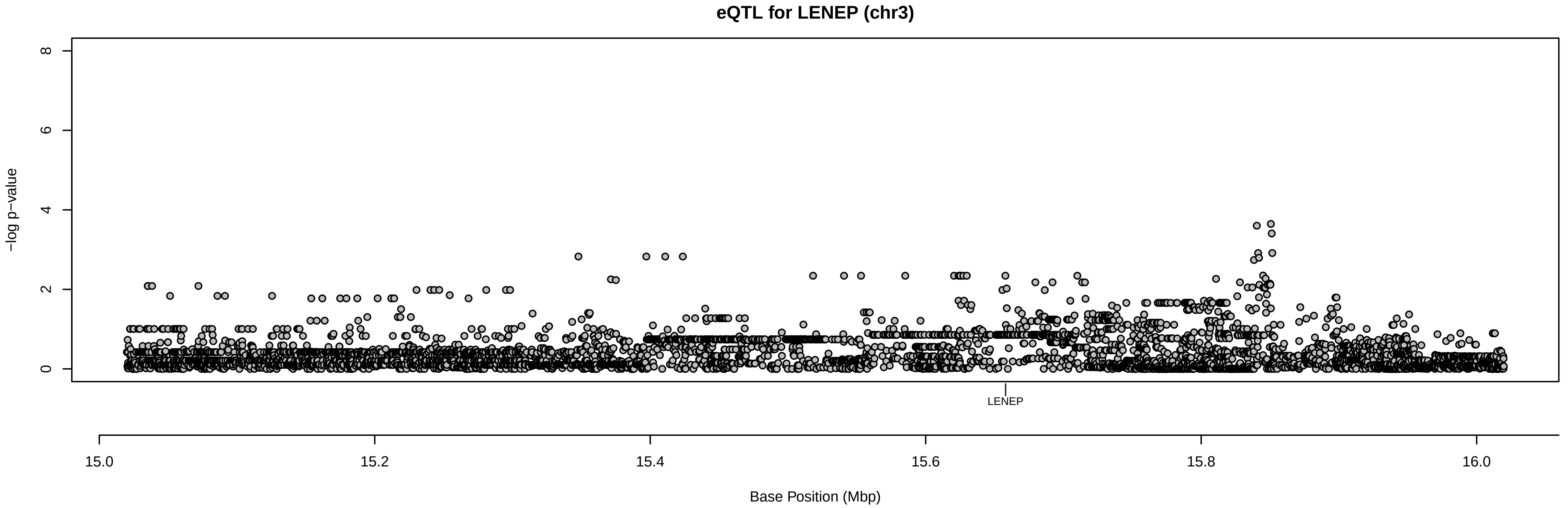
eQTL for LENEP (chr3)
−log p−value
0
2
4
6
8
LENEP
15.0
15.2
15.4
15.6
15.8
16.0
Base Position (Mbp)

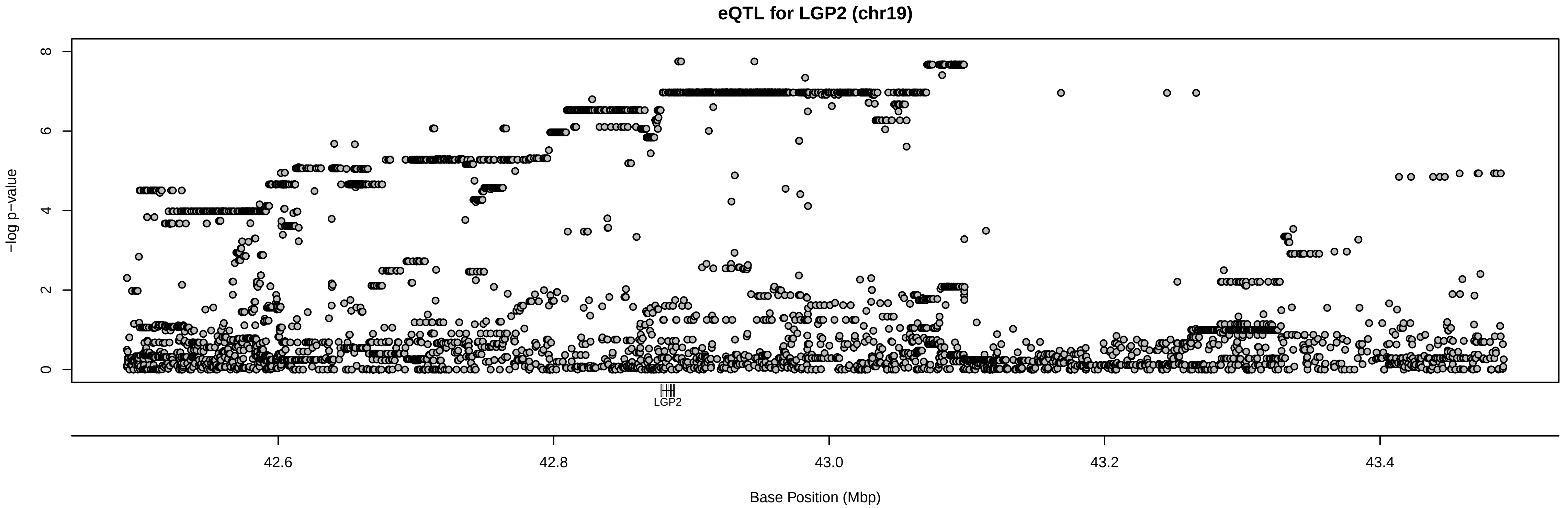
eQTL for LGP2 (chr19)
−log p−value
0
2
4
6
8
LGP2
42.6
42.8
43.0
43.2
43.4
Base Position (Mbp)

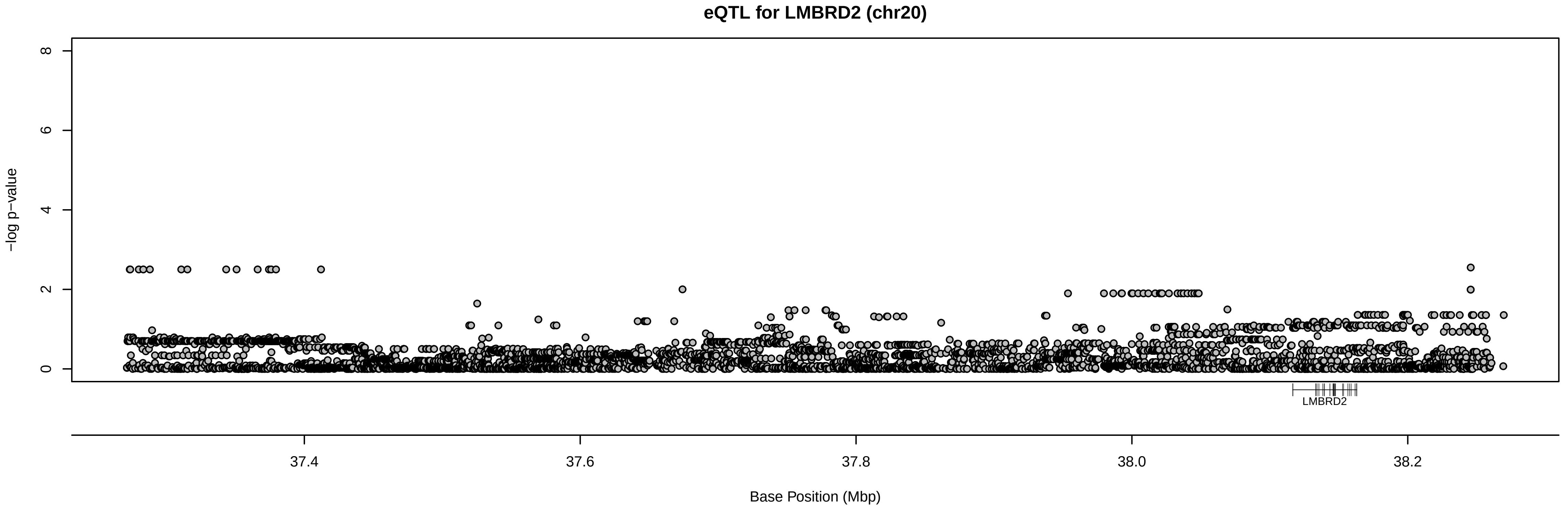
eQTL for LMBRD2 (chr20)
−log p−value
0
2
4
6
8
LMBRD2
37.4
37.6
37.8
38.0
38.2
Base Position (Mbp)

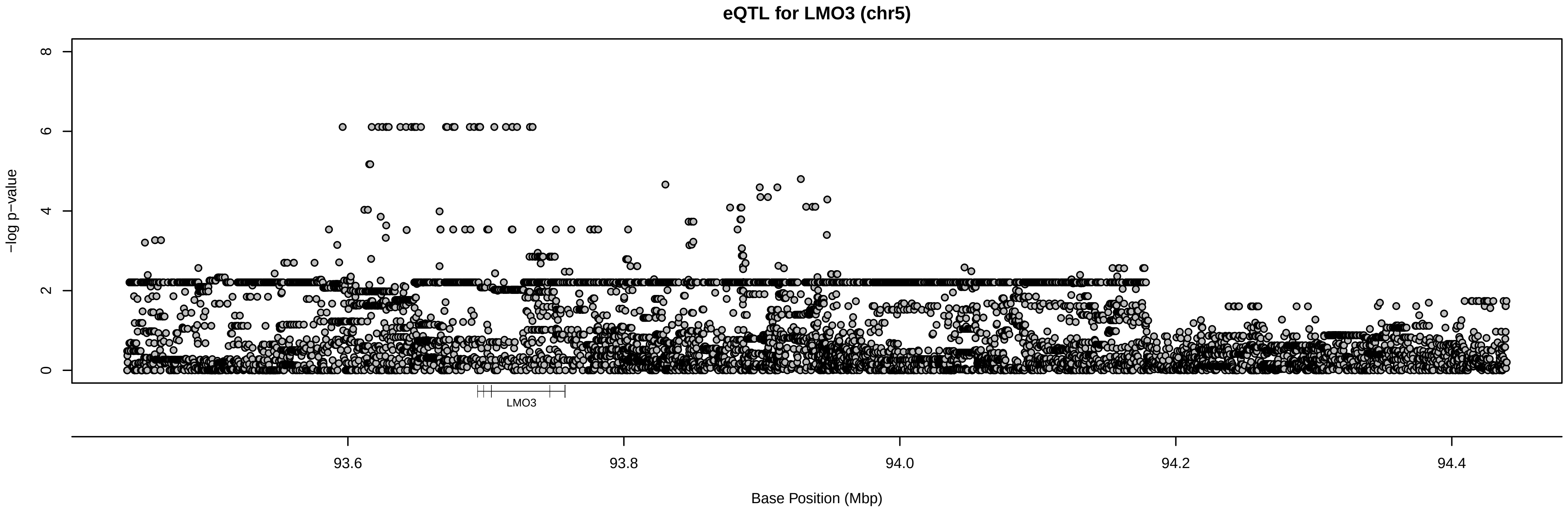
eQTL for LMO3 (chr5)
−log p−value
0
2
4
6
8
LMO3
93.6
93.8
94.0
94.2
94.4
Base Position (Mbp)

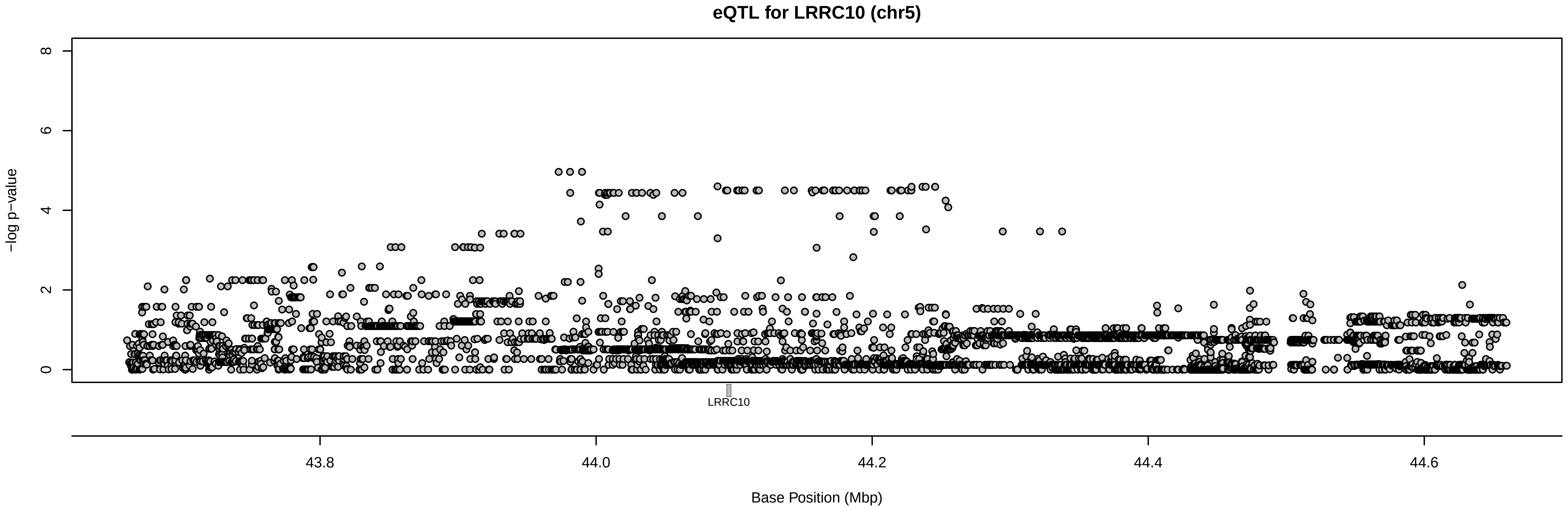
eQTL for LRRC10 (chr5)
−log p−value
0
2
4
6
8
LRRC10
43.8
44.0
44.2
44.4
44.6
Base Position (Mbp)

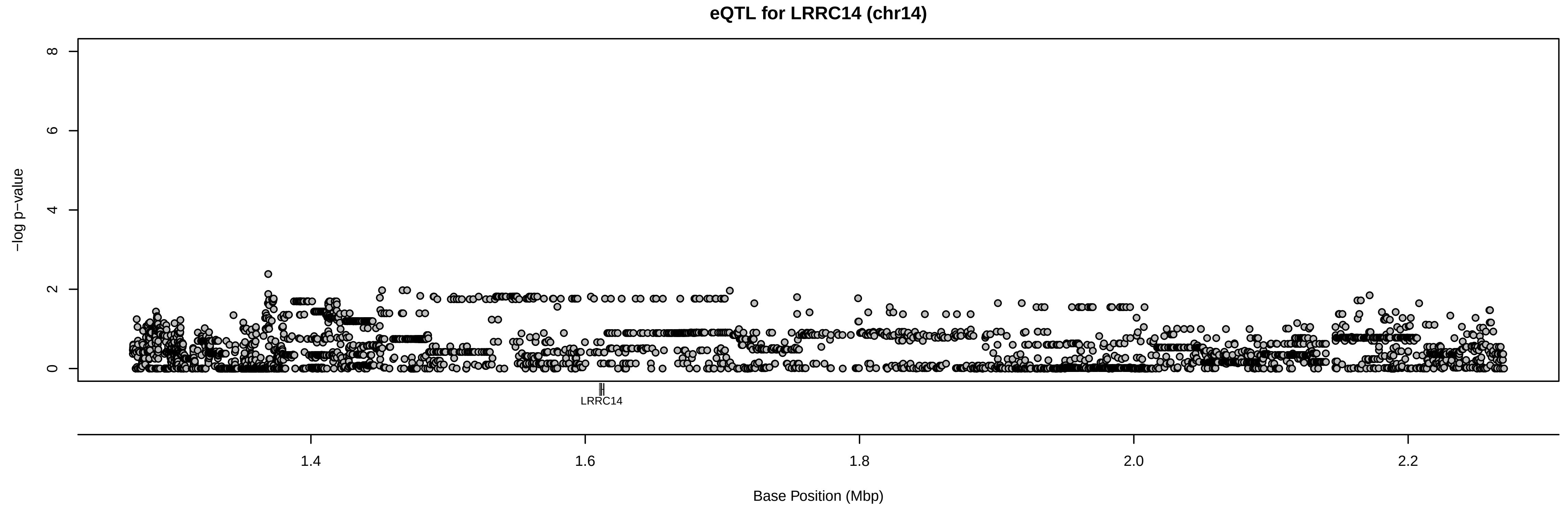
eQTL for LRRC14 (chr14)
−log p−value
0
2
4
6
8
LRRC14
1.4
1.6
1.8
2.0
2.2
Base Position (Mbp)

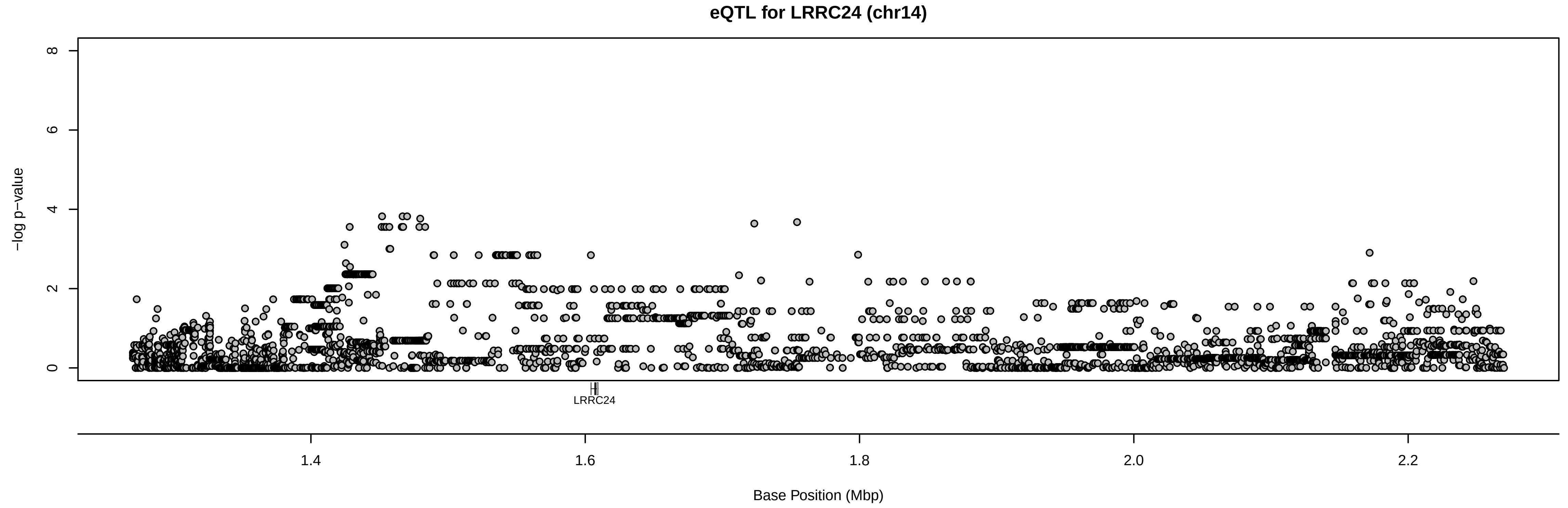
eQTL for LRRC24 (chr14)
−log p−value
0
2
4
6
8
LRRC24
1.4
1.6
1.8
2.0
2.2
Base Position (Mbp)

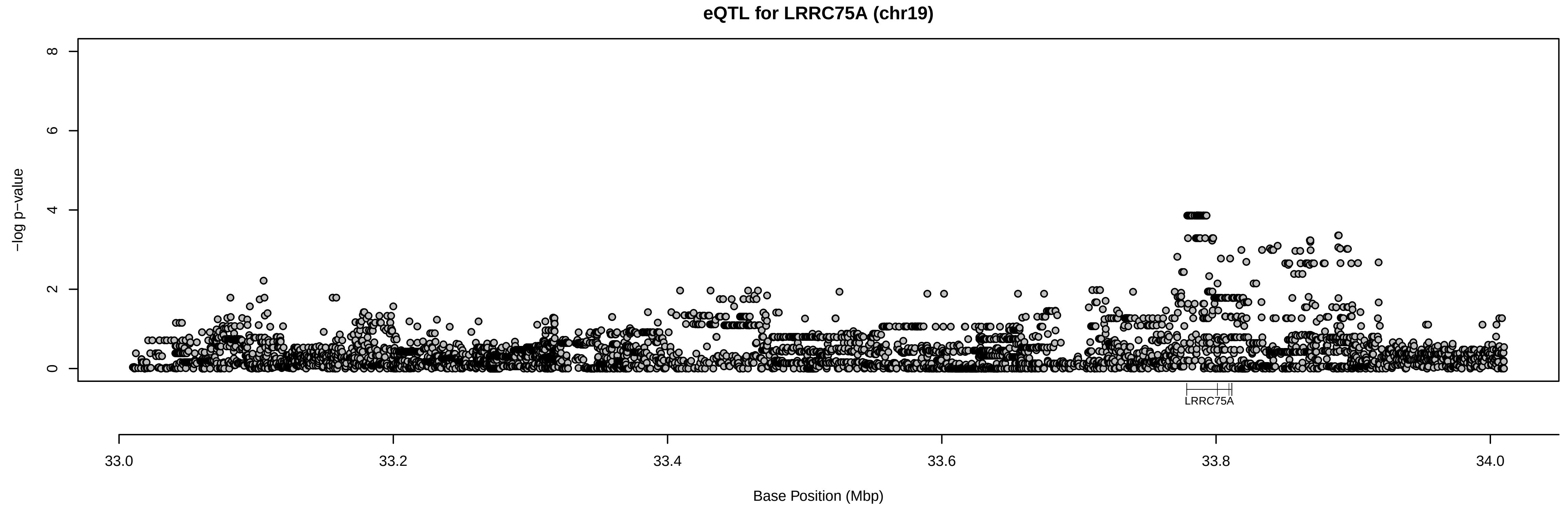

eQTL for LRRC75A (chr19)
−log p−value
0
2
4
6
8
LRRC75A
33.0
33.2
33.4
33.6
33.8
34.0
Base Position (Mbp)

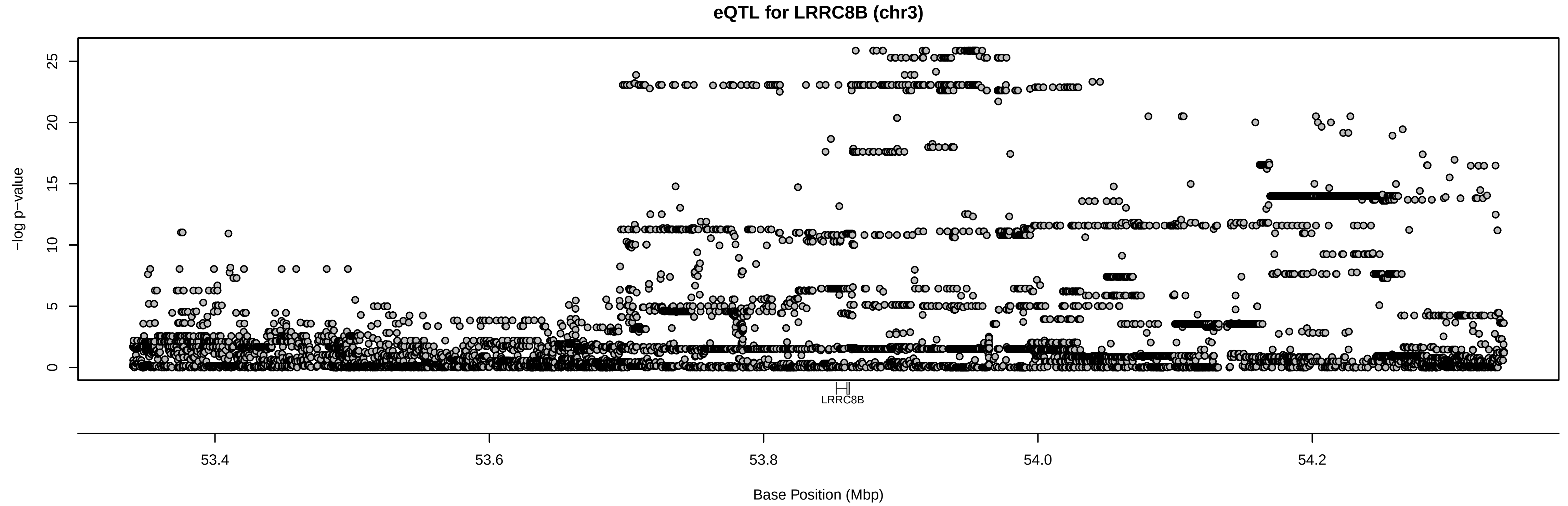

eQTL for LRRC8B (chr3)
−log p−value
0
5
10
15
20
25
LRRC8B
53.4
53.6
53.8
54.0
54.2
Base Position (Mbp)

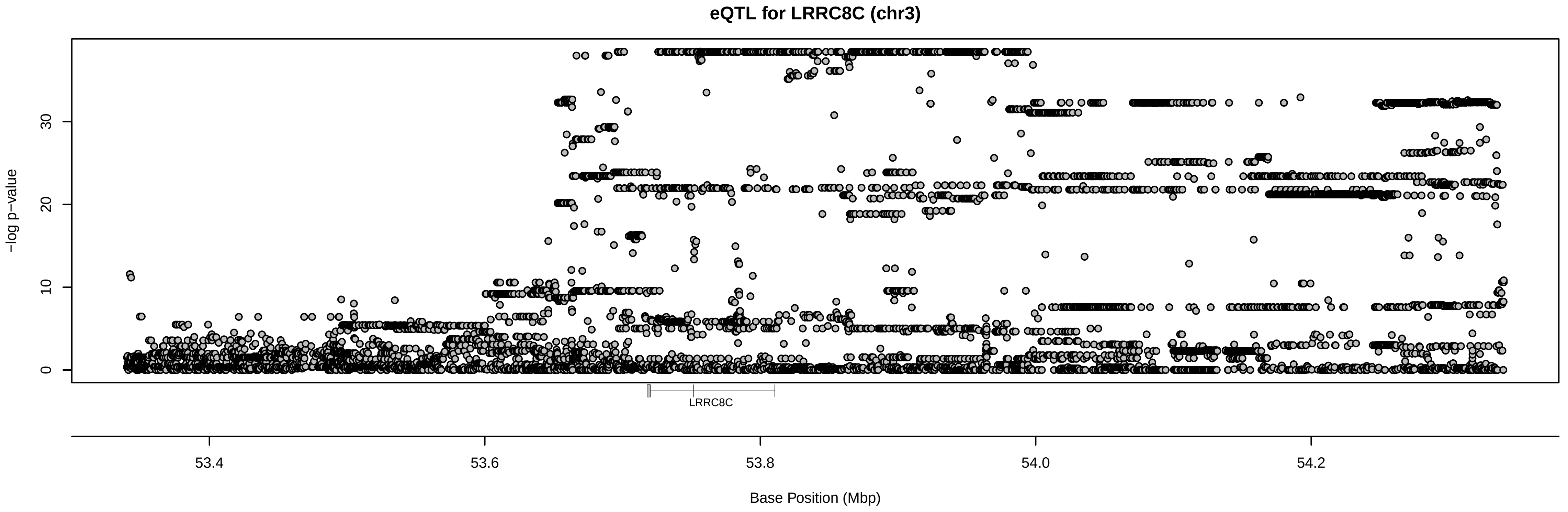
eQTL for LRRC8C (chr3)
−log p−value
0
10
20
30
LRRC8C
53.4
53.6
53.8
54.0
54.2
Base Position (Mbp)

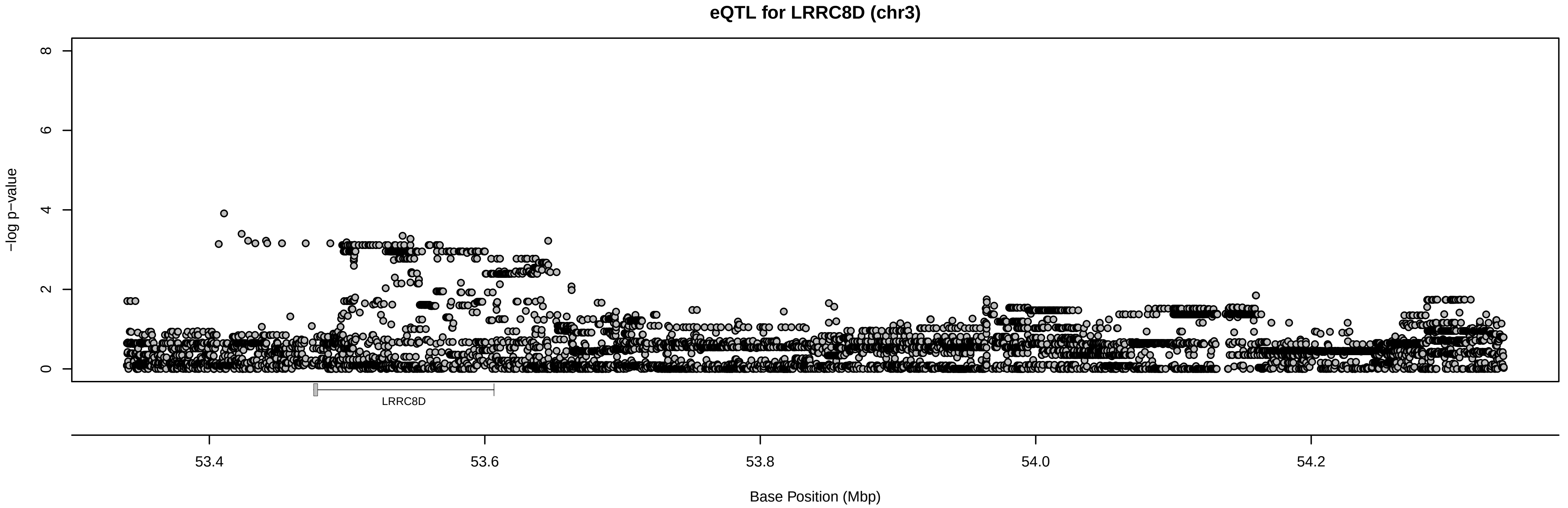
eQTL for LRRC8D (chr3)
−log p−value
0
2
4
6
8
LRRC8D
53.4
53.6
53.8
54.0
54.2
Base Position (Mbp)

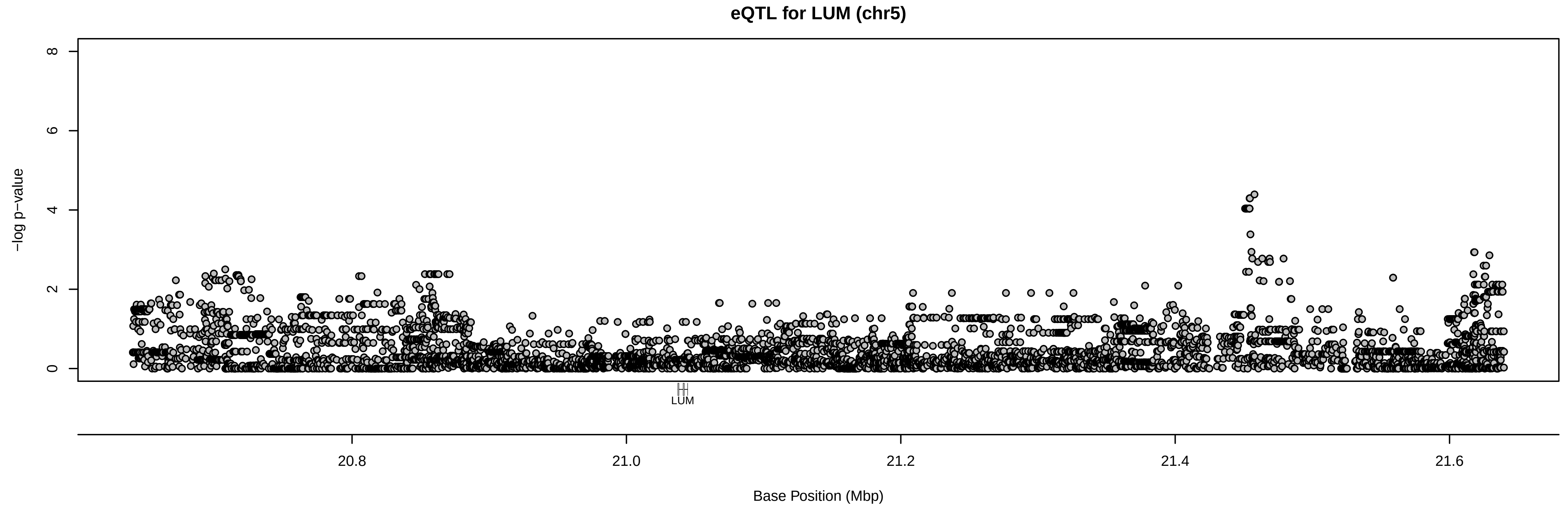

eQTL for LUM (chr5)
−log p−value
0
2
4
6
8
LUM
20.8
21.0
21.2
21.4
21.6
Base Position (Mbp)

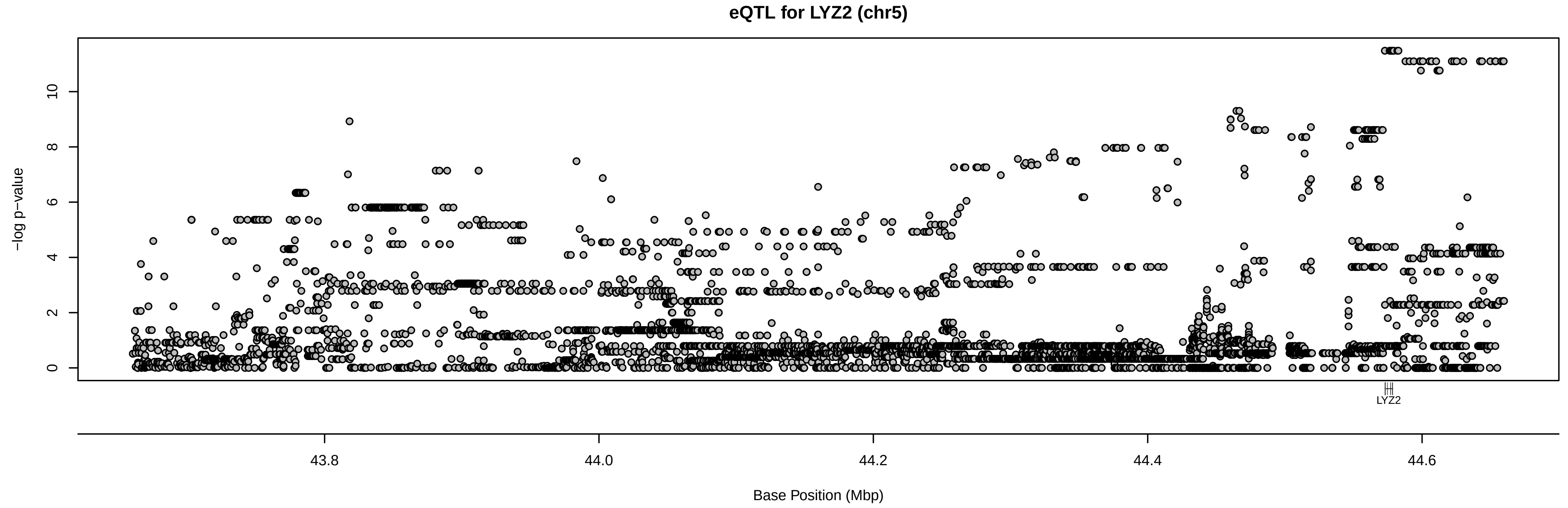

eQTL for LYZ2 (chr5)
−log p−value
0
2
4
6
8
10
LYZ2
43.8
44.0
44.2
44.4
44.6
Base Position (Mbp)

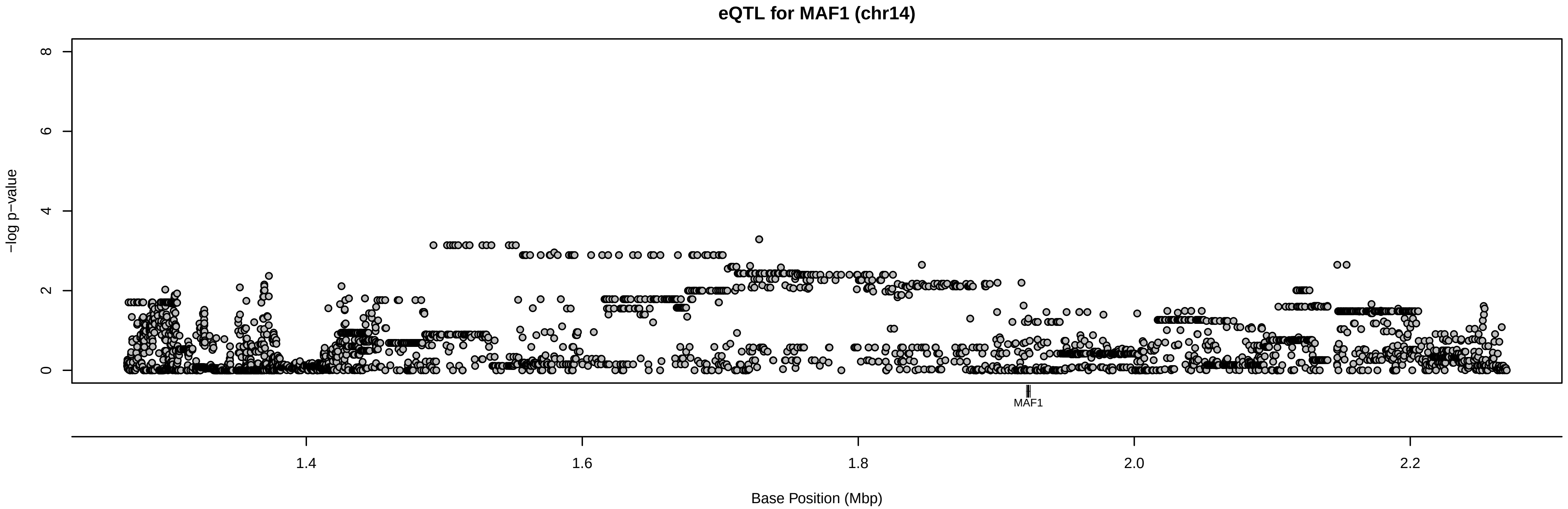
eQTL for MAF1 (chr14)
−log p−value
0
2
4
6
8
MAF1
1.4
1.6
1.8
2.0
2.2
Base Position (Mbp)

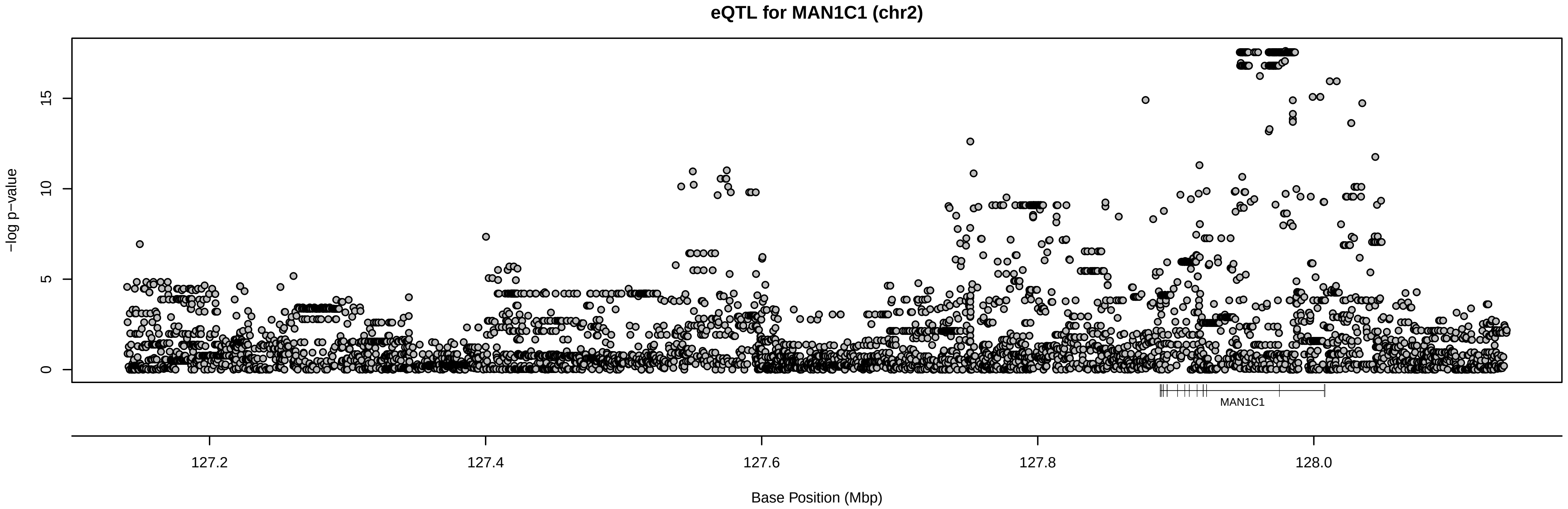
eQTL for MAN1C1 (chr2)
−log p−value
0
5
10
15
MAN1C1
127.2
127.4
127.6
127.8
128.0
Base Position (Mbp)

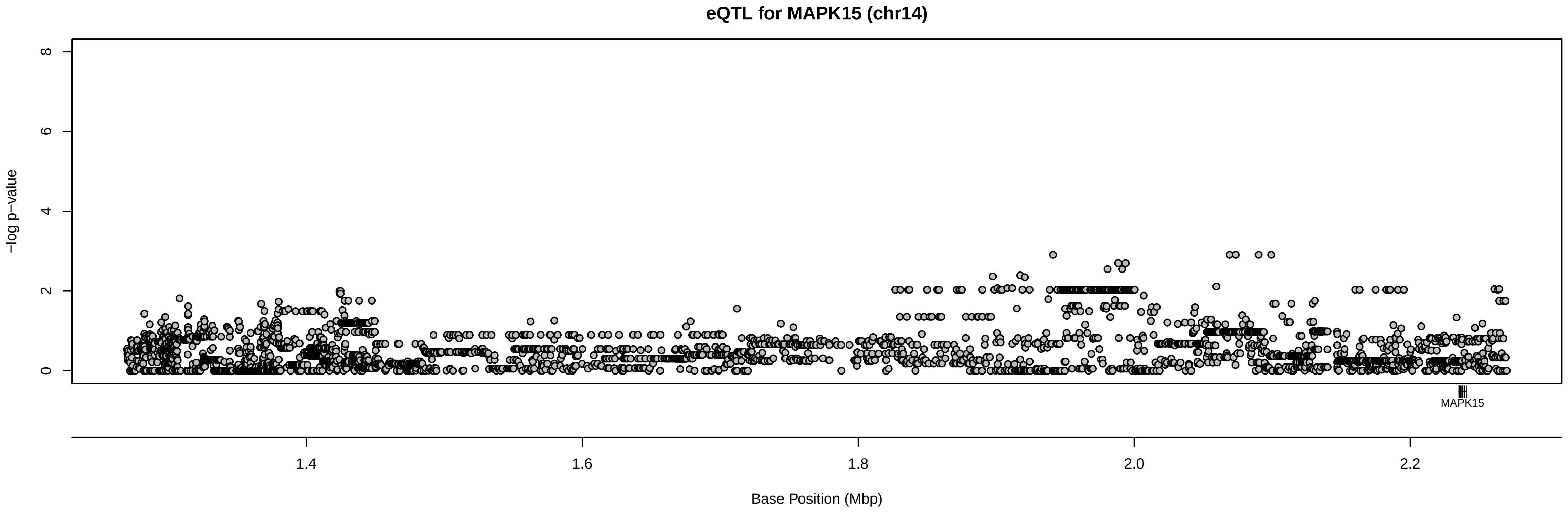
eQTL for MAPK15 (chr14)
−log p−value
0
2
4
6
8
MAPK15
1.4
1.6
1.8
2.0
2.2
Base Position (Mbp)

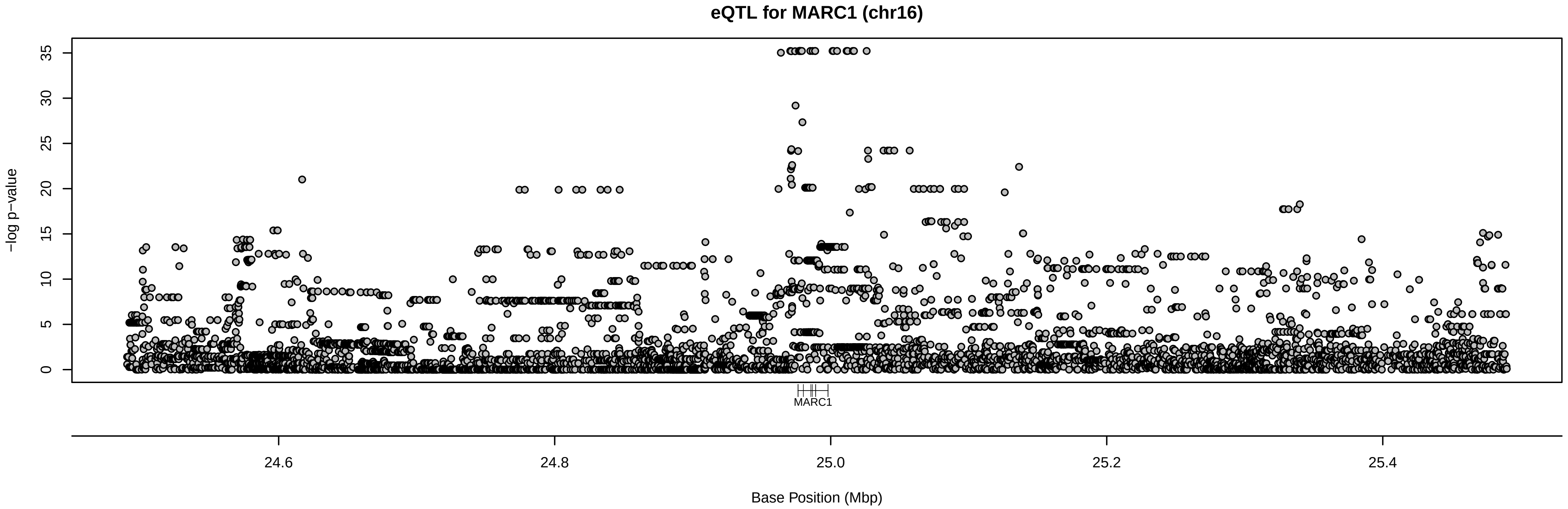
eQTL for MARC1 (chr16)
−log p−value
0
5
10
15
20
25
30
35
MARC1
24.6
24.8
25.0
25.2
25.4
Base Position (Mbp)

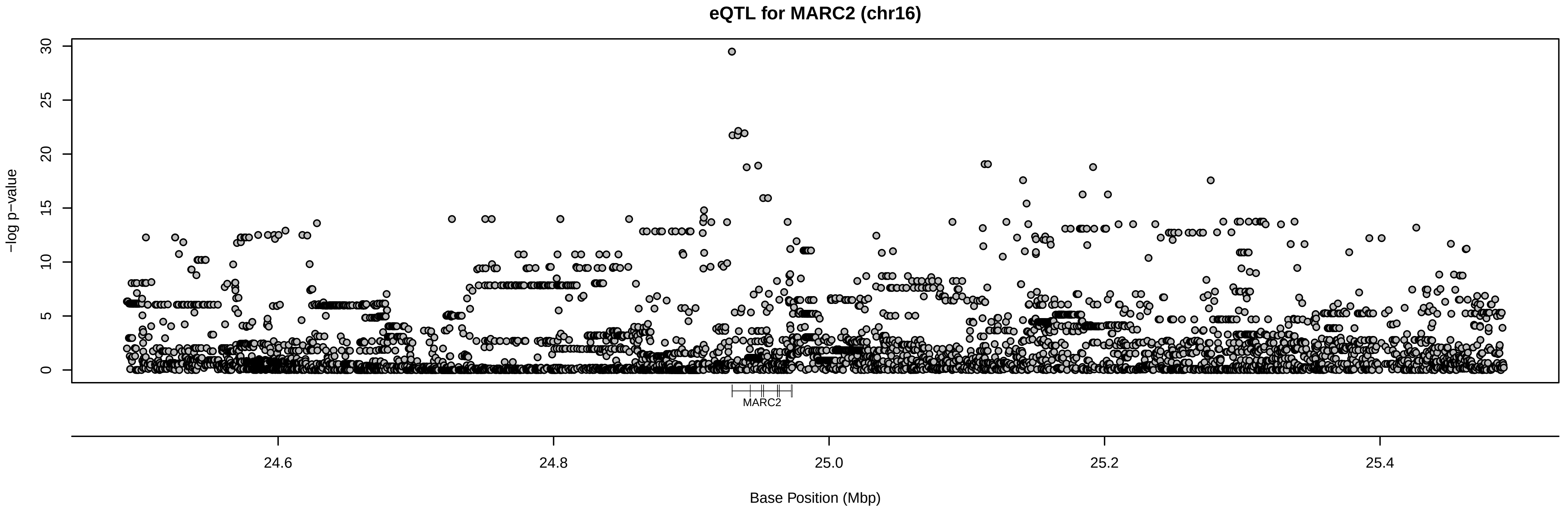
eQTL for MARC2 (chr16)
−log p−value
30
25
20
15
10
5
0
MARC2
24.6
24.8
25.0
25.2
25.4
Base Position (Mbp)

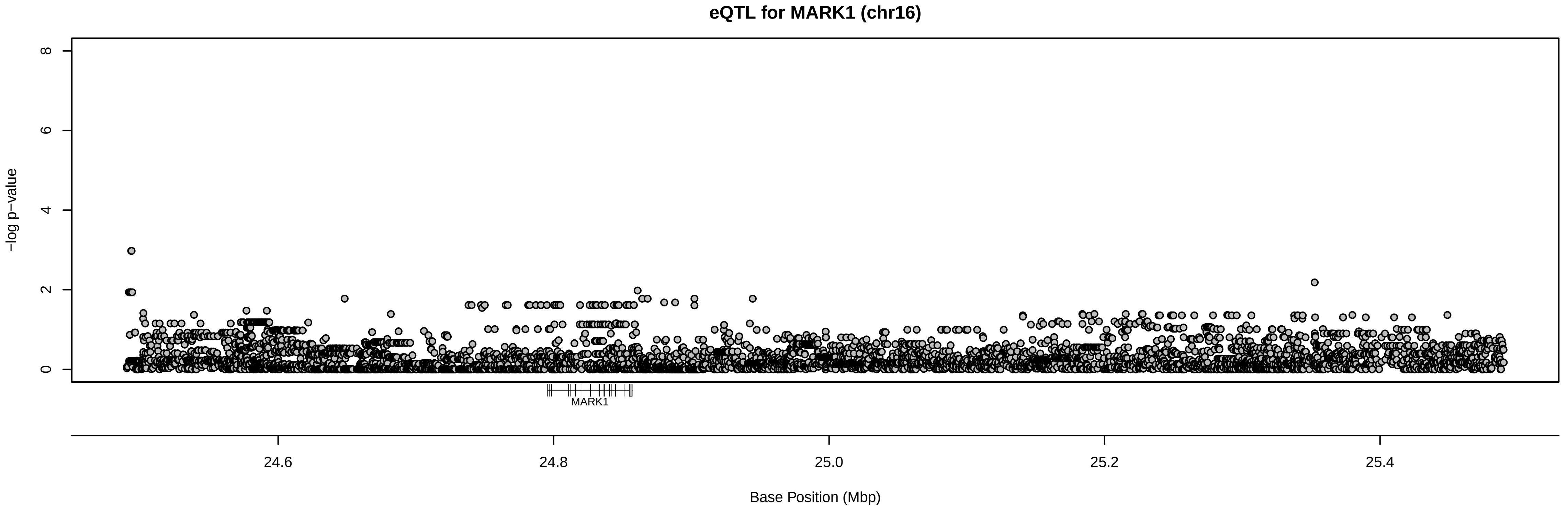
eQTL for MARK1 (chr16)
−log p−value
8
6
4
2
0
MARK1
24.6
24.8
25.0
25.2
25.4
Base Position (Mbp)

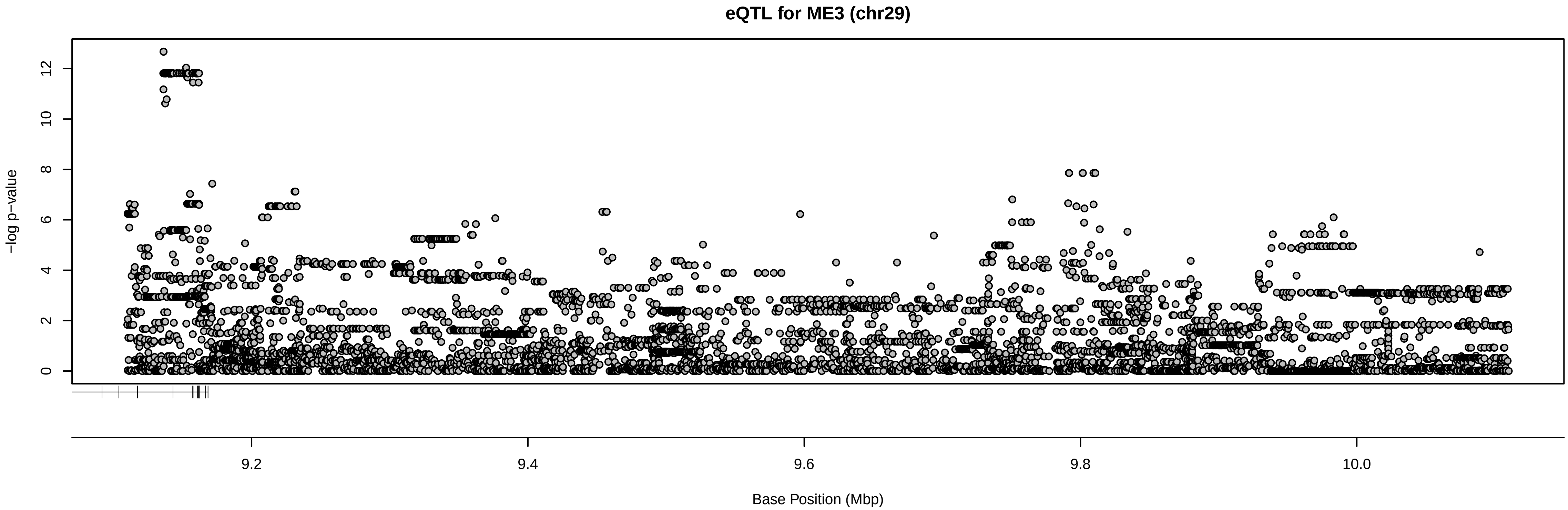

eQTL for ME3 (chr29)
−log p−value
0
2
4
6
8
10
12
9.2
9.4
9.6
9.8
10.0
Base Position (Mbp)

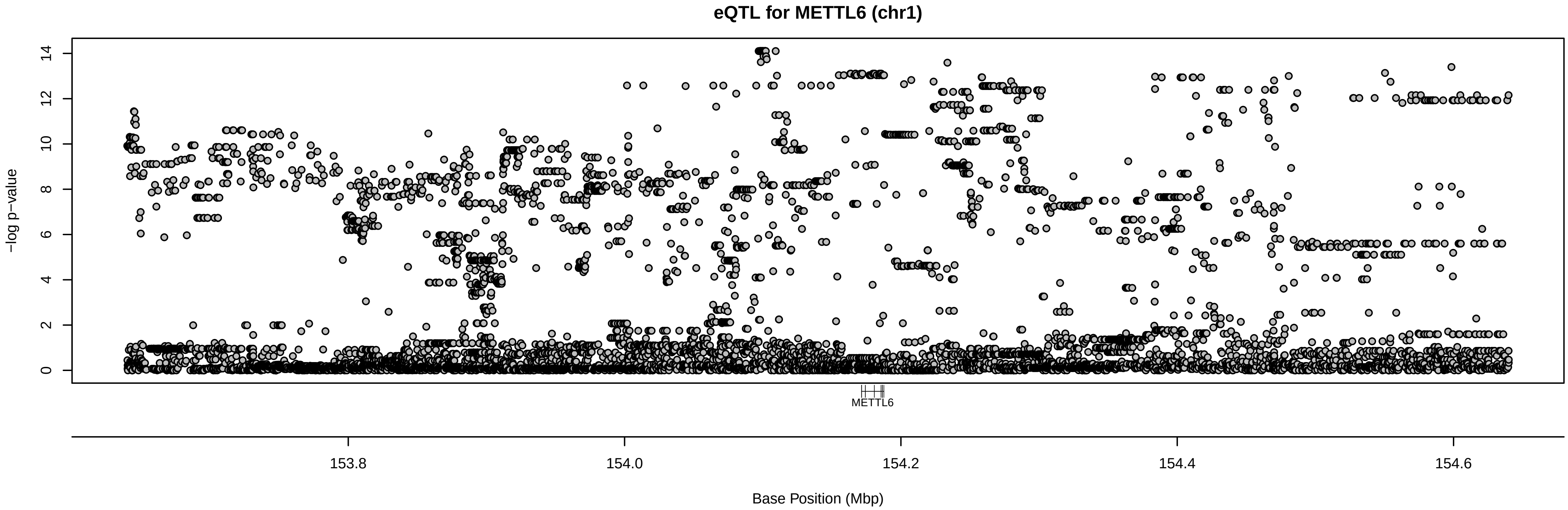

eQTL for METTL6 (chr1)
−log p−value
0
2
4
6
8
10
12
14
METTL6
153.8
154.0
154.2
154.4
154.6
Base Position (Mbp)

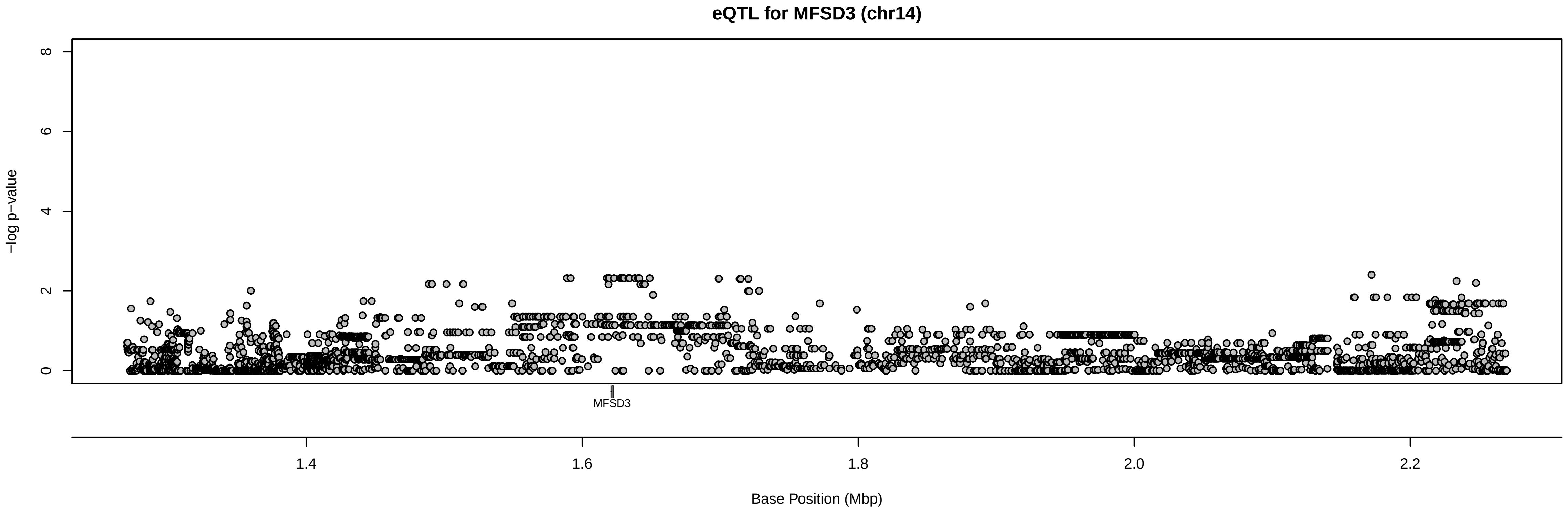
eQTL for MFSD3 (chr14)
−log p−value
0
2
4
6
8
MFSD3
1.4
1.6
1.8
2.0
2.2
Base Position (Mbp)

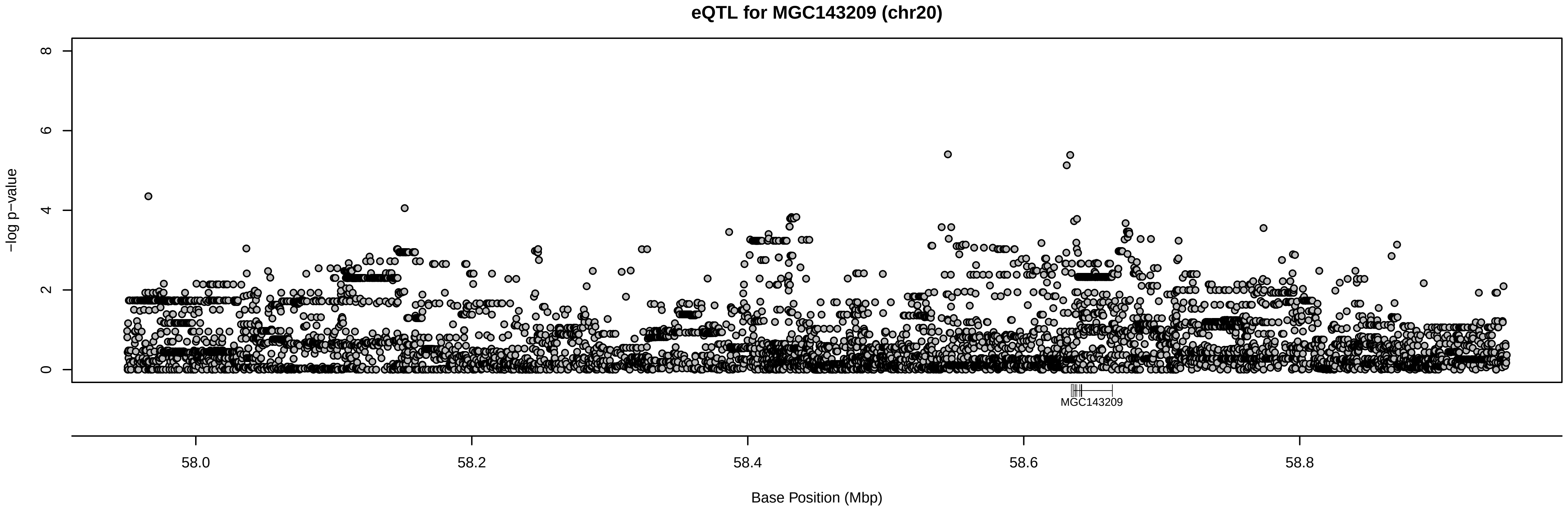
eQTL for MGC143209 (chr20)
−log p−value
0
2
4
6
8
MGC143209
58.0
58.2
58.4
58.6
58.8
Base Position (Mbp)

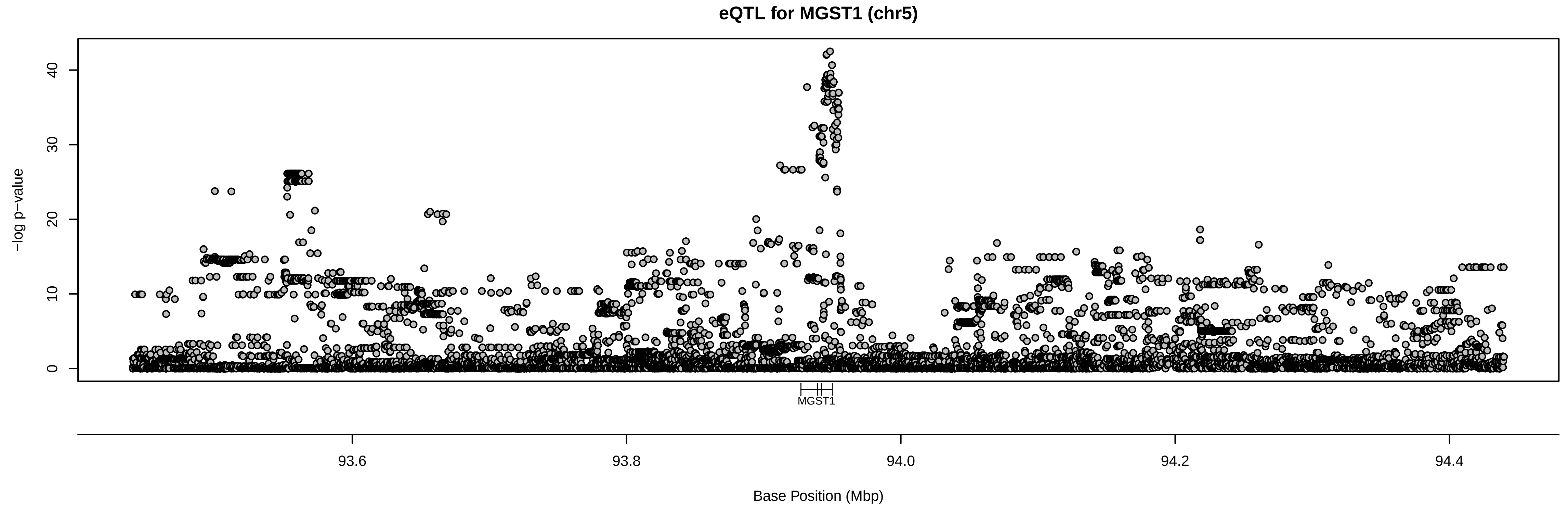

eQTL for MGST1 (chr5)
−log p−value
0
10
20
30
40
MGST1
93.6
93.8
94.0
94.2
94.4
Base Position (Mbp)

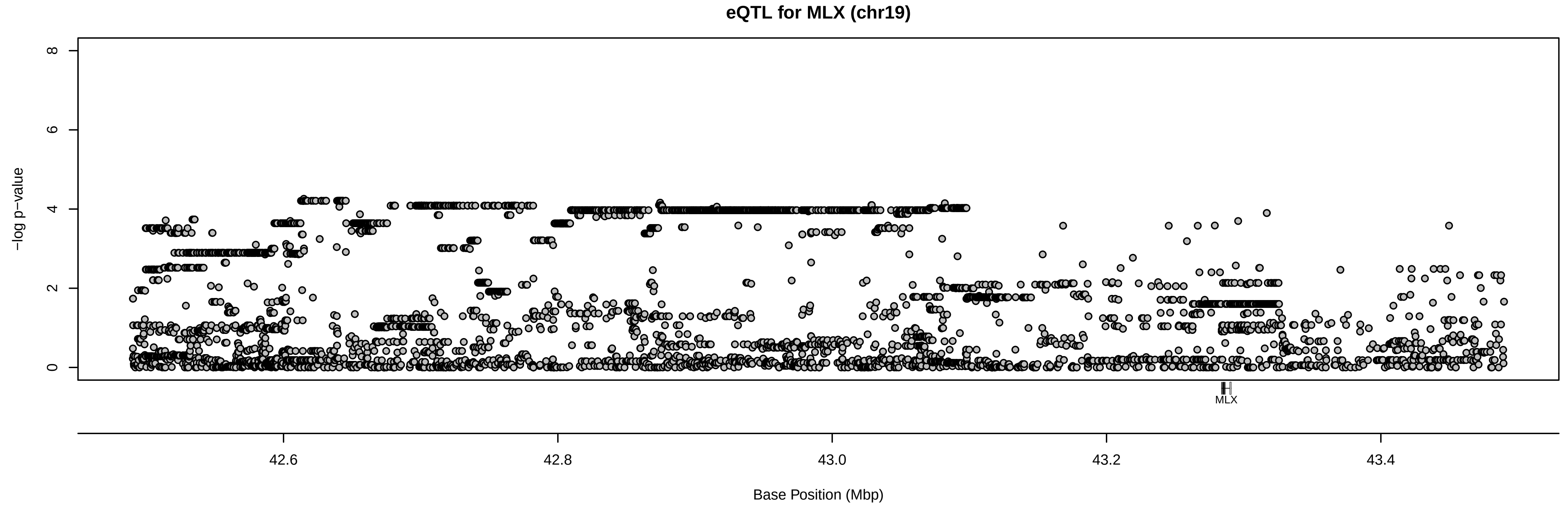

eQTL for MLX (chr19)
−log p−value
0
2
4
6
8
MLX
42.6
42.8
43.0
43.2
43.4
Base Position (Mbp)

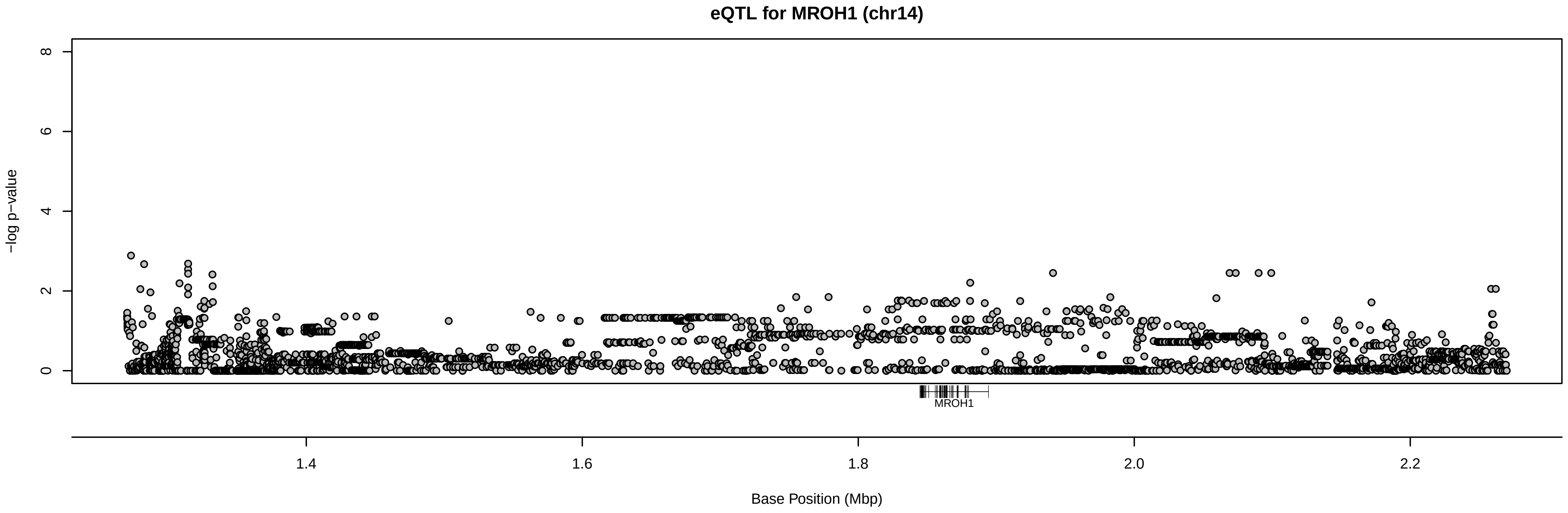

eQTL for MROH1 (chr14)
−log p−value
0
2
4
6
8
MROH1
1.4
1.6
1.8
2.0
2.2
Base Position (Mbp)

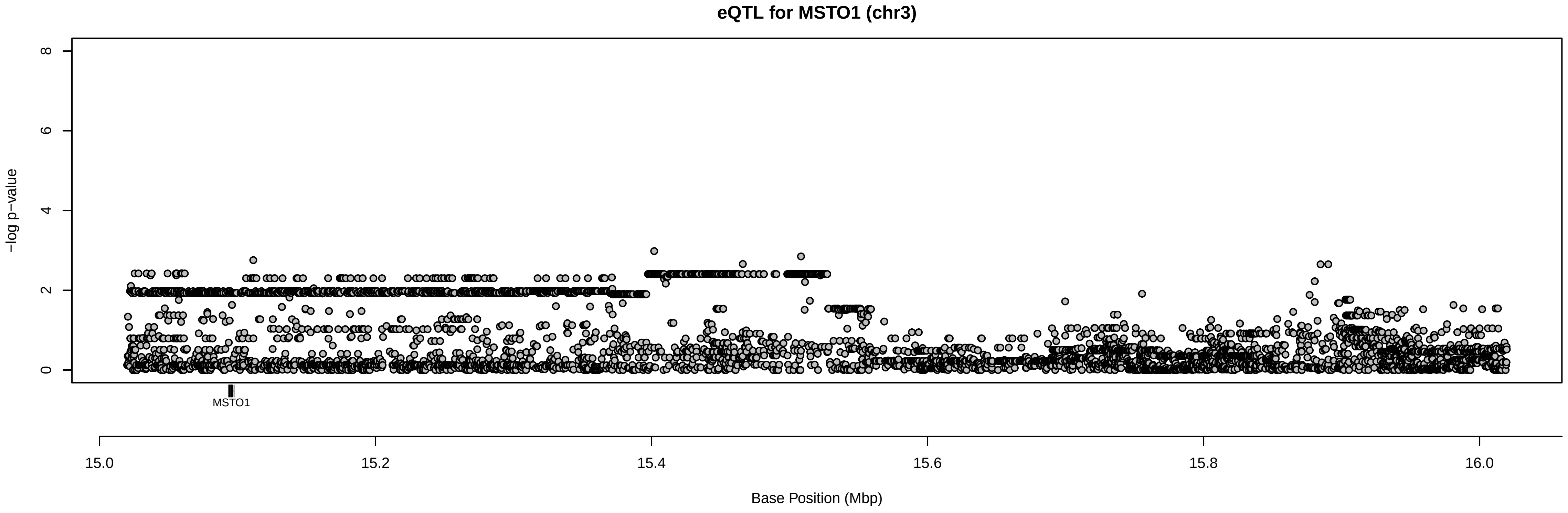

eQTL for MSTO1 (chr3)
−log p−value
0
2
4
6
8
MSTO1
15.0
15.2
15.4
15.6
15.8
16.0
Base Position (Mbp)

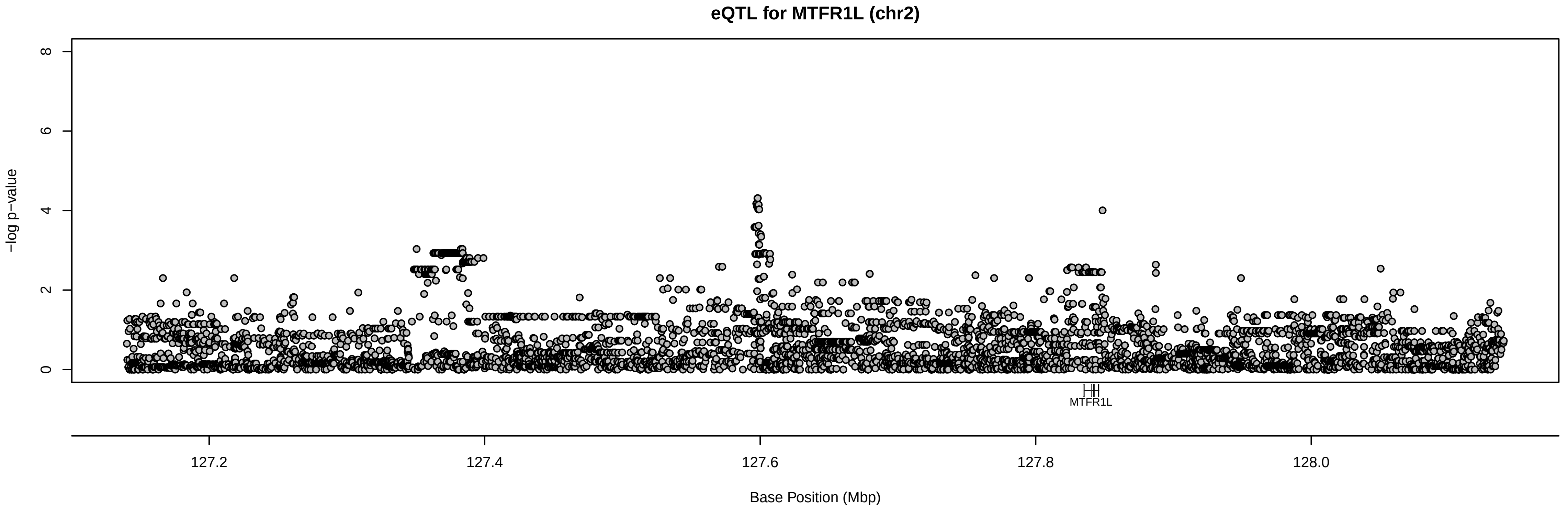
eQTL for MTFR1L (chr2)
−log p−value
0
2
4
6
8
MTFR1L
127.2
127.4
127.6
127.8
128.0
Base Position (Mbp)

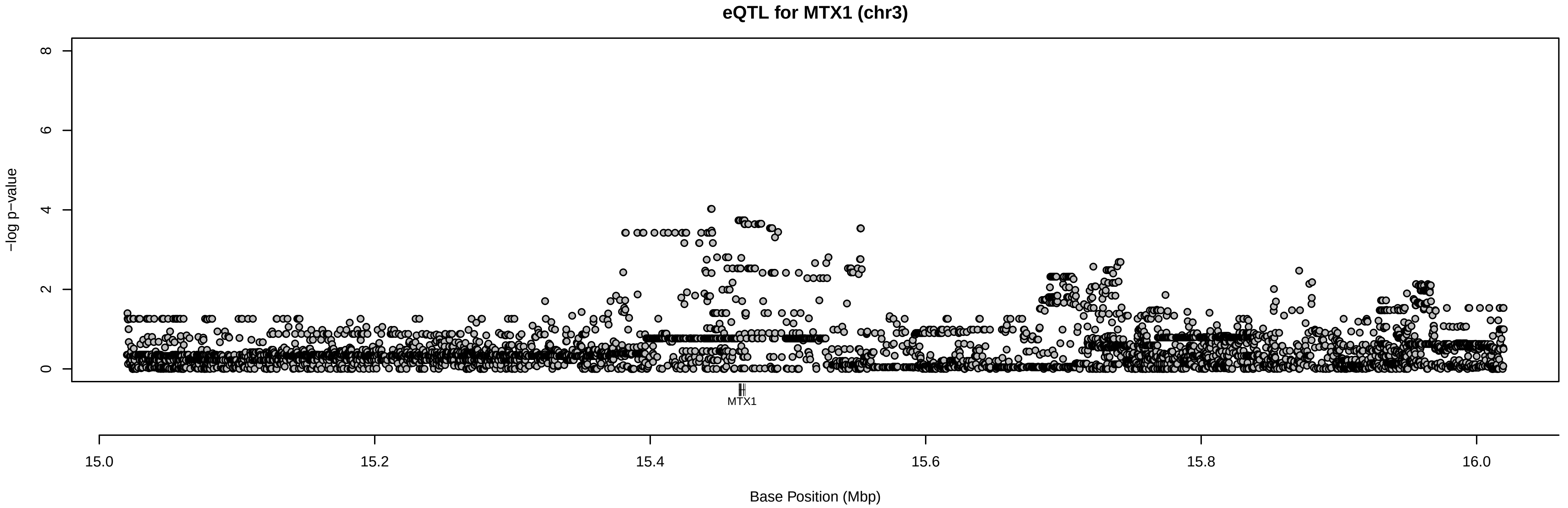
eQTL for MTX1 (chr3)
−log p−value
0
2
4
6
8
MTX1
15.0
15.2
15.4
15.6
15.8
16.0
Base Position (Mbp)

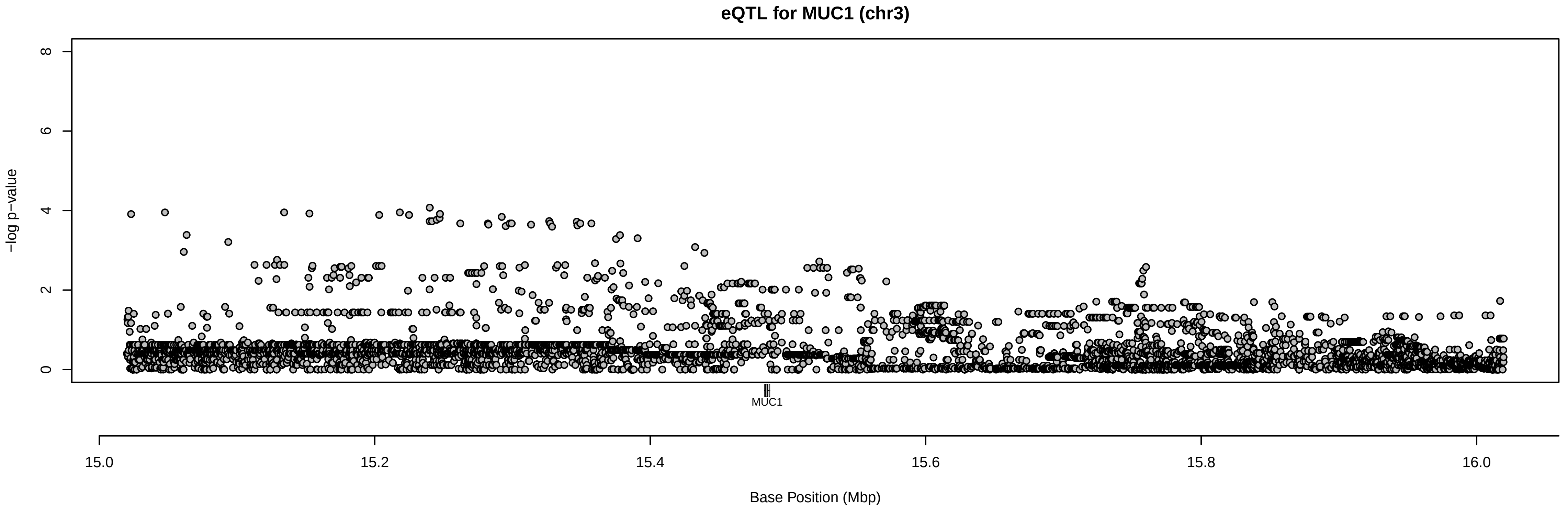
eQTL for MUC1 (chr3)
−log p−value
0
2
4
6
8
MUC1
15.0
15.2
15.4
15.6
15.8
16.0
Base Position (Mbp)

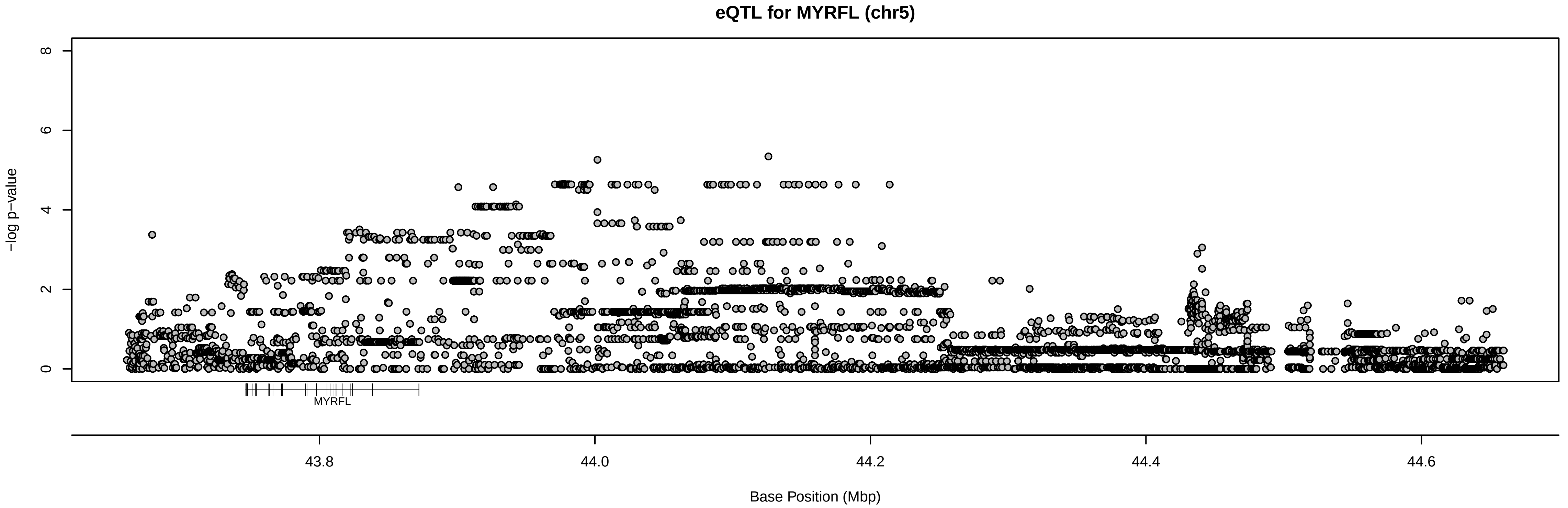
eQTL for MYRFL (chr5)
−log p−value
0
2
4
6
8
MYRFL
43.8
44.0
44.2
44.4
44.6
Base Position (Mbp)

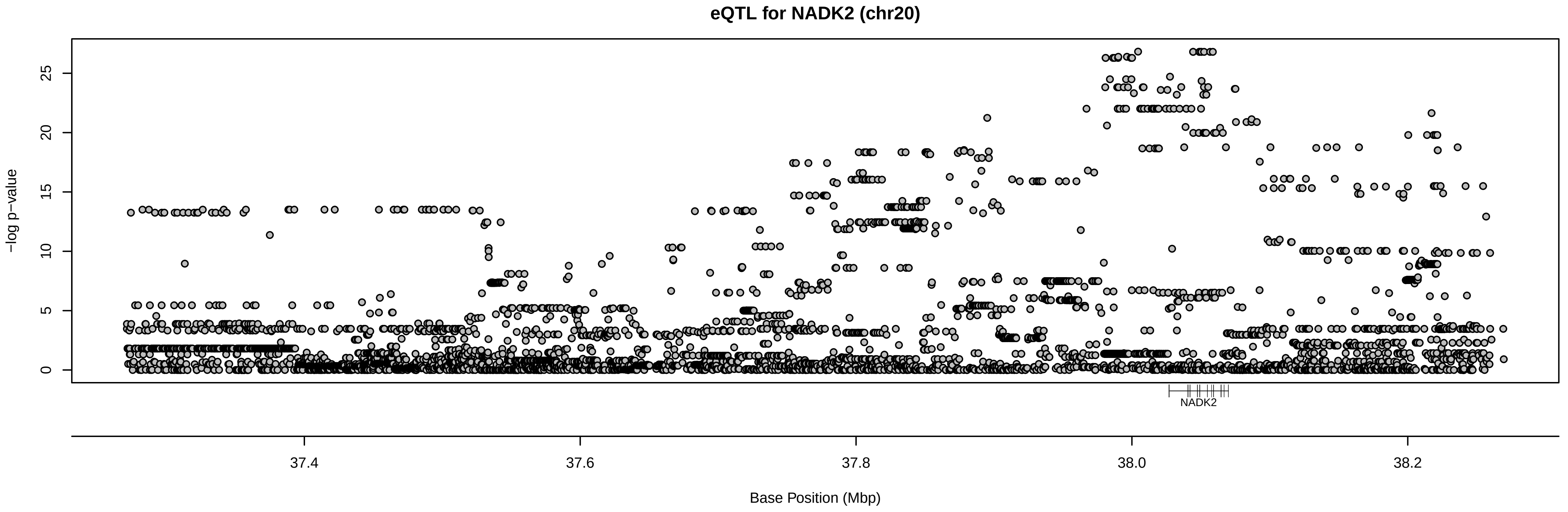
eQTL for NADK2 (chr20)
−log p−value
0
5
10
15
20
25
NADK2
37.4
37.6
37.8
38.0
38.2
Base Position (Mbp)

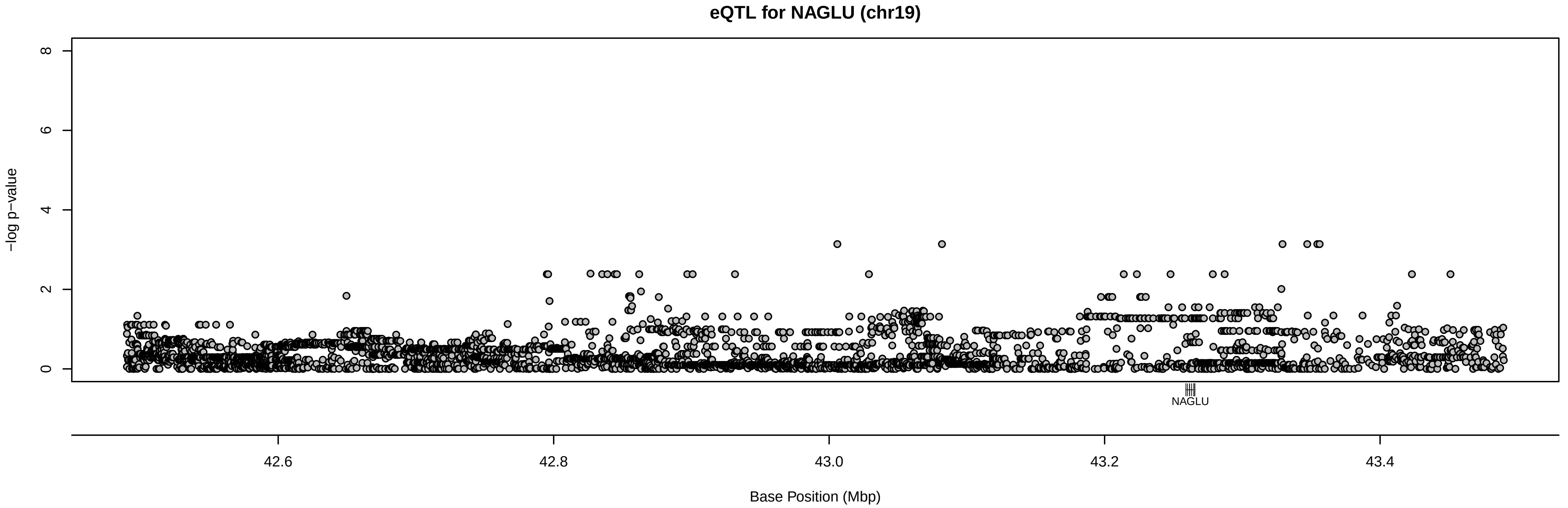
eQTL for NAGLU (chr19)
−log p−value
0
2
4
6
8
NAGLU
42.6
42.8
43.0
43.2
43.4
Base Position (Mbp)

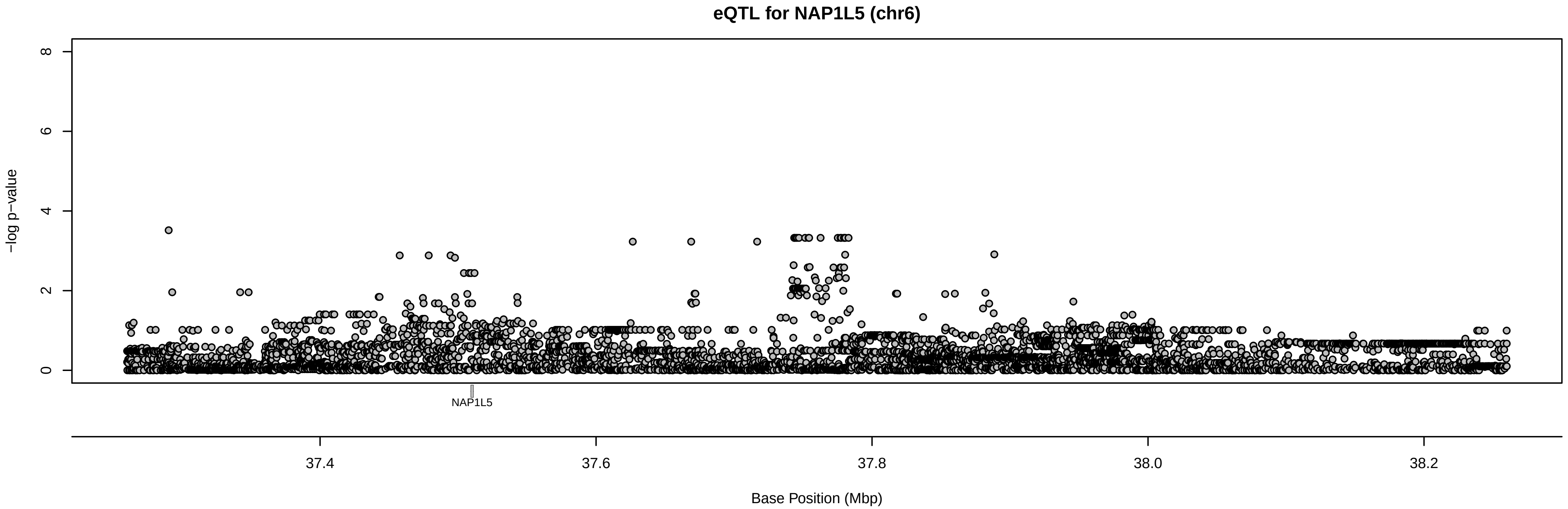
eQTL for NAP1L5 (chr6)
−log p−value
0
2
4
6
8
NAP1L5
37.4
37.6
37.8
38.0
38.2
Base Position (Mbp)

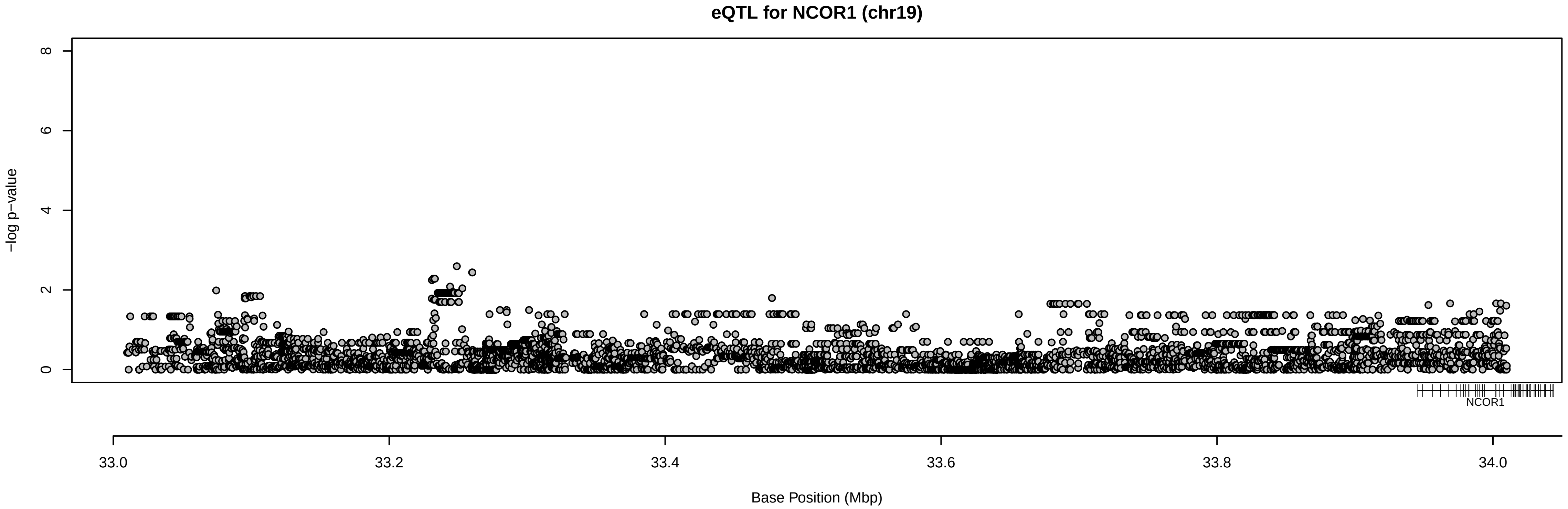
eQTL for NCOR1 (chr19)
−log p−value
0
2
4
6
8
NCOR1
33.0
33.2
33.4
33.6
33.8
34.0
Base Position (Mbp)

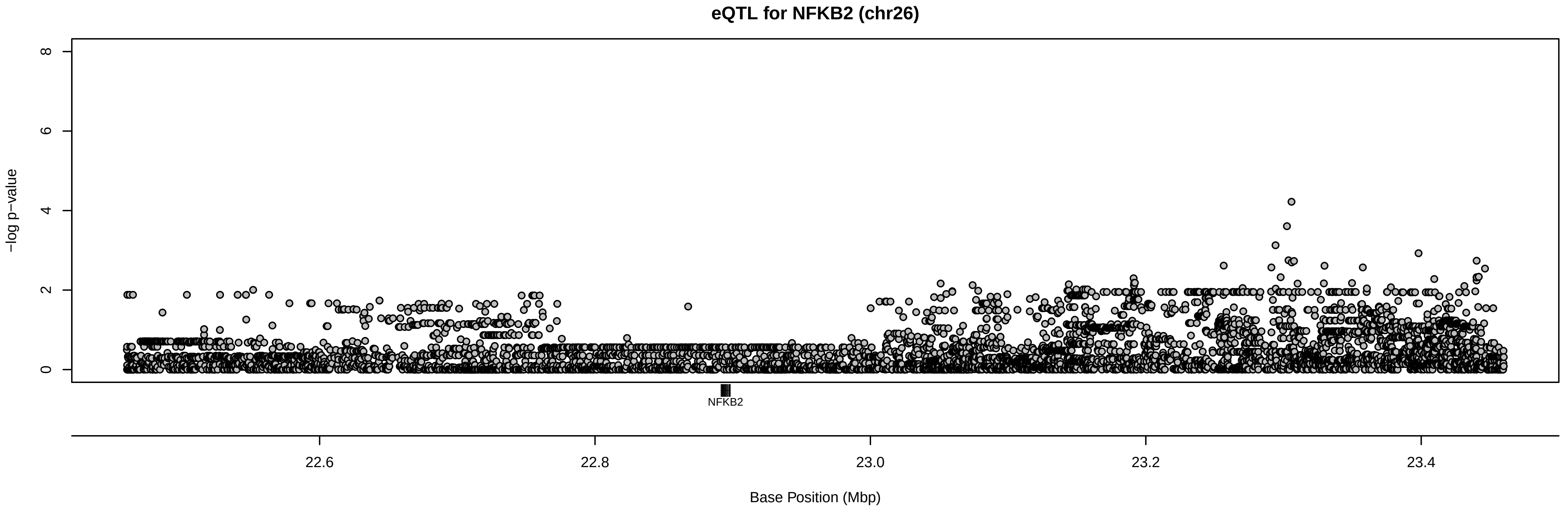
eQTL for NFKB2 (chr26)
−log p−value
0
2
4
6
8
NFKB2
22.6
22.8
23.0
23.2
23.4
Base Position (Mbp)

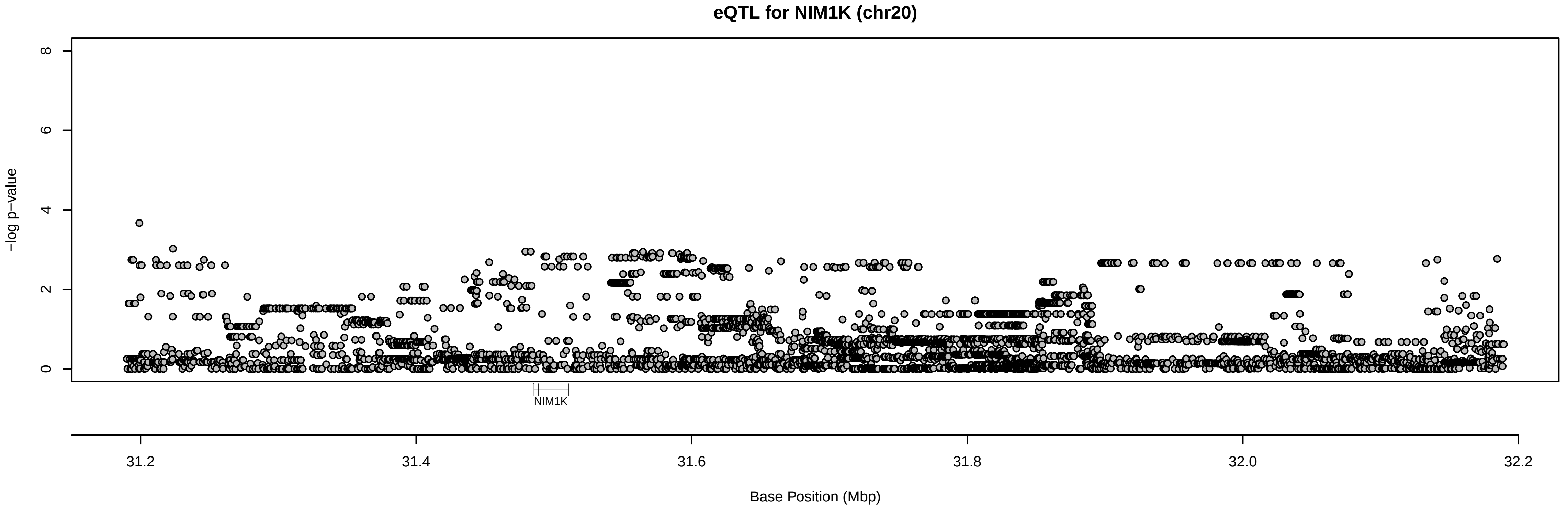
eQTL for NIM1K (chr20)
−log p−value
0
2
4
6
8
NIM1K
31.2
31.4
31.6
31.8
32.0
32.2
Base Position (Mbp)

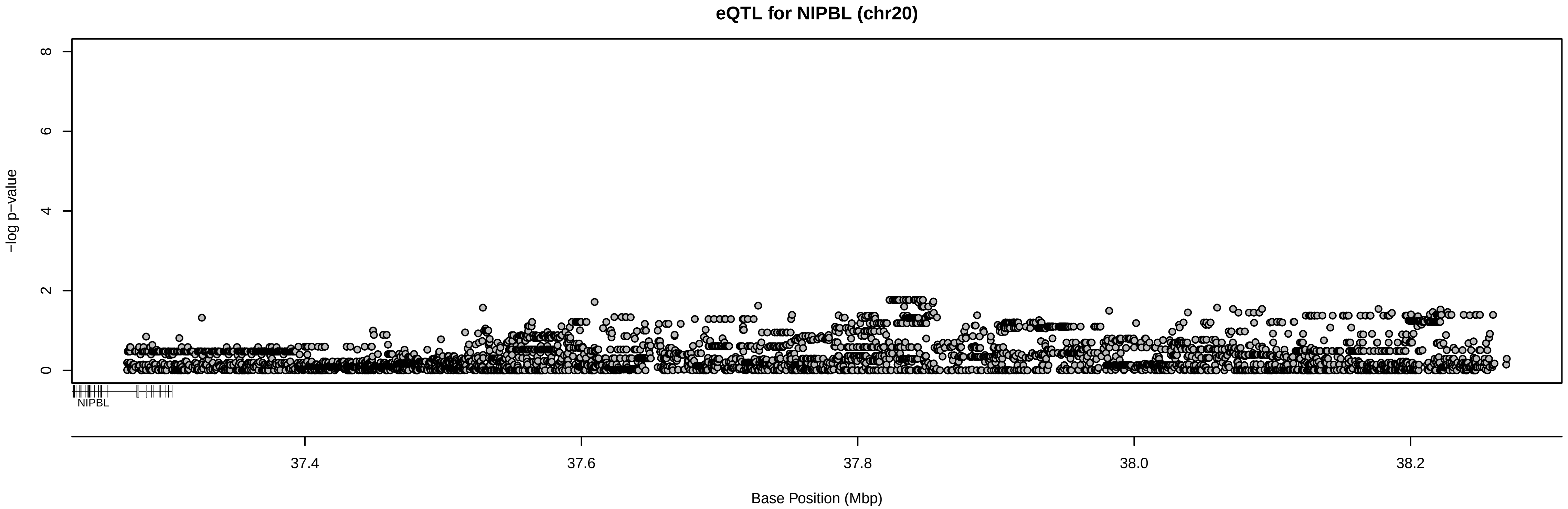
eQTL for NIPBL (chr20)
−log p−value
0
2
4
6
8
NIPBL
37.4
37.6
37.8
38.0
38.2
Base Position (Mbp)

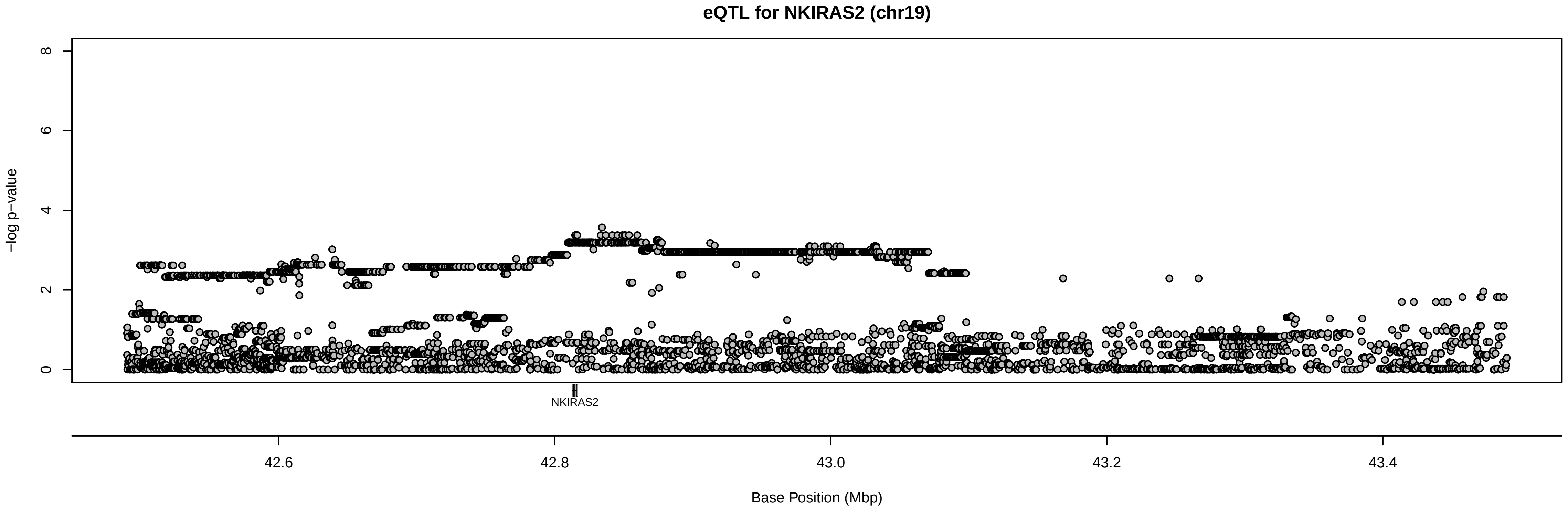
eQTL for NKIRAS2 (chr19)
−log p−value
0
2
4
6
8
NKIRAS2
42.6
42.8
43.0
43.2
43.4
Base Position (Mbp)

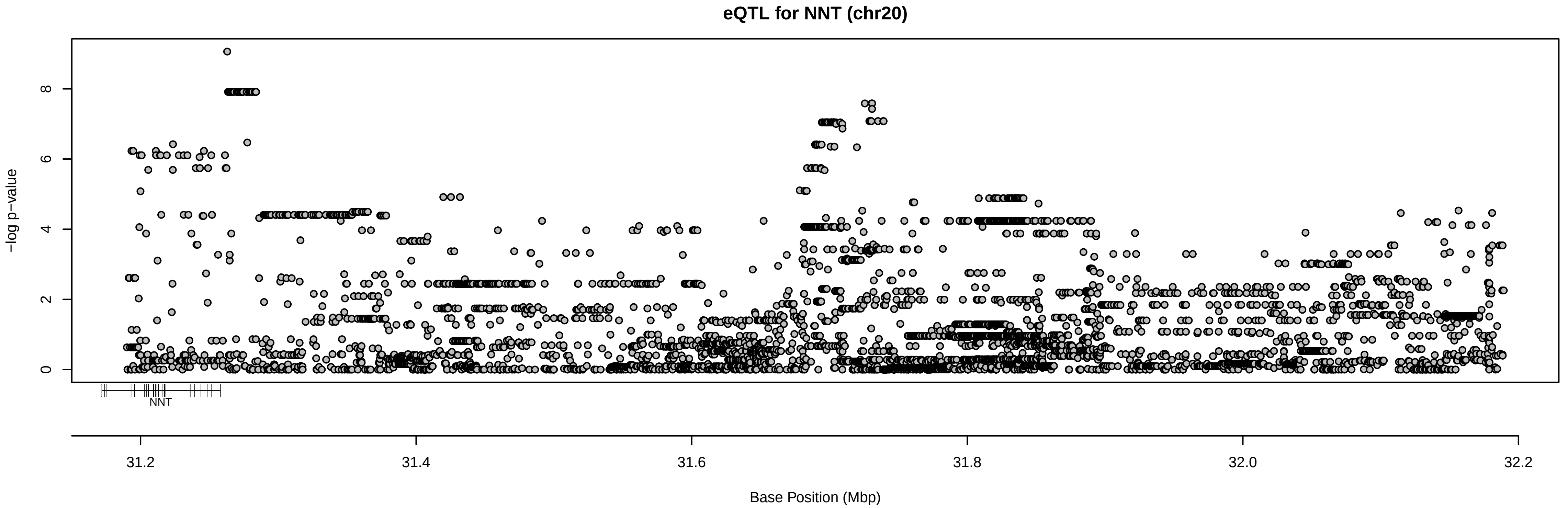
eQTL for NNT (chr20)
−log p−value
0
2
4
6
8
NNT
31.2
31.4
31.6
31.8
32.0
32.2
Base Position (Mbp)

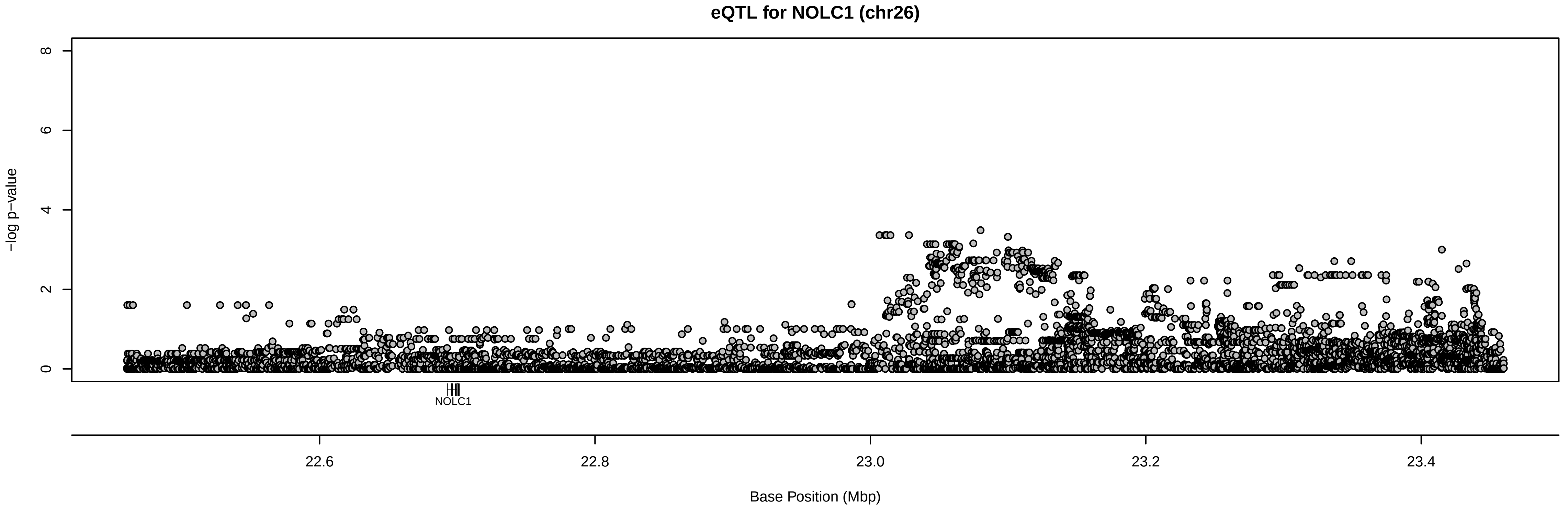
eQTL for NOLC1 (chr26)
−log p−value
0
2
4
6
8
NOLC1
22.6
22.8
23.0
23.2
23.4
Base Position (Mbp)

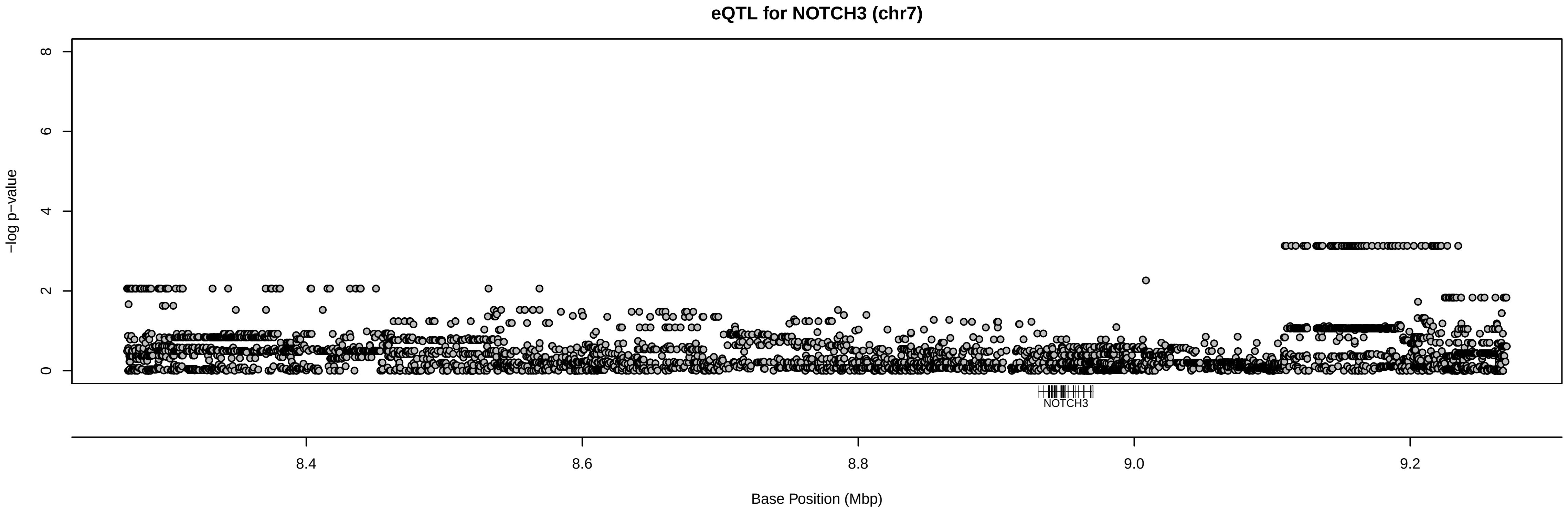
eQTL for NOTCH3 (chr7)
−log p−value
0
2
4
6
8
NOTCH3
8.4
8.6
8.8
9.0
9.2
Base Position (Mbp)

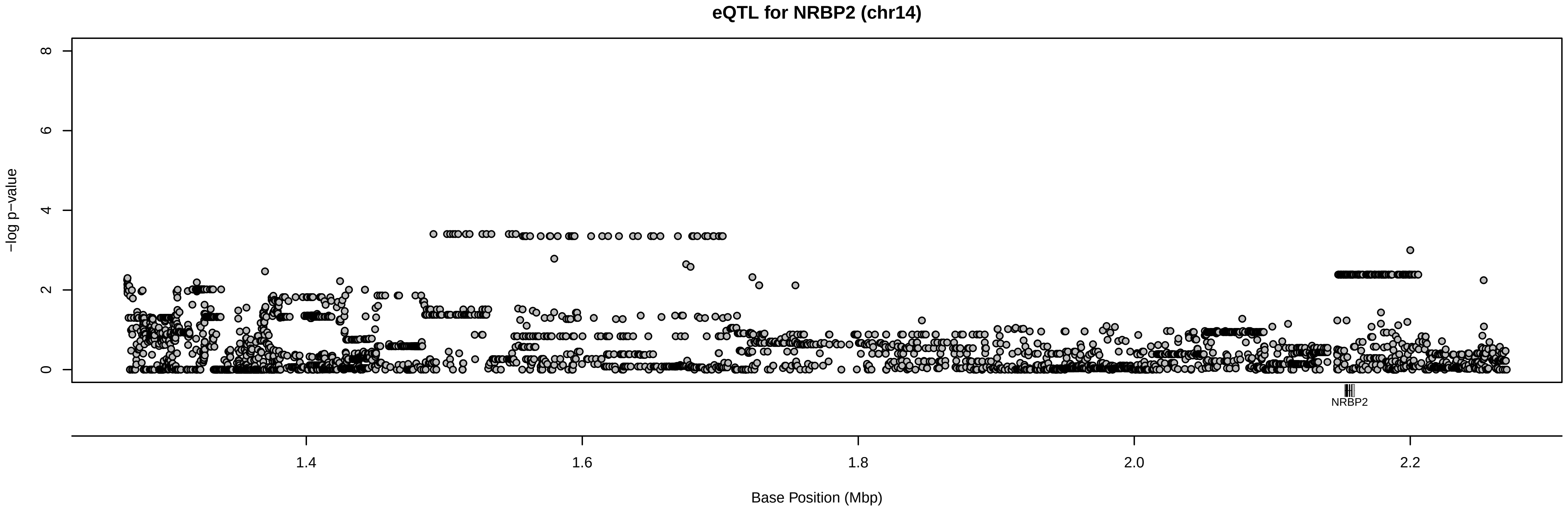
eQTL for NRBP2 (chr14)
−log p−value
0
2
4
6
8
NRBP2
1.4
1.6
1.8
2.0
2.2
Base Position (Mbp)

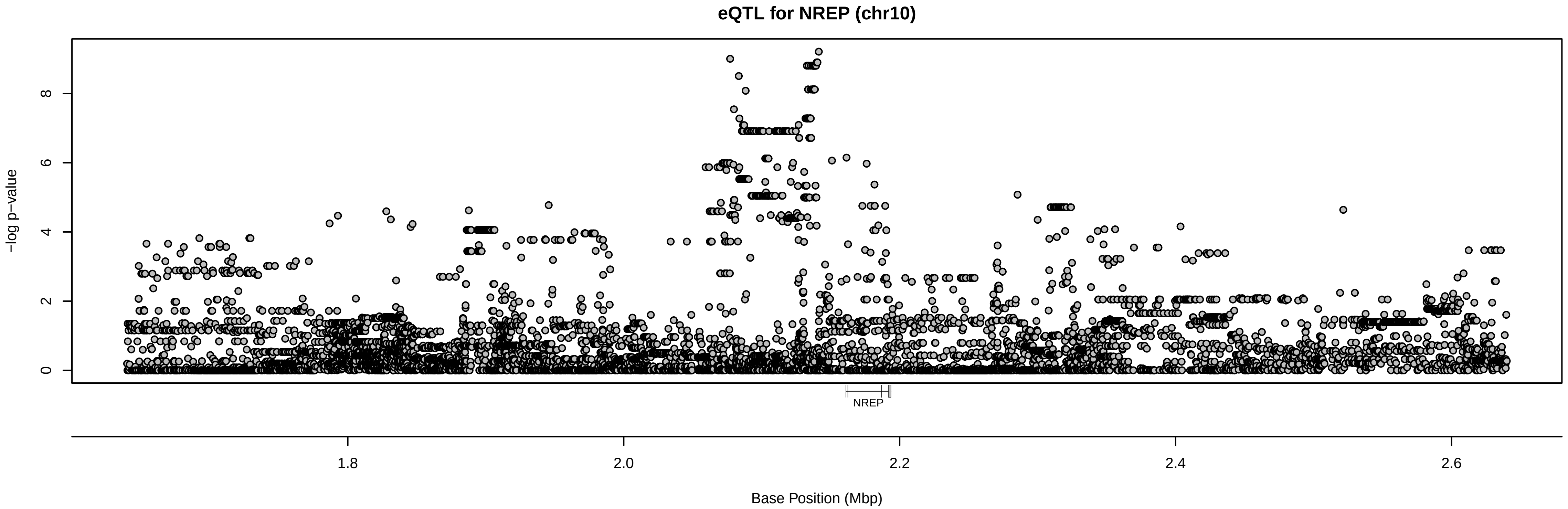

eQTL for NREP (chr10)
−log p−value
0
2
4
6
8
NREP
1.8
2.0
2.2
2.4
2.6
Base Position (Mbp)

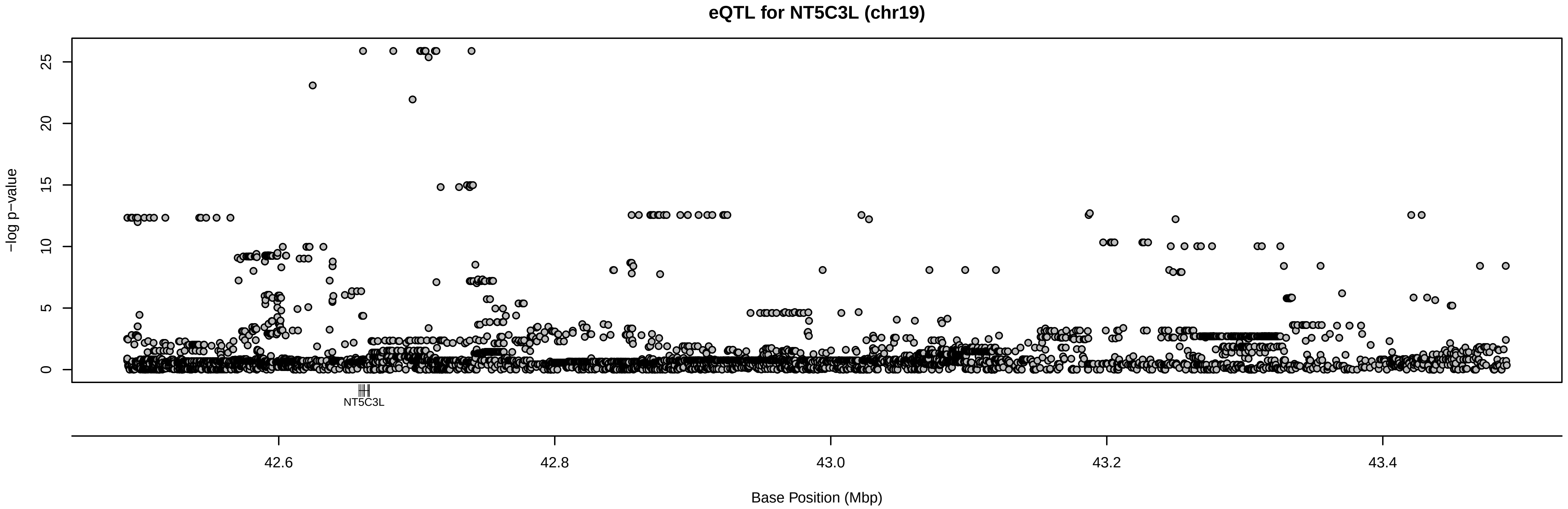

eQTL for NT5C3L (chr19)
−log p−value
0
5
10
15
20
25
NT5C3L
42.6
42.8
43.0
43.2
43.4
Base Position (Mbp)

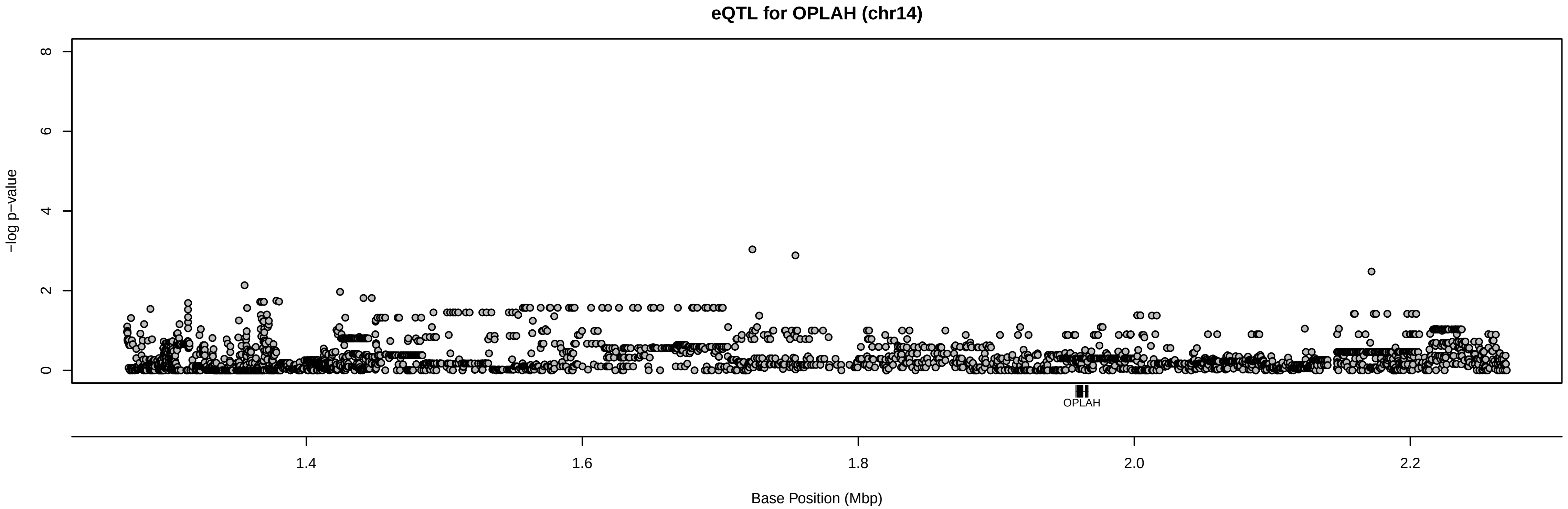
eQTL for OPLAH (chr14)
−log p−value
8
6
4
2
0
OPLAH
1.4
1.6
1.8
2.0
2.2
Base Position (Mbp)

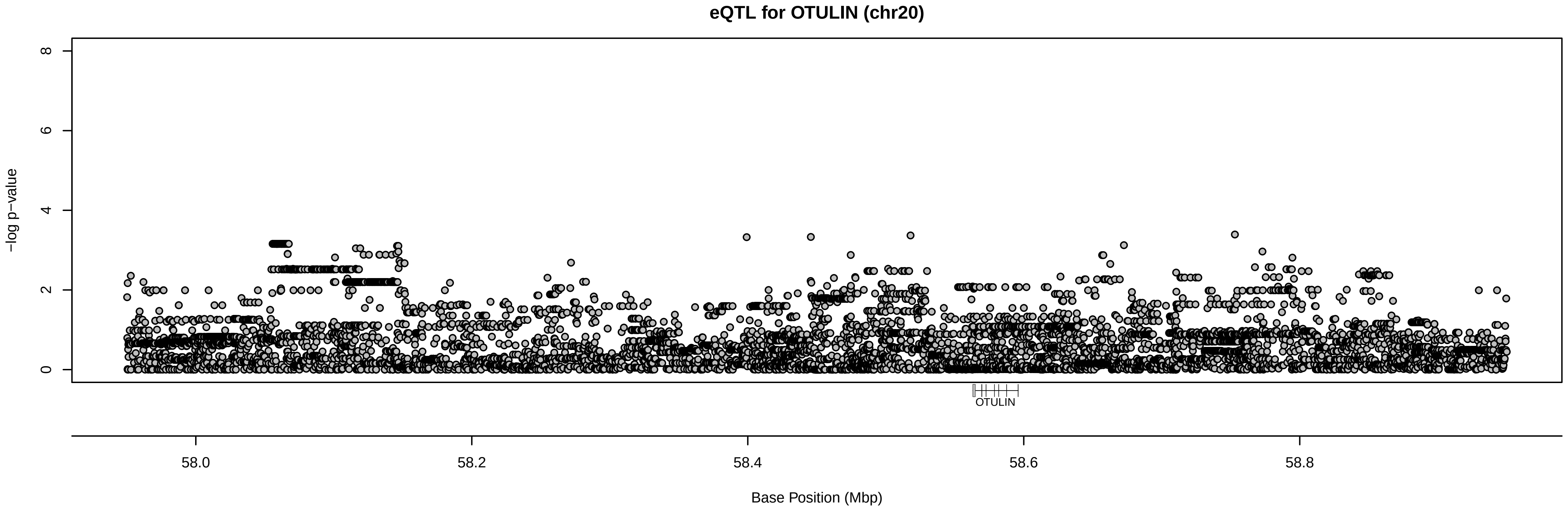
eQTL for OTULIN (chr20)
−log p−value
8
6
4
2
0
OTULIN
58.0
58.2
58.4
58.6
58.8
Base Position (Mbp)

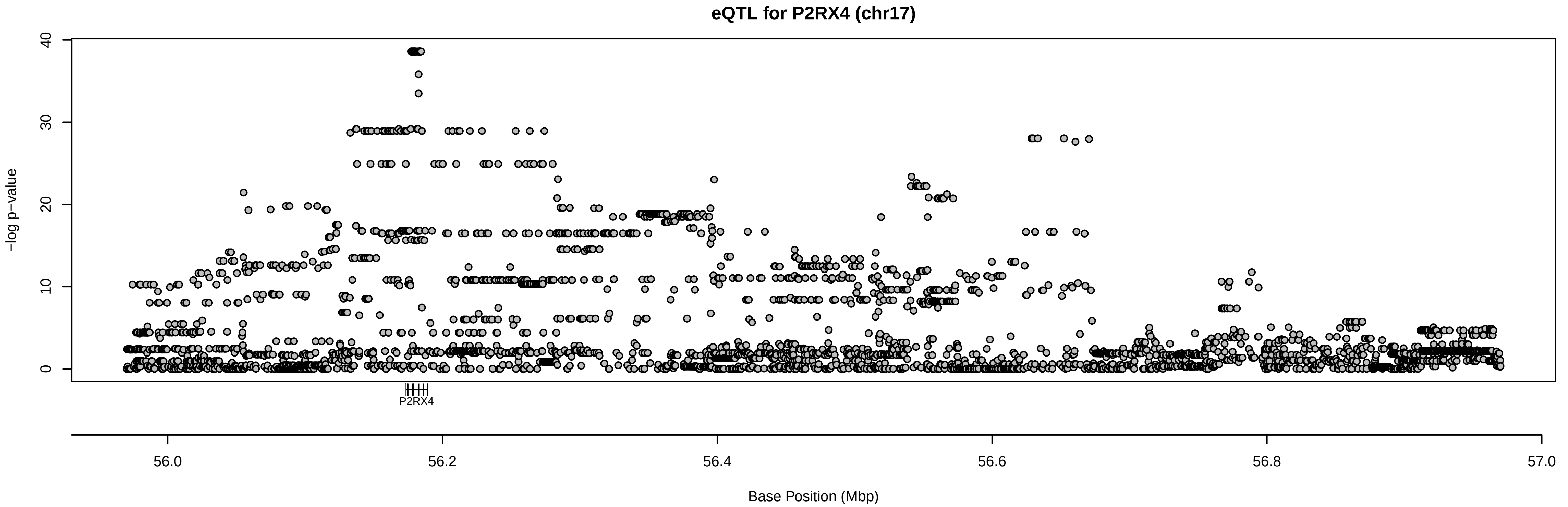
eQTL for P2RX4 (chr17)
−log p−value
0
10
20
30
40
P2RX4
56.0
56.2
56.4
56.6
56.8
57.0
Base Position (Mbp)

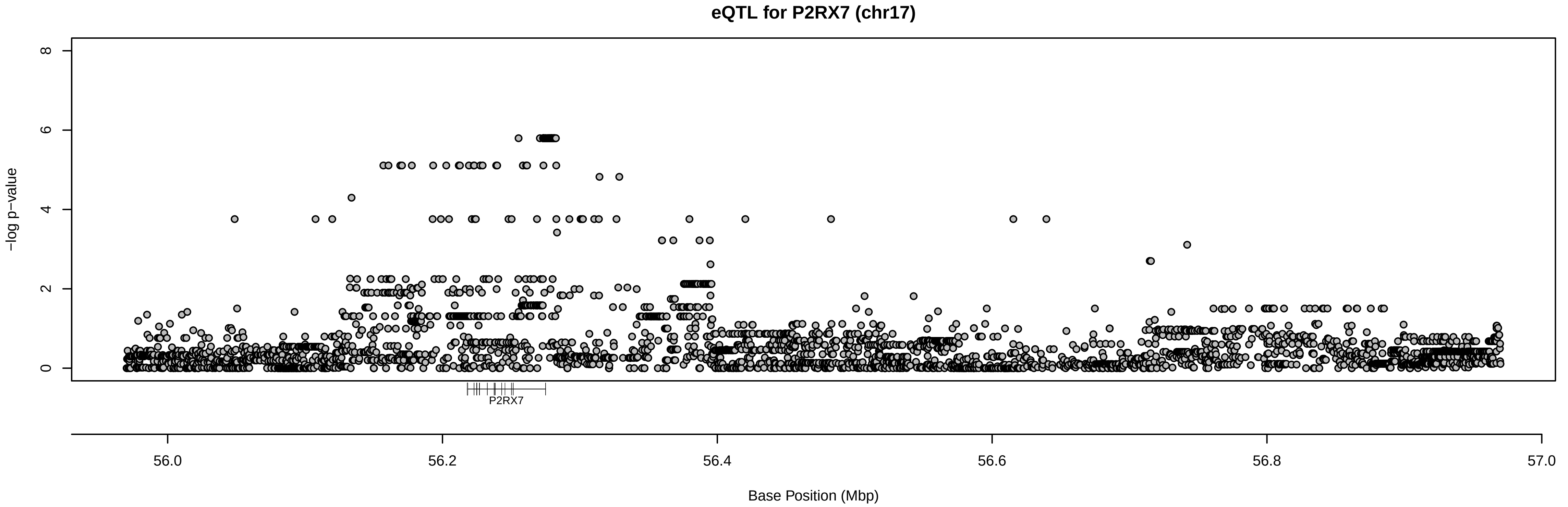
eQTL for P2RX7 (chr17)
−log p−value
0
2
4
6
8
P2RX7
56.0
56.2
56.4
56.6
56.8
57.0
Base Position (Mbp)

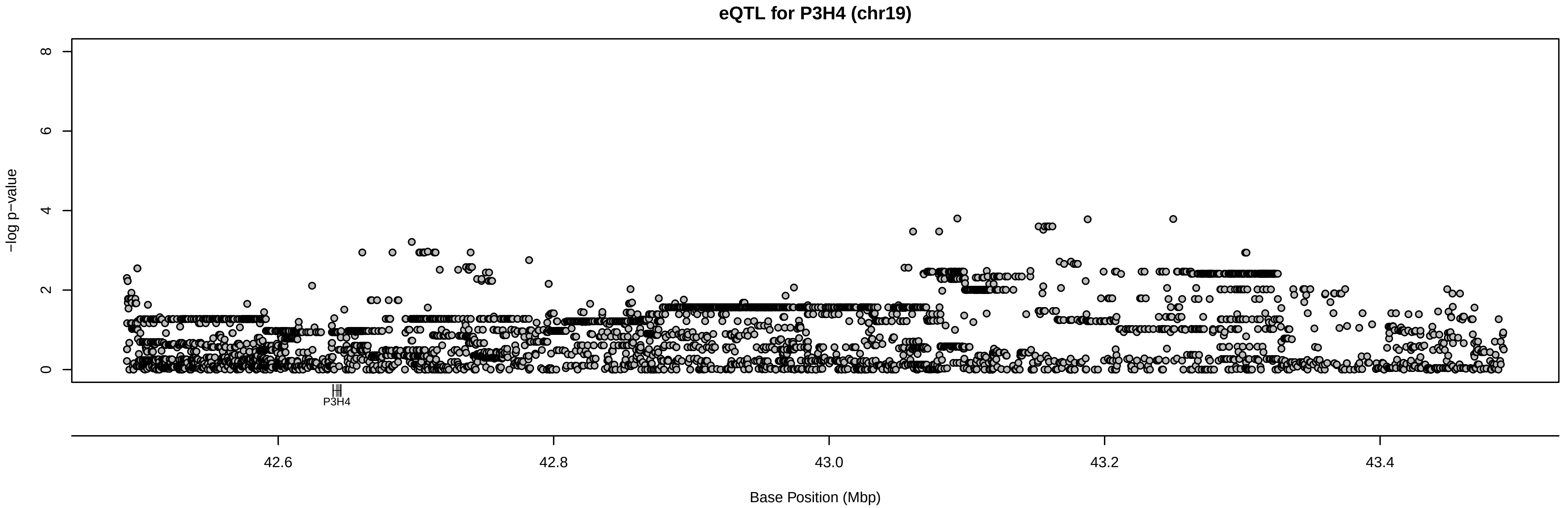
eQTL for P3H4 (chr19)
−log p−value
0
2
4
6
8
P3H4
42.6
42.8
43.0
43.2
43.4
Base Position (Mbp)

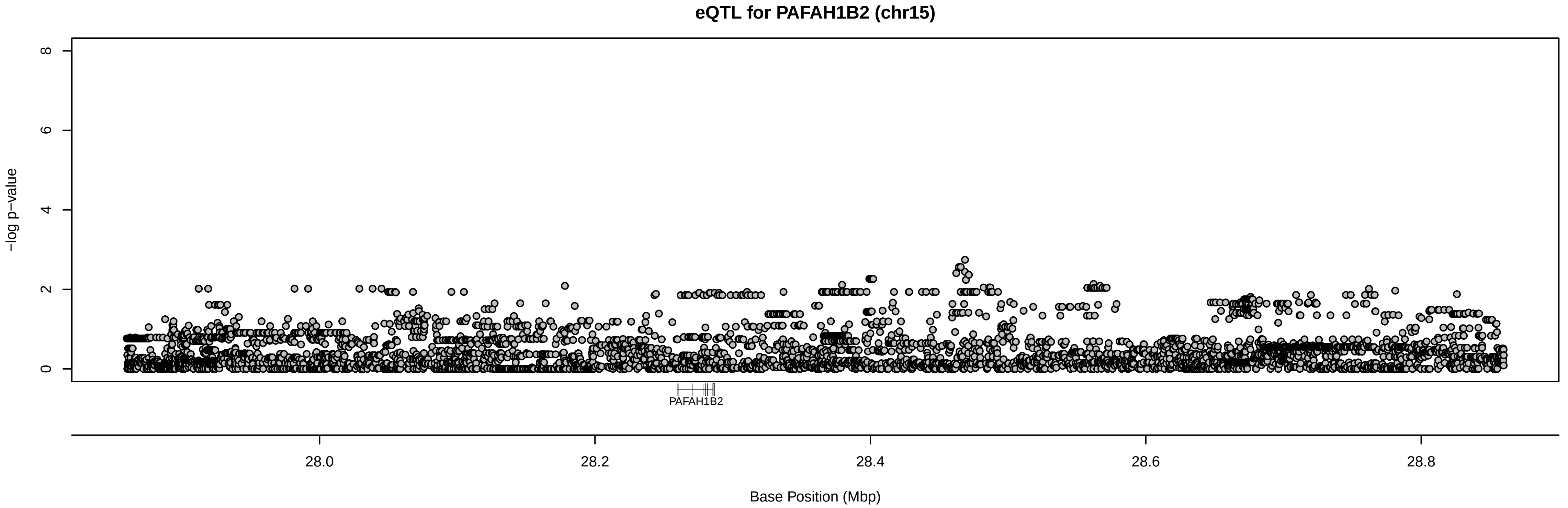
eQTL for PAFAH1B2 (chr15)
−log p−value
0
2
4
6
8
PAFAH1B2
28.0
28.2
28.4
28.6
28.8
Base Position (Mbp)

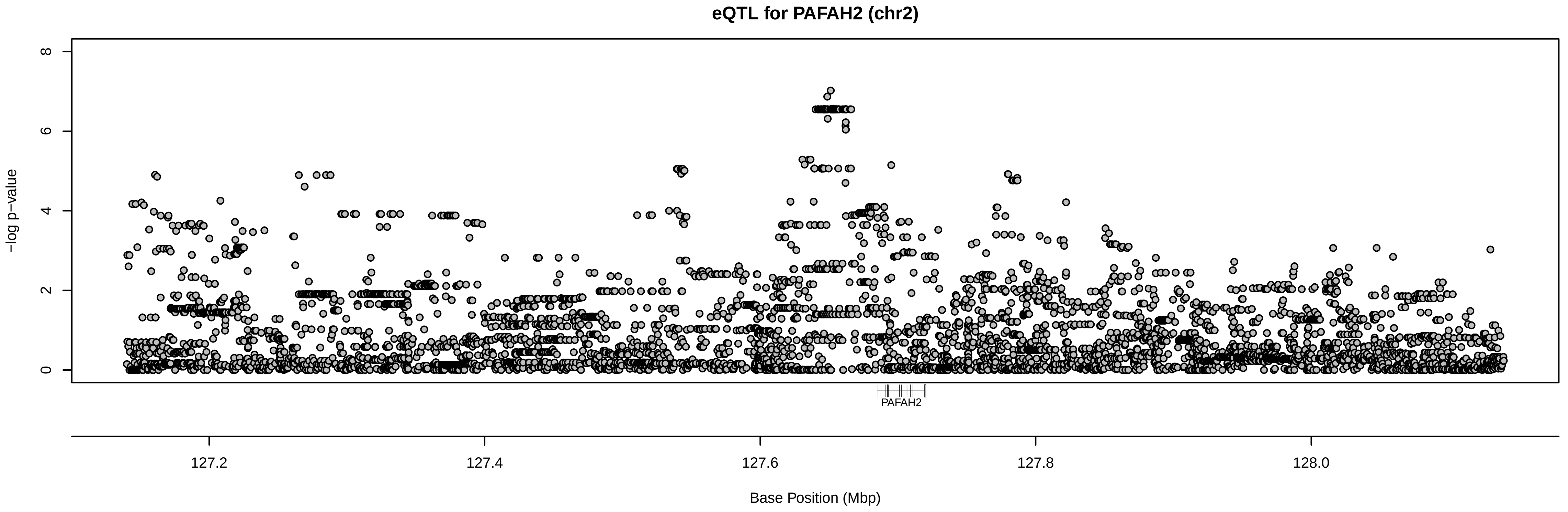

eQTL for PAFAH2 (chr2)
−log p−value
0
2
4
6
8
PAFAH2
127.2
127.4
127.6
127.8
128.0
Base Position (Mbp)

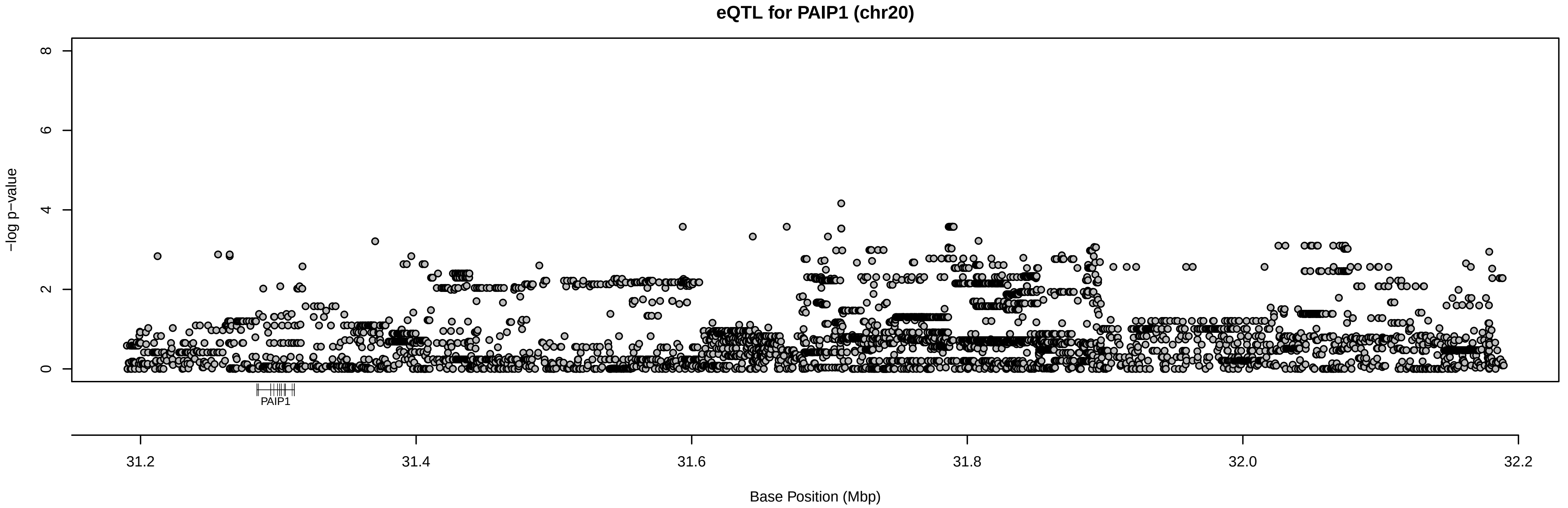

eQTL for PAIP1 (chr20)
−log p−value
0
2
4
6
8
PAIP1
31.2
31.4
31.6
31.8
32.0
32.2
Base Position (Mbp)

eQTL for PAQR7 (chr2)

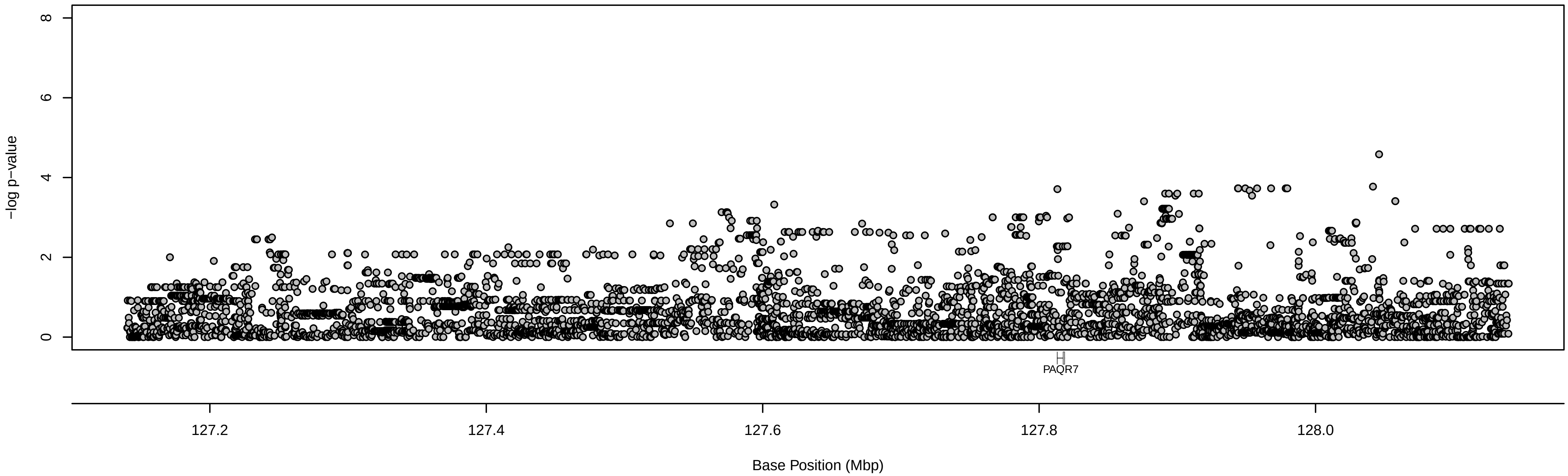

eQTL for PARP10 (chr14)

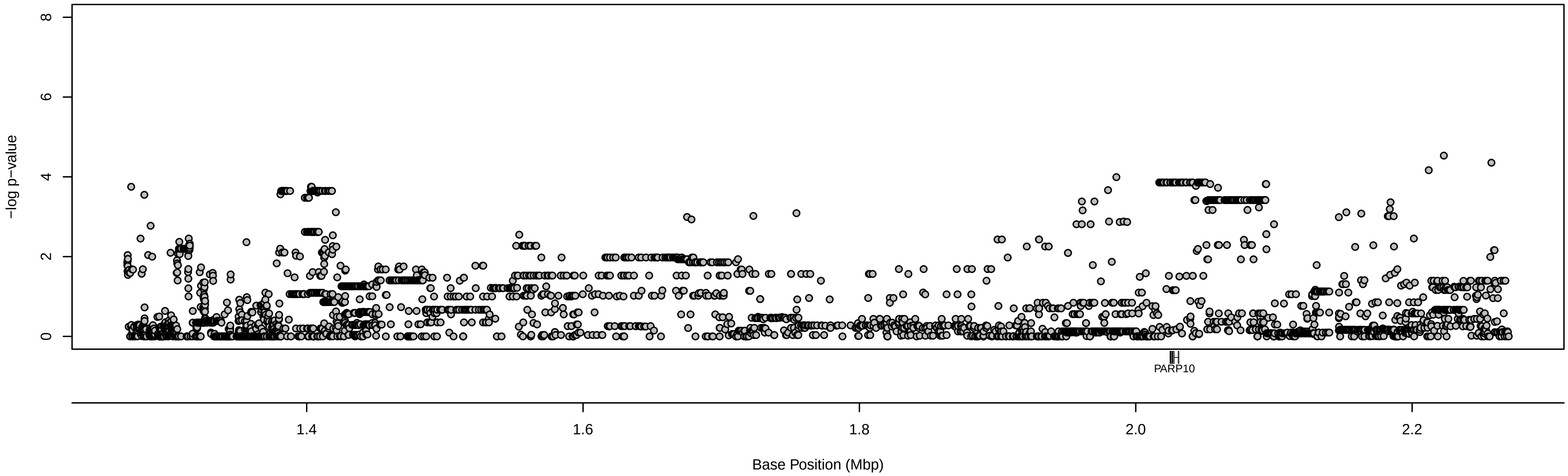

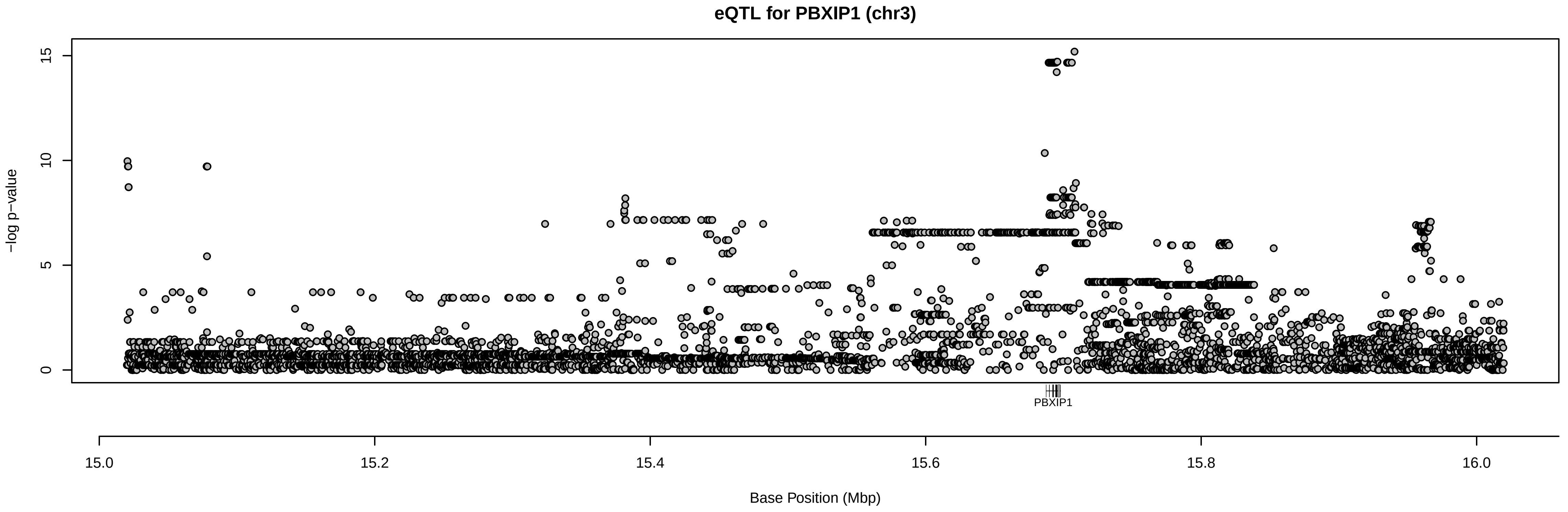

eQTL for PBXIP1 (chr3)
−log p−value
0
5
10
15
PBXIP1
15.0
15.2
15.4
15.6
15.8
16.0
Base Position (Mbp)

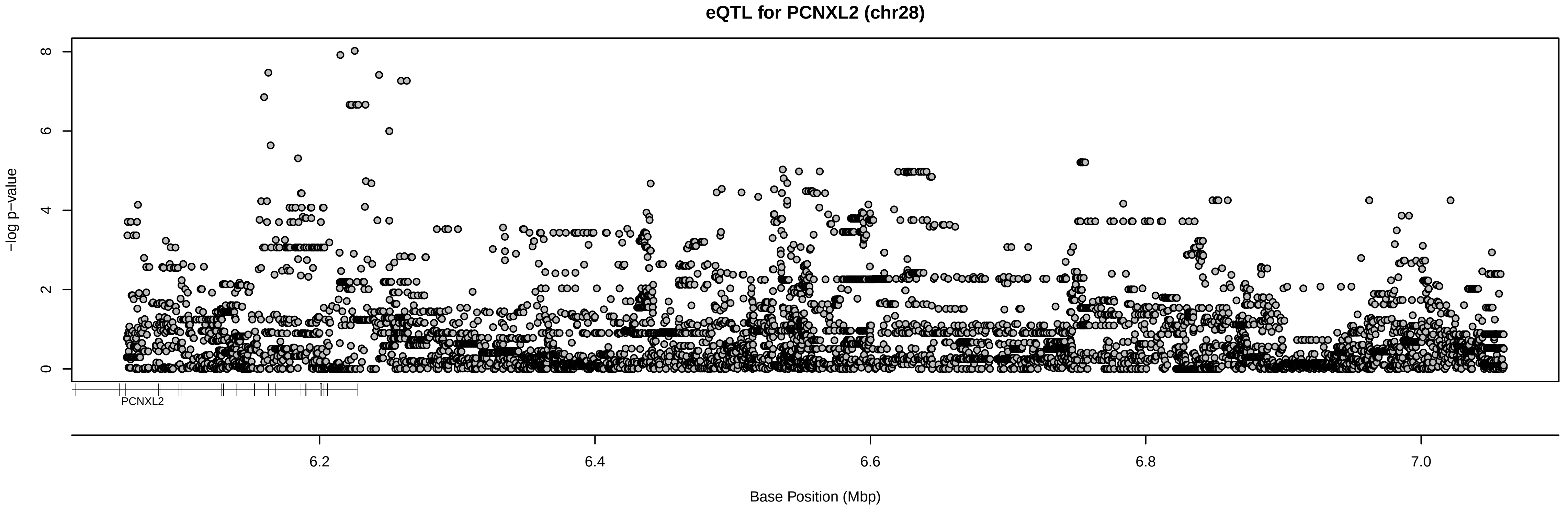

eQTL for PCNXL2 (chr28)
−log p−value
0
2
4
6
8
PCNXL2
6.2
6.4
6.6
6.8
7.0
Base Position (Mbp)

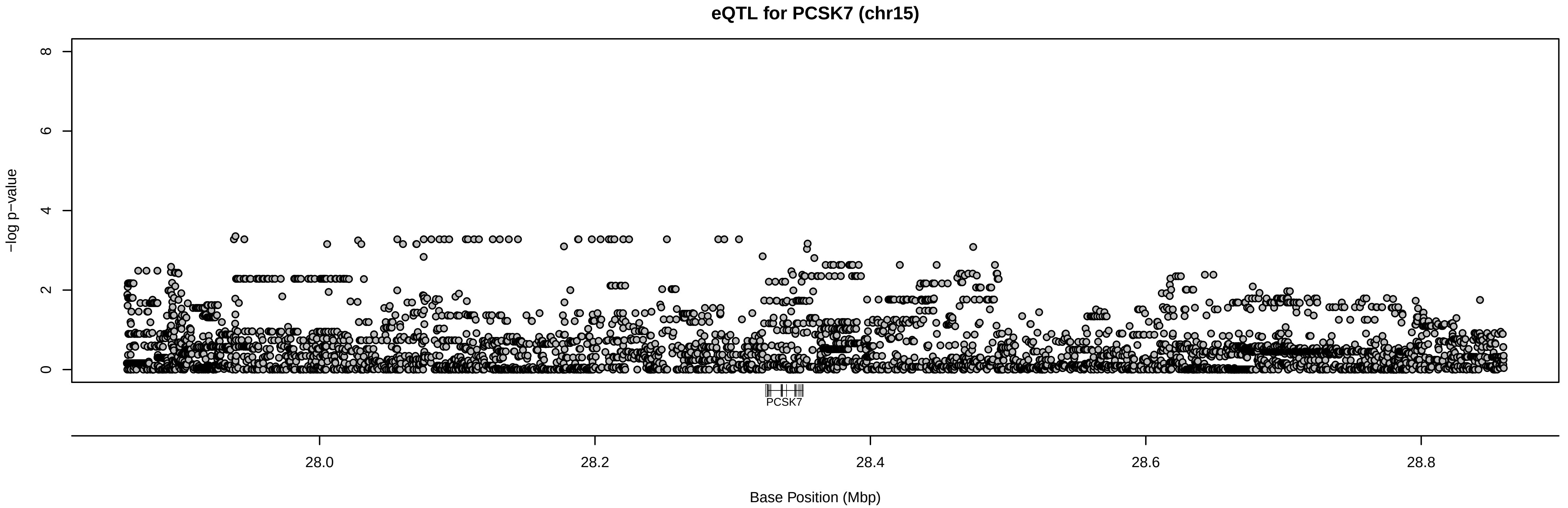
eQTL for PCSK7 (chr15)
−log p−value
0
2
4
6
8
PCSK7
28.0
28.2
28.4
28.6
28.8
Base Position (Mbp)

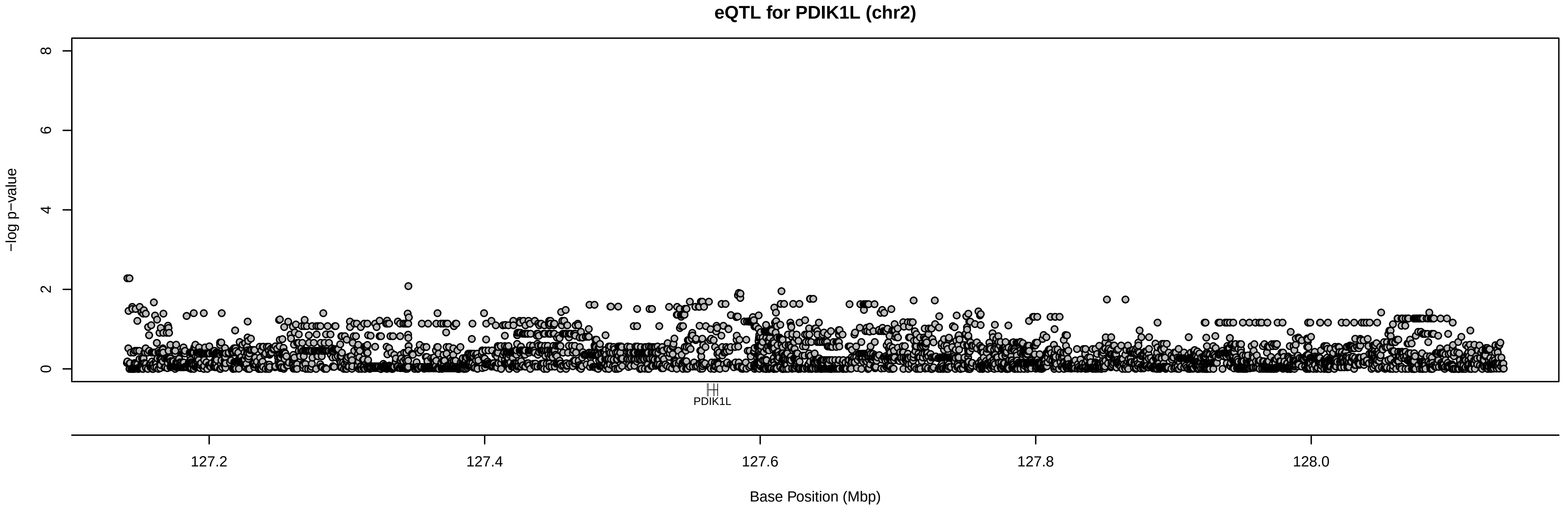
eQTL for PDIK1L (chr2)
−log p−value
0
2
4
6
8
PDIK1L
127.2
127.4
127.6
127.8
128.0
Base Position (Mbp)

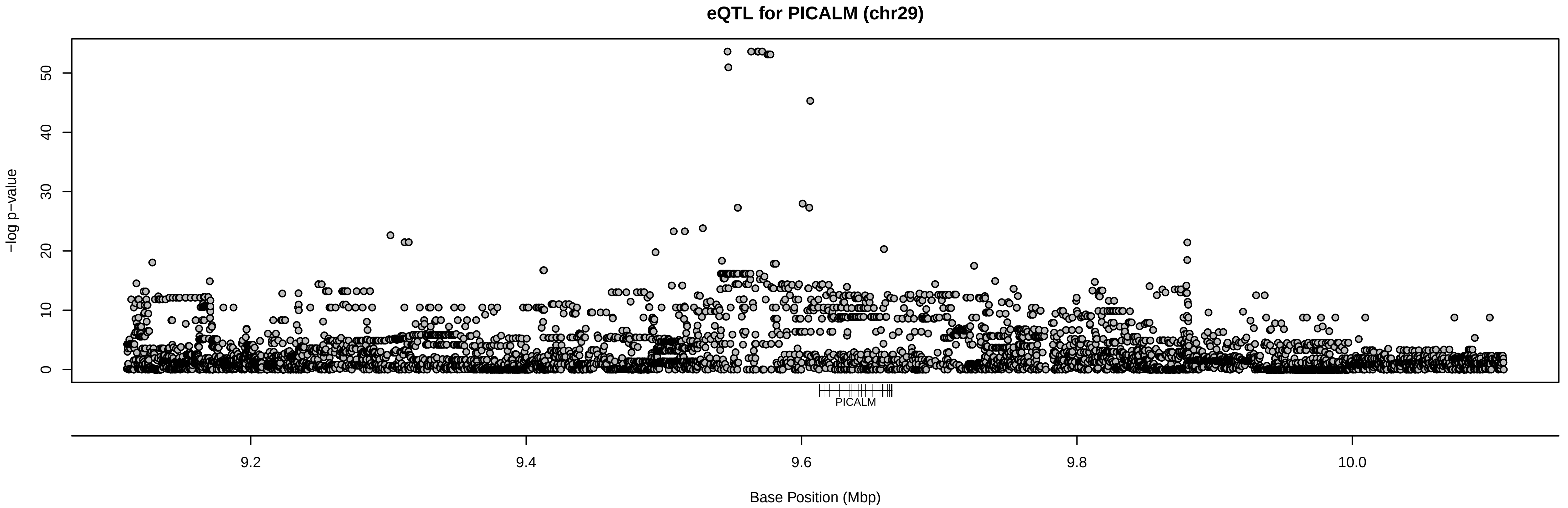

eQTL for PICALM (chr29)
−log p−value
0
10
20
30
40
50
PICALM
9.2
9.4
9.6
9.8
10.0
Base Position (Mbp)

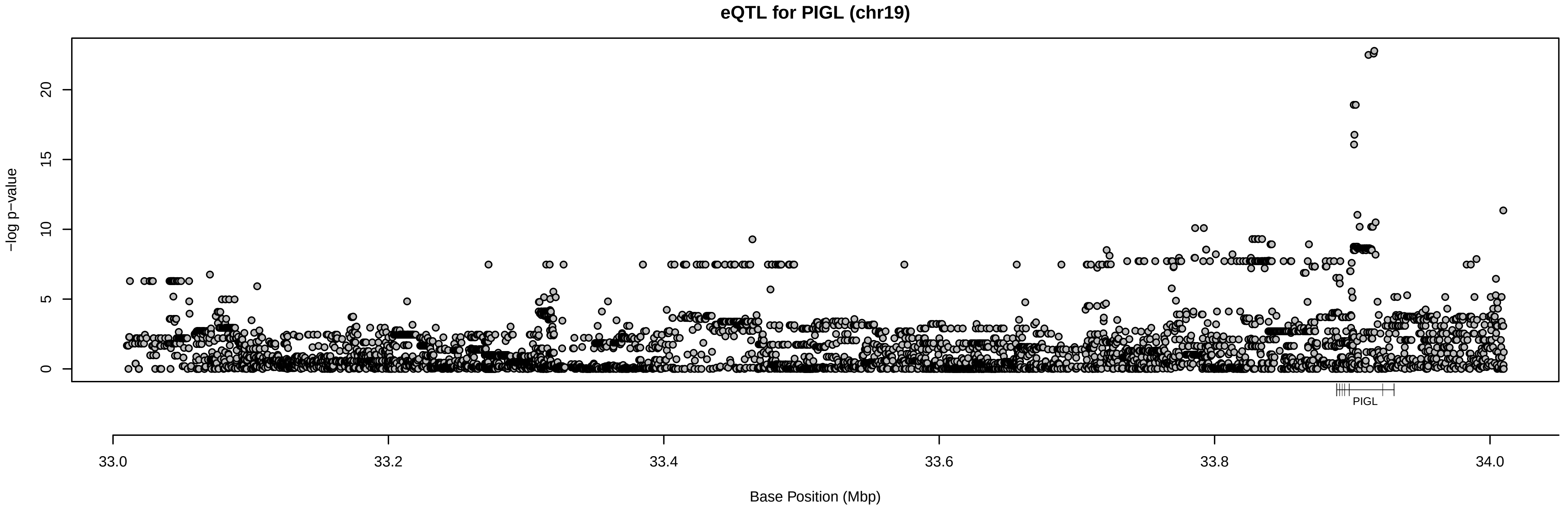

eQTL for PIGL (chr19)
−log p−value
0
5
10
15
20
PIGL
33.0
33.2
33.4
33.6
33.8
34.0
Base Position (Mbp)

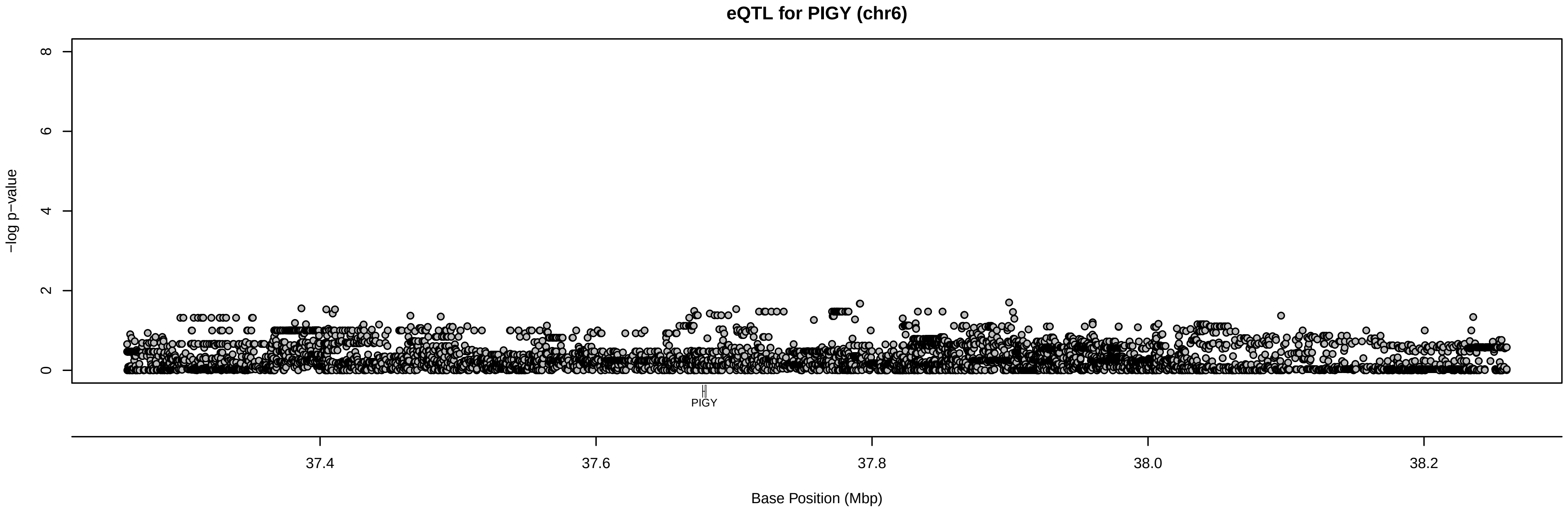
eQTL for PIGY (chr6)
−log p−value
0
2
4
6
8
PIGY
37.4
37.6
37.8
38.0
38.2
Base Position (Mbp)

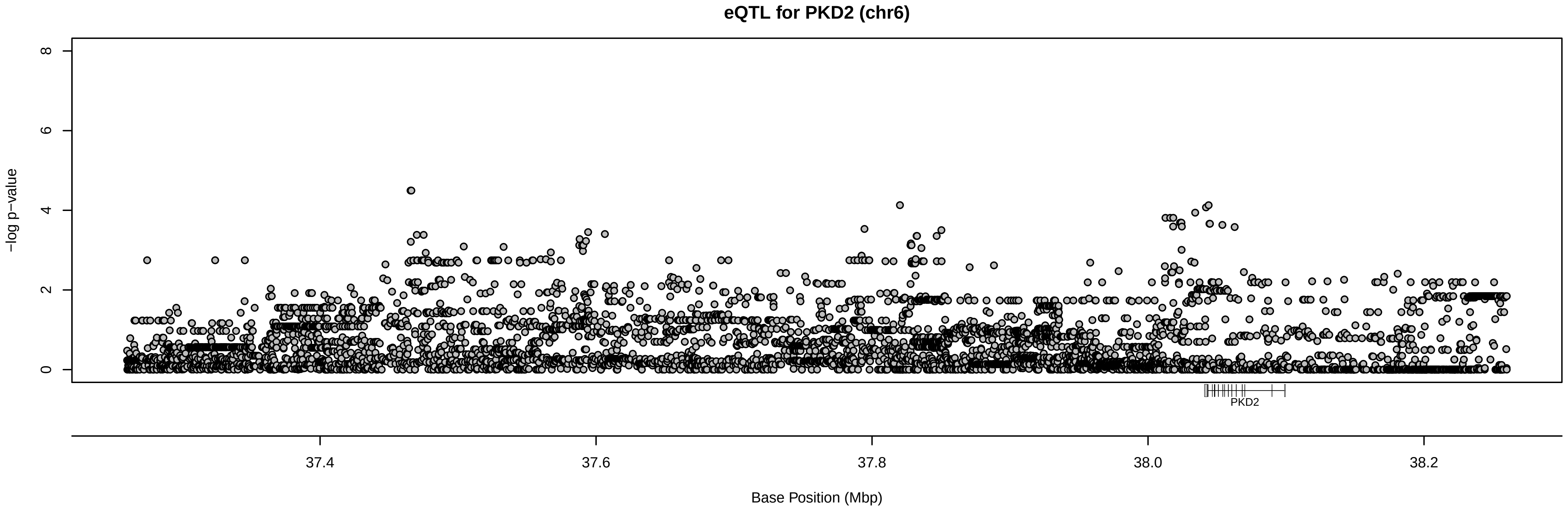
eQTL for PKD2 (chr6)
−log p−value
0
2
4
6
8
PKD2
37.4
37.6
37.8
38.0
38.2
Base Position (Mbp)

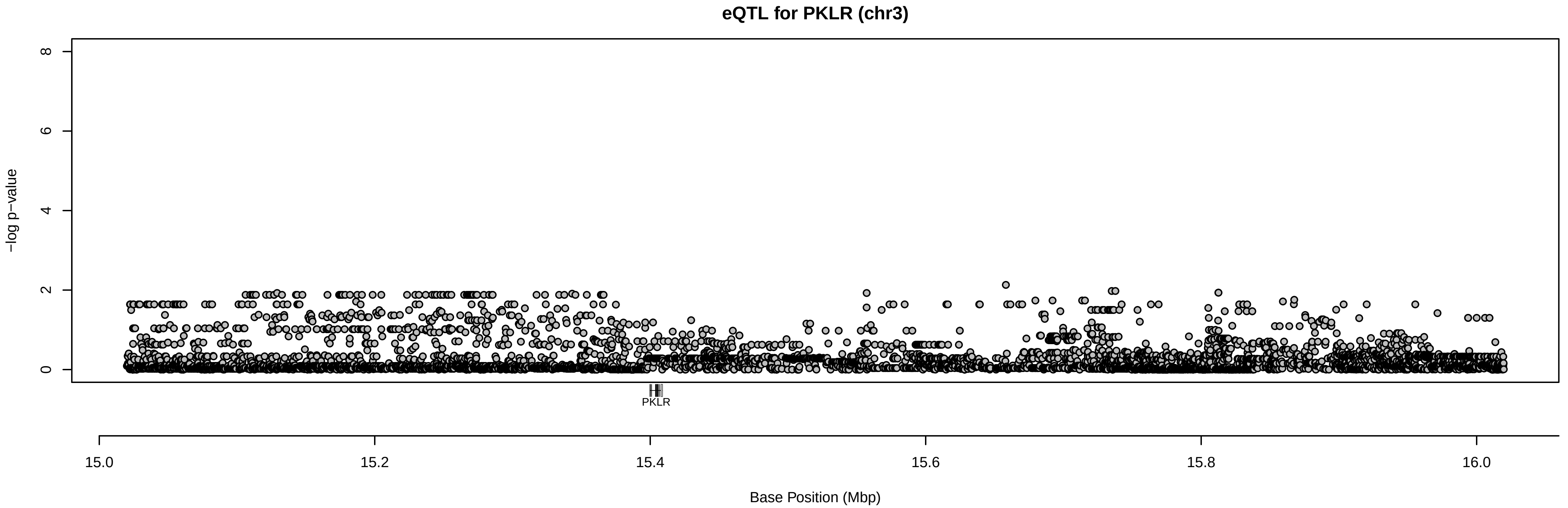
eQTL for PKLR (chr3)
−log p−value
0
2
4
6
8
PKLR
15.0
15.2
15.4
15.6
15.8
16.0
Base Position (Mbp)

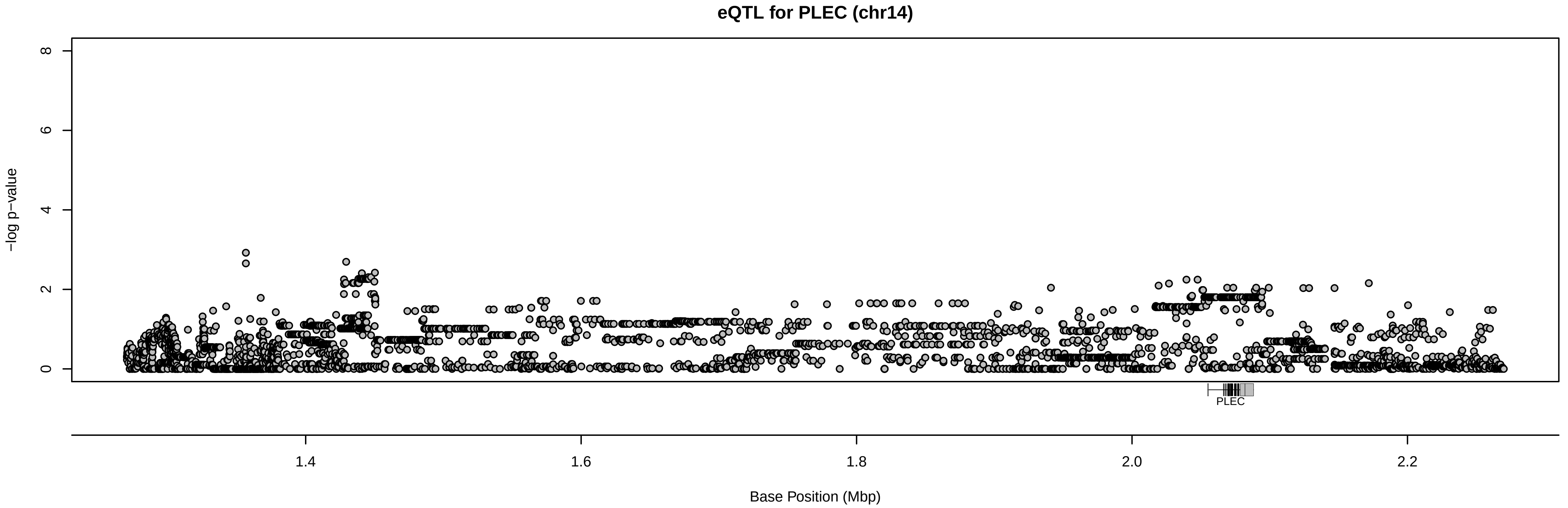
eQTL for PLEC (chr14)
−log p−value
0
2
4
6
8
PLEC
1.4
1.6
1.8
2.0
2.2
Base Position (Mbp)

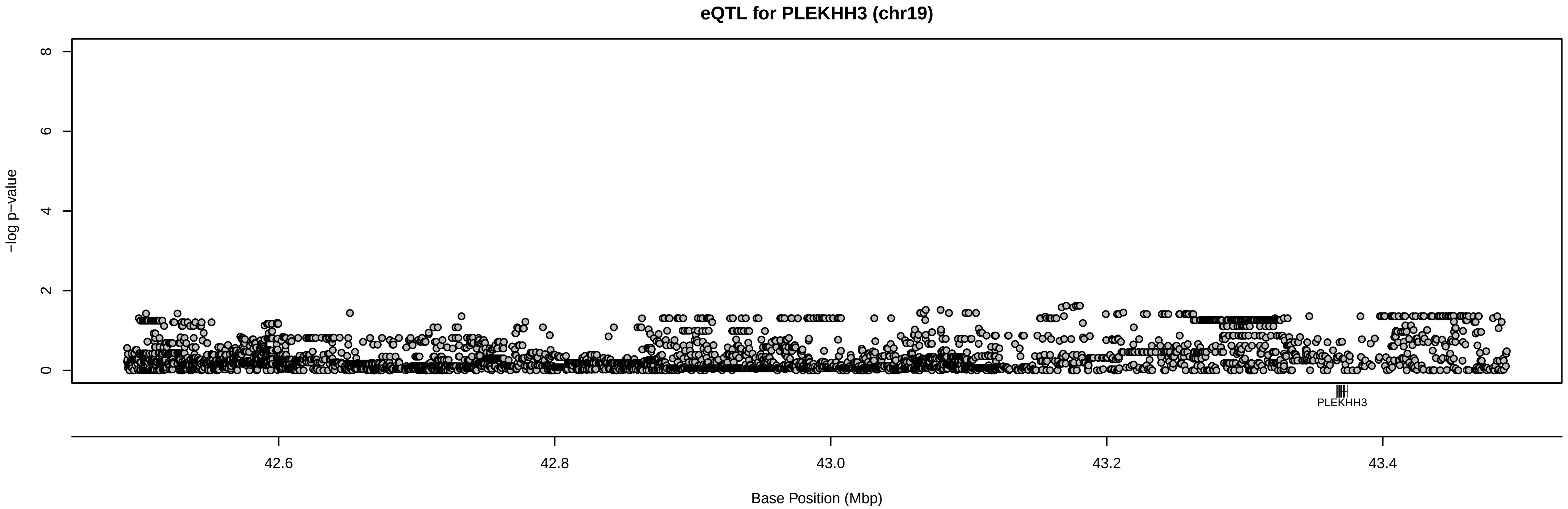
eQTL for PLEKHH3 (chr19)
−log p−value
0
2
4
6
8
PLEKHH3
42.6
42.8
43.0
43.2
43.4
Base Position (Mbp)

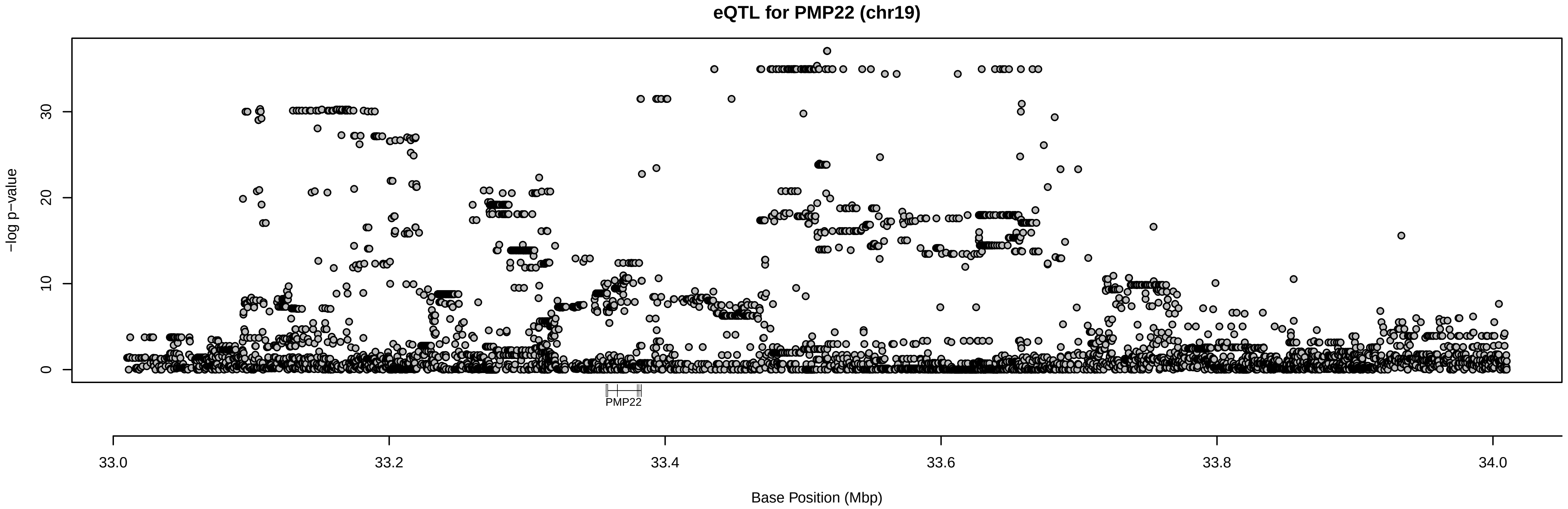
eQTL for PMP22 (chr19)
−log p−value
0
10
20
30
PMP22
33.0
33.2
33.4
33.6
33.8
34.0
Base Position (Mbp)

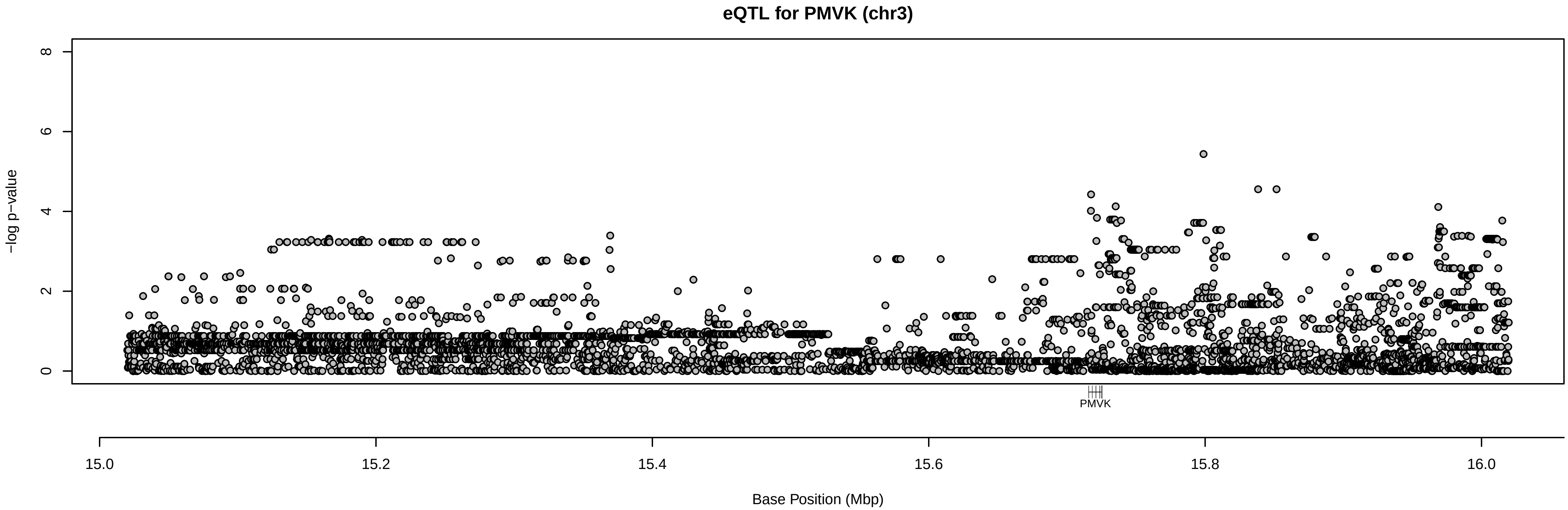
eQTL for PMVK (chr3)
−log p−value
0
2
4
6
8
PMVK
15.0
15.2
15.4
15.6
15.8
16.0
Base Position (Mbp)

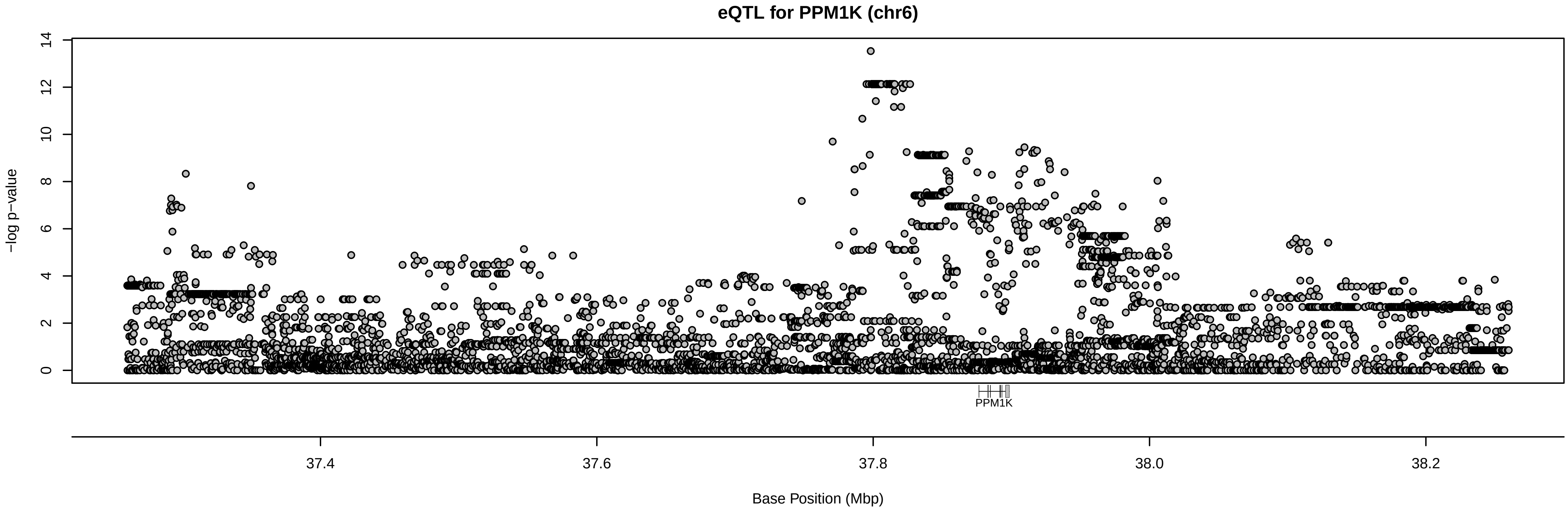
eQTL for PPM1K (chr6)
−log p−value
0
2
4
6
8
10
12
14
PPM1K
37.4
37.6
37.8
38.0
38.2
Base Position (Mbp)

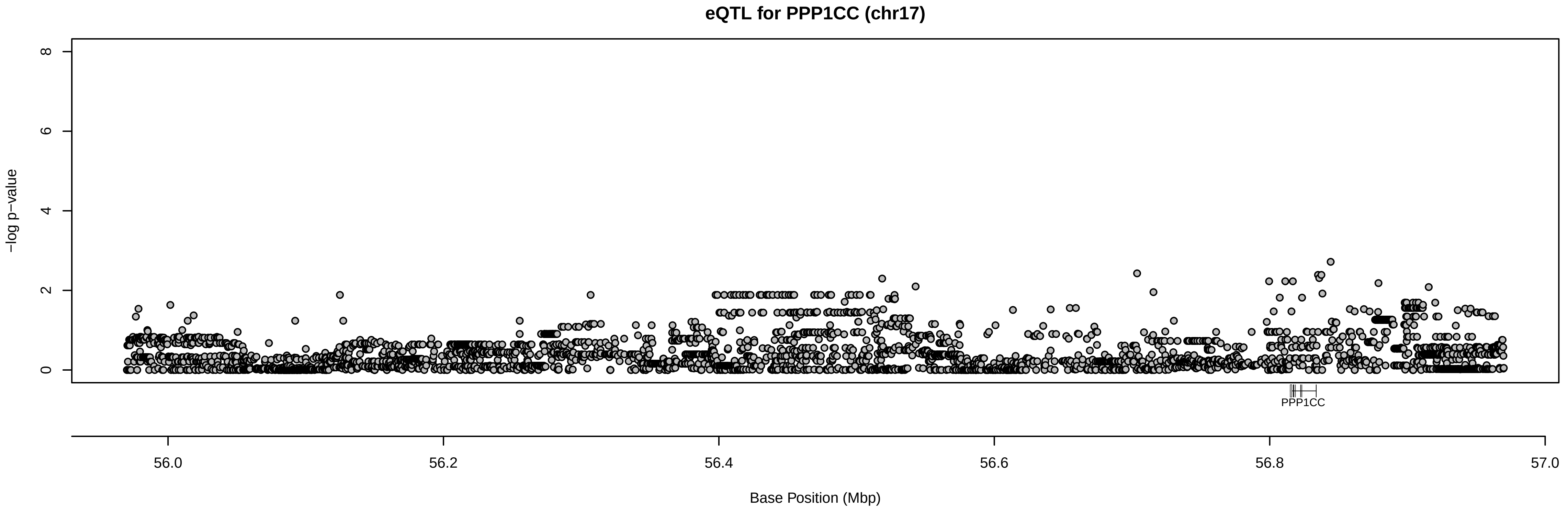
eQTL for PPP1CC (chr17)
−log p−value
0
2
4
6
8
PPP1CC
56.0
56.2
56.4
56.6
56.8
57.0
Base Position (Mbp)

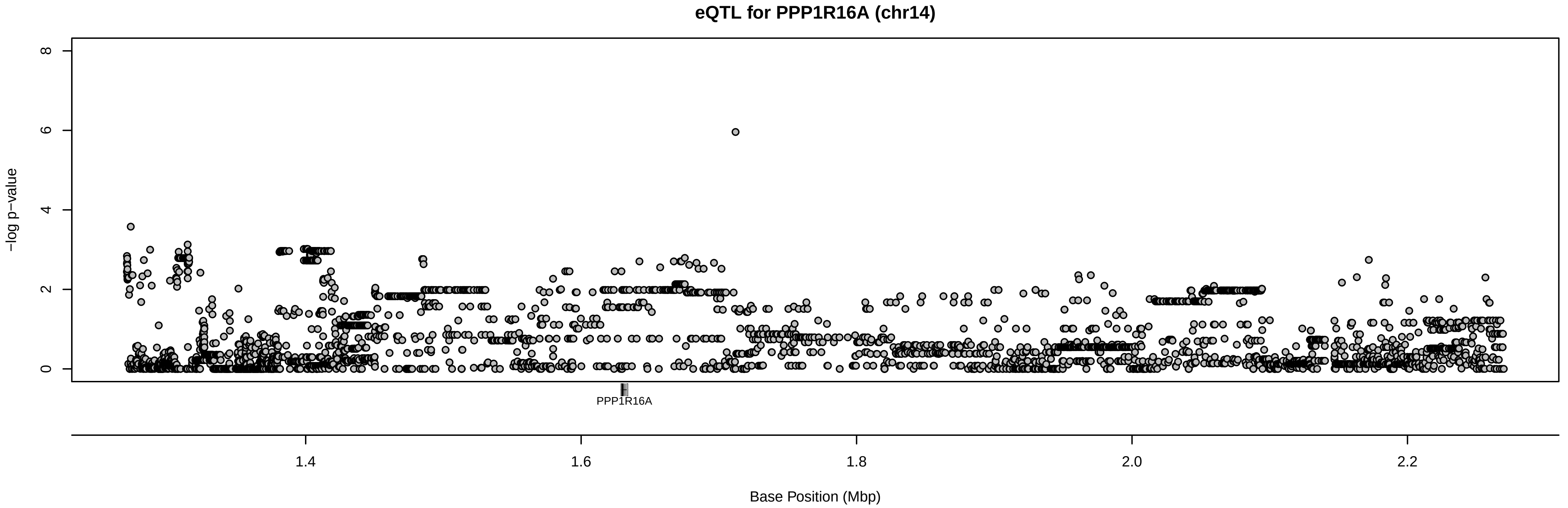
eQTL for PPP1R16A (chr14)
−log p−value
0
2
4
6
8
PPP1R16A
1.4
1.6
1.8
2.0
2.2
Base Position (Mbp)

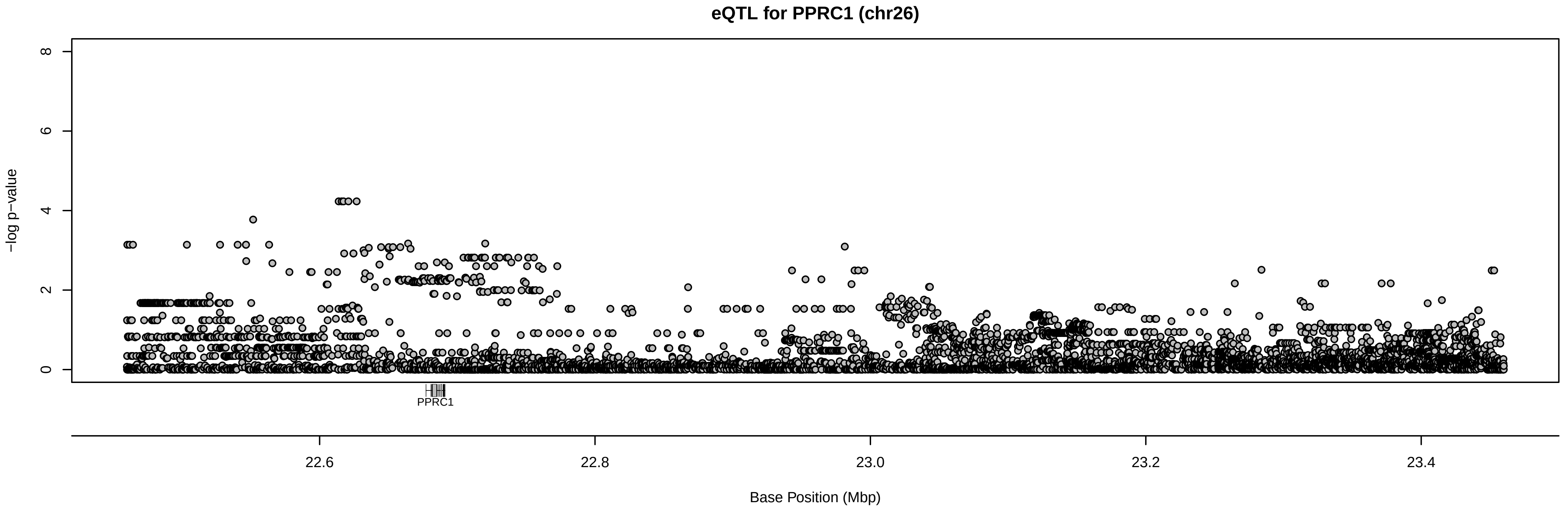

eQTL for PPRC1 (chr26)
−log p−value
0
2
4
6
8
PPRC1
22.6
22.8
23.0
23.2
23.4
Base Position (Mbp)

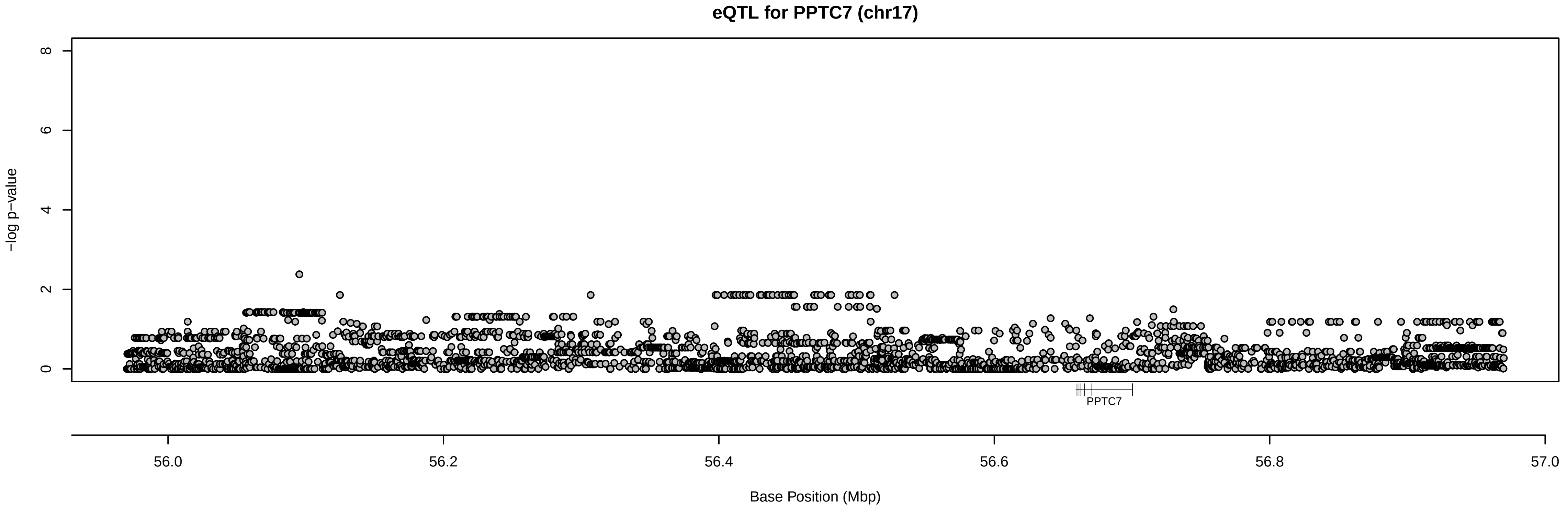

eQTL for PPTC7 (chr17)
−log p−value
0
2
4
6
8
PPTC7
56.0
56.2
56.4
56.6
56.8
57.0
Base Position (Mbp)

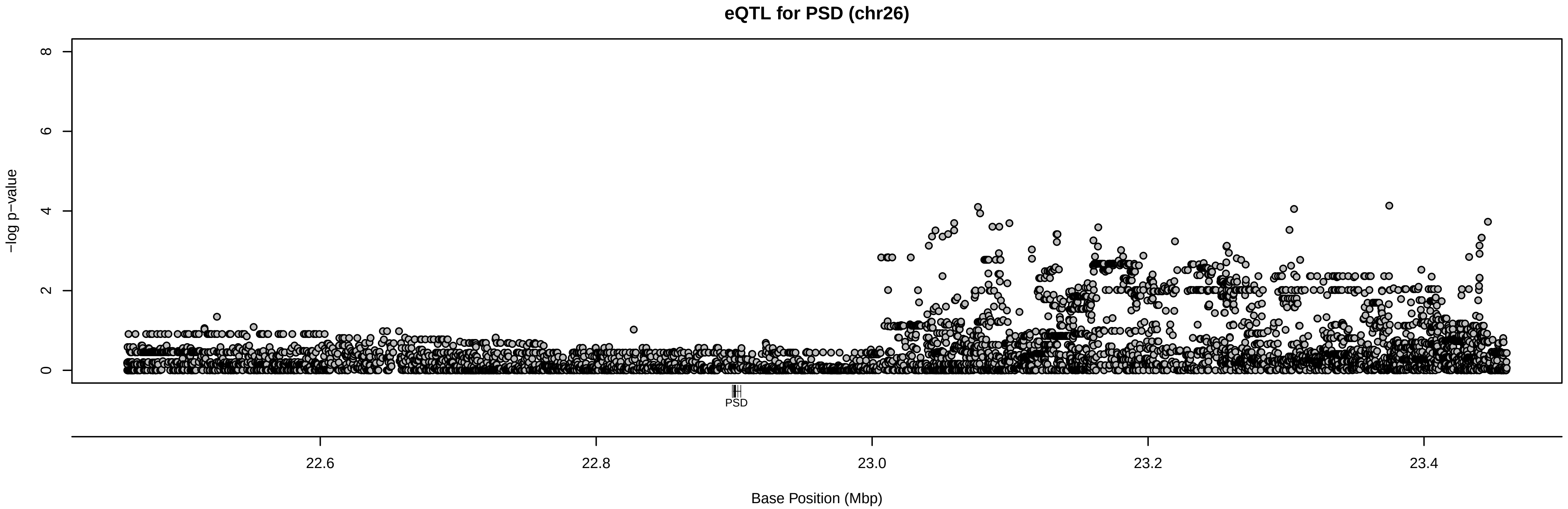

eQTL for PSD (chr26)
−log p−value
0
2
4
6
8
PSD
22.6
22.8
23.0
23.2
23.4
Base Position (Mbp)

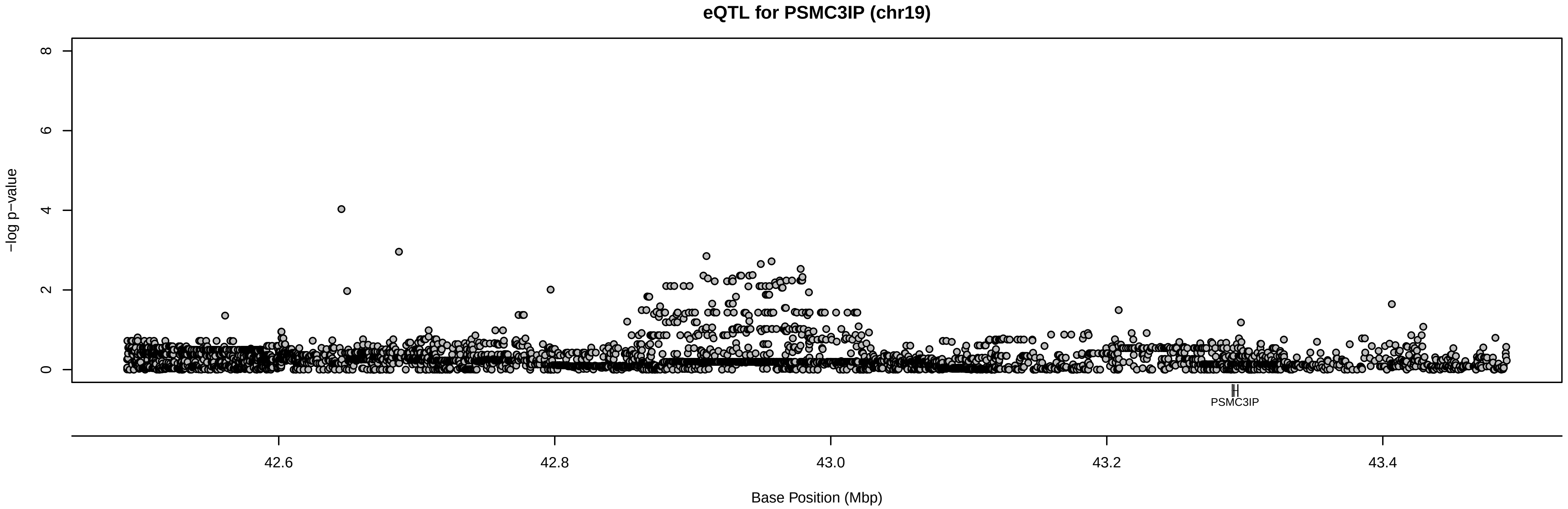

eQTL for PSMC3IP (chr19)
−log p−value
0
2
4
6
8
PSMC3IP
42.6
42.8
43.0
43.2
43.4
Base Position (Mbp)

eQTL for PTRF (chr19)

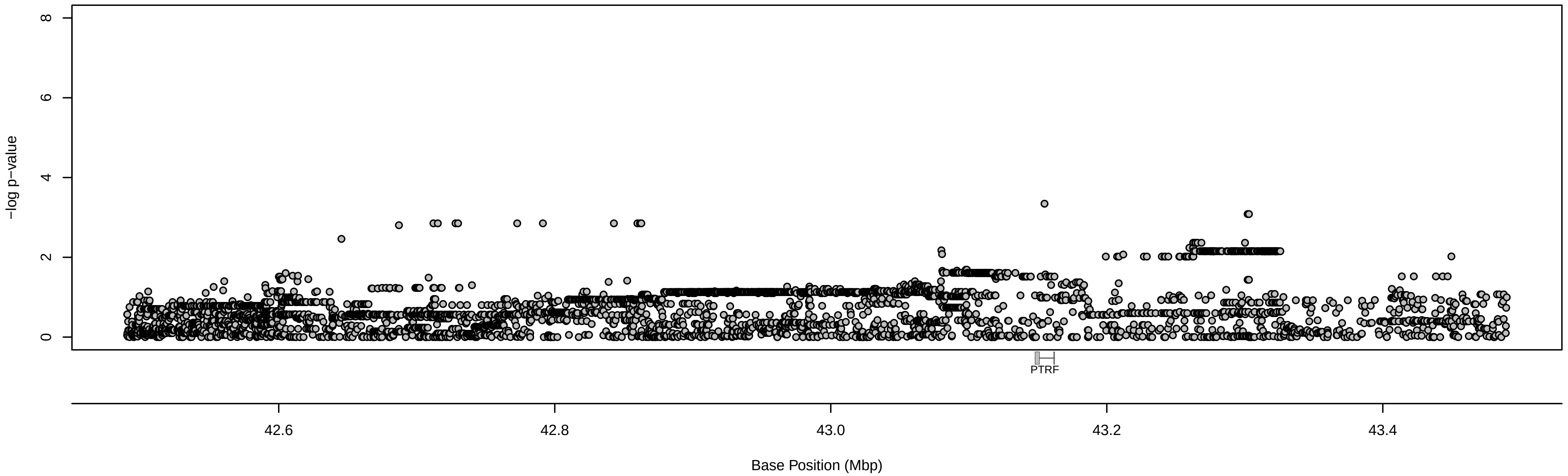

eQTL for PUF60 (chr14)

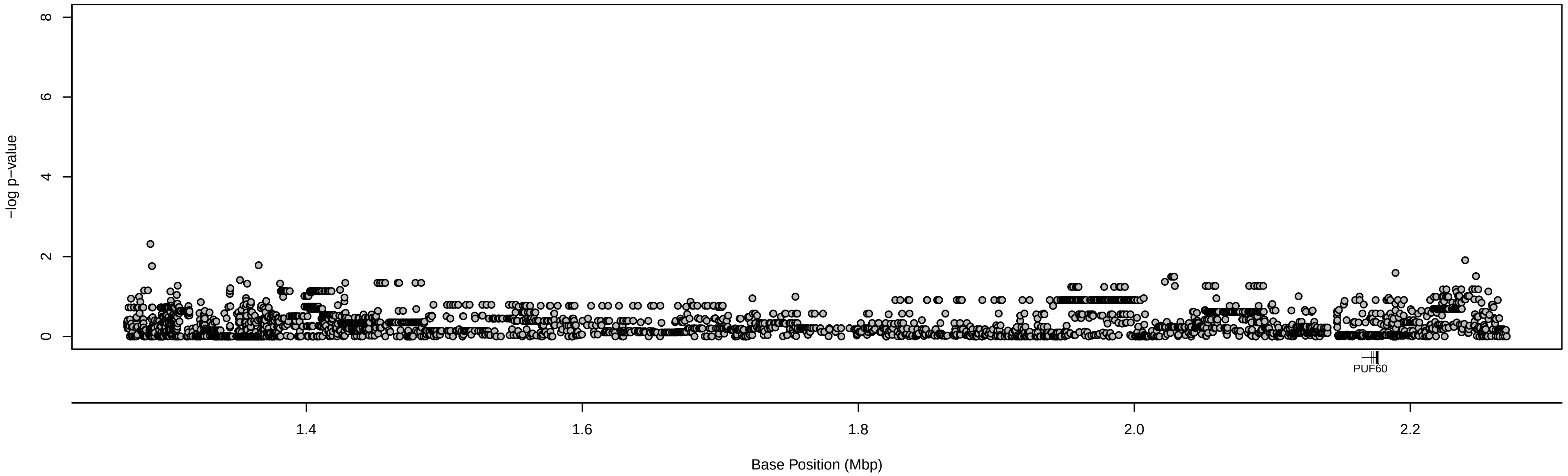

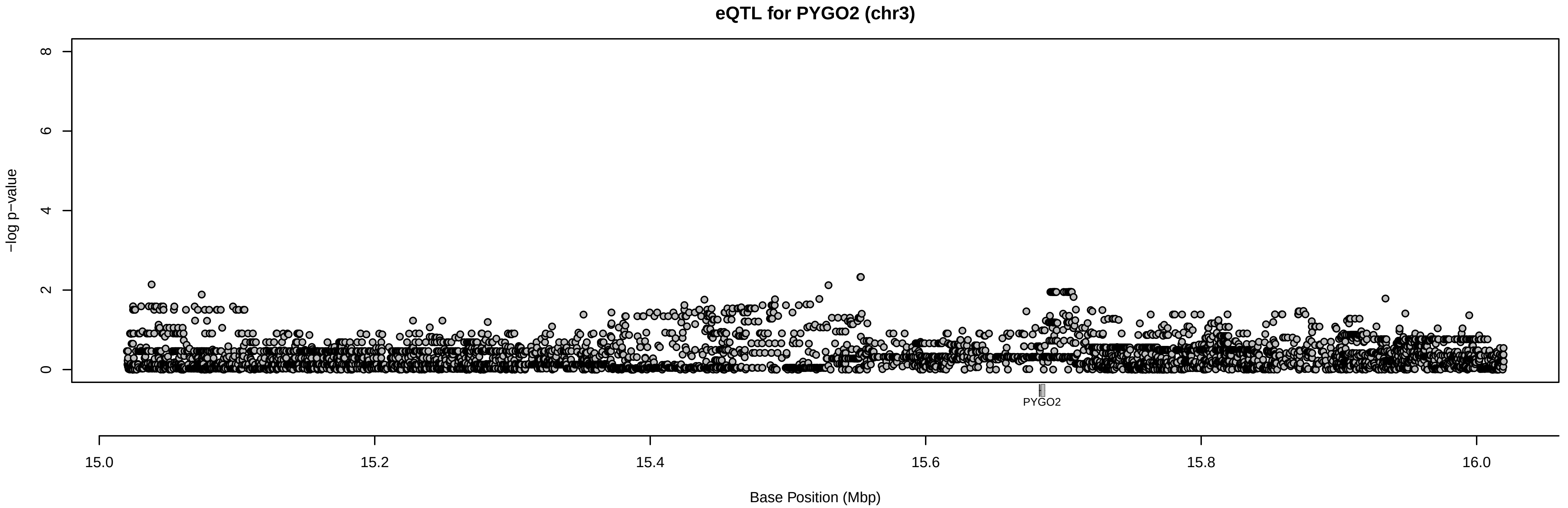
eQTL for PYGO2 (chr3)
−log p−value
0
2
4
6
8
PYGO2
15.0
15.2
15.4
15.6
15.8
16.0
Base Position (Mbp)

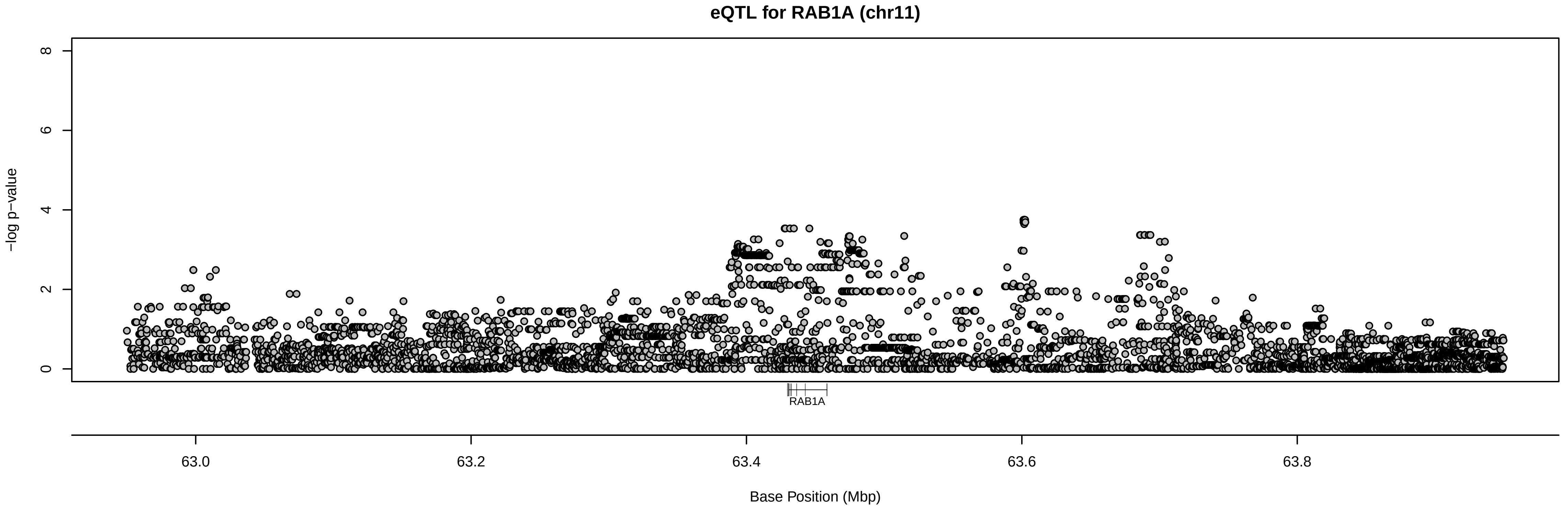
eQTL for RAB1A (chr11)
−log p−value
0
2
4
6
8
RAB1A
63.0
63.2
63.4
63.6
63.8
Base Position (Mbp)

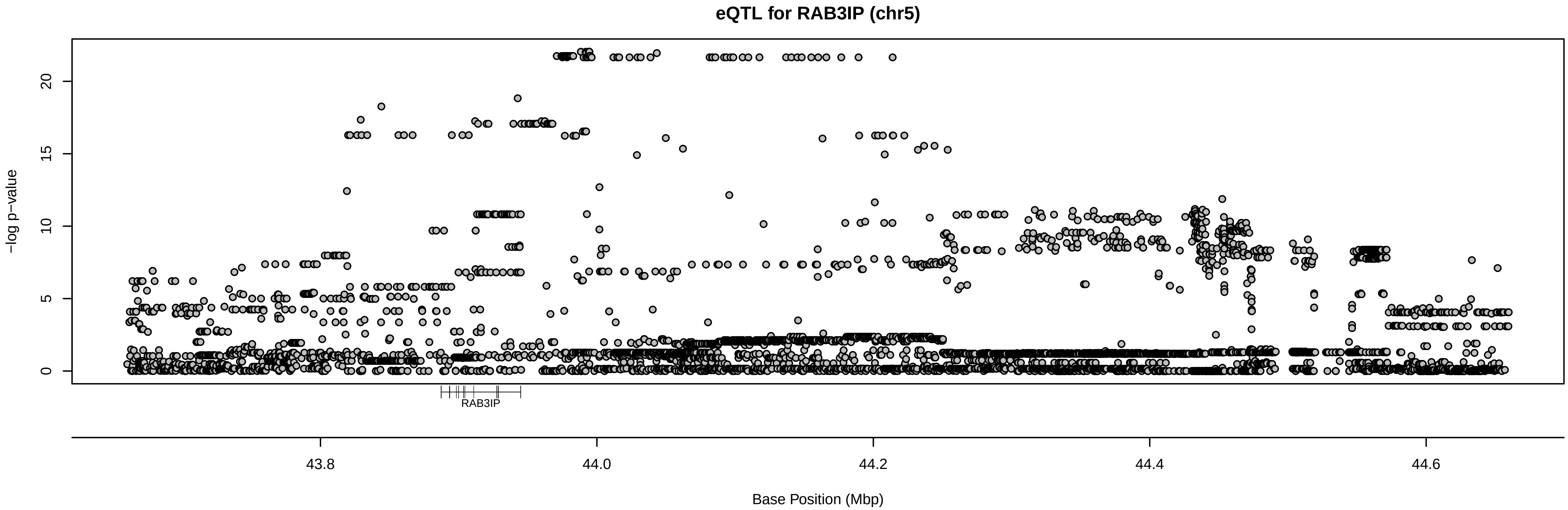

eQTL for RAB3IP (chr5)
−log p−value
0
5
10
15
20
RAB3IP
43.8
44.0
44.2
44.4
44.6
Base Position (Mbp)

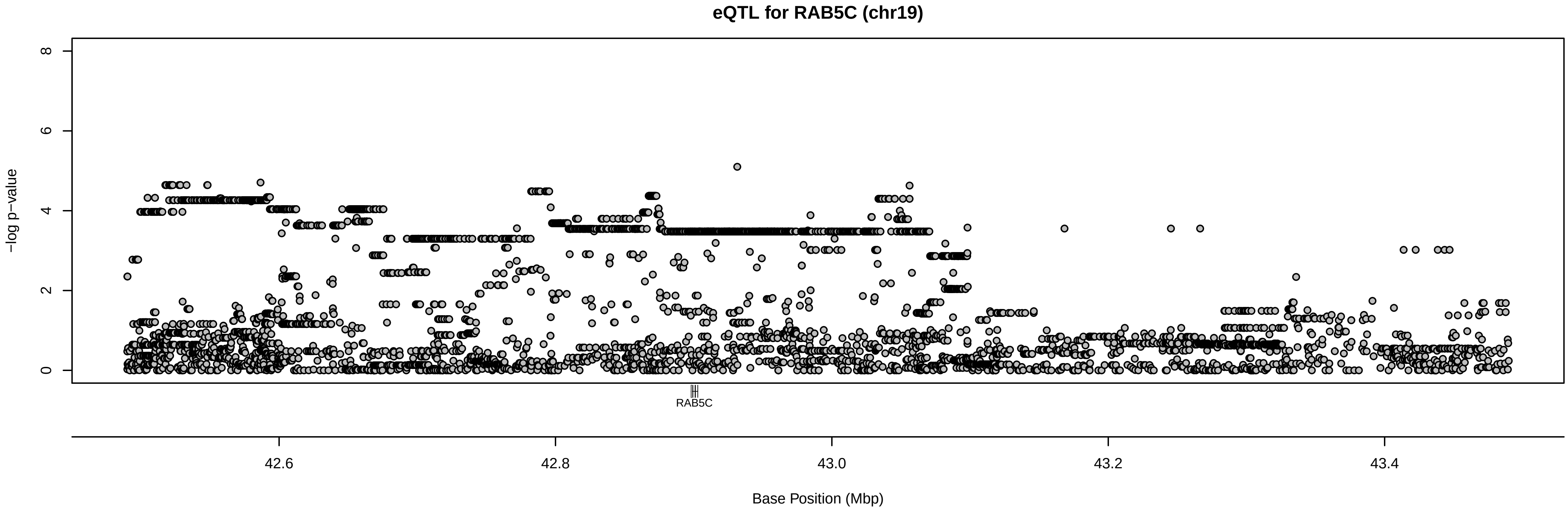

eQTL for RAB5C (chr19)
−log p−value
0
2
4
6
8
RAB5C
42.6
42.8
43.0
43.2
43.4
Base Position (Mbp)

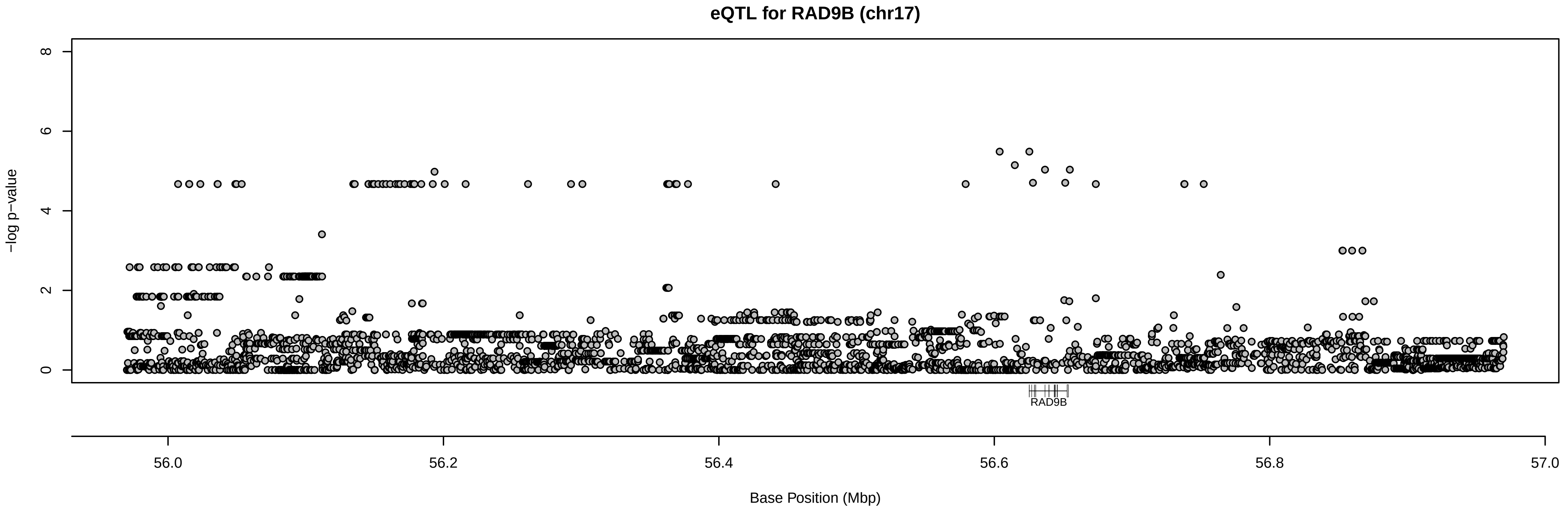
eQTL for RAD9B (chr17)
−log p−value
0
2
4
6
8
RAD9B
56.0
56.2
56.4
56.6
56.8
57.0
Base Position (Mbp)

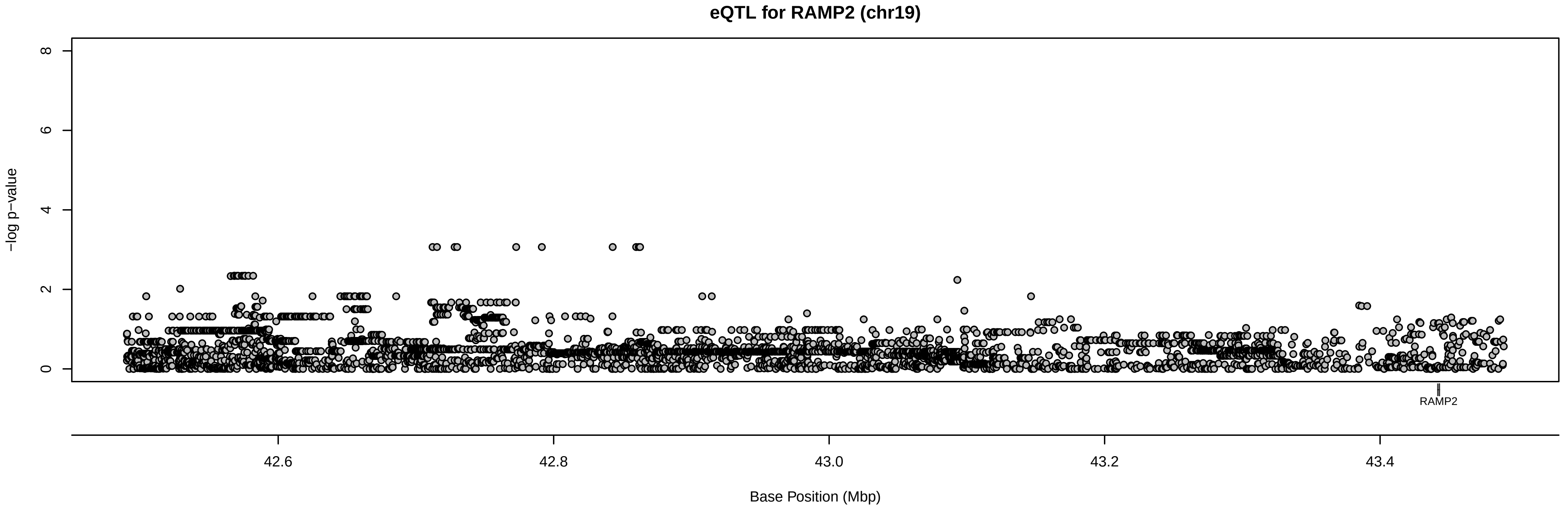
eQTL for RAMP2 (chr19)
−log p−value
0
2
4
6
8
RAMP2
42.6
42.8
43.0
43.2
43.4
Base Position (Mbp)

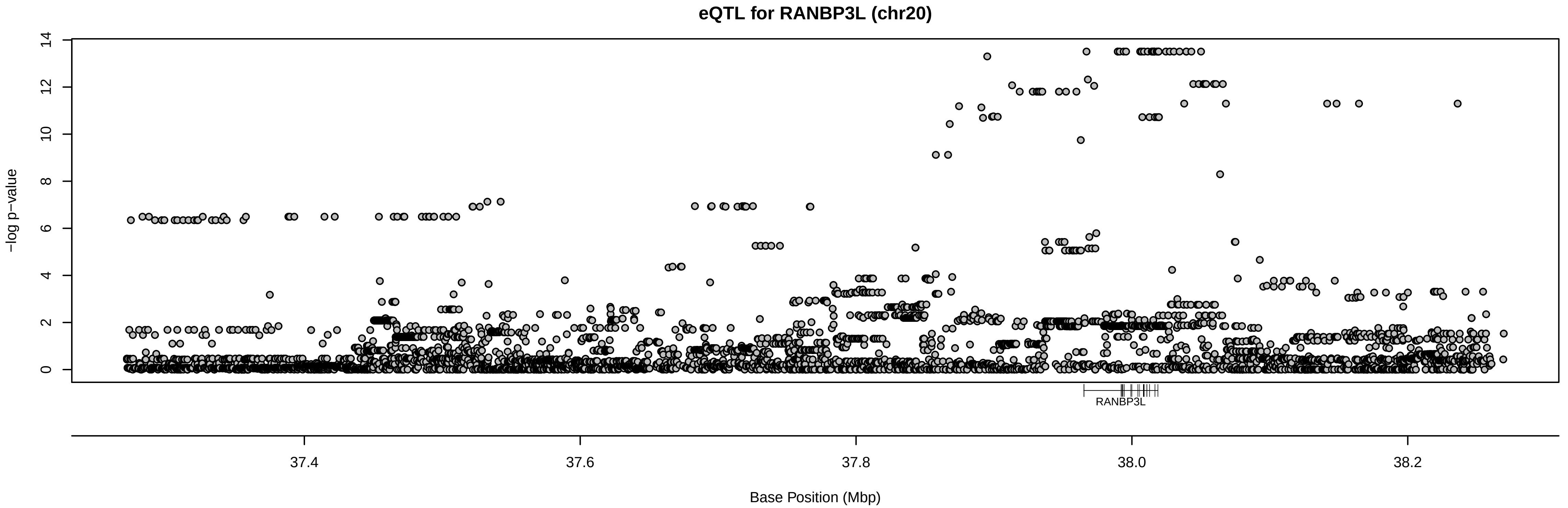
eQTL for RANBP3L (chr20)
−log p−value
0
2
4
6
8
10
12
14
RANBP3L
37.4
37.6
37.8
38.0
38.2
Base Position (Mbp)

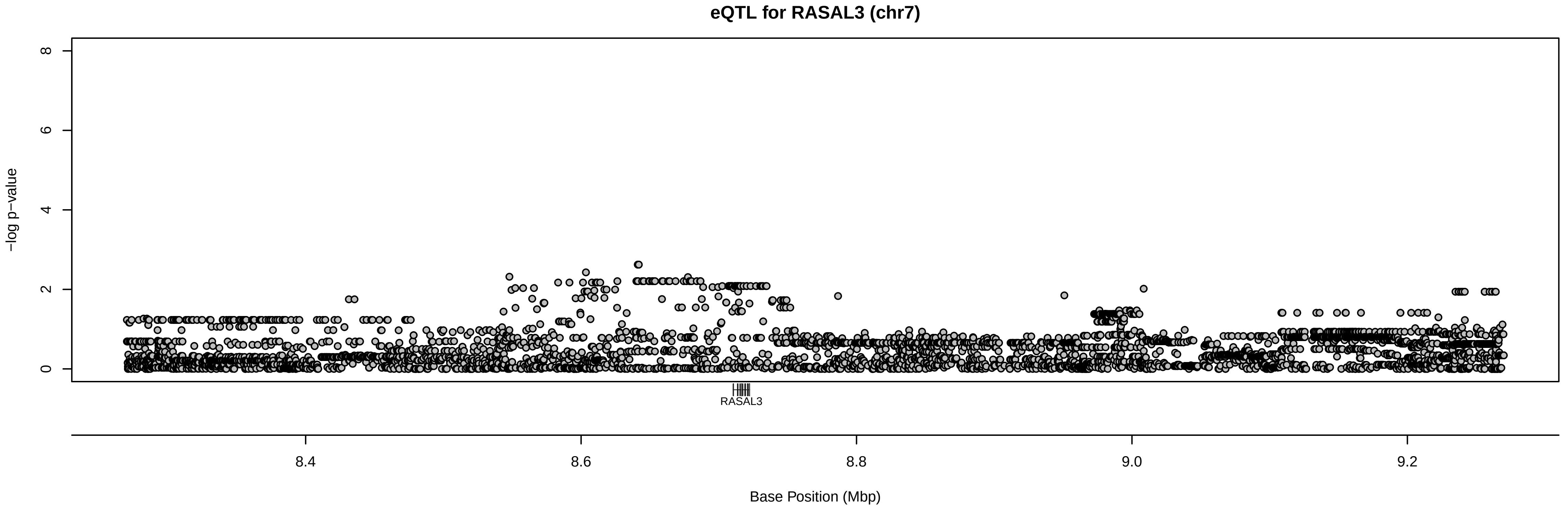
eQTL for RASAL3 (chr7)
−log p−value
0
2
4
6
8
RASAL3
8.4
8.6
8.8
9.0
9.2
Base Position (Mbp)

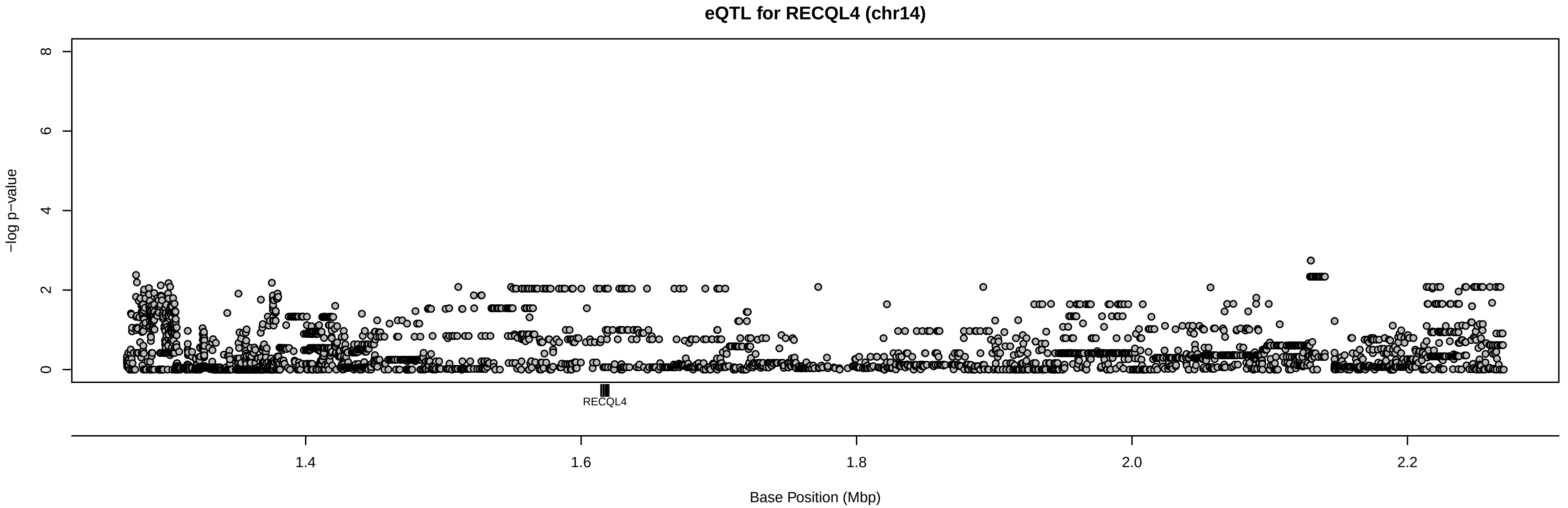

eQTL for RECQL4 (chr14)
−log p−value
0
2
4
6
8
RECQL4
1.4
1.6
1.8
2.0
2.2
Base Position (Mbp)

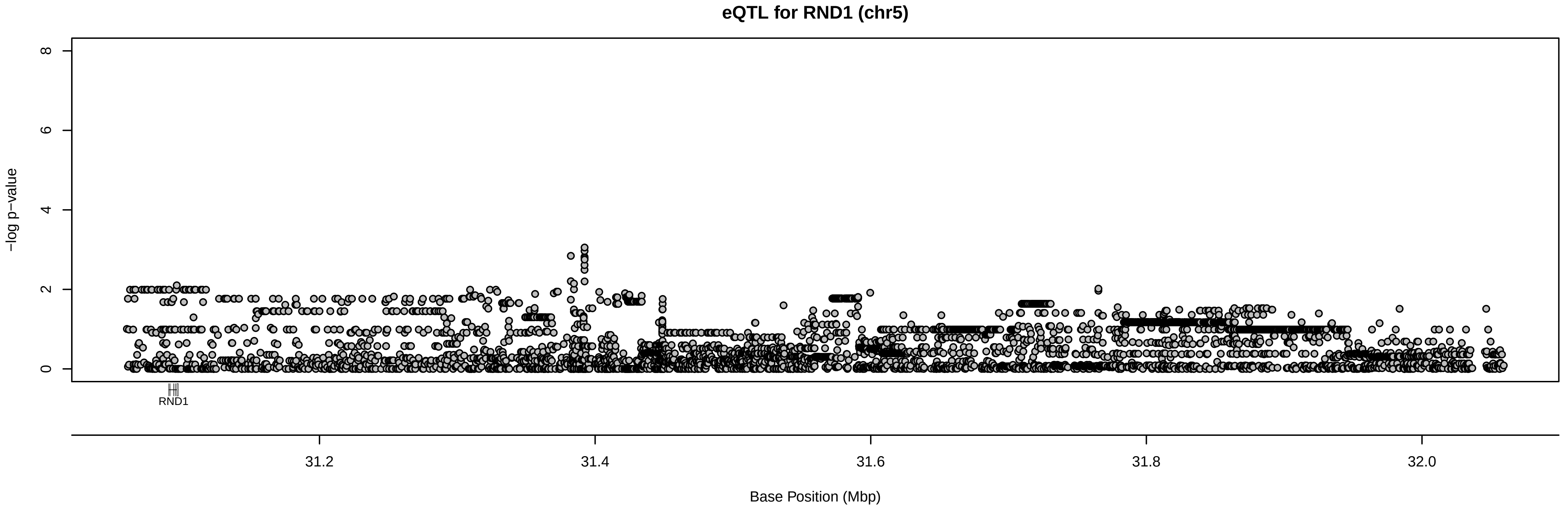

eQTL for RND1 (chr5)
−log p−value
0
2
4
6
8
RND1
31.2
31.4
31.6
31.8
32.0
Base Position (Mbp)

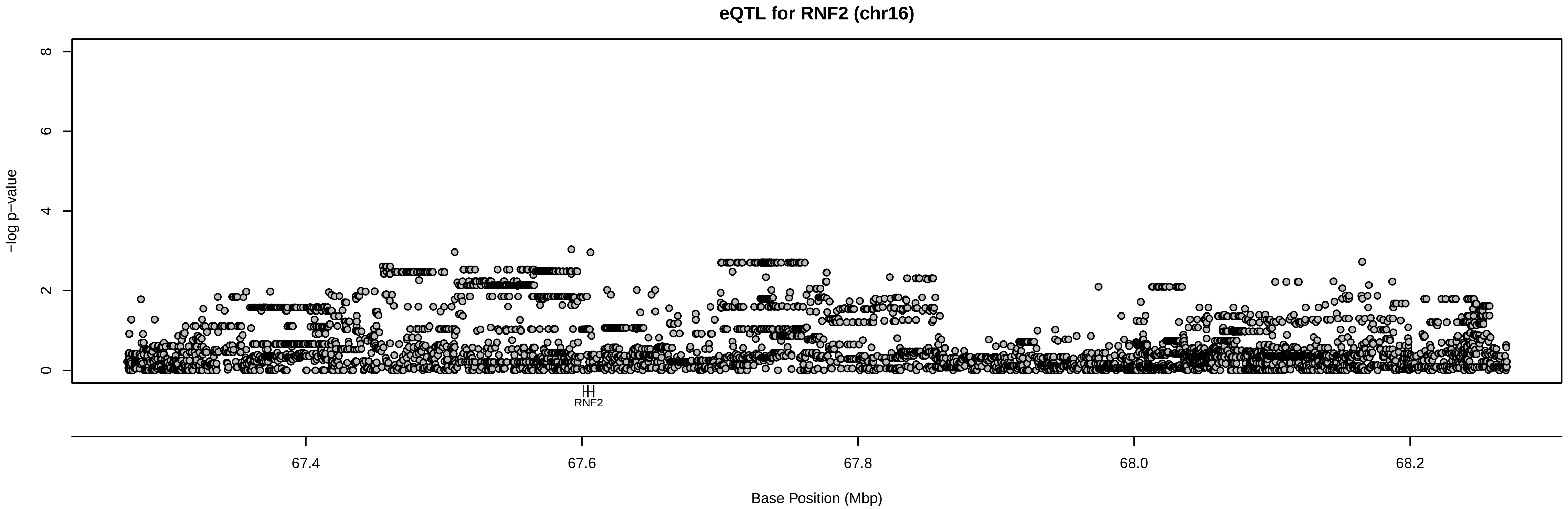
eQTL for RNF2 (chr16)
−log p−value
0
2
4
6
8
RNF2
67.4
67.6
67.8
68.0
68.2
Base Position (Mbp)

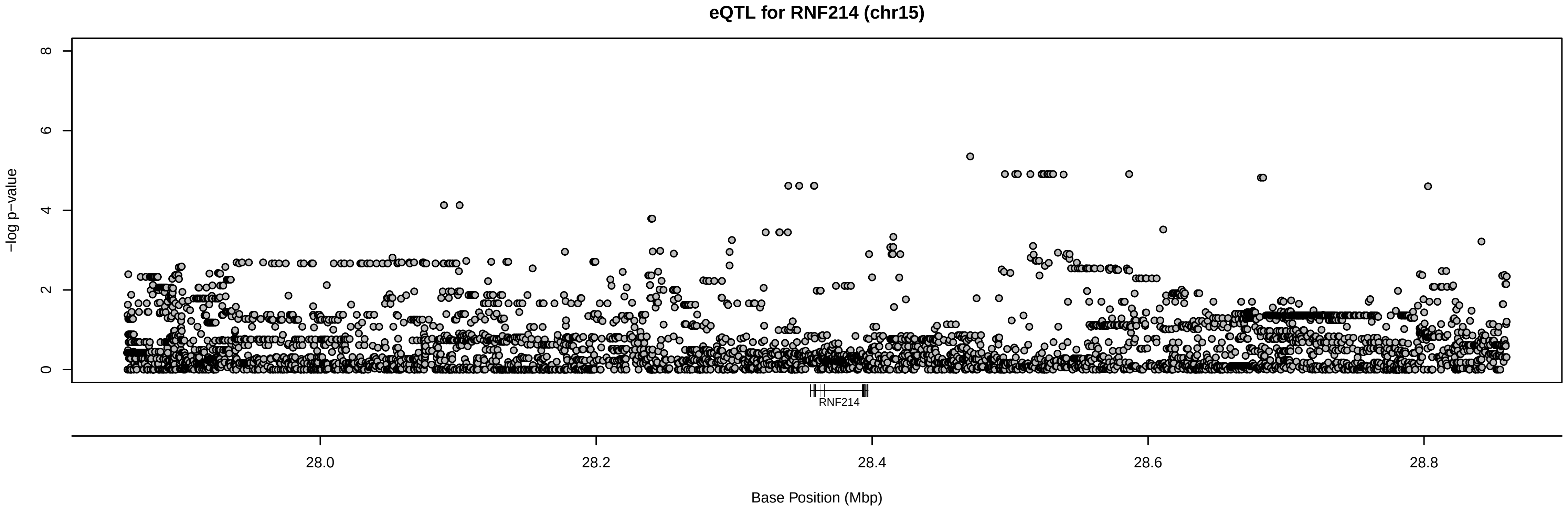
eQTL for RNF214 (chr15)
−log p−value
0
2
4
6
8
RNF214
28.0
28.2
28.4
28.6
28.8
Base Position (Mbp)

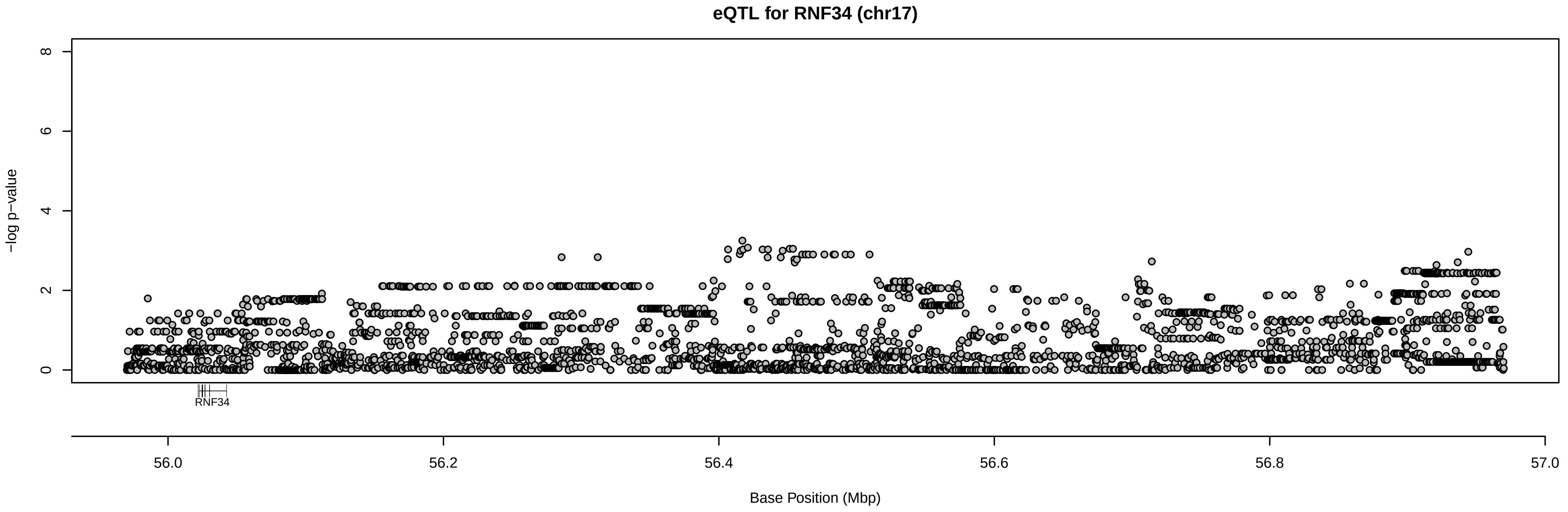
eQTL for RNF34 (chr17)
−log p−value
0
2
4
6
8
RNF34
56.0
56.2
56.4
56.6
56.8
57.0
Base Position (Mbp)

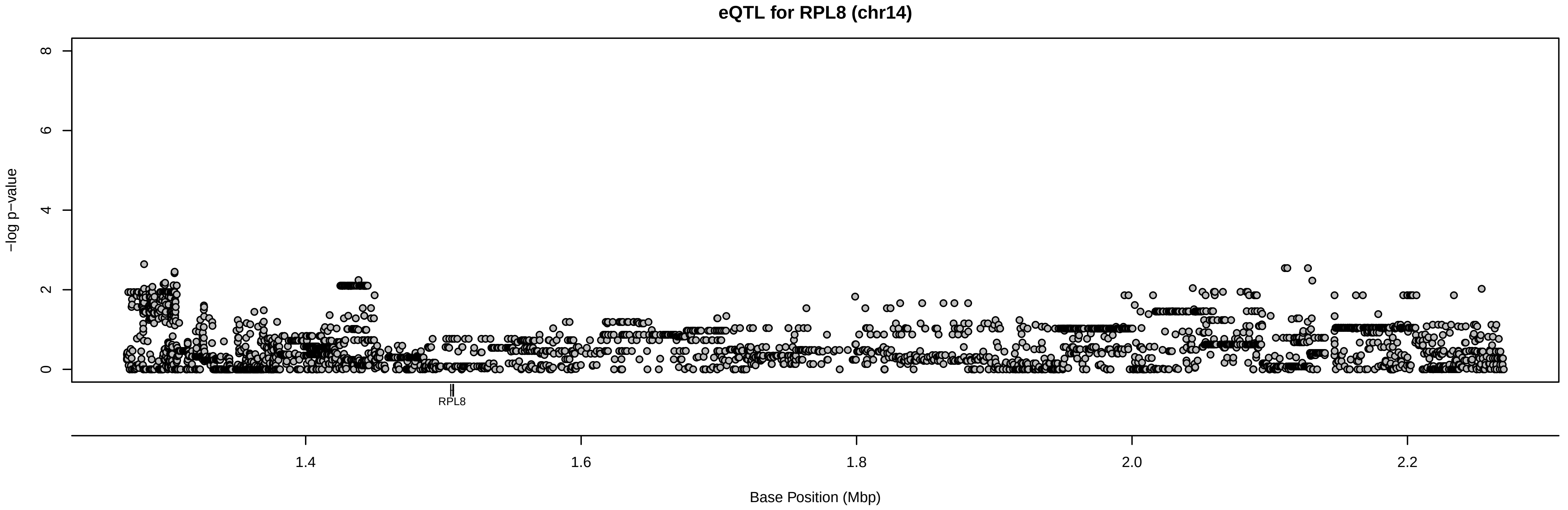
eQTL for RPL8 (chr14)
−log p−value
0
2
4
6
8
RPL8
1.4
1.6
1.8
2.0
2.2
Base Position (Mbp)

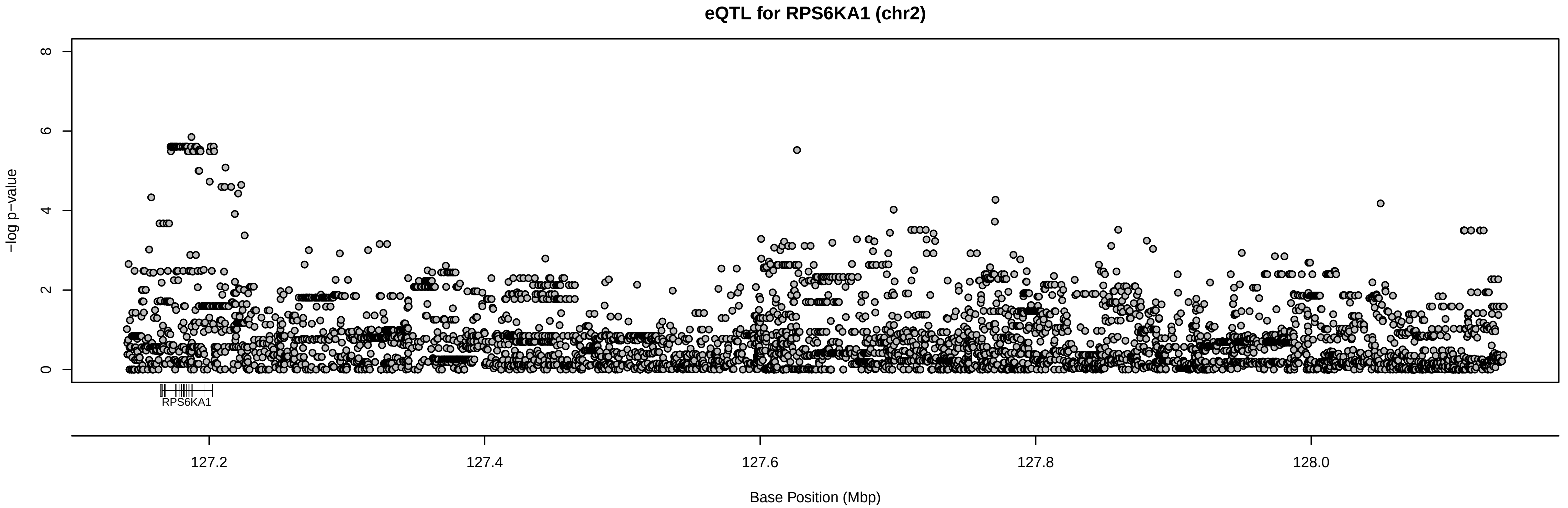

eQTL for RPS6KA1 (chr2)
−log p−value
0
2
4
6
8
RPS6KA1
127.2
127.4
127.6
127.8
128.0
Base Position (Mbp)

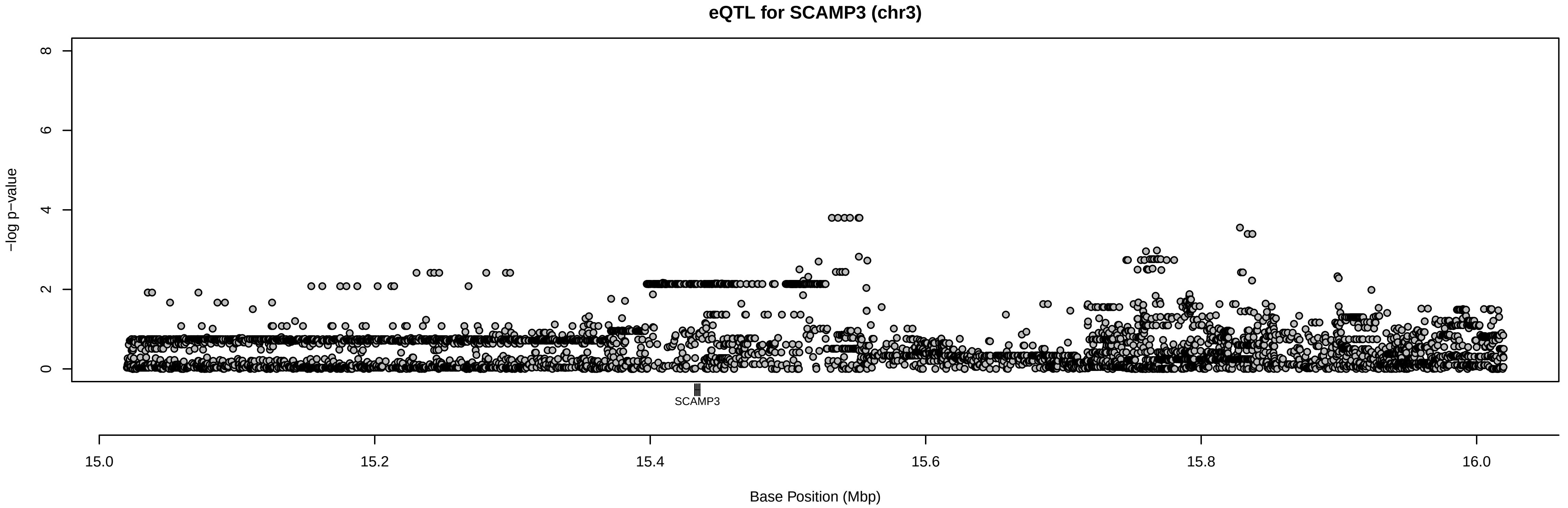

eQTL for SCAMP3 (chr3)
−log p−value
0
2
4
6
8
SCAMP3
15.0
15.2
15.4
15.6
15.8
16.0
Base Position (Mbp)

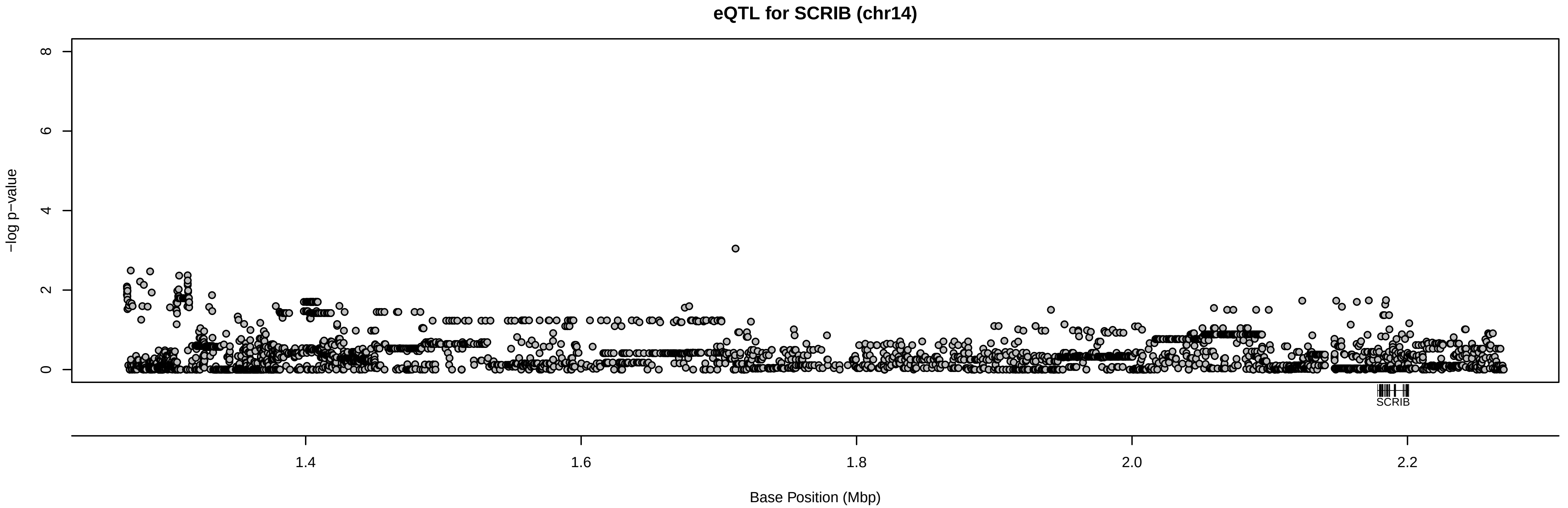
eQTL for SCRIB (chr14)
−log p−value
0
2
4
6
8
SCRIB
1.4
1.6
1.8
2.0
2.2
Base Position (Mbp)

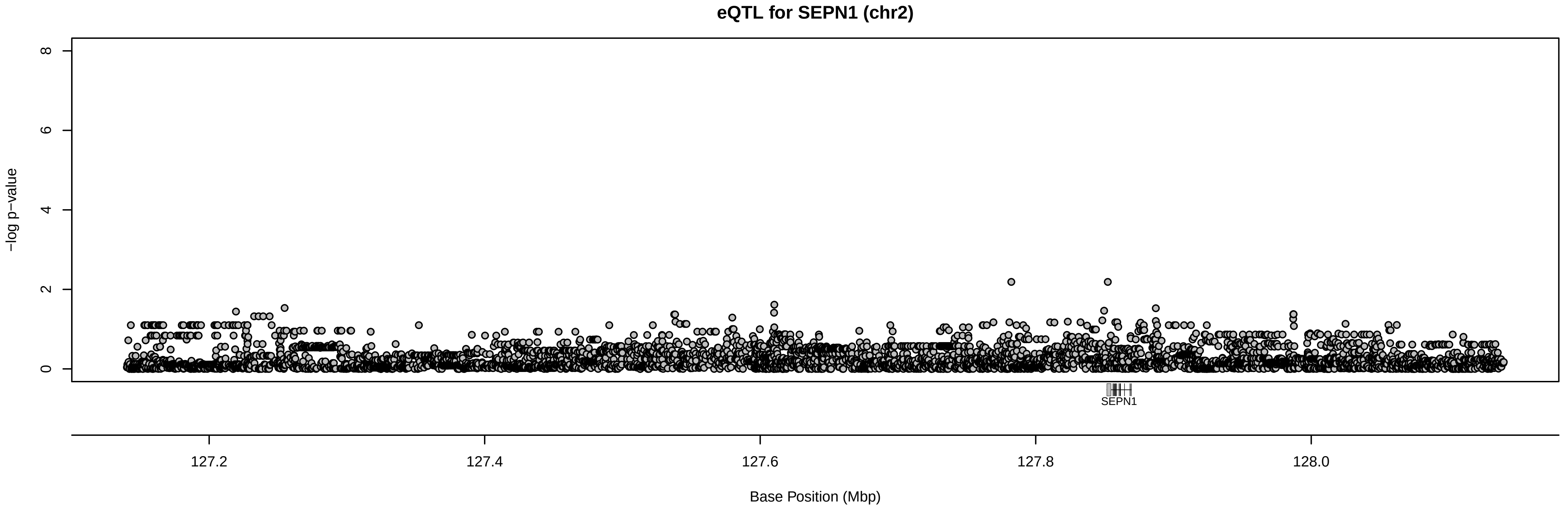
eQTL for SEPN1 (chr2)
−log p−value
0
2
4
6
8
SEPN1
127.2
127.4
127.6
127.8
128.0
Base Position (Mbp)

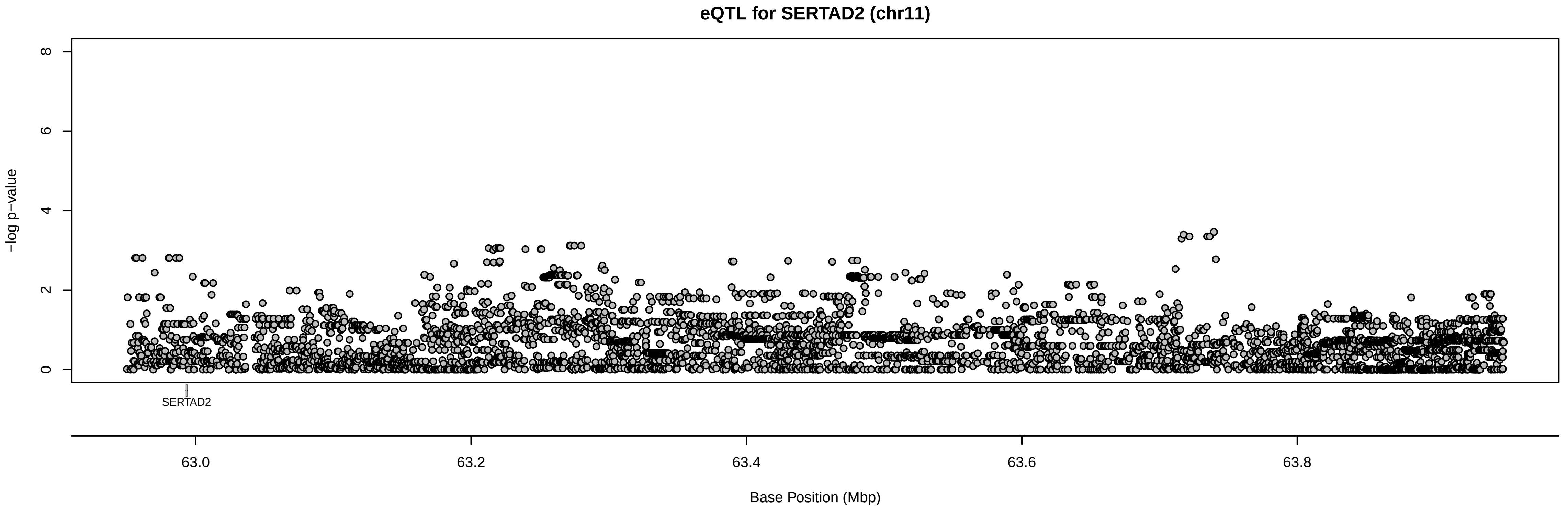
eQTL for SERTAD2 (chr11)
−log p−value
0
2
4
6
8
SERTAD2
63.0
63.2
63.4
63.6
63.8
Base Position (Mbp)

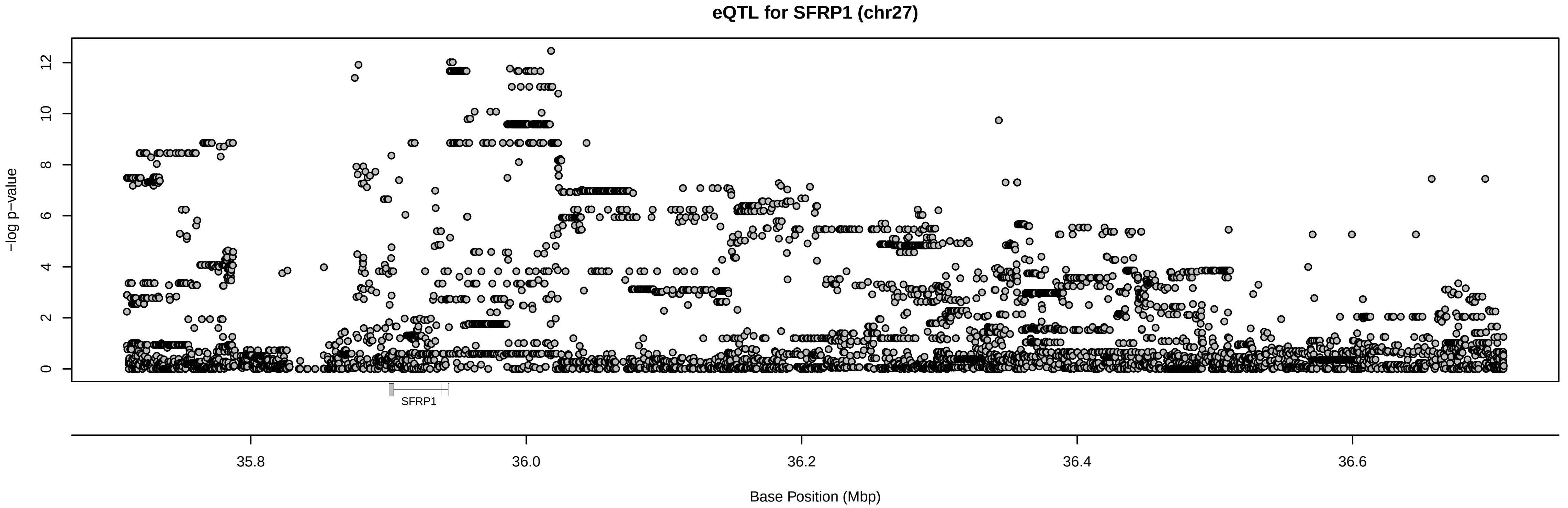
eQTL for SFRP1 (chr27)
−log p−value
0
2
4
6
8
10
12
SFRP1
35.8
36.0
36.2
36.4
36.6
Base Position (Mbp)

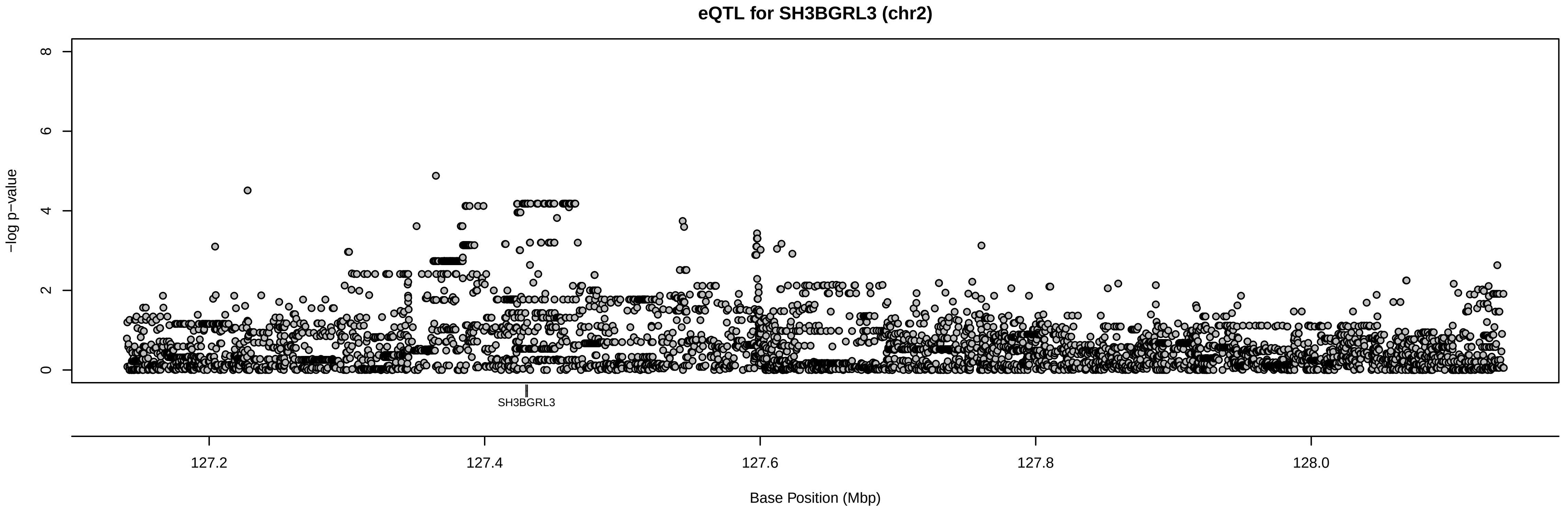

eQTL for SH3BGRL3 (chr2)
−log p−value
0
2
4
6
8
SH3BGRL3
127.2
127.4
127.6
127.8
128.0
Base Position (Mbp)

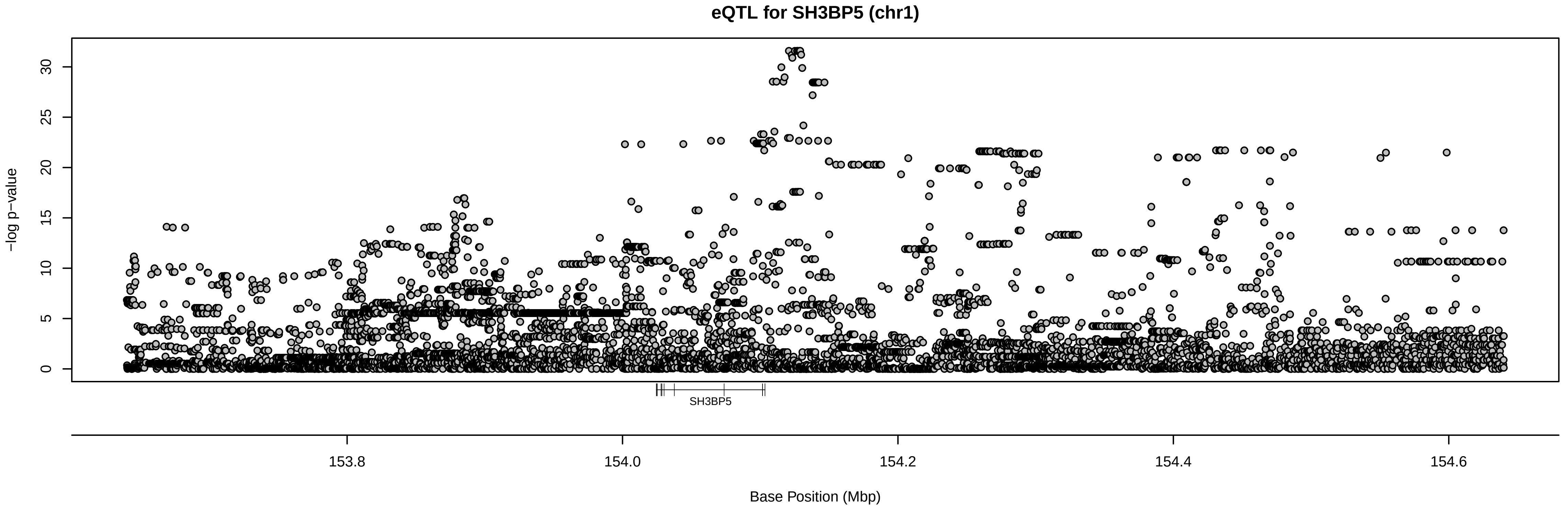

eQTL for SH3BP5 (chr1)
−log p−value
0
5
10
15
20
25
30
SH3BP5
153.8
154.0
154.2
154.4
154.6
Base Position (Mbp)

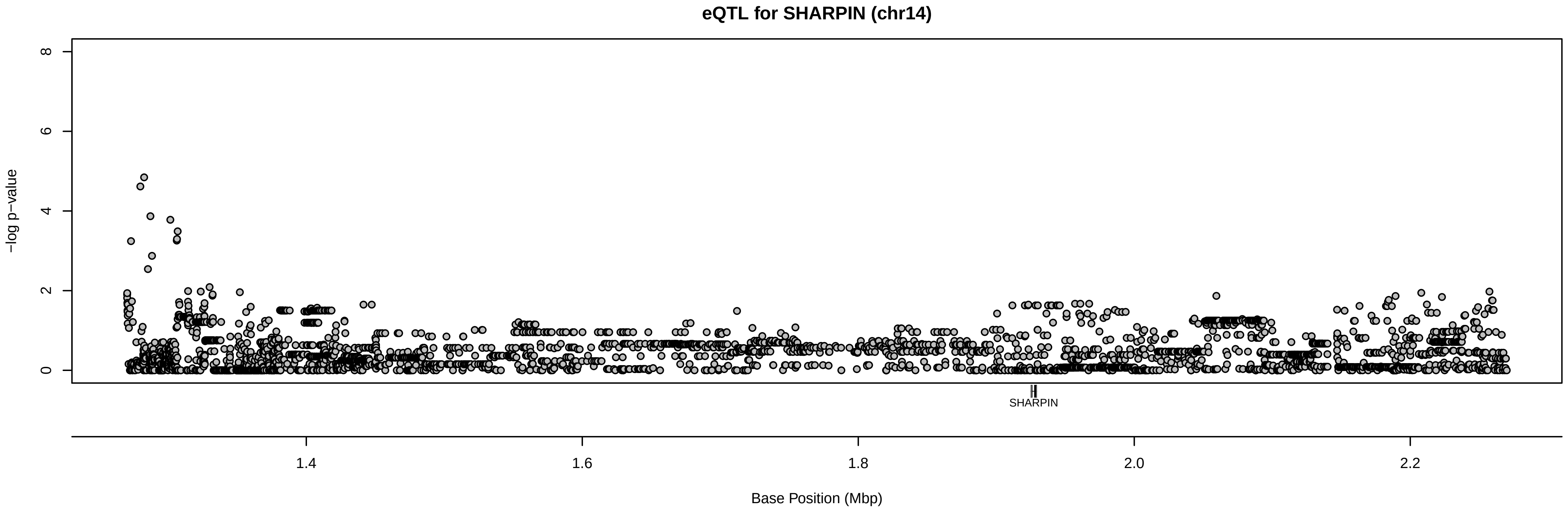
eQTL for SHARPIN (chr14)
−log p−value
0
2
4
6
8
SHARPIN
1.4
1.6
1.8
2.0
2.2
Base Position (Mbp)

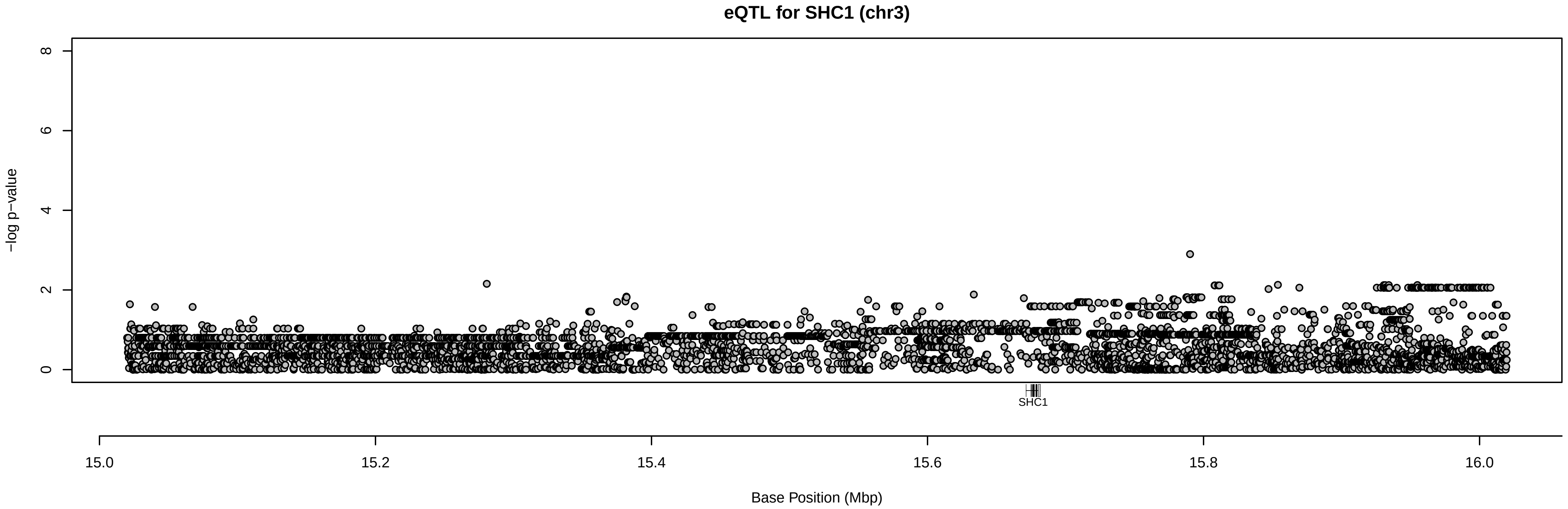
eQTL for SHC1 (chr3)
−log p−value
0
2
4
6
8
SHC1
15.0
15.2
15.4
15.6
15.8
16.0
Base Position (Mbp)

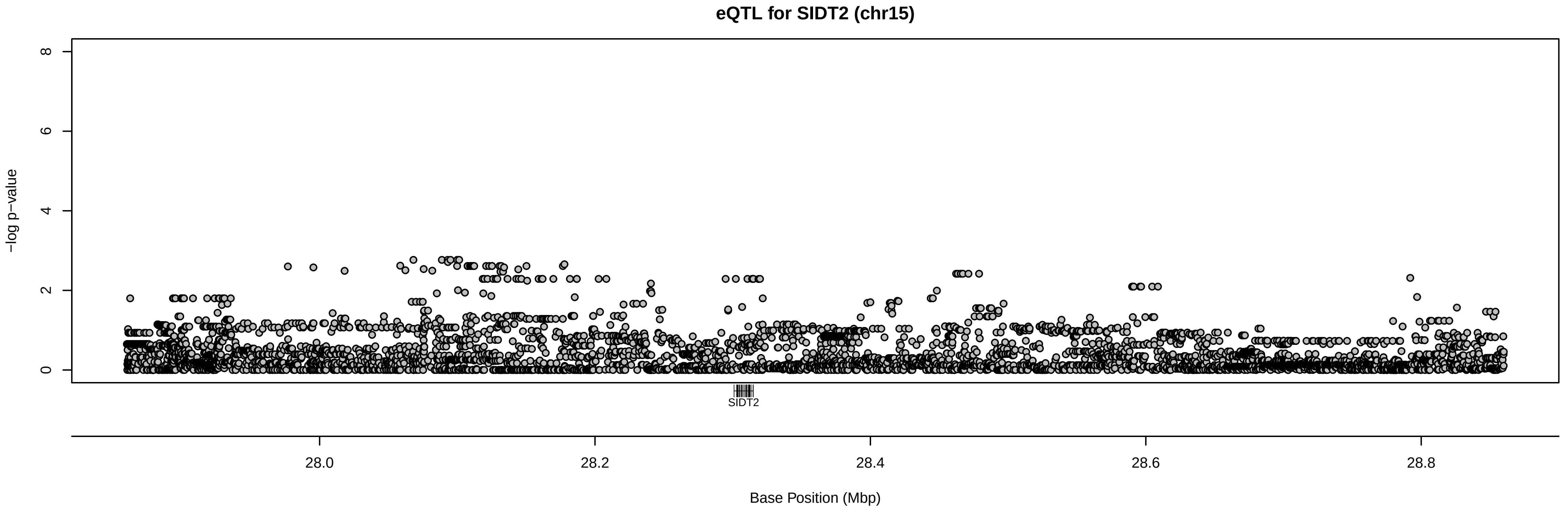
eQTL for SIDT2 (chr15)
−log p−value
0
2
4
6
8
SIDT2
28.0
28.2
28.4
28.6
28.8
Base Position (Mbp)

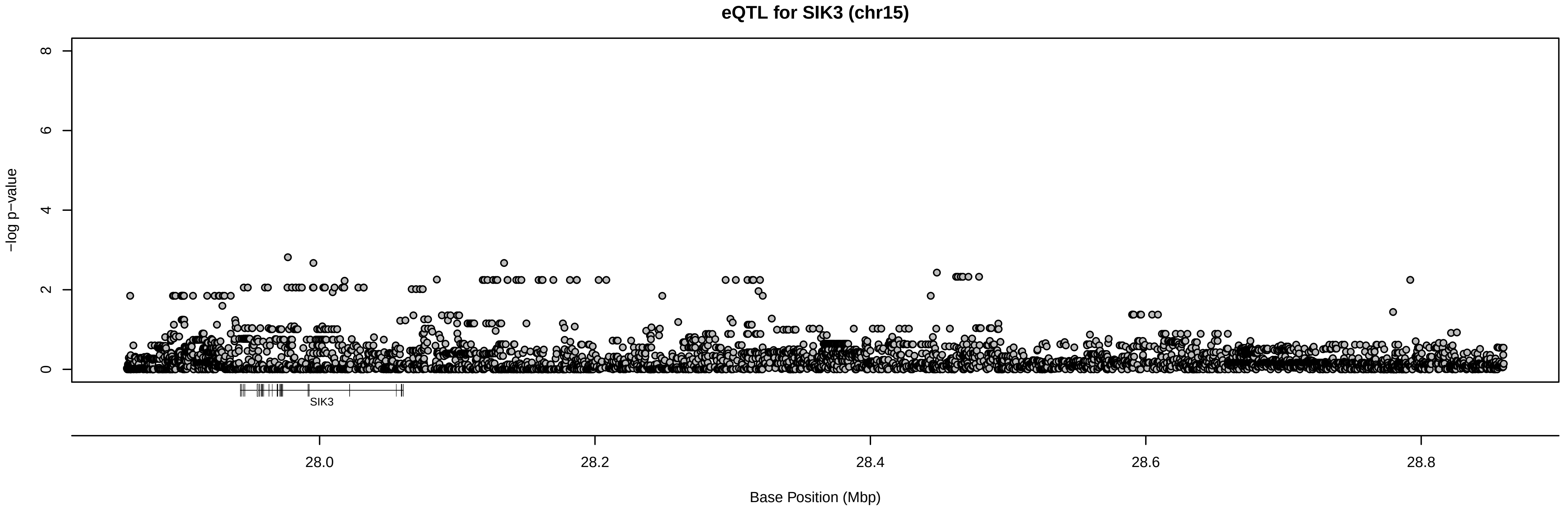
eQTL for SIK3 (chr15)
−log p−value
0
2
4
6
8
SIK3
28.0
28.2
28.4
28.6
28.8
Base Position (Mbp)

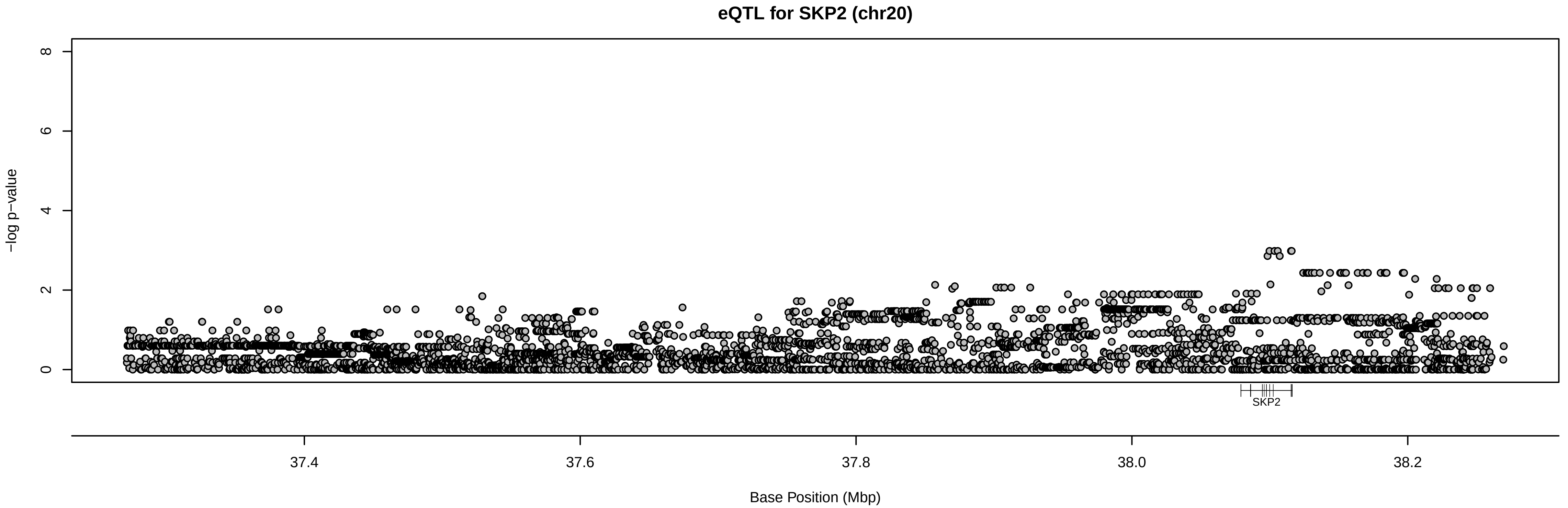

eQTL for SKP2 (chr20)
−log p−value
0
2
4
6
8
SKP2
37.4
37.6
37.8
38.0
38.2
Base Position (Mbp)

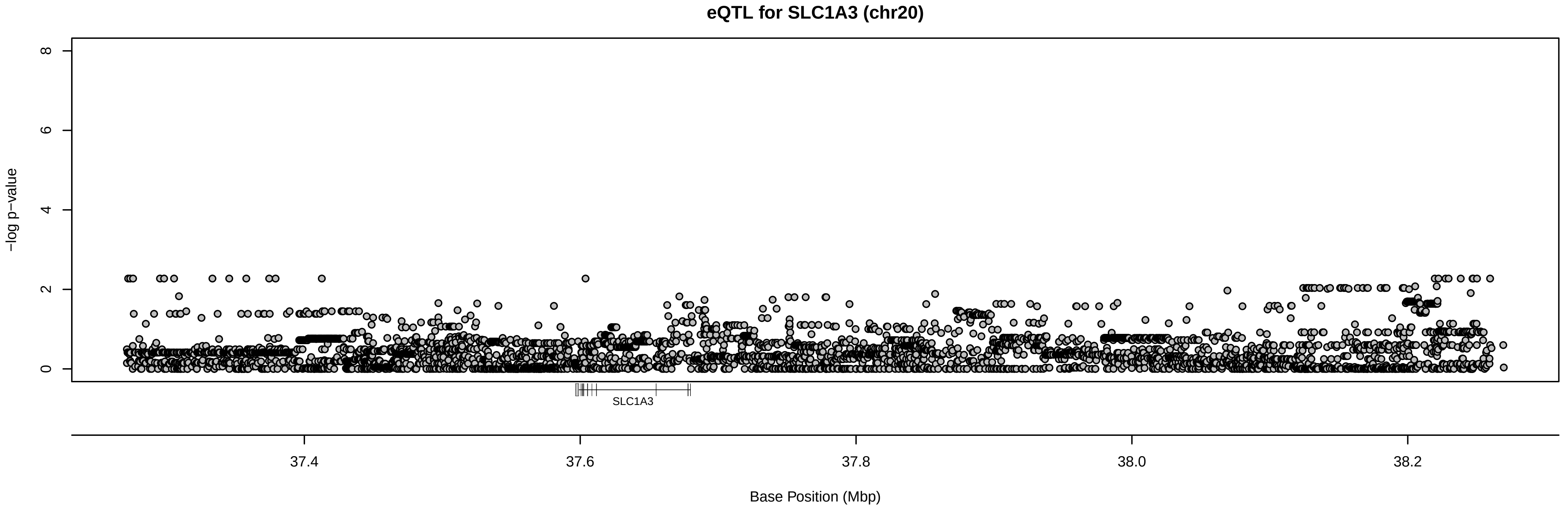

eQTL for SLC1A3 (chr20)
−log p−value
0
2
4
6
8
SLC1A3
37.4
37.6
37.8
38.0
38.2
Base Position (Mbp)

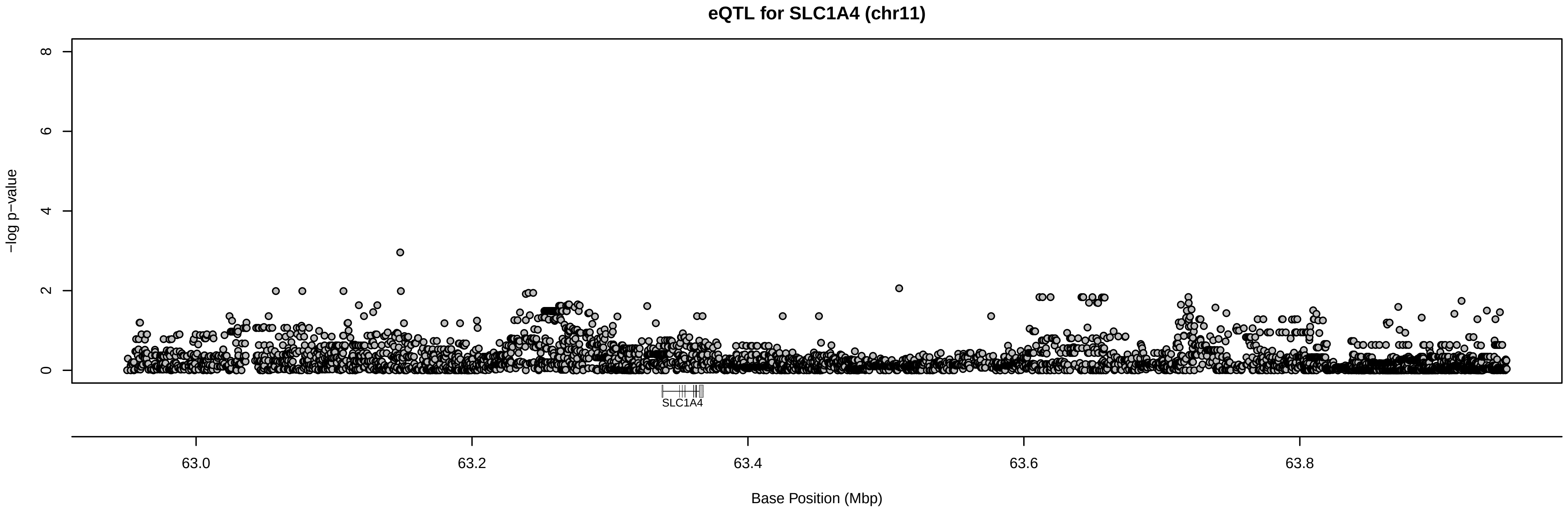
eQTL for SLC1A4 (chr11)
−log p−value
0
2
4
6
8
SLC1A4
63.0
63.2
63.4
63.6
63.8
Base Position (Mbp)

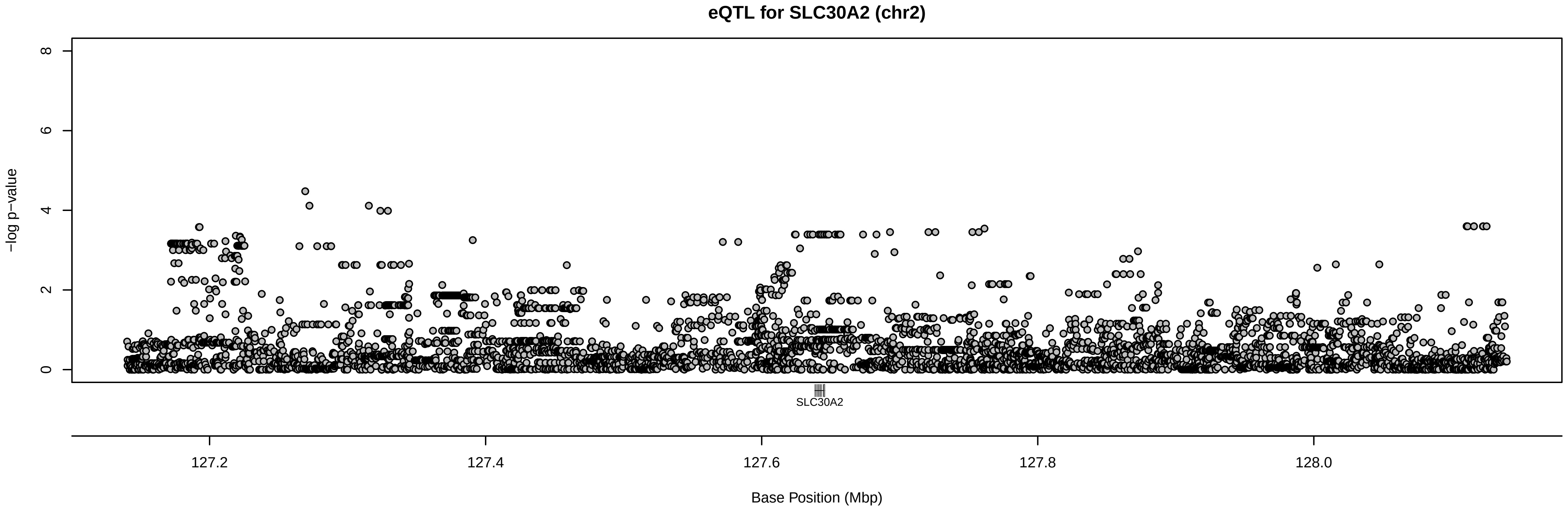
eQTL for SLC30A2 (chr2)
−log p−value
0
2
4
6
8
SLC30A2
127.2
127.4
127.6
127.8
128.0
Base Position (Mbp)

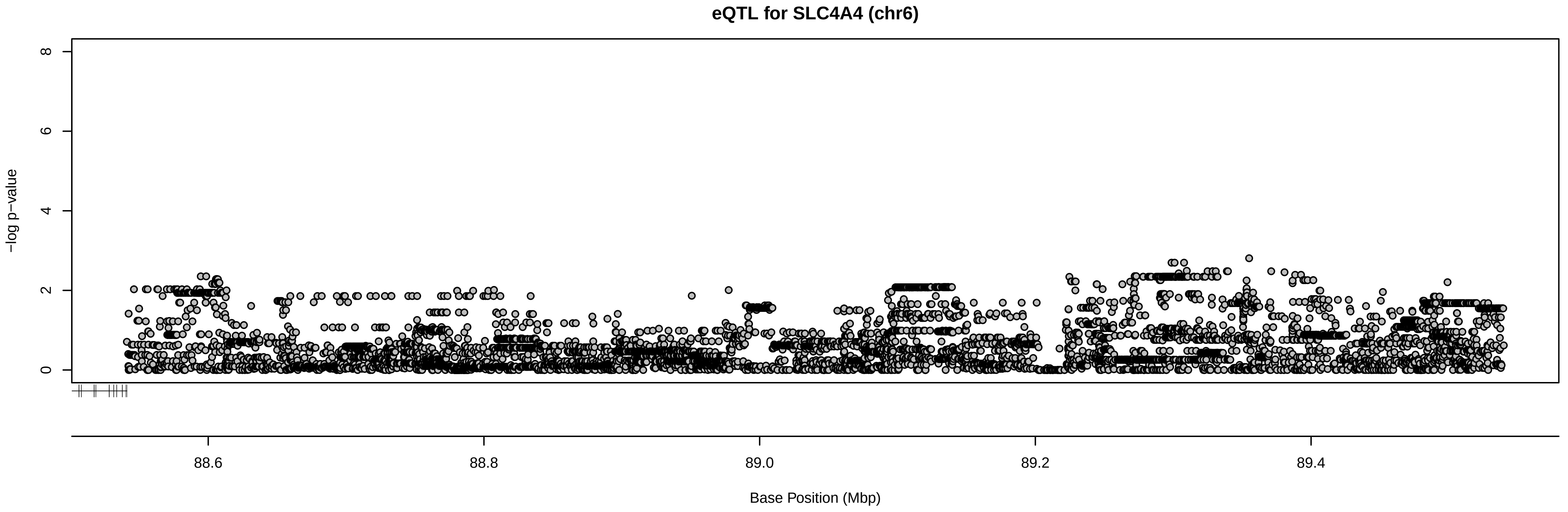
eQTL for SLC4A4 (chr6)
−log p−value
0
2
4
6
8
88.6
88.8
89.0
89.2
89.4
Base Position (Mbp)

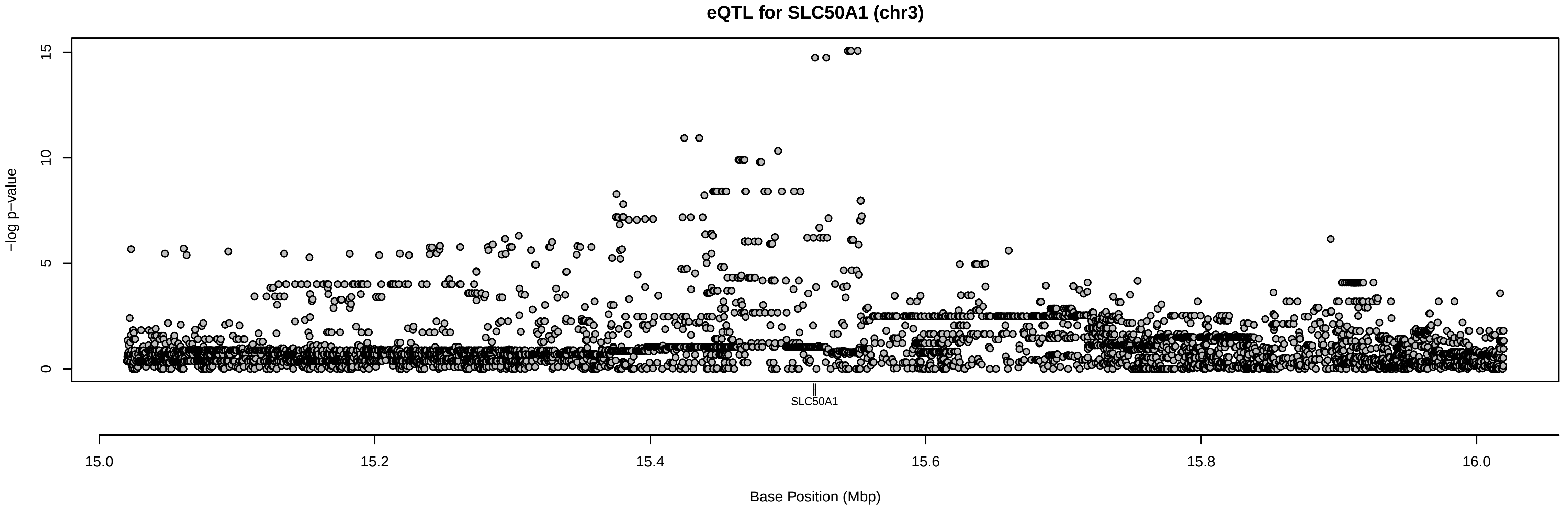
eQTL for SLC50A1 (chr3)
−log p−value
0
5
10
15
SLC50A1
15.0
15.2
15.4
15.6
15.8
16.0
Base Position (Mbp)

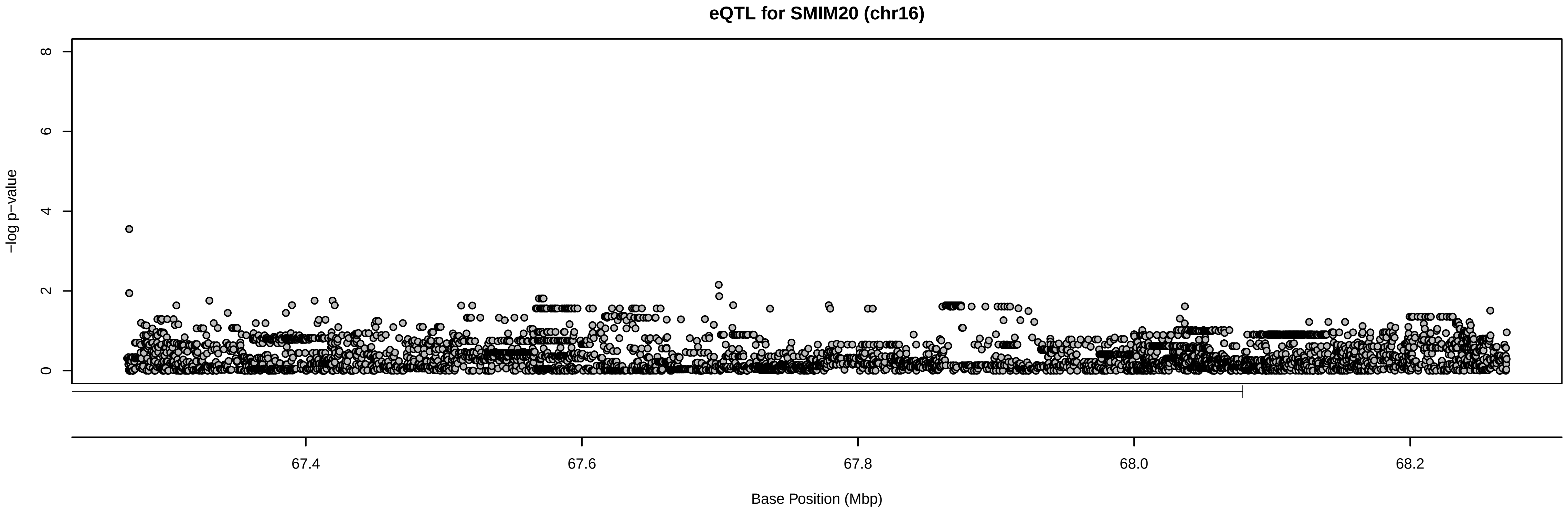
eQTL for SMIM20 (chr16)
8
6
4
2
0
−log p−value
67.4
67.6
67.8
68.0
68.2
Base Position (Mbp)

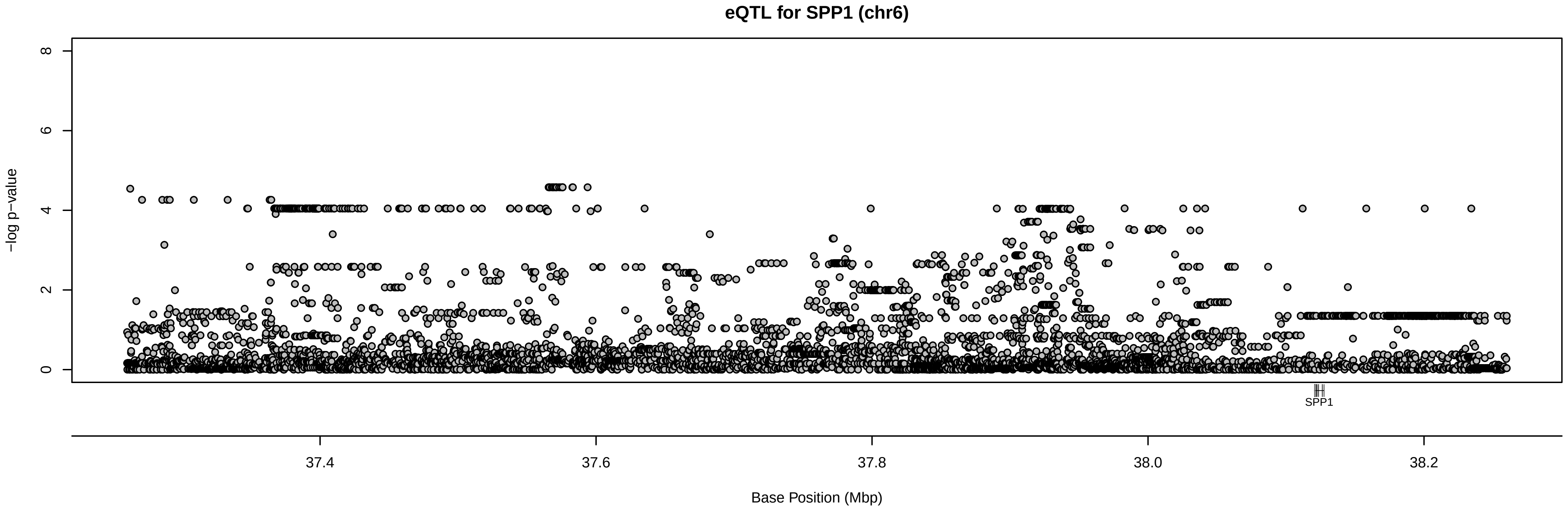
eQTL for SPP1 (chr6)
8
6
4
2
0
−log p−value
SPP1
37.4
37.6
37.8
38.0
38.2
Base Position (Mbp)

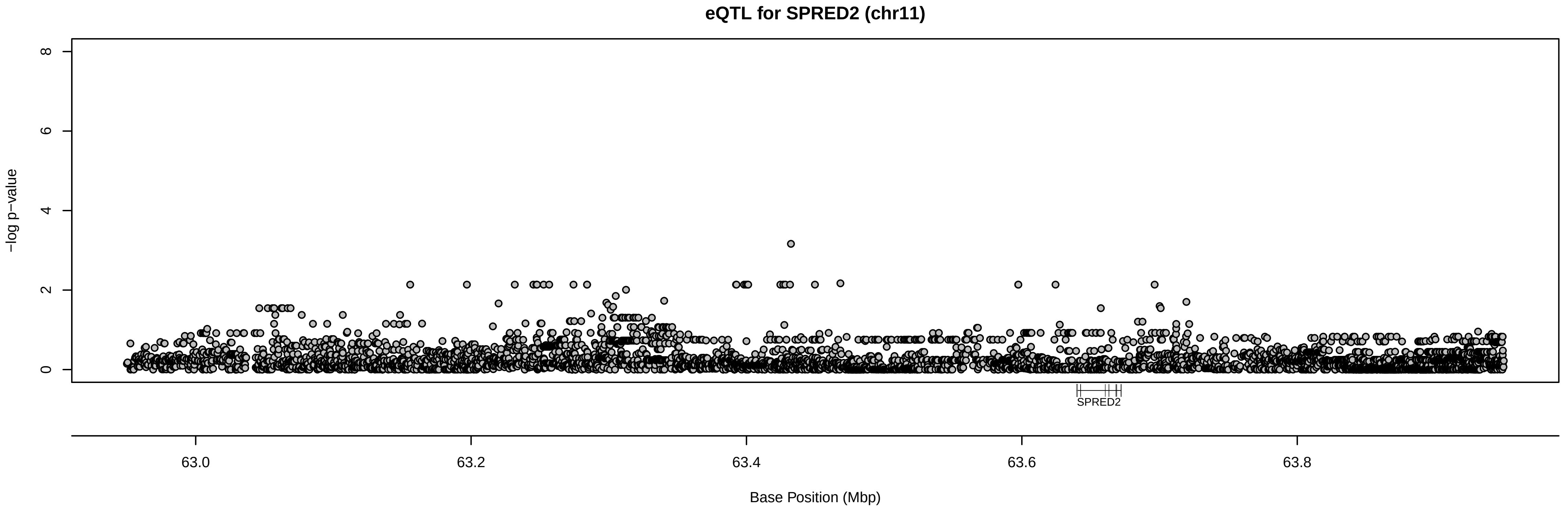
eQTL for SPRED2 (chr11)
−log p−value
0
2
4
6
8
SPRED2
63.0
63.2
63.4
63.6
63.8
Base Position (Mbp)

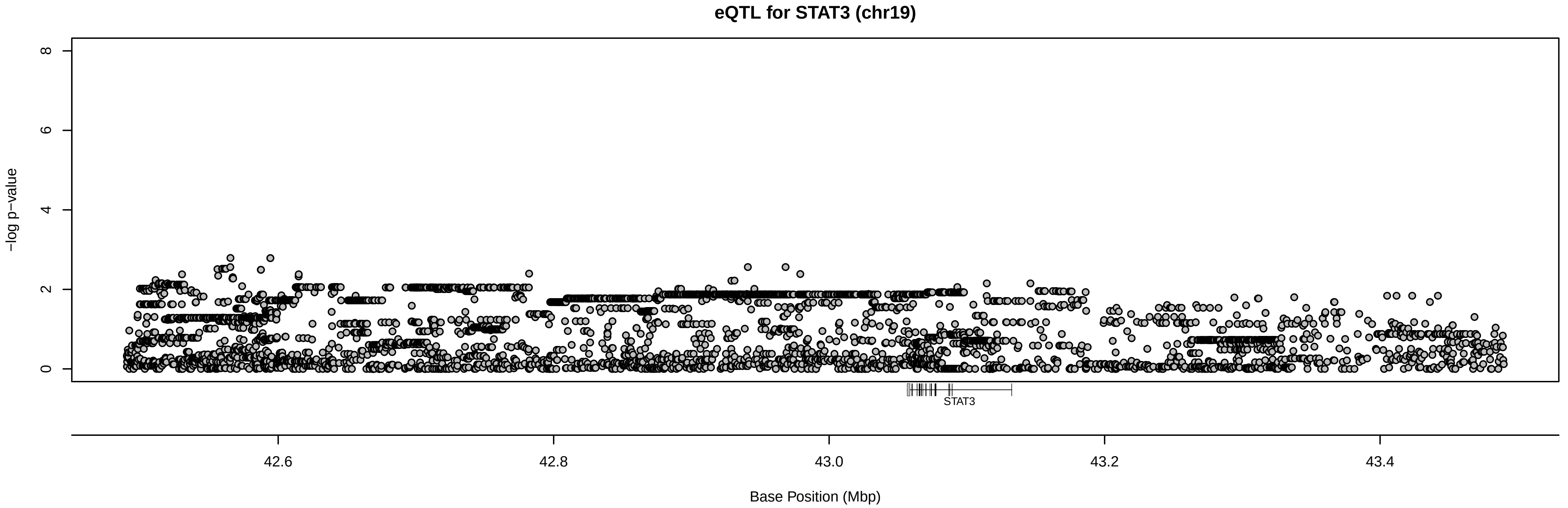
eQTL for STAT3 (chr19)
−log p−value
0
2
4
6
8
STAT3
42.6
42.8
43.0
43.2
43.4
Base Position (Mbp)

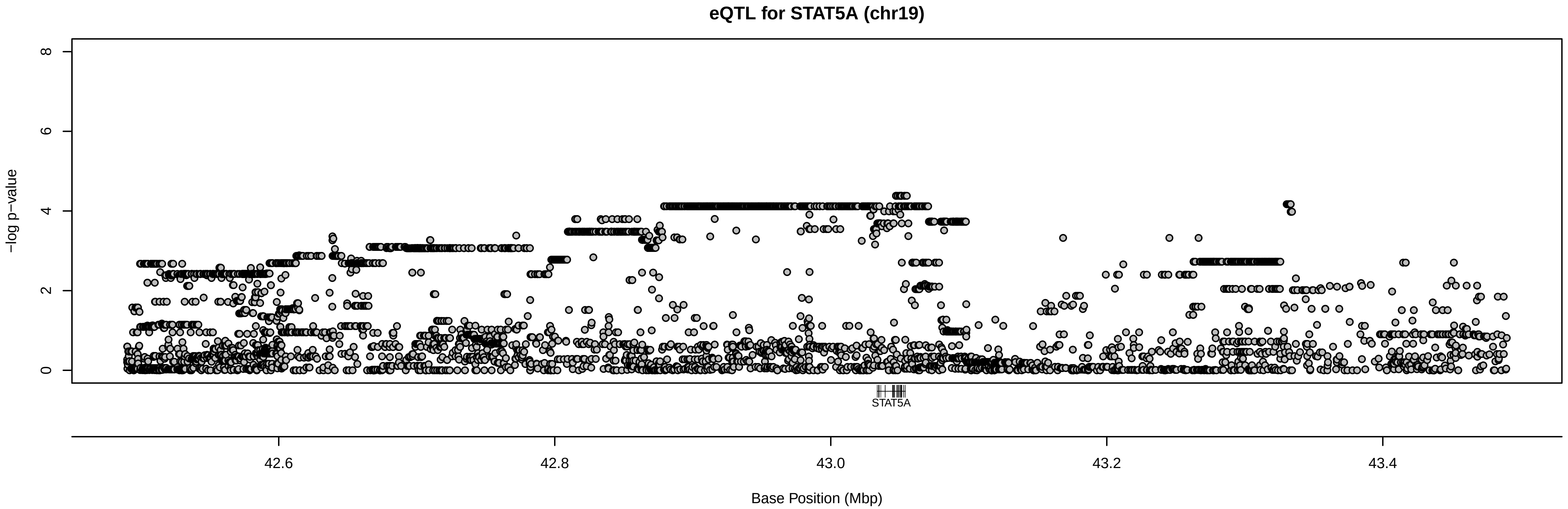

eQTL for STAT5A (chr19)
−log p−value
0
2
4
6
8
STAT5A
42.6
42.8
43.0
43.2
43.4
Base Position (Mbp)

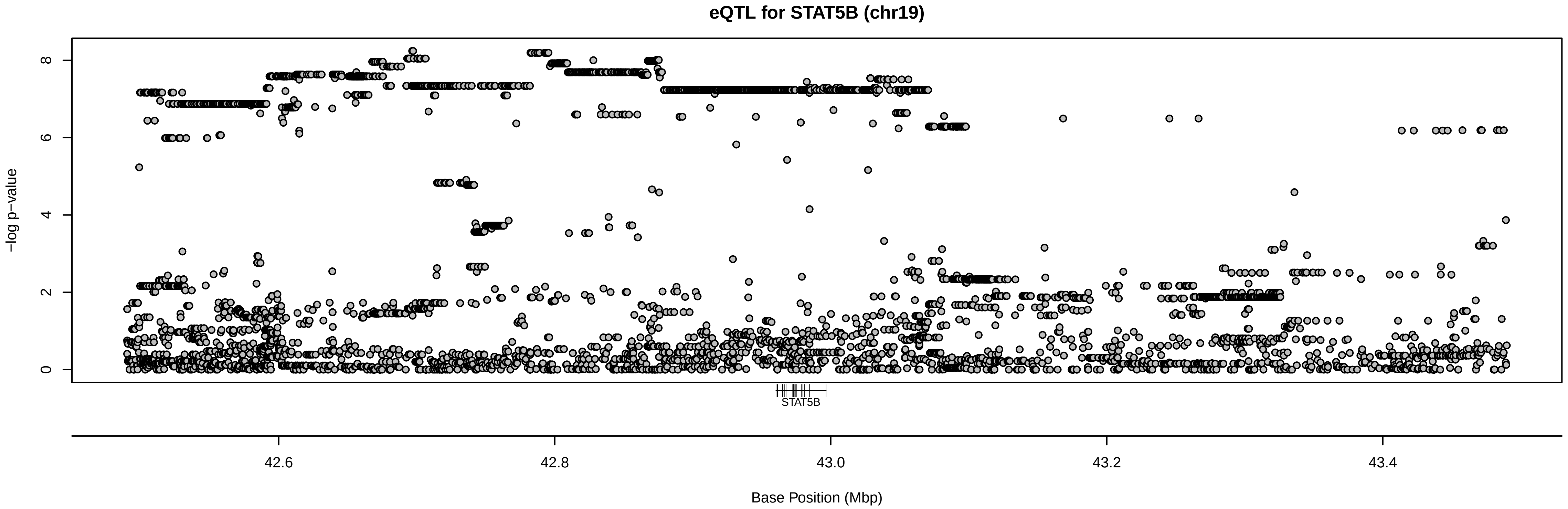

eQTL for STAT5B (chr19)
−log p−value
0
2
4
6
8
STAT5B
42.6
42.8
43.0
43.2
43.4
Base Position (Mbp)

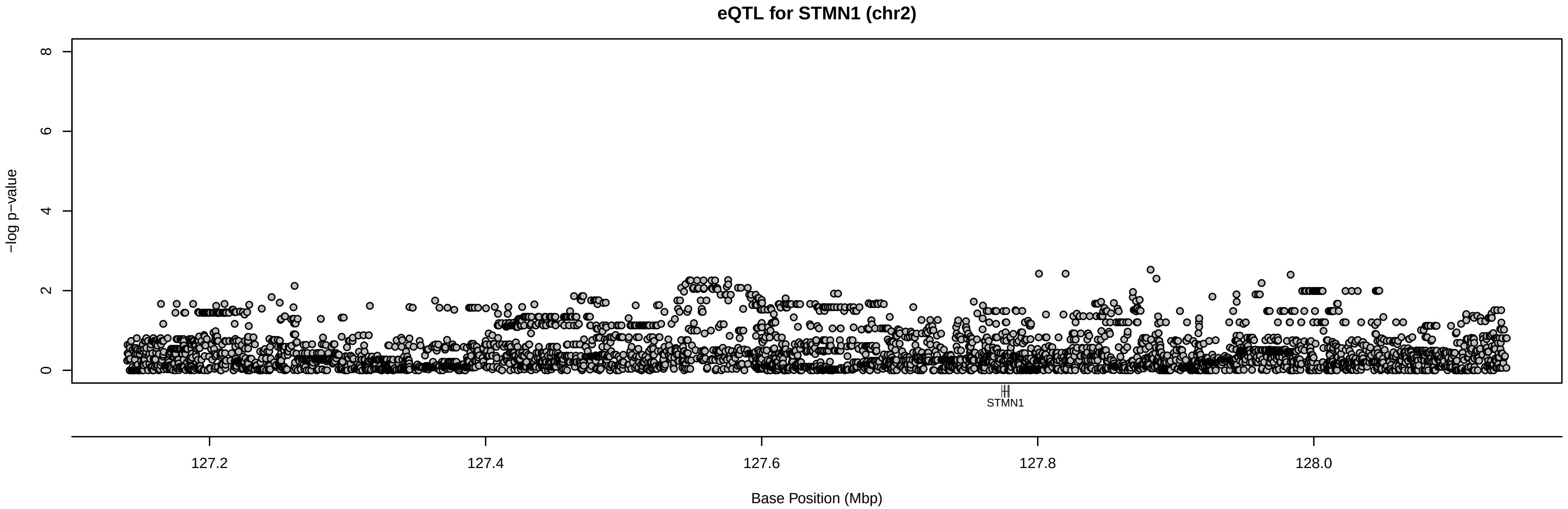
eQTL for STMN1 (chr2)
−log p−value
0
2
4
6
8
STMN1
127.2
127.4
127.6
127.8
128.0
Base Position (Mbp)

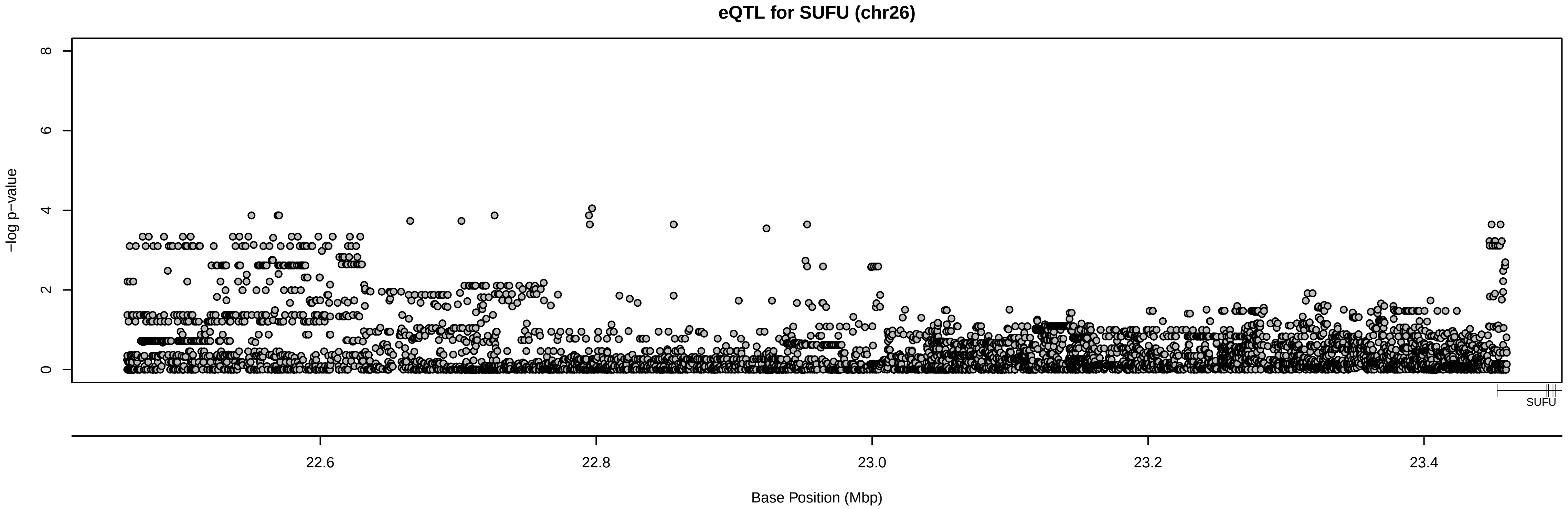
eQTL for SUFU (chr26)
−log p−value
0
2
4
6
8
SUFU
22.6
22.8
23.0
23.2
23.4
Base Position (Mbp)

eQTL for SYDE1 (chr7)

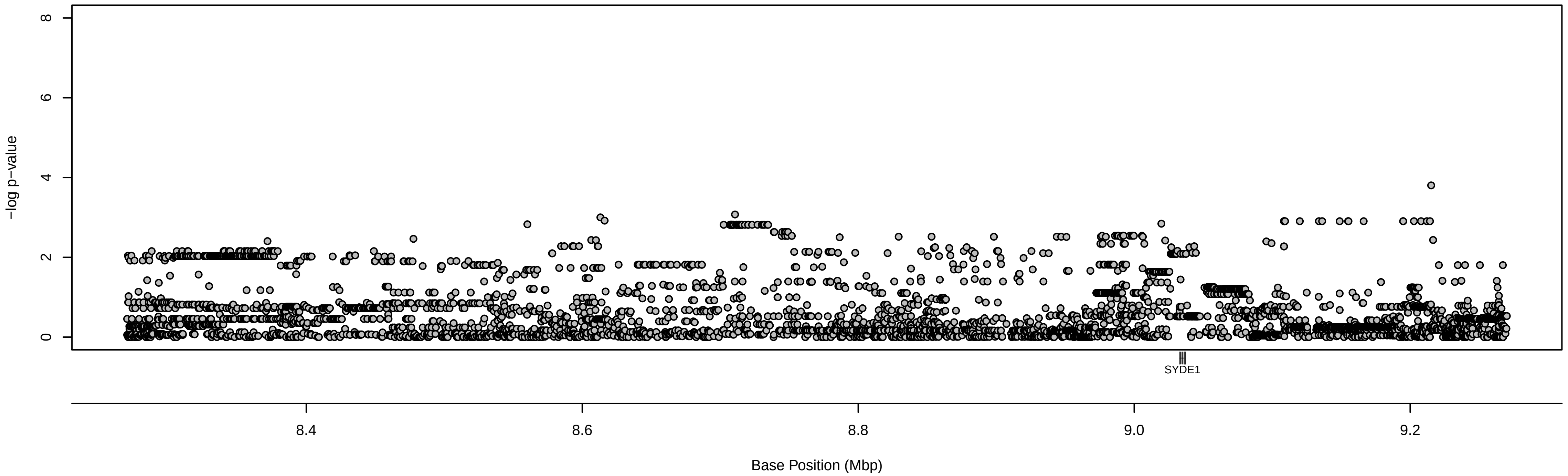

eQTL for SYTL2 (chr29)

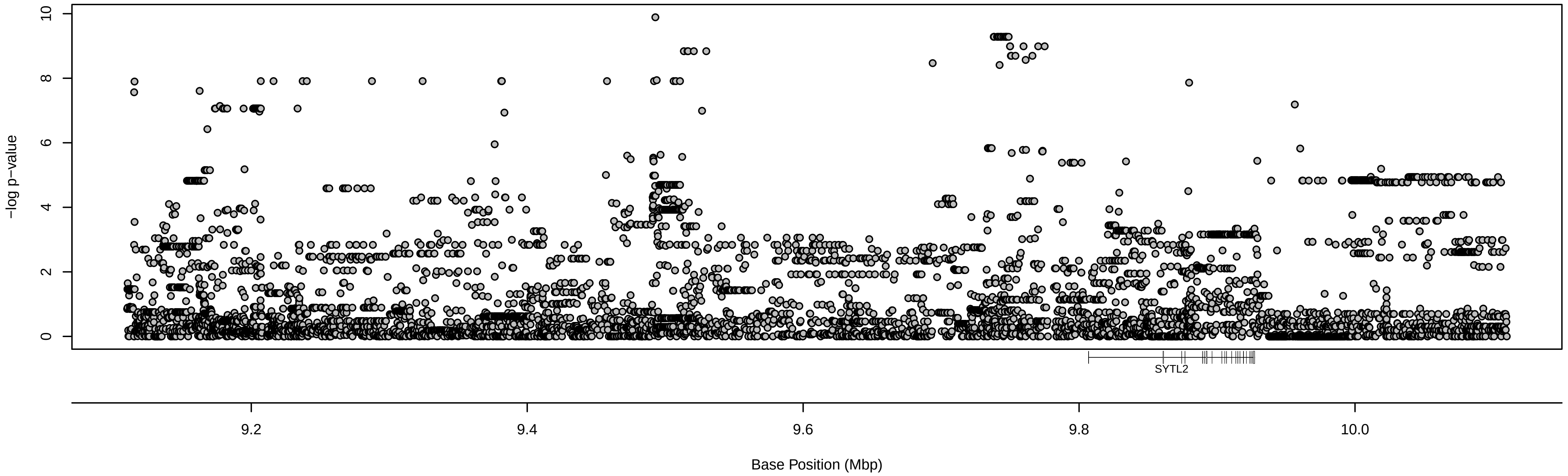

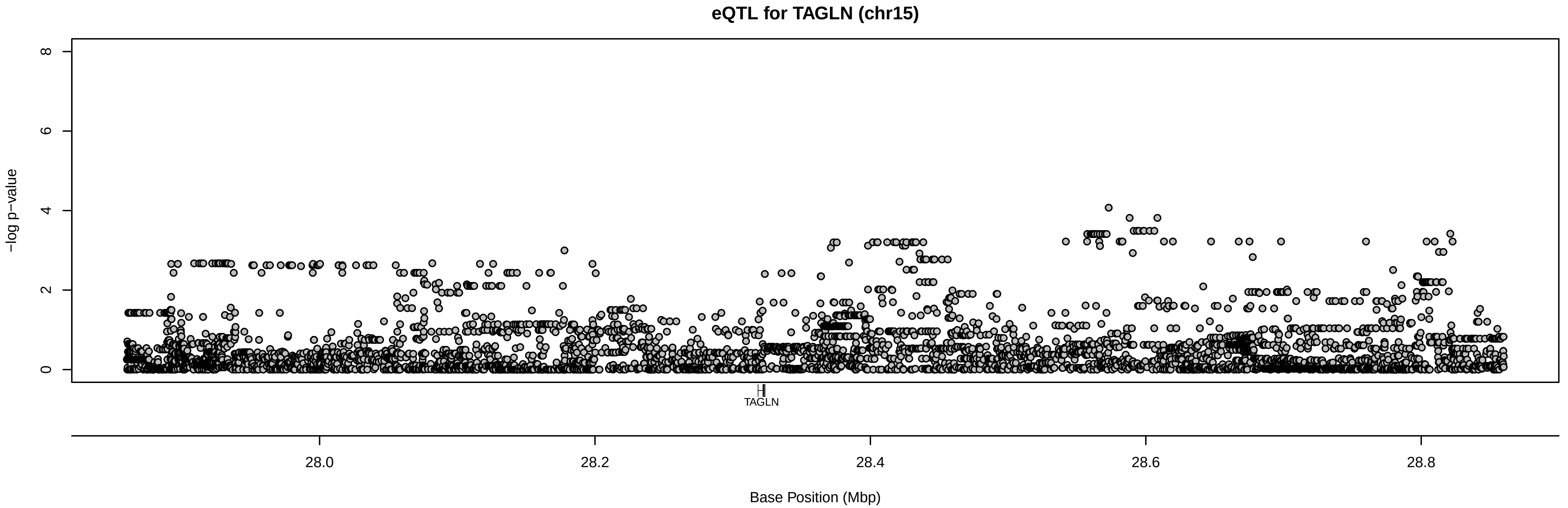
eQTL for TAGLN (chr15)
−log p−value
0
2
4
6
8
TAGLN
28.0
28.2
28.4
28.6
28.8
Base Position (Mbp)

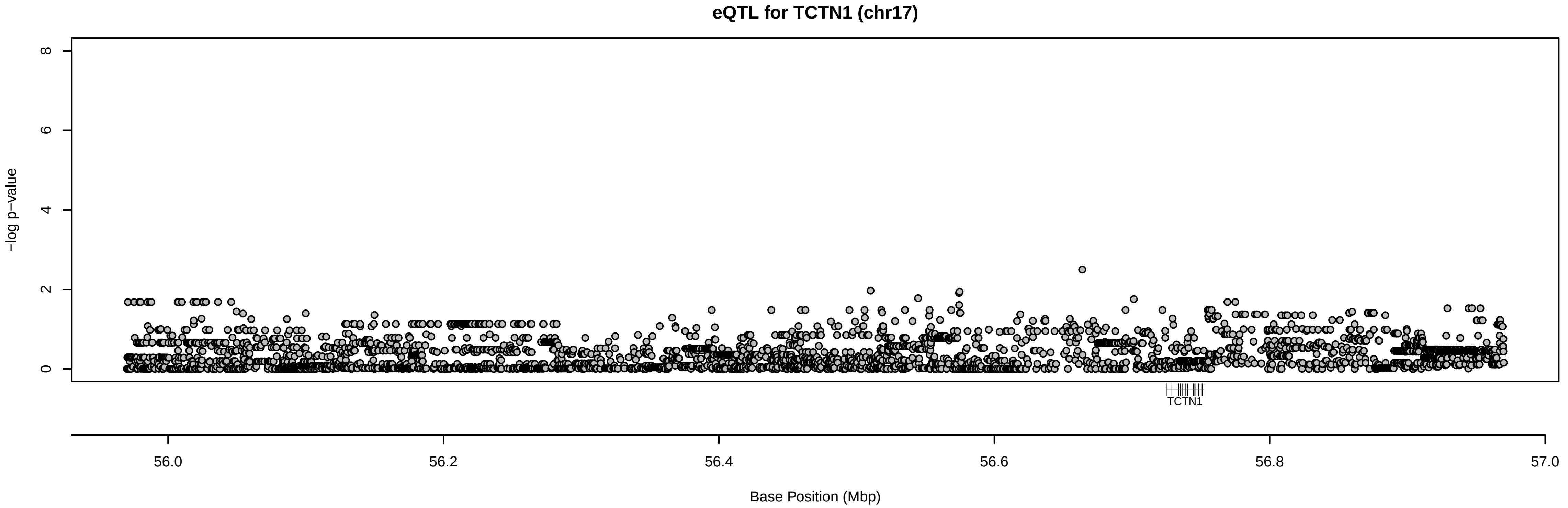
eQTL for TCTN1 (chr17)
−log p−value
0
2
4
6
8
TCTN1
56.0
56.2
56.4
56.6
56.8
57.0
Base Position (Mbp)

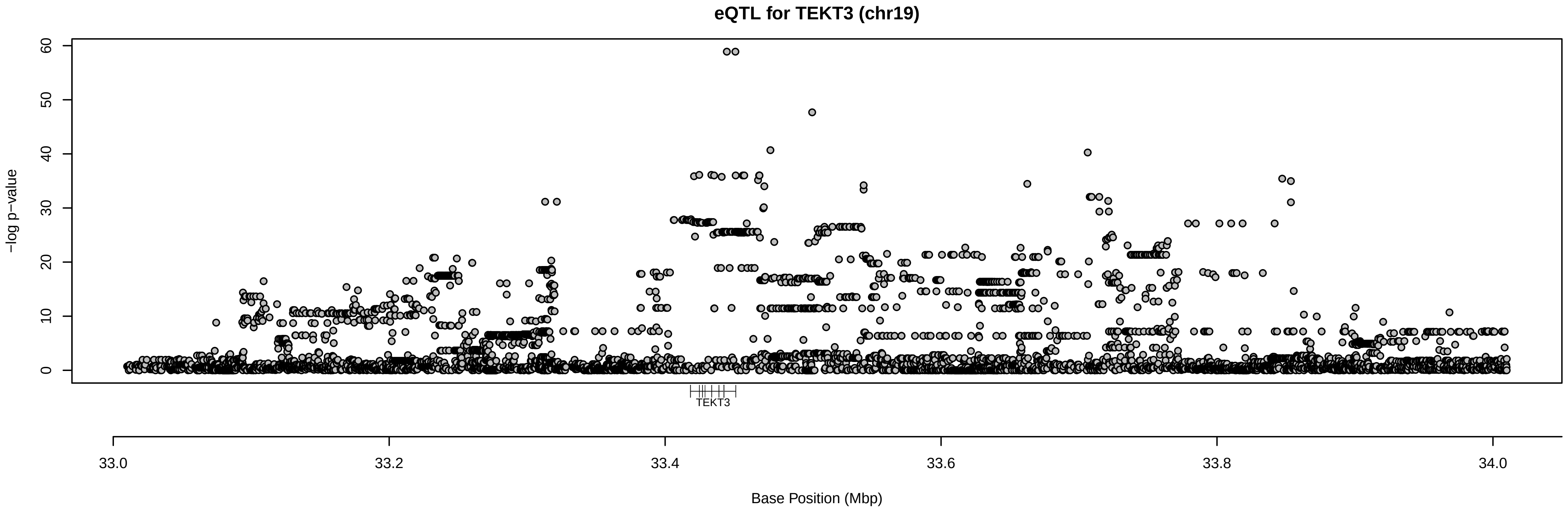
eQTL for TEKT3 (chr19)
−log p−value
0
10
20
30
40
50
60
TEKT3
33.0
33.2
33.4
33.6
33.8
34.0
Base Position (Mbp)

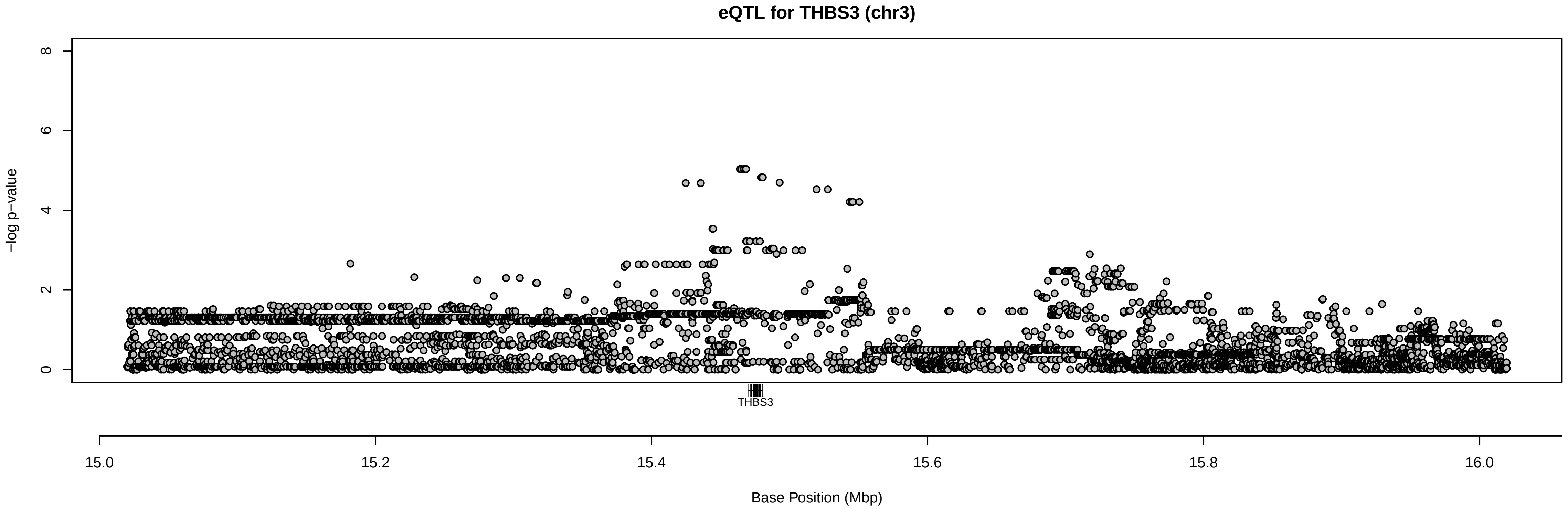
eQTL for THBS3 (chr3)
−log p−value
0
2
4
6
8
THBS3
15.0
15.2
15.4
15.6
15.8
16.0
Base Position (Mbp)

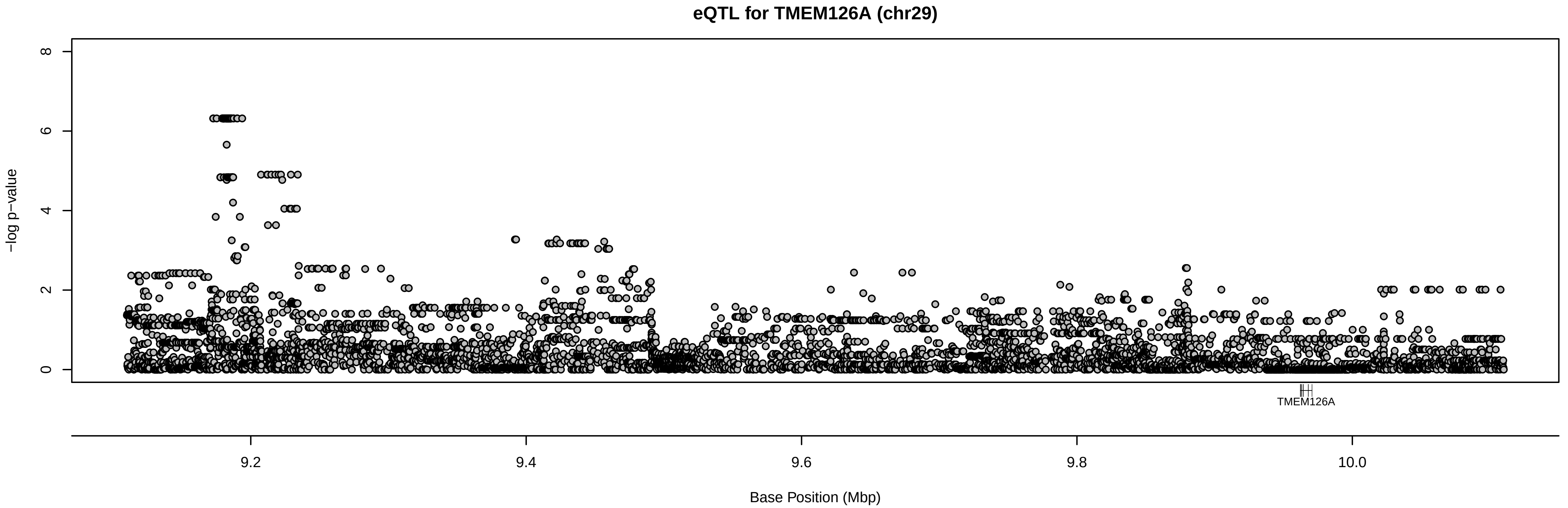
eQTL for TMEM126A (chr29)
−log p−value
0
2
4
6
8
TMEM126A
9.2
9.4
9.6
9.8
10.0
Base Position (Mbp)

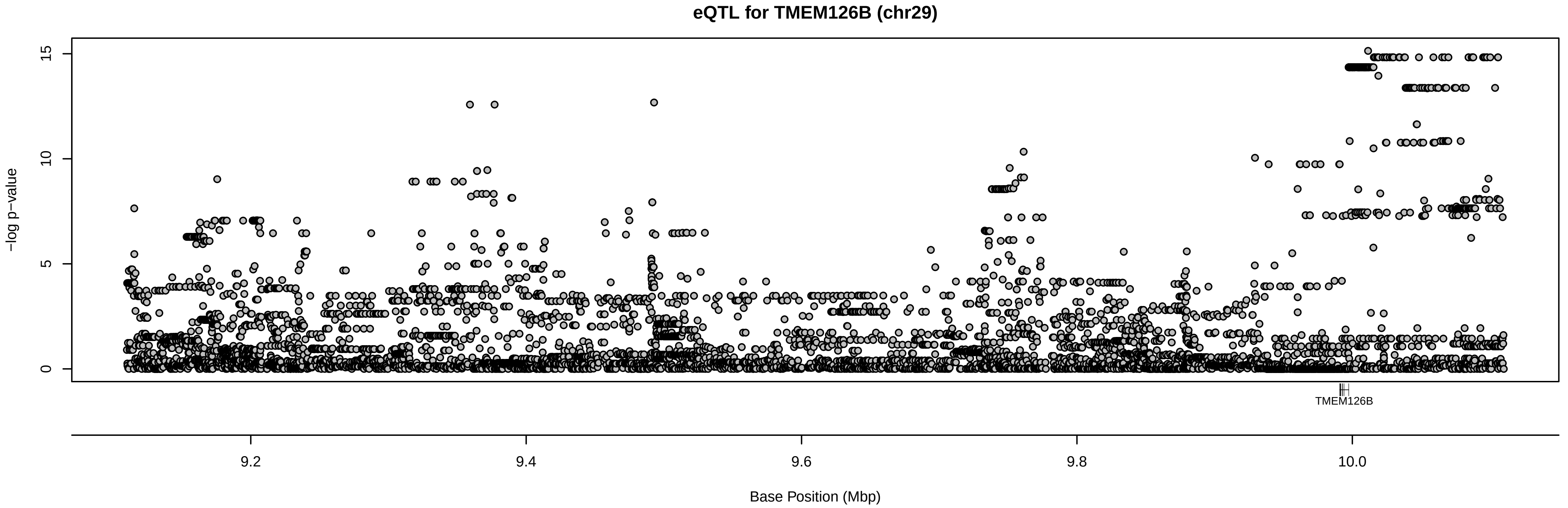
eQTL for TMEM126B (chr29)
−log p−value
0
5
10
15
TMEM126B
9.2
9.4
9.6
9.8
10.0
Base Position (Mbp)

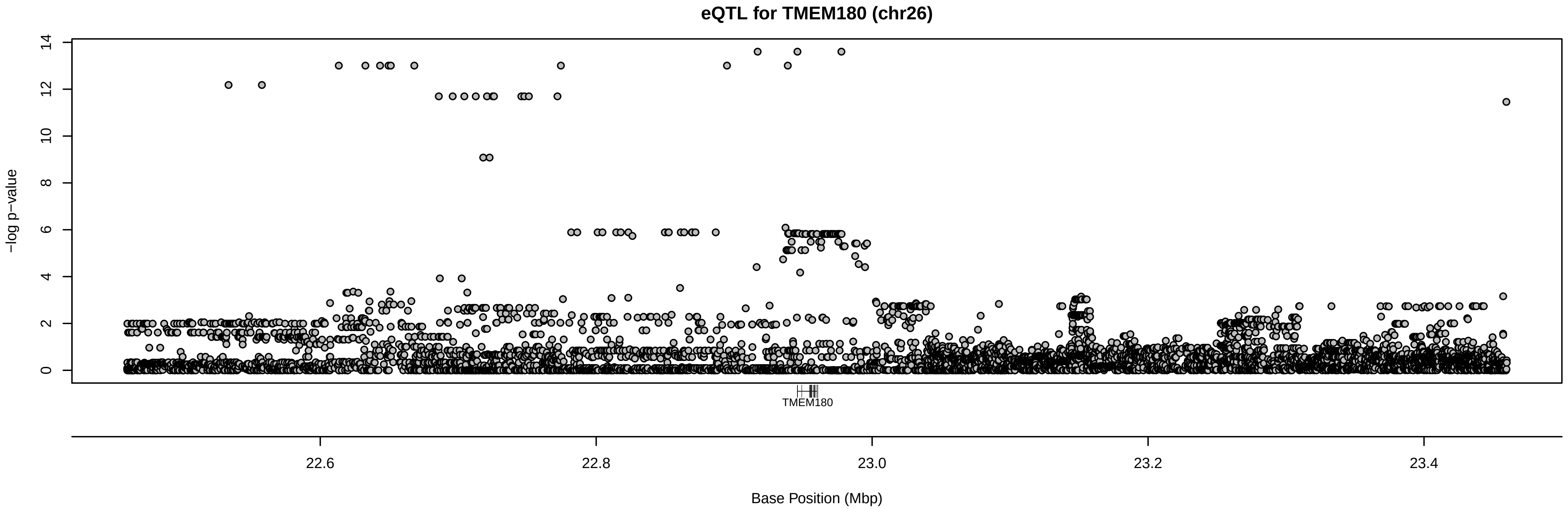

eQTL for TMEM180 (chr26)
−log p−value
0
2
4
6
8
10
12
14
TMEM180
22.6
22.8
23.0
23.2
23.4
Base Position (Mbp)

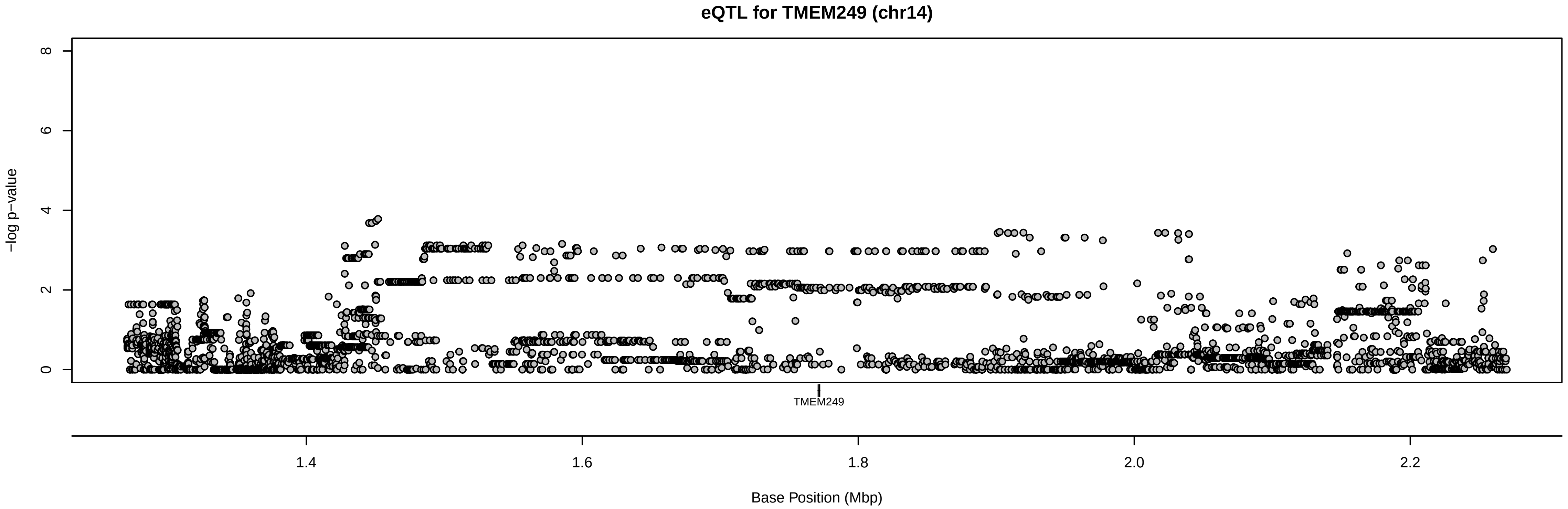

eQTL for TMEM249 (chr14)
−log p−value
0
2
4
6
8
TMEM249
1.4
1.6
1.8
2.0
2.2
Base Position (Mbp)

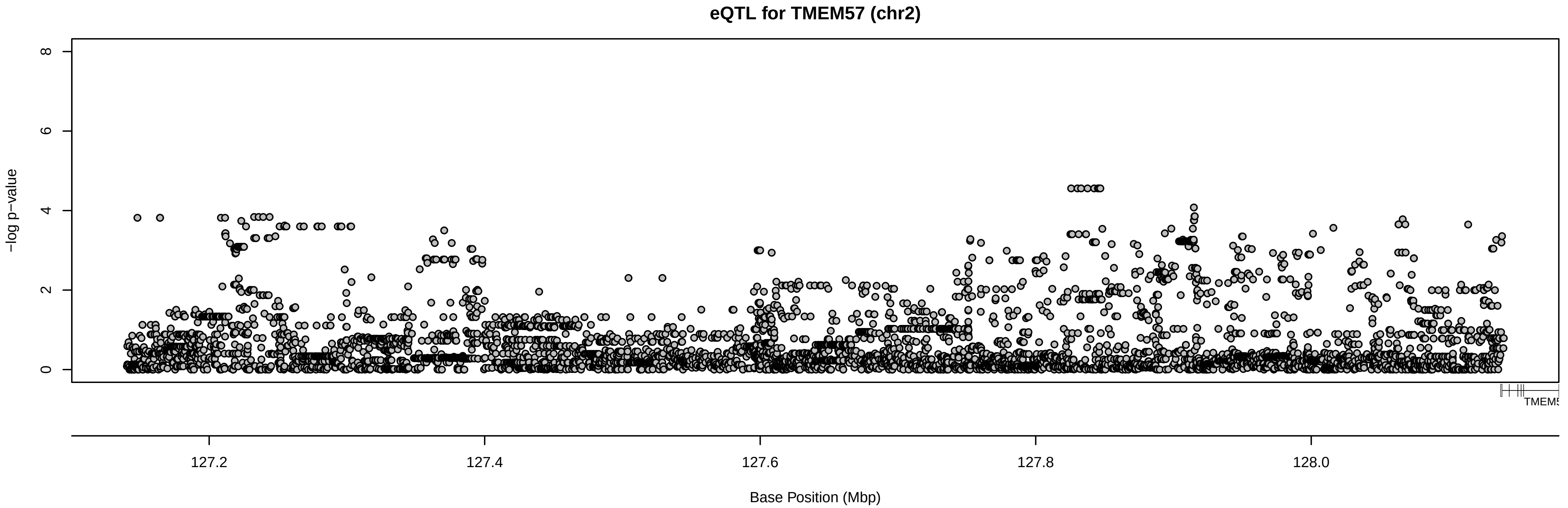

eQTL for TMEM57 (chr2)
−log p−value
0
2
4
6
8
TMEM5
127.2
127.4
127.6
127.8
128.0
Base Position (Mbp)

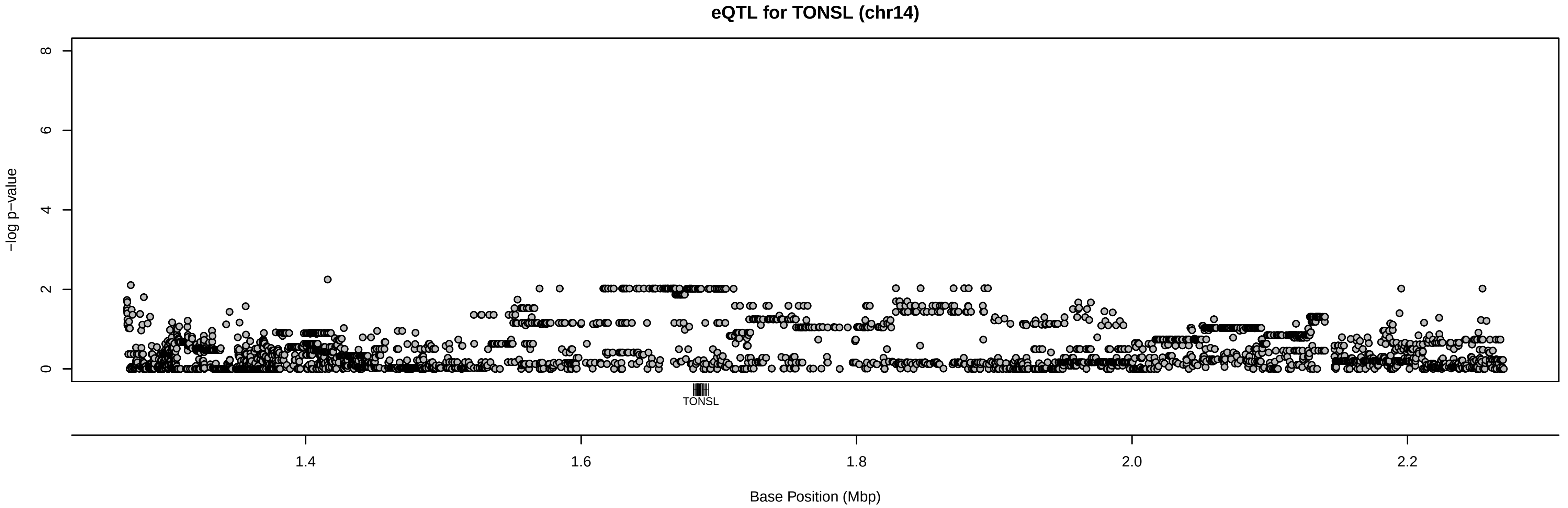

eQTL for TONSL (chr14)
−log p−value
0
2
4
6
8
TONSL
1.4
1.6
1.8
2.0
2.2
Base Position (Mbp)

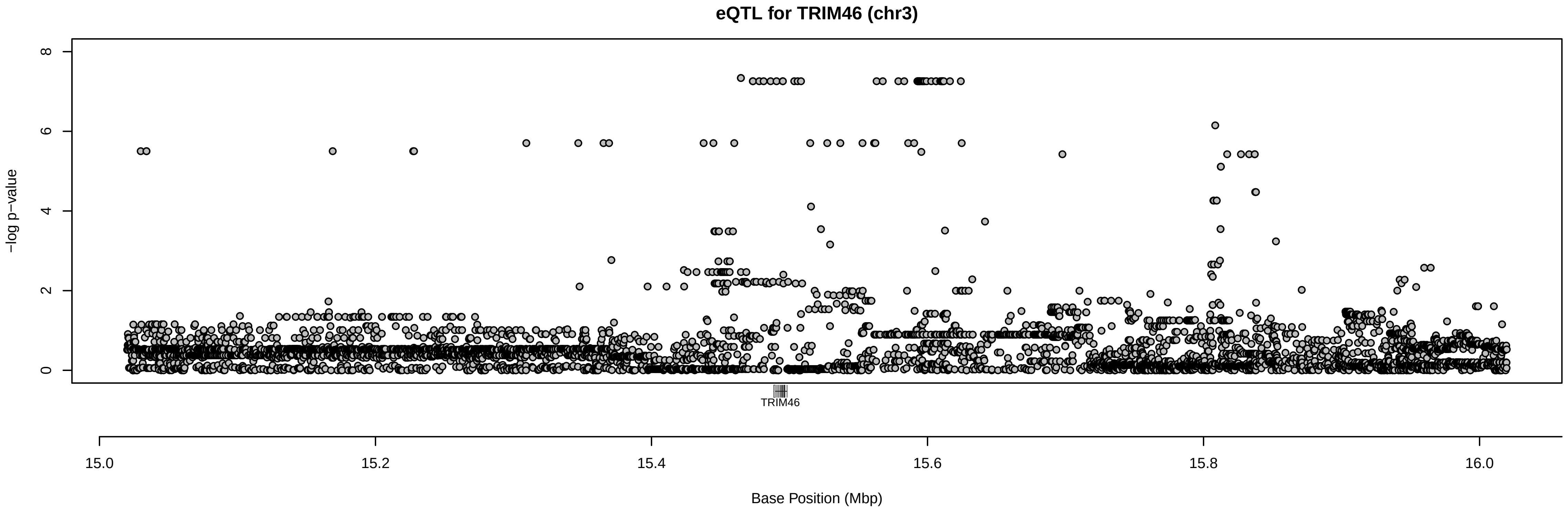
eQTL for TRIM46 (chr3)
−log p−value
0
2
4
6
8
TRIM46
15.0
15.2
15.4
15.6
15.8
16.0
Base Position (Mbp)

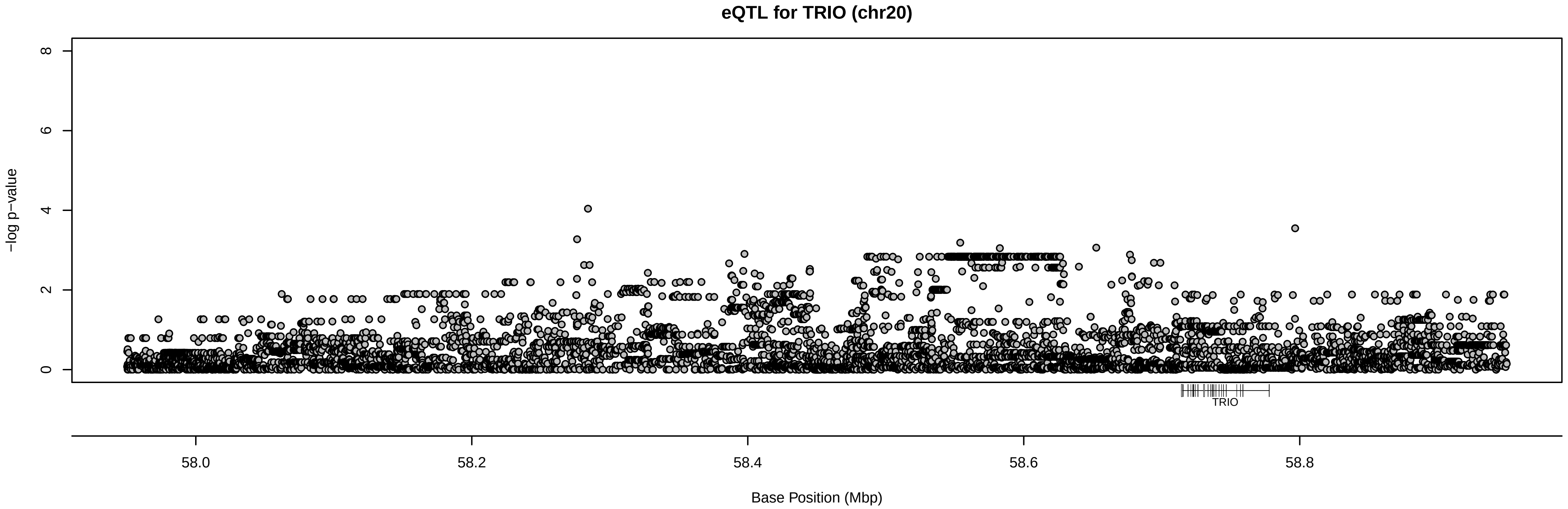
eQTL for TRIO (chr20)
−log p−value
0
2
4
6
8
TRIO
58.0
58.2
58.4
58.6
58.8
Base Position (Mbp)

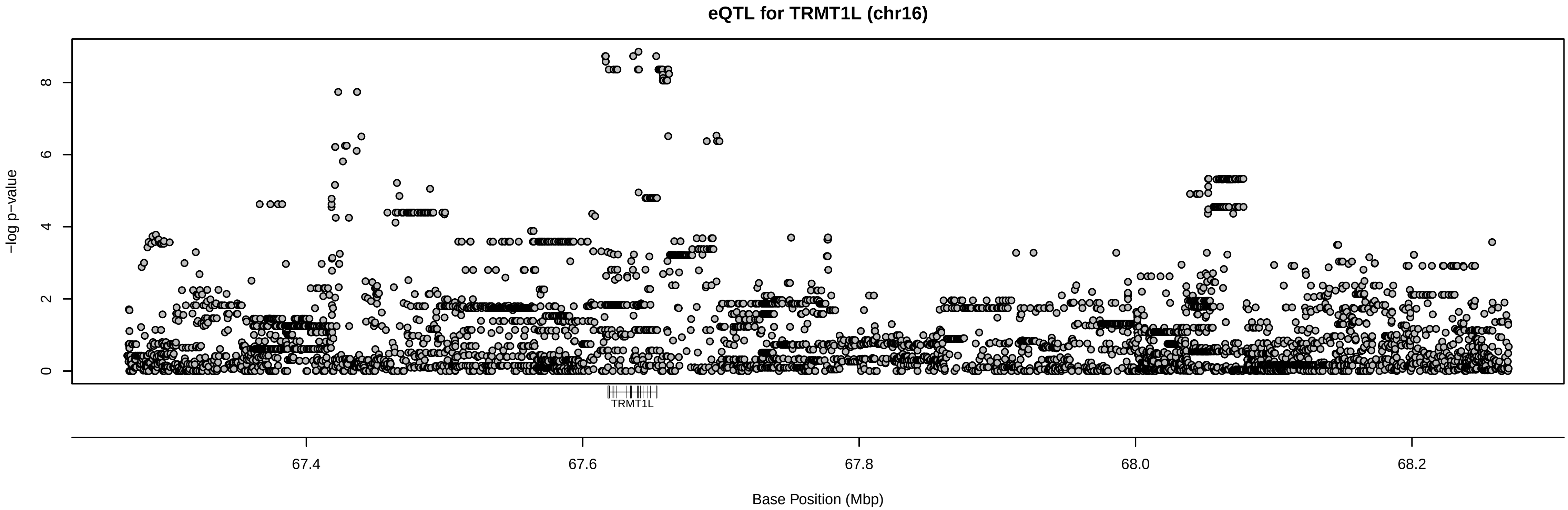
eQTL for TRMT1L (chr16)
−log p−value
0
2
4
6
8
TRMT1L
67.4
67.6
67.8
68.0
68.2
Base Position (Mbp)

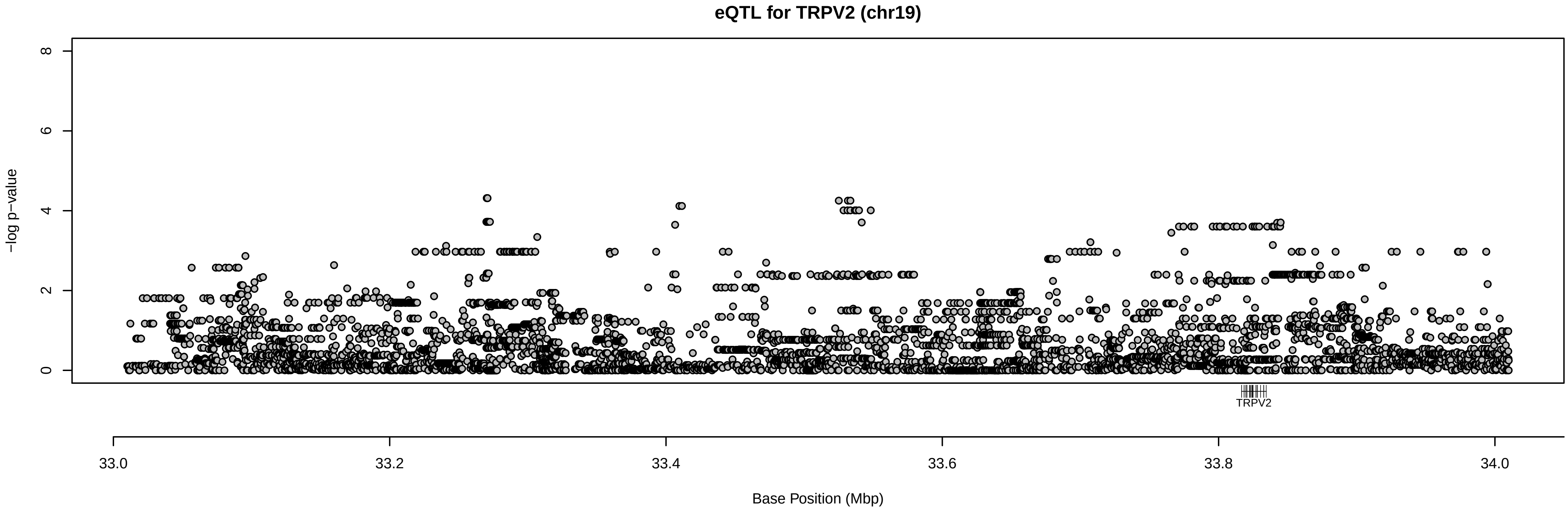
eQTL for TRPV2 (chr19)
−log p−value
0
2
4
6
8
TRPV2
33.0
33.2
33.4
33.6
33.8
34.0
Base Position (Mbp)

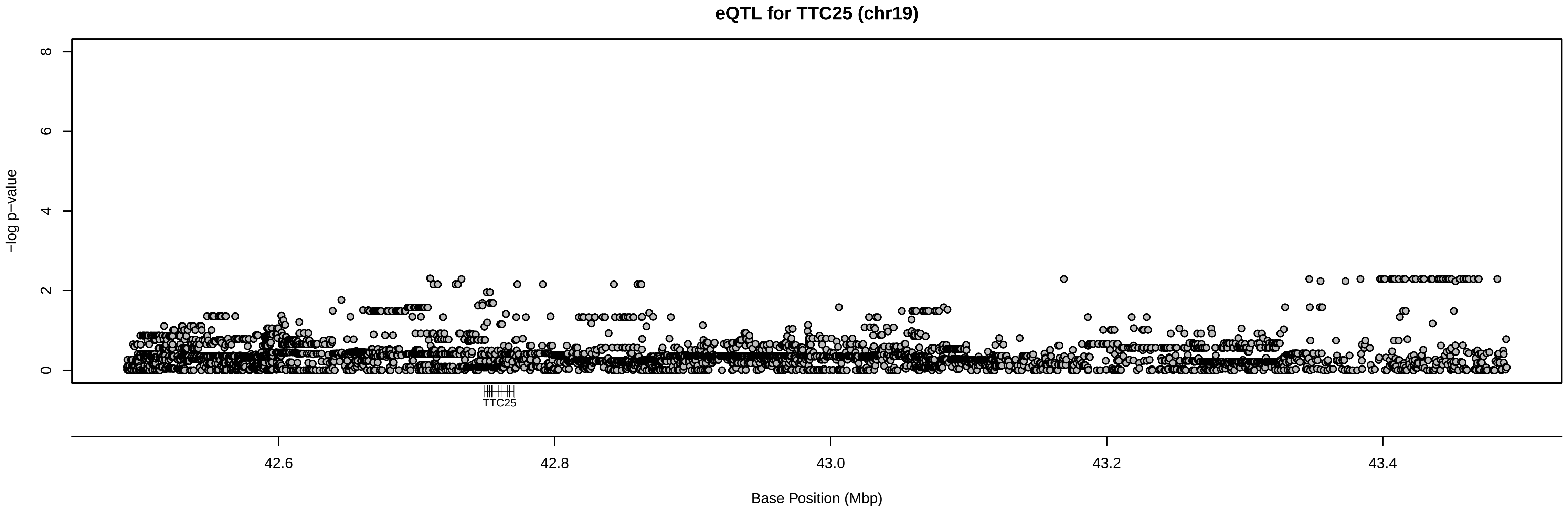
eQTL for TTC25 (chr19)
−log p−value
0
2
4
6
8
TTC25
42.6
42.8
43.0
43.2
43.4
Base Position (Mbp)

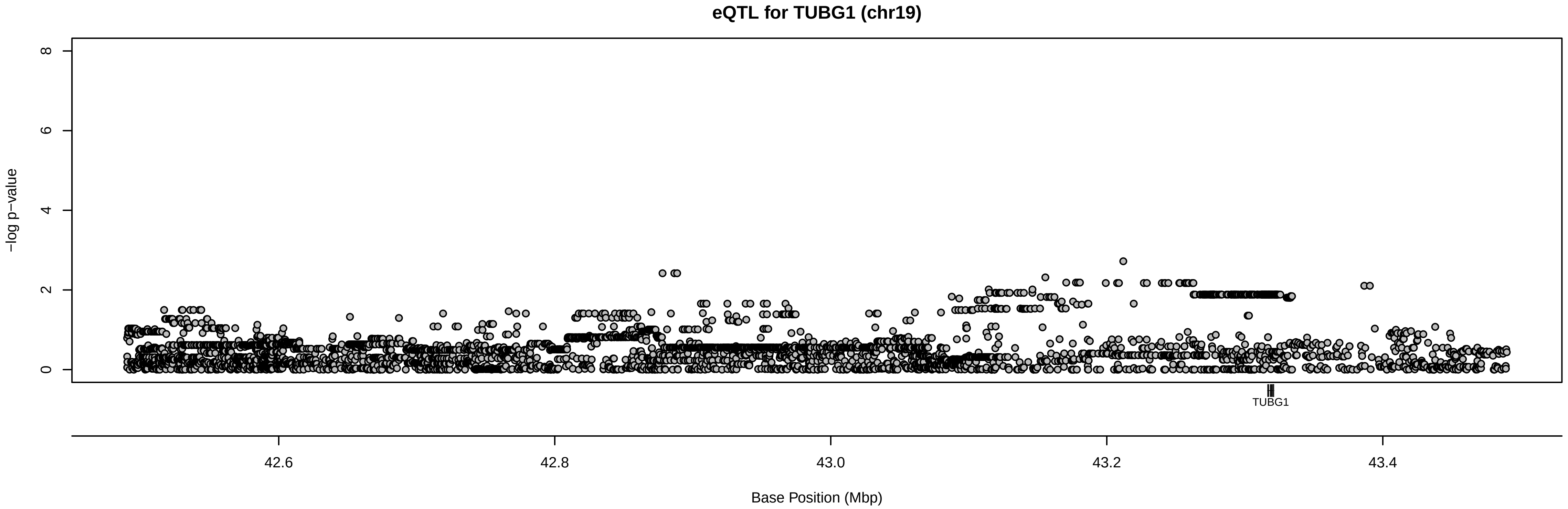
eQTL for TUBG1 (chr19)
−log p−value
0
2
4
6
8
TUBG1
42.6
42.8
43.0
43.2
43.4
Base Position (Mbp)

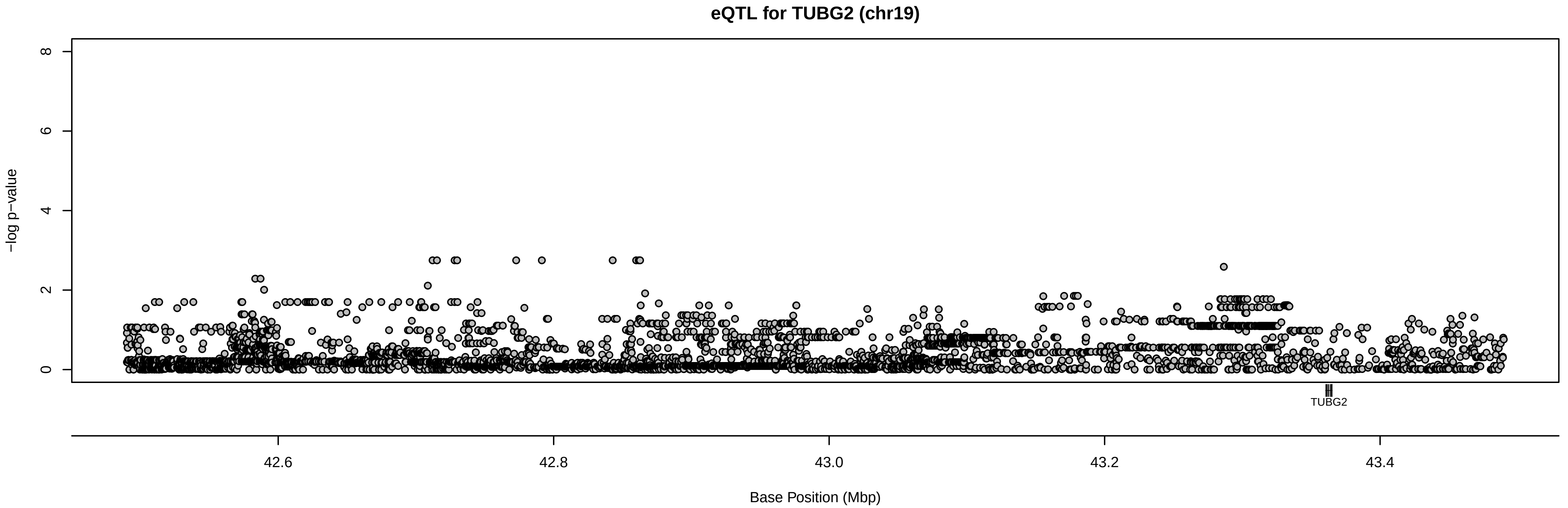
eQTL for TUBG2 (chr19)
−log p−value
0
2
4
6
8
TUBG2
42.6
42.8
43.0
43.2
43.4
Base Position (Mbp)

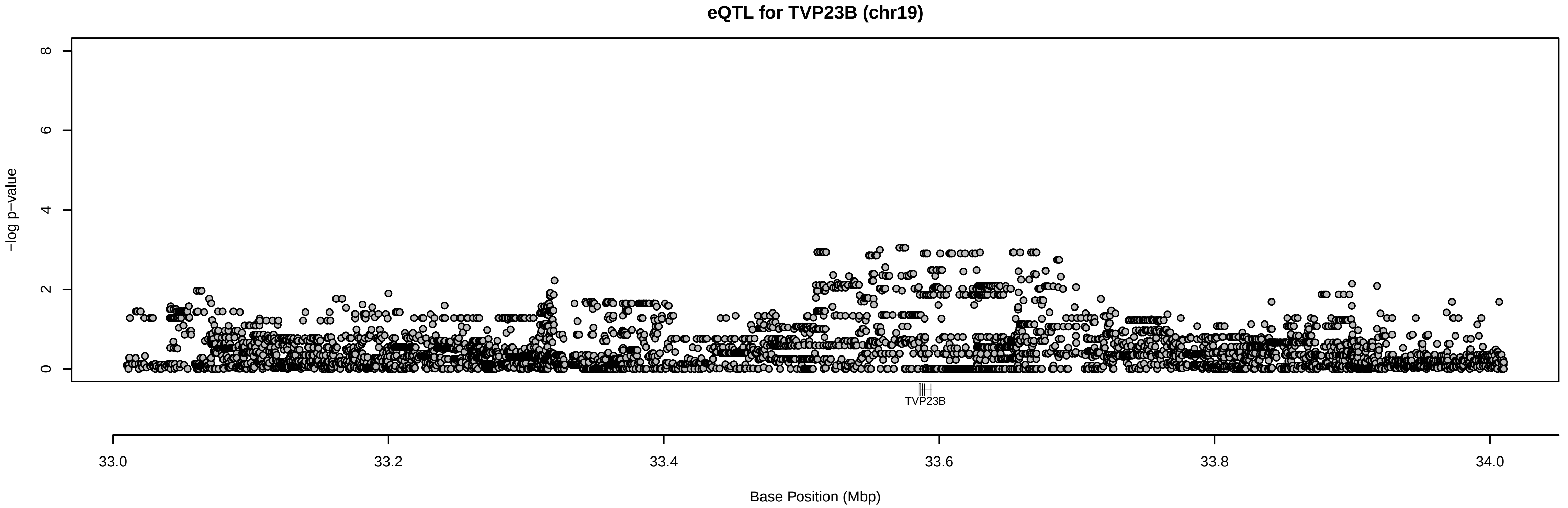
eQTL for TVP23B (chr19)
−log p−value
0
2
4
6
8
TVP23B
33.0
33.2
33.4
33.6
33.8
34.0
Base Position (Mbp)

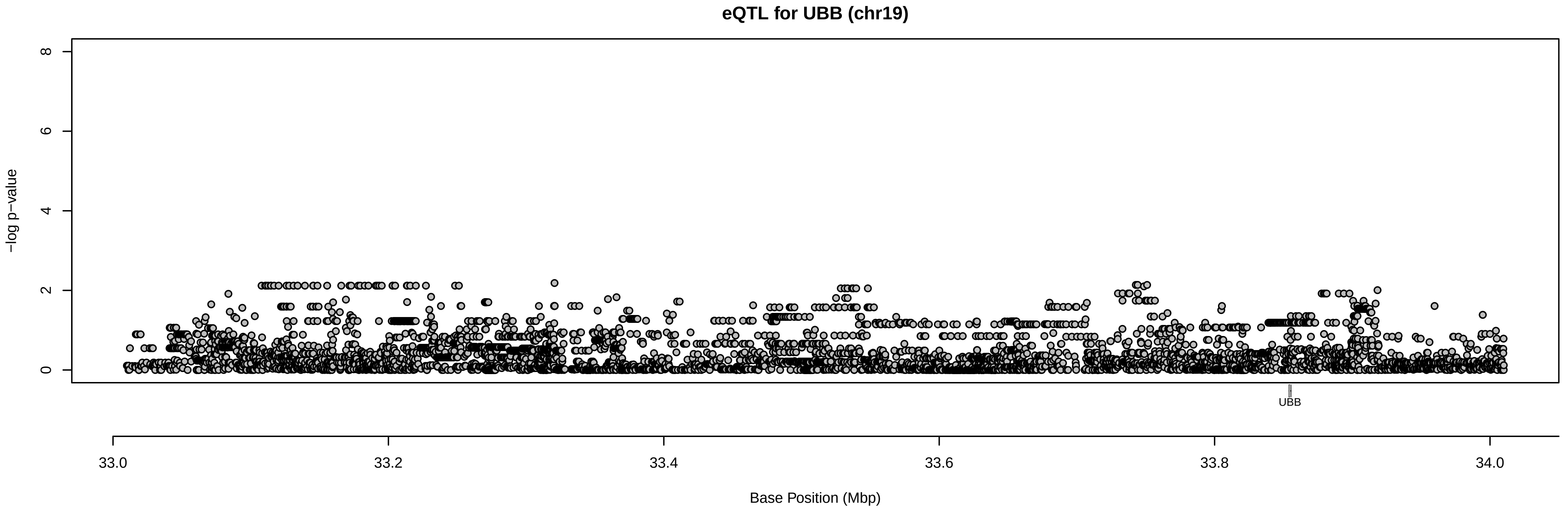

eQTL for UBB (chr19)
−log p−value
0
2
4
6
8
UBB
33.0
33.2
33.4
33.6
33.8
34.0
Base Position (Mbp)

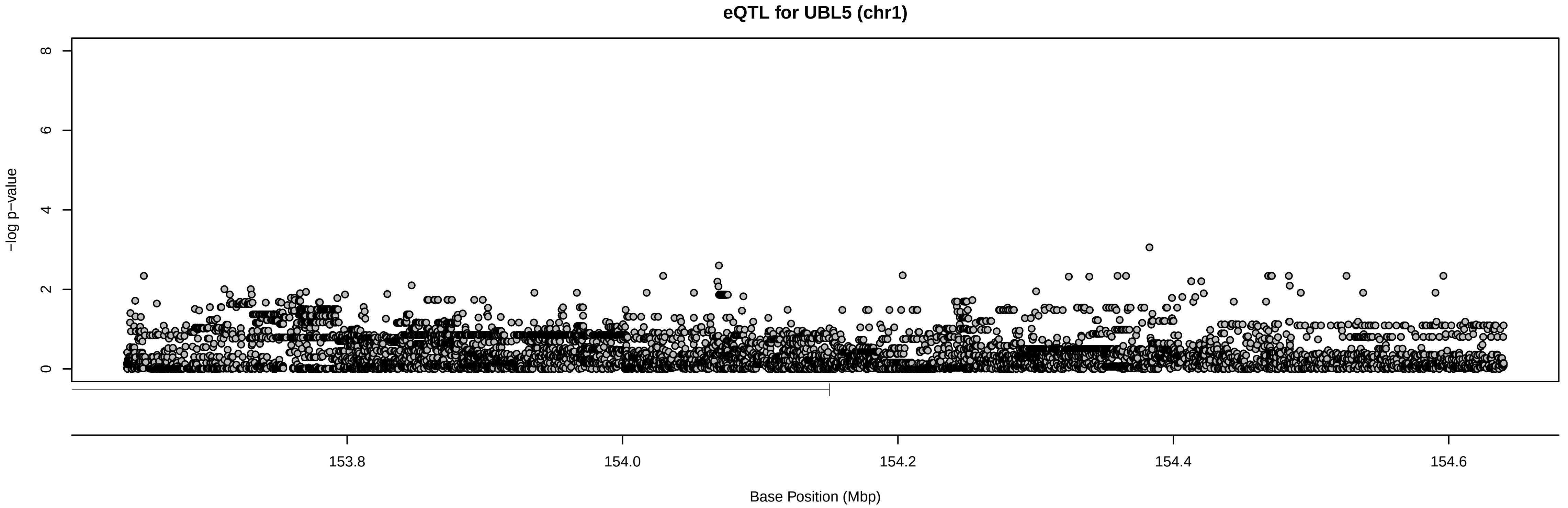

eQTL for UBL5 (chr1)
−log p−value
0
2
4
6
8
153.8
154.0
154.2
154.4
154.6
Base Position (Mbp)

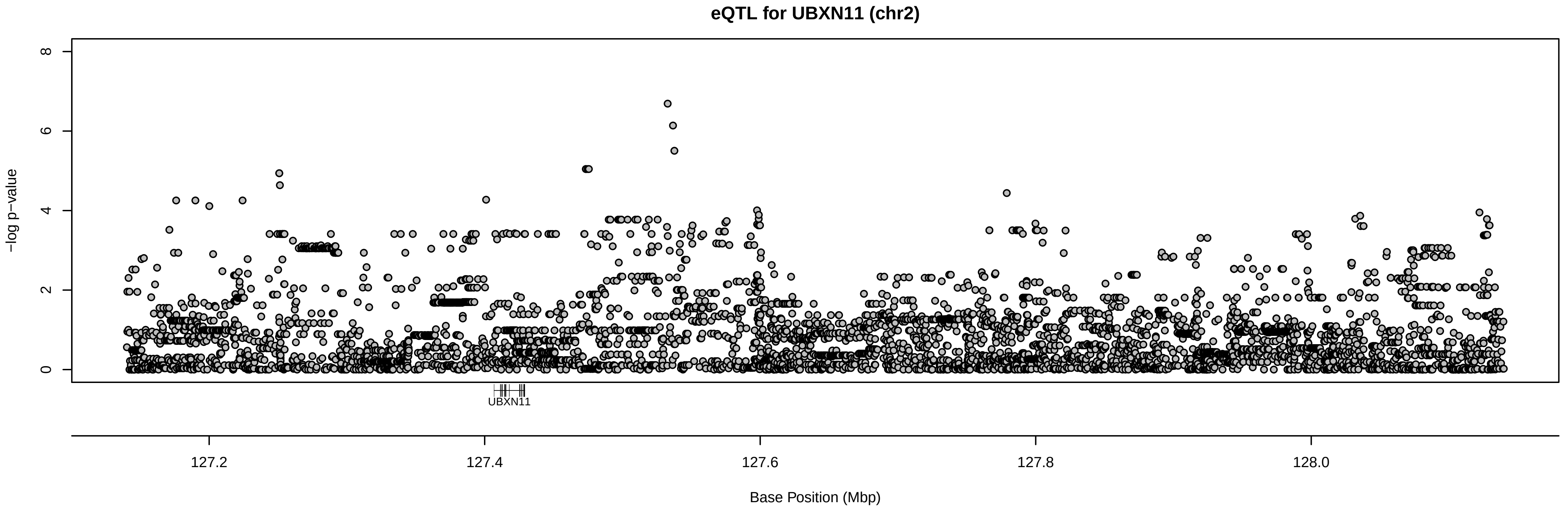
eQTL for UBXN11 (chr2)
−log p−value
0
2
4
6
8
UBXN11
127.2
127.4
127.6
127.8
128.0
Base Position (Mbp)

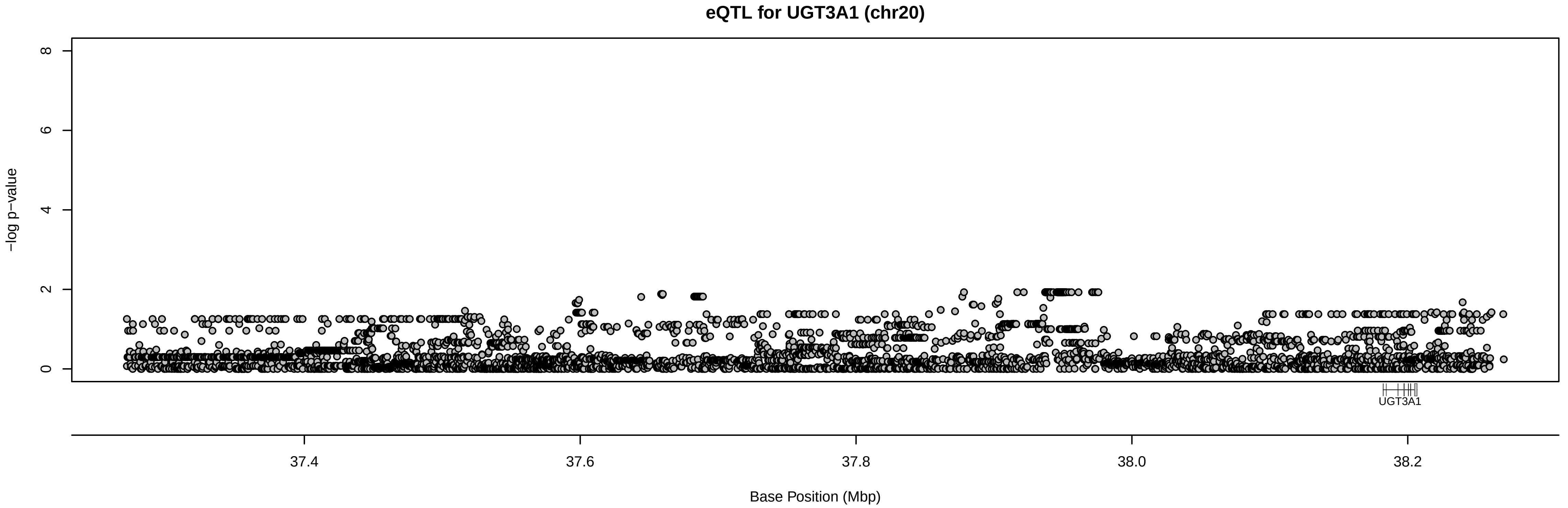
eQTL for UGT3A1 (chr20)
−log p−value
0
2
4
6
8
UGT3A1
37.4
37.6
37.8
38.0
38.2
Base Position (Mbp)

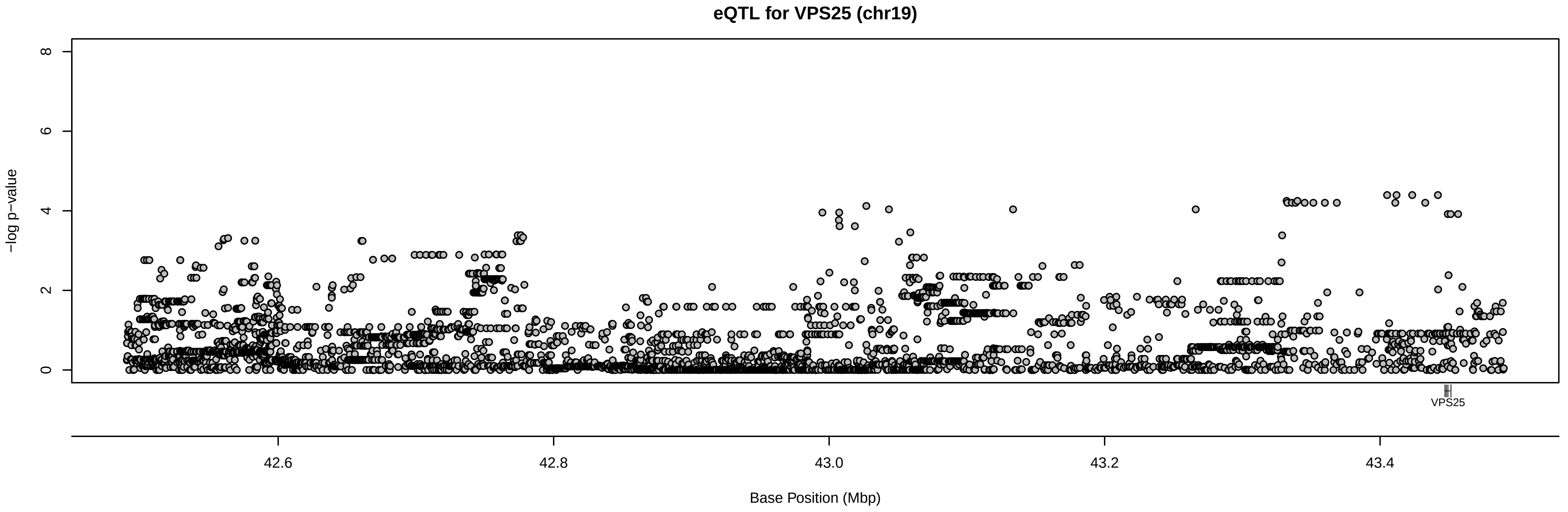
eQTL for VPS25 (chr19)
−log p−value
0
2
4
6
8
VPS25
42.6
42.8
43.0
43.2
43.4
Base Position (Mbp)

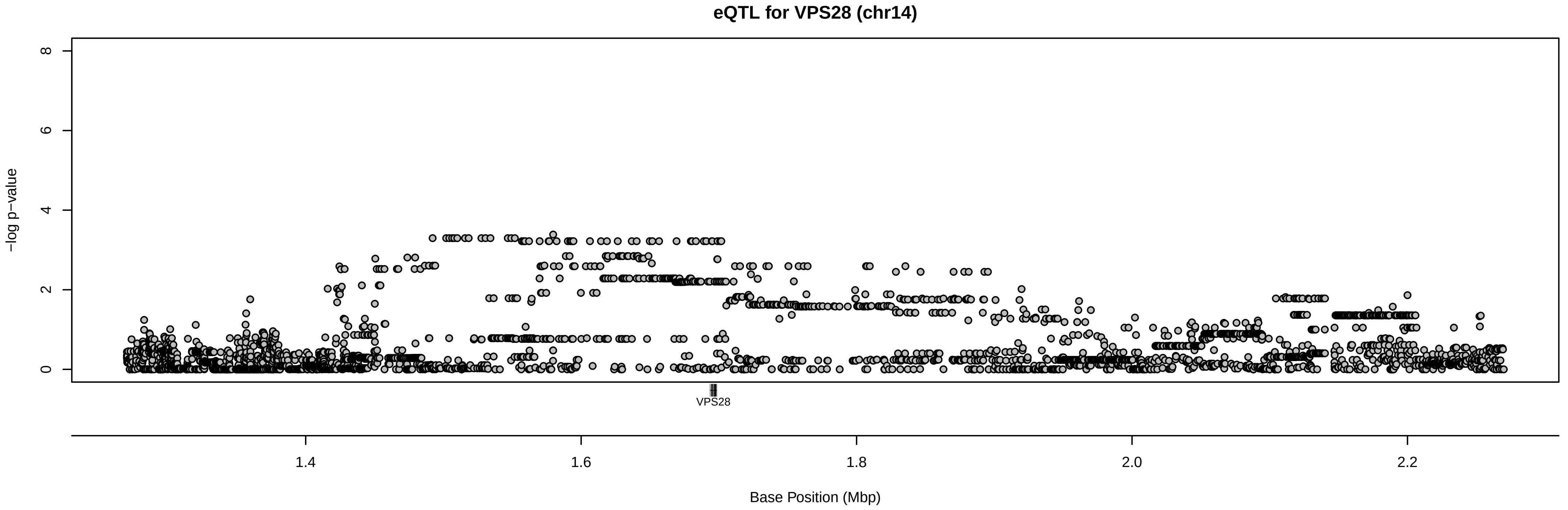
eQTL for VPS28 (chr14)
−log p−value
0
2
4
6
8
VPS28
1.4
1.6
1.8
2.0
2.2
Base Position (Mbp)

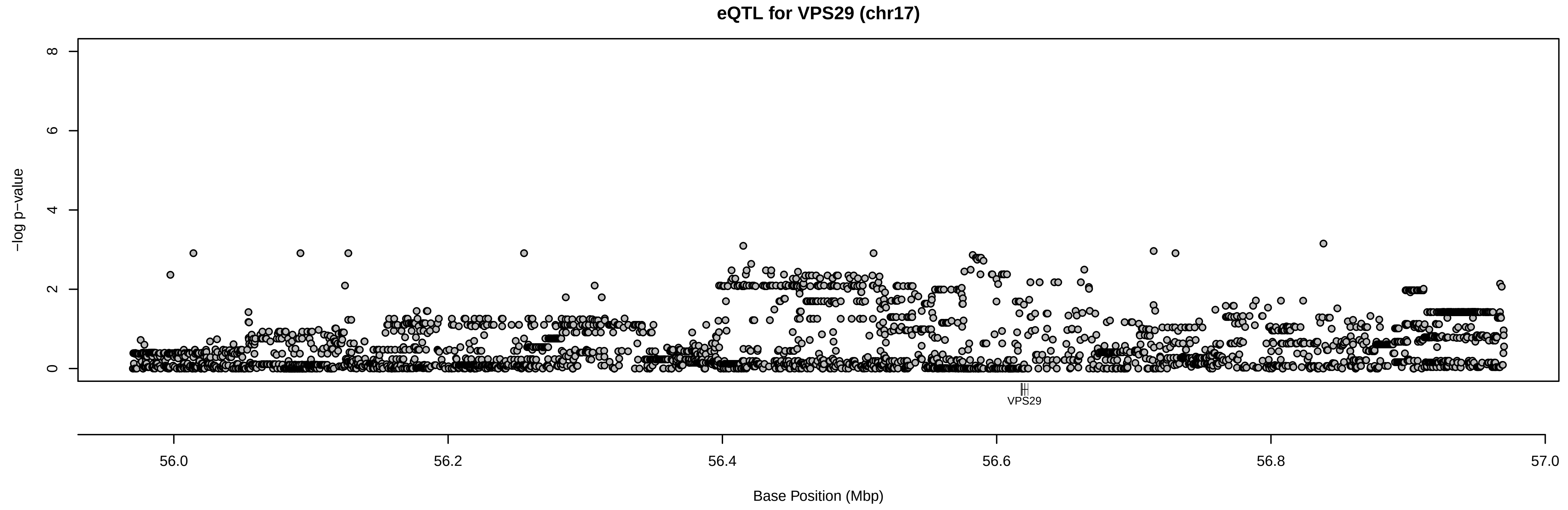

eQTL for VPS29 (chr17)
−log p−value
0
2
4
6
8
VPS29
56.0
56.2
56.4
56.6
56.8
57.0
Base Position (Mbp)

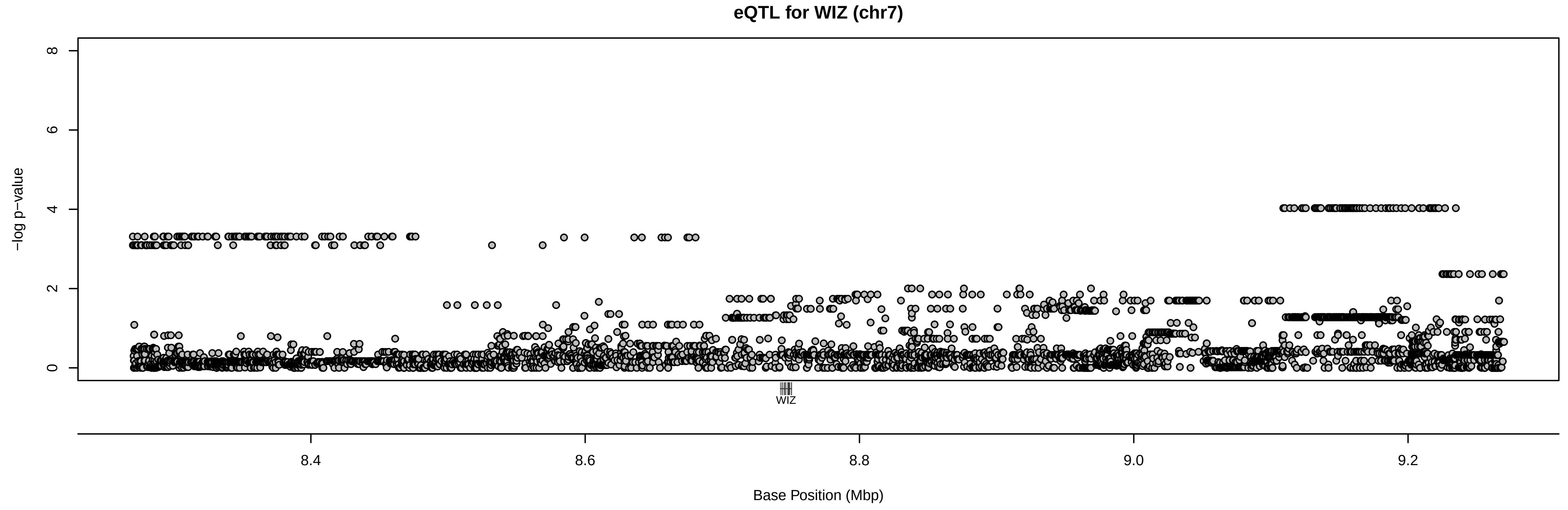

eQTL for WIZ (chr7)
−log p−value
0
2
4
6
8
WIZ
8.4
8.6
8.8
9.0
9.2
Base Position (Mbp)

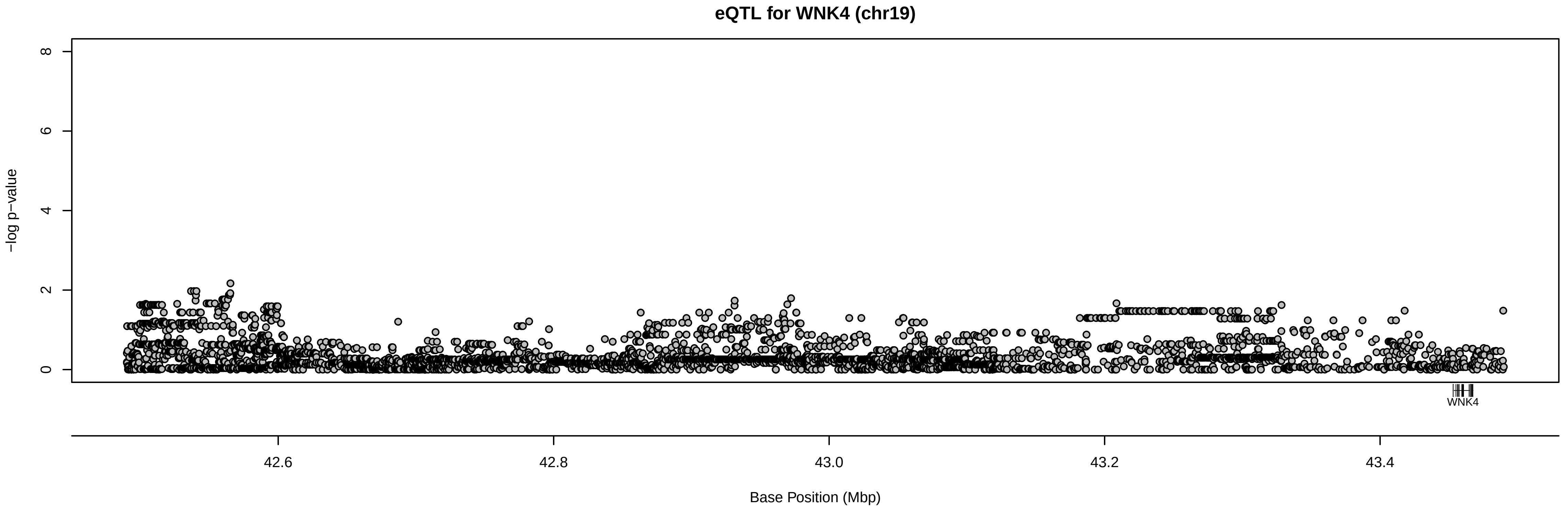
eQTL for WNK4 (chr19)
−log p−value
0
2
4
6
8
WNK4
42.6
42.8
43.0
43.2
43.4
Base Position (Mbp)

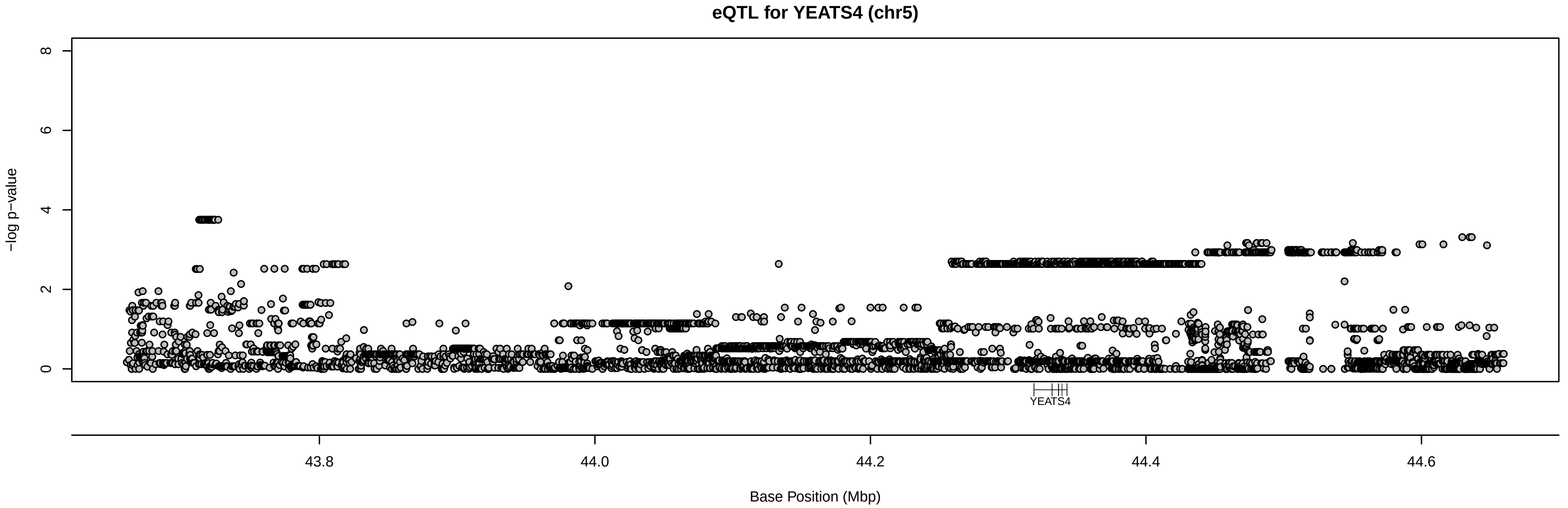
eQTL for YEATS4 (chr5)
−log p−value
0
2
4
6
8
YEATS4
43.8
44.0
44.2
44.4
44.6
Base Position (Mbp)

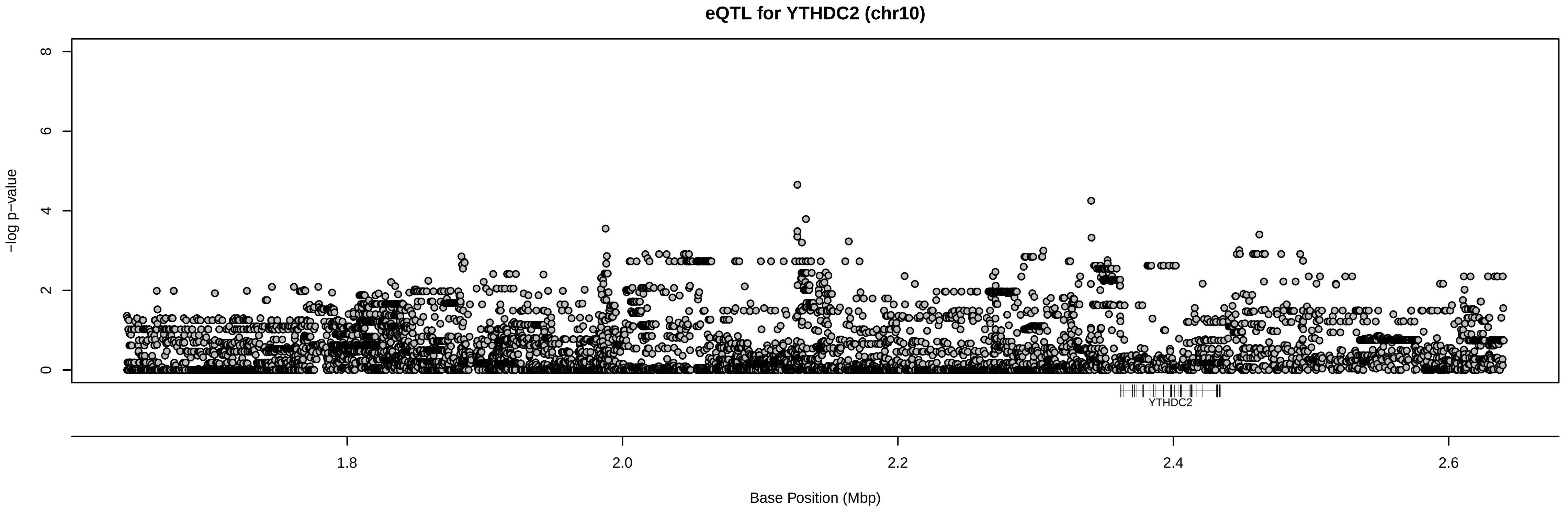
eQTL for YTHDC2 (chr10)
−log p−value
0
2
4
6
8
YTHDC2
1.8
2.0
2.2
2.4
2.6
Base Position (Mbp)

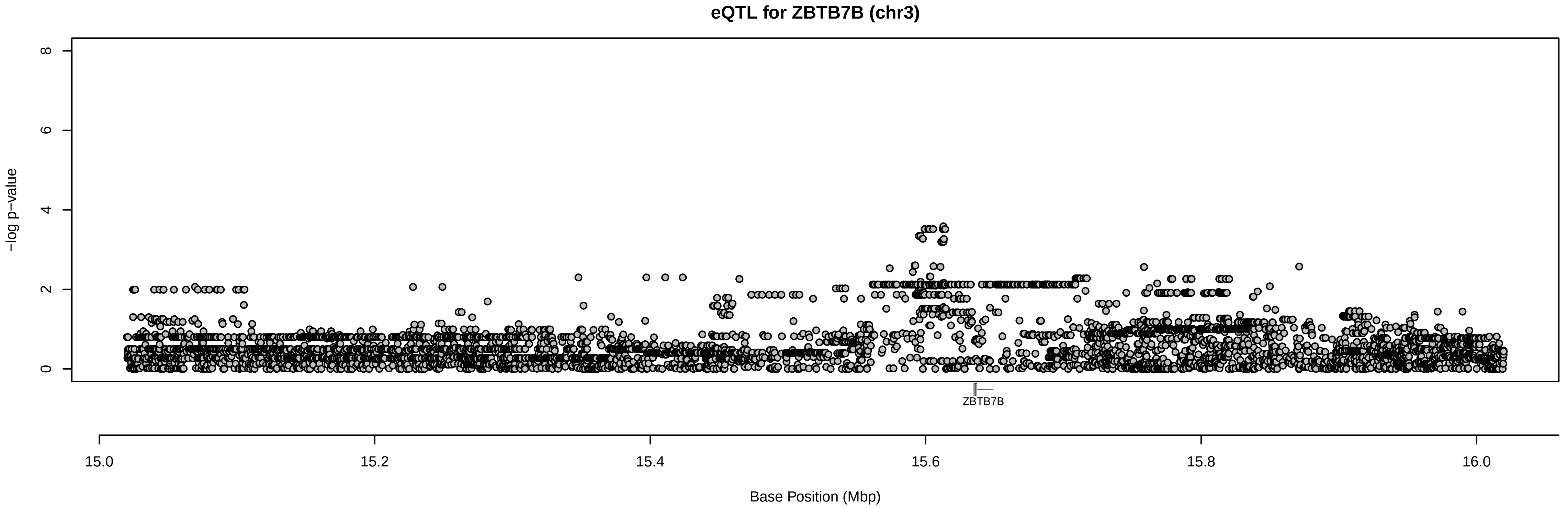
eQTL for ZBTB7B (chr3)
−log p−value
0
2
4
6
8
ZBTB7B
15.0
15.2
15.4
15.6
15.8
16.0
Base Position (Mbp)

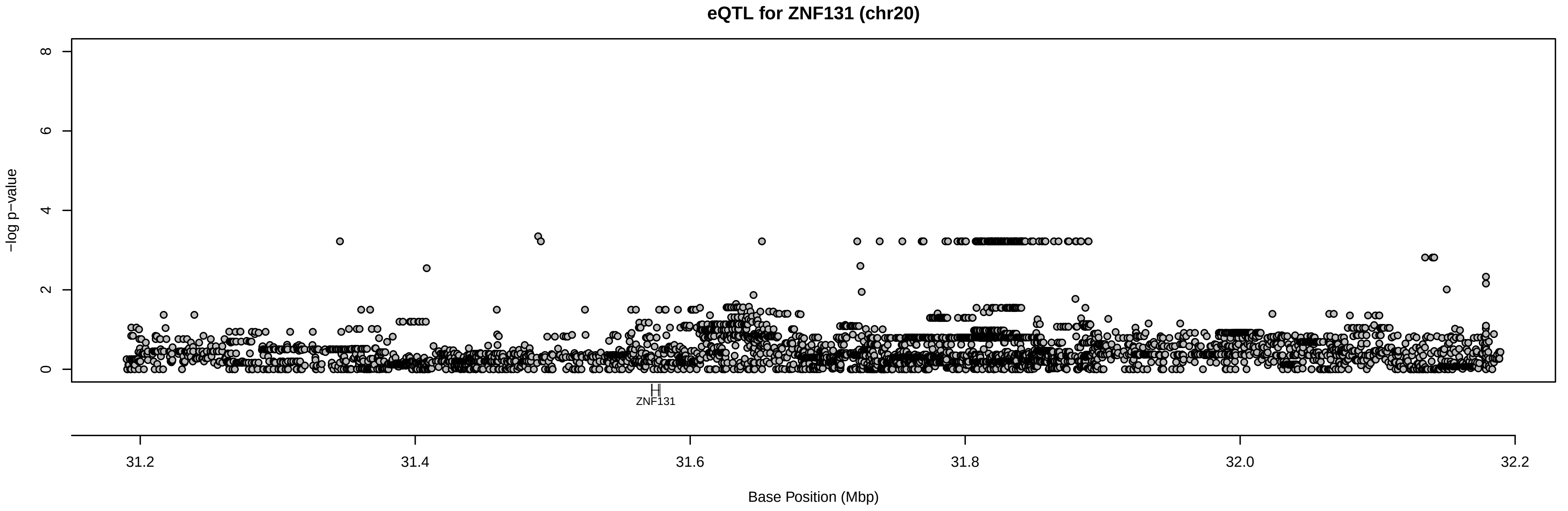
eQTL for ZNF131 (chr20)
−log p−value
0
2
4
6
8
ZNF131
31.2
31.4
31.6
31.8
32.0
32.2
Base Position (Mbp)

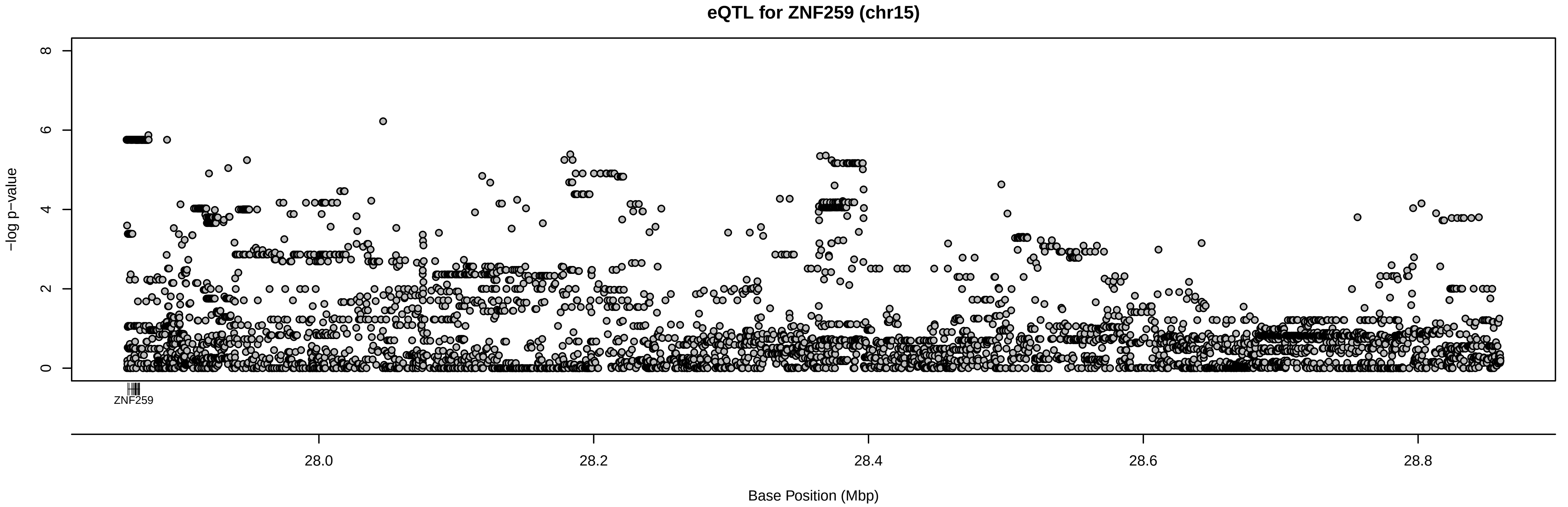
eQTL for ZNF259 (chr15)
−log p−value
0
2
4
6
8
ZNF259
28.0
28.2
28.4
28.6
28.8
Base Position (Mbp)

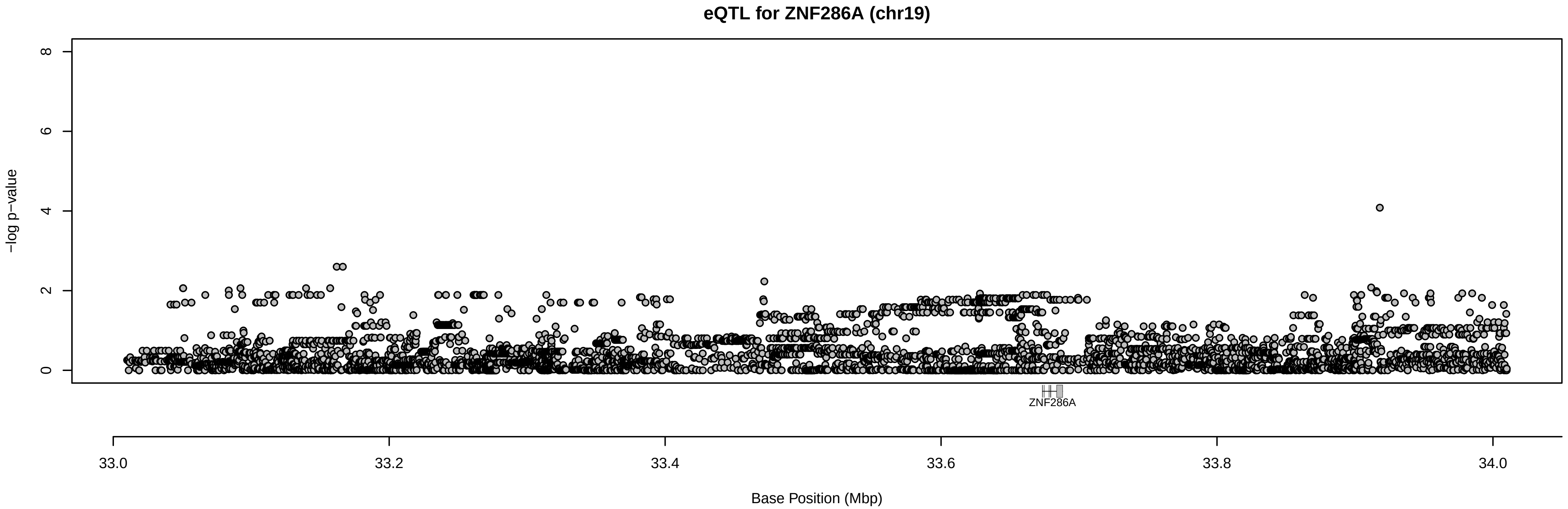

eQTL for ZNF286A (chr19)
−log p−value
0
2
4
6
8
ZNF286A
33.0
33.2
33.4
33.6
33.8
34.0
Base Position (Mbp)

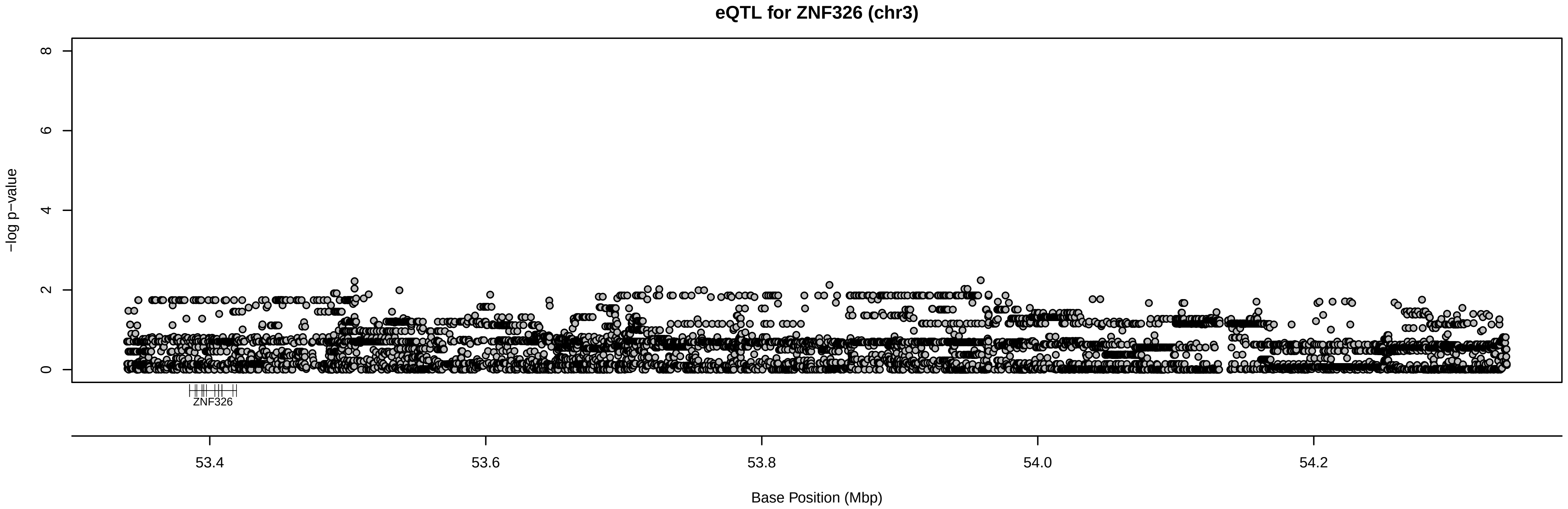

eQTL for ZNF326 (chr3)
−log p−value
0
2
4
6
8
ZNF326
53.4
53.6
53.8
54.0
54.2
Base Position (Mbp)

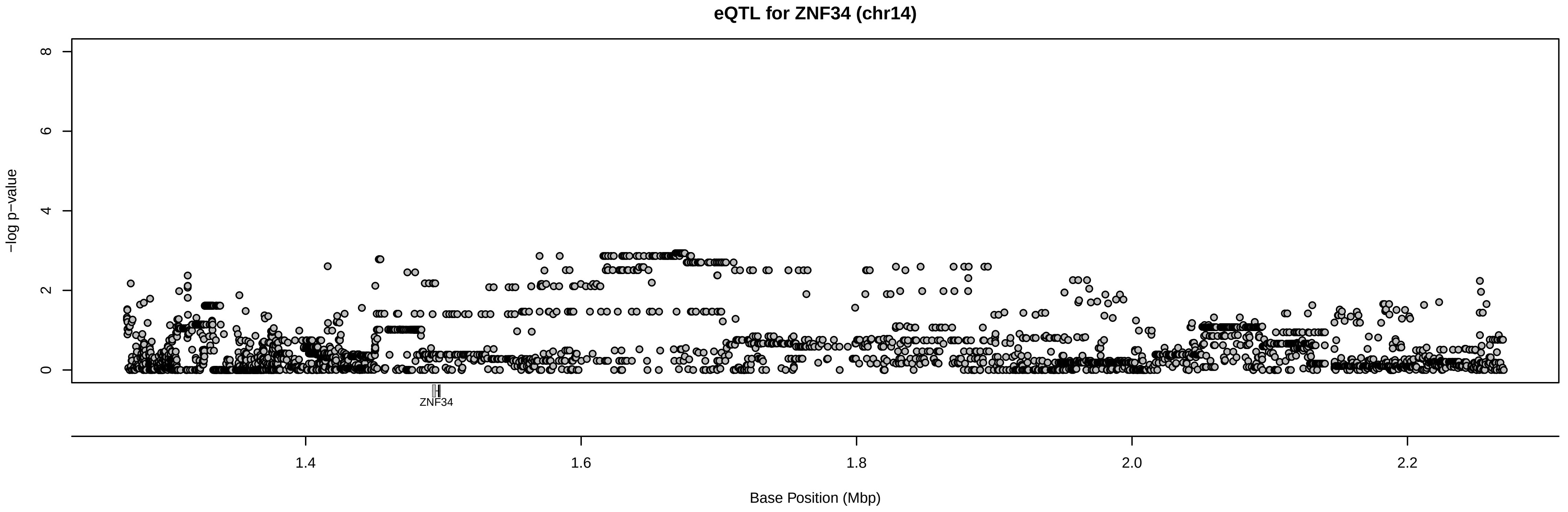
eQTL for ZNF34 (chr14)
−log p−value
0
2
4
6
8
ZNF34
1.4
1.6
1.8
2.0
2.2
Base Position (Mbp)

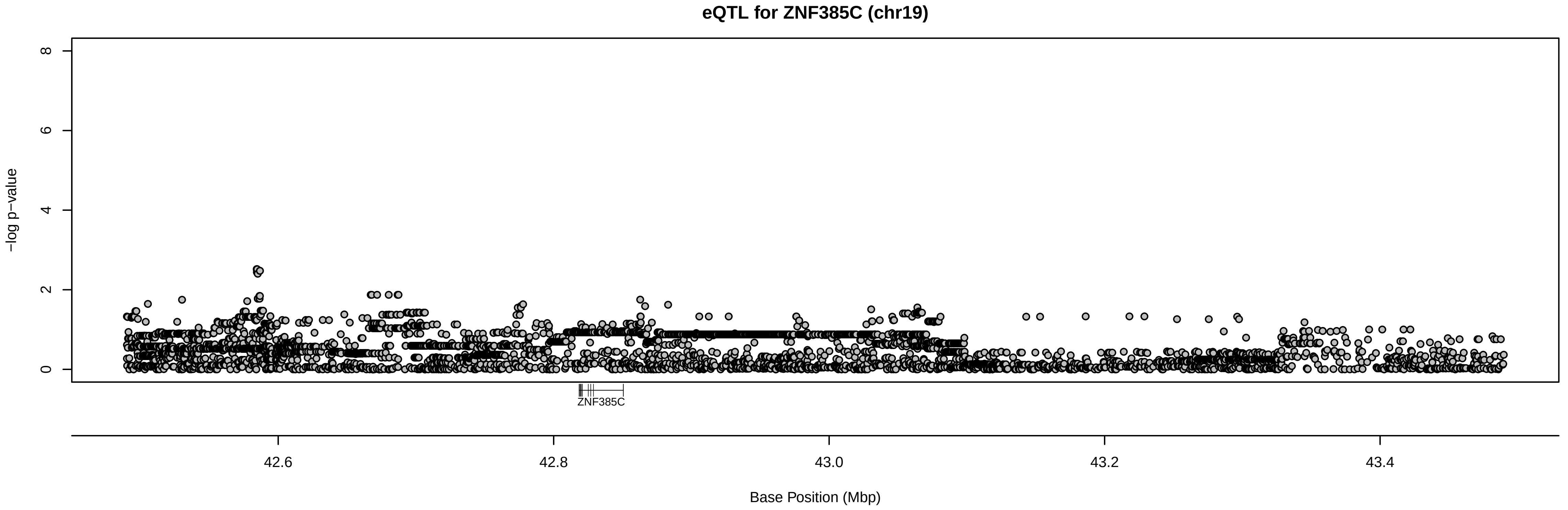
eQTL for ZNF385C (chr19)
−log p−value
0
2
4
6
8
ZNF385C
42.6
42.8
43.0
43.2
43.4
Base Position (Mbp)

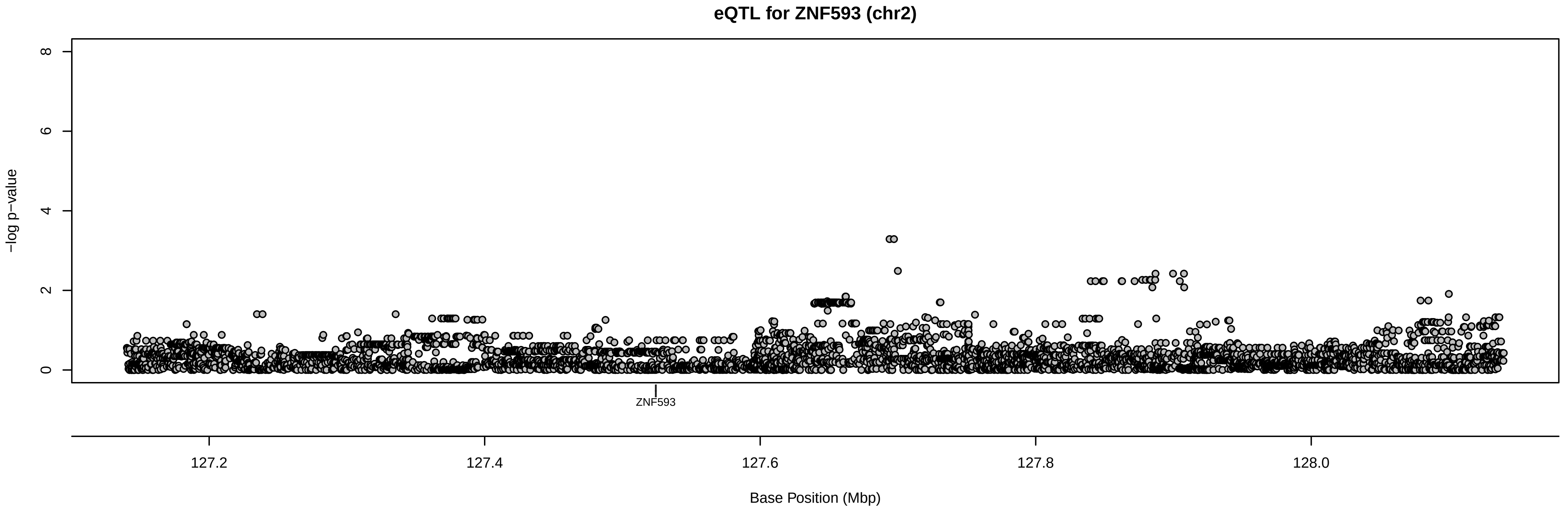
eQTL for ZNF593 (chr2)
−log p−value
0
2
4
6
8
ZNF593
127.2
127.4
127.6
127.8
128.0
Base Position (Mbp)

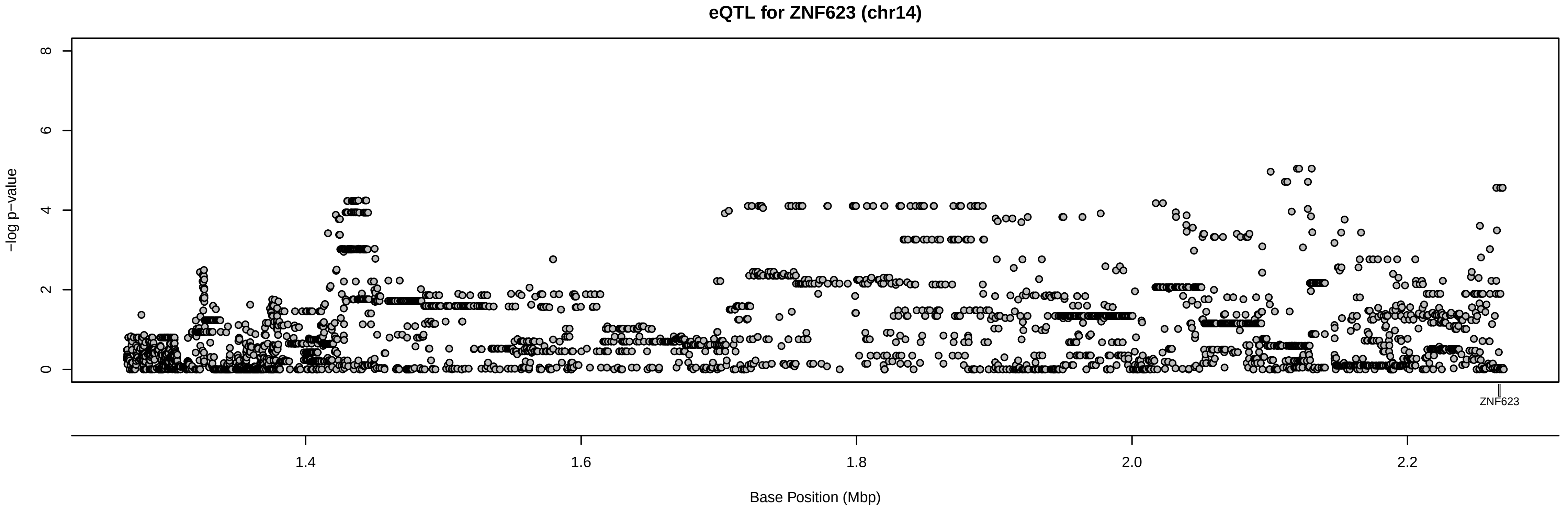
eQTL for ZNF623 (chr14)
−log p−value
0
2
4
6
8
ZNF623
1.4
1.6
1.8
2.0
2.2
Base Position (Mbp)

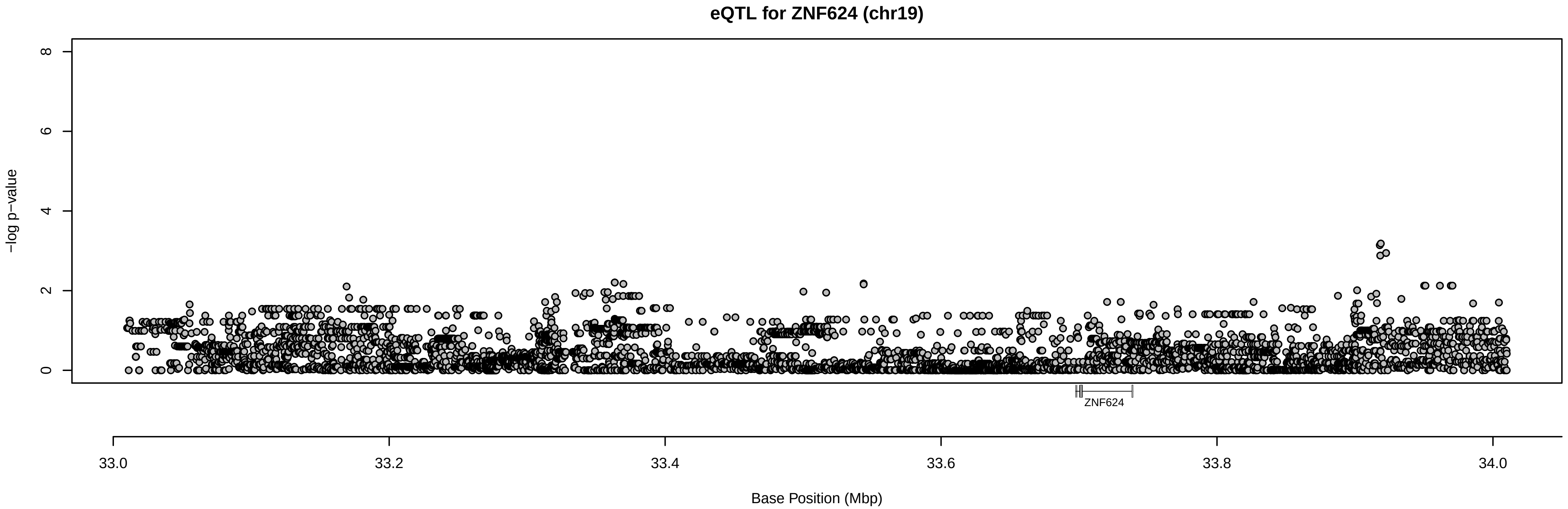
eQTL for ZNF624 (chr19)
−log p−value
0
2
4
6
8
ZNF624
33.0
33.2
33.4
33.6
33.8
34.0
Base Position (Mbp)

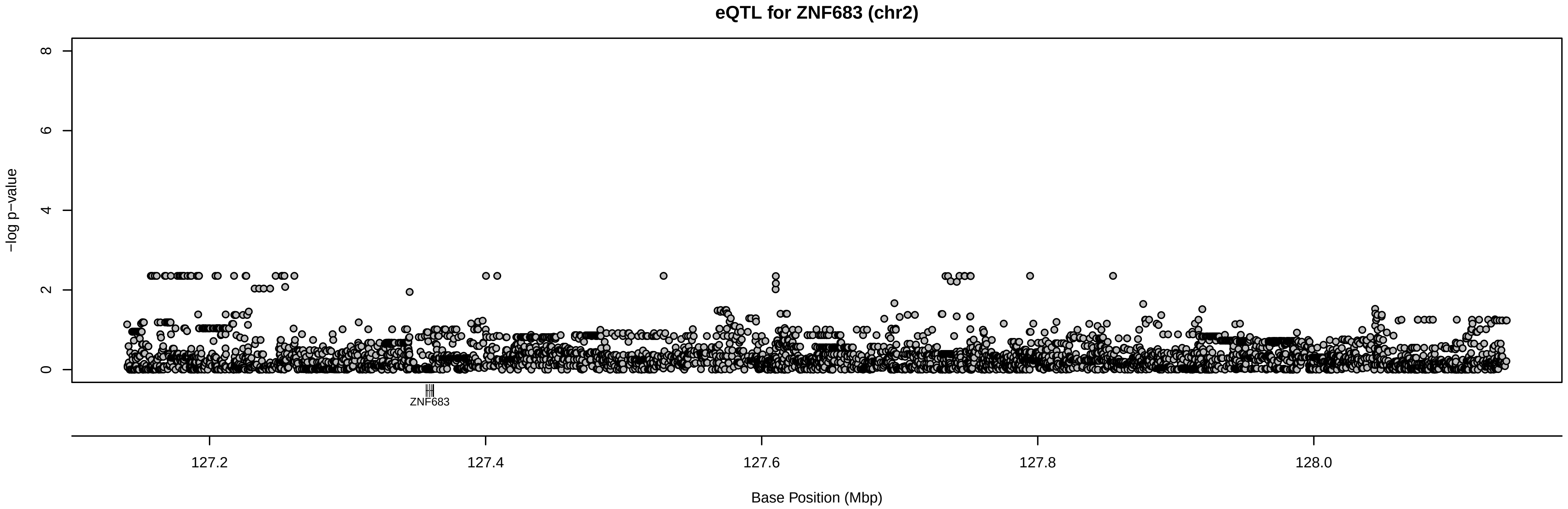
eQTL for ZNF683 (chr2)
−log p−value
0
2
4
6
8
ZNF683
127.2
127.4
127.6
127.8
128.0
Base Position (Mbp)

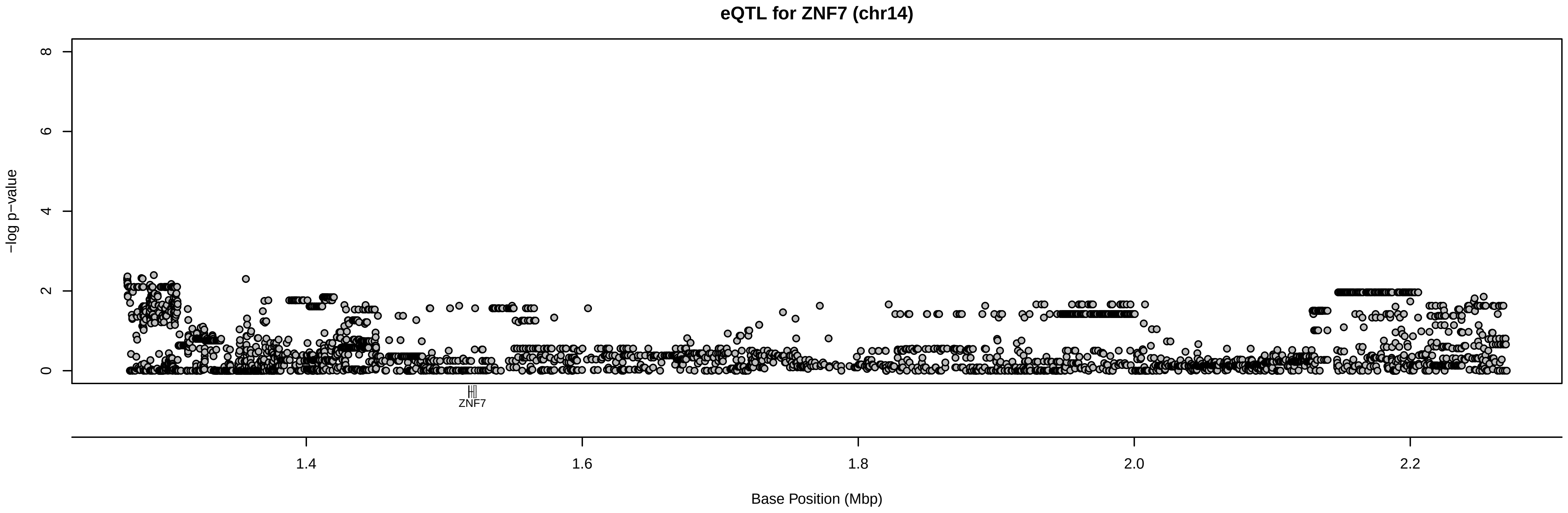

eQTL for ZNF7 (chr14)
−log p−value
0
2
4
6
8
ZNF7
1.4
1.6
1.8
2.0
2.2
Base Position (Mbp)
